# Computer Vision for Kinetic Analysis of Lab- and Process-Scale Mixing Phenomena

**DOI:** 10.1021/acs.oprd.2c00216

**Published:** 2022-11-04

**Authors:** Henry Barrington, Alan Dickinson, Jake McGuire, Chunhui Yan, Marc Reid

**Affiliations:** † Department of Pure & Applied Chemistry, University of Strathclyde, Royal College Building 204 George Street, Glasgow G1 1XW, U.K.; ‡ Colorants Technology Centre, FUJIFILM Imaging Colorants, Earls Road, Grangemouth FK3 8XG, U.K.

**Keywords:** mixing, computer vision, kinetics, imaging

## Abstract

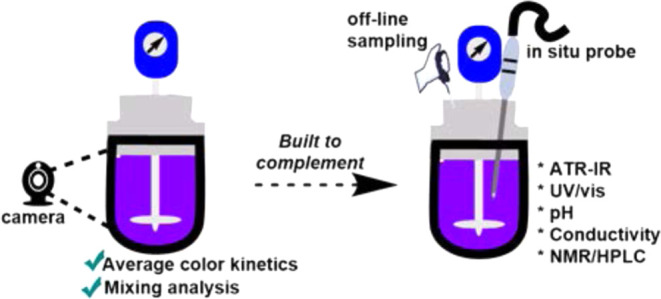

A software platform for the computer vision-enabled analysis of mixing phenomena of relevance to process scale-up is described. By bringing new and known time-resolved mixing metrics under one platform, hitherto unavailable comparisons of pixel-derived mixing metrics are exemplified across non-chemical and chemical processes. The analytical methods described are applicable using any camera and across an appreciable range of reactor scales, from development through to process scale-up. A case study in nucleophilic aromatic substitution run on a 5 L scale in a stirred tank reactor shows how camera and offline concentration analyses can be correlated. In some cases, it can be shown that camera data hold the power to predict reaction progress.

## 1 Introduction

### 1.1 Impact of Mixing

Mixing is crucial to many process scale-up projects.^1−4^ It affects a diverse range of chemically intensive processes such as phase-transfer catalysis,^5−8^ additive manufacturing,^9−11^ fuel combustion,^12^ flow chemistry,^13^ powder formulation,^14,15^ biotechnology,^16^ and (most pertinent to this paper) myriad reaction scale-ups in tank reactors.^16−20^ Indeed, it is well established that mixing in tank reactors is subject to micro-, meso-, and macro-mixing phenomena, all of which are sensitive to the chemistry, temperature, reactor geometry, and impeller shape, among other parameters.^21^ Mixing represents one of the most effective, non-chemical means of process improvement. From an economic perspective, mixing issues have been cited as leading to losses of $1–10 billion (US).^22,23^ In relation, mixing deficiencies have also been reported at the root cause of fatal accidents.^24^

Despite clear need for the chemical engineering community to maintain high-quality education and awareness of mixing challenges,^25,26^ it can be argued that mixing phenomena are less often considered in chemistry education.^25,27^ Mixing requirements are often neglected in small-scale chemical method development projects, speculatively due to the reasonable assumption that mixing is generally efficient on the small scale. Conversely, mixing phenomena are more obvious and critical (from both a safety and economic perspective) when reactions are scaled up. Going further, a comparative lack of analytical tools with which to measure mixing on small, developmental scales may render the topic of mixing one that is a more engineering-focused consideration.

### 1.2 Mixing Analysis

The cross-sector and cross-scale importance of mixing has driven the development of a wealth of analytical methods through which to quantify it.^28,29^ These methods include tomography,^20,30^ near-infrared imaging,^31^ fluorescence,^32^ and acoustics,^33^ among others.^28,29^ From the range of analytical methods explored in mixing analysis, visible-range imaging and computer vision approaches are attractive on account of being non-contact, spatially resolved (by area), flexible in resolution (to enable study of macro-, meso-, and microscale mixing phenomena), of low cost, and requiring minimal departure from how the reactions would routinely be setup. Fundamentally, computer vision is the quantification of visually informative phenomena using the camera technology and computer algorithms.^34^ Few time-resolved analytical technologies are applicable on both small and large scales. Camera-enabled analytics have the potential to enable more time-, cost-, and safety-effective monitoring of high-value chemical processes compared to more commonplace in situ analytical methods. Video analysis, in particular, holds potential for analyzing wide-ranging chemical phenomena in the laboratory, on plant, in batch, and in flow.^35^

In the chemical engineering space, much has been accomplished using grayscale imaging in powder mixing.^14,15,36−42^ These efforts have attended applications to quantify powder concentrations, homogeneity, surface texture, and overall mixing time. On the breadth of applications, domains spanning pharmaceutical formulation, food mixing, and construction have benefited from these innovations in mixing analysis. An exemplary case of grayscale imaging applied to tank reactor mixing analysis was reported by Fitschen and co-workers, wherein pH titrations served to build detailed and data-rich monitoring methods to capture both local and global mixing phenomena, validating imaging data interpretations with computational fluid dynamics simulations.^2^ Beyond grayscale, full color analysis has enabled the investigation of particle mixing in rotary drums^43^ and soft-elastic reactors.^26^ Further still, hyperspectral imaging has helped quantify pharmaceutical powder blends.^44^

### 1.3 Problem

At Fujifilm, where the industrial portion of this collaborative work resided, most process development for full-scale manufacture (10–20 tonnes) takes place in glass reactors that are small-scale replicas of plant reaction vessels. These mimic kits range from approximately 2.5–10 L in size and are oftentimes equipped with various process analytical technology (PAT) probes. During our collaborative academia-industry discussions, the emerging video analysis platform described herein offered the potential to provide non-contact mixing analysis to ensure that any PAT probes used in the vessels (for more specific molecular analyses) had not significantly disrupted the intended mixing profile of the reaction. For the aforementioned safety and economic reasons, achieving realistic mixing in the process-scale laboratory, before final scale-up to a plant campaign, is of paramount importance. Additionally, with some experiments lasting longer than a normal working day, the camera technology described herein offered an opportunity to explore a means of tracking any temporary loss of mixing, when other PAT data may be unreliable or not applicable on such a timescale.

### 1.4 Aims

Our present contribution centers on the developmental software, Kineticolor, that enables users to quantify average color changes of a reaction bulk—for any size of non-opaque vessel—as a function of time, using any camera. The same software allows the user to select different regions of interest, providing kinetic information as a function of space and time. Building on above-cited innovations in imaging-enabled mixing analysis, our platform enables analysis with either grayscale, or full color images, enables both pixel-averaged and spatially resolved analysis, and captures disparately reported texture analysis techniques under one platform.

**Scheme 1:**
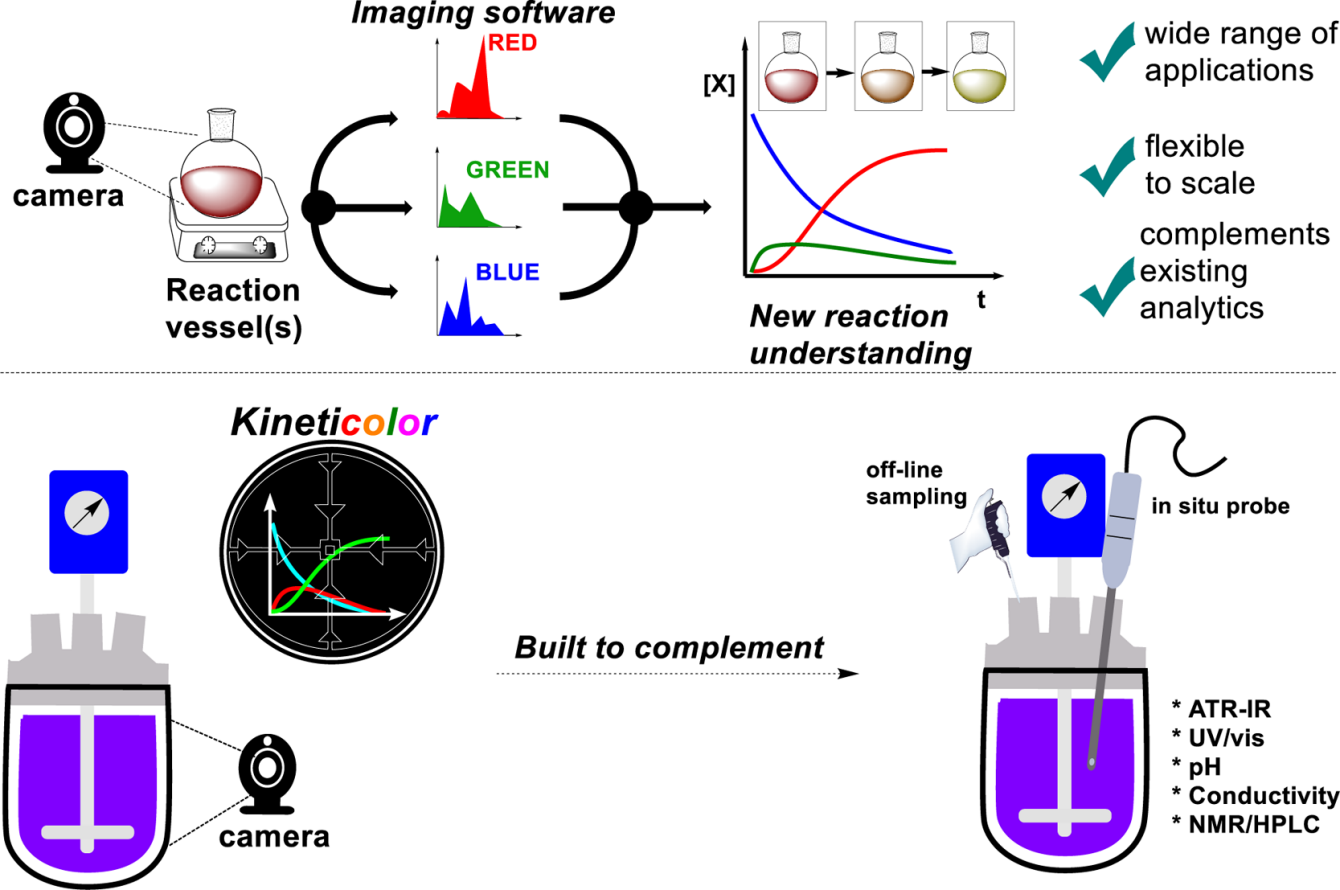
Kineticolor Overview and Its Complementarity to Known Reaction Monitoring Tools While more common techniques capture molecular specifics, Kineticolor captures bulk-level visible information.

Kineticolor provides a rare and chemistry-agnostic example of a non-contact analytical tool that can provide quantifiable insights on reaction bulk, complementing the large suite of more specific analytical tools (mainly in situ probes) used to analyze small- and intermediate scale reaction systems (Scheme 1). The tool has been developed for end users in small-scale chemical development and intermediate-scale process chemistry and collaboratively designed with industrial chemists working in these domains.

Following earlier applications of this technology in small-scale electrochemical reaction development^45^ and palladium catalyst degradation kinetics,^46^ the present study focused on extending the application of Kineticolor to mixing analysis. In other words, where previous studies focused primarily on overall reaction bulk and color averaging, the present study more deeply explored spatially resolved image analysis methods. The intention here was to provide a color-based language through which to quantify mixing in a way that gives translatable decision-making power to chemists and chemical engineers alike. It is important to note that this investigation was focused on providing intuitive visualization of mixing progress over time, not on the simulation of detailed, mathematically rigorous mixing models.

From the aforementioned imaging innovations in mixing analysis,^2,14,15,25−29,31,37−41,43,44,47−49,49−55^ most of these are disparately reported, differently encoded, and not comparable within a single platform. Herein, we employ a series of mixing case studies to compare analysis derived from the core Kineticolor platform, tracking an averaged color region over time, as well as newly integrated and spatially resolved mixing metrics.

## 2 Methodology

### 2.1 Model Development

To build on existing Kineticolor developments and incorporate mixing analysis functionality, we first reviewed and encoded a series of six mixing metrics into the Kineticolor platform. To enable comparison of these metrics, each was calculated based on the same region of interest and same selection of video frames for all reaction videos later analyzed. These metrics are described below.

#### 2.1.1 Contact

**Scheme 2:**
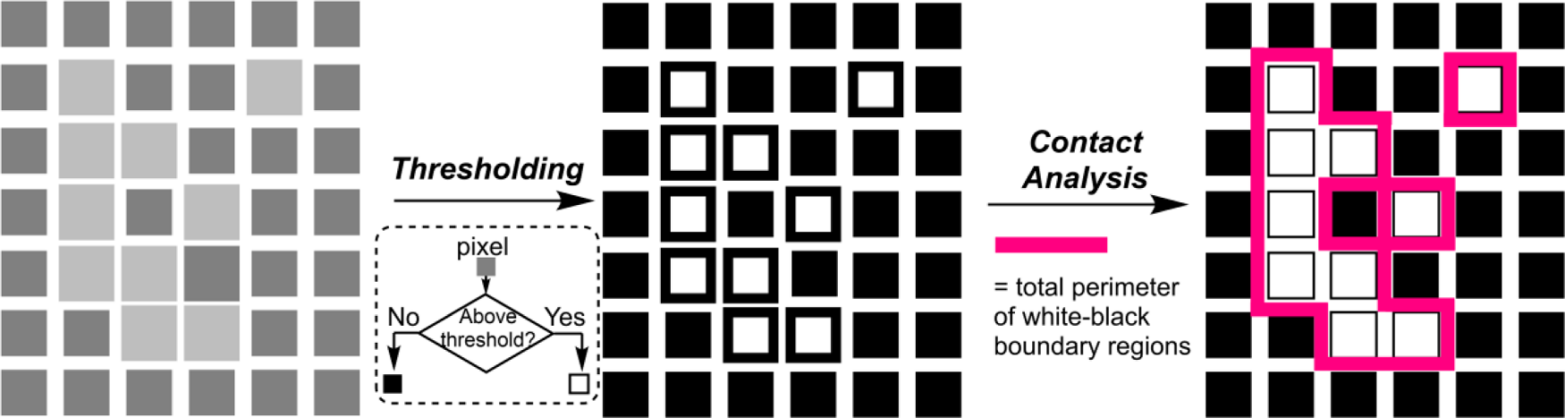
Conceptual Representation of Contact Analysis of Mixing

Inspired by the teams of Rodrigue and Lui on the analysis of mixing in rotary drums, we developed a new encoding for the so-called contact analysis.^36,43^ Each frame is converted into a binary image; each pixel is made either white or black, depending on whether they are brighter or darker than a user-selected grayscale threshold. The perimeter between white and black pixels is then calculated, that is the amount of contact between the white and black regions is summed (see Scheme 2).

The contact value has local minima both before and after mixing as both unmixed and mixed solutions often possess some uniformity in color. In other words, more uniform mixtures have fewer measurable regions of black and white pixels. However, during the mixing transition, there will be both mixed and unmixed regions, which will produce a higher contact value. Artefacts within the image can also affect the starting and ending magnitudes of the contact metric (see below). Since the user can set the grayscale threshold specifically, this metric can be used to detect small but distinct spatial effects, within the selected region of interest in the video selected for analysis.

#### 2.1.2 Gray-Level Co-Occurrence Matrices

**Scheme 3:**
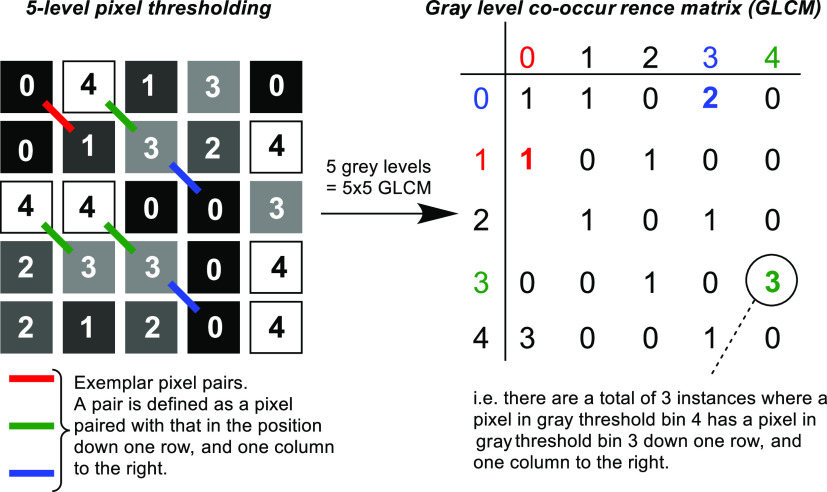
Conceptual Representation of a Gray-Level Co-Occurrence Matrix Left: a simplified representation of pixels in a selected region of interest. Right: the resulting GLCM based on the defined pixel pairings in the original video frame.

From the teams of Haralick^56^ and Rodrigue,^36^ a gray-level co-occurrence matrix (GLCM) has been used to assess texture properties of powders in mixing drums. For each selected region of interest, within each video frame, a matrix is produced, according to the gray level of each pixel within a set of pixel pairs. The gray level is simply the grayscale value of each pixel, split into groups, or levels, defined by user-determined thresholds.^51^ The number of gray levels used determines the size of the GLCM produced; for *N* gray levels, an *N* × *N* matrix is made (Scheme 3, right). Therefore, when the pixels in a pair belong to levels *i* and *j*, respectively, the matrix element *ij* is increased by one. The relative pixel grid position of the pixel pairs is defined before analysis. For example, in this report, all pixels in the grayscale image frames are paired with the pixel in the position that is one to the right and one down in the frame (Scheme 3, left). The GLCMs are used to calculate three derived mixing metrics, described below.

**Scheme 4:**
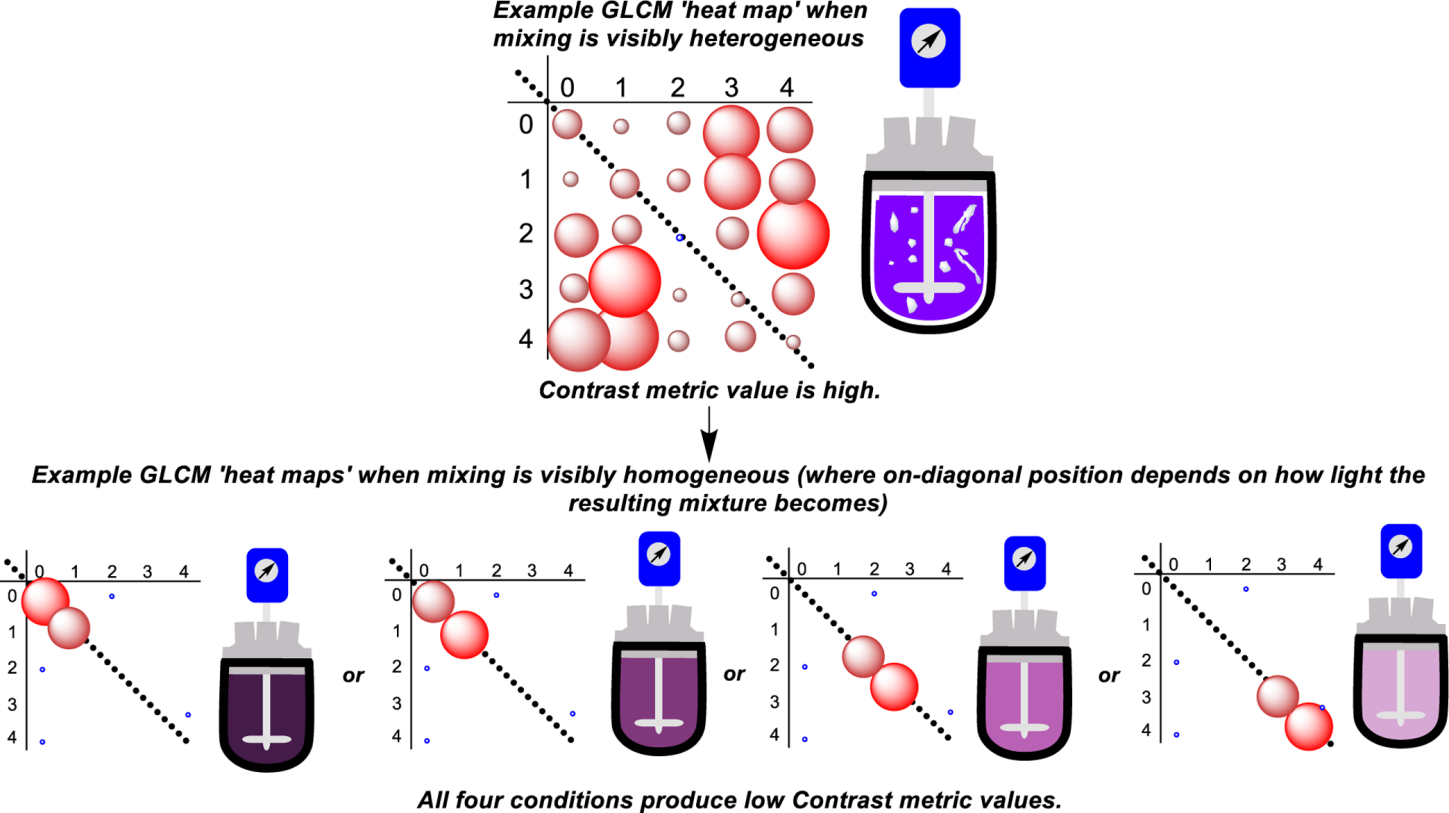
Conceptual Representation of the Contrast Metric Derived from a Gray-Level Co-Occurrence Matrix Top: visibly heterogeneous mixtures will produce more off-diagonal pixel pairs that have higher differences in the gray level. Bottom: different diagonalized GLCM conditions that satisfy a more homogeneous state relative to the case shown at the top.

The contrast metric is calculated from the GLCM using eq 1
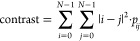
1
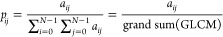
2
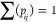
3where *i* and *j* are the GLCM matrix row and column indices (not the element values). *p*_*ij*_ is the normalized element value at position (*i,j*) of the GLCM, as expressed in eq 2, where each element value, *a*, in the GLCM is divided by the grand sum of all elements in the GLCM. This normalization ensures that all GLCM elements sum to unity; eq 3. The contrast metric represents the magnitude of gray-level contrast for the ensemble of pixel pairs. Contrast is highest when mixing is most visibly heterogeneous, and the GLCM off-diagonal values are highly populated (Scheme 4, top). As mixing progresses toward visible homogeneity, fewer pixel pairs with large differences in the gray level will exist (e.g., there will be fewer pixels in bin 4 paired with pixels in bin 1). At the same time, as mixing evolves, more and more pixel pairs from the same gray level bin will emerge (Scheme 4, bottom). The calculated contrast will thus decrease, tending toward a minimum value of zero.

A second mixing metric derived from the GLCM is homogeneity (*H*), quantifying how close the GLCM is to a diagonal matrix, calculated using eq 4
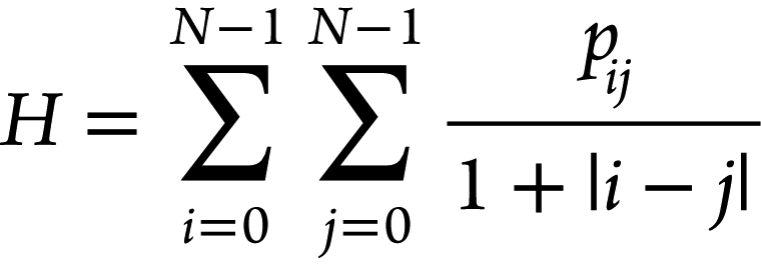
4

Homogeneity captures the similarity of color across an image. The more the distribution of pixels is similar in color, the higher the *H* value. The contrast metric has been considered to be broadly more useful than homogeneity since contrast is more easily compared to the contact metric than homogeneity. We consider *H* in this study for completeness.

Angular second moment (ASM),^56^ also known as energy,^36,57^ is the third metric we considered, as derived from the GLCM. It is defined in eq 5
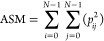
5

ASM describes the amount of “block color” in an image. Completely random noise would produce the lowest ASM value, while a single block color would produce the highest. Similarly, gradient color will produce a relatively low ASM value, while a checkerboard or regular polka-dot pattern would produce a relatively high ASM value (see intuition-building examples below).

**Scheme 5:**
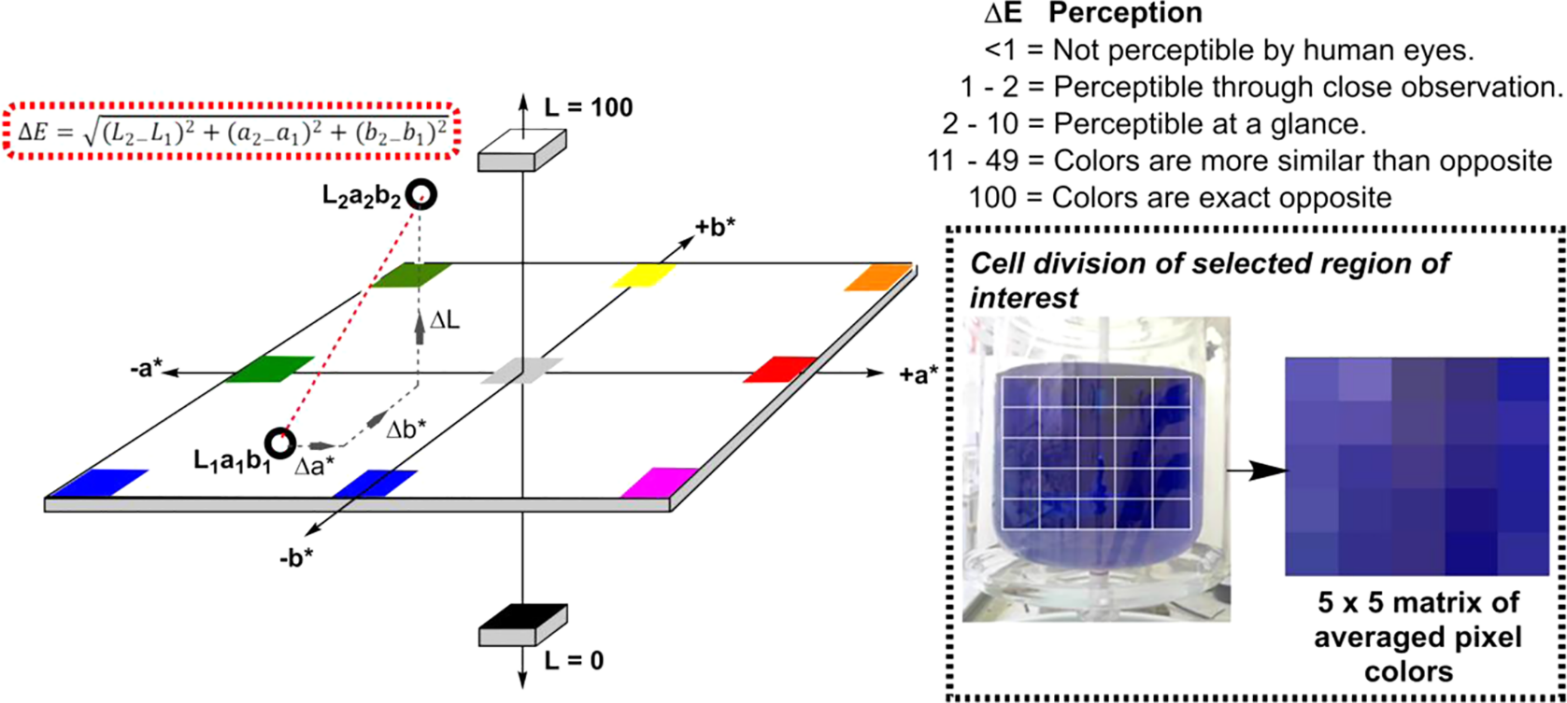
Left: Conceptual Depiction of the CIE-L*a*b* Color Space, wherein the Euclidean Distance between Two Colors (Open Circles) is Defined as Δ*Ε* (Red Dashed Line) Right: exemplar cell-based portioning of a reactor captured in a video frame, leading to a matrix of averaged pixel regions to be analyzed over time. Each cell is analyzed as an average by row, by column, and individually. The same cell-based approach is applied to calculate variance of any one color space component across the cells.

Beyond texture analysis-inspired metrics, additional metrics were defined based on the averaged analysis of portioned sections of each video frame (Scheme 5, right). We herein more deeply explore the Δ*Ε* (or delta *E*) metric, derived from the CIE-L*a*b* color space. In its simplest form, Δ*Ε* is the Euclidean (“straight line”) distance between one color and a reference color across the CIE-L*a*b* space. Each color defining Δ*Ε* is represented by its 3D coordinates in the CIE-L*a*b* space, as per Scheme 5 (left). In practice, Δ*Ε* serves as a color-agnostic measure of contrast over time, as measured relative to the color recorded at time zero. In earlier work, we applied *ΔΕ*-time profiles to effectively capture productive and degradative processes in palladium-catalyzed reactions.^46^ The same metric has found limited applications in powder mixing analysis using a progressive single image analysis approach.^52,53^ Here, we enable full video-based Δ*Ε* analysis, both as a grand average of all color progress across the entire bulk of a vessel and as a spatially resolved cell-based analysis to capture meso-mixing phenomena in an intuitive manner. By the same cell-based method, the variance in average color between cells can be calculated, with lower variance values likely to indicate more homogeneous mixing.

**Scheme 6:**
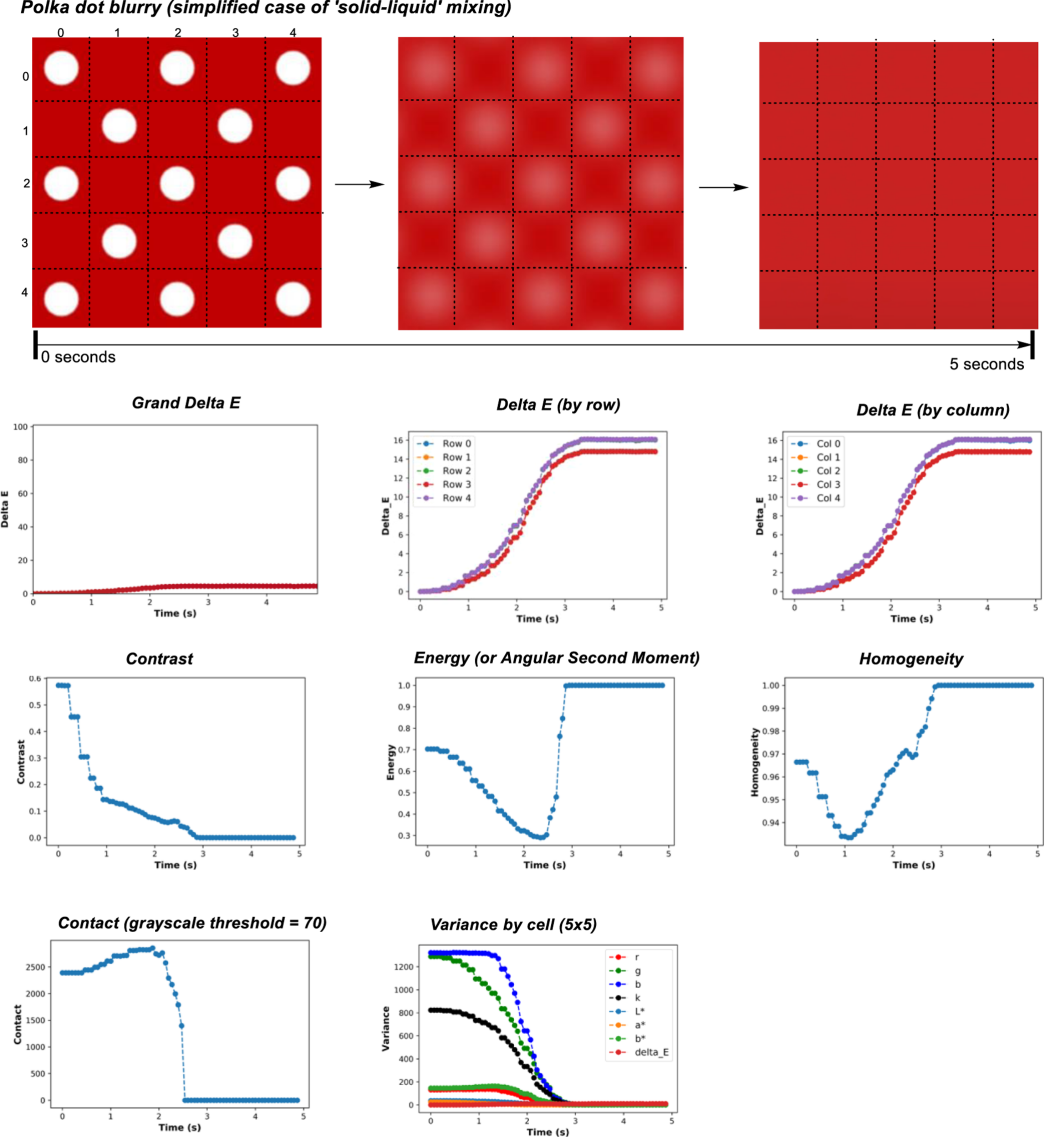
Top: a Five-Second Video of a Polka-Dot Pattern Undergoing Non-Linear Camera Blur Dashed lines on the video frames represent row, column, and cell segmentation used during spatially resolved Δ*Ε* analysis. Bottom: mixing analysis of the video using the Δ*Ε*, contrast, ASM (or energy), homogeneity, contact, and variance.

Before investigating this collective approach to mixing analysis through the Kineticolor platform, we analyzed a simplified, intuition-building video simulation of a polka-dot pattern blurring over time (Scheme 6). Analyzing the averaged pixels from the entire selected region of interest, the grand (or averaged) Δ*Ε* over time evidenced a maximum Δ*Ε* of ∼4, representing a very subtle contrast change over time, barely registerable by the eye. Our earlier, catalysis-focused investigation captured subtle and obvious color contrast changes spanning *ΔΕ*s of 5 and 70, respectively.^46^ The exact color change can be shown to be progressively darker and redder over time (see the computational Supporting Information for details). Looking more closely at the segmentation of the frame (dashed lines in Scheme 6, top), three rows have three polka-dots and two rows have two polka-dots at the beginning of the video and likewise for columns. The result: Δ*Ε* analysis showed rows 0, 2, and 4 to be distinct from rows 1 and 3. The result was the same for the numbered columns; columns 0, 2, and 4 produced the same absolute values, as did columns 1 and 3. As the polka-dots blurred, the contrast metric (amount of the local variation in the image) decreased till it reached 0, at which time the dots had completely blurred into the red background. We might imagine the time for contrast to plateau as representing the dissolution time of the white powder into the red liquid, for example. Energy (or the ASM) reached a minimum when the video blurring showed its most pronounced gradient between the original white dots and the red background. The ASM hits its maximum possible value when the video reached complete blurring, that is, a block red image. The starting value for ASM, measuring overall block color, was high (though not at the maximum of 1) because the start of the video had more areas of similar block color and no gradient between white spots and red background. Homogeneity somewhat tracked ASM, being designed to measure whole image similarity. The contact metric tracked a swelling in the traceable perimeter of the blurring dots to a maximum before decaying to 0 once all pixels eventually fell below the defined grayscale threshold used to define the black and white recoloration of each pixel in each frame. For variance, all values decayed to 0, doing so with varying sensitivity, according to the color channel tracked. This last measurement served to show that such variance analyses, while commonly limited to grayscale, can be applied over all available color channels.

**Scheme 7:**
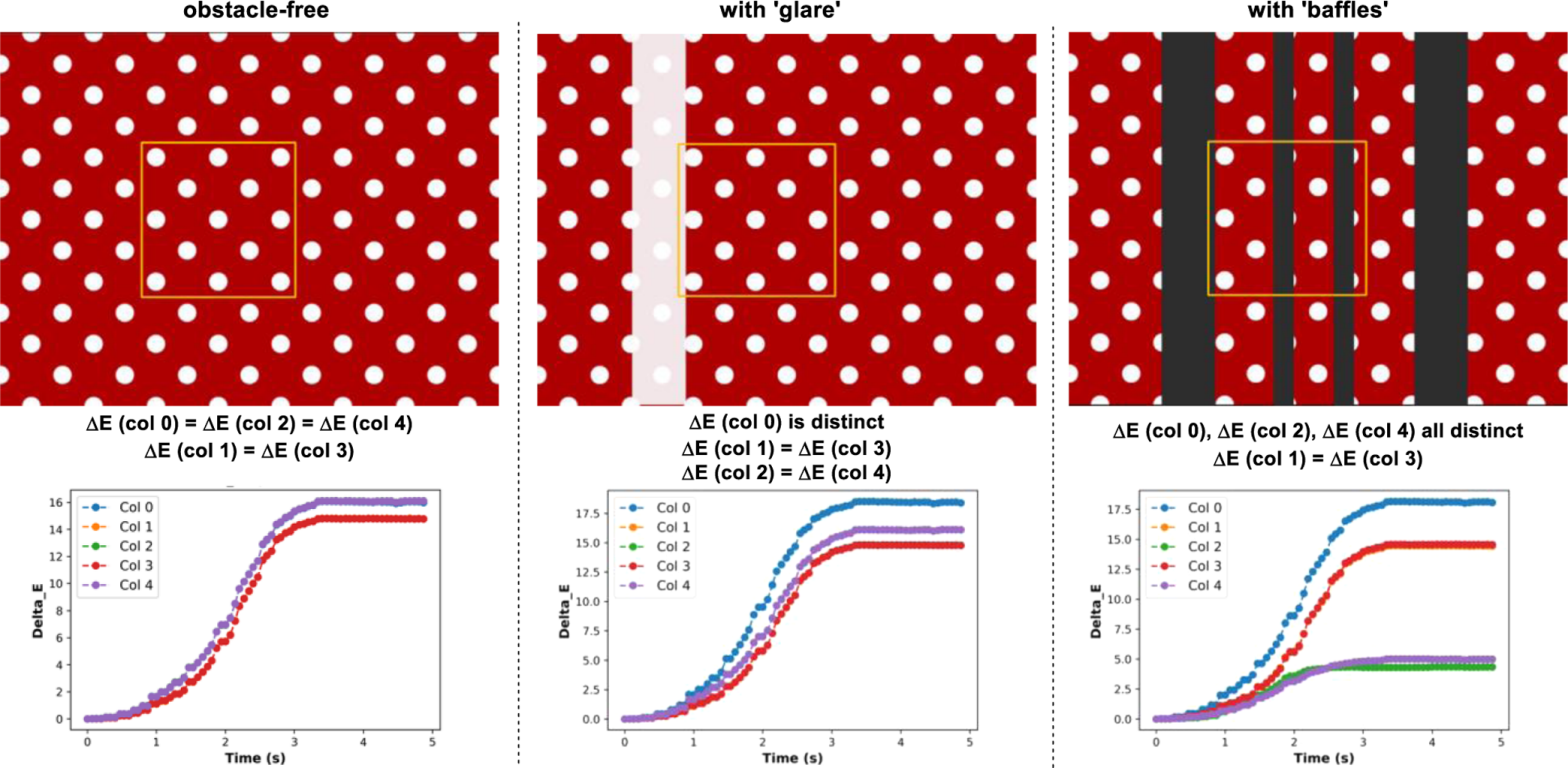
Top: First Frame for Simulated Polka-Dot Pattern Blurring with No Obstacle, with 80% Opaque White Rectangle to Represent Glass Glare, and with Four Opaque Gray Rectangles to Represent a Baffle Cage Bottom: exemplar Δ*Ε* by column data for the 5 × 5 cell portioned analysis of the yellow region of interest in each case.

Modified versions of the simplified polka-dot “mixing” simulation were able to demonstrate that neither glare (points of uneven or saturated lighting reflections on glass reactors) nor metallic baffles present in the reactor adversely affected the ability of each of the abovementioned metrics to track overall mixing time. This point held even though the said obstructions impacted some measured local changes over time (exemplified by Δ*Ε* analysis in Scheme 7, and with all other metrics in the Supporting Information).

## 3 Results and Discussion

Across all results shared below, the metrics—both averaged and spatially resolved—were chosen in order to best illustrate the quantifiable comparisons made possible by such kinetic imaging approaches. The examples in the schemes below are thus not the only comparative plots available. All raw data are available in the spreadsheet form in the Supporting Information, enabling full exploration of all average color and spatially resolved mixing metrics for each experiment described herein. To investigate the ability to quantify mixing on both developmental and intermediate scales of synthesis, we considered vessels ranging from 50 mL round-bottom and Schlenk tubes, to 3 L beakers, and finally 5 L single-jacketed stirred tank reactors (STRs).

### 3.1 Qualitative Visualization of Mixing Phenomena in Plant Mimic Vessels

We began laboratory experimentation by looking at non-reacting mixtures of solids stirred in liquids. Using super-saturated aqueous solutions of sodium hydrogen carbonate, we monitored the solid settling times after stirring was ceased. This series of experiments included comparisons between overhead stirrer speeds, impeller shapes, and presence or absence of baffles.

**Scheme 8:**
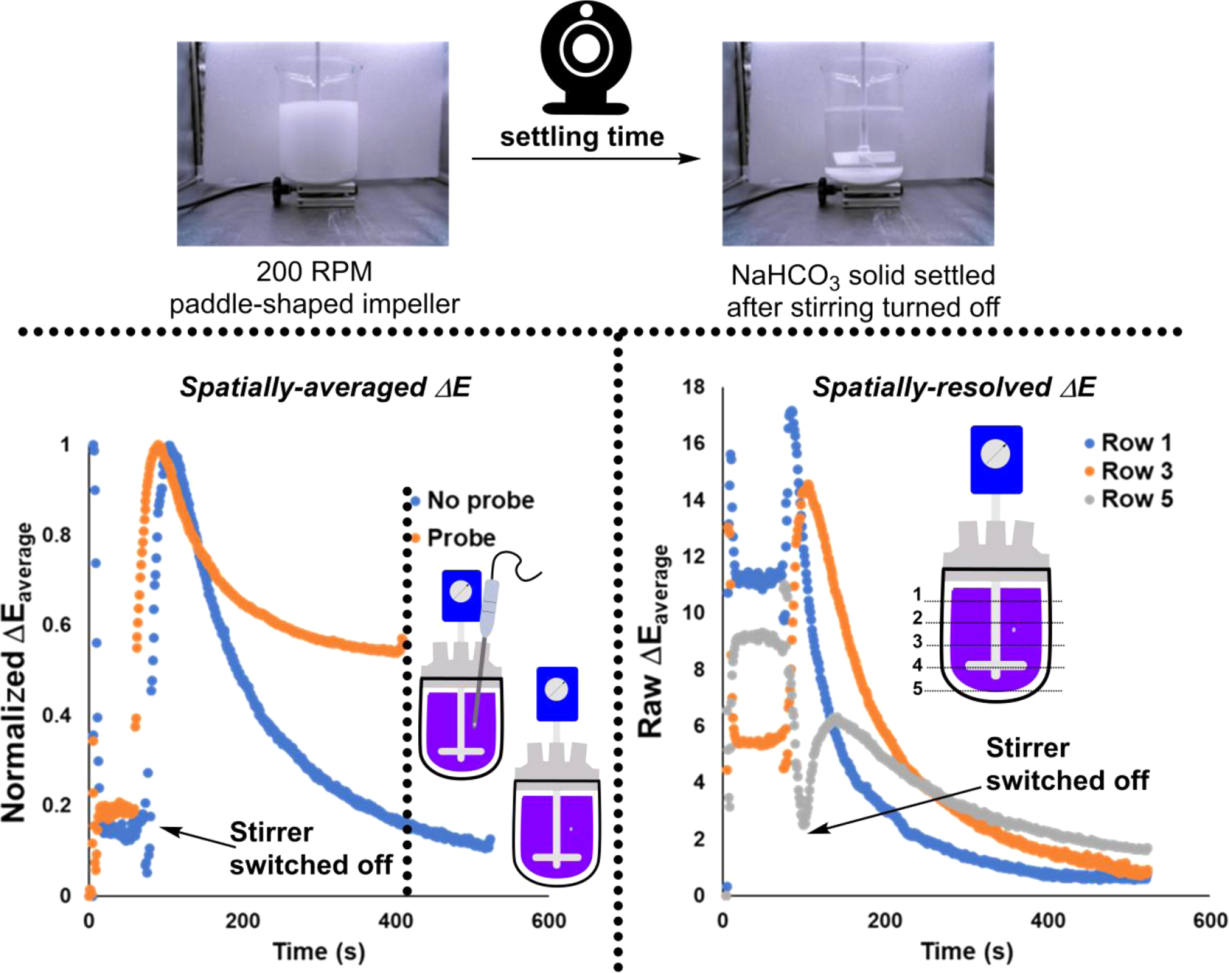
Left: Exemplar Comparison of Solid NaHCO_3_ (sat.) Settling Time in a 3 L Beaker Using the Grand (or Average) Δ*Ε* Metric Right: The same example for the probe-free reaction where the Δ*Ε* metric has been resolved by row. The default spatial resolution is a 5 × 5 a.u. matrix, where each of the resulting 25 cells is made equal in size within the selected region of interest. The Δ*Ε* metric for each cell is available, along with row averages (shown) and column averages (see the Supporting Information).

As exemplified by the Δ*Ε* metric, these proof-of-concept studies were able to show that: (i) settling time was longer when using an anchor-shaped impeller versus a paddle, (ii) the presence of a probe in the reactor reduced settling time across all stirring rates and impeller shapes, (iii) the presence of beaver tail baffles (in the subset of conditions explored) reduced settling time (Table 1). Exemplar analyses, both spatially averaged and spatially resolved, are shown in Scheme 8. In this case, average Δ*Ε* changes across the whole reactor suffice to capture the impact of including a probe-shaped object in the reactor. Presence of a probe reduces settling time (Scheme 8, left). In complement, the segmentation of Δ*Ε* into measures by row reveals the differences in rate of solid settling across the vertical height of the reactor. At the point of turning stirring off, rate of change of Δ*Ε* is highest at the top of the reactor, becoming progressively slower when observing the middle and bottom of the reactor (Scheme 8, right).

**Table 1:** Comparative Analysis of Solid Settling Time in an Overhead-Stirred 3 L Beaker, Approximated from Imaging-Derived Δ*Ε* Mixing Metrics^a^

entry	stirring rate (RPM)	impeller shape	baffles?	estimated settling time with no probe (s)	estimated settling time with probe (s)
1	60	paddle	no	75	50
2	100	paddle	no	300	80
3	210	paddle	no	400	200
4	60	anchor	no	200	55
5	100	anchor	no	350	175
6	210	anchor	no	400	225
7	100	paddle	yes	50	N.D.
8	210	paddle	yes	80	N.D.
9	60	anchor	yes	N.D.	N.D.
10	100	anchor	yes	N.D.	N.D.
11	210	anchor	yes	100	N.D.

a N.D. = not determined.

For all solid settling experiments, the full suite of above-exemplified mixing metrics was calculated. In selected illustrative cases, we also exemplified the use of higher contrasting backgrounds (e.g., red and blue card in place of white) to enable more sensitive measurements of contrast change as the white solid moved from being stirred in the suspension to being settled on the reactor base (see the Supporting Information for full details).

### 3.2 pH Titrations as a Model System for Kinetic Imaging of Mixing Phenomena

#### 3.2.1 Phenolphthalein Titrations—Assessing the Impact of Baffles

At the core of this academia–industry collaboration was a specific interest in using colorimetric means of analyzing mixing kinetics in overhead stirred reactors. Moving from earlier solid–liquid experiments to the liquid–liquid regime, we used the Kineticolor platform to analyze legacy footage from educational mixing analysis video recording, available within Fujifilm.

**Scheme 9:**
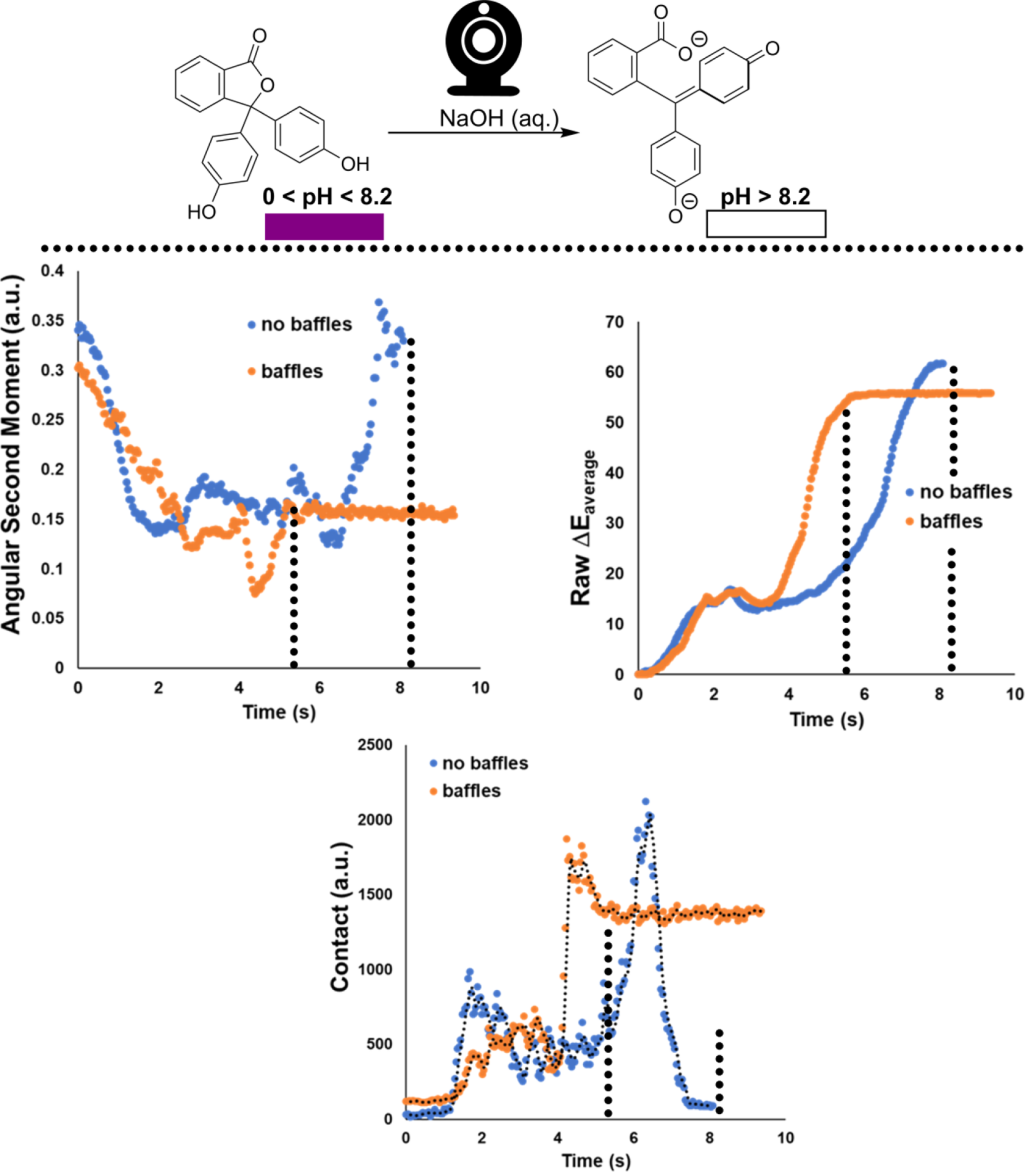
Exemplar Phenolphthalein Colorimetric pH Titration Visualizing the Impact of Beaver-Tailed Baffles on the Mixing Efficiency in an Overhead Stirred 5 L Beaker Stirred at 400 RPM Top left: ASM (or energy). Top right: global average Δ*Ε*. Bottom: contact. For all mixing metrics, the value plateaus more rapidly in the baffled versus non-baffled reactor. The dotted lines against both curve shapes and time points are provided solely as a guide to the eye.

Titration of acidified phenolphthalein with aqueous sodium hydroxide, accompanied by a purple to clear color change, was employed to assess the impact of baffles in a reactor. Under otherwise identical chemical and physical conditions, the more rapid color change in the baffle-containing reactor was captured and quantified using the Kineticolor platform. Quantifying the improved mixing efficiency in the baffled versus non-baffled reactor was captured across several mixing metrics from the full suite calculated. For co-plotted comparison of baffled and unbaffled reactors, Scheme 9 shows that:

(i) Angular second moment (ASM), capturing high levels of pixel order in high values (and vice versa), showed the baffled reactor plateaued at a lower value after mixing than that at the start of the reaction.
(ii) Contact, capturing higher values for longer perimeters outlining pixels at above and below grayscale threshold positions (and vice versa), settled to a new high level versus the low starting level when baffles are present.
(iii) Average (or grand) Δ*Ε*, capturing higher levels of color-independent contrast versus time zero at higher values (and vice versa), showed the baffled reactor settling at a slightly lower Δ*Ε* value in less time compared to the non-baffled reactor.

In all three cases, the most important point of similarity is being able to extract macro-scale mixing times (plateaus) for each reactor type. The absolute differences in each metric’s *y*-axis values are, in part, contributed to by the inclusion of the baffles in the selected region of interest for analysis. The baffles become more visibly distinct from the reaction medium as the reaction progresses from purple to colorless.

**Scheme 10:**
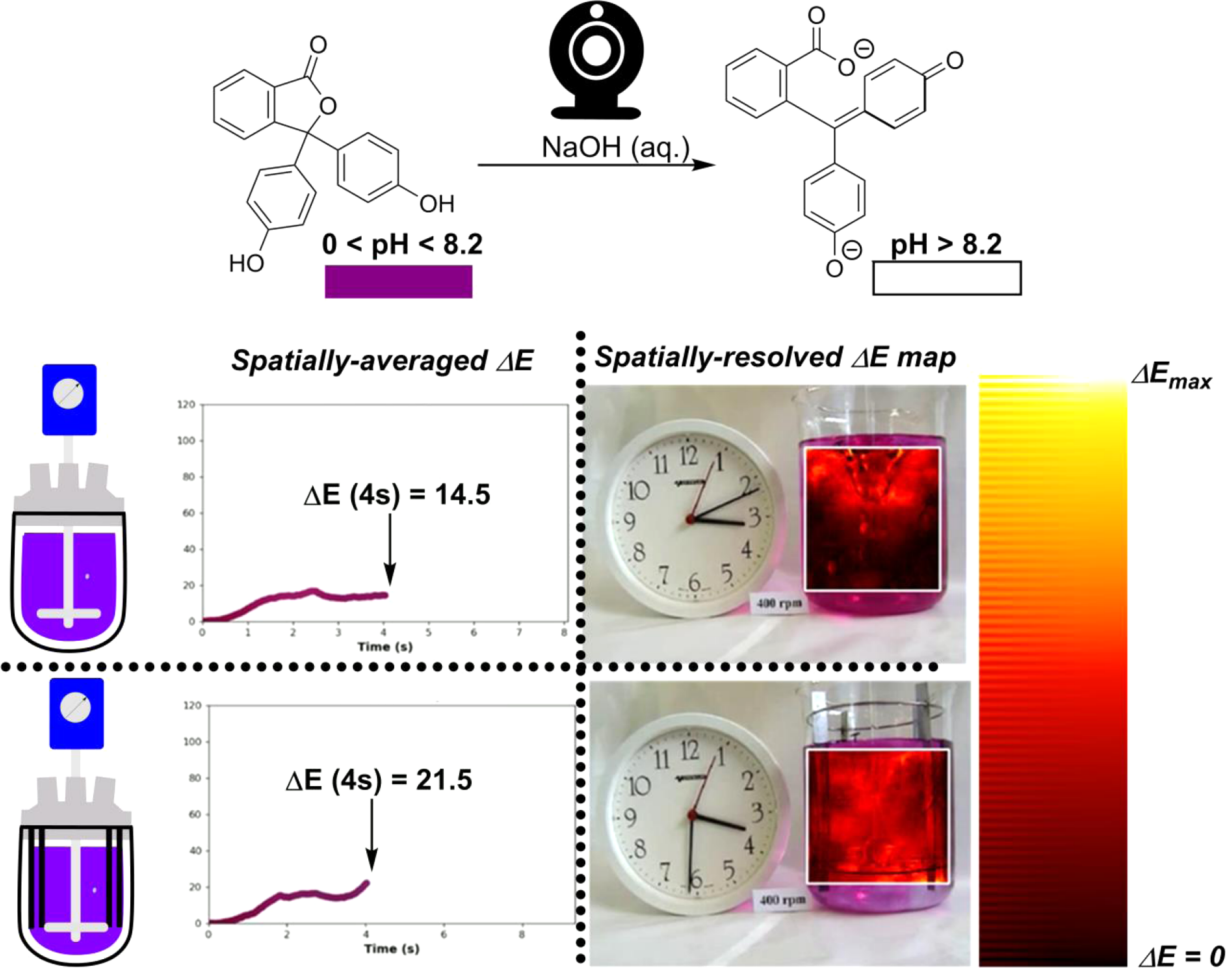
Exemplar Phenolphthalein Colorimetric pH Titration Visualizing the Impact of Beaver-Tailed Baffles on the Mixing Efficiency in an Overhead Stirred 3 L Beaker Stirred at 400 RPM Stills at 4 s from videographic reports of Δ*Ε* versus time show lower overall progress of Δ*Ε*_average_ when no baffles are present. Fully spatially resolved maps of Δ*Ε* per pixel in the region of interest show a higher proportion of “red hot” and “white hot” pixilation in the baffled reactor versus non-baffled reactor.

While both ASM and contact metrics shown above captured part of the spatial component of mixing, we show in Scheme 10 that the arguably more intuitive Δ*Ε* metric can be extended to provide full spatial resolution at the level of individual pixels. Indeed, this enabled the creation of Δ*Ε* “heatmaps,” intuitively displaying the distribution of contrast change across the reactor, and not merely overall average contrast change within the selected region of interest.

#### 3.2.2 Bromothymol Blue Titrations—the Impact of Baffles, Stirrers, Probes, Impellers, and Vessel Geometry

**Scheme 11:**
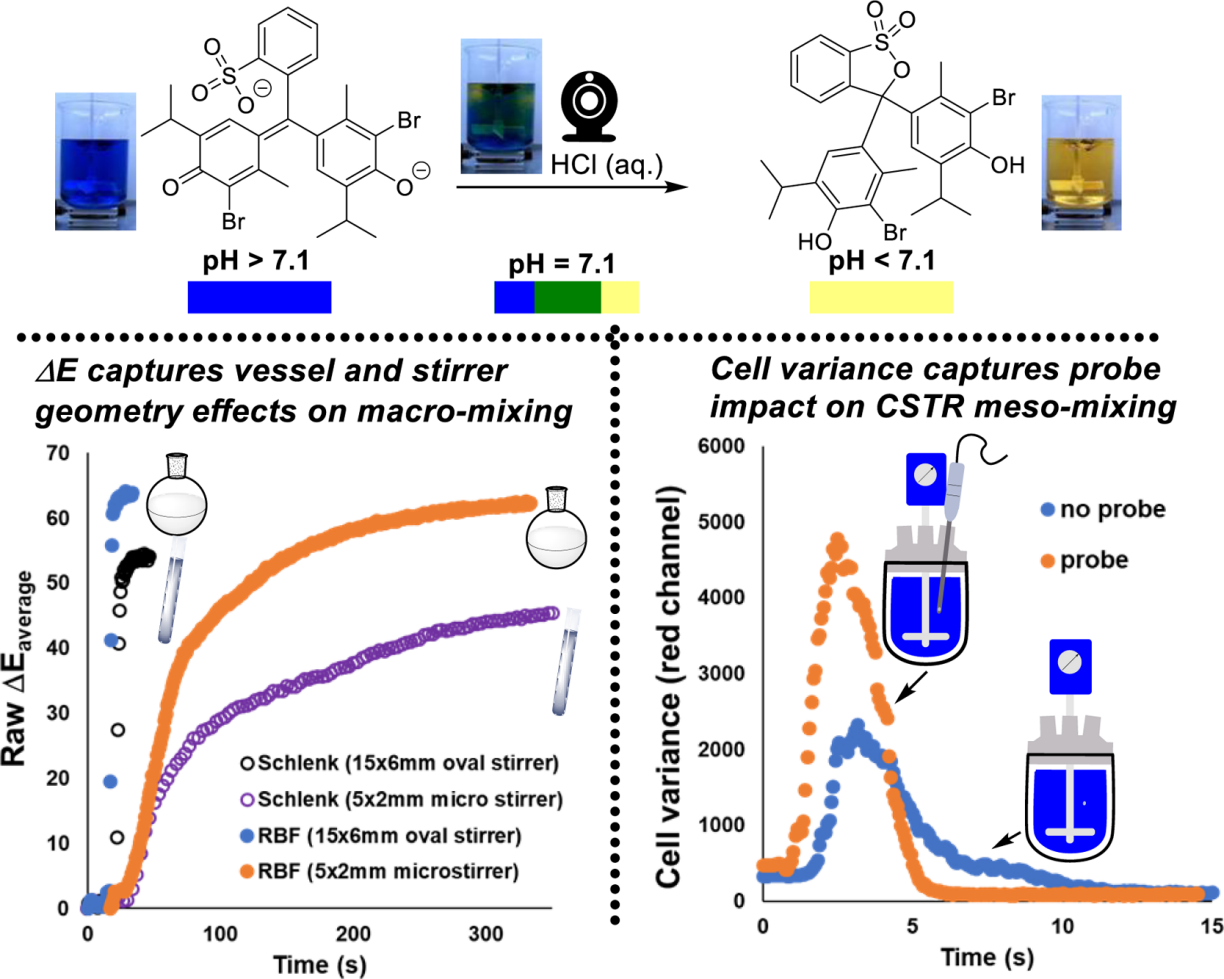
Left: Exemplar Bromothymol Blue Colorimetric pH Titration Using Δ*Ε*_average_ to Visualize the Impact of Vessel Shape (50 mL Round-Bottom Flask versus 50 mL Schlenk Tube) and Magnetic Stirrer Size on the Mixing Efficiency Use of larger oval stirrers gives lower mixing times than micro-stirrers. Round-bottom flasks enable faster mixing times than Schlenk tubes. Right: using red channel variance as a measure of the range of color over a segmented squared grid in the 5 L STR reactor area. The presence of a probe in the 5 L STR approximately halved the overall mixing time.

Moving beyond the legacy titration footage from Fujifilm archives, we generated new pH titration data for mixing analysis using bromothymol blue. Inspired by the work of Fitschen et al. in the analysis of macro- and micro-mixing phenomena using high-contrast color changes,^2^ we recorded a series of bromothymol blue titrations (tracking blue through green to yellow color changes) across a series of reaction vessels. Scheme 11 (left) emphasizes the fact that such mixing concerns are not particular to overhead stirred (larger scale) tank reactors. Development-scale round-bottom and Schlenk flasks are also affected. Averaged Δ*Ε* changes over time sufficed to demonstrate the point.

Extending the bromothymol blue experiments to the 5 L STR scale enabled further demonstration of the expected impact of “probes” present in the reactor. Probes act like pseudo-baffles.

**Scheme 12:**
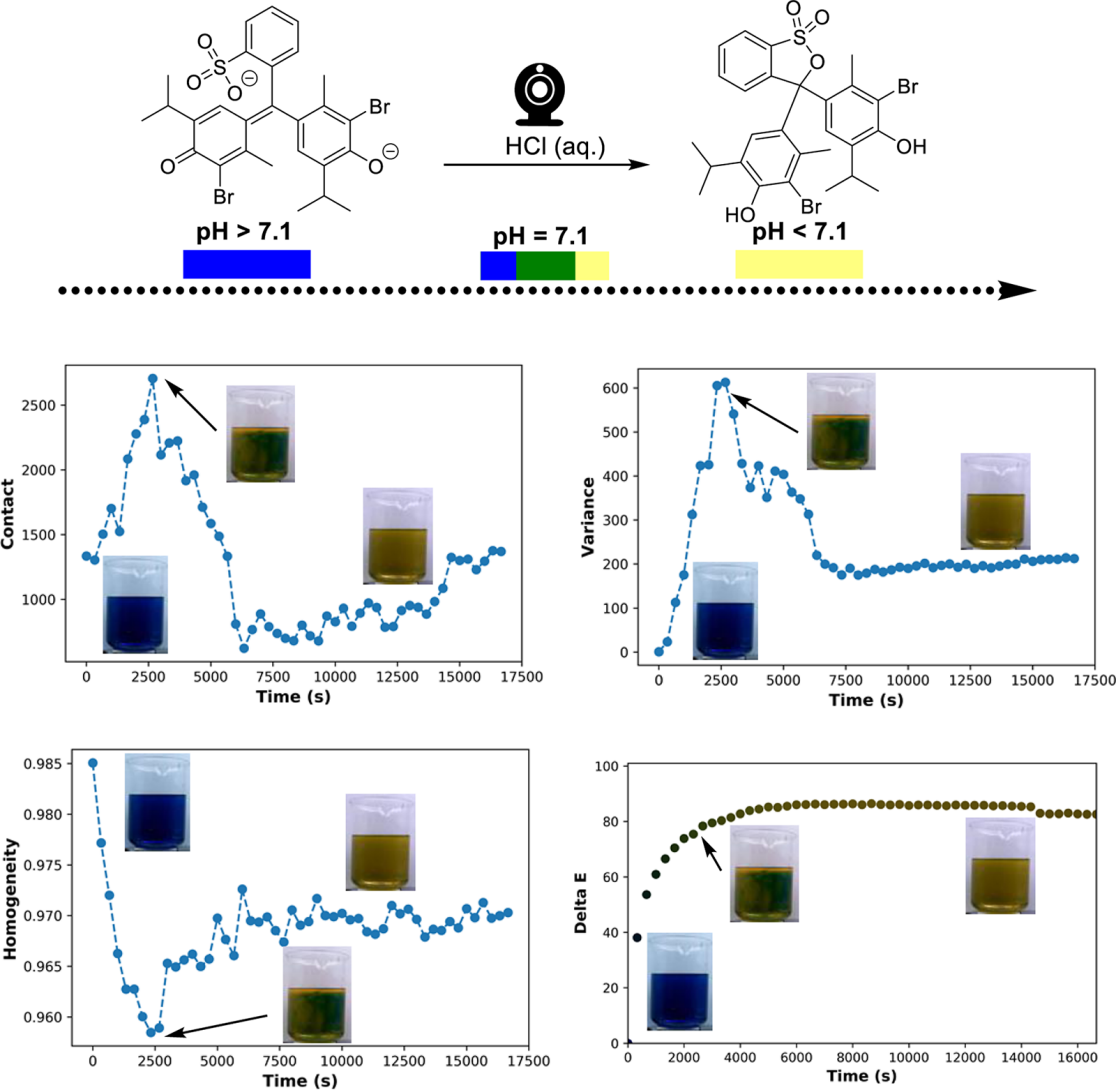
Non-Stirred Bromothymol Blue Colorimetric pH Titration in 3 L Overhead-Stirred Beaker, Using Contact, Homogeneity, and Variance (RGB Red Channel) Metrics to Capture the Point of Maximum Heterogeneity (Arrow) This point in the reaction visibly showed the most spread of blue, green, and yellow coloration between the blue and yellow extrema. Averaged Δ*Ε* (bottom right graph) does not capture this spatially specific phenomenon. Examples of the bromothymol blue titration conducted in a STR with different impeller types are available in the Supporting Information.

To demonstrate the deeper value of spatially resolved mixing metrics over averaged color metrics, we carried out a bromothymol blue titration in a 3 L beaker with no stirring. The 4 h experiment also served to exemplify the ability to manage data set size by using Kineticolor’s frame skip setting to analyze, in this case, just 50 of over 500,000 (0.01%) of all video frames collected (Scheme 12).

#### 3.2.3 Colorimetric Kinetic Analysis of Competing, Mixing-Sensitive Reactions

**Scheme 13:**
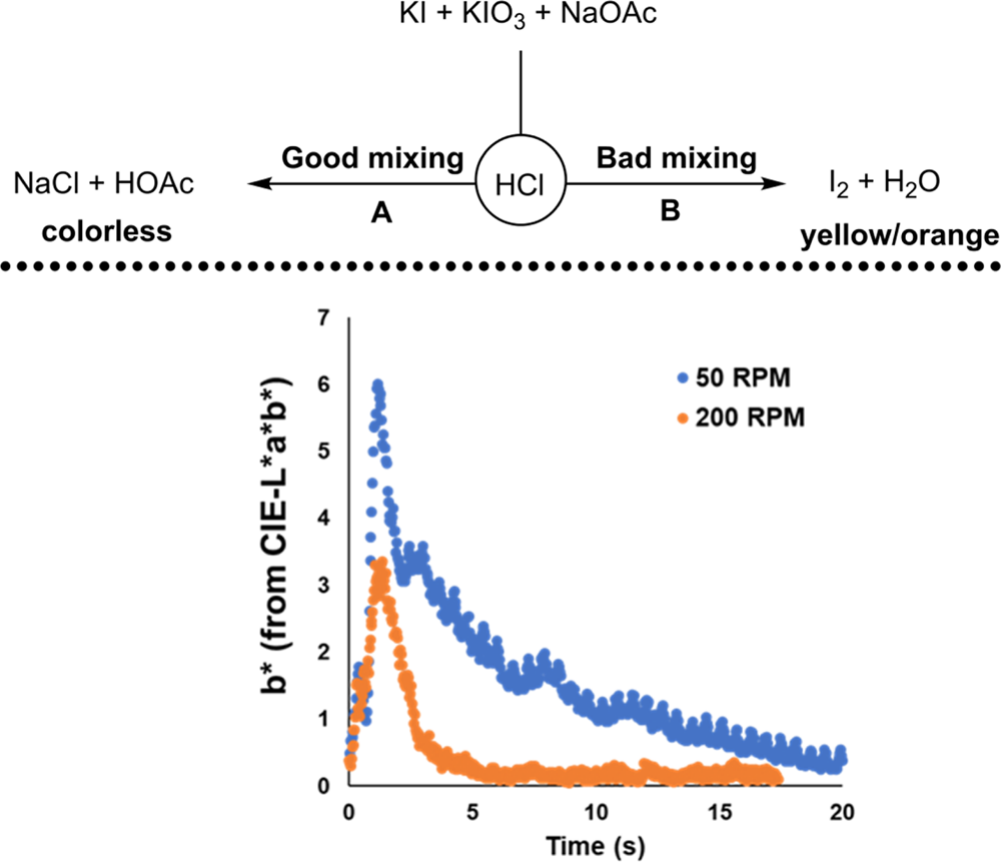
Using the b* Channel from CIE-L*a*b* Color Space to Track Average Change in Yellow Coloration in the 5 L STR versus Time Higher values on the *y*-axis denote more yellowing of the reaction mixture. It is shown that a larger component of side reaction B competes with reaction A at lower stirring rates.

Complementary to the relative simplicity of linear reactions exemplified by the pH titrations, we investigated the computer vision-enabled analysis of competing parallel reactions using the Villermaux method.^58,59^ When an aqueous solution of potassium iodide, potassium iodate, and sodium acetate is mixed with aqueous hydrochloric acid, two reactions take place in varying proportions, depending on the mixing efficiency (Scheme 13, top). With efficient mixing, parallel reaction kinetics are dominated by the relative rate constants, and thus, the formation of colorless sodium chloride and acetic acid dominates as major products (reaction A). However, when mixing is poor, the local concentration of acid is such that, once all local sodium acetate is quenched, some acid remains to participate in the comparatively slower iodine formation (reaction B).

Scheme 13 (bottom) shows how such specific colorimetric signals of mixing quality can be analyzed using a single component (or dimension) of a color space, without the need for more complex spatial calculations. Because poor mixing was marked by the characteristic yellow/orange of iodine, the b* component of the CIE-L*a*b* color space (where positive b* values signify more yellow, and negative b* values signify more blue coloration) was sensitive enough to provide a comparative analysis of two different stirring rates. At 50 RPM, the b* channel hit a higher peak and decayed more slowly than for the same process run with a 200 RPM stirring rate. These data semi-quantified (to an intrusively useful standard) the greater persistence of iodine in the more slowly stirred reactor.

### 3.3 Application of Mixing Data in Highly Mixing Sensitive Chemistries

**Scheme 14:**
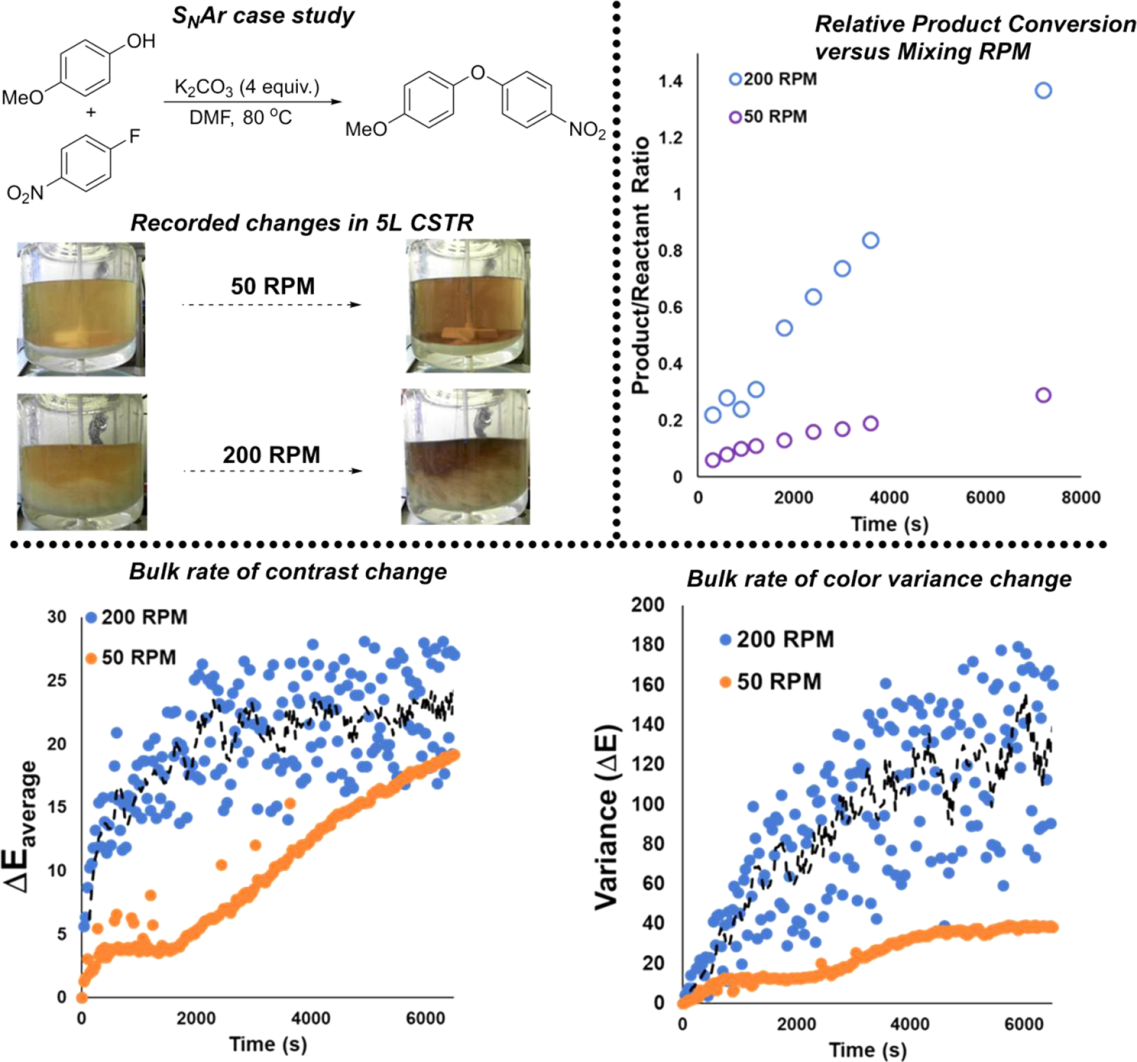
Top Left: S_N_Ar Case Study (Approx. 0.8 mol or 112 g Scale Relative to 4-Nitro-fluorobenzene) Top right: relative product/phenol reactant ratio, showing the positive influence of more effective stirring and suspension of K_2_CO_3_. Bottom left: global or average Δ*Ε* for each stirring regime, with higher RPM leading to the more rapid rate of visible color contrast changes. Bottom right: Δ*Ε* measured across a 5 × 5 square grid of pixel cells. As the liquid darkens over time, the variance metric captures the increasing contrast between the liquid and suspended white solid. Dotted lines depict 5-point moving averages added as a guide to the eye.

We applied this collective computer vision-enabled mixing analysis to a more practically relevant organic reaction scale-up. Namely, we looked at the impact of extreme changes in the impeller stirring rate for a nucleophilic aromatic substitution (S_N_Ar) reaction. The chosen reaction relies on the effective suspension of the potassium carbonate base in the bulk solution. As shown in Scheme 14, offline ^1^ NMR measurements of relative product conversion drastically improved with the higher stirring rate. The higher stirring rate led to visibly obvious suspension of the solid base throughout the bulk DMF liquid. At the lower stirring rate, the base was concentrated on the bottom of the reactor and not visibly suspended to any extent.

The bottom of Scheme 14 captures efforts to track mixing-related effects using Kineticolor. For all comparisons (here and in the Supporting Information), the data for the efficient mixing regime were noisier on account of the periodic swirling of white solid across the darkening DMF solution. Nonetheless, average Δ*Ε* measurements evidenced the appreciably faster rate of color (strictly contrast) change in the 200 RPM case over the 50 RPM case (Scheme 14, bottom left). Turning to the spatially resolved mixing metrics, the variance of Δ*Ε* across a 5 × 5 square grid of pixel cells mapped over the whole reactor showed that the contrast change was increasingly varied over the reactor’s visible cross-sectional area with time. In the more efficiently stirred reactor, the variance in color across the reactor increased more quickly than that for the more poorly stirred reactor. This observation is consistent with the bulk Δ*Ε* change and with the more effective suspension of a white solid throughout a darkening liquid.

**Scheme 15:**
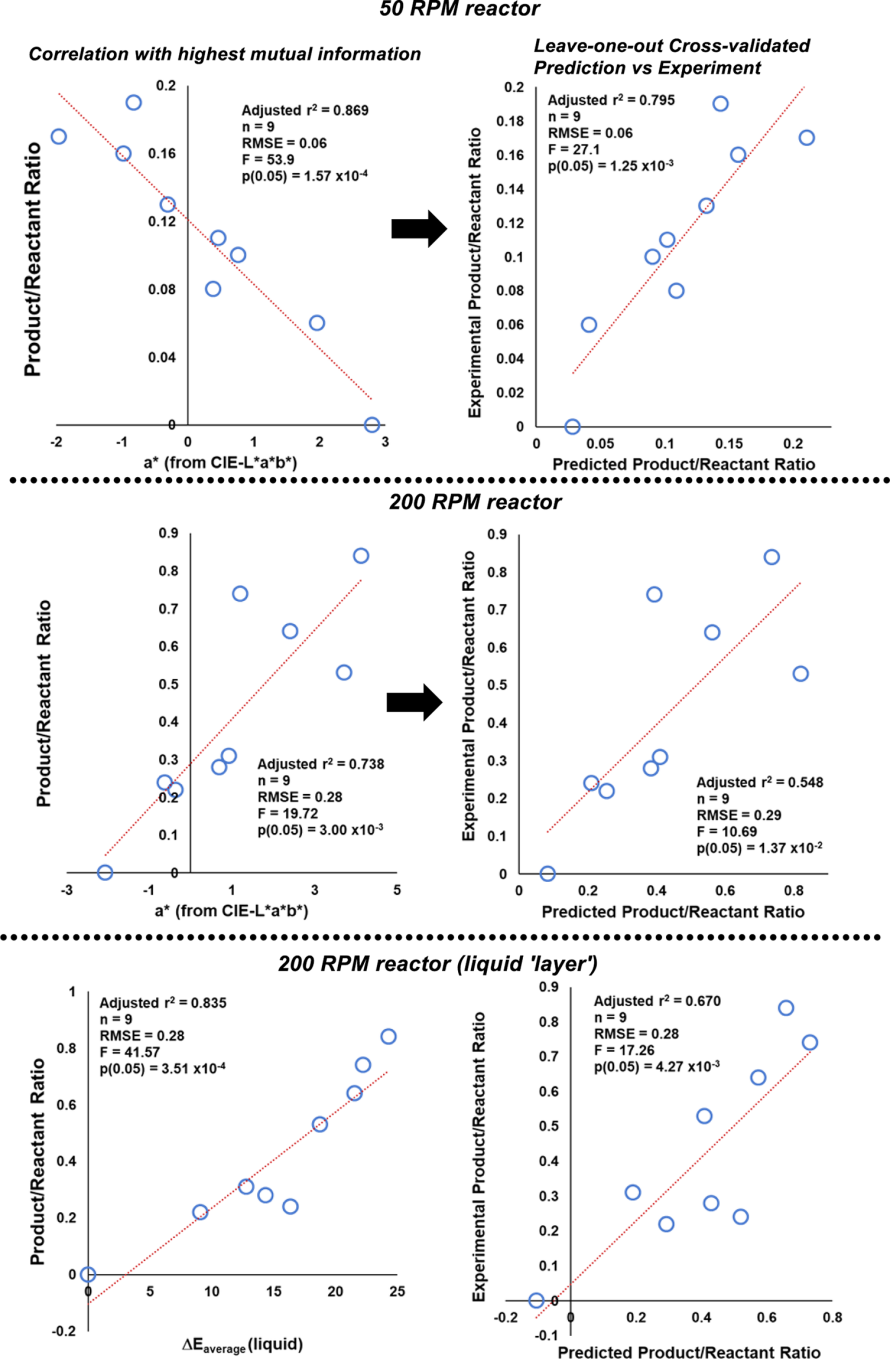
Top: Correlation (Left) and Leave-One-Out Cross-Validated Prediction (Right) for S_N_Ar Reaction Stirred at 50 RPM Middle: related data for the 200 RPM case. Bottom: improved predictive power using average Δ*Ε* when analysis is restricted to the primarily liquid-containing top of the reaction mass, avoiding the solid.

We concluded the study by investigating the predictive power to determine the relative product/reactant ratios using the colorimetric data extracted with Kineticolor. As per our earlier study investigating palladium-catalyzed borylation processes,^46^ we applied Shannon’s mutual information (M.I.)^60^ method as a non-parametric (not linearly dependent) guide to searching for single-component regression models of color versus concentration data. Applying the analysis to the first, regularly sampled hour of the S_N_Ar reactions, M.I. analysis revealed the a* component (covering positive red through to negative green values, from the CIE-L*a*b* color space) to contain the highest level shared information with offline NMR concentration measurements (see the Supporting Information for details). For both the 50 and 200 RPM reactors, Scheme 15 shows the correlation of a* versus ^1^H NMR measurement of reaction progress. The leave-one-out cross-validated prediction of ^1^H NMR data from color data (a*) performed better for the more poorly stirred reactor in which swirling solid was less disruptive to video analysis. Having said this, in the more rapidly stirred reactor, when video analysis was restricted to the top of the reaction bulk (mostly free from swirling solid), a more powerful prediction of reaction progress was revealed through the Δ*Ε* (as opposed to the a*) metric (Scheme 15, bottom).

## 4 Limitations and Future Work

As exemplified in the analysis of saturated sodium hydrogen carbonate suspensions in water, the captured video data can be judiciously tuned by considering deliberate changes to the experimental hardware (e.g., background color). While not attended here, an investigation of useful lighting alterations (e.g., to investigate particle size effects) is the subject of future work. In relation, applications of this combined approach to studying the bulk kinetics of averaged and spatially resolved color changes across a broader range of synthetic transformations and crystallization problems should be considered.

It is important to note that all analysis enabled by the approach described herein captures primarily surface-visible bulk reactor information, with no explicit penetration into or analysis of the inner fluid not visible through the reactor walls. The methods here should complement, rather than replace, any of the analytical methods discussed in the Introduction section.

## 5 Conclusions

We have compared and applied a range of computer vision-enabled mixing metrics for process-relevant mixing phenomena. Through the development of the Kineticolor platform, both averaged and spatially resolved insights into the evolution of mixing in reactors across scales of operation (from Schlenk and round-bottom flasks to plant mimic vessels) have been exemplified. Visually useful signals of non-chemical and chemical processes were both (at least) semi-quantifiable using this computer vision approach. Indeed, perceptually uniform measures of contrast such as Δ*Ε* have been shown to capture color changes that are not detectable by the eye. Moreover, simple single-step homogeneous reactions (i.e., pH titrations), as well as more complex parallel and heterogeneous reactions, could be tracked using averaged and spatially resolved mixing and bulk color metrics. This imaging strategy has also been shown to hold great potential in building quantitative models mapping non-contact color data to more established offline measures of analyte concentrations. Overall, we predict that the computer vision analysis exemplified by the Kineticolor platform will be applicable to multiple other process-relevant reaction monitoring problems wherein the quantification of reaction bulk will serve as a valuable complement to more established and invasive reaction monitoring methods.

## Supporting Information Available

Experimental details of all mixing experiments and camera setups (PDF)
Computational details of computer vision and statistical analyses (PDF)
Collected spreadsheet outputs (all computer vision data) (ZIP)
