# Supplementary material for: Computer Vision for Kinetic Analysis of Lab- and Process-Scale Mixing Phenomena
Source: Org Process Res Dev. 2022 Nov 4;26(11):3073–88. doi: 10.1021/acs.oprd.2c00216 (PMC9680030; doi:10.1021/acs.oprd.2c00216)
Supplement: Supplementary file 1 — op2c00216_si_001.pdf [file op2c00216_si_001.pdf]

Computer Vision for Kinetic Analysis of Lab- and Process-scale Mixing Phenomena

Henry Barrington, ‡[a] Alan Dickinson,[b] Jake McGuire, ‡[a] Chunhui Yan, ‡[a] and Marc Reid*[a]

a. Department of Pure & Applied Chemistry, University of Strathclyde, Royal College Building
204 George St, Glasgow G1 1XW.
b. Colorants Technology Centre, FUJIFILM Imaging Colorants, Earls Road, Grangemouth, FK3 8XG

‡These authors contributed equally.

Experimental Supporting Information

Contents

1. General Considerations 3
2. Solid-Liquid Mixing 3
2.1 Sodium Hydrogen carbonate 3
2.2 Nucleophilic Aromatic Substitution 4
3.0 Liquid-Liquid Mixing 5
3.1 Bromothymol Blue in Beaker 5
3.2 Bromothymol Blue in STR 5
3.3 Bromothymol Blue in Round Bottom and Schlenk Flasks 6
3.4 Villermaux-Dushman Reaction 7
4.0 Potassium Carbonate Catalysed Etherification Product Ratio 8
5.0 Nucleophilic Aromatic Substitution ^{1}H-NMR Spectra 10
5.1 4-Methoxyphenol 10
5.2 1-Fluoro-4-nitrobenzene 10
5.3 50 RPM reactor 11
5.4 188 RPM reactor 16
6.0 Kineticolor and Video Analysis 21
7.0 References 23

1. General Considerations

The videos were recorded at a frame rate of 30 Hz and a spatial resolution of 1920 × 1080. ^{1}H spectra were recorded on a Bruker AVIII-400 spectrometer at 400 MHz. Chemical shifts are reported in ppm. Coupling constants are reported in Hz. The following chemicals were employed:

- Bromothymol blue ACS reagent dye content 95% (*SIGMA-ALDRICH*)
- Sodium hydrogen carbonate, ≥ 99% (*Fisher Scientific*)
- 4-Methoxyphenol (*Fluorochem*)
- 1-Fluoro-4-nitrobenzene (*Fluorochem*)
- Potassium carbonate ACS reagent ≥ 99% (*SIGMA-ALDRICH*)
- N,N-dimethylformamide for peptide synthesis (*Rathburn*).

2. Solid-Liquid Mixing

2.1 Sodium Hydrogen carbonate

Sodium hydrogen carbonate (392 g, 4.666 mol) was added to a 3.5 L beaker with 2 L water at 20 °C to give a saturated sodium hydrogen carbonate aqueous solution with approx. 200 g suspended particulate. A Heidolph RZR 2040 overhead stirrer fitted with an anchor or paddle stirrer was employed at a height of 1.5 cm above the bottom of the beaker. Apparatus was stationed in a GODOX LST60 LED mini photography tent 60 × 60 × 60 cm lightbox. OBS studio and a Microsoft LifeCam Studio 1425 were employed as recording software and device respectively. Recording was started and stirring commenced. The suspension was allowed to form a homogenous mixture, stirred for at least 30 seconds, then stirring was ceased and the suspension allowed to settle. A simulated probe was added by affixing a 25 × 8 cm cylindrical Teflon magnetic stir bar retriever at 0.5 cm height from the bottom of the beaker and 3 cm from the side. Experiments were repeated at 60, 100 and 210 rpm for both anchor and paddle stirrers and vessels containing a simulated probe and no probe.

2.2 Nucleophilic Aromatic Substitution

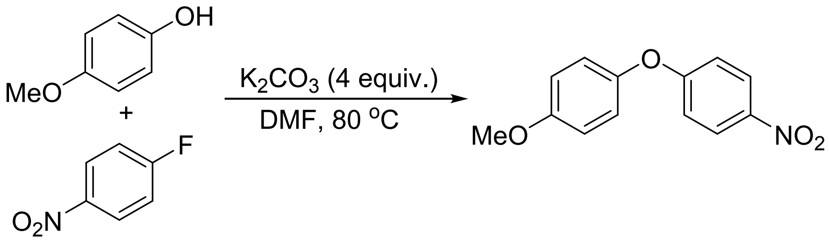

This reaction was scaled up from a published procedure.[1] A 5 L jacketed plant-mimic Radleys tank reactor was filled with 3 L N,N-dimethylformamide. An IKA EUROSTAR digital Electronic Overhead Stirrer (EURO-ST D Rührwerk Homogenizer) fitted with an anchor or paddle stirrer was employed at a height of 5.5 cm above the bottom of the reactor tank. The mixture was heated to 60 °C with a circulator while stirring with a 4-bladed propeller stirrer. To this was added 1-fluoro-4-nitrobenzene (112.0 g, 0.794 mol) and 4-methoxyphenol (98.5 g, 0.794 mol). The reagents were dissolved giving a canary solution. OBS studio and a Microsoft LifeCam Studio 1425 were employed as recording software and device respectively. After checking the internal reaction mixture temperature via thermometer stirring was commenced and recording started. Solid potassium carbonate (164.5 g, 1.190 mol, ~1.5 equiv.) was added to form a honey suspension with white solids. The reaction mixture was sampled at 0, 5, 10, 15, 20, 30, 40, 50, 60, and 120 minutes. Sampling was performed using a cannula and quenching into an excess of 1 M hydrochloric acid aqueous solution. After half an hour the quench also induces precipitation of yellow solids which become more voluminous at increased sampling times. Samples were subjected to ^1H NMR spectroscopy using the ratio between methoxy protons of 4-methoxyphenol and 1-methoxy-4-(4-nitrophenoxy)benzene as a measure of reaction progression. Spectra were calibrated to the $Me_2NCO\mathit{H}$ resonance as 8.03 ppm. The methoxy protons of 4-methoxyphenol and 1-methoxy-4-(4-nitrophenoxy)benzene can be found between 3.71–3.73 ppm and 3.86–3.87 ppm respectively.

Experiment was repeated at 50 rpm and an anchor stirrer. On the repeat an excess (~ 500 mL) of N,N-dimethylformamide was used.

3.0 Liquid-Liquid Mixing

3.1 Bromothymol Blue in Beaker

This preparation was adapted from a published procedure.[2] Bromothymol blue (12 mg) was dissolved in 90 µL 2 M sodium hydroxide aqueous solution, added to a 3.5 L beaker and made up to 2 L with water at 20 °C to give a deep blue bromothymol solution. The pH was recorded with an Orion Star A211 benchtop pH meter using a ROSS Sure-Flow combination pH electrode and adjusted to a pH of 8.5. A Heidolph RZR 2040 overhead stirrer fitted with an anchor or paddle stirrer was employed at a height of 1.5 cm above the bottom of the beaker. Apparatus was stationed in a GODOX LST60 LED mini photography tent 60 × 60 × 60 cm lightbox. OBS studio and a Microsoft LifeCam Studio 1425 were employed as recording software and device respectively. Stirring was commenced and recording started. A 180 µL aliquot of 2 M hydrochloric acid aqueous solution was added via a IKA Pette vario 100 – 1000 µL micropipette and the mixture allowed to run to completion, signaled by the color change of blue to yellow. The pH is brought back to 8.5 for concurrent runs. Experiments were repeated at 200 rpm for both anchor and paddle stirrers, in the presence of mock in-house manufactured baffles or without. Another repetition was performed without stirring for an exaggerated example of “bad” mixing.

3.2 Bromothymol Blue in STR

An aqueous alkaline bromothymol blue was prepared analogously as in the experiments conducted in the beaker scaled up to 3 L. This was added to a Radleys 5000 mL Single Jacketed Process Vessel. An IKA EUROSTAR digital Electronic Overhead Stirrer (EURO-ST D Rührwerk Homogenizer fitted with an anchor or paddle stirrer was employed at a height of 5.5 cm above the bottom of the reactor. OBS studio and a Microsoft LifeCam Studio 1425 were employed as recording software and device respectively. Stirring was commenced and recording started. A 180 µL aliquot of 2 M hydrochloric acid aqueous solution was added via a IKA Pette vario 100 – 1000 µL micropipette and the mixture allowed to run to completion, signalled by the colour change of blue to yellow. The pH is brought back to 8.5 for

concurrent runs. A simulated probe was added by fitting a 45 × 8 cm cylindrical Teflon magnetic stir bar retriever in a fixture of the tank reactor lid. Experiments were repeated at 200 rpm for both anchor and paddle stirrers, in the presence of a simulated probe and without.

3.3 Bromothymol Blue in Round Bottom and Schlenk Flasks

A stock solution of aqueous alkaline bromothymol blue was prepared analogously as in the experiments conducted in the large scale glassware. 5 mL of stock solution was added to a 25 mL round bottom flask (R.B.F.) equipped with a 15 × 6 mm oval magnetic stirrer. Apparatus was stationed in a GODOX LST60 LED mini photography tent 60 × 60 × 60 cm lightbox. OBS studio and a Microsoft LifeCam Studio 1425 were employed as recording software and device respectively. Stirring was commenced at 300 rpm and recording started. A 4.5 μL aliquot of 2 M hydrochloric acid aqueous solution was added via a IKA Pette vario 0.5 – 10 μL micropipette and the mixture allowed to run to completion, signaled by the color change of blue to yellow. Experiments were repeated using a 25 mL R.B.F., 25 mL Schlenk flask, 50 mL R.B.F., 50 mL Schlenk flask, with a 15 × 6 mm oval magnetic stirrer and a 5 × 2 mm microbar. 25 mL of alkaline aqueous bromothymol blue solution and a 22.5 μL aliquot of 2 M hydrochloric acid aqueous solution added via a IKA Pette vario 10 – 100 μL micropipette was employed for the 50 mL glassware.

3.4 Villermaux-Dushman Reaction

Quantities used were adapted from published procedures.[3] A 3 L aqueous solution of potassium iodide (7.968 g), potassium iodate (1.926 g), and sodium hydroxide (5.4 g) was prepared and transferred to a Radleys 5000 mL Single Jacketed Process Vessel. An IKA EUROSTAR digital Electronic Overhead Stirrer (EURO-ST D Rührwerk Homogenizer fitted with an anchor or paddle stirrer was employed at a height of 5.5 cm above the bottom of the reactor. OBS studio and a Microsoft LifeCam Studio 1425 were employed as recording software and device respectively. Stirring was commenced and recording started. Hydrochloric acid aqueous solution (0.1 M, 30 mL) was added via funnel and recording continued for at least 30 seconds. Recording was recommenced and a second addition of hydrochloric acid aqueous solution (0.1 M, 30 mL) was performed. Again, recording was continued for at least 30 seconds after addition. Experiments were repeated at 50 or 200 rpm.

4.0 Potassium Carbonate Catalysed Etherification Product Ratio

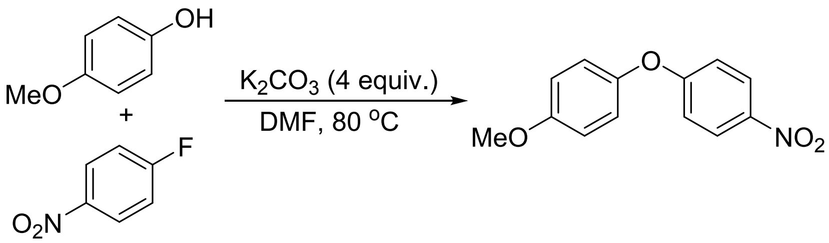

Table S1 Summarised ^{1}H NMR sample results for nucleophilic aromatic substitution reactions described in manuscript Scheme 14.

time (min)	time (s)	Ether/Phenol (188 RPM)	Ether/Phenol (50 RPM)
0	0	-	-
5	300	0.22	0.06
10	600	0.28	0.08
15	900	0.24	0.1
20	1200	0.31	0.11
30	1800	0.53	0.13
40	2400	0.64	0.16
50	3000	0.74	0.17
60	3600	0.84	0.19
120	7200	1.37	0.29

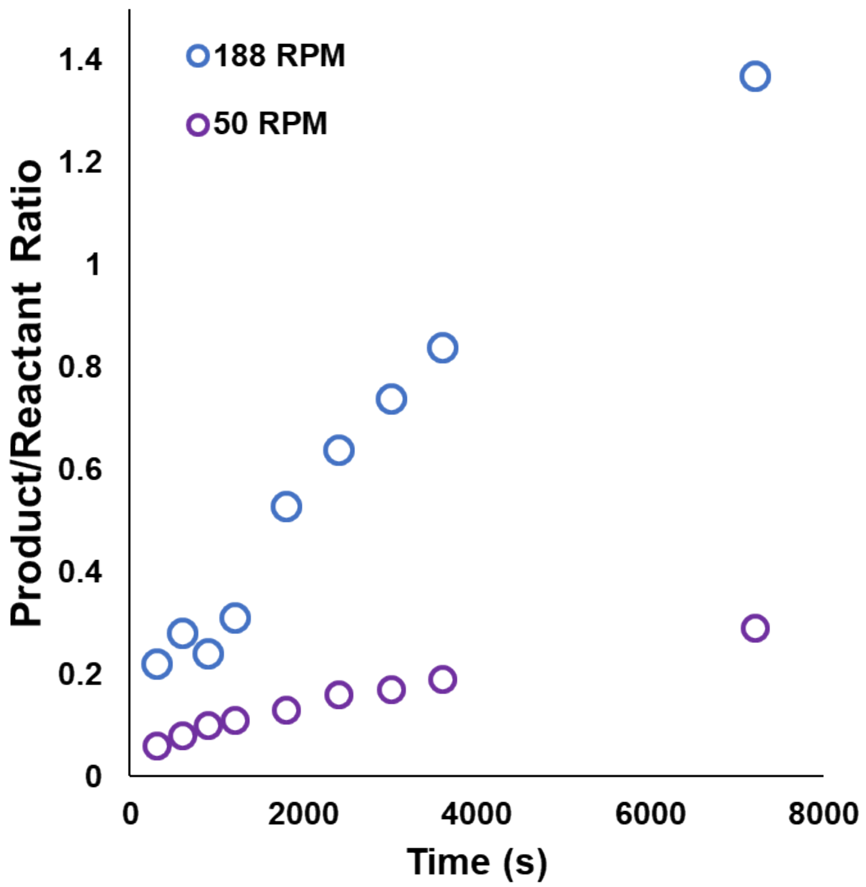

Figure S1 Reprinted from manuscript Scheme 15. Ratio between the integrated methoxy proton ^{1}H-NMR signal of 1-methoxy-4-(4-nitrophenoxy)benzene (product) and 4-methoxyphenol (substrate) versus time at 50 and 188 RPM stirrer speeds.

5.0 Nucleophilic Aromatic Substitution ¹H-NMR Spectra

5.1 4-Methoxyphenol

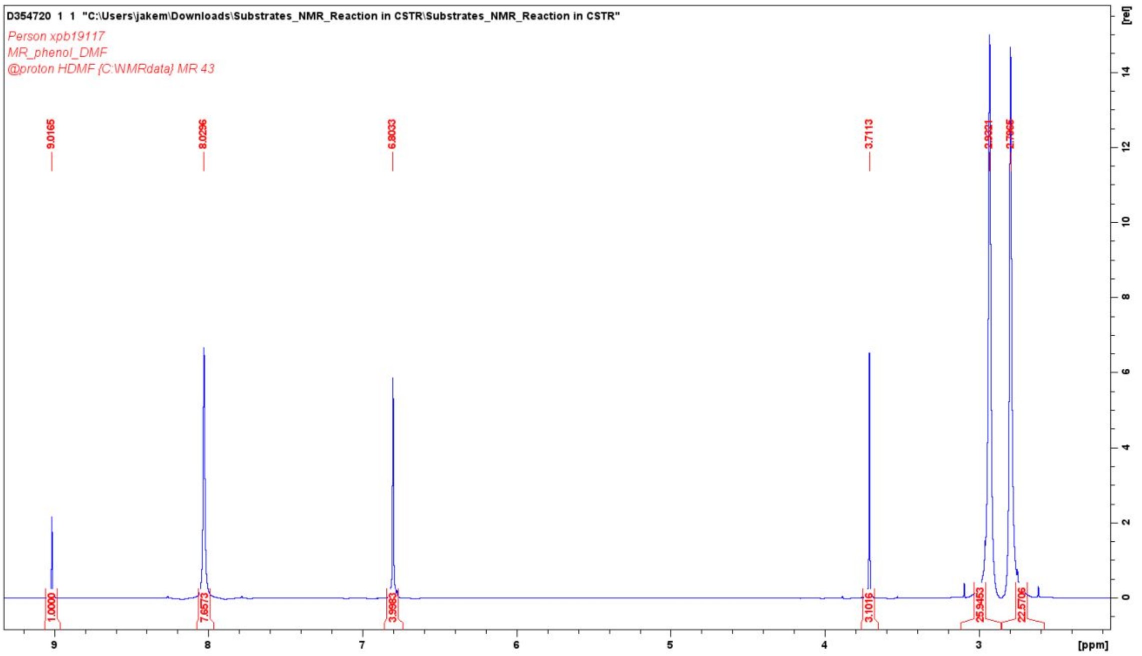

Figure S2 ¹H-NMR spectra of 4-methoxyphenol in non-deuterated DMF.

5.2 1-Fluoro-4-nitrobenzene

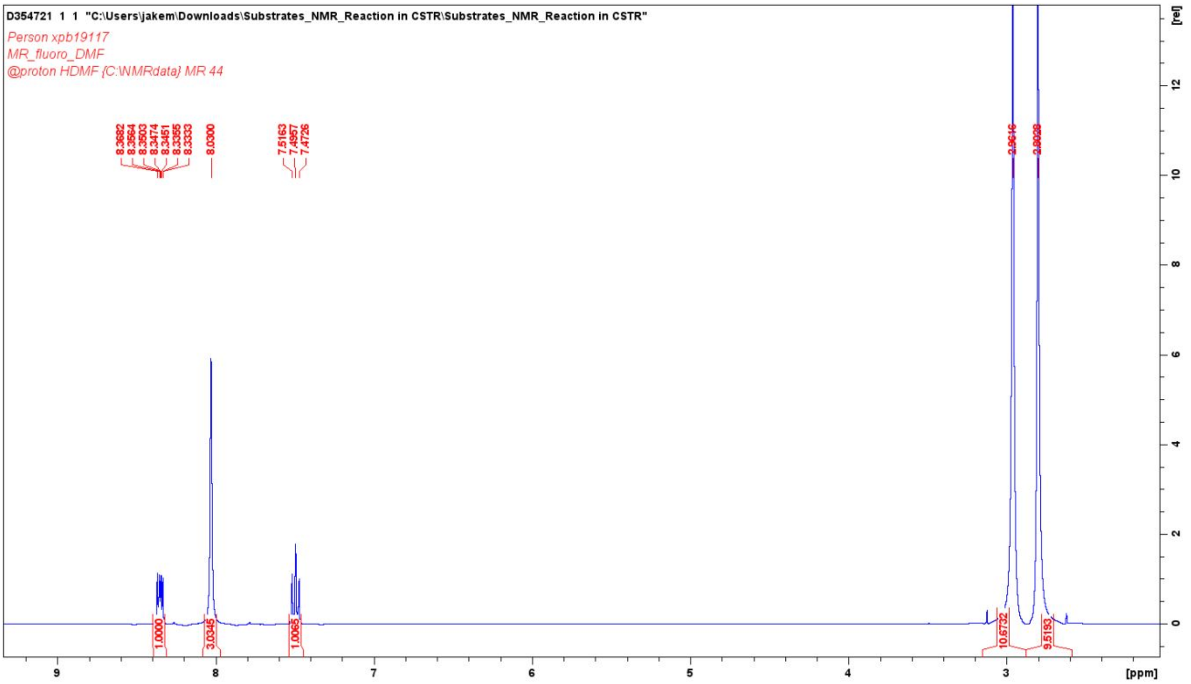

Figure S3 ¹H-NMR spectra of 1-fluoro-4-nitrobenzene in non-deuterated DMF.

5.3 50 RPM reactor

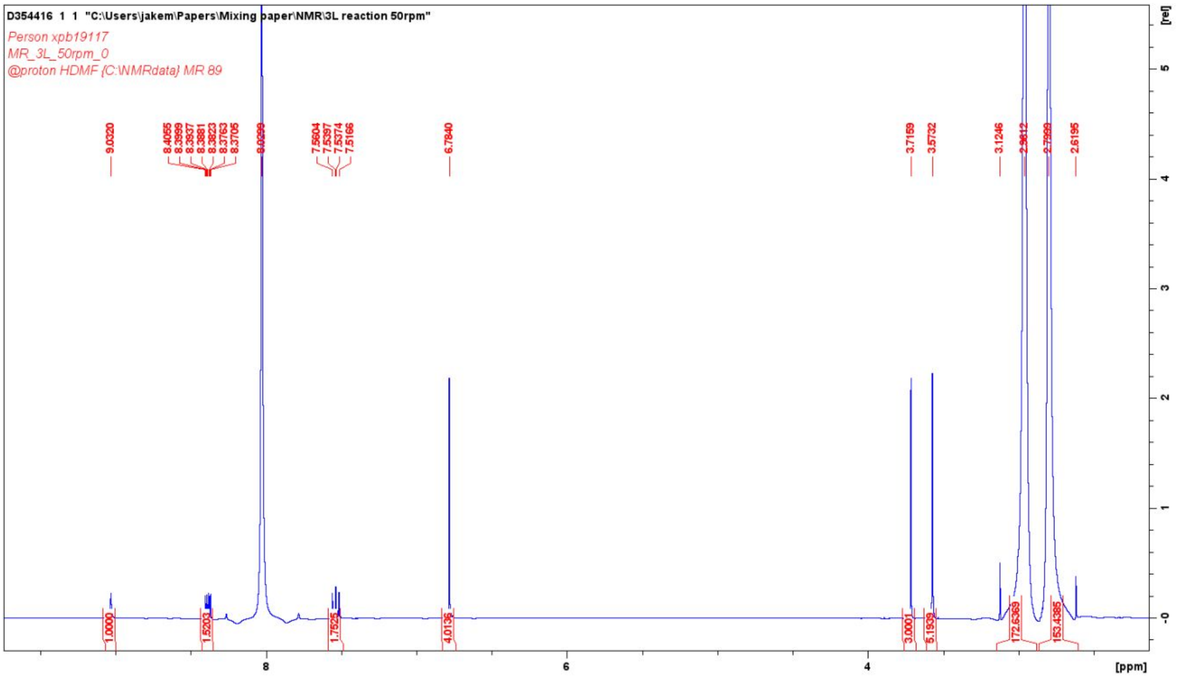

Figure S4 ^{1}H-NMR spectra of reaction mixture after quench at 0 minutes in non-deuterated DMF.

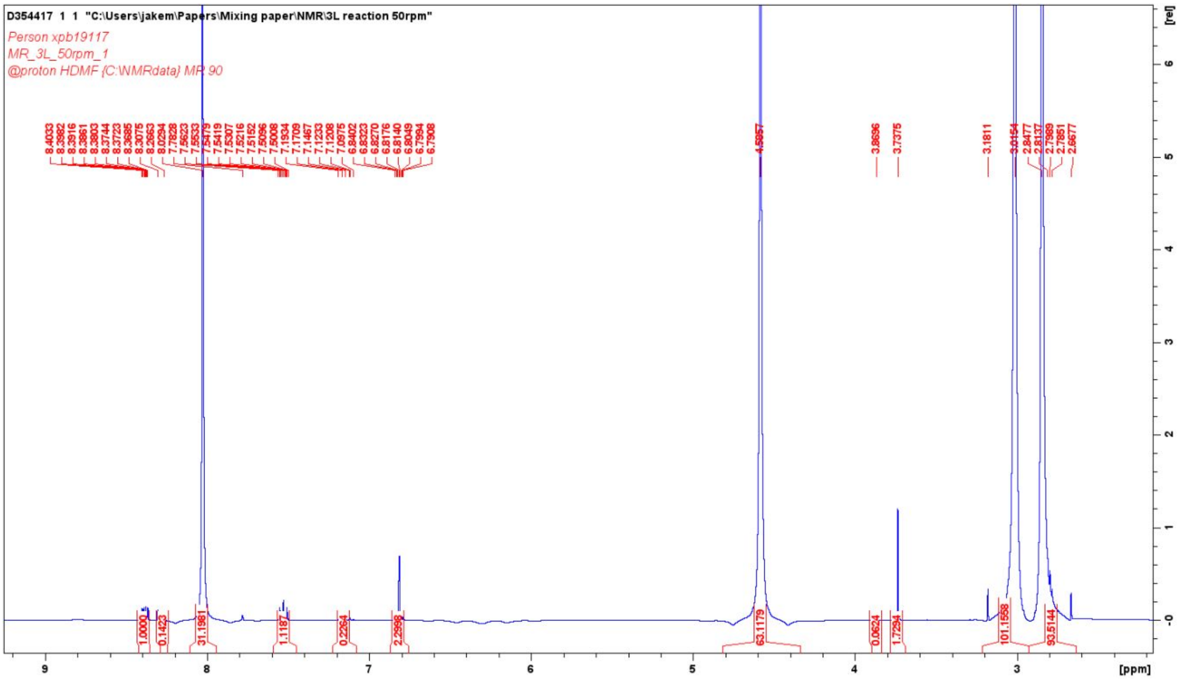

Figure S5 ^{1}H-NMR spectra of reaction mixture after quench at 5 minutes in non-deuterated DMF.

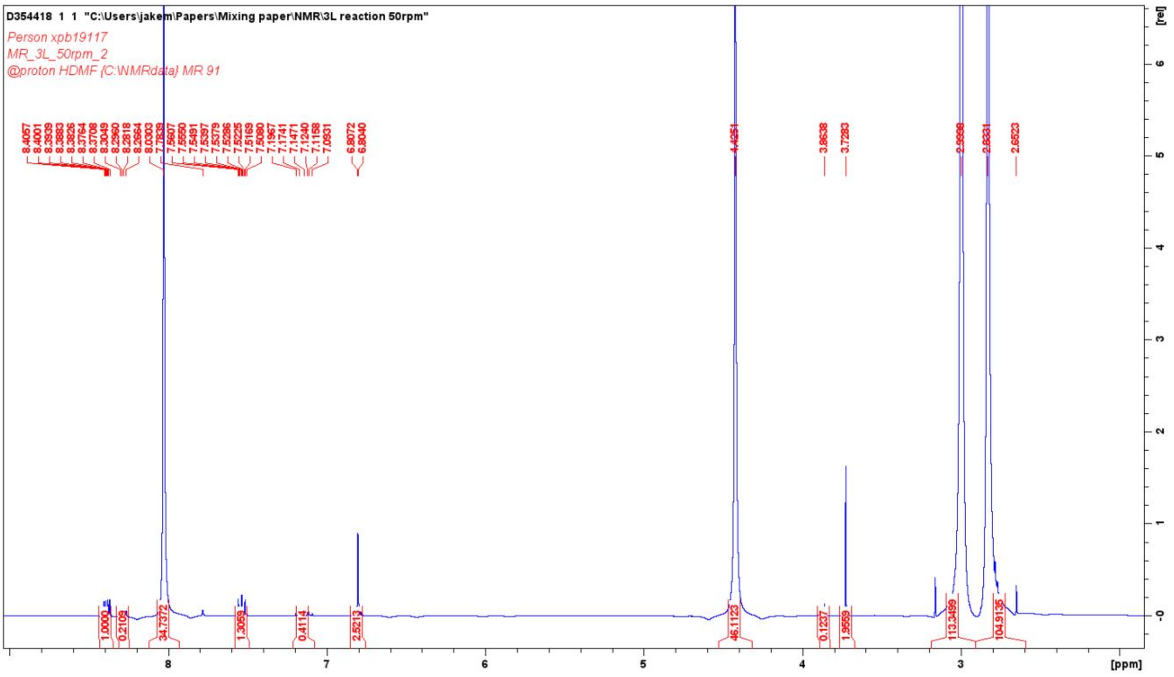

Figure S6 ^{1}H-NMR spectra of reaction mixture after quench at 10 minutes in non-deuterated DMF.

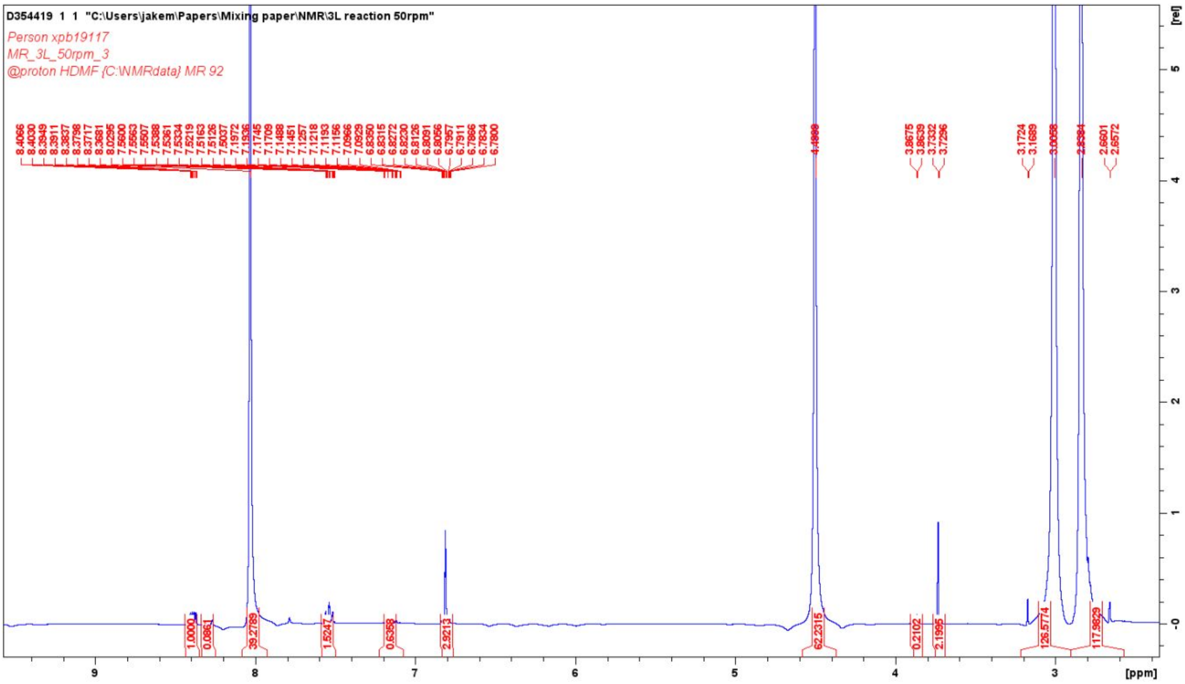

Figure S7 ^{1}H-NMR spectra of reaction mixture after quench at 15 minutes in non-deuterated DMF.

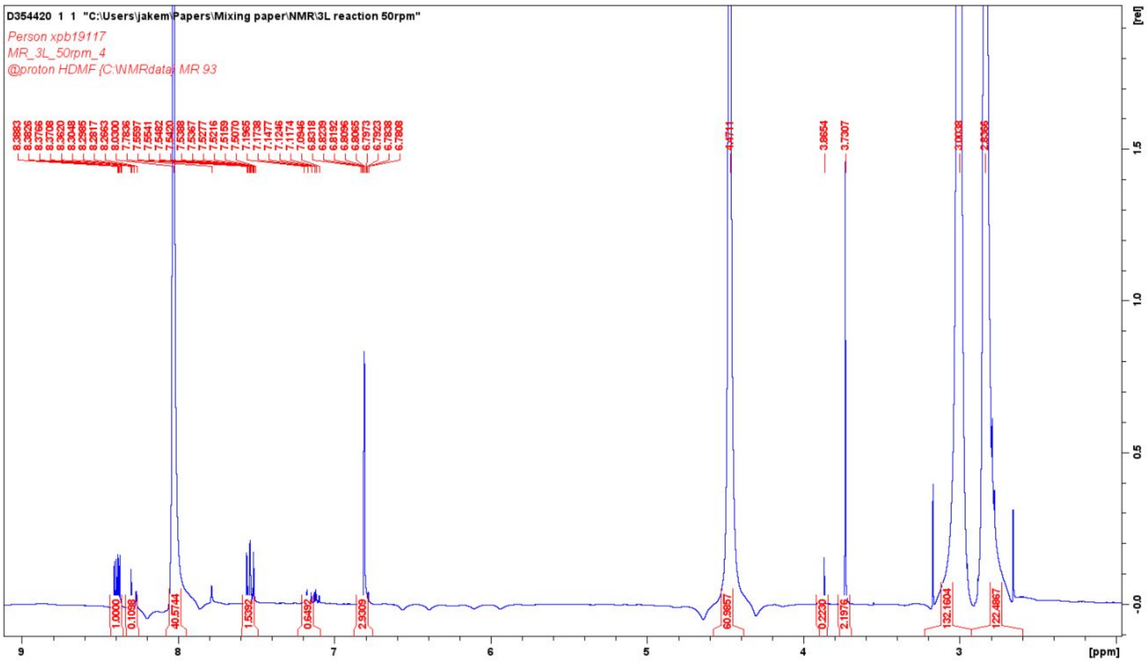

Figure S8 ^{1}H-NMR spectra of reaction mixture after quench at 20 minutes in non-deuterated DMF.

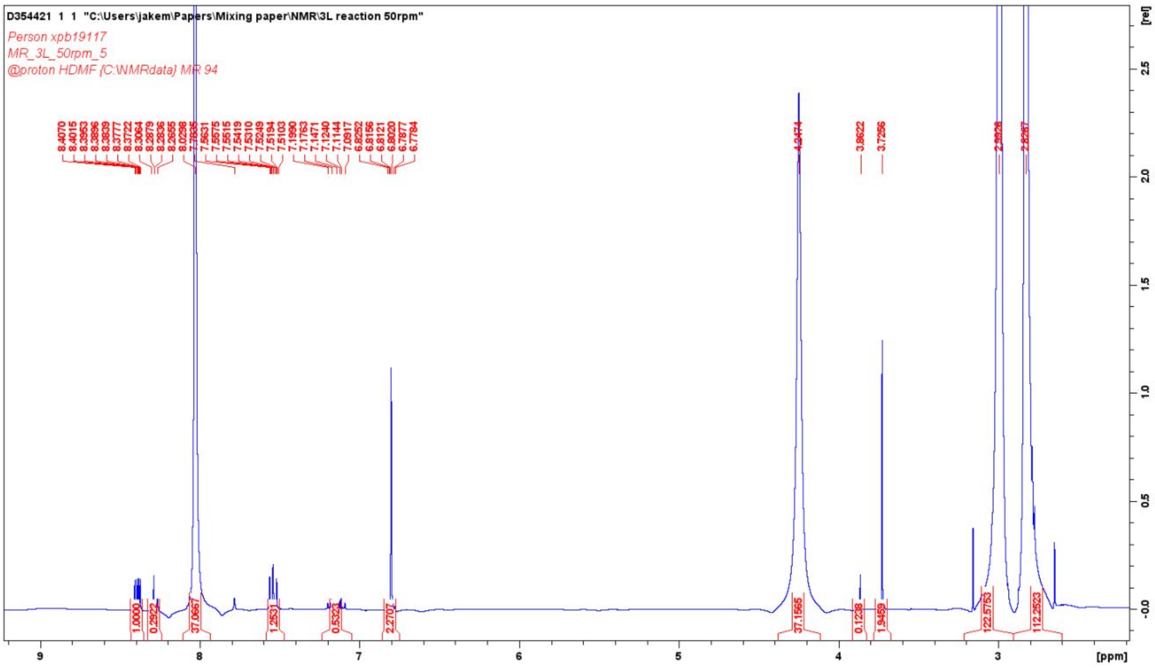

Figure S9 ^{1}H-NMR spectra of reaction mixture after quench at 30 minutes in non-deuterated DMF.

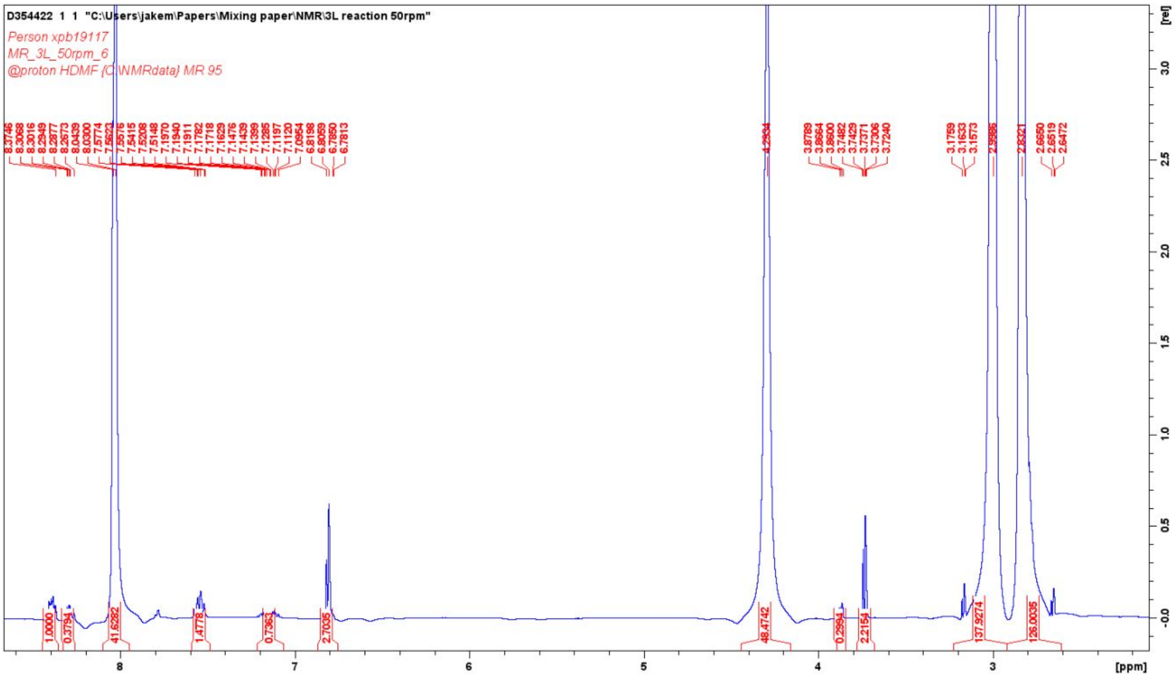

Figure S10 ^{1}H-NMR spectra of reaction mixture after quench at 40 minutes in non-deuterated DMF.

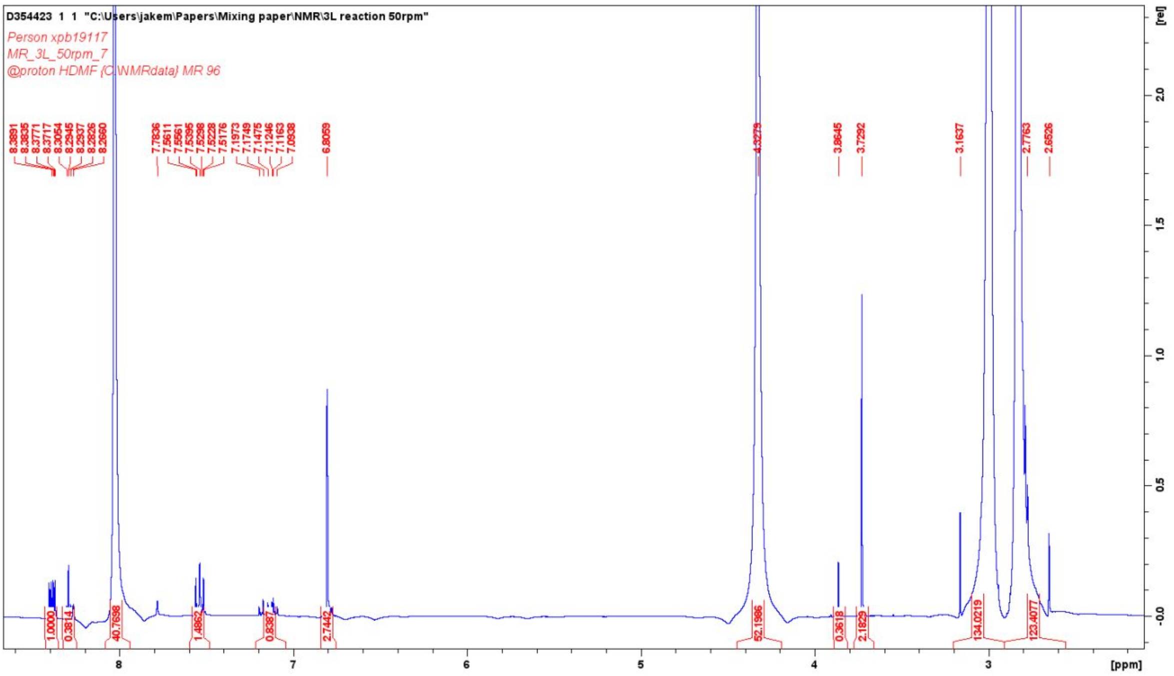

Figure S11 ^{1}H-NMR spectra of reaction mixture after quench at 50 minutes in non-deuterated DMF.

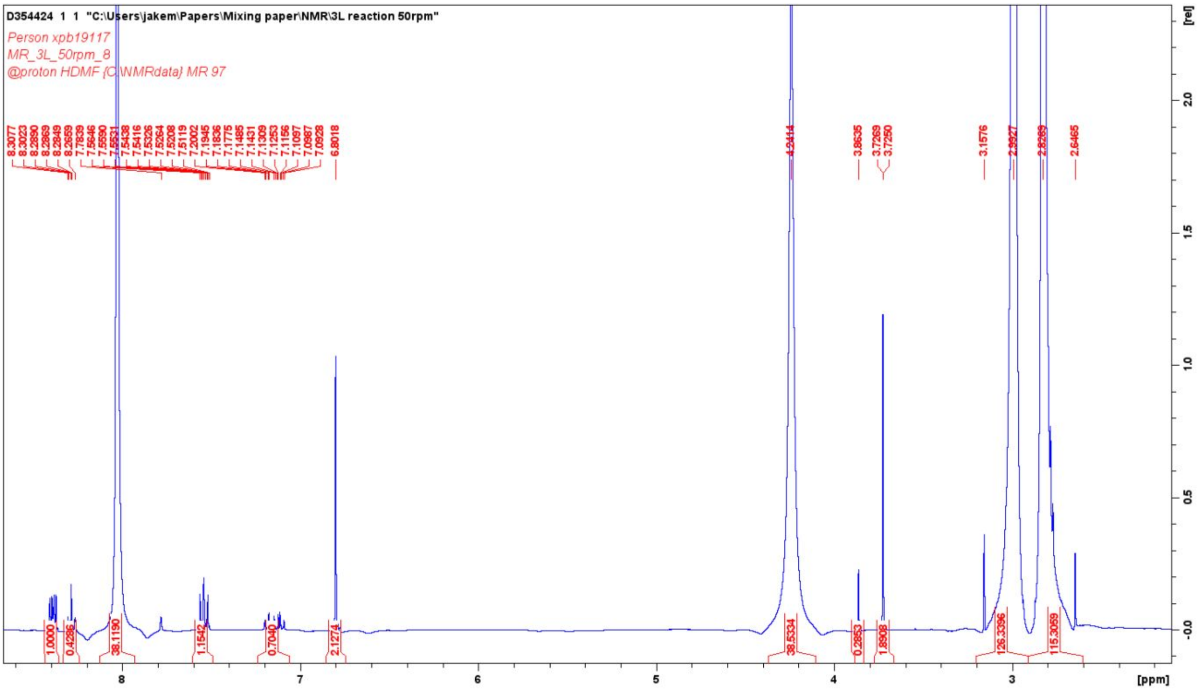

Figure S12 ^{1}H-NMR spectra of reaction mixture after quench at 60 minutes in non-deuterated DMF.

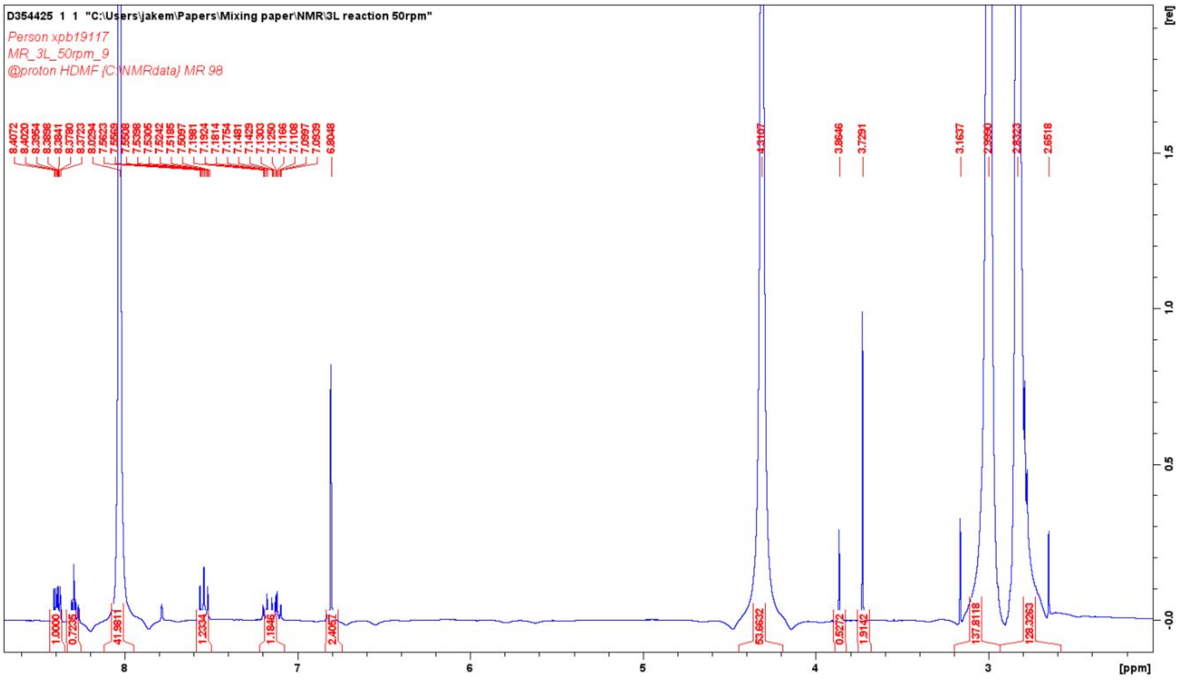

Figure S13 ^{1}H-NMR spectra of reaction mixture after quench at 120 minutes in non-deuterated DMF.

5.4 188 RPM reactor

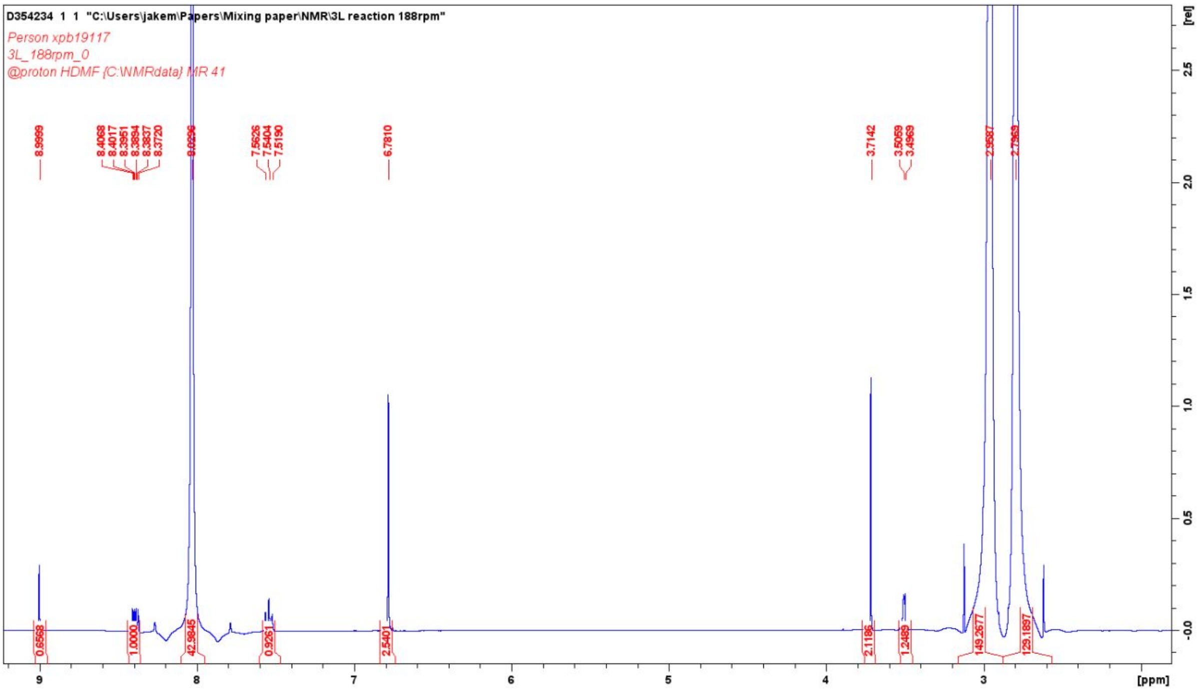

Figure S14 ^{1}H-NMR spectra of reaction mixture after quench at 0 minutes in non-deuterated DMF.

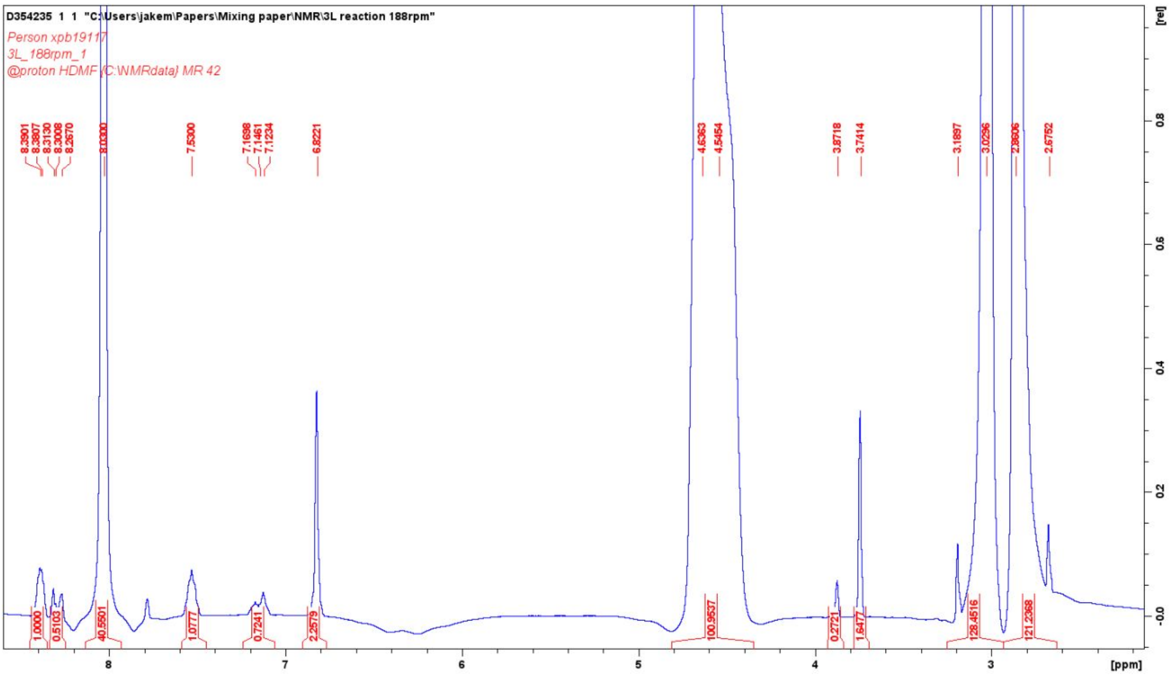

Figure S15 ^{1}H-NMR spectra of reaction mixture after quench at 5 minutes in non-deuterated DMF.

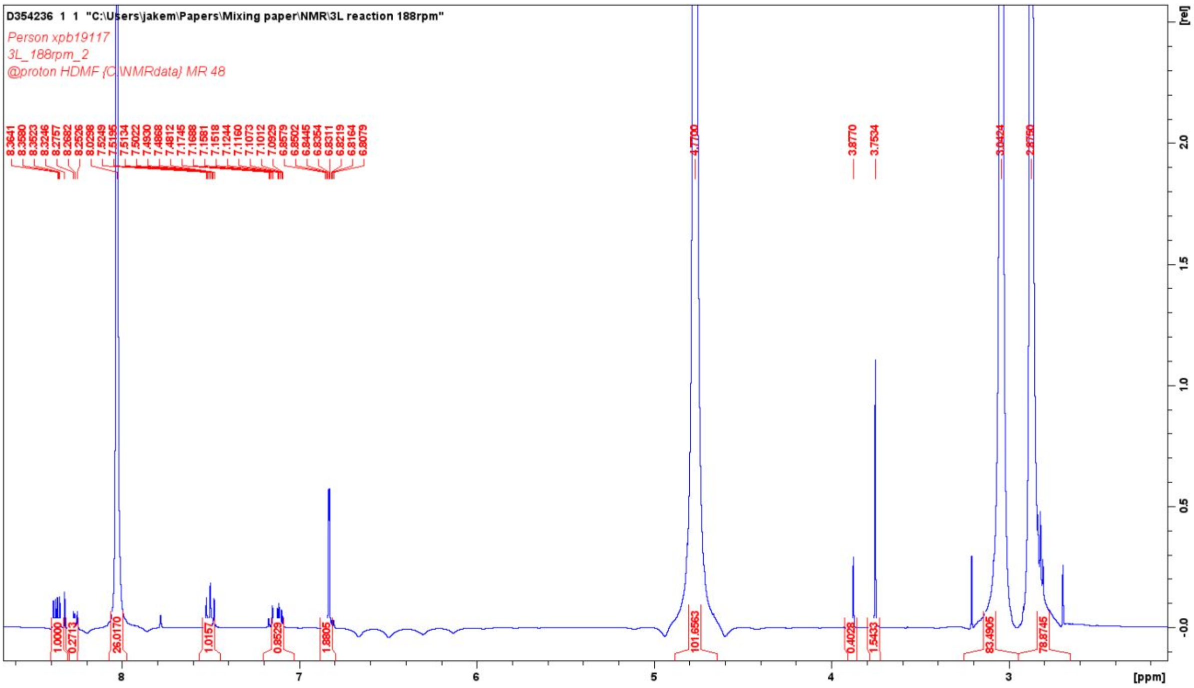

Figure S16 ^{1}H-NMR spectra of reaction mixture after quench at 10 minutes in non-deuterated DMF.

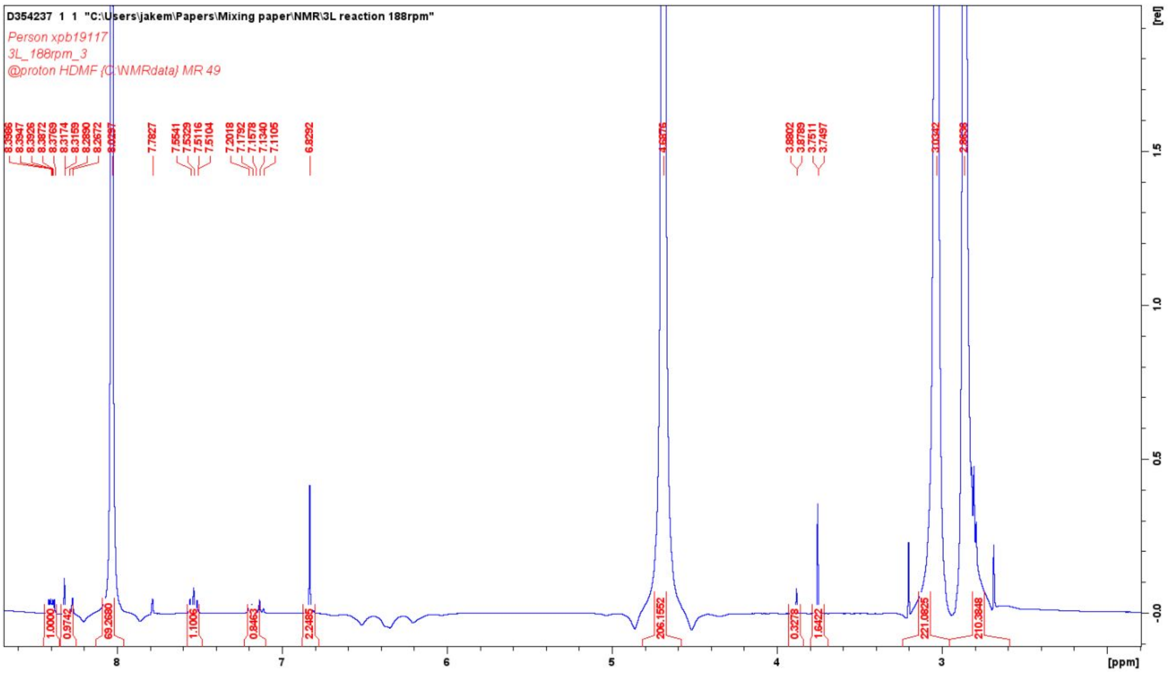

Figure S17 ^{1}H-NMR spectra of reaction mixture after quench at 15 minutes in non-deuterated DMF.

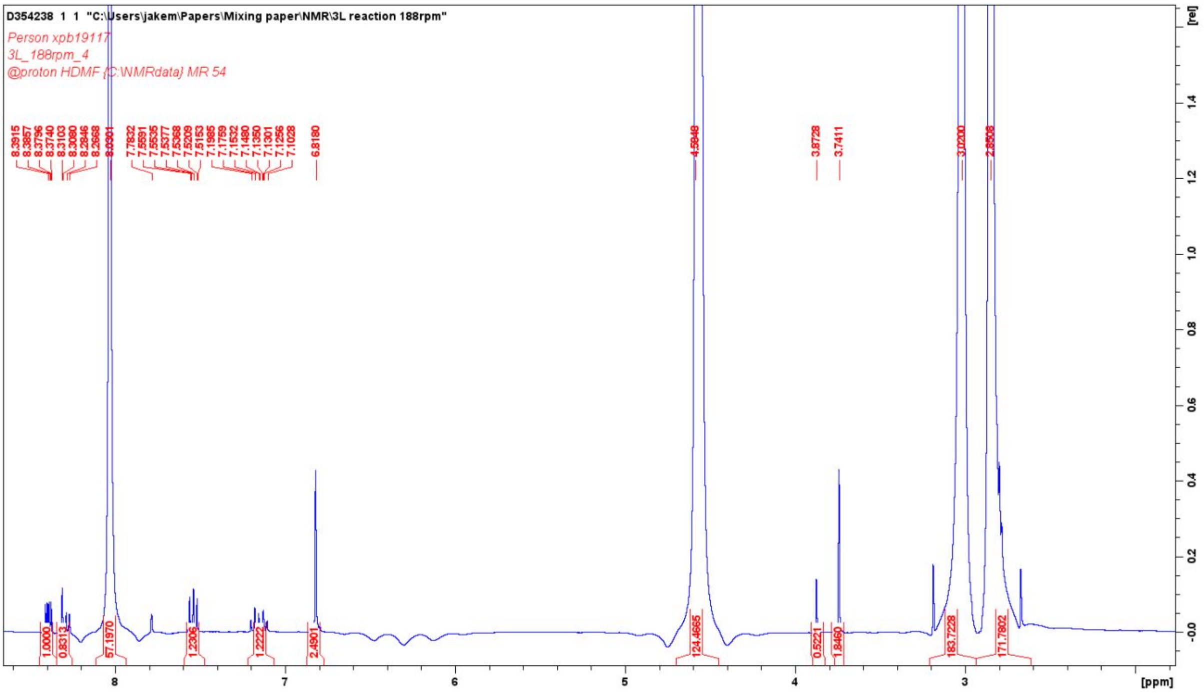

Figure S18 ^{1}H-NMR spectra of reaction mixture after quench at 20 minutes in non-deuterated DMF.

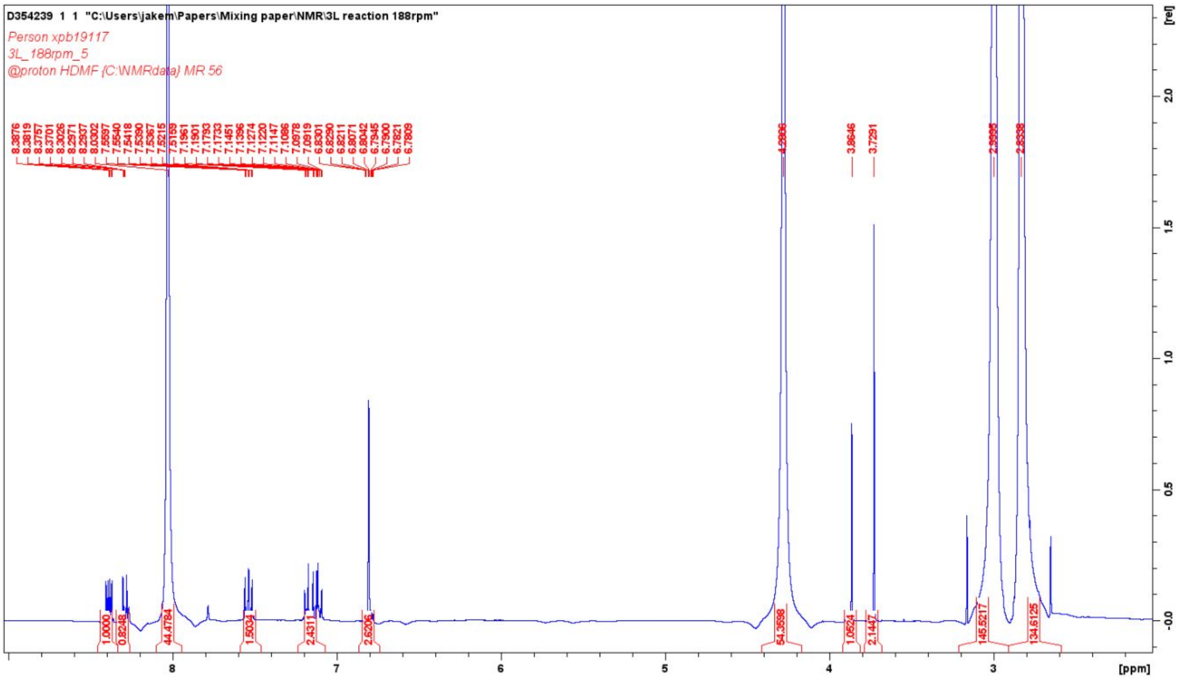

Figure S19 ^{1}H-NMR spectra of reaction mixture after quench at 30 minutes in non-deuterated DMF.

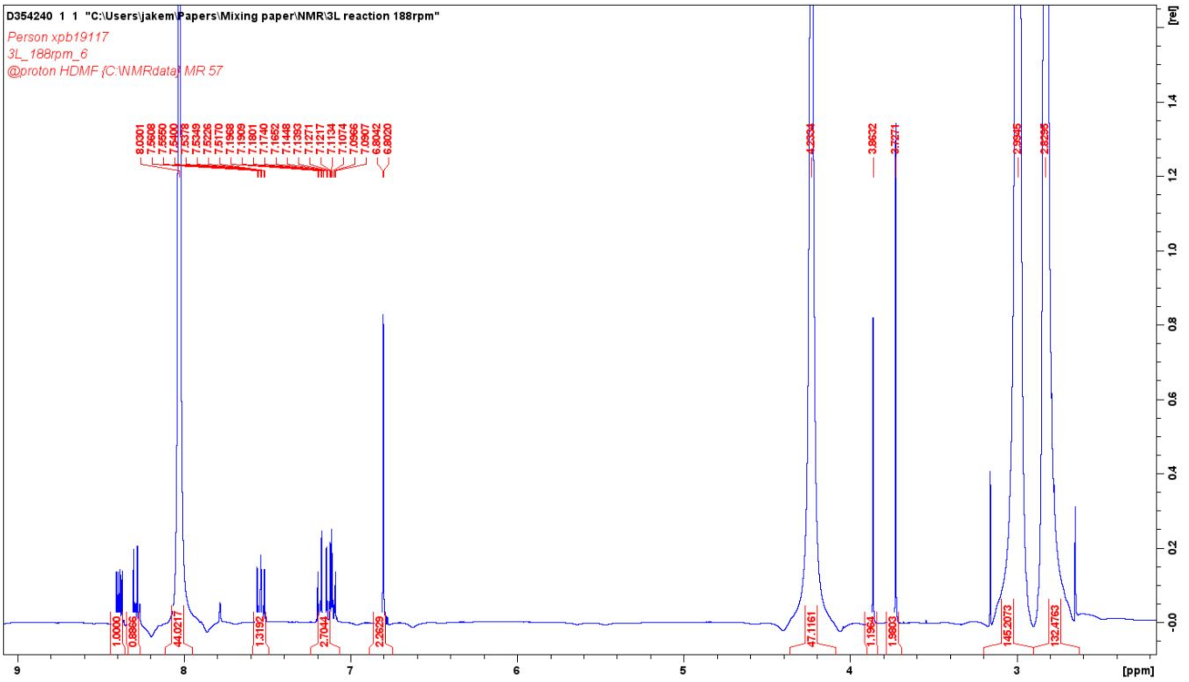

Figure S20 ^{1}H-NMR spectra of reaction mixture after quench at 40 minutes in non-deuterated DMF.

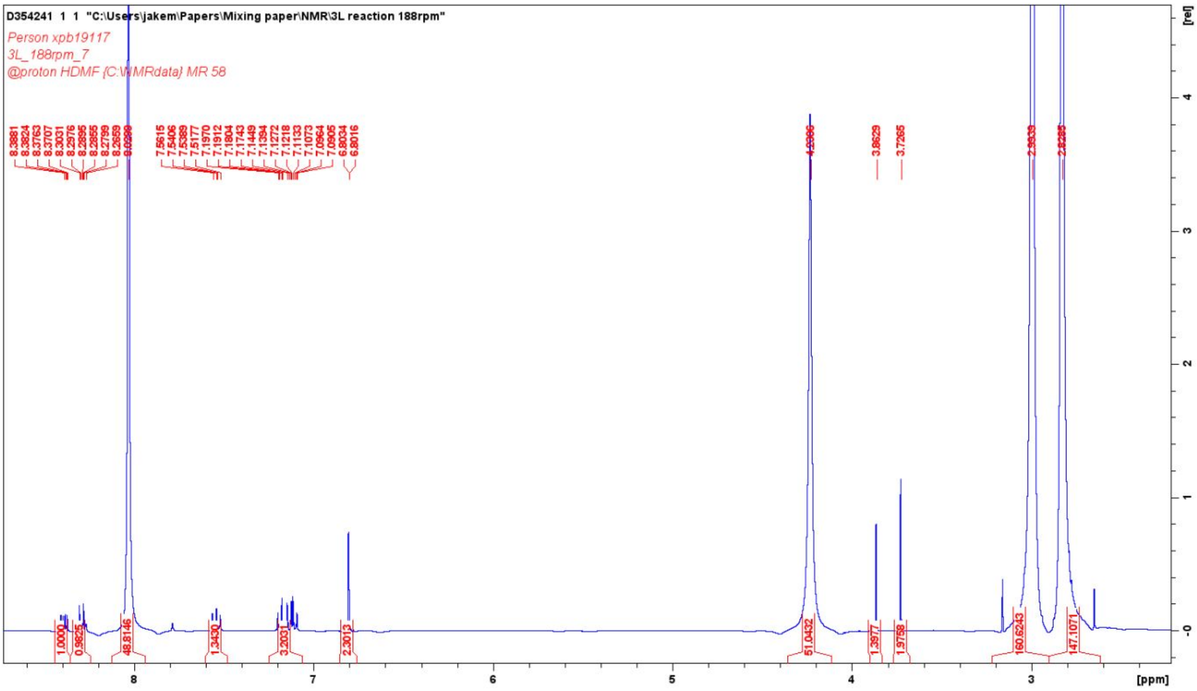

Figure S21 ^{1}H-NMR spectra of reaction mixture after quench at 50 minutes in non-deuterated DMF.

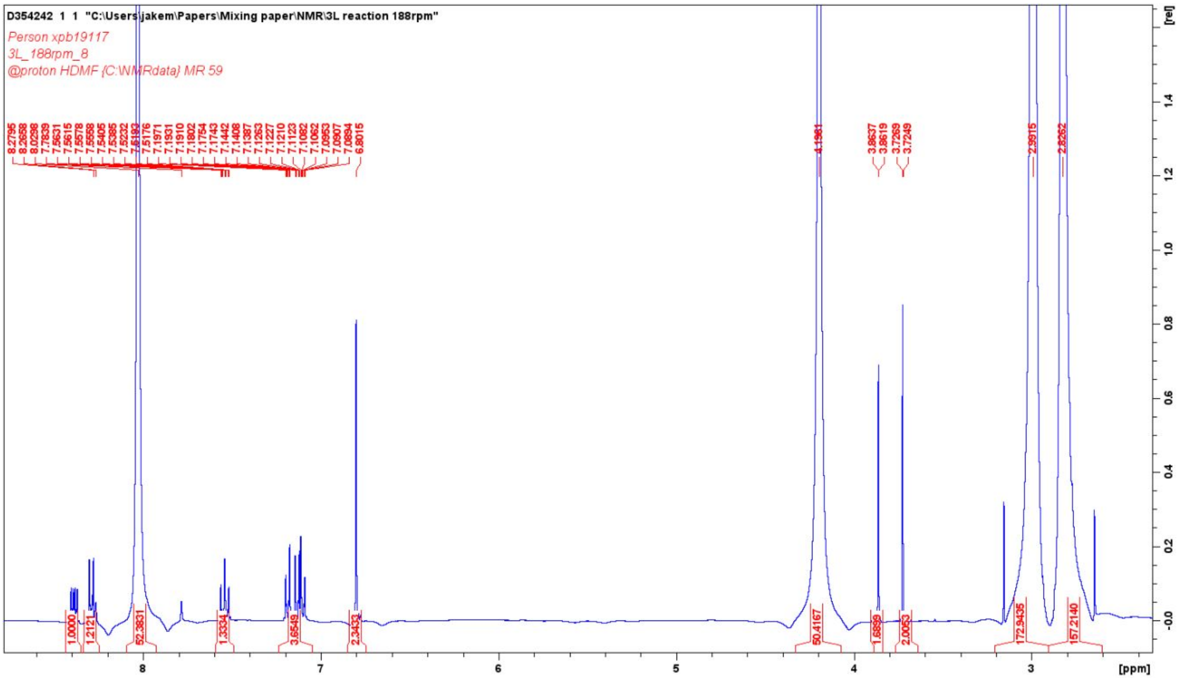

Figure S22 ^{1}H-NMR spectra of reaction mixture after quench at 60 minutes in non-deuterated DMF.

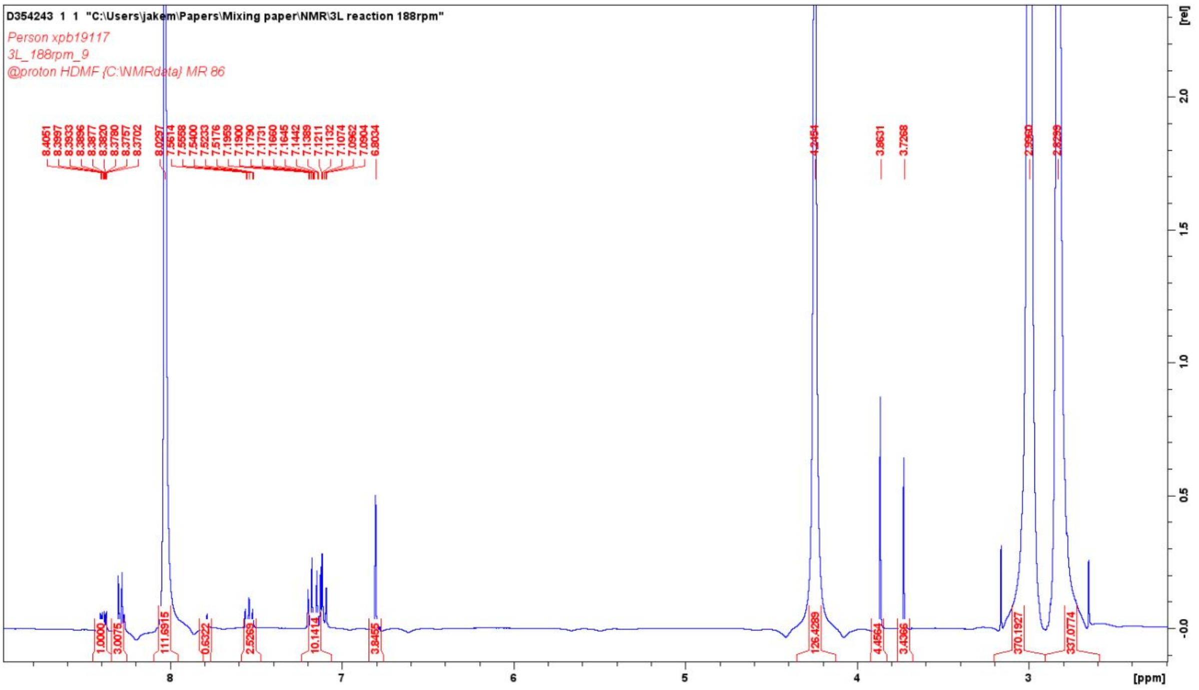

Figure S23 ^{1}H-NMR spectra of reaction mixture after quench at 120 minutes in non-deuterated DMF.

6.0 Kineticolor and Video Analysis

As described at the conceptual level in manuscript Scheme 1, and in our previous publication,[4] Kineticolor operates on the basis of video (mp4, mov, or avi) video input. The software breaks the video into its component frames and conducts a series of averaged and spatially-resolved computations based on pixel analysis.

All raw spreadsheet outputs for averaged and spatially-resolved mixing metrics, for all reactions discussed in the manuscript, are provided in an accompanying zipped folder of Excel files.

In all cases (except in cases specified in the folder), files contain the following tabs:

- Colour analysis – (averaged colour quantification versus time for the selected video region of interest (ROI).
- Mixing analysis – mixing metrics versus time for the selected ROI.
- Complete cell analysis – averaged colour channel analyses for all 25 square cells of pixels across the segmented ROI. AS a reminder, such segmentation was used to calculate, for example, ΔE by row or column and variance. See manuscript Scheme 5 (right).
- Config – the complete configuration of the analysis for a particular video and ROI. The Config tab stores:
    - Video Name
    - Video Path (wherein the original analysis was stored)
    - Frames per second (FPS) of collected video
    - Total number of frames
    - Video Length (s)
    - Step Interval or frame skip (the number of frames skipped between frames analysed).
    - Start Frame (to track any video trimming done before analysis)
    - Start Time
    - End Frame
    - End Time
    - Region of Interest Info (dimensions of the selected video area in which all calculations are carried out).
    - Input min X

- Input max X
- Input min Y
- Input max Y
- angle
- Rotated ROI bounds (information on regions of interest that are not parallel to the video frame)
- X Coordinate
- Y Coordinate
- Width
- Height
- Mixing Analysis Settings (e.g. any divergence from the default 5x5 segmentation of the ROI)
- Grayscale Contact Threshold
- GLCM
- Number of Gray Levels
- Pixel Pair Vector
- Cell Splitting
- Number of Cells
- Cell Height
- Cell Width

7.0 References

(1) Pichette Drapeau, M.; Ollevier, T.; Taillefer, M. On the Frontier Between Nucleophilic Aromatic Substitution and Catalysis. *Chemistry – A European Journal* **2014**, *20* (18), 5231–5236. https://doi.org/https://doi.org/10.1002/chem.201304164.

(2) Fitschen, J.; Hofmann, S.; Wutz, J.; Kameke, A. v.; Hoffmann, M.; Wucherpfennig, T.; Schlüter, M. Novel Evaluation Method to Determine the Local Mixing Time Distribution in Stirred Tank Reactors. *Chemical Engineering Science: X* **2021**, *10*, 100098. https://doi.org/https://doi.org/10.1016/j.cesx.2021.100098.

(3) Kölbl, A.; Kraut, M.; Schubert, K. The Iodide Iodate Method to Characterize Microstructured Mixing Devices. *AIChE Journal* **2008**, *54* (3), 639–645. https://doi.org/https://doi.org/10.1002/aic.11408.

(4) Yan, C.; Cowie, M.; Howcutt, C.; Wheelhouse, K. M.; Hodnett, N. S.; Kollie, M.; Gildea, M.; Goodfellow, M. H.; Reid, M. Computer Vision for Catalyst Degradation Kinetics. *ChemRxiv* **2022**. https://doi.org/10.26434/chemrxiv-2022-n0wf3.
