# Supplementary material for: Computer Vision for Kinetic Analysis of Lab- and Process-Scale Mixing Phenomena
Source: Org Process Res Dev. 2022 Nov 4;26(11):3073–88. doi: 10.1021/acs.oprd.2c00216 (PMC9680030; doi:10.1021/acs.oprd.2c00216)
Supplement: Supplementary file 2 — op2c00216_si_003.pdf [file op2c00216_si_003.pdf]

Computer Vision for Kinetic Analysis of Lab- and Process-scale Mixing Phenomena

Henry Barrington, ‡[a] Alan Dickinson,[b] Jake McGuire, ‡[a] Chunhui Yan, ‡[a] and Marc Reid*[a]

a. Department of Pure & Applied Chemistry, University of Strathclyde, Royal College Building
204 George St, Glasgow G1 1XW.
b. Colorants Technology Centre, FUJIFILM Imaging Colorants, Earls Road, Grangemouth, FK3 8XG

‡These authors contributed equally.

Computational Supporting Information

Contents

1. Mixing Metrics Overview 4

2. Additional Discussion on Calculated Metrics and Level of Quantification 4

3. Model Development – Polka Dot Video Simulations 5

Data output for base polka dot video 6

Data output for polka dot with grey obstructions representing baffles in reactor 11

Data output for polka dot with white obstructions representing glass glare 16

Comparison of different polka dot spacings to build ASM and Homogeneity Intuition 20

4. Qualitative Visualization of Mixing Phenomena in Plant Mimic Vessels 23

Table 1, Entry 1: 60 RPM, Paddle, No baffles, No probe 24

Table 1, Entry 1: 60 RPM, Paddle, No baffles, with probe 29

Table 1, Entry 2: 100 RPM, Paddle, No baffles, with probe 34

Table 1, Entry 2: 210 RPM, Paddle, No baffles, with probe 39

Table 1, Entry 2: 100 RPM, Paddle, No baffles 44

Table 1, Entry 3: 210 RPM, Paddle, No baffles 49

Table 1, Entry 4: 60 RPM, Anchor, No baffles 54

Table 1, Entry 4: 60 RPM, Anchor, No baffles, with probe 59

Table 1, Entry 5: 100 RPM, Anchor, No baffles 64

Table 1, Entry 5: 100 RPM, Anchor, No baffles, with probe...69
Table 1, Entry 6: 210 RPM, Anchor, No baffles...74
Table 1, Entry 6: 210 RPM, Anchor, No baffles, with probe...79
Table 1, Entry 7: 60 RPM, paddle, with baffle...84
Table 1, Entry 8: 100 RPM, paddle, with baffle, no probe...89
Table 1, Entry 9: 210 RPM, paddle, with baffle, no probe...94
Table 1, Entry 10: 100 RPM, anchor, no baffle, no probe...99
Table 1, Entry 10: 100 RPM, anchor, with baffle, no probe...104
Table 1, Entry 11: 210 RPM, anchor, no baffle, no probe...109
Table 1, Entry 11: 210 RPM, anchor, with baffle, no probe...114
Exemplification of choosing different-colored vessel backgrounds...118
5. pH Titrations as a Model System for Kinetic Imaging of Mixing Phenomena...123
5.1 Phenolphthalein titrations...123
phenolphthalein titration with no baffle in reactor...123
phenolphthalein titration with baffle in reactor...128
5.2 Bromothymol blue titrations...133
50 mL Schlenk tube, large oval stirrer bar (15x6 mm)...133
50 mL Schlenk tube, micro-stirrer bar (5x2 mm)...138
50 mL round bottom flask, large oval stirrer bar (15x6 mm)...143
50 mL round bottom flask, micro-stirrer bar (5x2 mm)...148
3 L beaker, no baffles or stirring...153
5 L STR, paddle, no probe...158
5 L STR, paddle, with probe...163
5 L STR, anchor, no probe...168
5 L STR, anchor, with probe...173
50 RPM stirring rate...178
200 RPM stirring rate...183
7. Application of Mixing Data in Highly Mixing Sensitive Chemistries...188
50 RPM...188
188 RPM (full reactor analysis)...193
188 RPM (top ‘layer’ analysis; average colour analysis only)...198
8. Mutual Information and Color-Concentration Regression Analyses...199
9. Further Details on Video Analysis...200

10. References..201

1. Mixing Metrics Overview

Mixing Metric	Formalism	What it captures
Contact	Greyscale thresholding for binary images via: $a_{ij} = \begin{cases} 1, & if\ a_{ij} > threshold \\ 0, & if\ a_{ij} < threshold \end{cases}$	Total perimeter around black-white pixel boundaries.
Contrast	$\sum_{i=0}^{N-1}\sum_{j=0}^{N-1} \lvert i-j \rvert^2 . p_{ij}$ $p_{ij} = \frac{a_{ij}}{\sum_{i=0}^{N-1}\sum_{j=0}^{N-1} a_{ij}} = \frac{a_{ij}}{grandsum(GLCM)}$ $\sum(p_{ij}) = 1$	The magnitude of grey level contrast for an ensemble of pixel pairs, where pixel pairs are defined by a specific pair of relative pixel locations.
Homogeneity	$= \sum_{i=0}^{N-1}\sum_{j=0}^{N-1} \frac{p_{ij}}{1 + \lvert i-j \rvert}$	Similarity of color across an image.
Angular Second Moment (ASM) or Energy	$ASM = \sum_{i=0}^{N-1}\sum_{j=0}^{N-1} (p_{ij}^2)$	Block color and level of order in an image.
ΔE or Delta E	$\Delta E = \sqrt{(L_2^* - L_1^*)^2 + (a_2^* - a)^2 + (b_2^* - b_1^*)^2}$	The contrast between the current frame or image and the reference frame (usually at time-zero).
Variance	$Variance = \sum_{g=1}^{\max(g)}\sum_{h=1}^{\max(h)} \frac{(\overline{C_{gh}} - \bar{G})^2}{[\max(g) + \max(h) - 1]}$ $\bar{C} = average\ pixel\ value\ for\ a\ cell$ $\bar{G} = overall\ average\ for\ all\ grid\ cells$ *max (g) = max (h) = 5 (by default)*	For a chosen colour component, this is the sum of squared differences for all pairwise differences of a cell and the overall grid average.
$The\ definition\ of\ p_{ij}\ for\ Contrast\ holds\ for\ the\ other\ GLCM\ metrics\ (Homoegeneity\ and\ ASM).$		

2. Additional Discussion on Calculated Metrics and Level of Quantification

The mixing metrics used in this report produce a mostly semi-quantitative view of the mixing process over time. Since the values calculated are determined by the colour values in each frame, the metrics can vary depending on the lighting of the mixture, the colour of the mixture under study, or the background in the video. For these reasons, the focus of the present work has been to exemplify the value of the kinetic (time-based) component of such computer vision methods.

The possible range of output color values is also dependent on the size of the selected region of interest (ROI). While it is best to control these elements where possible, it would be inadvisable to rely on these ROI dimensions alone for a direct comparison between videos. Indeed, in manuscript Scheme 15, the ability to select different ROI sizes, enabling multiple analyses of a single video, was used to more fully explore color-concentration correlations based on the spatially-resolved mixing phenomena at play in the S_NAr reactions.

Six metrics were used to analyse each video, calculated based on the same selection of frames, and the same region of interest.

3. Model Development – Polka Dot Video Simulations

Using one of several available and open source images revealed by a search engine term "polka dot red white", the saved PNG image was imported into Adobe Premier Pro 2022 and a 5 second video time set.

From available video efforts in Premier Pro, a Camera Blur effect was layered on top of the polka dot image. The blur progress from 0 to 100% over the 5 second video.

The resulting composition was exported as an mp4 video file for use as input for computer vision analysis in Kineticolor.

Variations on the original video were created by adding grey rectangles on top of the base polka dot pattern to represent reactor baffles. Similarly, a third variation was created by layering a white rectangle on top of the base polka dot pattern, the resulting video then being used to build intuition on glare effects.

For all video analysis, raw spreadsheet outputs are provided in a supplementary zipped folder.

Data output for base polka dot video

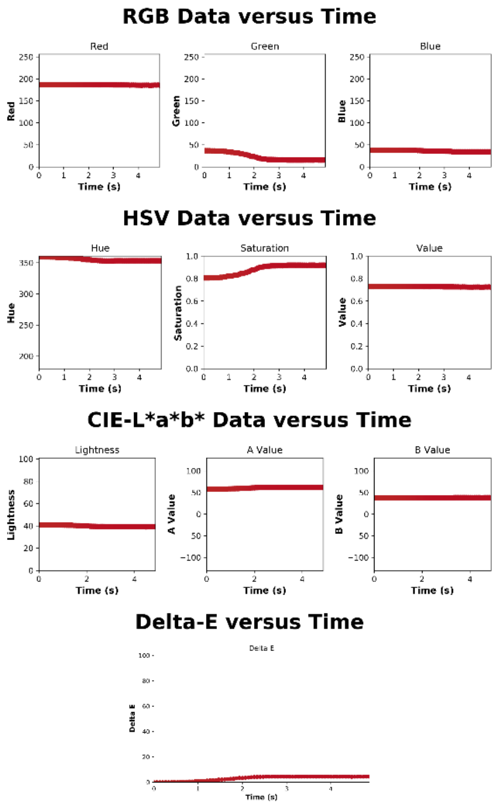

Kineticolor

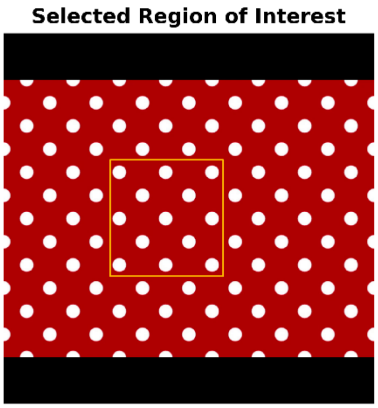

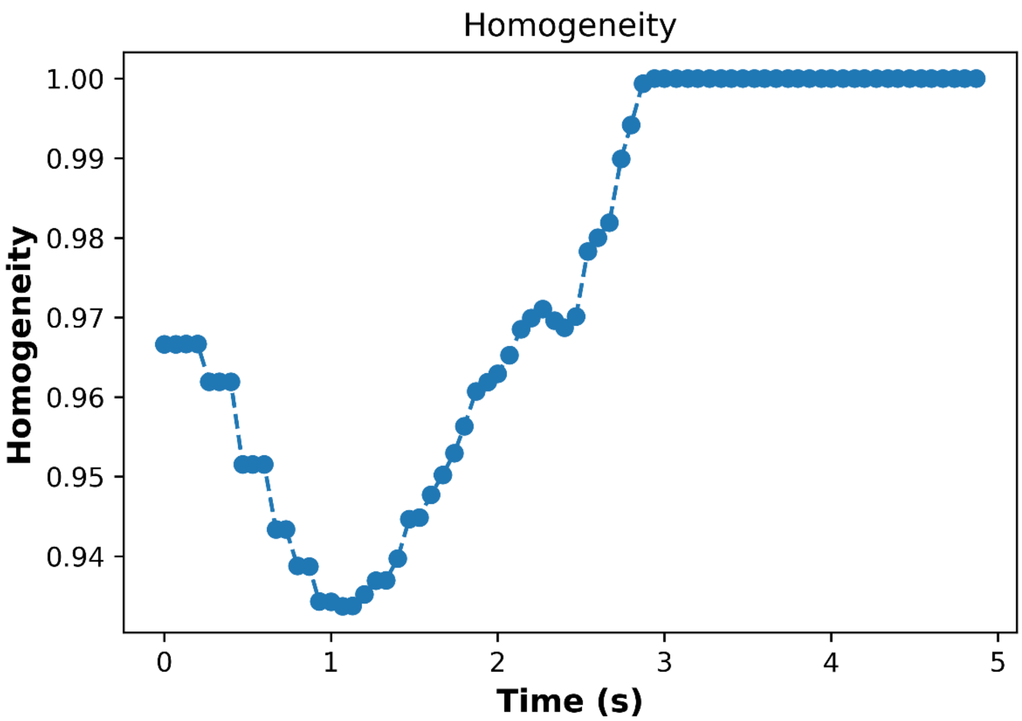
Homogeneity
1.00
0.99
0.98
0.97
0.96
0.95
0.94
Homogeneity
0
1
2
3
4
5
Time (s)

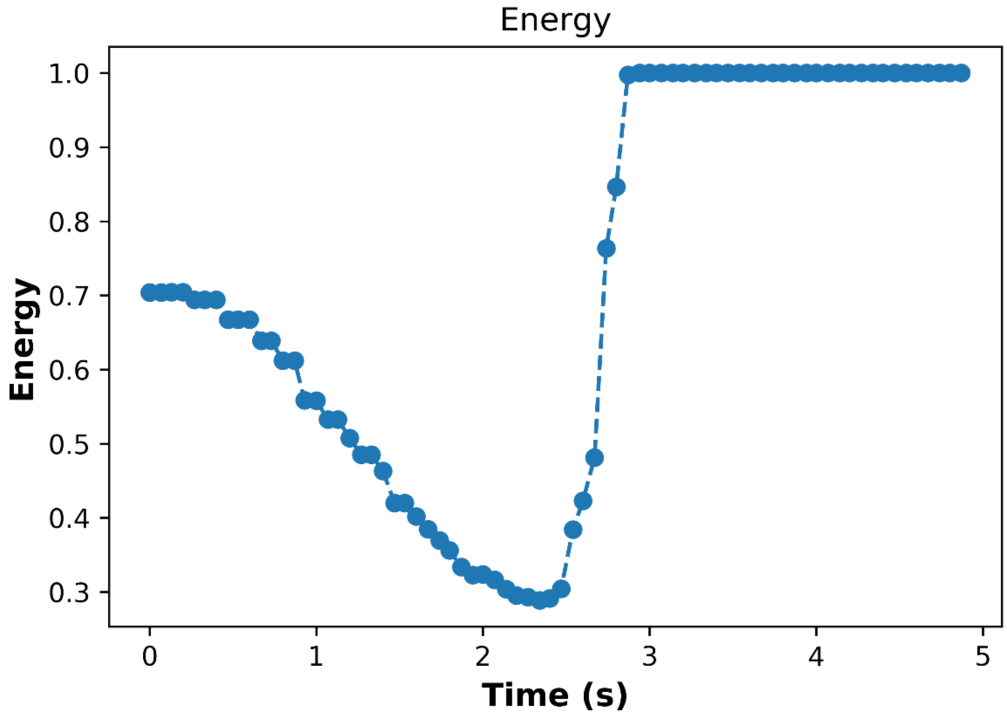
Energy
1.0
0.9
0.8
0.7
0.6
0.5
0.4
0.3
Energy
0
1
2
3
4
5
Time (s)

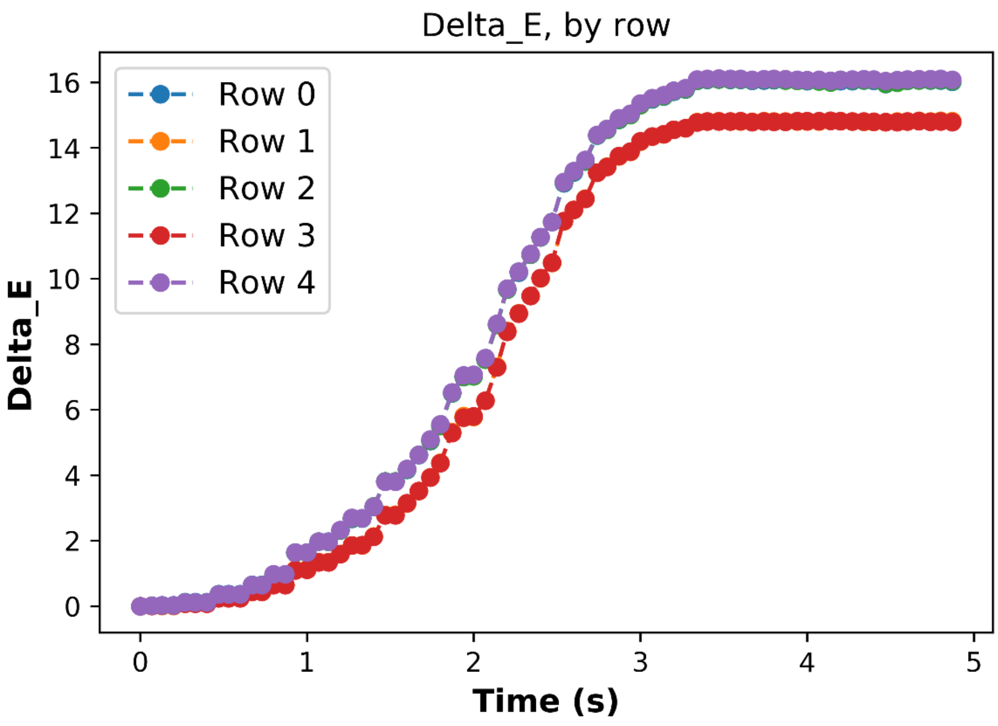
Delta_E, by row
Delta_E
Time (s)
Row 0
Row 1
Row 2
Row 3
Row 4
0
2
4
6
8
10
12
14
16
0
1
2
3
4
5

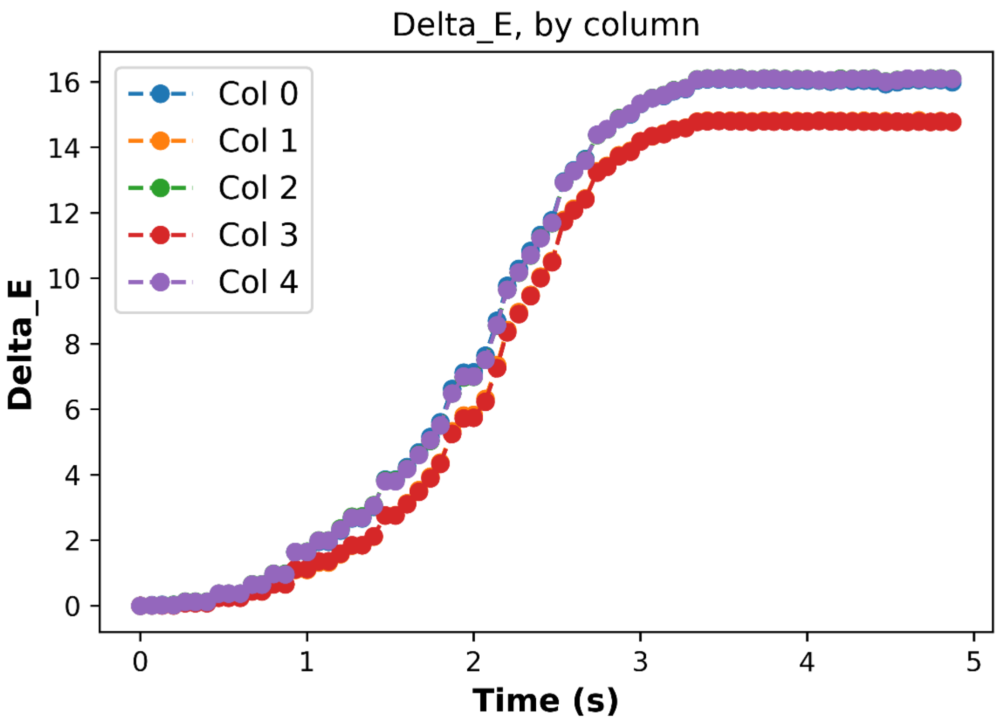
Delta_E, by column
Delta_E
Time (s)
Col 0
Col 1
Col 2
Col 3
Col 4
0
2
4
6
8
10
12
14
16
0
1
2
3
4
5

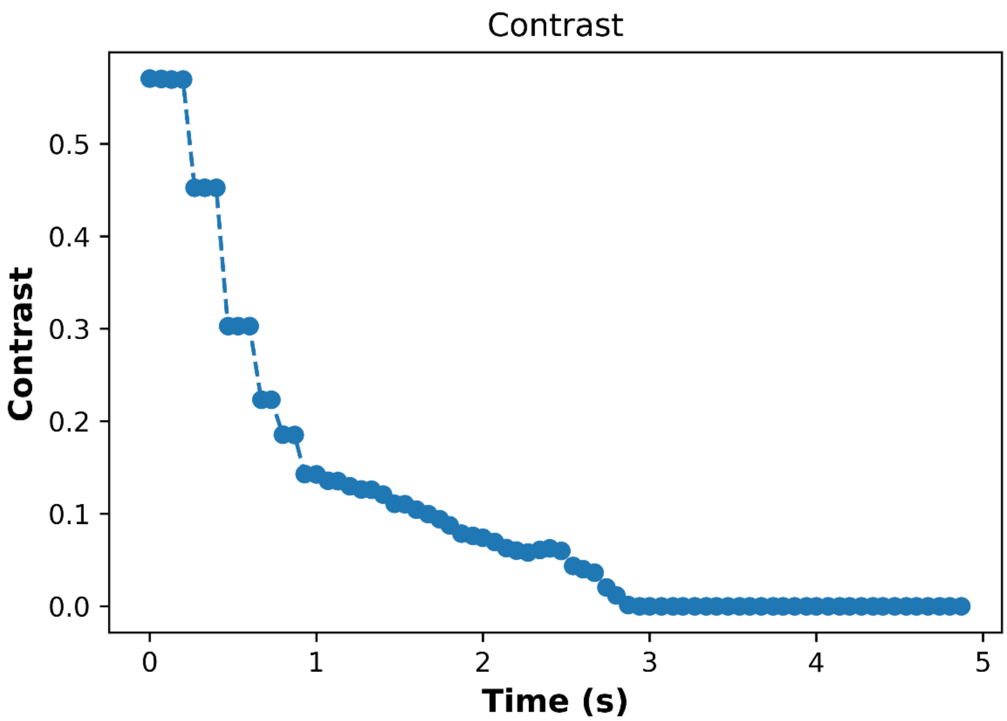
Contrast
0.5
0.4
0.3
0.2
0.1
0.0
Contrast
0
1
2
3
4
5
Time (s)

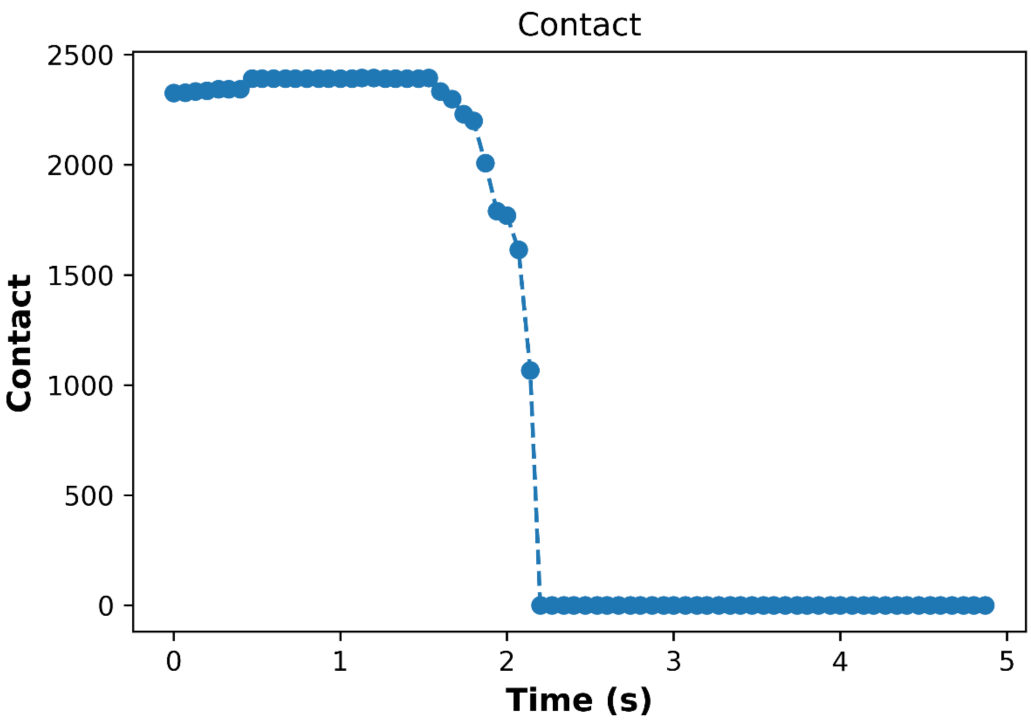
Contact
2500
2000
1500
1000
500
0
Contact
0
1
2
3
4
5
Time (s)

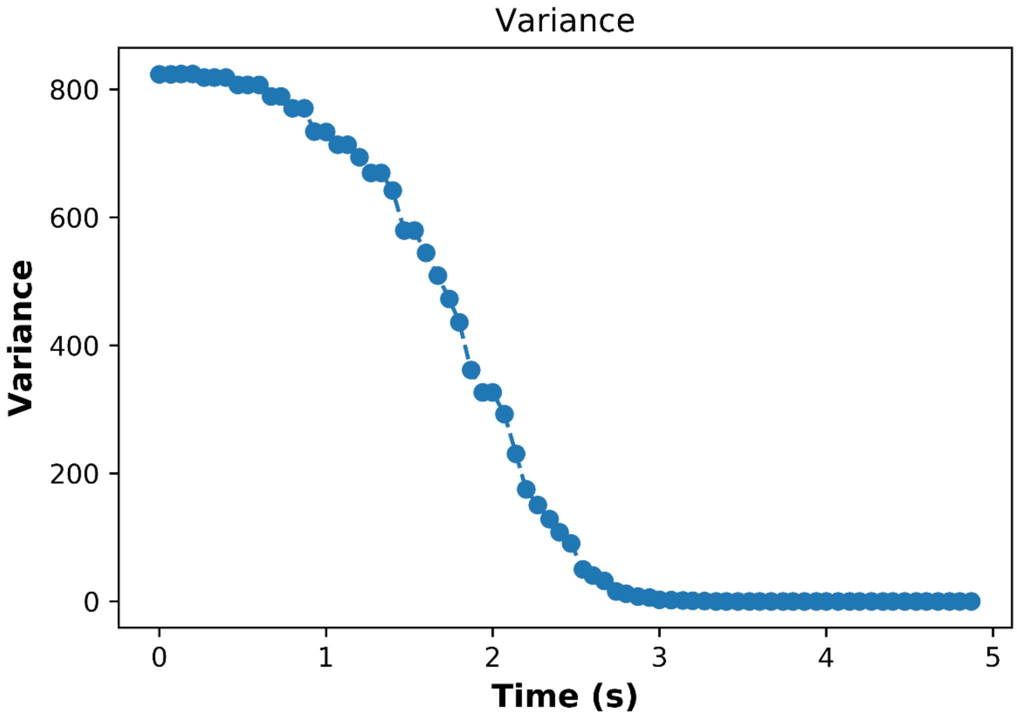
Variance
800
600
400
200
0
Variance
0
1
2
3
4
5
Time (s)

Data output for polka dot with grey obstructions representing baffles in reactor

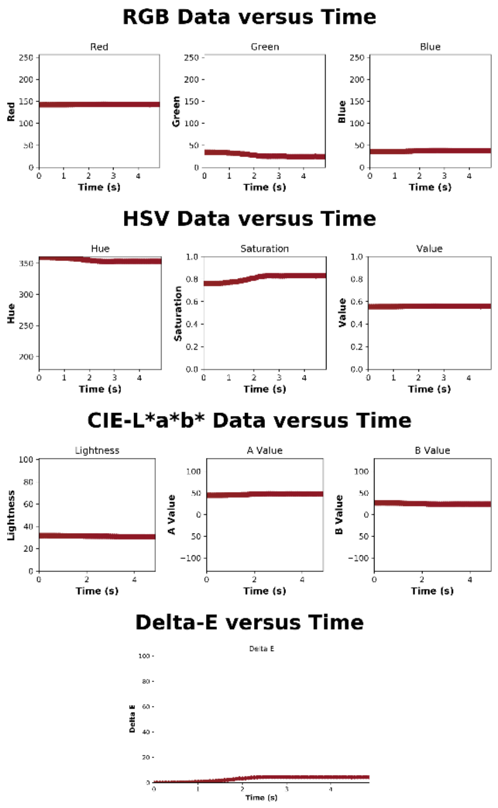

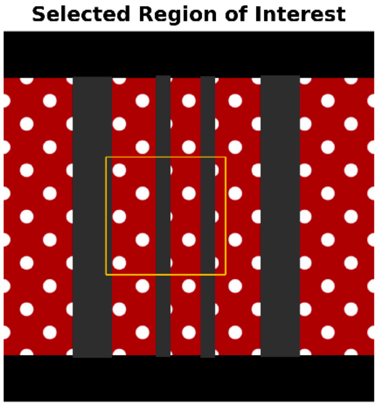

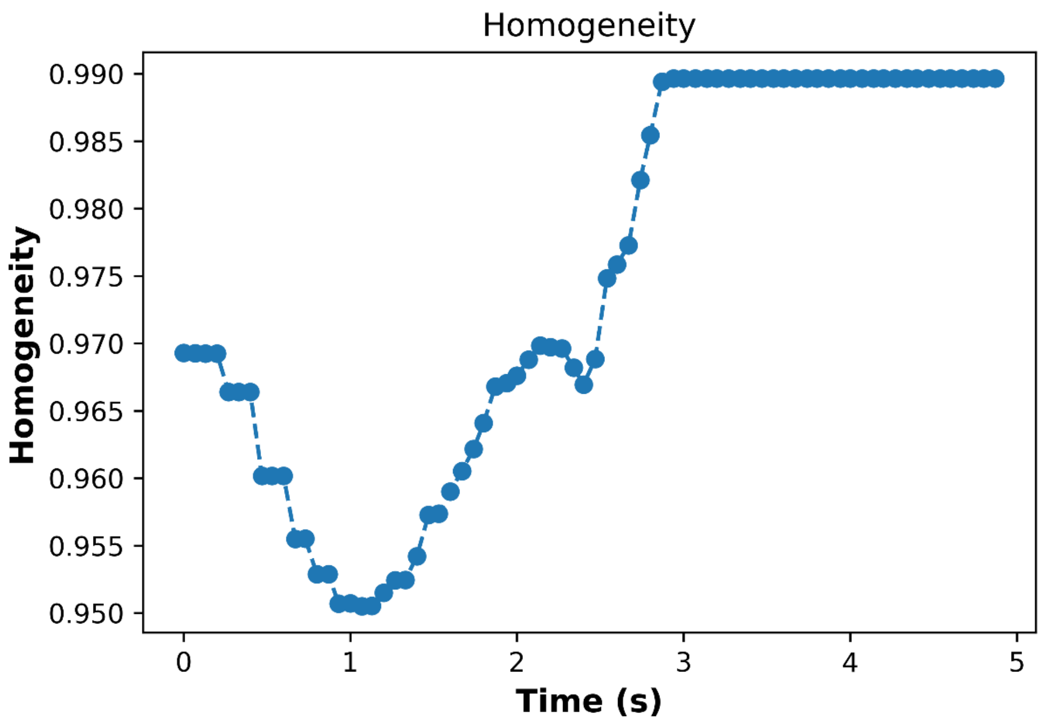
Homogeneity
0.990
0.985
0.980
0.975
0.970
0.965
0.960
0.955
0.950
Homogeneity
0
1
2
3
4
5
Time (s)

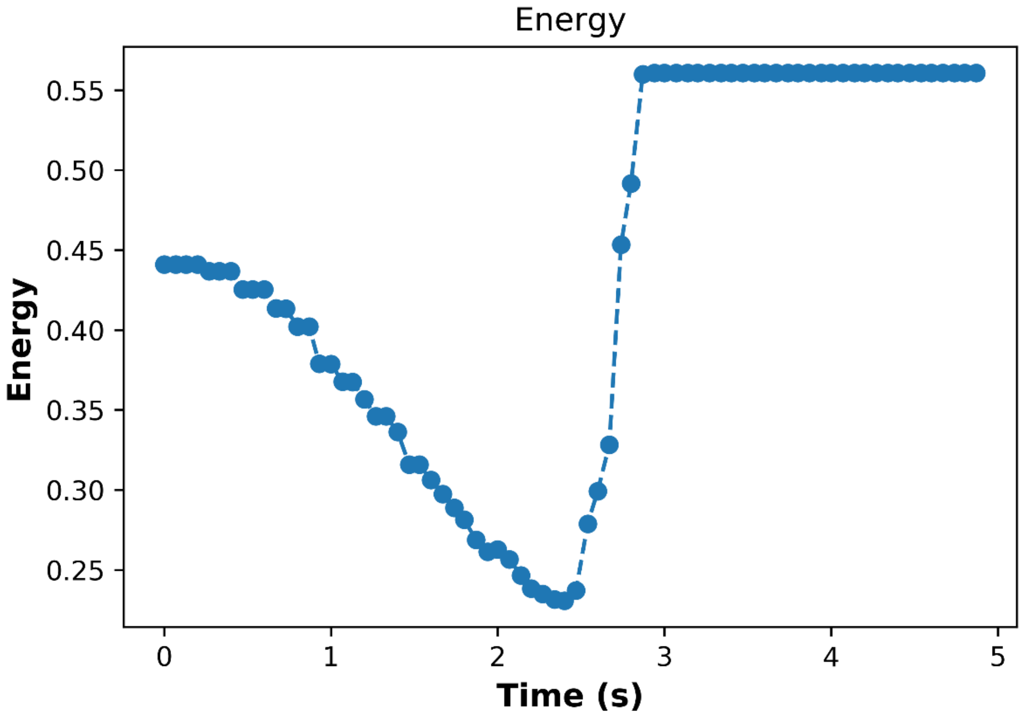
Energy
0.55
0.50
0.45
0.40
0.35
0.30
0.25
Energy
0
1
2
3
4
5
Time (s)

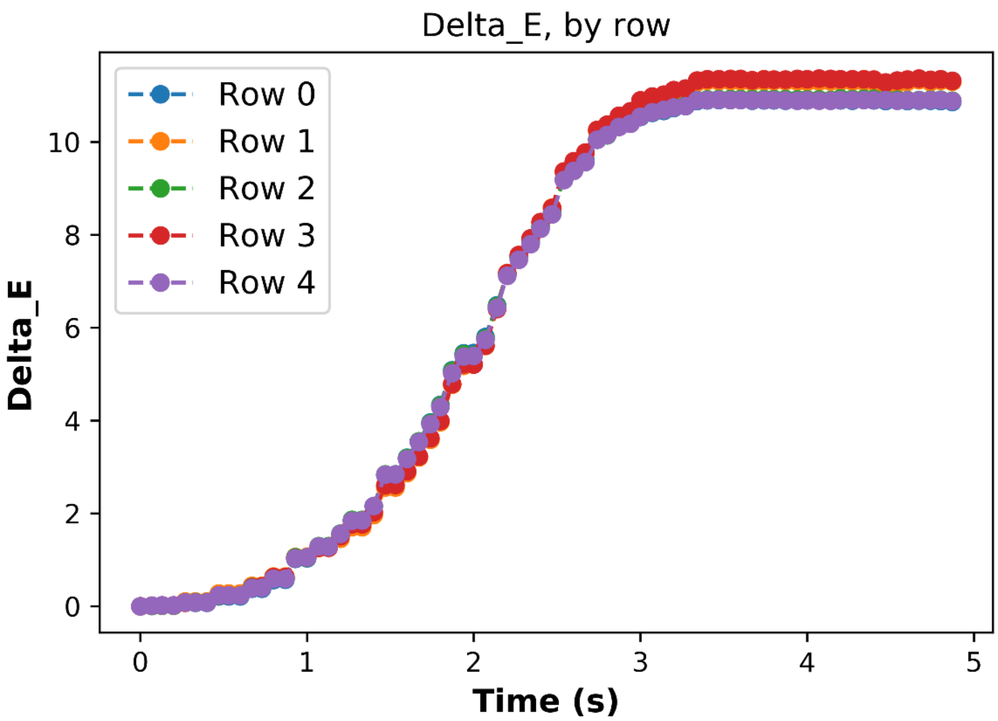
Delta_E, by row
Row 0
Row 1
Row 2
Row 3
Row 4
Delta_E
Time (s)
0
2
4
6
8
10
0
1
2
3
4
5

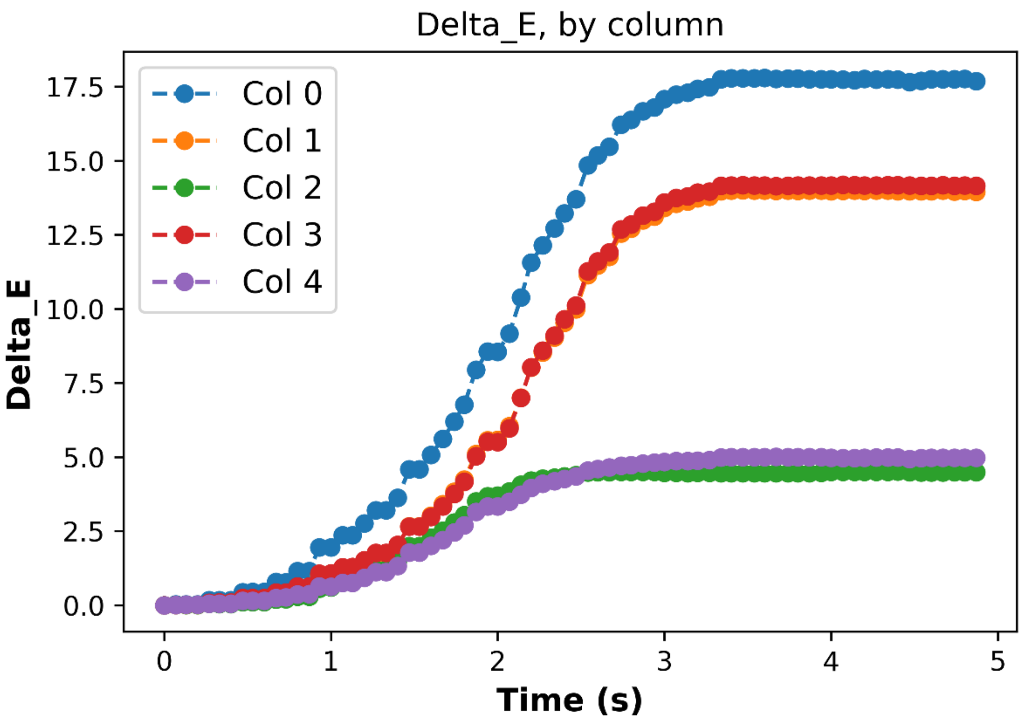
Delta_E, by column
Col 0
Col 1
Col 2
Col 3
Col 4
Delta_E
Time (s)
0.0
2.5
5.0
7.5
10.0
12.5
15.0
17.5
0
1
2
3
4
5

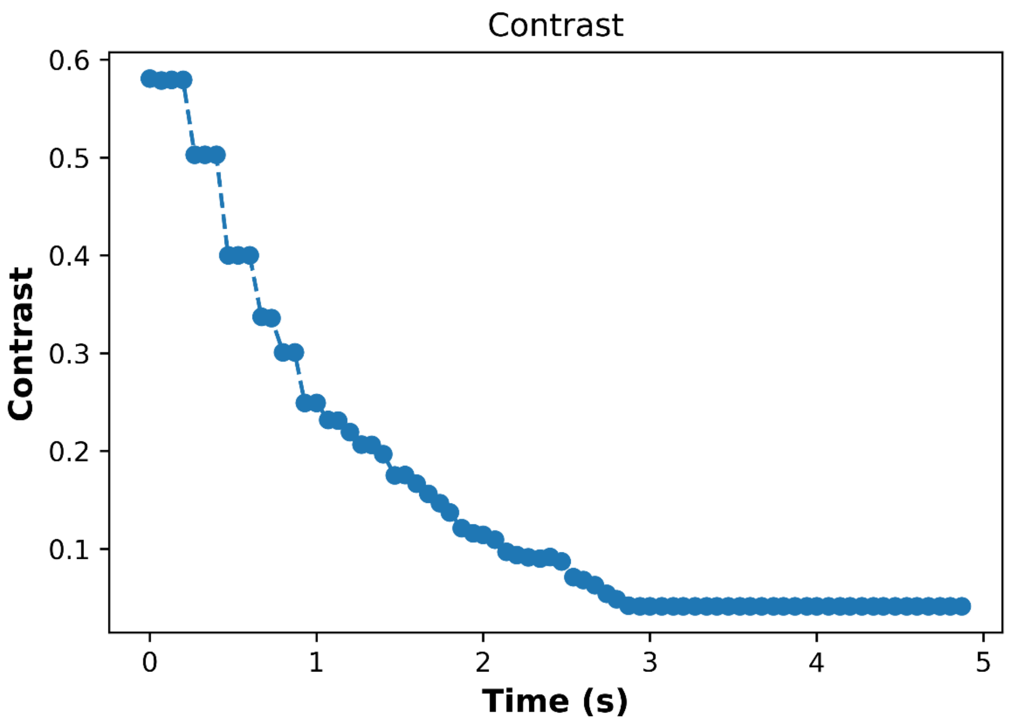
Contrast
0.6
0.5
0.4
0.3
0.2
0.1
Contrast
0
1
2
3
4
5
Time (s)

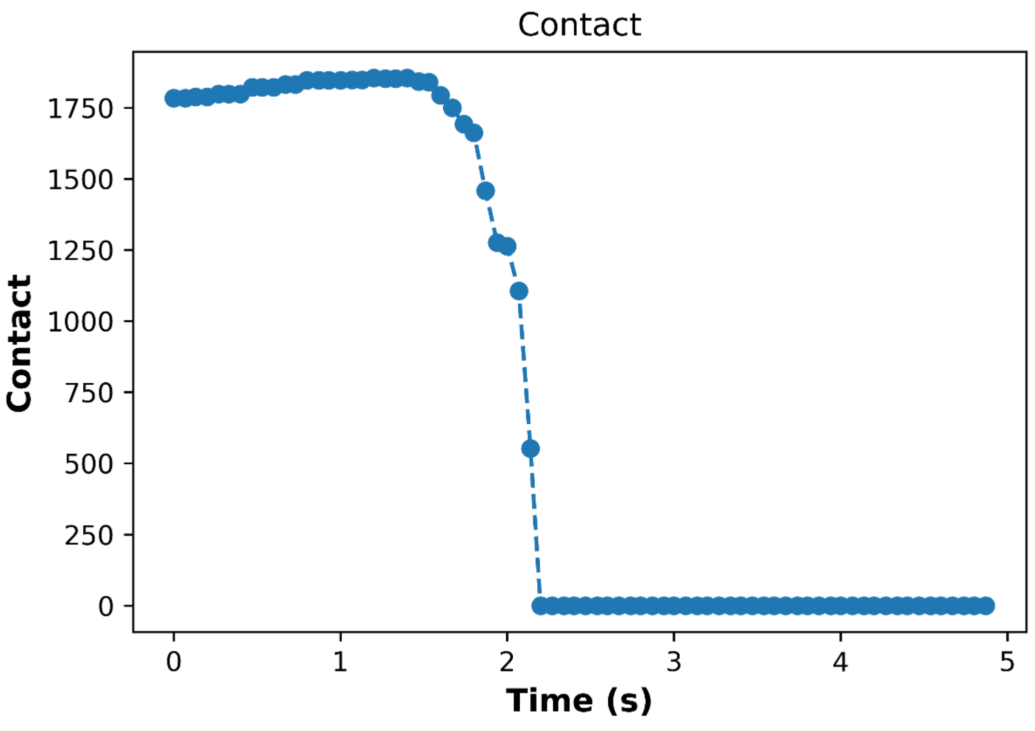
Contact
1750
1500
1250
1000
750
500
250
0
Contact
0
1
2
3
4
5
Time (s)

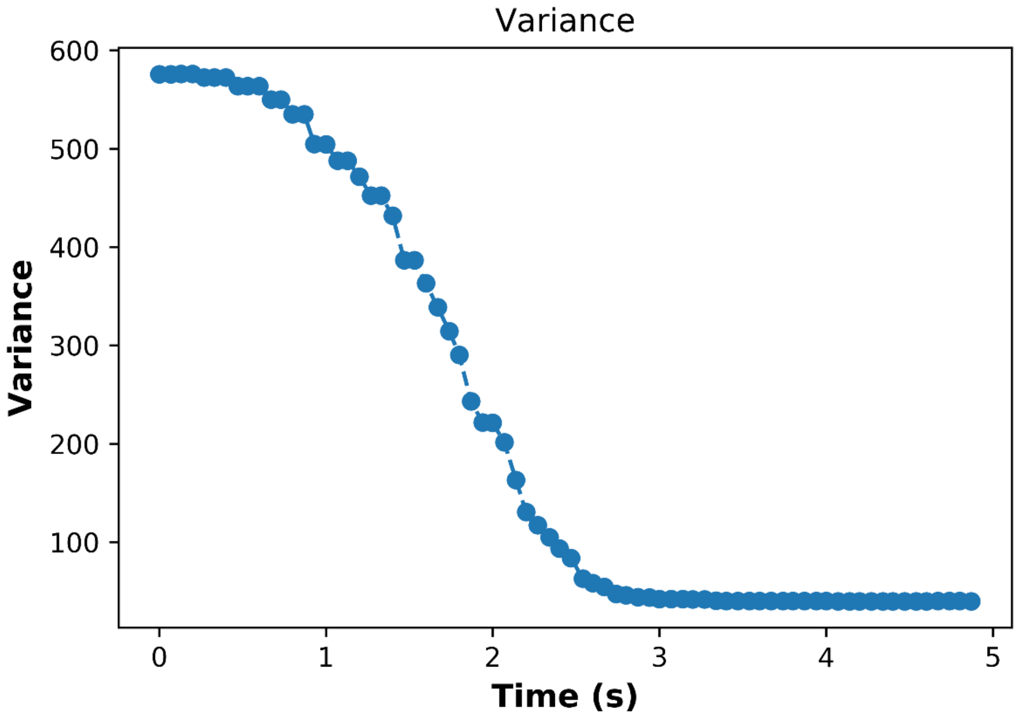
Variance
600
500
400
300
200
100
Variance
0
1
2
3
4
5
Time (s)

Data output for polka dot with white obstructions representing glass glare

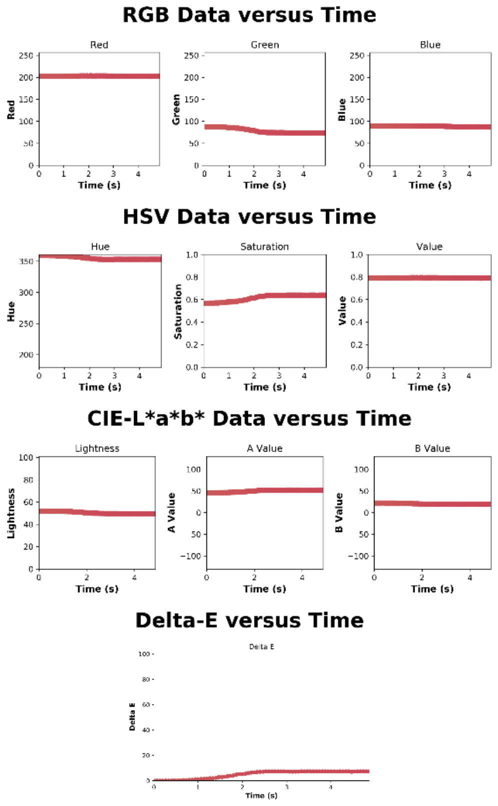

Kineticolor

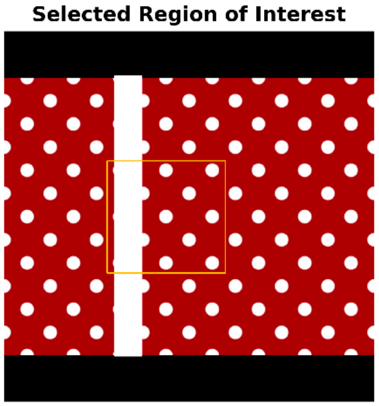

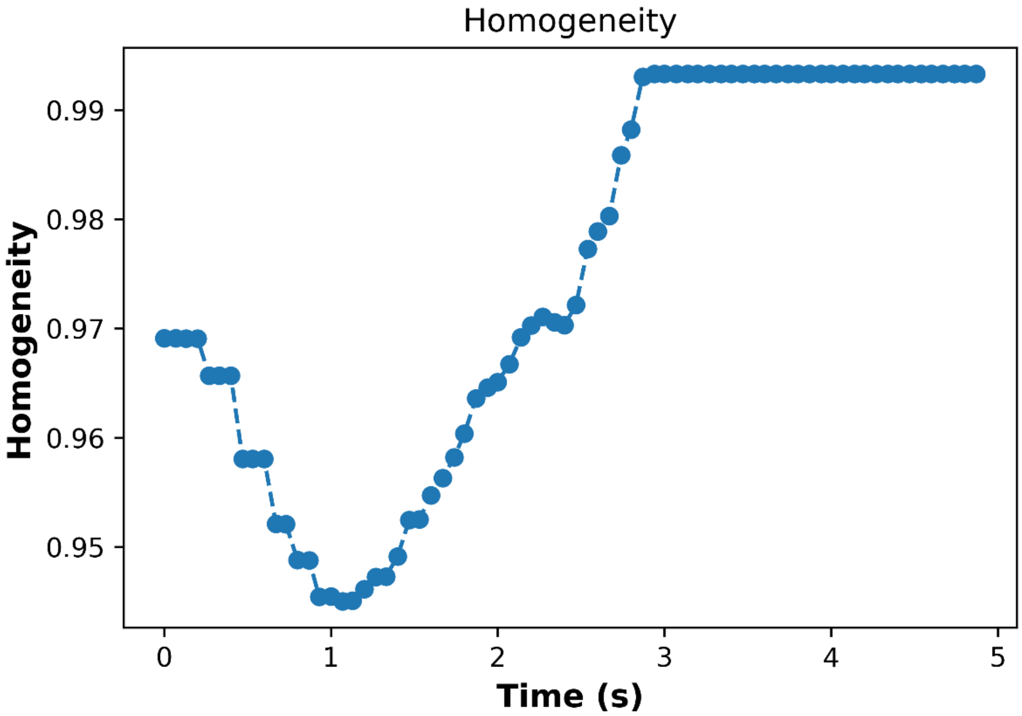

Homogeneity
Homogeneity
0.99
0.98
0.97
0.96
0.95
0
1
2
3
4
5
Time (s)

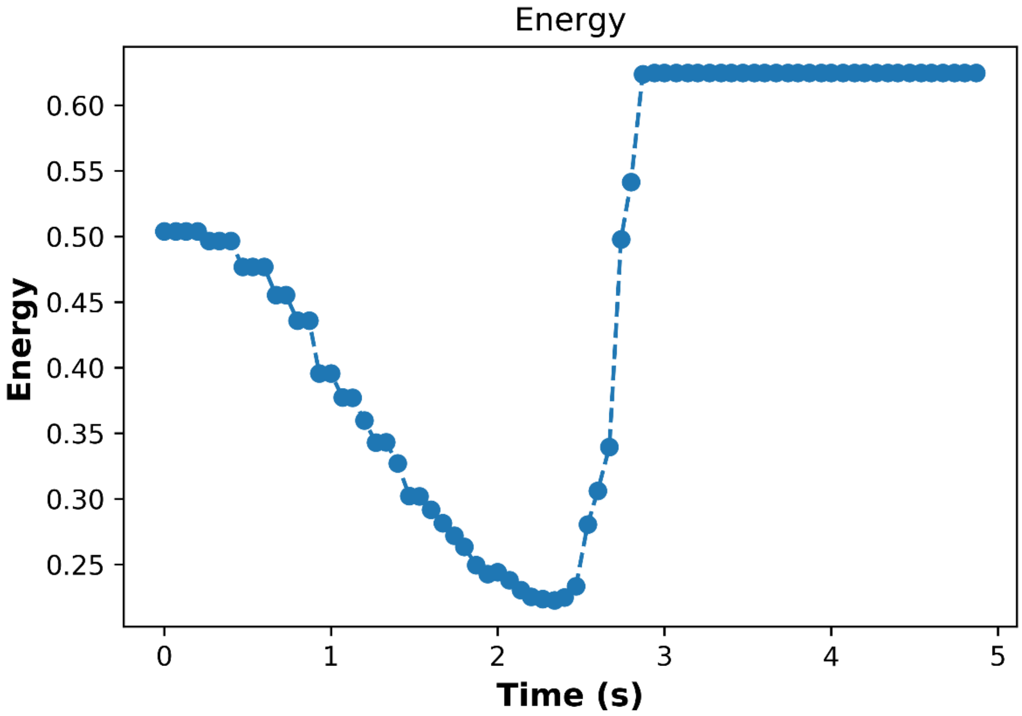

Energy
Energy
0.60
0.55
0.50
0.45
0.40
0.35
0.30
0.25
0
1
2
3
4
5
Time (s)

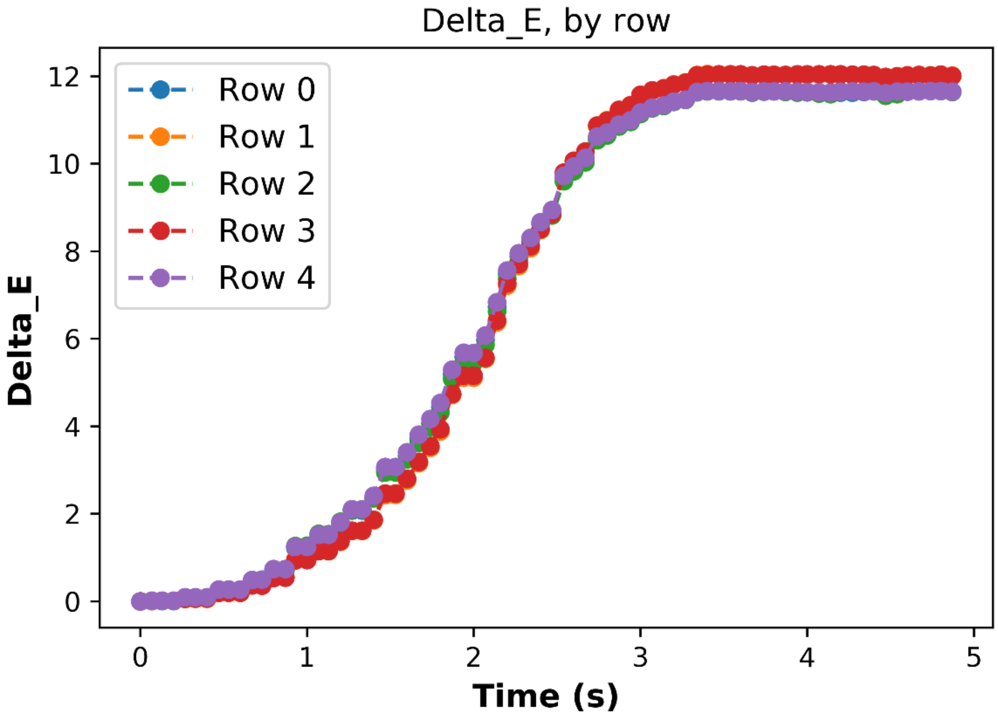
Delta_E, by row
Row 0
Row 1
Row 2
Row 3
Row 4
Delta_E
Time (s)
0
2
4
6
8
10
12
0
1
2
3
4
5

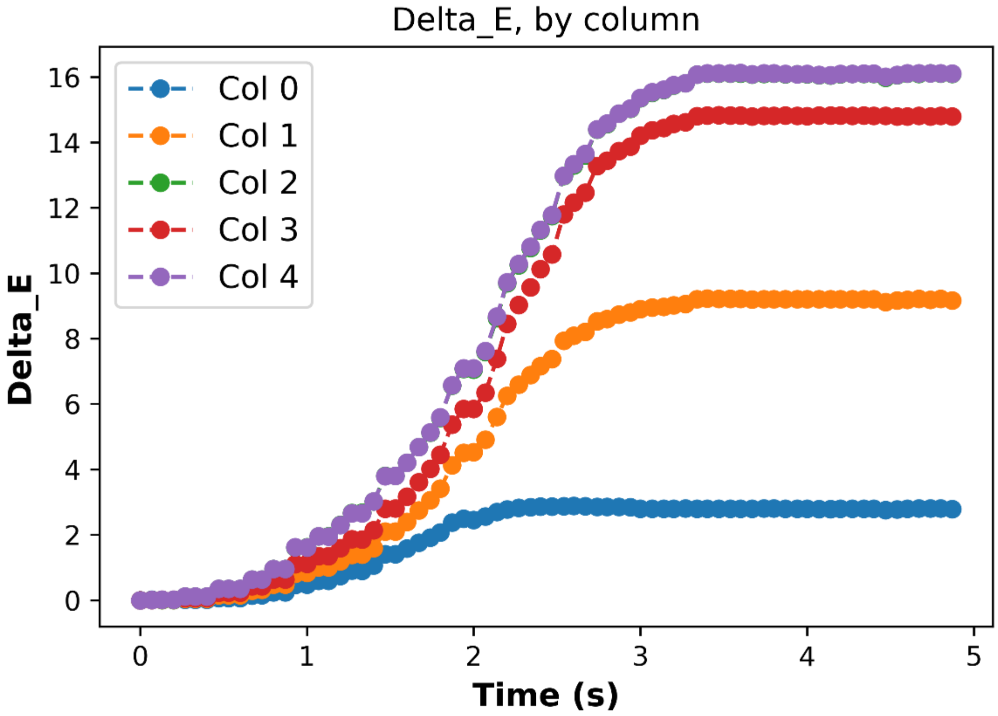
Delta_E, by column
Col 0
Col 1
Col 2
Col 3
Col 4
Delta_E
Time (s)
0
2
4
6
8
10
12
14
16
0
1
2
3
4
5

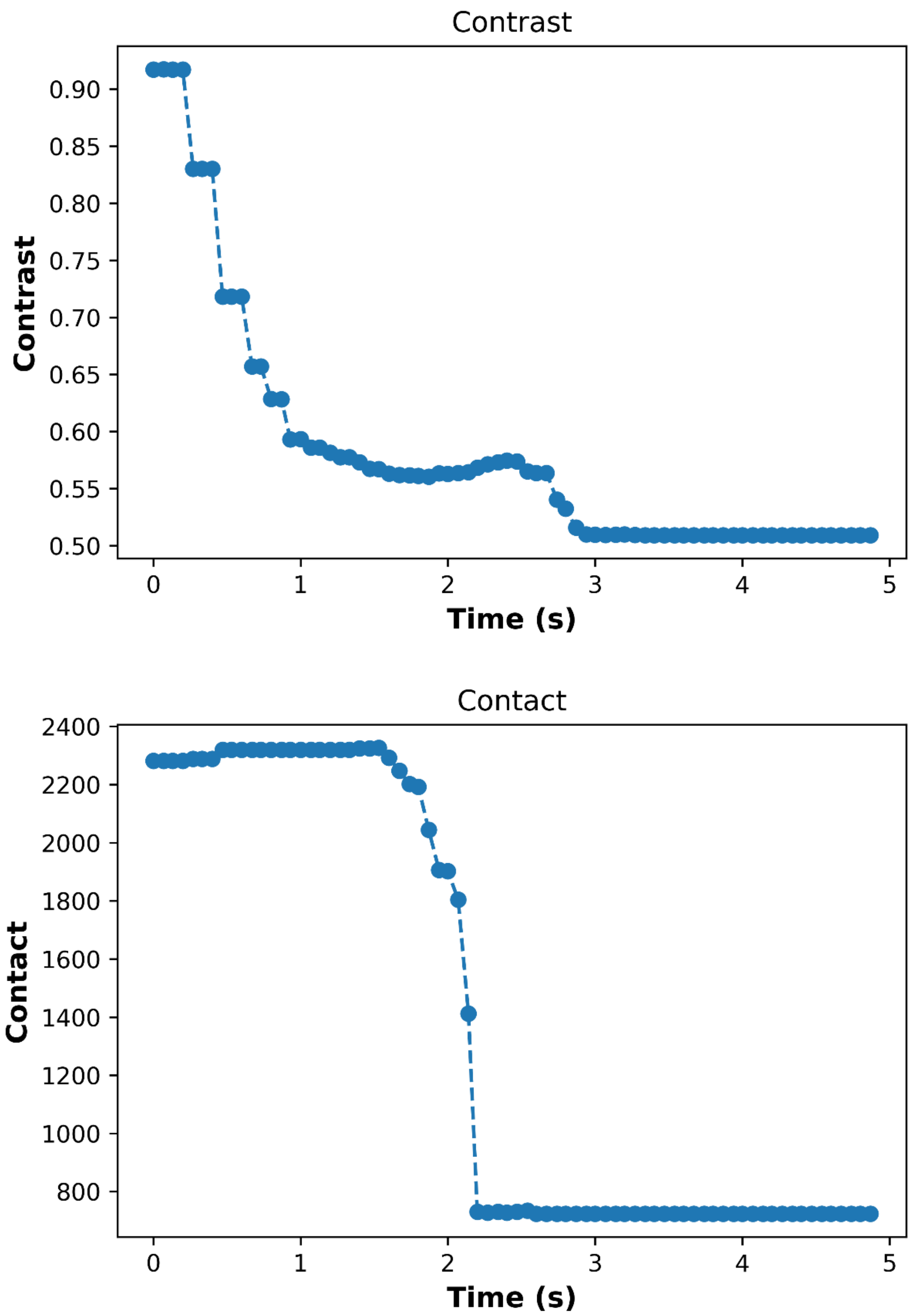
Contrast
0.90
0.85
0.80
0.75
0.70
0.65
0.60
0.55
0.50
Contrast
0
1
2
3
4
5
Time (s)
Contact
2400
2200
2000
1800
1600
1400
1200
1000
800
Contact
0
1
2
3
4
5
Time (s)

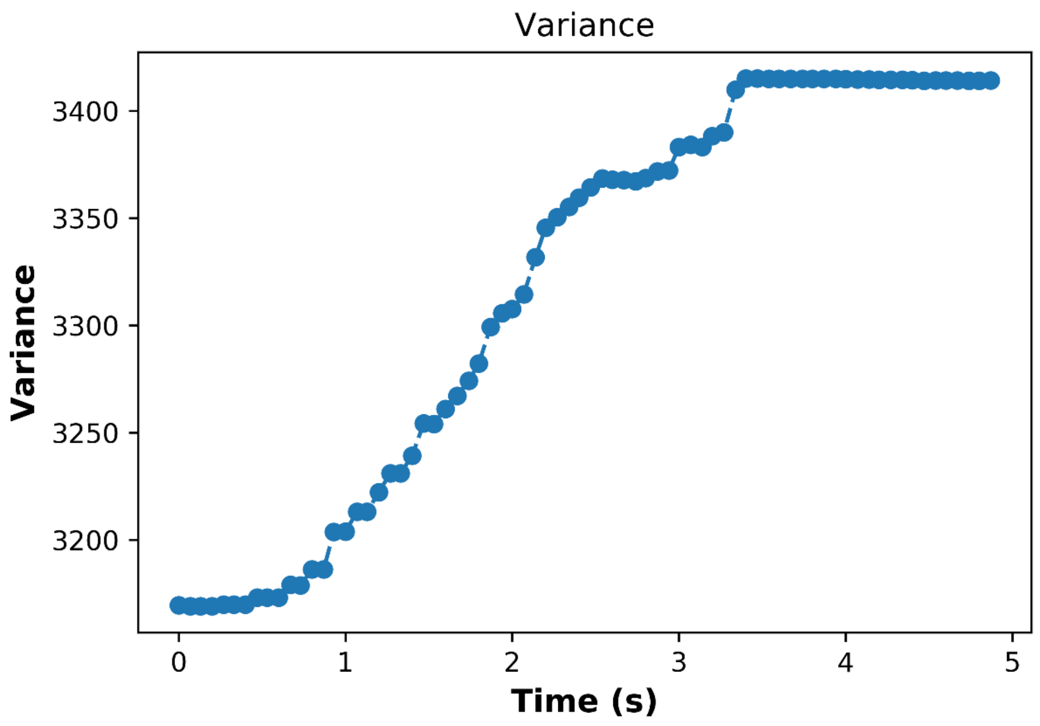

Comparison of different polka dot spacings to build ASM and Homogeneity Intuition

Regularly-spaced spots:

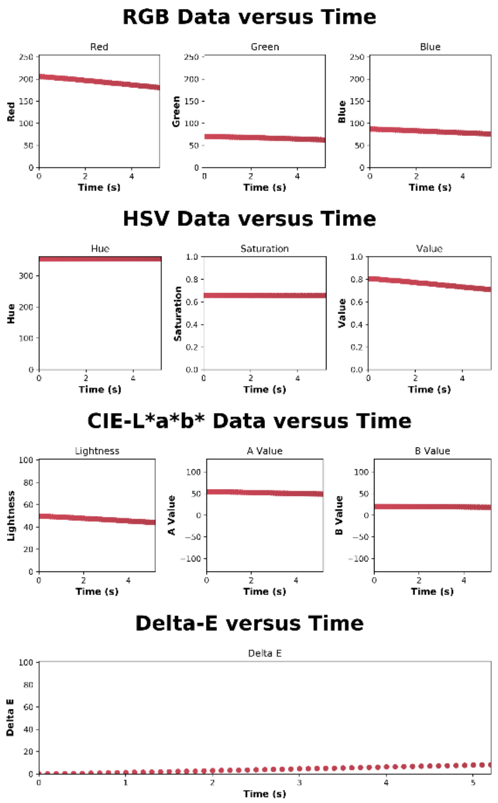

Kineticolor

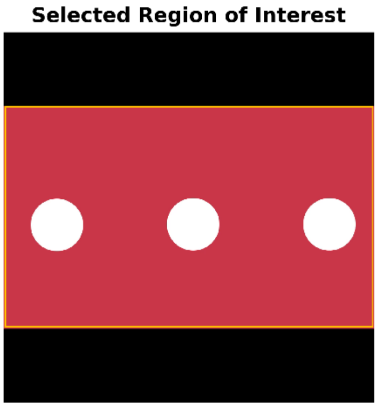

Irregularly spaced spots:

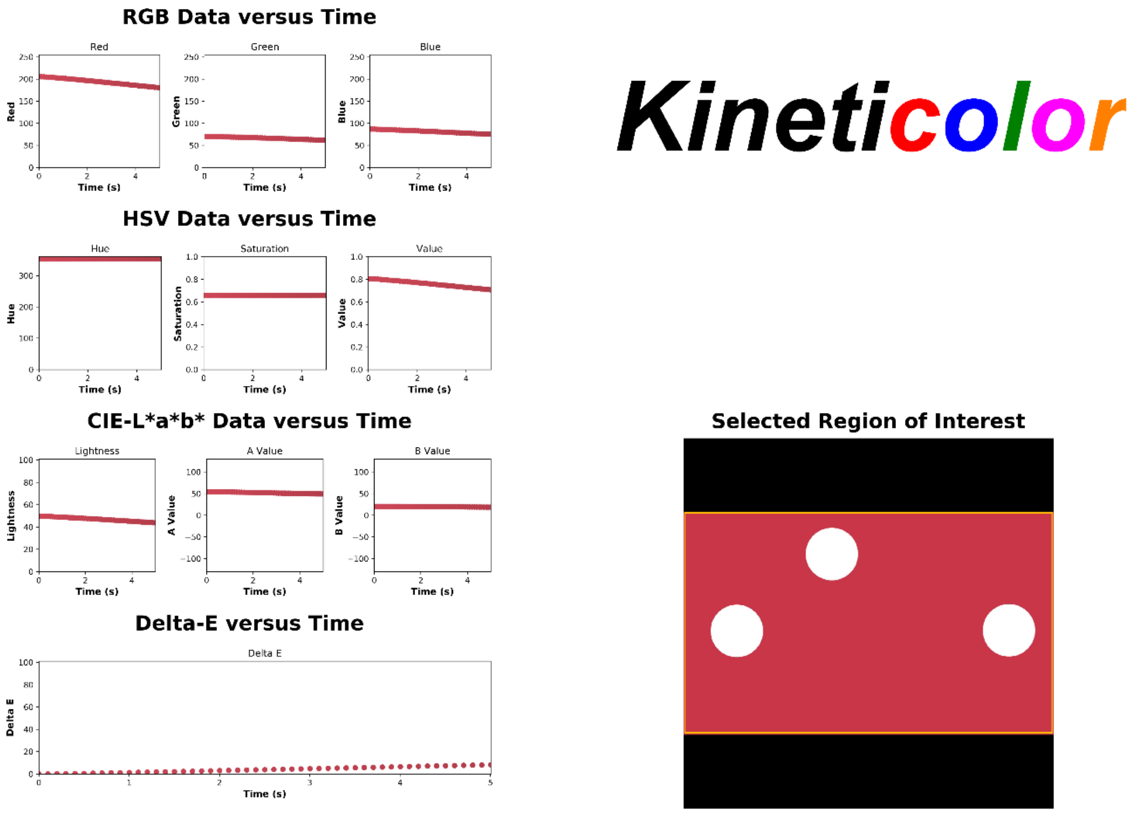
RGB Data versus Time
Red
Green
Blue
Time (s)
HSV Data versus Time
Hue
Saturation
Value
CIE-L*a*b* Data versus Time
Lightness
A Value
B Value
Delta-E versus Time
Delta E
Kineticolor
Selected Region of Interest

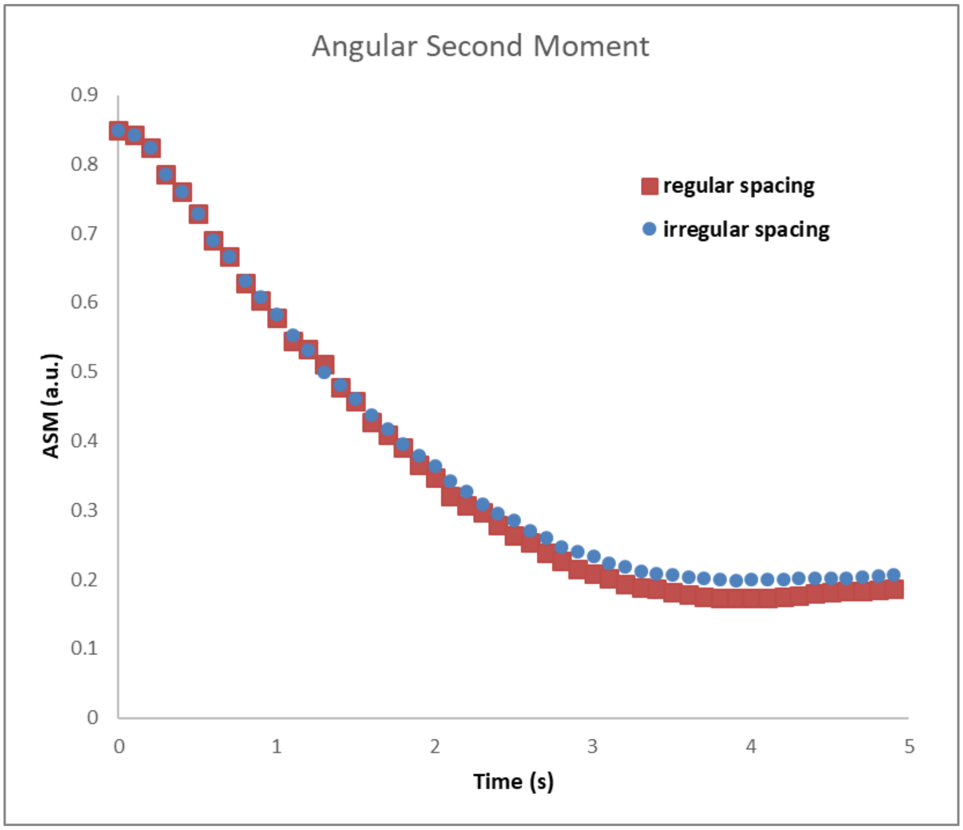
Angular Second Moment
ASM (a.u.)
Time (s)
regular spacing
irregular spacing

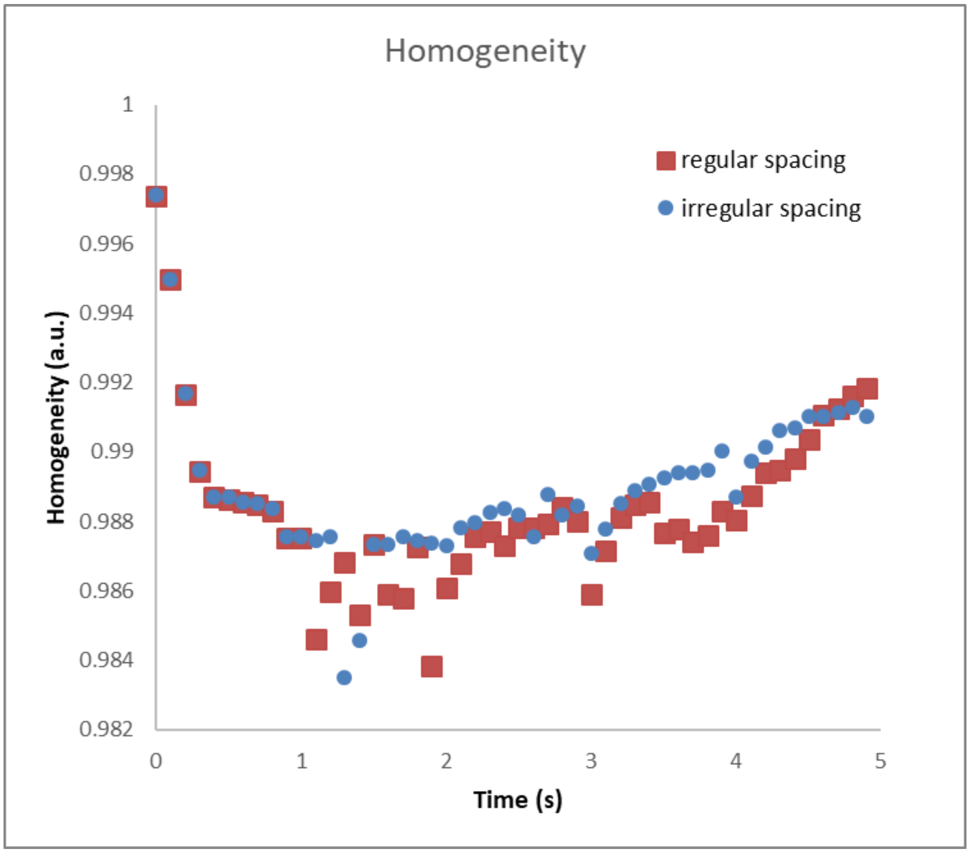

For approximately the same ROI size, the ASM and Homogeneity values overlap for both video types.

These simulations more clearly demonstrate that the spacing (the level of symmetry or order) doesn't matter directly. It is the overall image similarity that matters when affecting ASM and Homogeneity.

4. Qualitative Visualization of Mixing Phenomena in Plant Mimic Vessels

Scheme 8 in the manuscript provides exemplar comparative analyses of saturated $NaHCO_3$ mixtures settling after mixing was stopped. Table 1 give the full comparative suite of approximate settling times.

The following data outputs represent the raw data generated for each mixing condition described in manuscript Table 1.

All analyses below capture mixing before and after stirring was stopped.

Table 1, Entry 1: 60 RPM, Paddle, No baffles, No probe

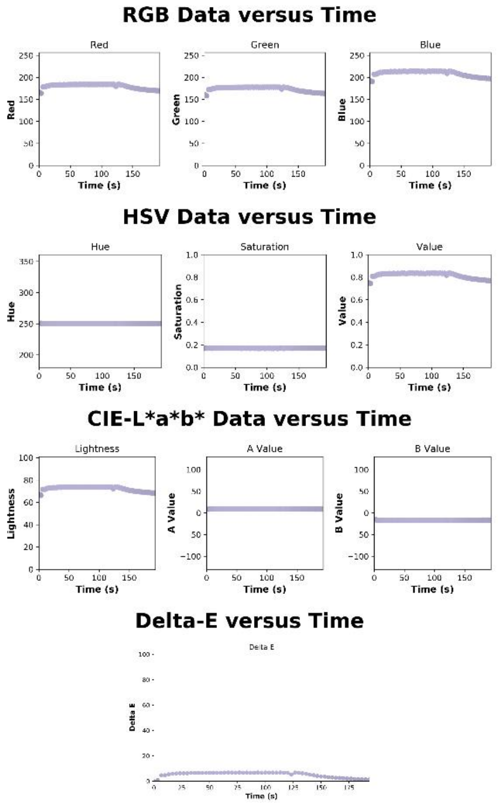

Selected Region of Interest

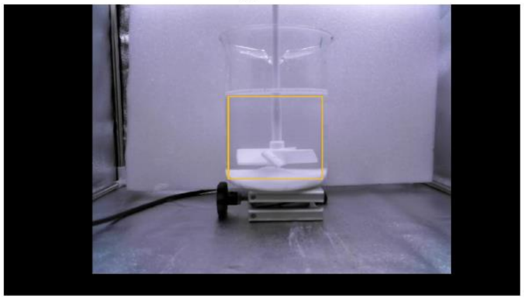

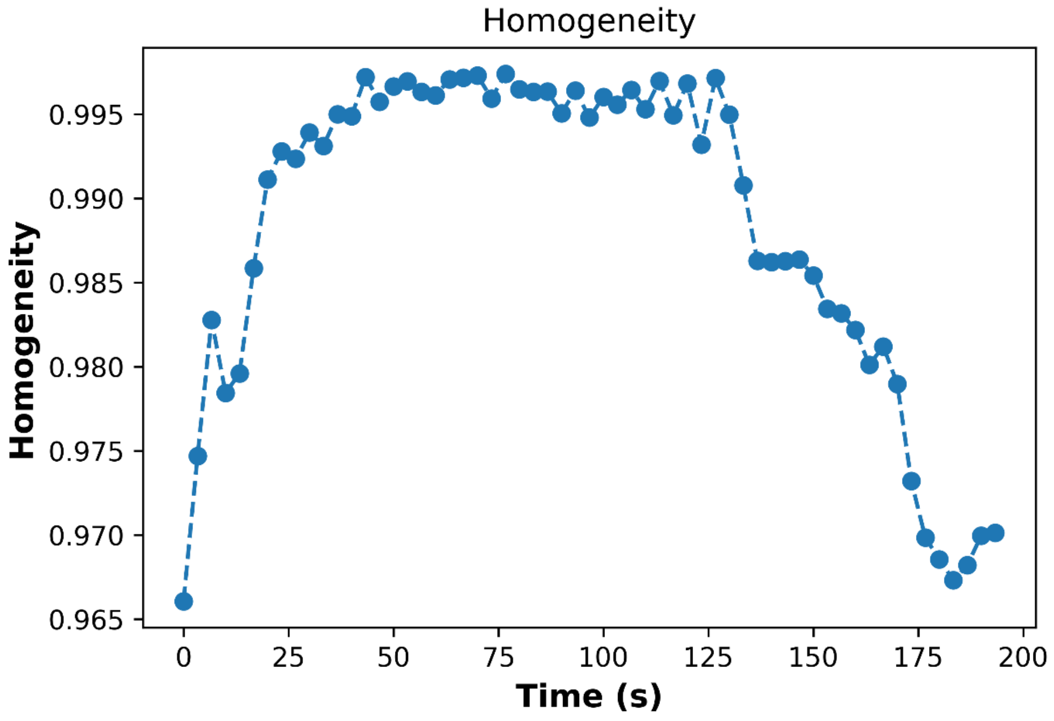
Homogeneity
Homogeneity
0.995
0.990
0.985
0.980
0.975
0.970
0.965
0
25
50
75
100
125
150
175
200
Time (s)

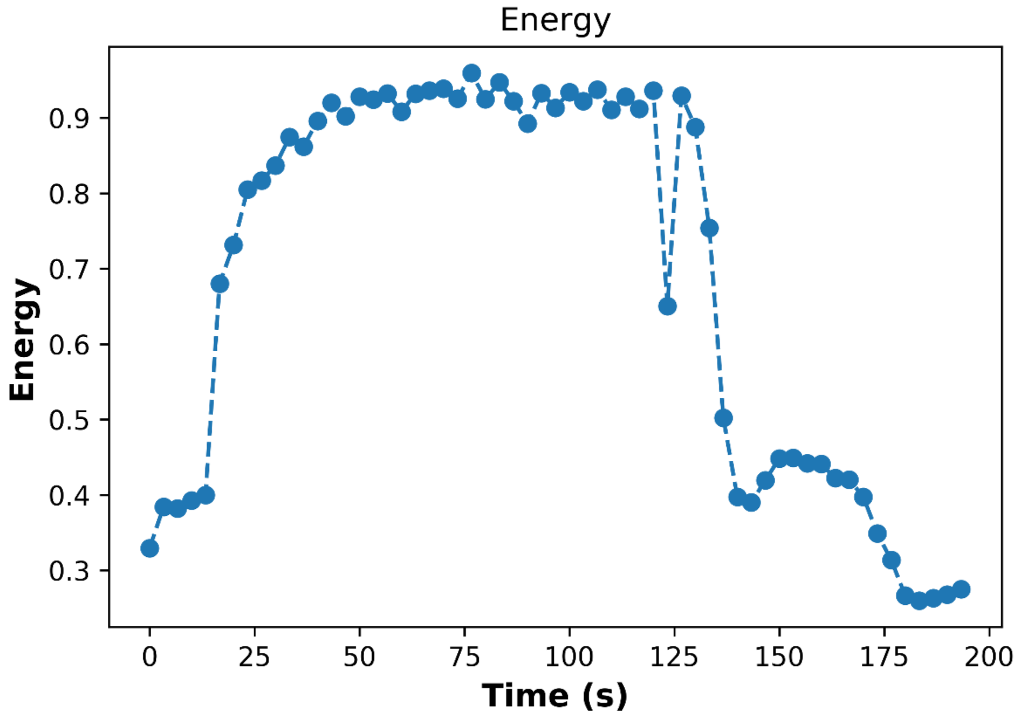
Energy
Energy
0.9
0.8
0.7
0.6
0.5
0.4
0.3
0
25
50
75
100
125
150
175
200
Time (s)

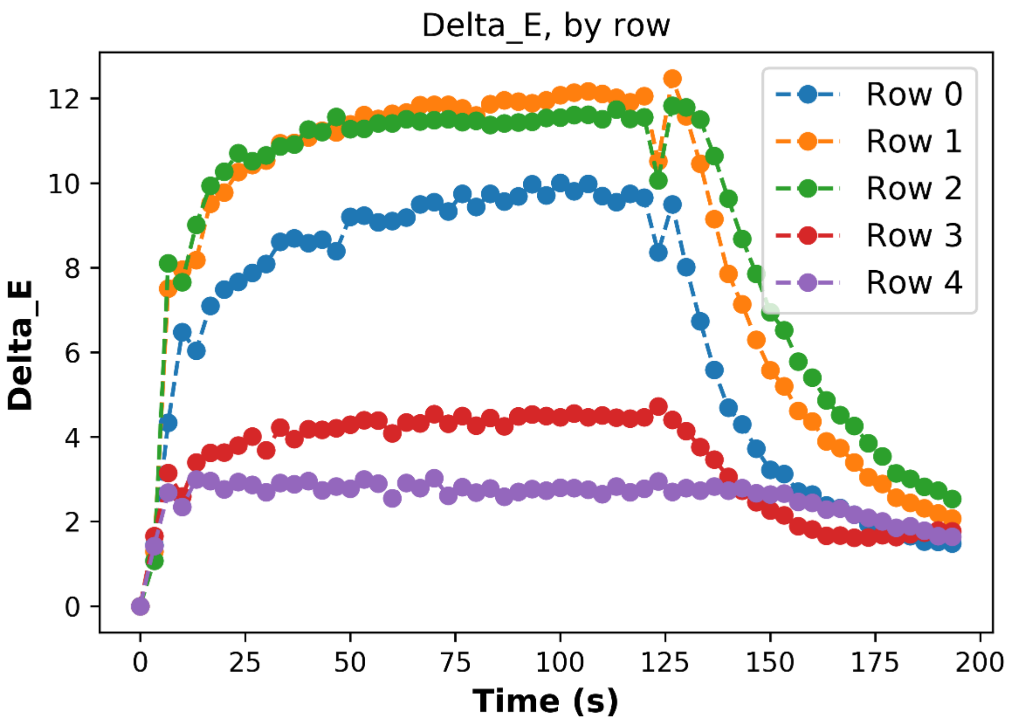
Delta_E, by row
Delta_E
Time (s)
Row 0
Row 1
Row 2
Row 3
Row 4
0
2
4
6
8
10
12
0
25
50
75
100
125
150
175
200

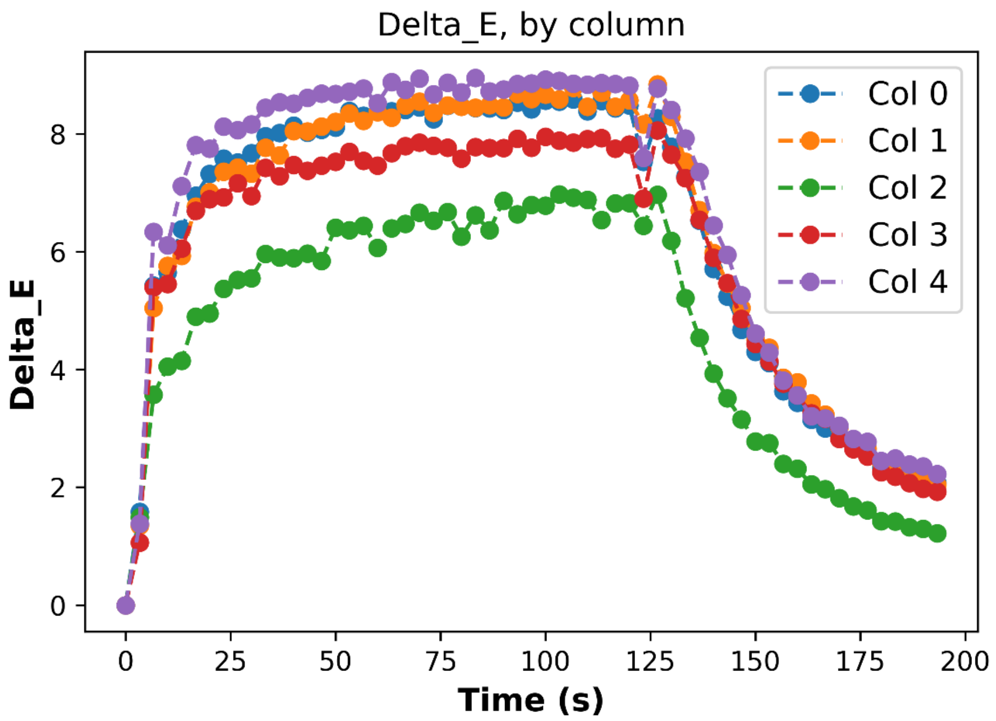
Delta_E, by column
Delta_E
Time (s)
Col 0
Col 1
Col 2
Col 3
Col 4
0
2
4
6
8
0
25
50
75
100
125
150
175
200

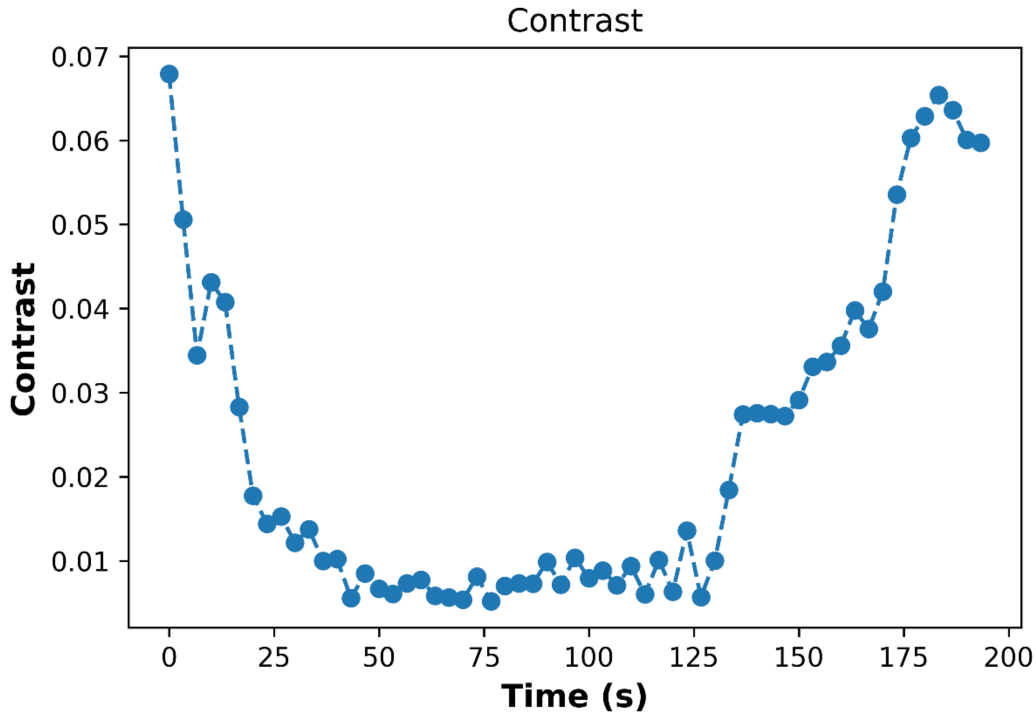
Contrast
0.07
0.06
0.05
0.04
0.03
0.02
0.01
Contrast
0
25
50
75
100
125
150
175
200
Time (s)

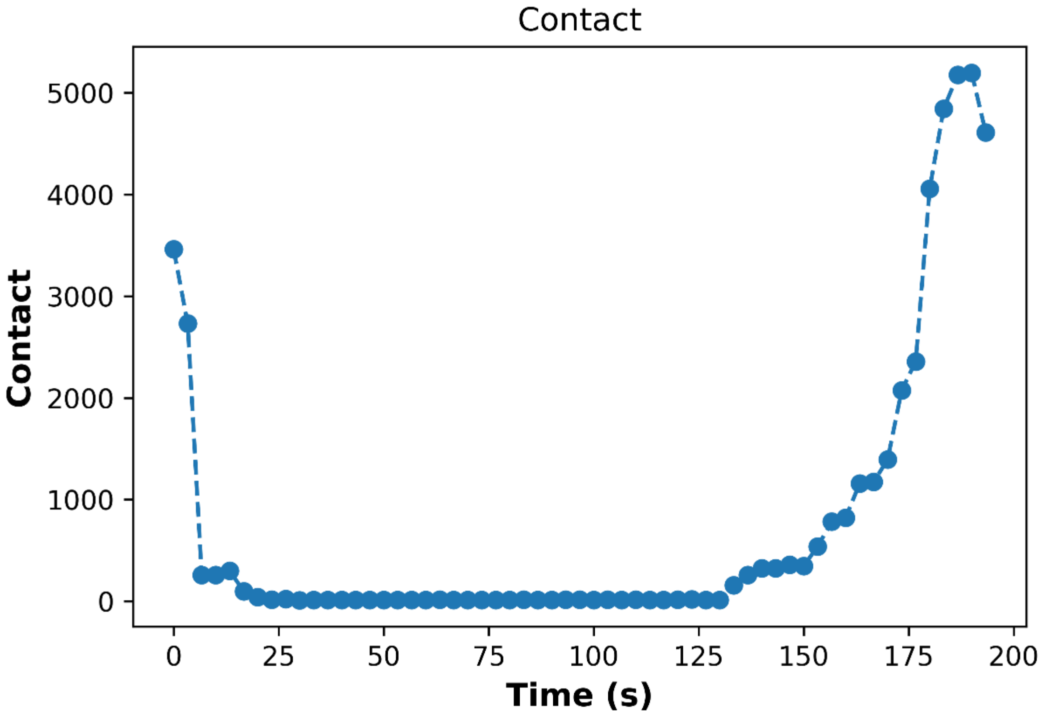
Contact
5000
4000
3000
2000
1000
0
Contact
0
25
50
75
100
125
150
175
200
Time (s)

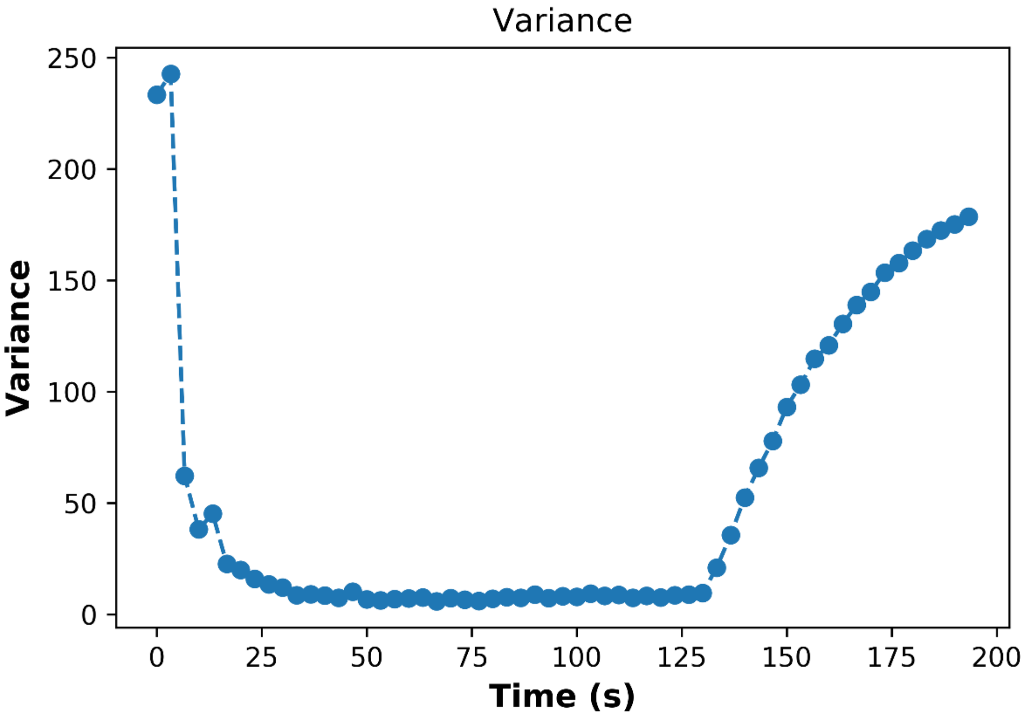

Variance
Variance
250
200
150
100
50
0
0
25
50
75
100
125
150
175
200
Time (s)

Table 1, Entry 1: 60 RPM, Paddle, No baffles, with probe

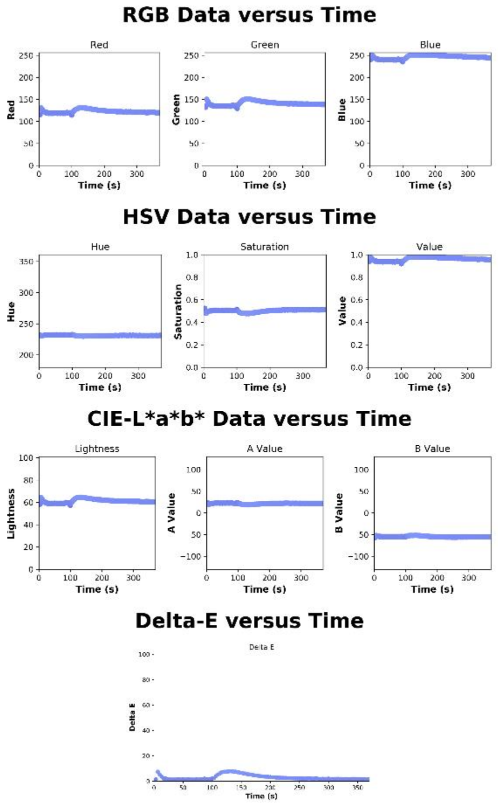

Kineticolor

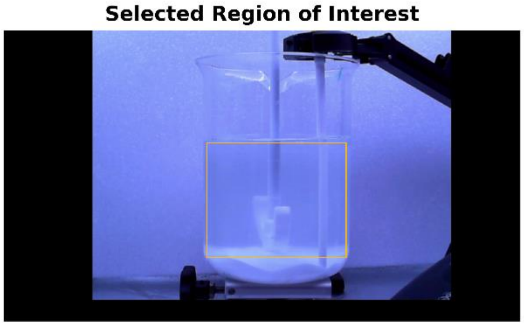

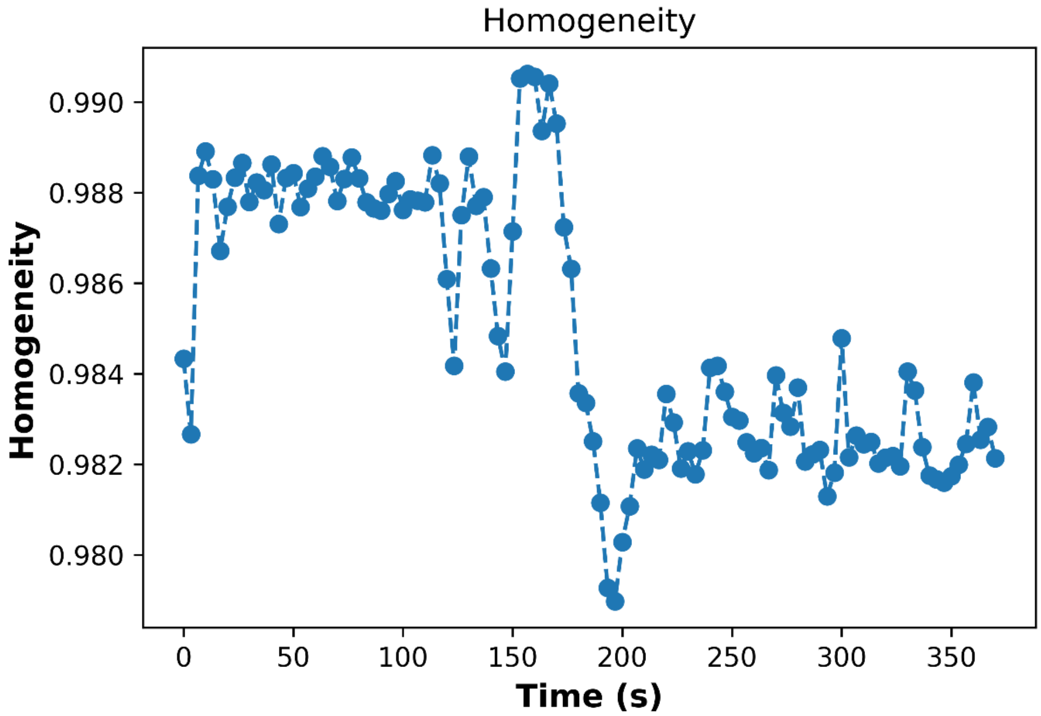
Homogeneity
0.990
0.988
0.986
0.984
0.982
0.980
Homogeneity
0
50
100
150
200
250
300
350
Time (s)

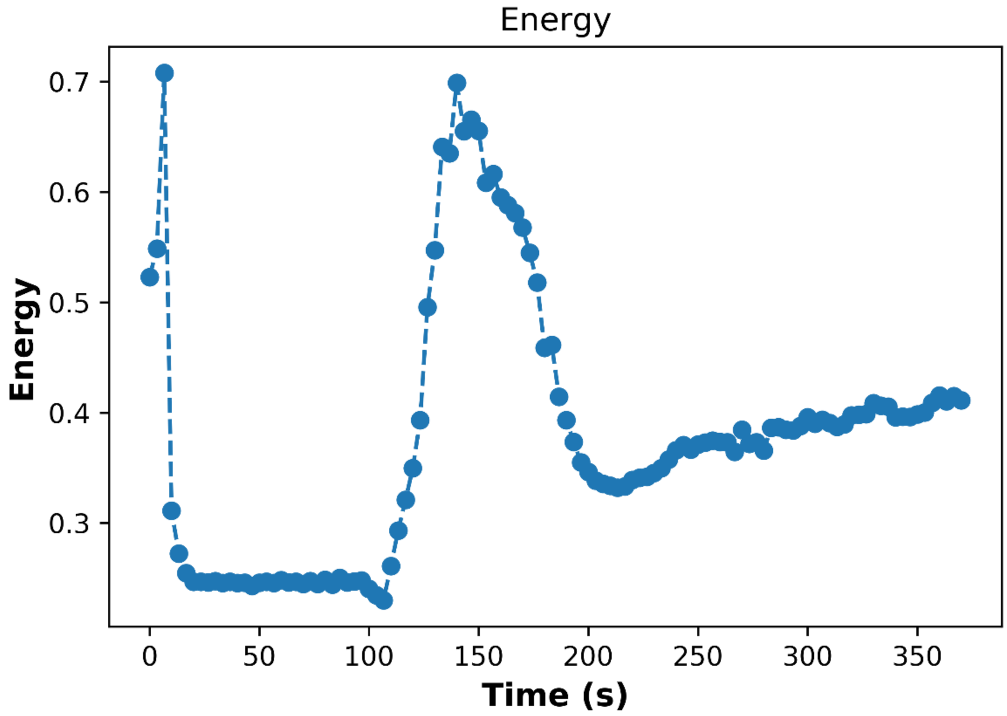
Energy
0.7
0.6
0.5
0.4
0.3
Energy
0
50
100
150
200
250
300
350
Time (s)

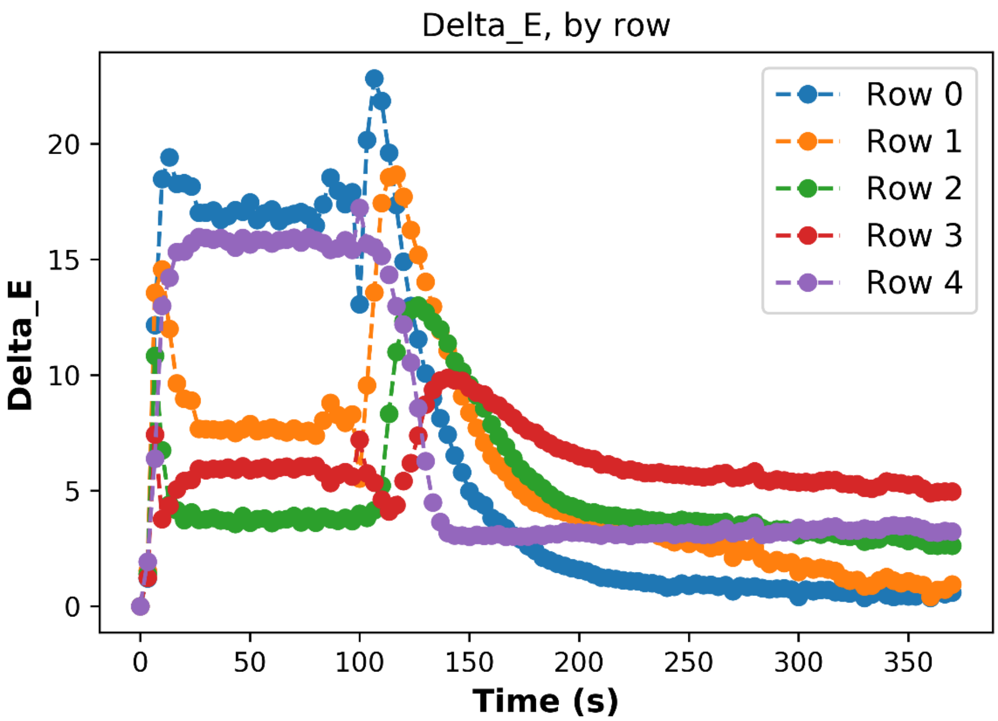

Delta_E, by row
Row 0
Row 1
Row 2
Row 3
Row 4
Delta_E
Time (s)
0
5
10
15
20
0
50
100
150
200
250
300
350

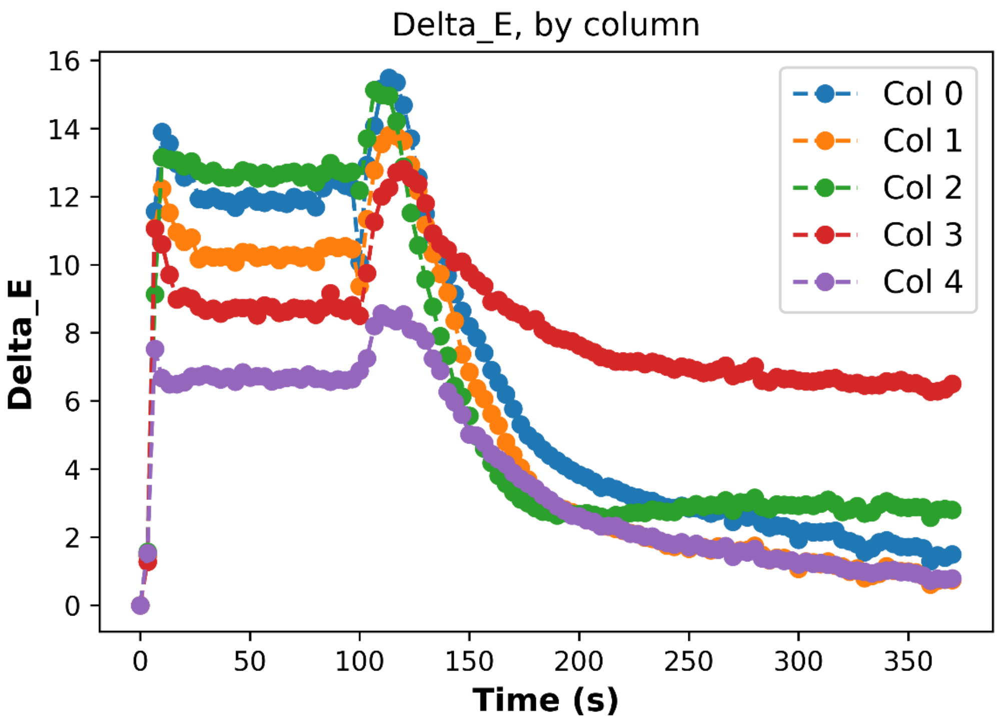

Delta_E, by column
Col 0
Col 1
Col 2
Col 3
Col 4
Delta_E
Time (s)
0
2
4
6
8
10
12
14
16
0
50
100
150
200
250
300
350

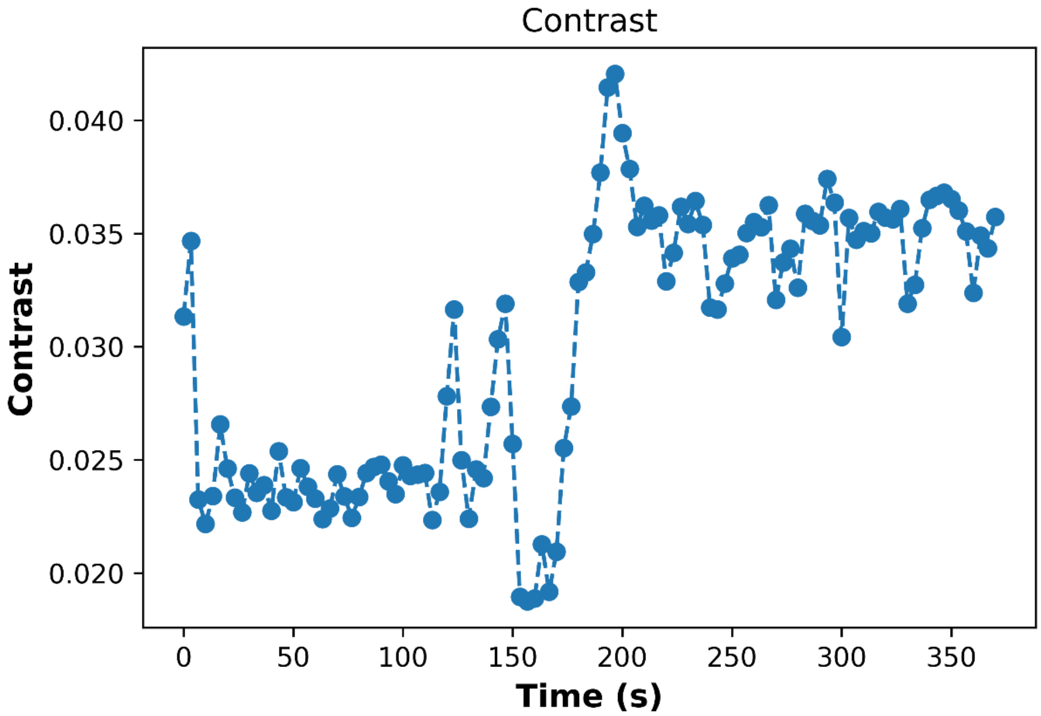
Contrast
0.040
0.035
0.030
0.025
0.020
Contrast
0
50
100
150
200
250
300
350
Time (s)

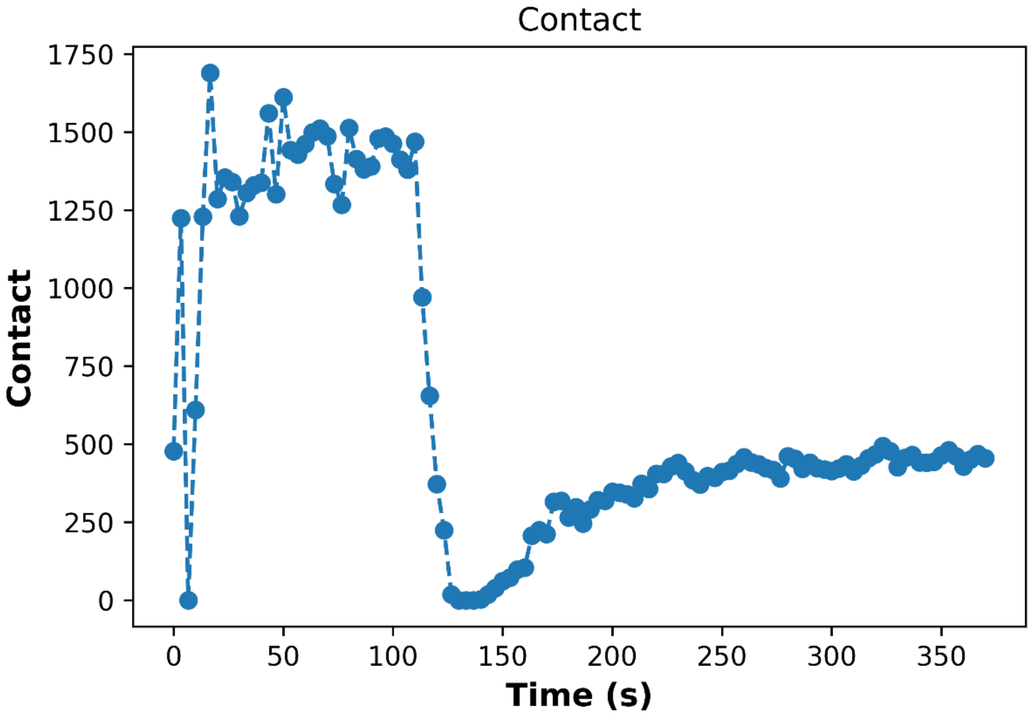
Contact
1750
1500
1250
1000
750
500
250
0
Contact
0
50
100
150
200
250
300
350
Time (s)

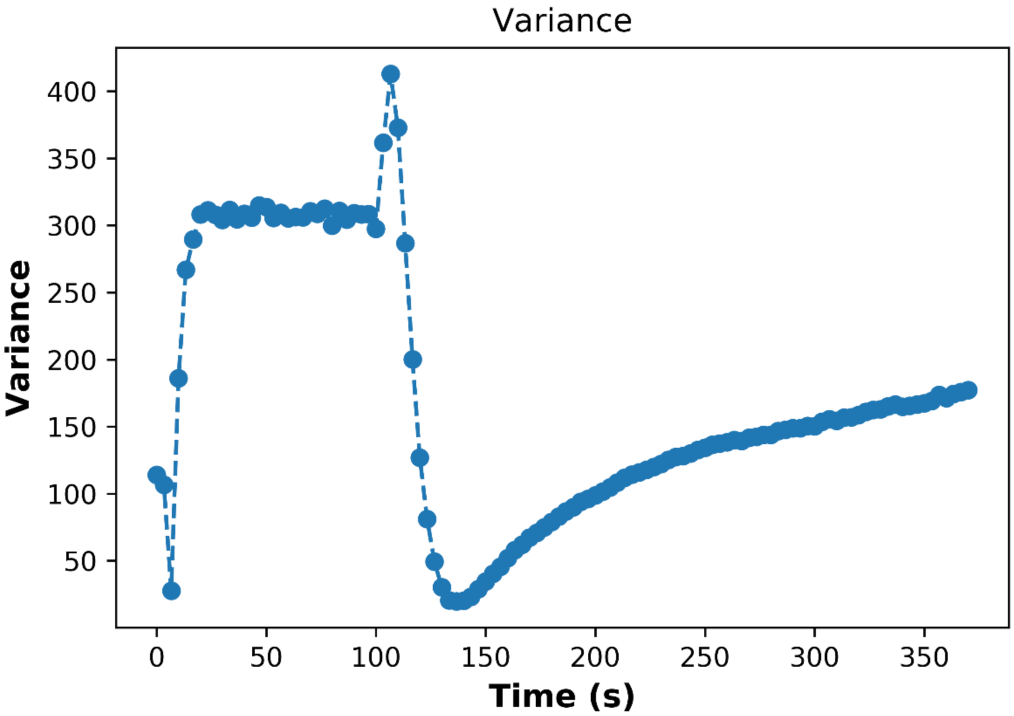

Variance
400
350
300
250
200
150
100
50
Variance
0
50
100
150
200
250
300
350
Time (s)

Table 1, Entry 2: 100 RPM, Paddle, No baffles, with probe

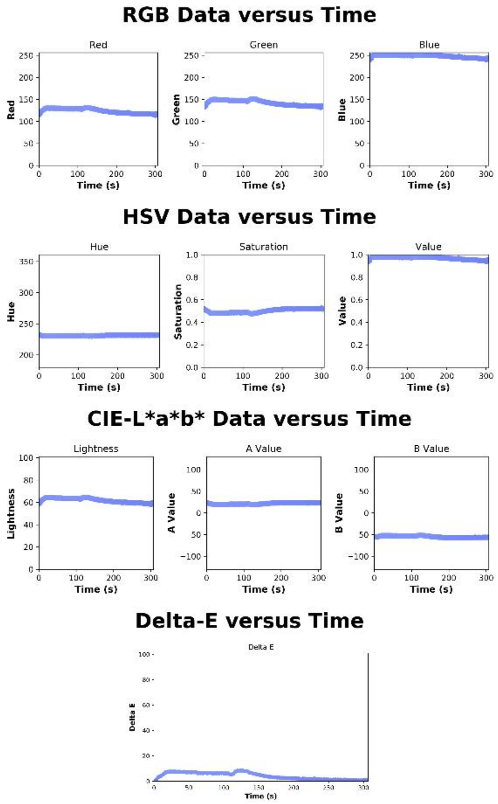

Kineticolor

Selected Region of Interest

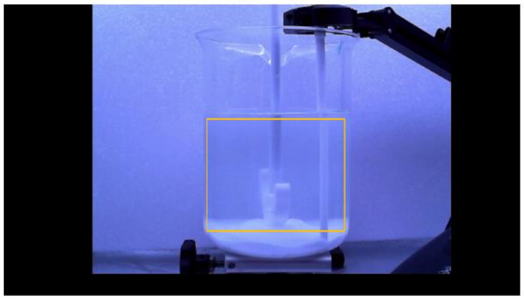

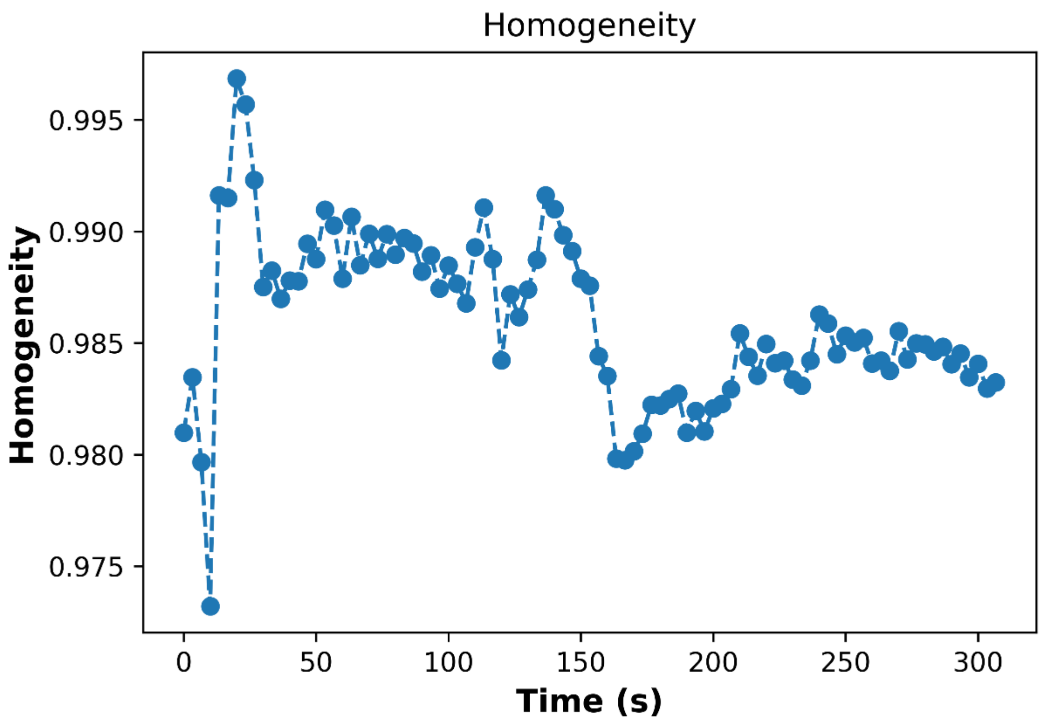
Homogeneity
Homogeneity
0.995
0.990
0.985
0.980
0.975
0
50
100
150
200
250
300
Time (s)

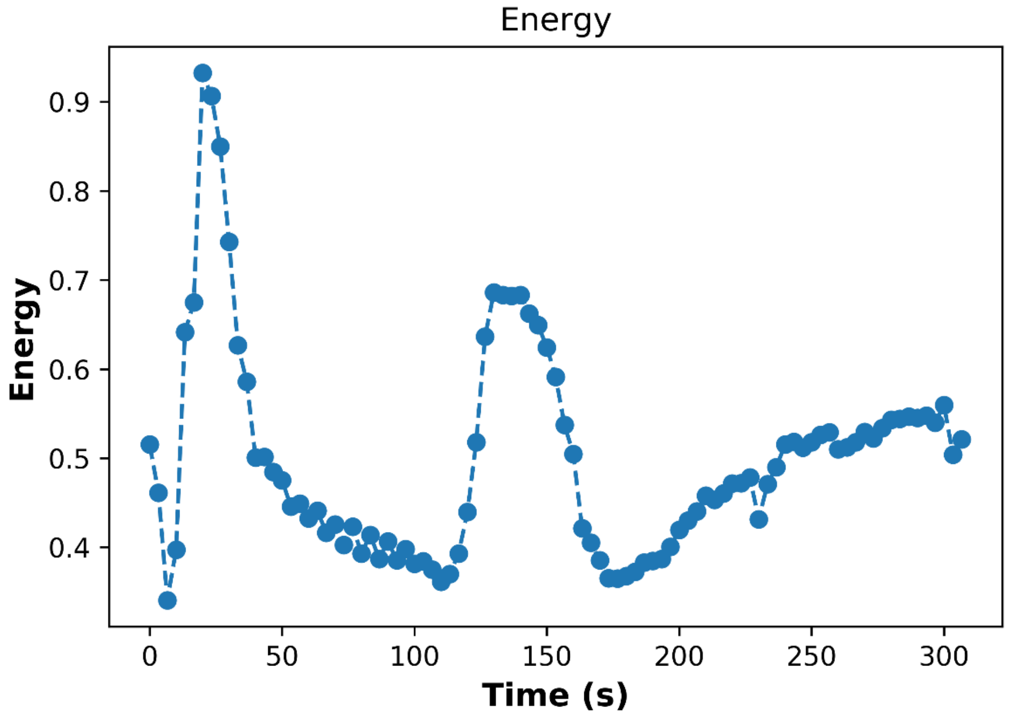
Energy
Energy
0.9
0.8
0.7
0.6
0.5
0.4
0
50
100
150
200
250
300
Time (s)

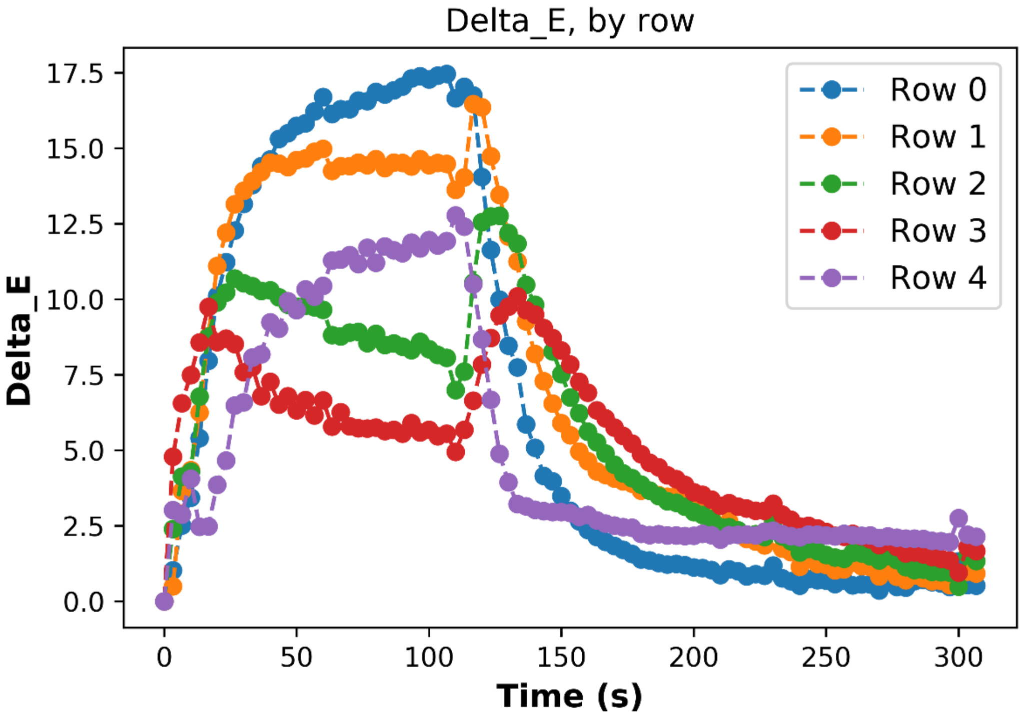
Delta_E, by row
Delta_E
Time (s)
Row 0
Row 1
Row 2
Row 3
Row 4

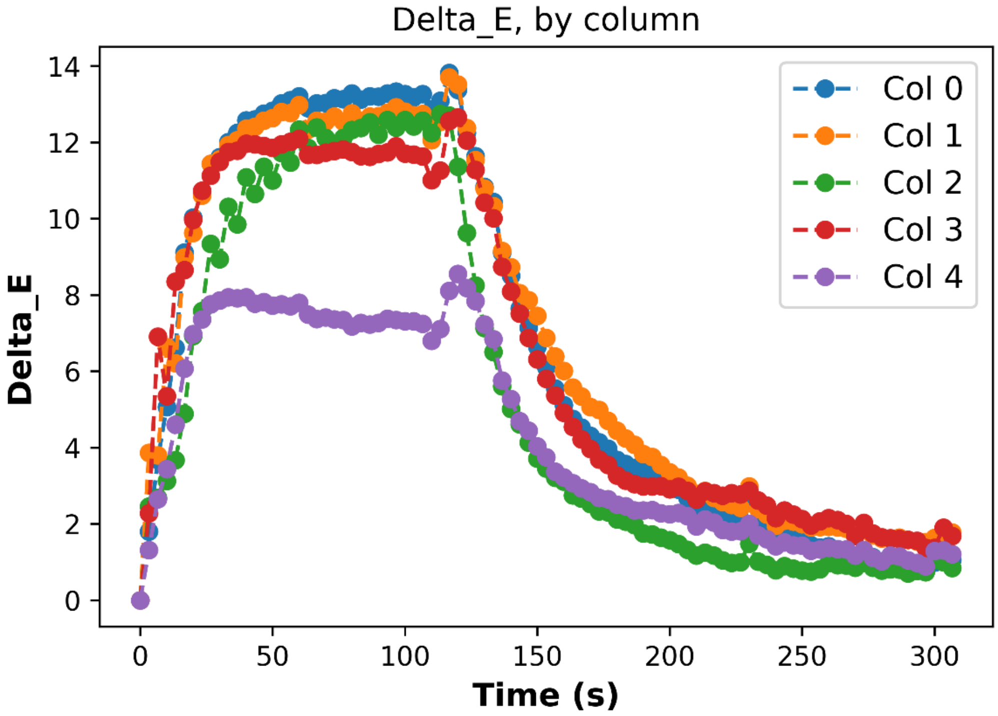
Delta_E, by column
Delta_E
Time (s)
Col 0
Col 1
Col 2
Col 3
Col 4

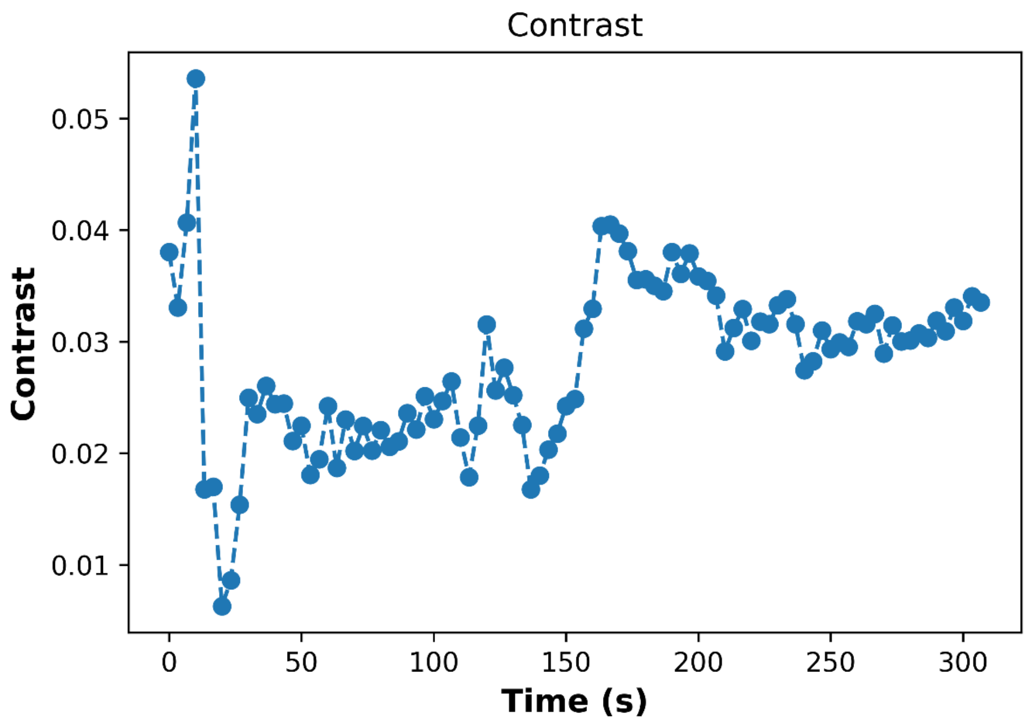
Contrast
0.05
0.04
0.03
0.02
0.01
Contrast
0
50
100
150
200
250
300
Time (s)

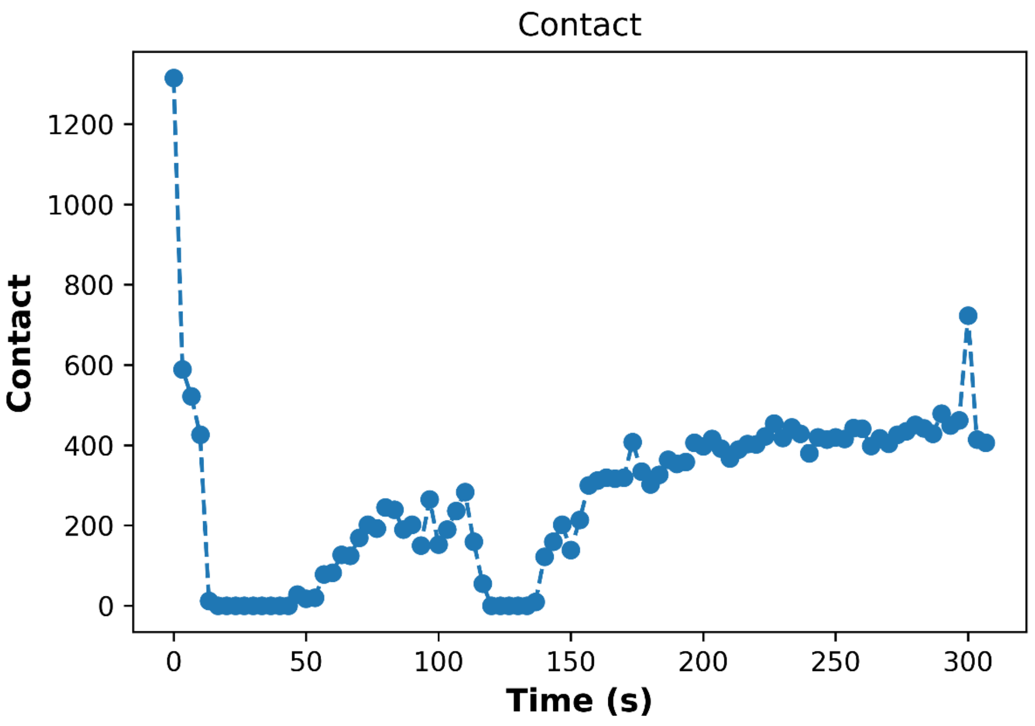
Contact
1200
1000
800
600
400
200
0
Contact
0
50
100
150
200
250
300
Time (s)

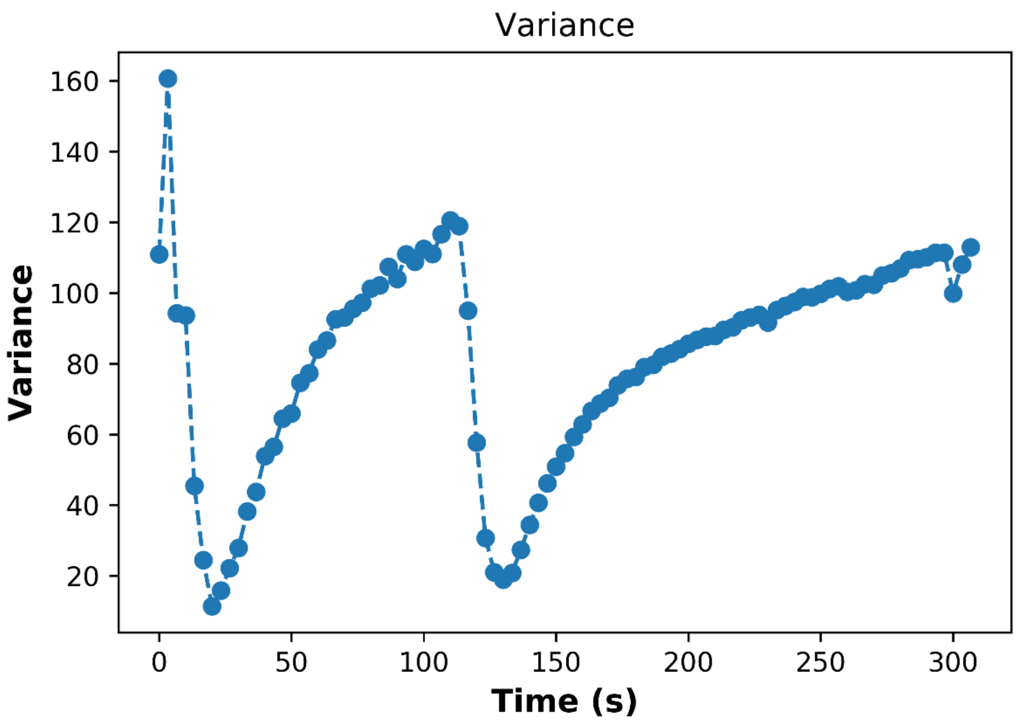
Variance
160
140
120
100
80
60
40
20
Variance
0
50
100
150
200
250
300
Time (s)

Table 1, Entry 2: 210 RPM, Paddle, No baffles, with probe

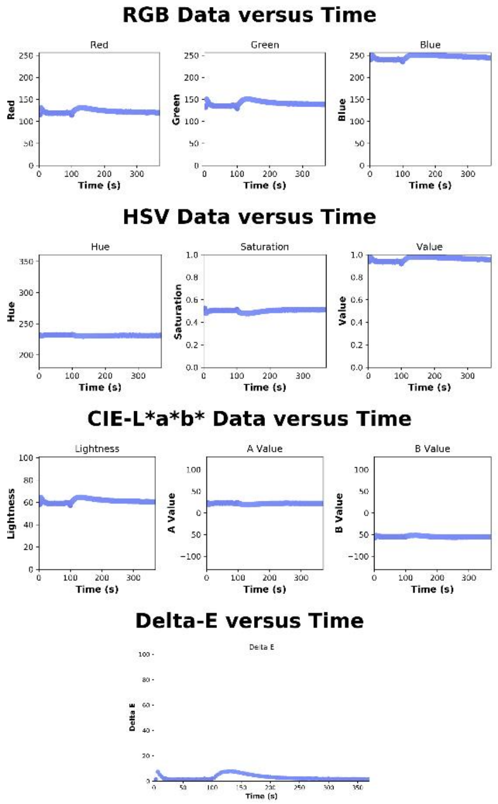

Kineticolor

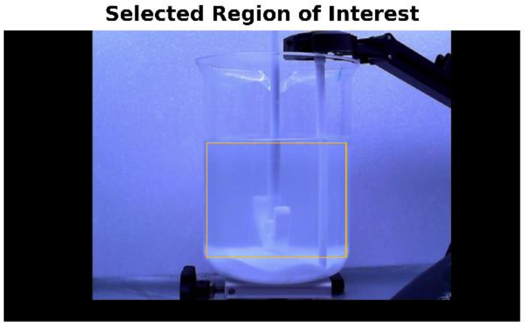

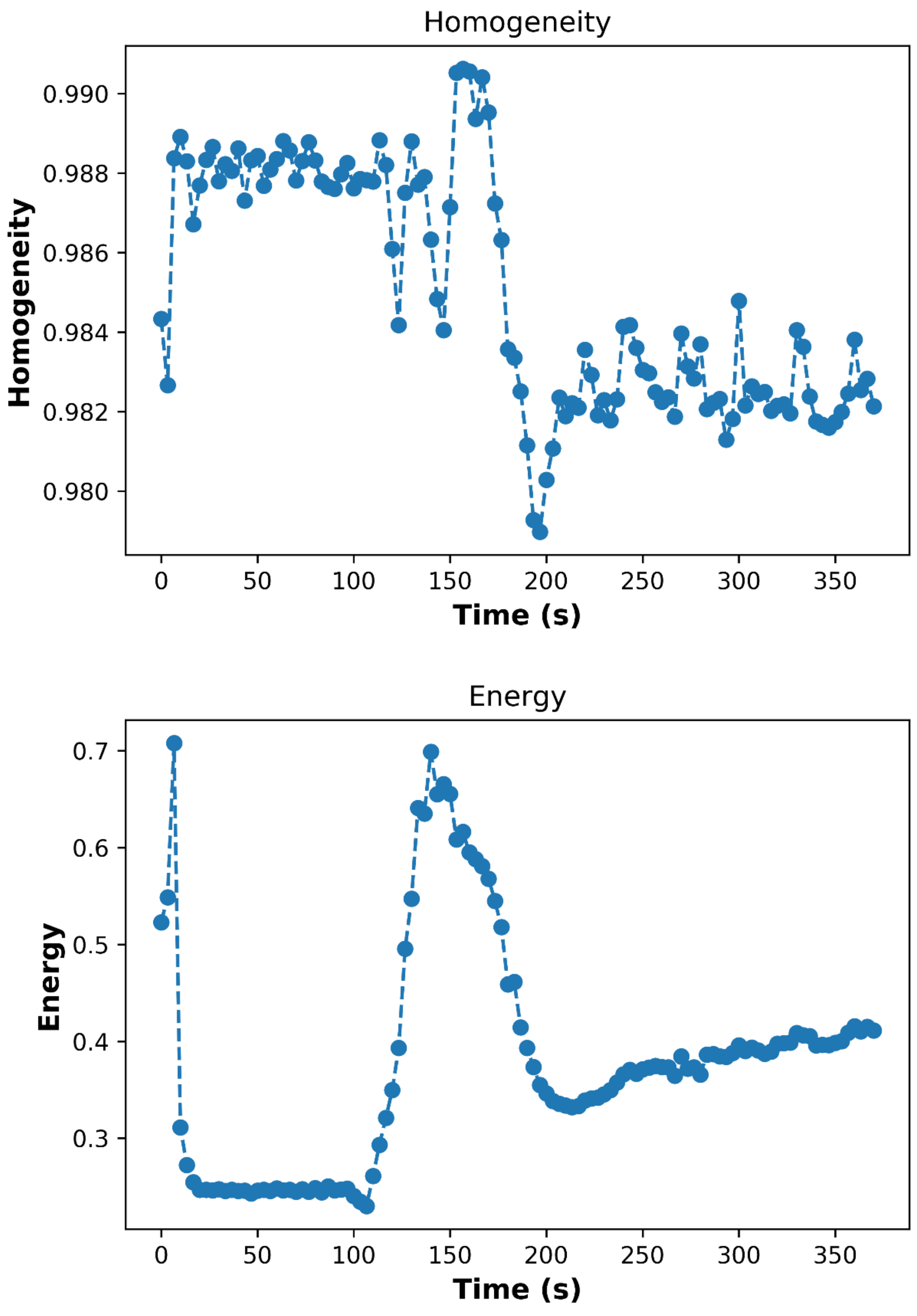
Homogeneity
0.990
0.988
0.986
0.984
0.982
0.980
Homogeneity
0
50
100
150
200
250
300
350
Time (s)
Energy
0.7
0.6
0.5
0.4
0.3
Energy
0
50
100
150
200
250
300
350
Time (s)

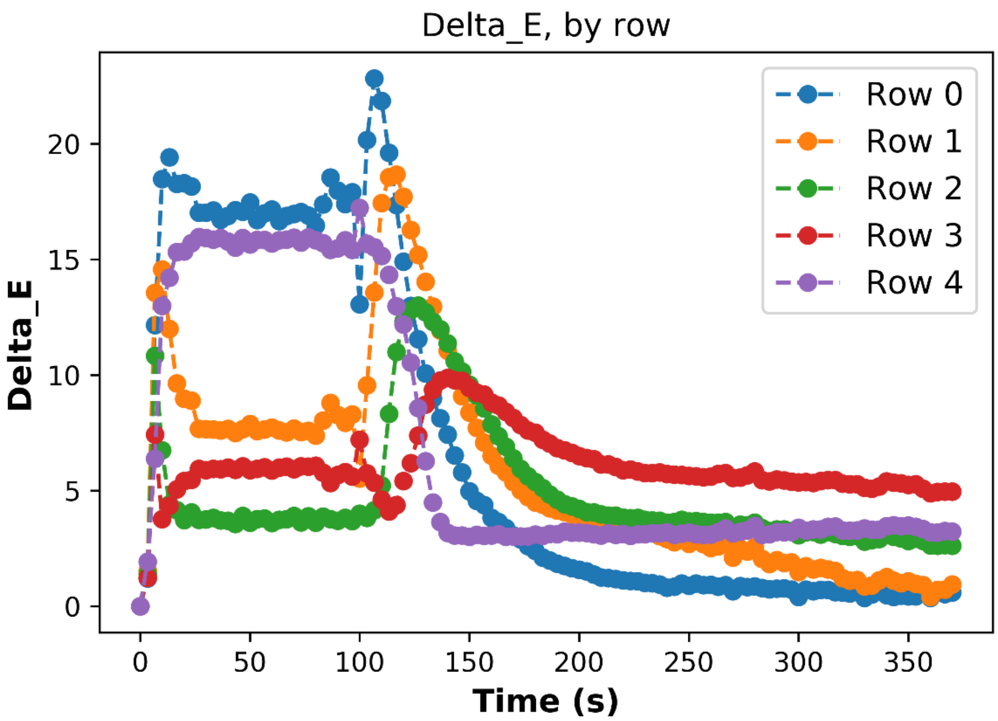

Delta_E, by row
Delta_E
Time (s)
Row 0
Row 1
Row 2
Row 3
Row 4

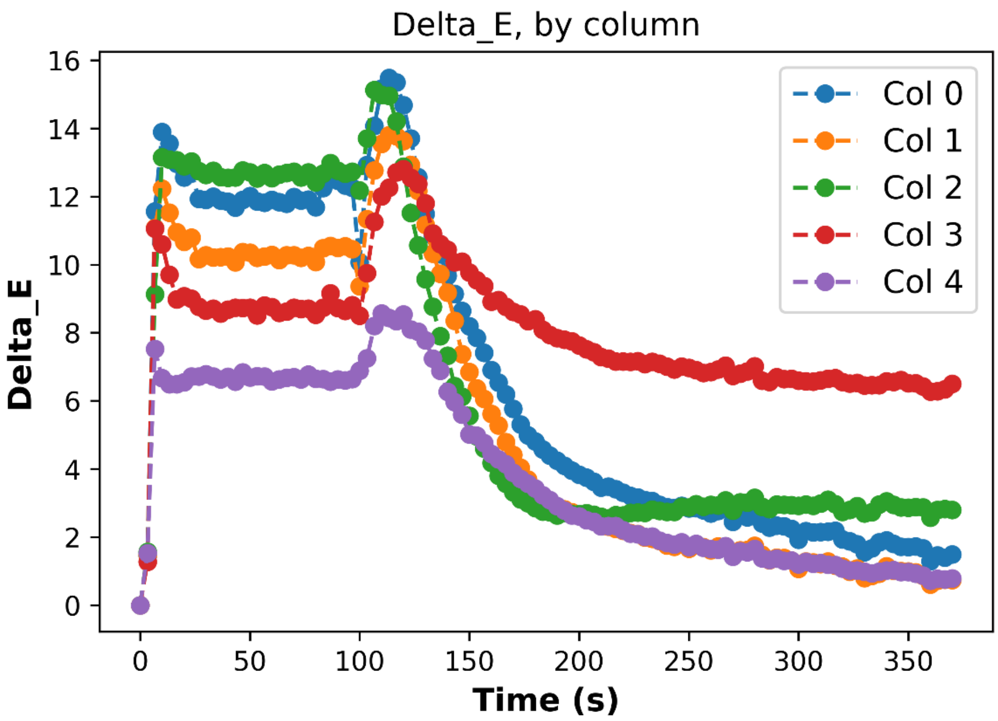

Delta_E, by column
Delta_E
Time (s)
Col 0
Col 1
Col 2
Col 3
Col 4

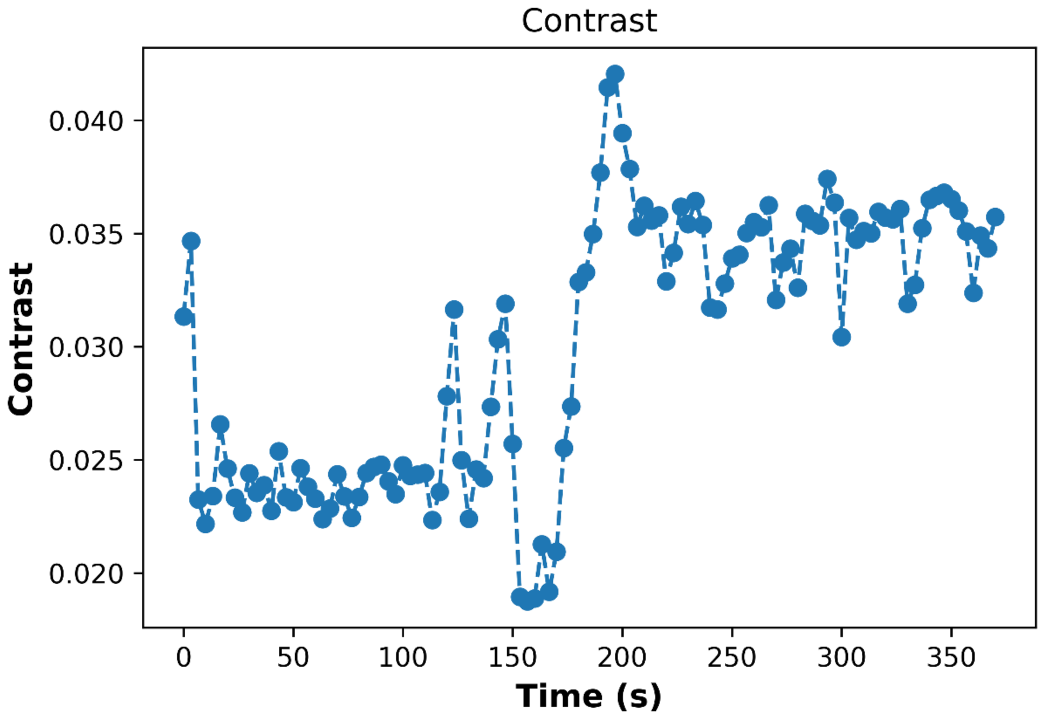
Contrast
Contrast
0.040
0.035
0.030
0.025
0.020
0
50
100
150
200
250
300
350
Time (s)

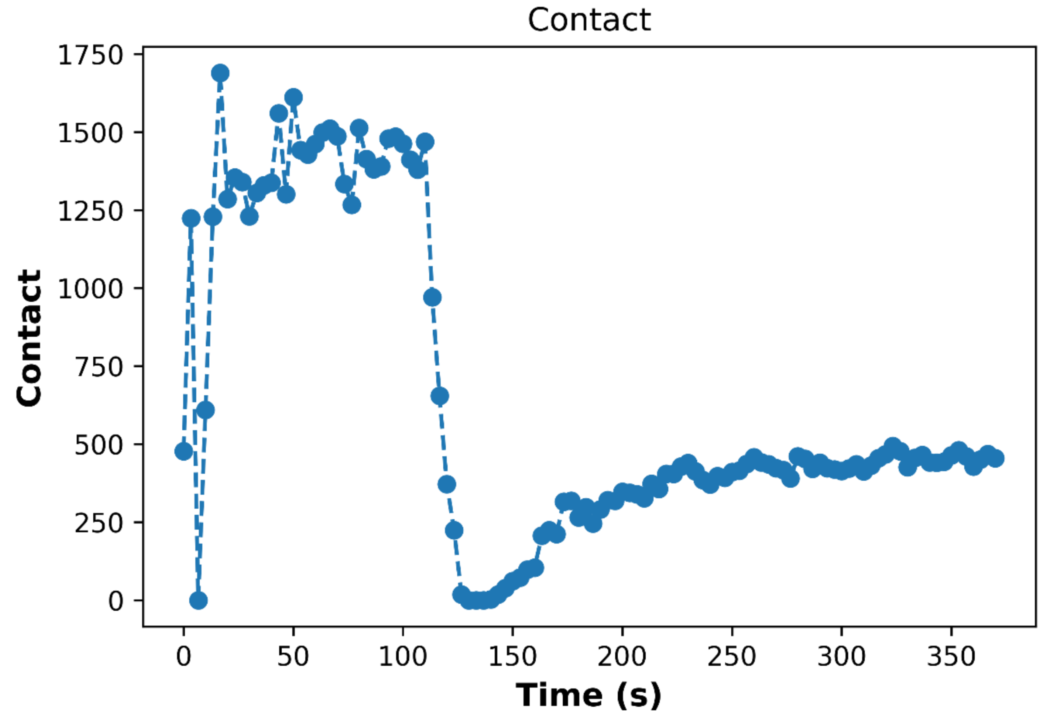
Contact
Contact
1750
1500
1250
1000
750
500
250
0
0
50
100
150
200
250
300
350
Time (s)

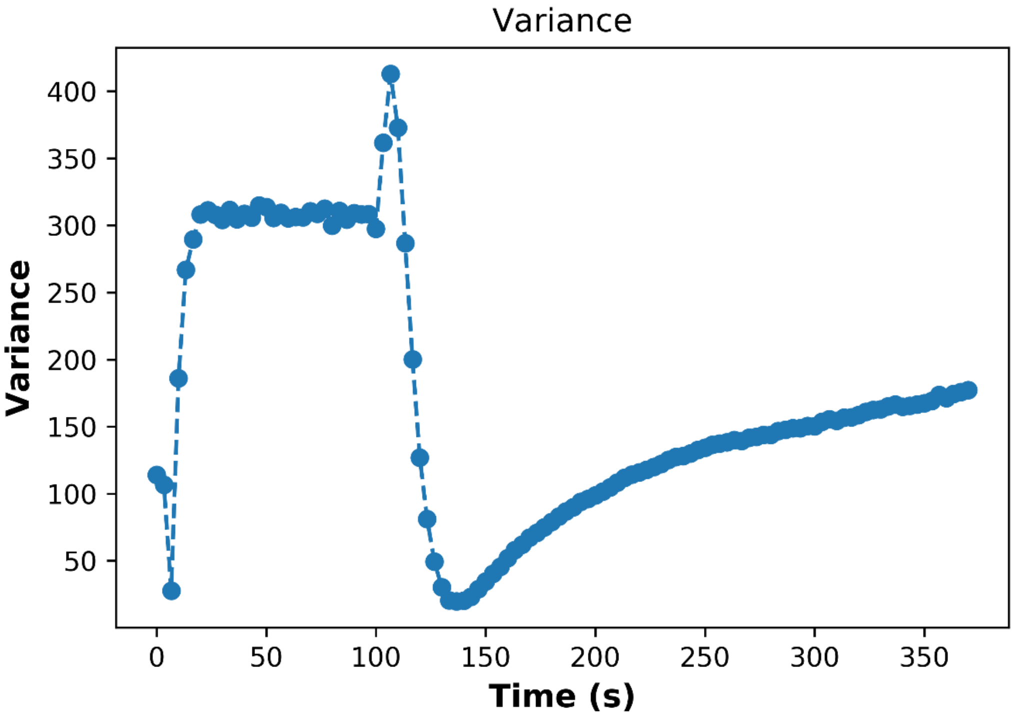

Variance
Variance
Time (s)
0
50
100
150
200
250
300
350
400

Table 1, Entry 2: 100 RPM, Paddle, No baffles

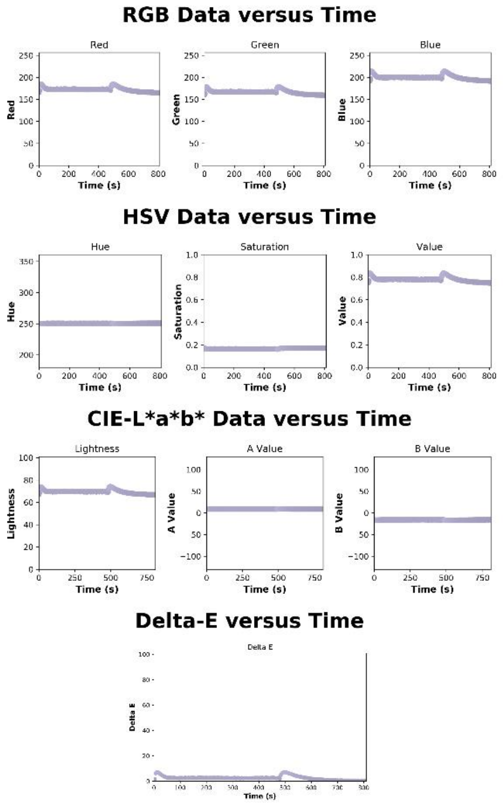

Kineticolor

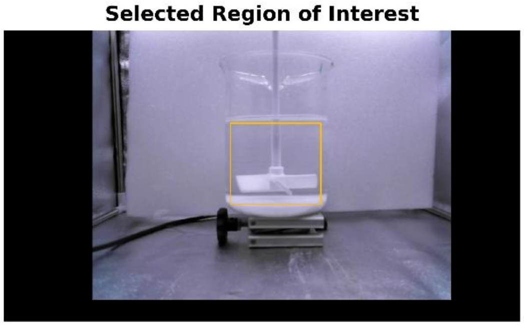

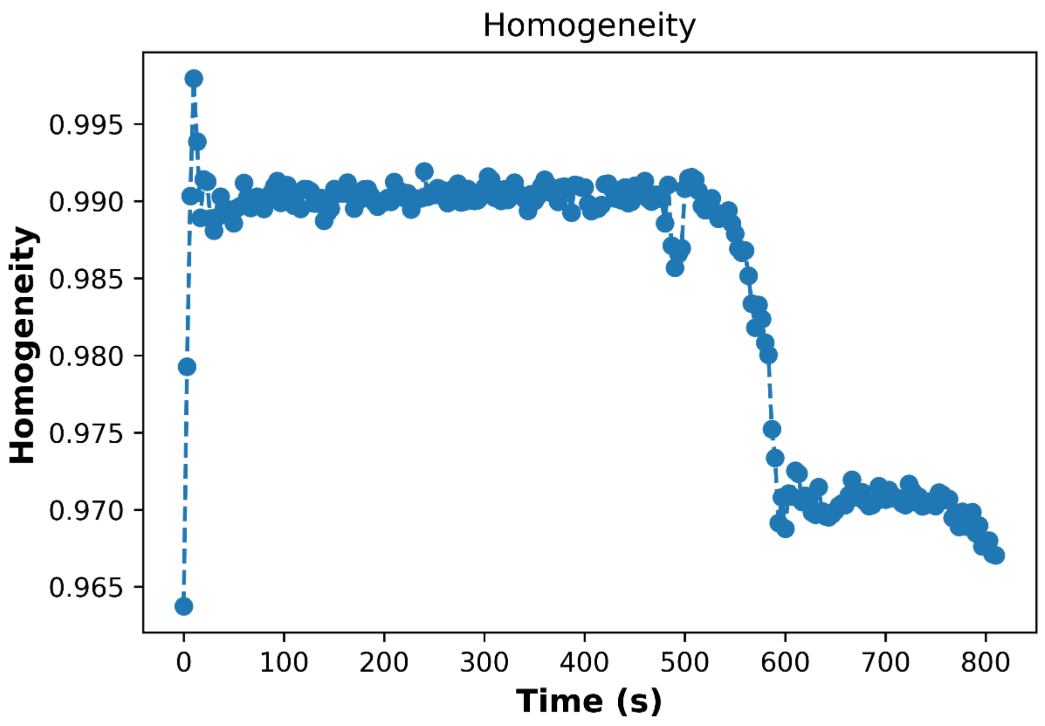
Homogeneity
Homogeneity
0.995
0.990
0.985
0.980
0.975
0.970
0.965
0
100
200
300
400
500
600
700
800
Time (s)

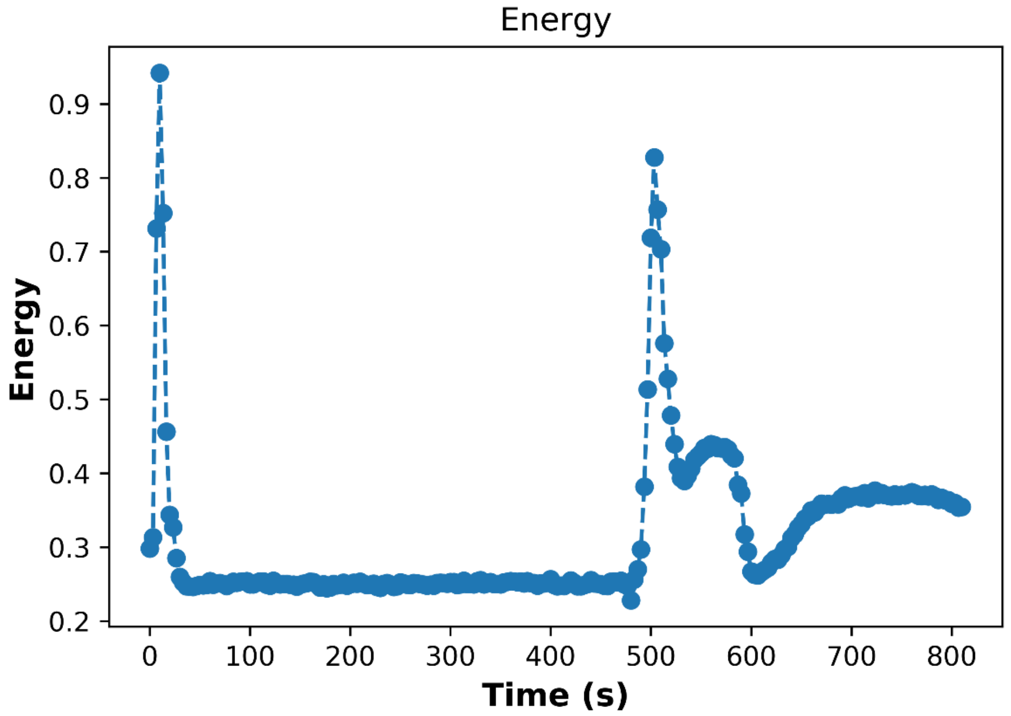
Energy
Energy
0.9
0.8
0.7
0.6
0.5
0.4
0.3
0.2
0
100
200
300
400
500
600
700
800
Time (s)

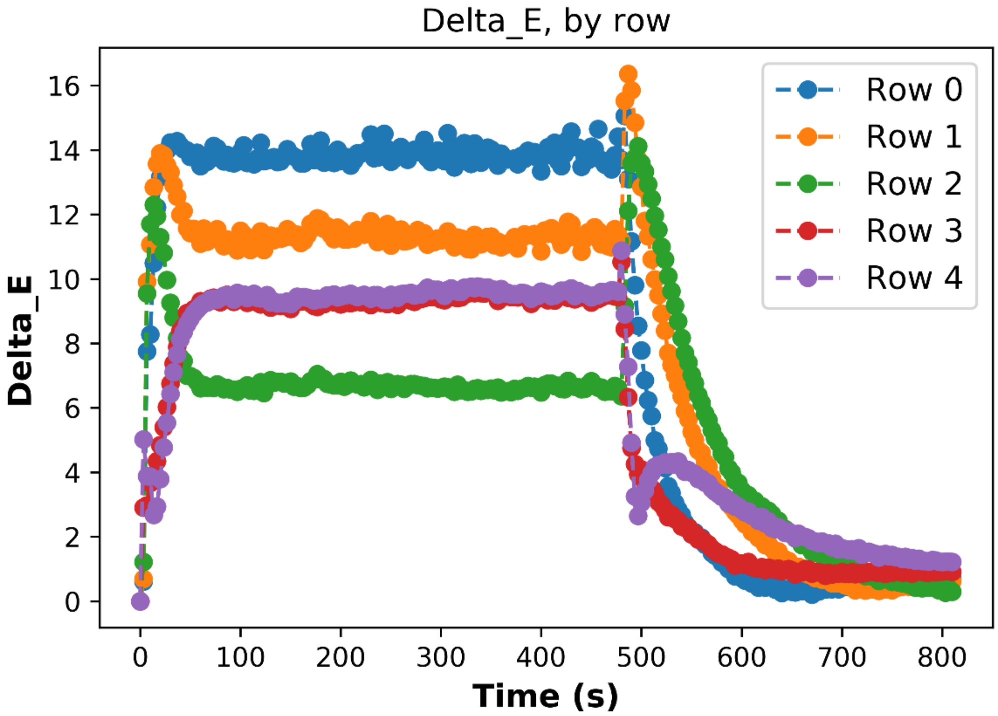
Delta_E, by row
Delta_E
Time (s)
Row 0
Row 1
Row 2
Row 3
Row 4
0
2
4
6
8
10
12
14
16
0
100
200
300
400
500
600
700
800

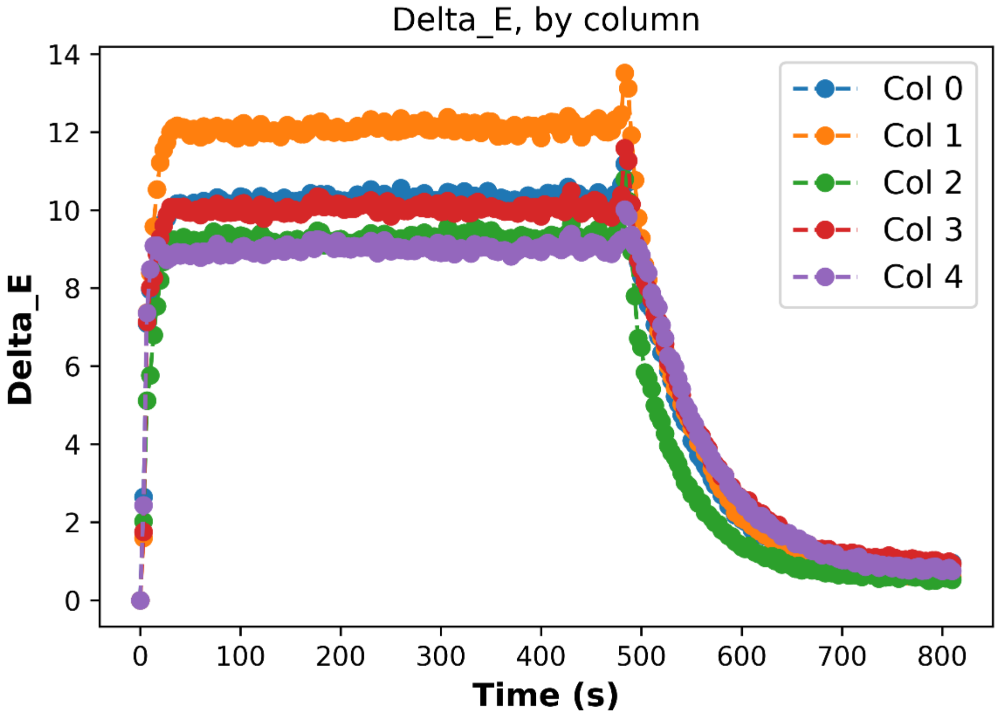
Delta_E, by column
Delta_E
Time (s)
Col 0
Col 1
Col 2
Col 3
Col 4
0
2
4
6
8
10
12
14
0
100
200
300
400
500
600
700
800

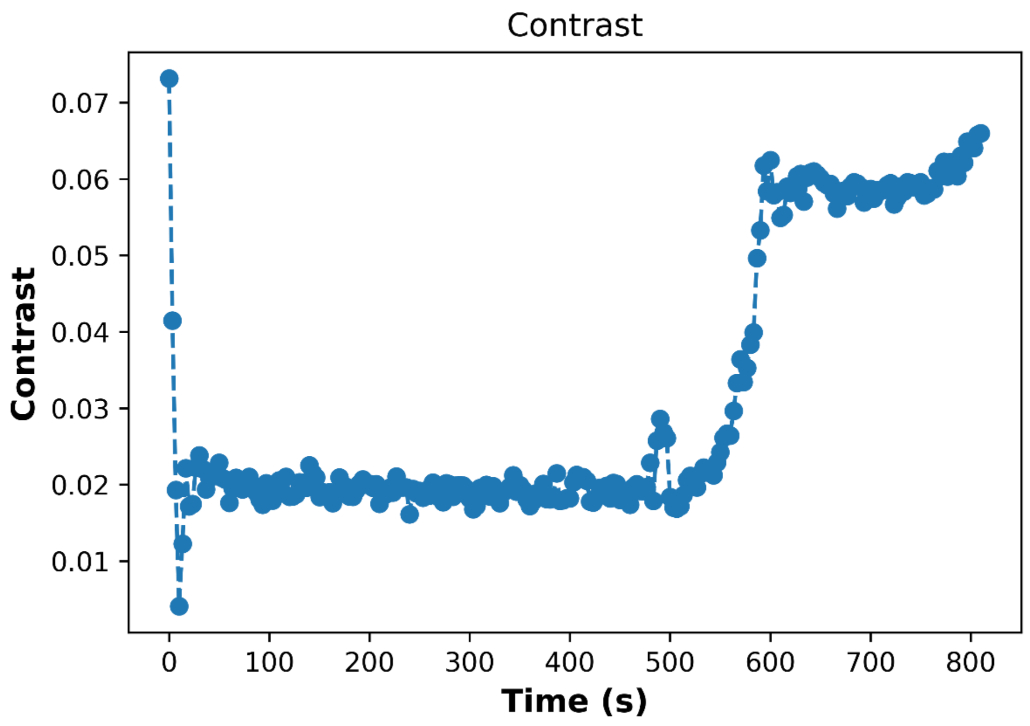
Contrast
0.07
0.06
0.05
0.04
0.03
0.02
0.01
Contrast
0
100
200
300
400
500
600
700
800
Time (s)

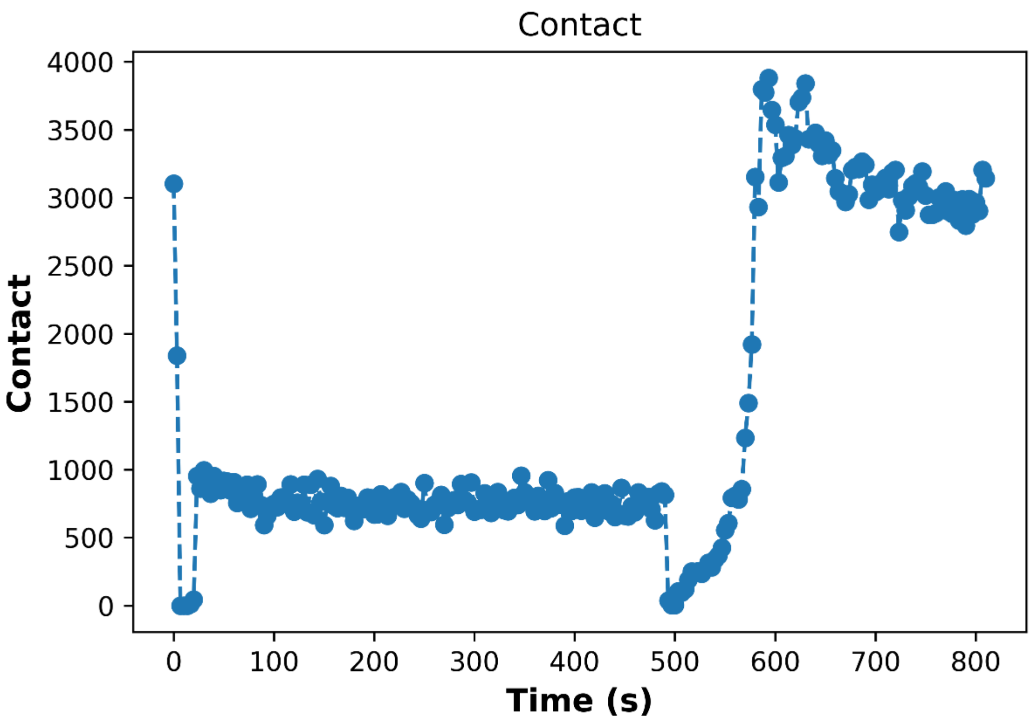
Contact
4000
3500
3000
2500
2000
1500
1000
500
0
Contact
0
100
200
300
400
500
600
700
800
Time (s)

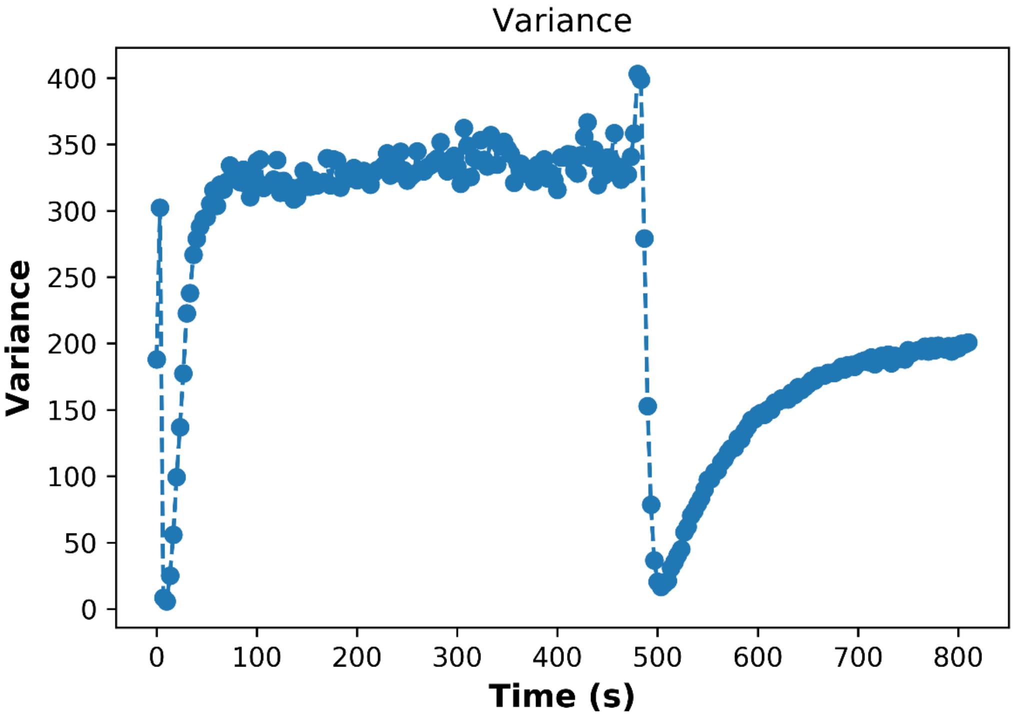
Variance
400
350
300
250
200
150
100
50
0
Variance
0
100
200
300
400
500
600
700
800
Time (s)

Table 1, Entry 3: 210 RPM, Paddle, No baffles

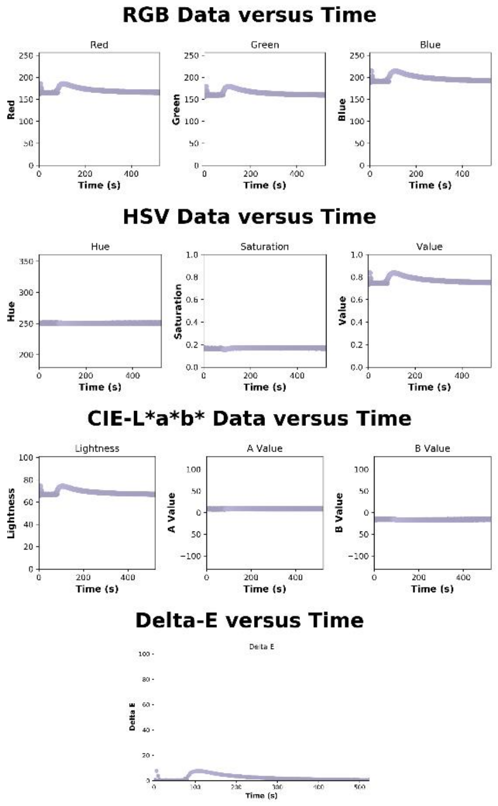

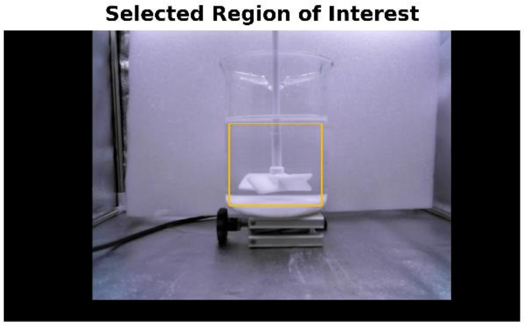

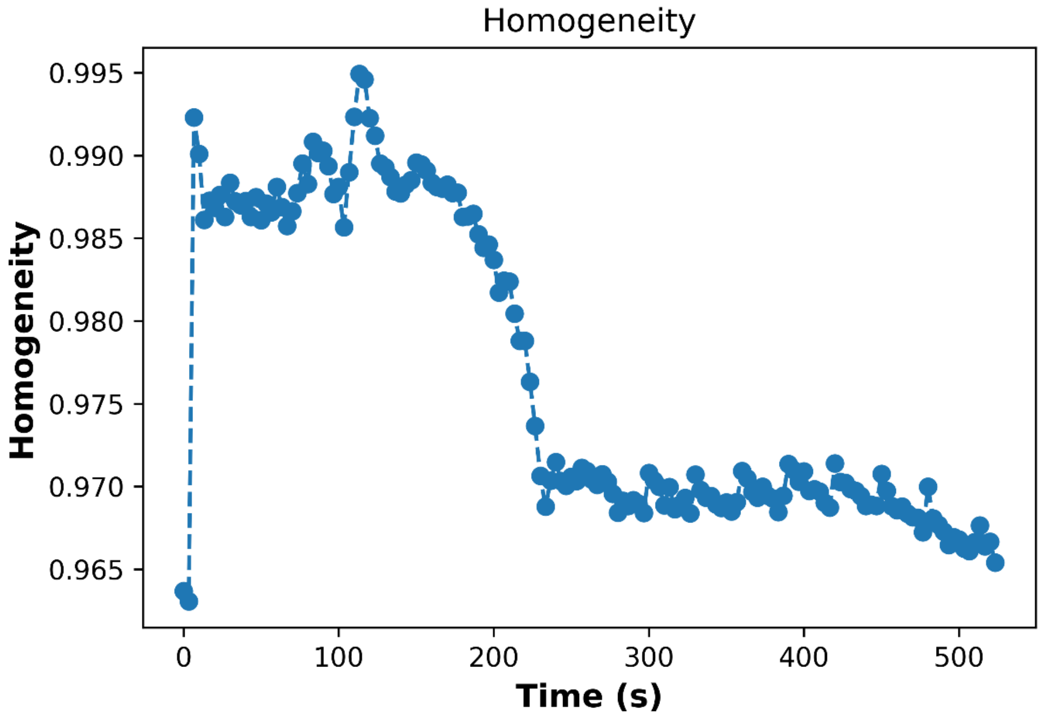

Homogeneity
0.995
0.990
0.985
0.980
0.975
0.970
0.965
Homogeneity
0
100
200
300
400
500
Time (s)

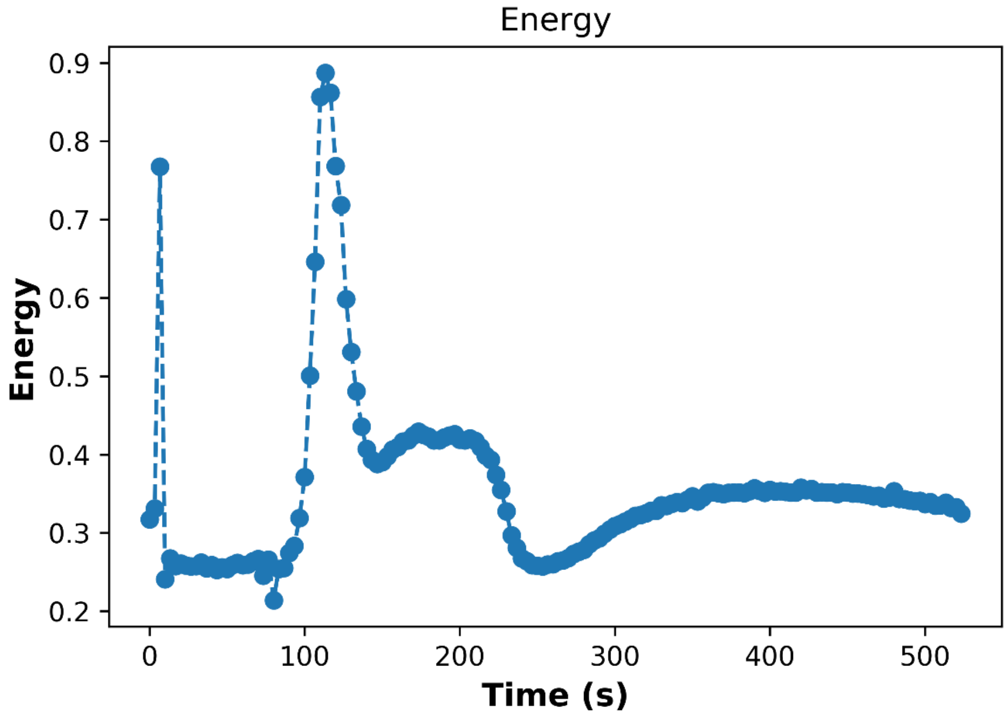

Energy
0.9
0.8
0.7
0.6
0.5
0.4
0.3
0.2
Energy
0
100
200
300
400
500
Time (s)

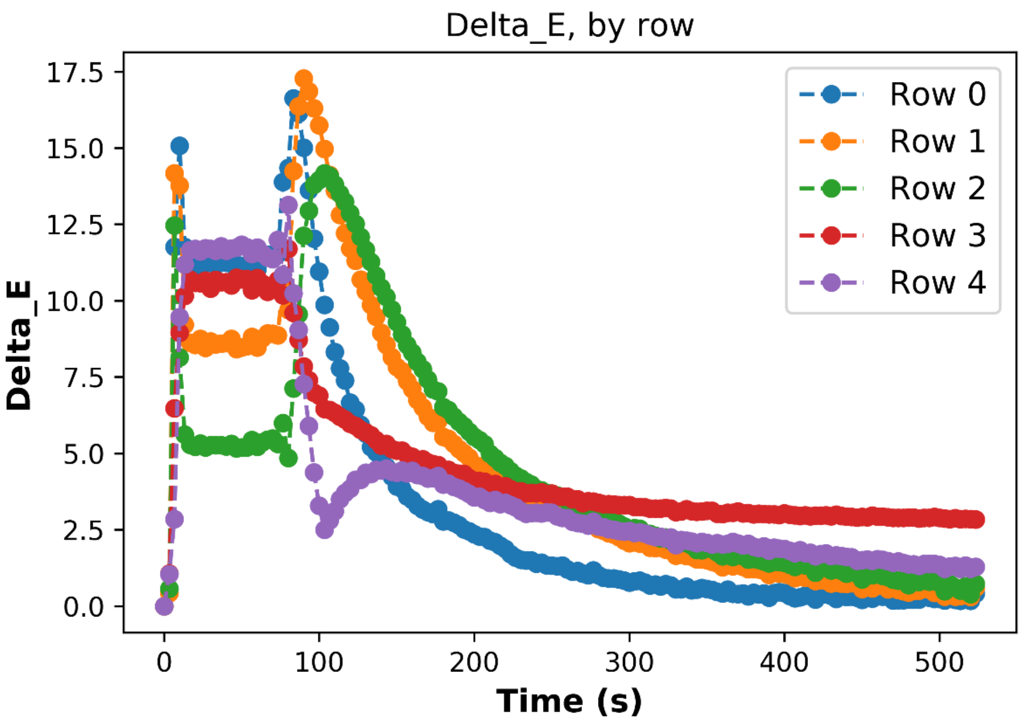
Delta_E, by row
Row 0
Row 1
Row 2
Row 3
Row 4
Delta_E
Time (s)
17.5
15.0
12.5
10.0
7.5
5.0
2.5
0.0
0
100
200
300
400
500

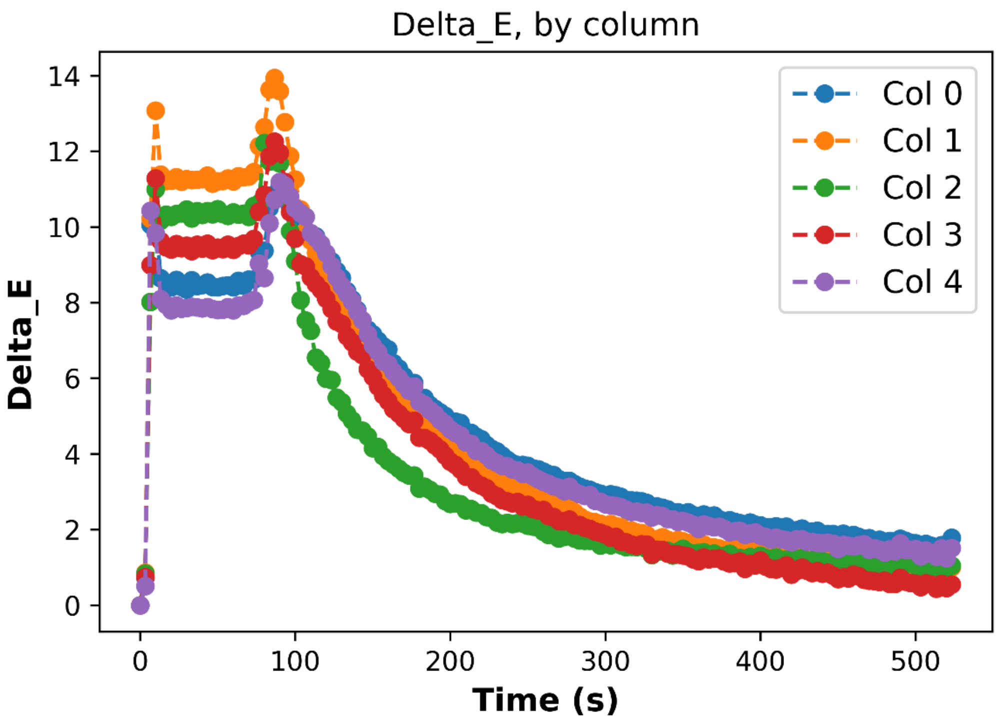
Delta_E, by column
Col 0
Col 1
Col 2
Col 3
Col 4
Delta_E
Time (s)
14
12
10
8
6
4
2
0
0
100
200
300
400
500

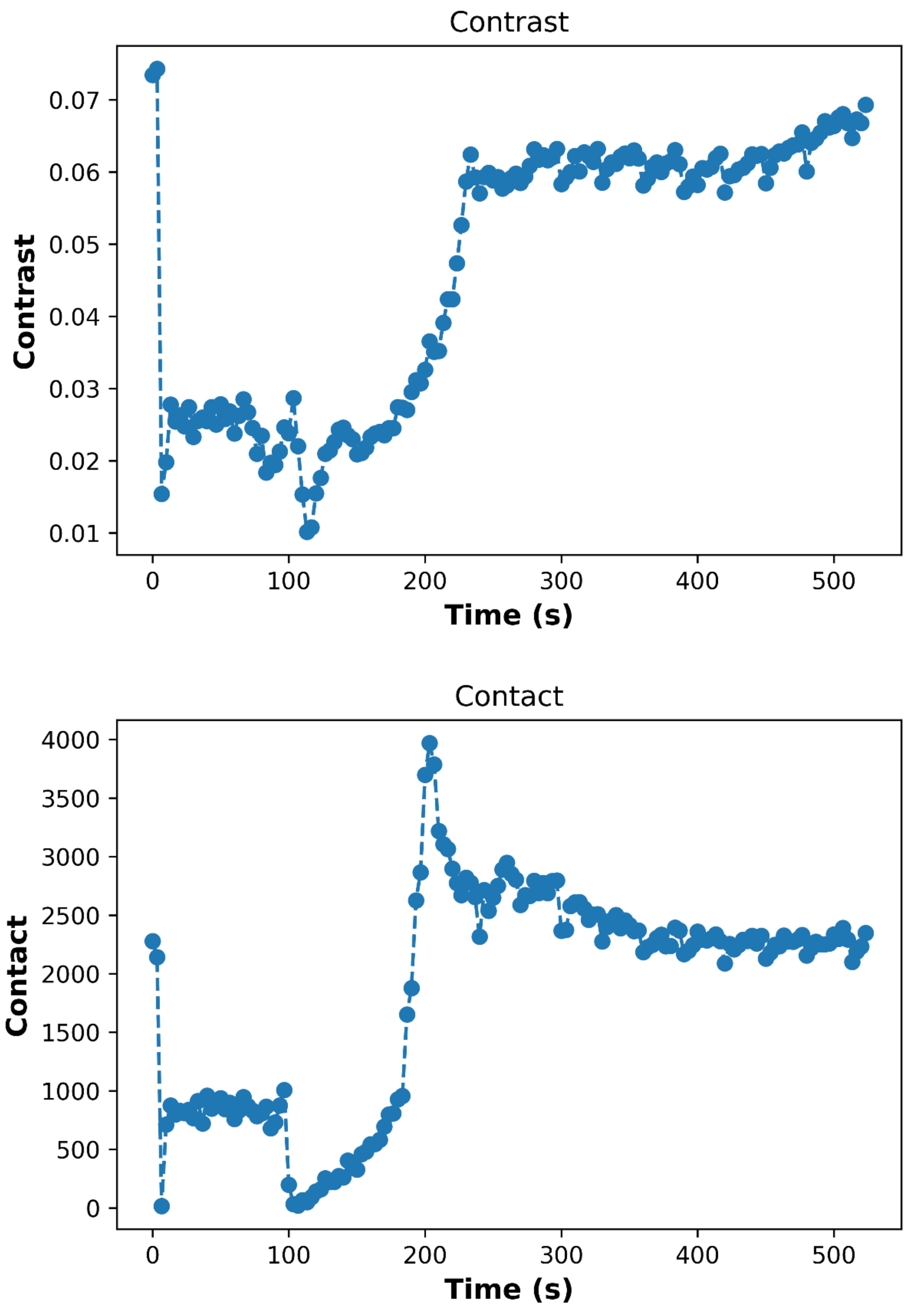
Contrast
Contrast
0.07
0.06
0.05
0.04
0.03
0.02
0.01
0
100
200
300
400
500
Time (s)
Contact
Contact
4000
3500
3000
2500
2000
1500
1000
500
0
0
100
200
300
400
500
Time (s)

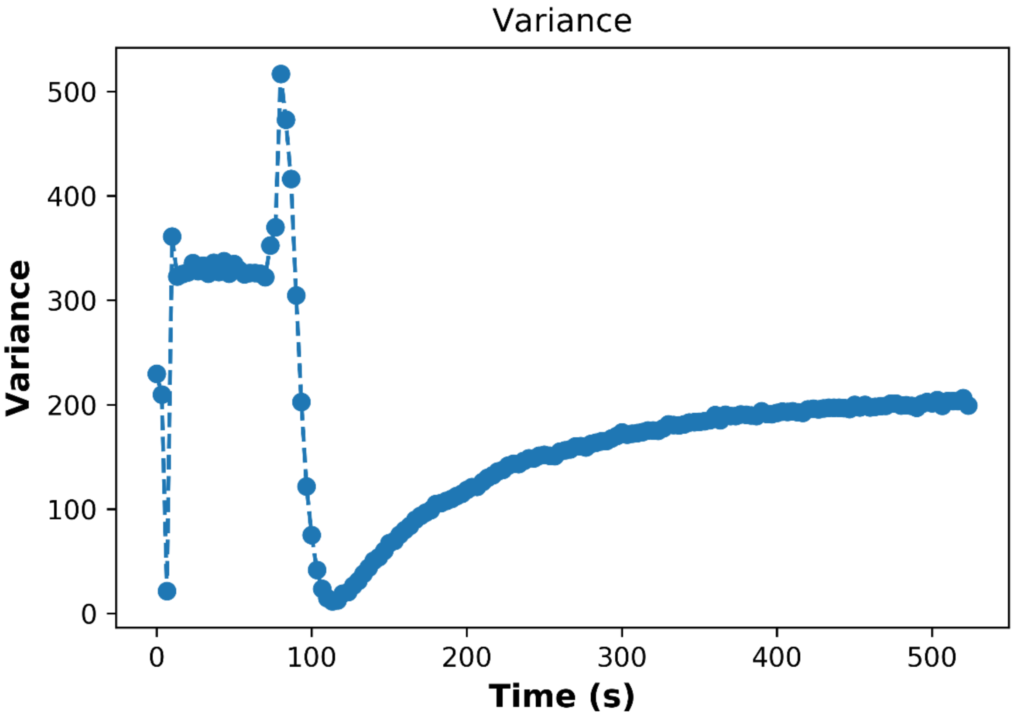

Variance
500
400
300
200
100
0
Variance
0
100
200
300
400
500
Time (s)

Table 1, Entry 4: 60 RPM, Anchor, No baffles

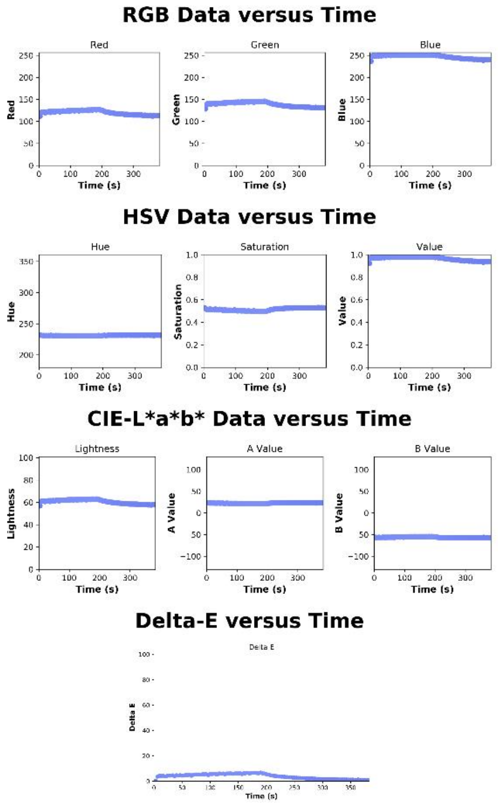

Kineticolor

Selected Region of Interest

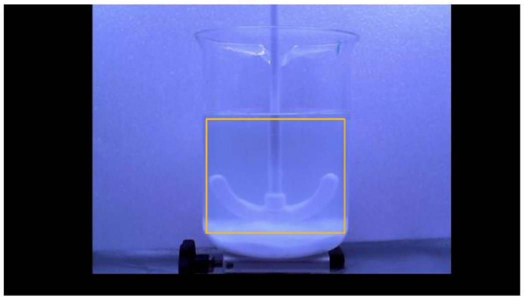

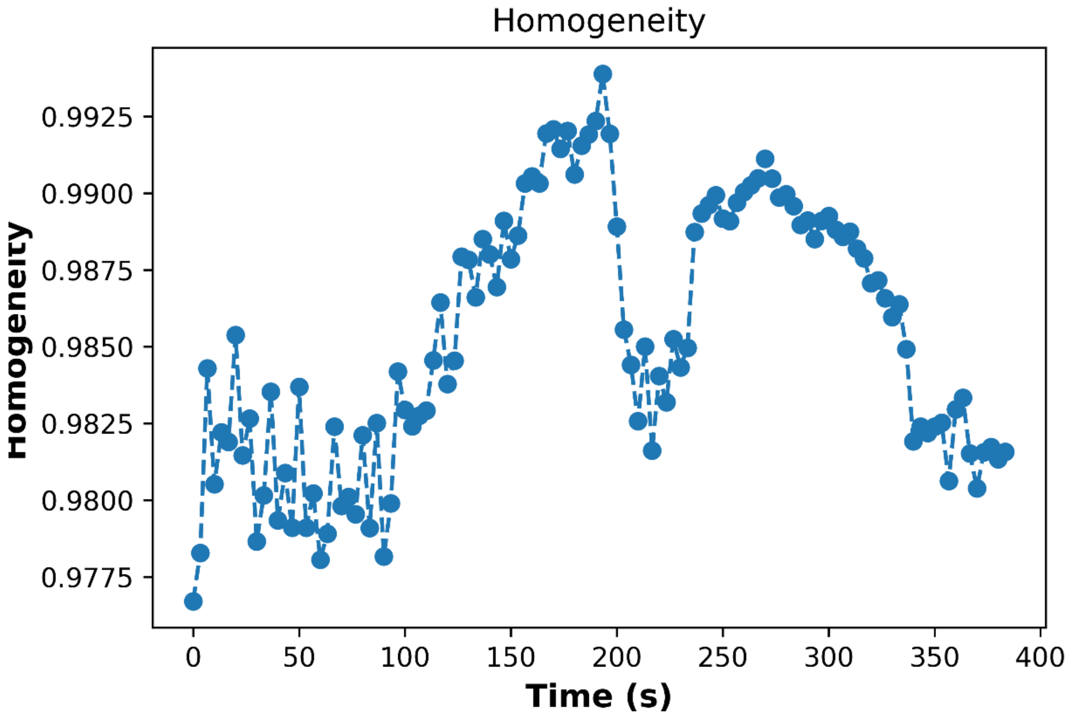

Homogeneity
Homogeneity
0.9925
0.9900
0.9875
0.9850
0.9825
0.9800
0.9775
0
50
100
150
200
250
300
350
400
Time (s)

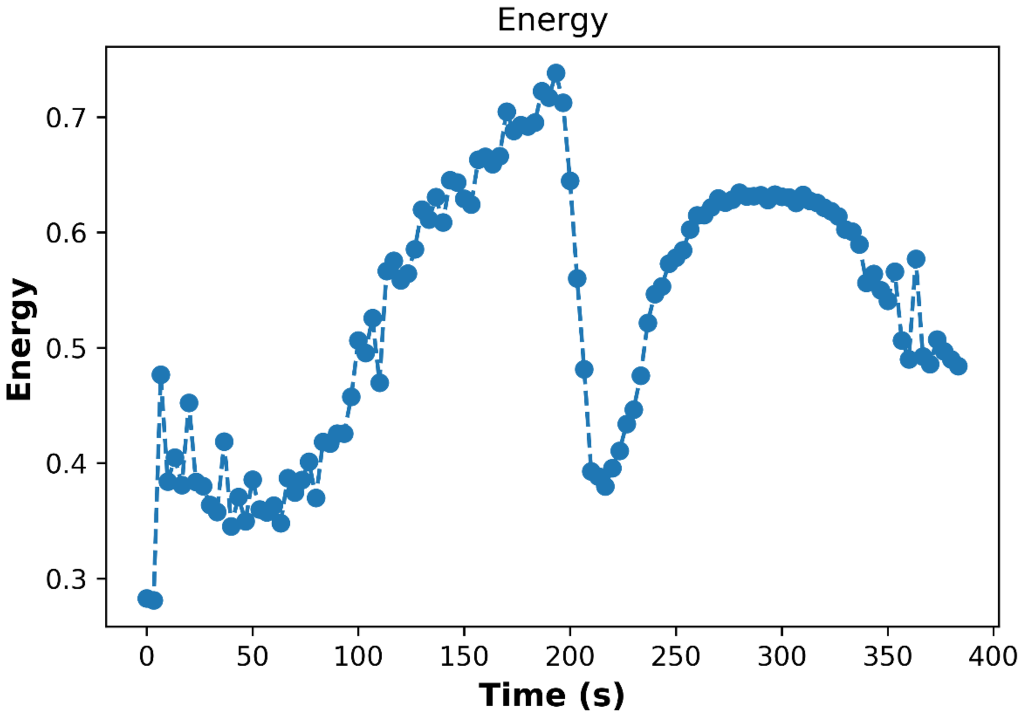

Energy
Energy
0.7
0.6
0.5
0.4
0.3
0
50
100
150
200
250
300
350
400
Time (s)

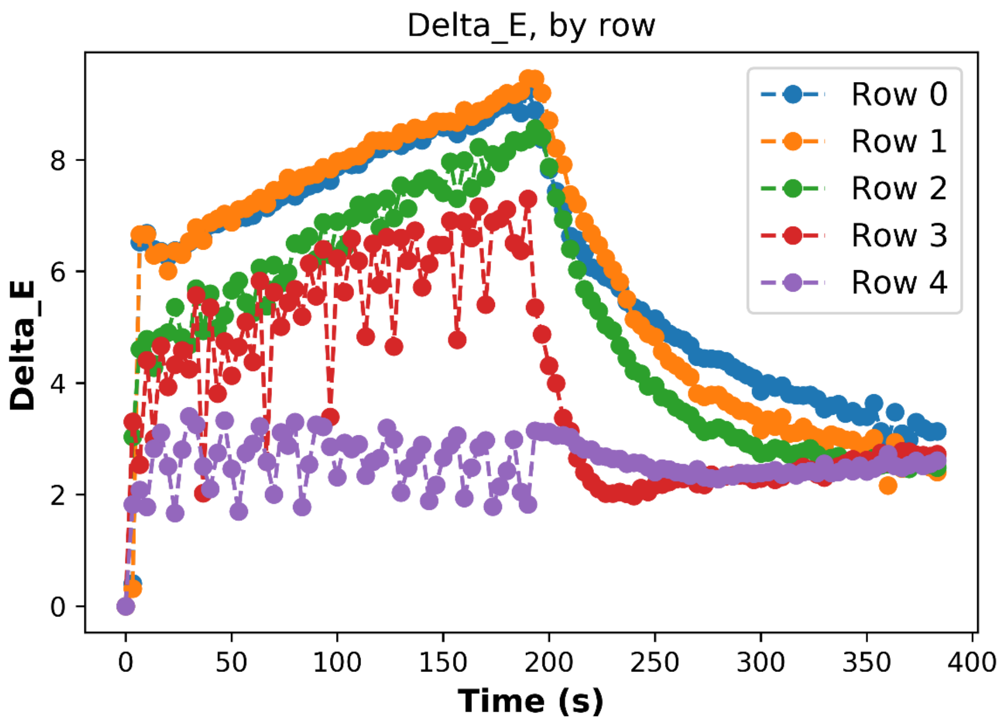
Delta_E, by row
Row 0
Row 1
Row 2
Row 3
Row 4
Delta_E
0
2
4
6
8
0
50
100
150
200
250
300
350
400
Time (s)

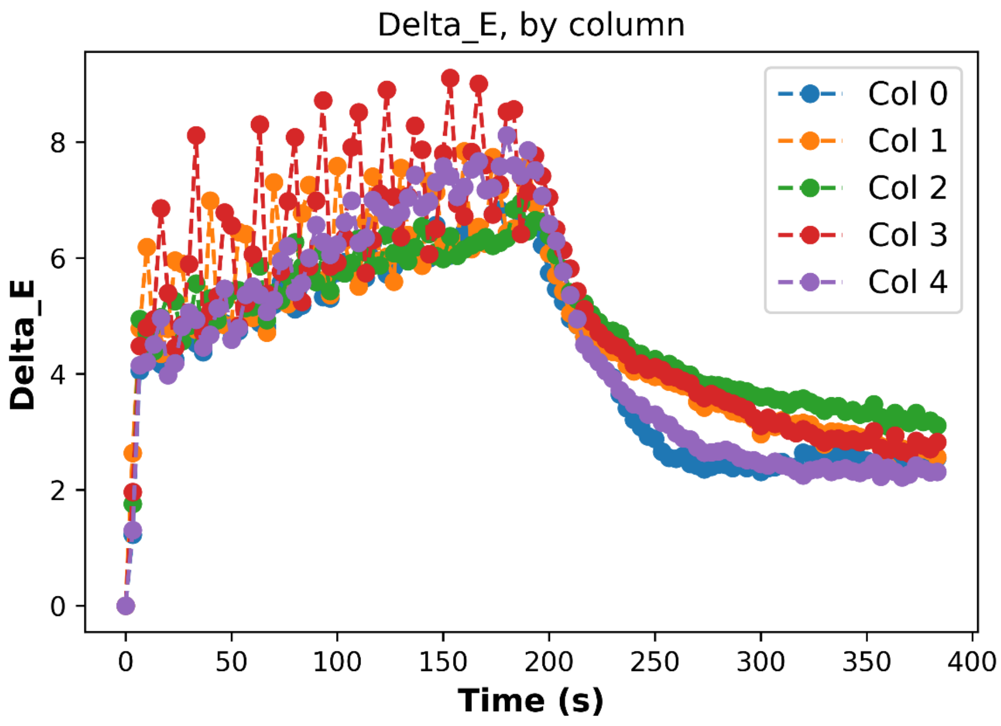
Delta_E, by column
Col 0
Col 1
Col 2
Col 3
Col 4
Delta_E
0
2
4
6
8
0
50
100
150
200
250
300
350
400
Time (s)

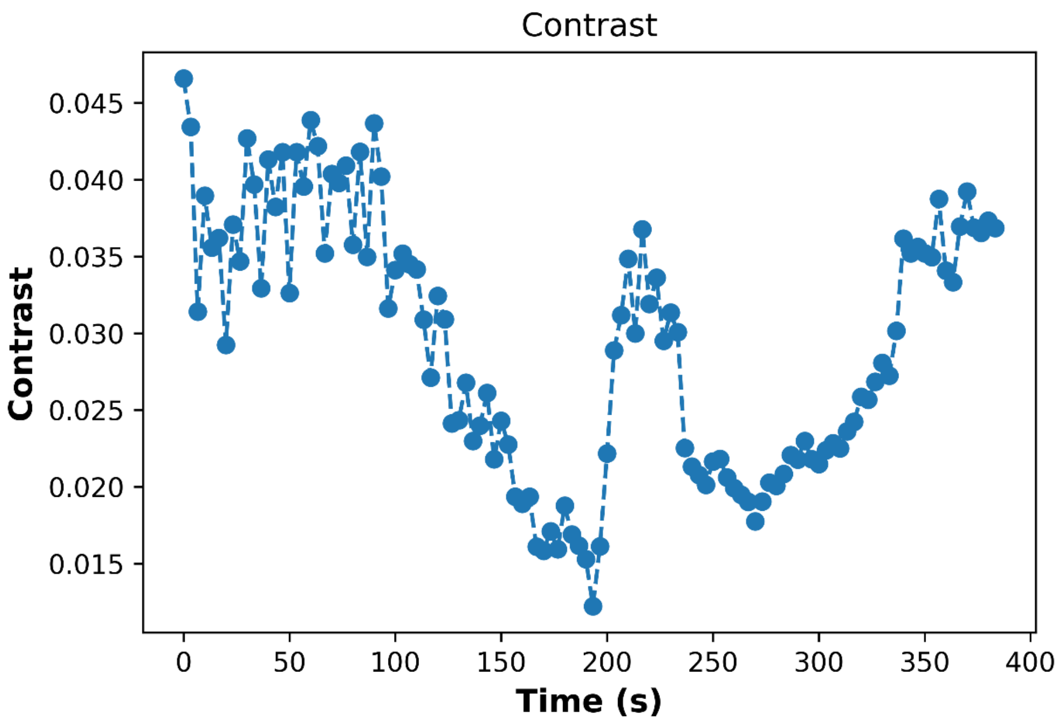
Contrast
Contrast
0.045
0.040
0.035
0.030
0.025
0.020
0.015
0
50
100
150
200
250
300
350
400
Time (s)

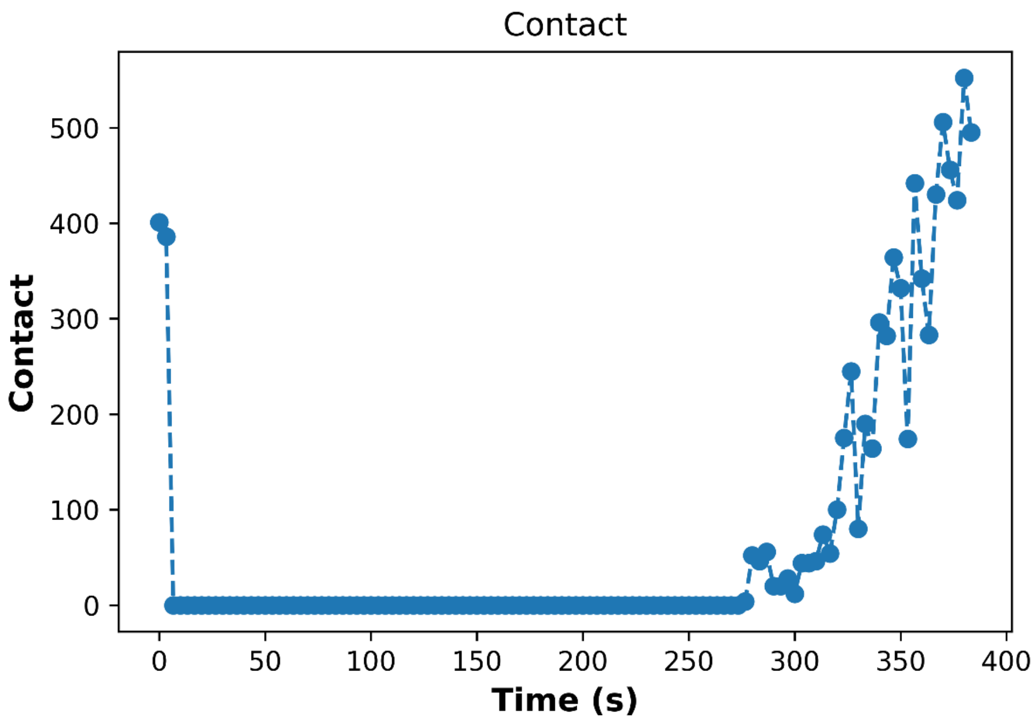
Contact
Contact
500
400
300
200
100
0
0
50
100
150
200
250
300
350
400
Time (s)

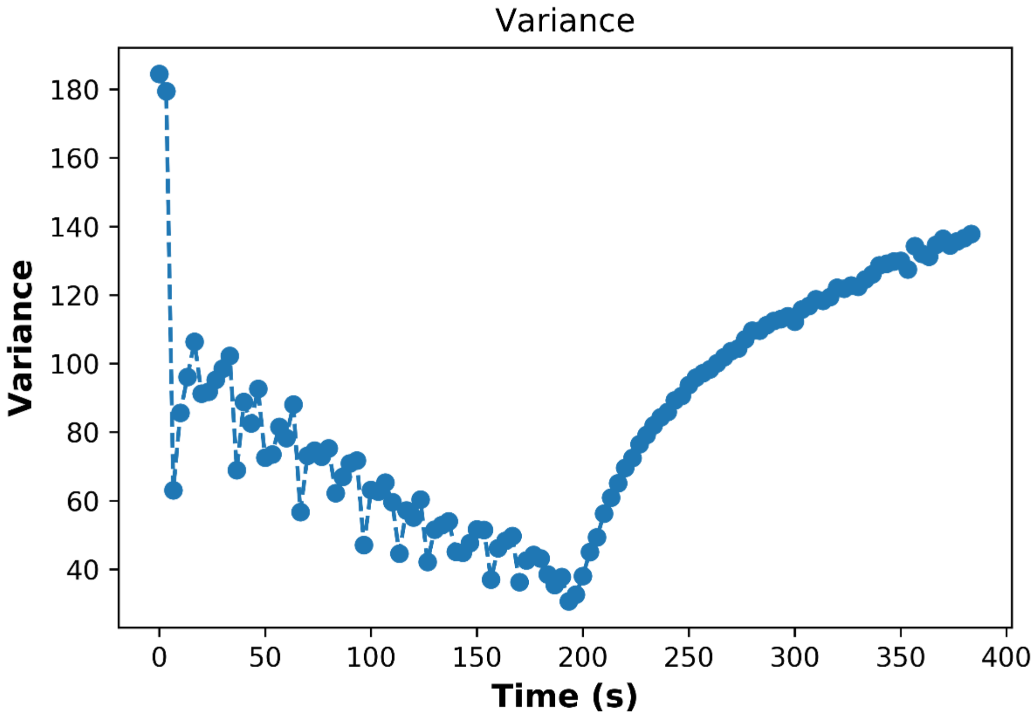
Variance
180
160
140
120
100
80
60
40
Variance
0
50
100
150
200
250
300
350
400
Time (s)

Table 1, Entry 4: 60 RPM, Anchor, No baffles, with probe

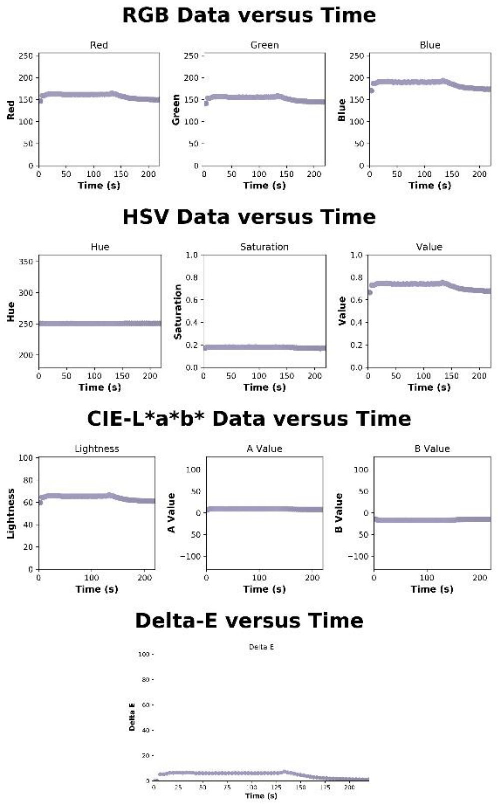

Kineticolor

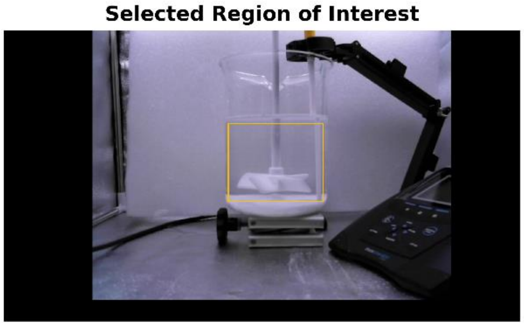

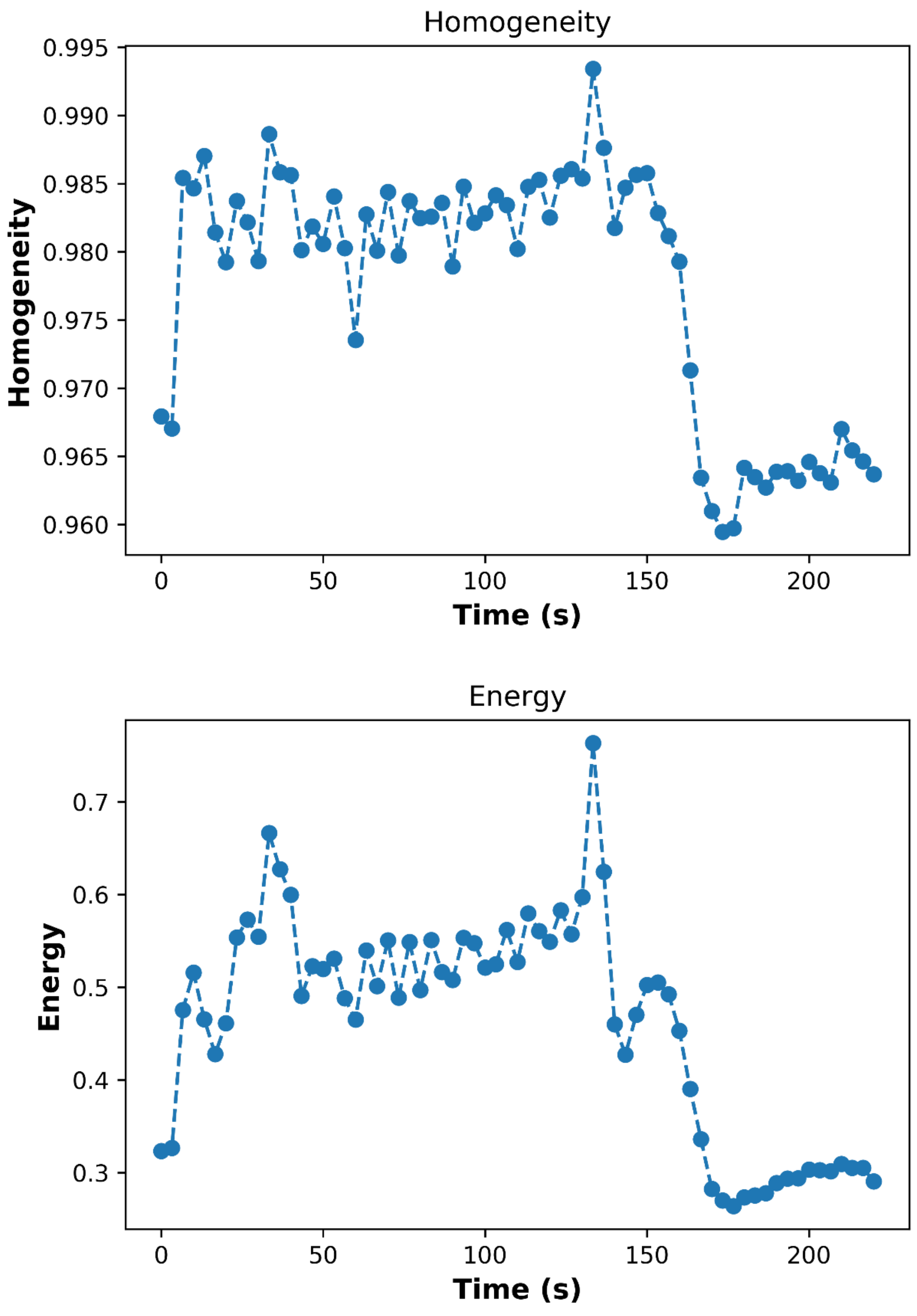
Homogeneity
0.995
0.990
0.985
0.980
0.975
0.970
0.965
0.960
Homogeneity
0
50
100
150
200
Time (s)
Energy
0.7
0.6
0.5
0.4
0.3
Energy
0
50
100
150
200
Time (s)

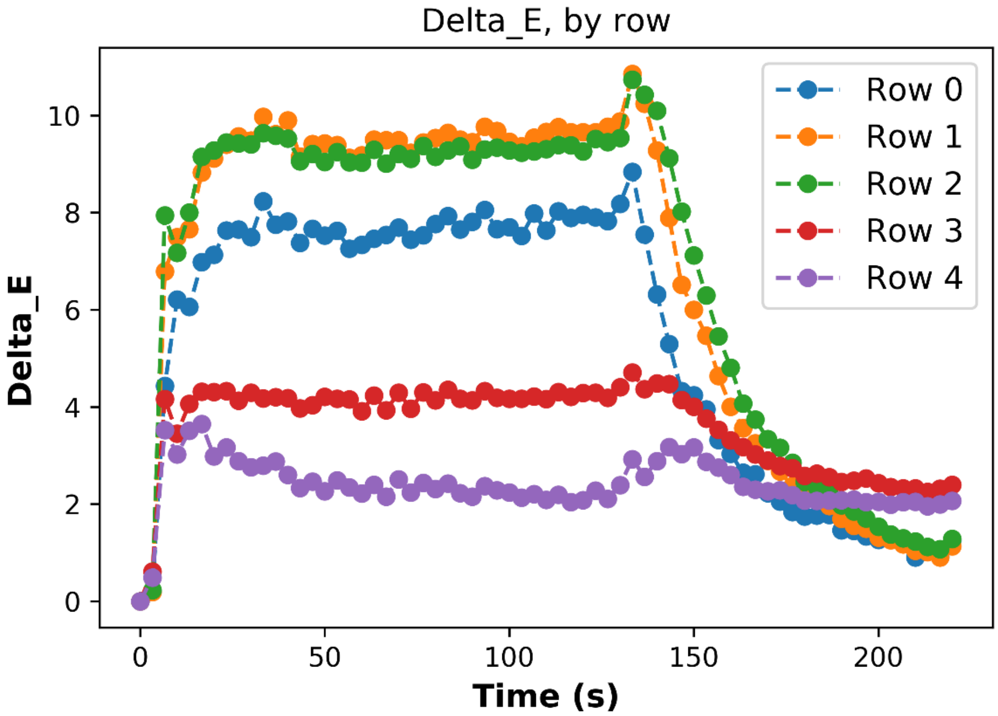
Delta_E, by row
Row 0
Row 1
Row 2
Row 3
Row 4
Delta_E
Time (s)
0
2
4
6
8
10
0
50
100
150
200

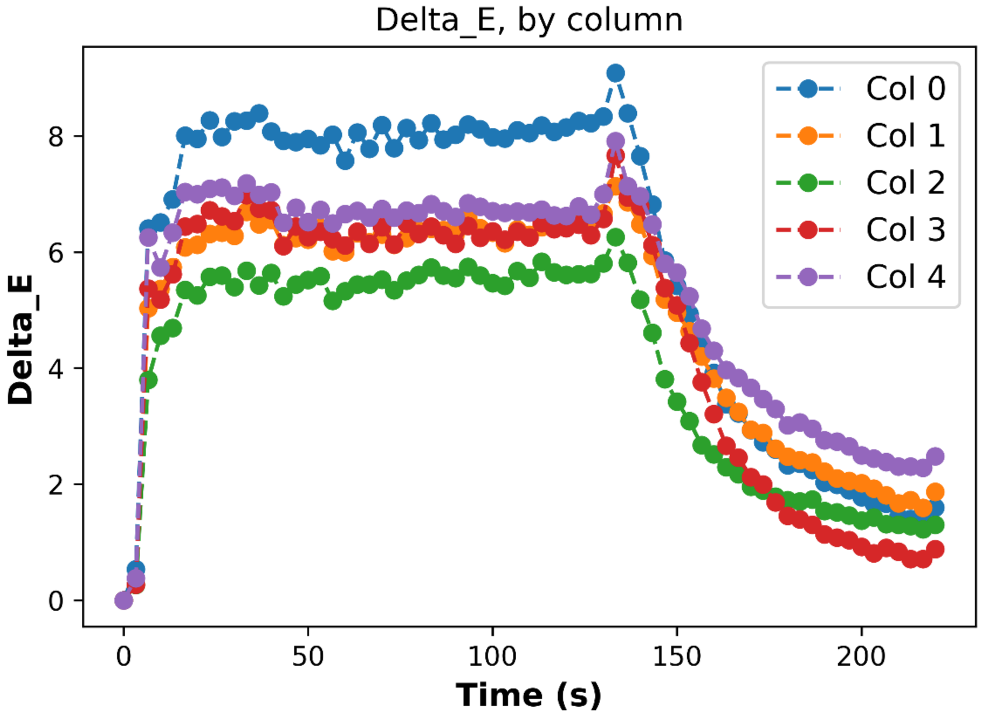
Delta_E, by column
Col 0
Col 1
Col 2
Col 3
Col 4
Delta_E
Time (s)
0
2
4
6
8
0
50
100
150
200

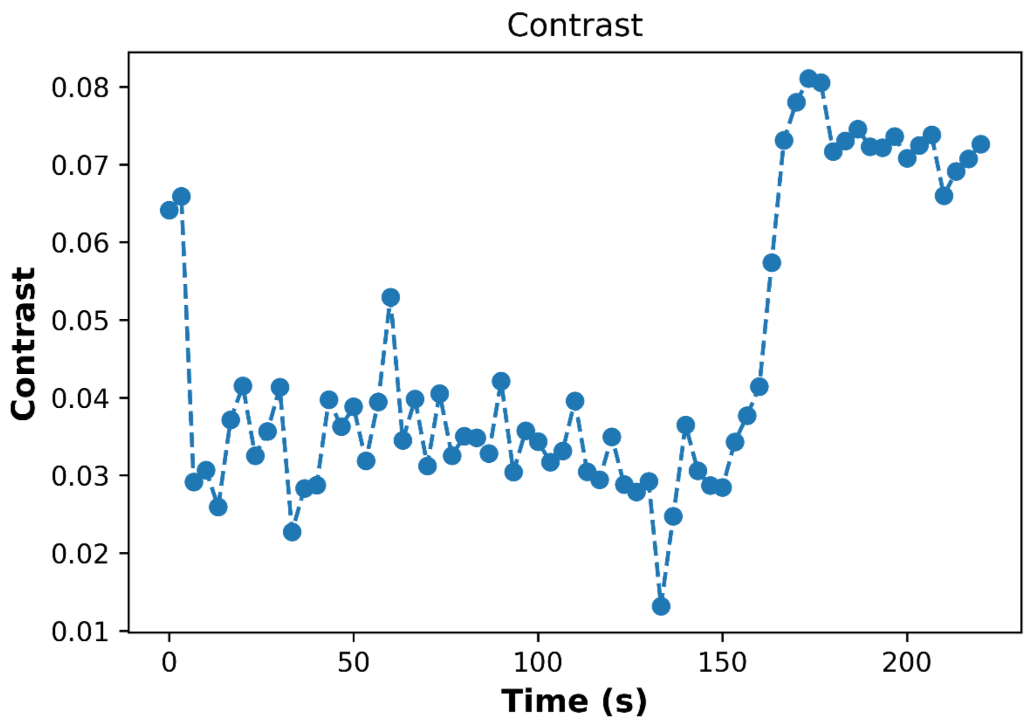

Contrast
Contrast
0.08
0.07
0.06
0.05
0.04
0.03
0.02
0.01
0
50
100
150
200
Time (s)

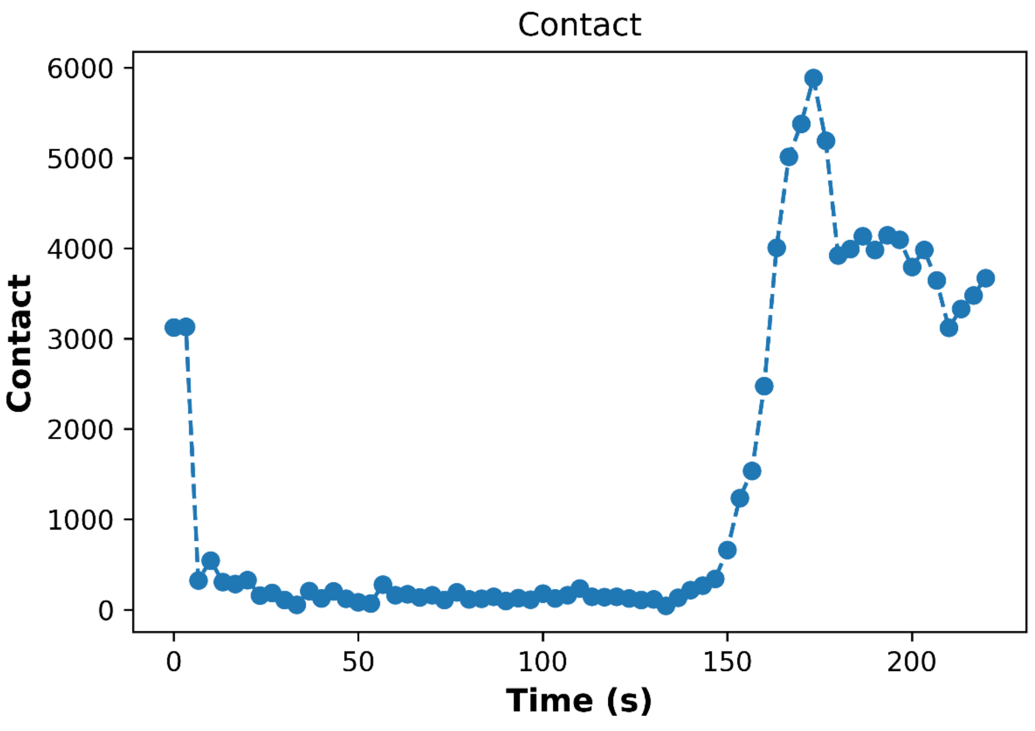

Contact
Contact
6000
5000
4000
3000
2000
1000
0
0
50
100
150
200
Time (s)

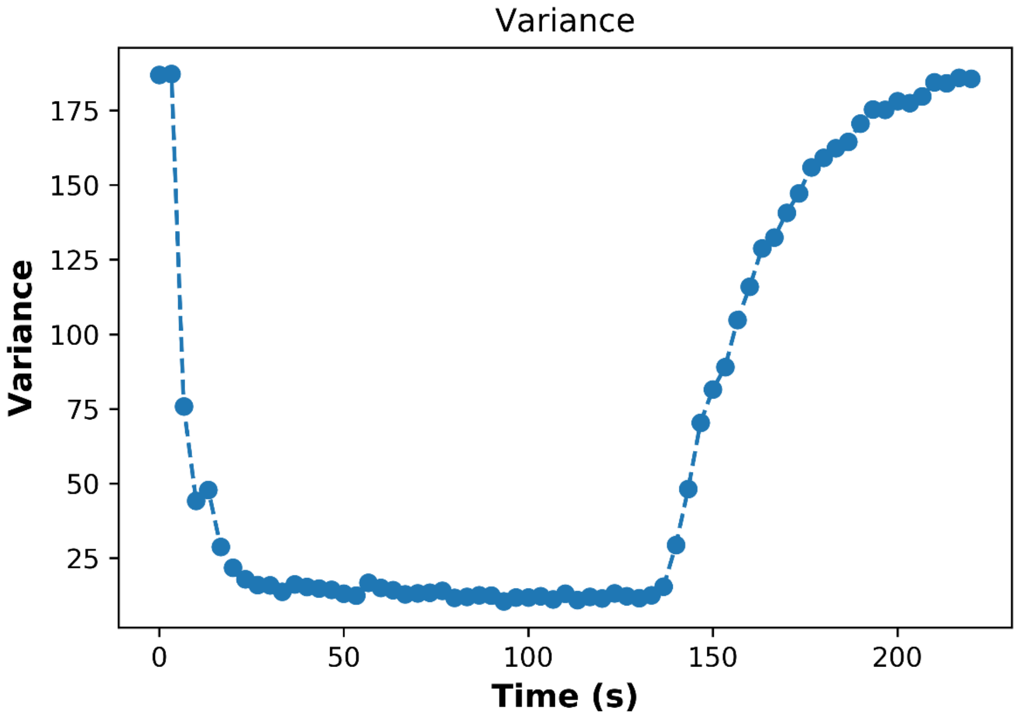
Variance
175
150
125
100
75
50
25
Variance
0
50
100
150
200
Time (s)

Table 1, Entry 5: 100 RPM, Anchor, No baffles

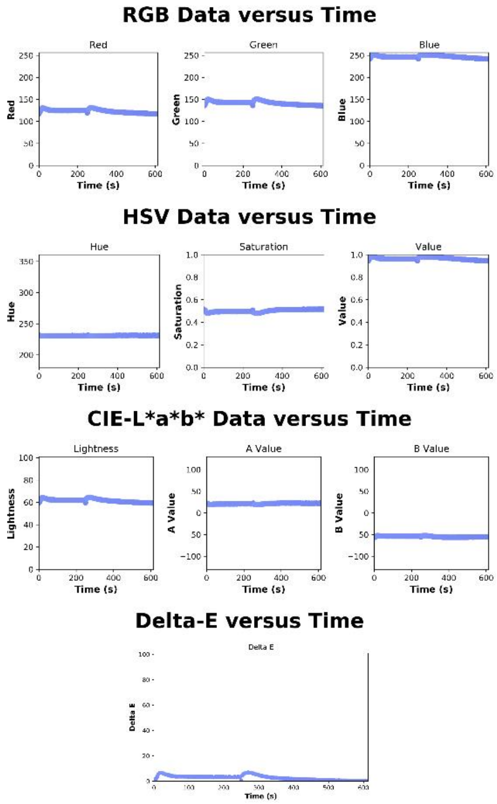

Kineticolor

Selected Region of Interest

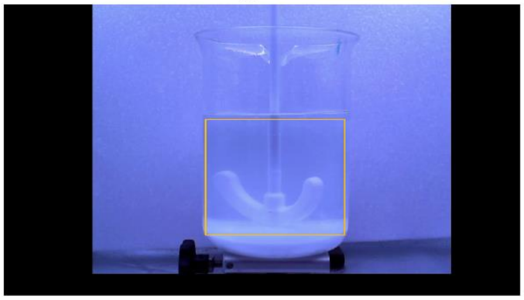

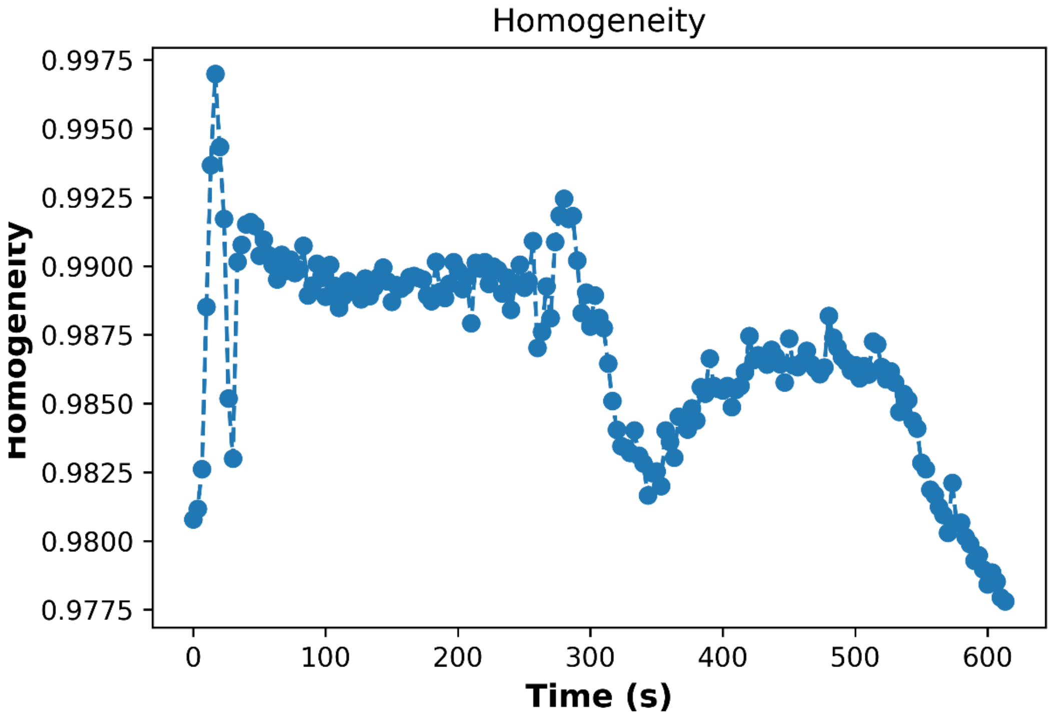
Homogeneity
Homogeneity
Time (s)
0.9975
0.9950
0.9925
0.9900
0.9875
0.9850
0.9825
0.9800
0.9775
0
100
200
300
400
500
600

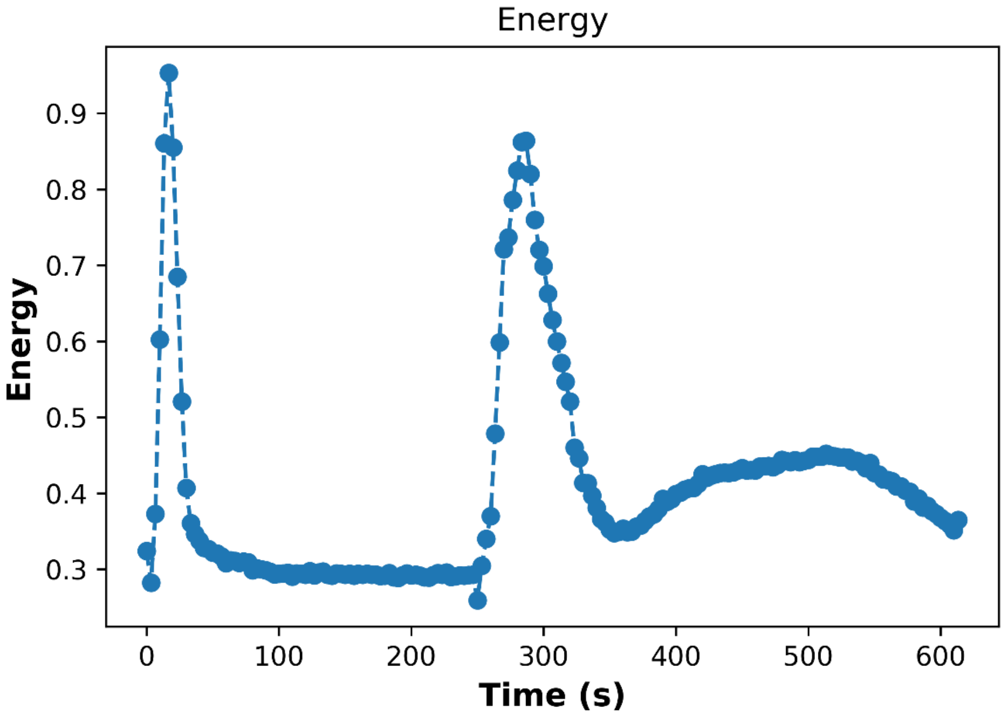
Energy
Energy
Time (s)
0.9
0.8
0.7
0.6
0.5
0.4
0.3
0
100
200
300
400
500
600

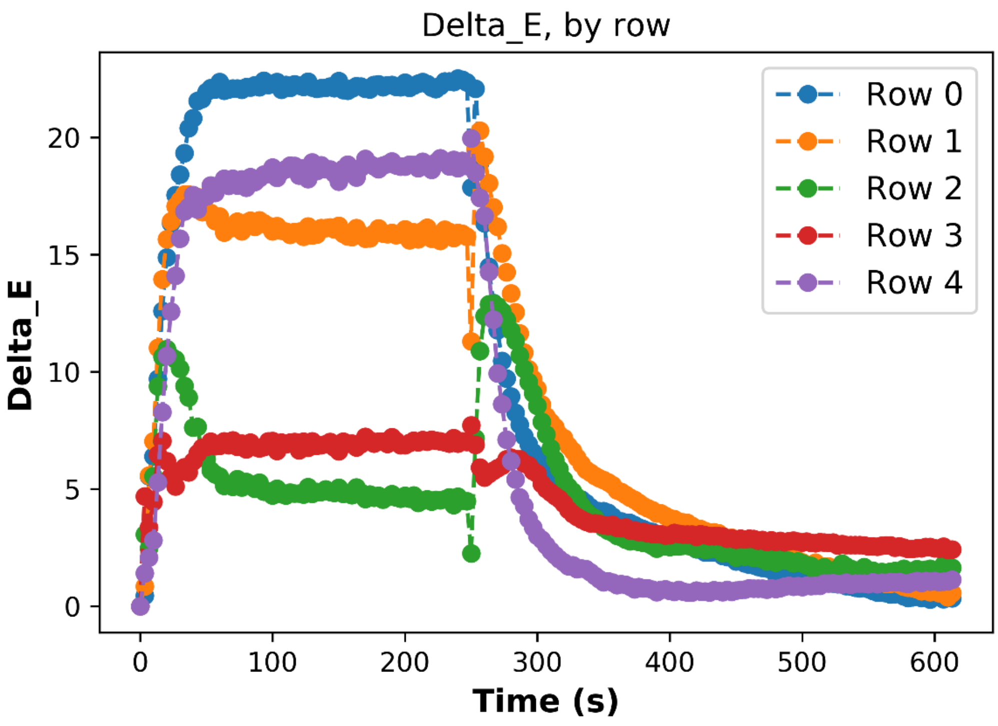
Delta_E, by row
Row 0
Row 1
Row 2
Row 3
Row 4
Delta_E
Time (s)
0
5
10
15
20
0
100
200
300
400
500
600

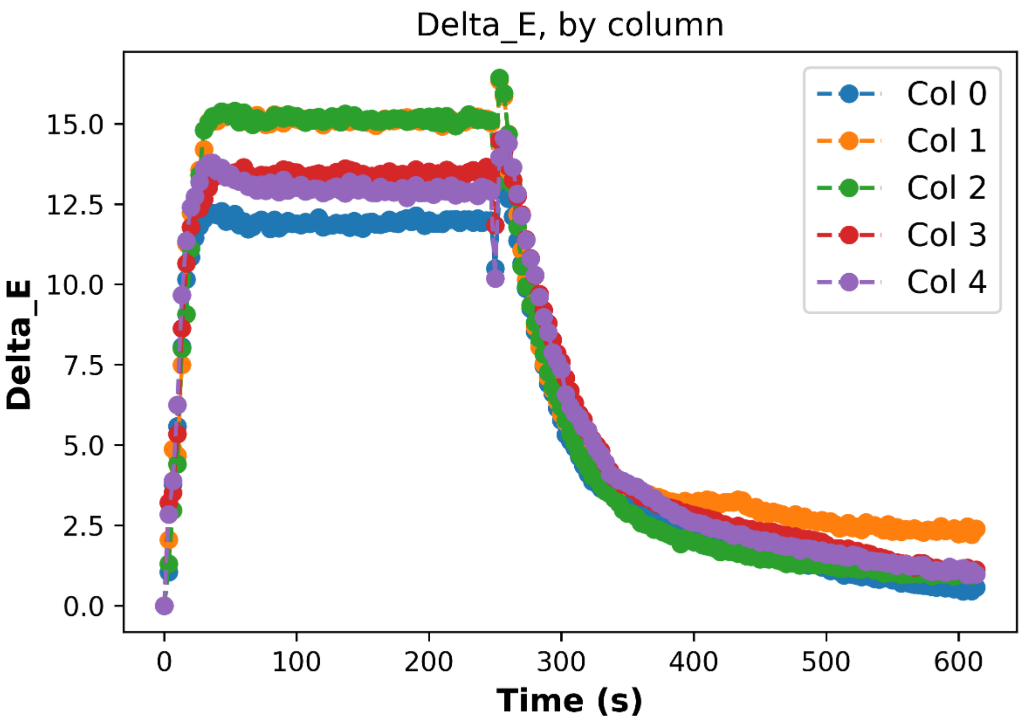
Delta_E, by column
Col 0
Col 1
Col 2
Col 3
Col 4
Delta_E
Time (s)
0.0
2.5
5.0
7.5
10.0
12.5
15.0
0
100
200
300
400
500
600

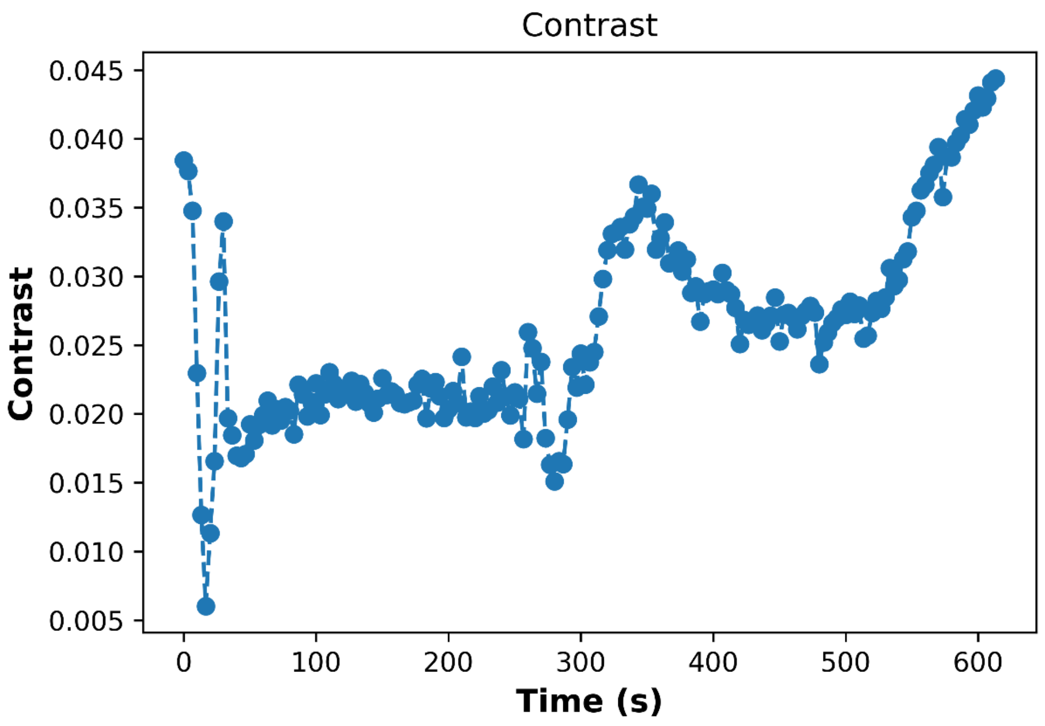
Contrast
0.045
0.040
0.035
0.030
0.025
0.020
0.015
0.010
0.005
Contrast
0
100
200
300
400
500
600
Time (s)

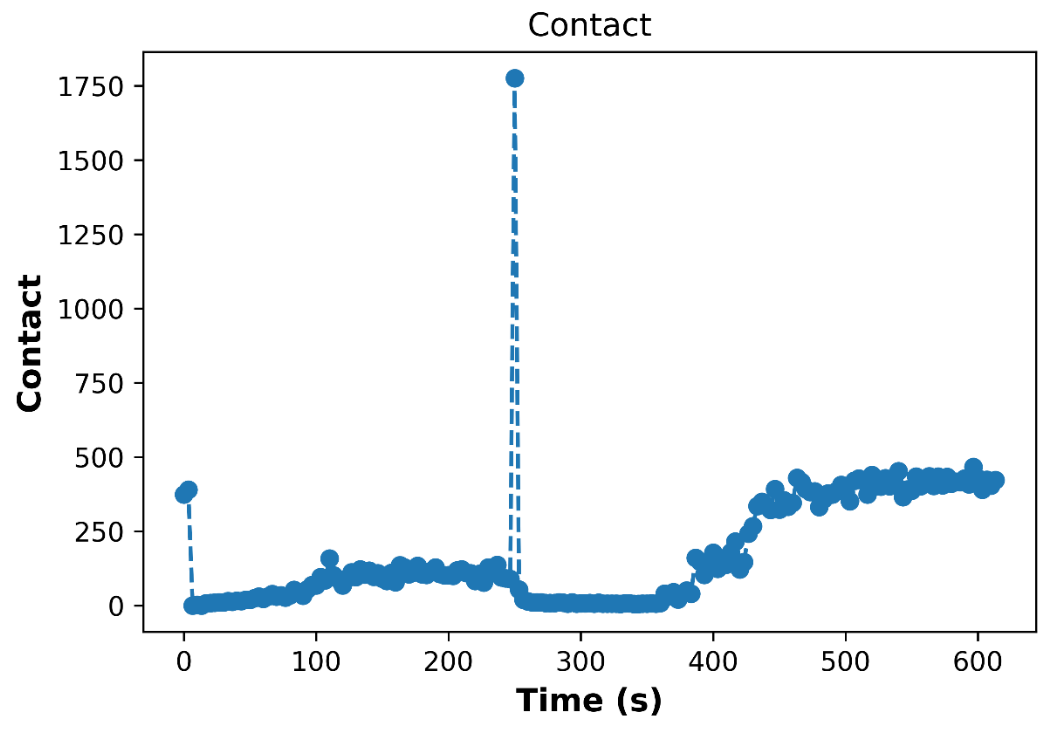
Contact
1750
1500
1250
1000
750
500
250
0
Contact
0
100
200
300
400
500
600
Time (s)

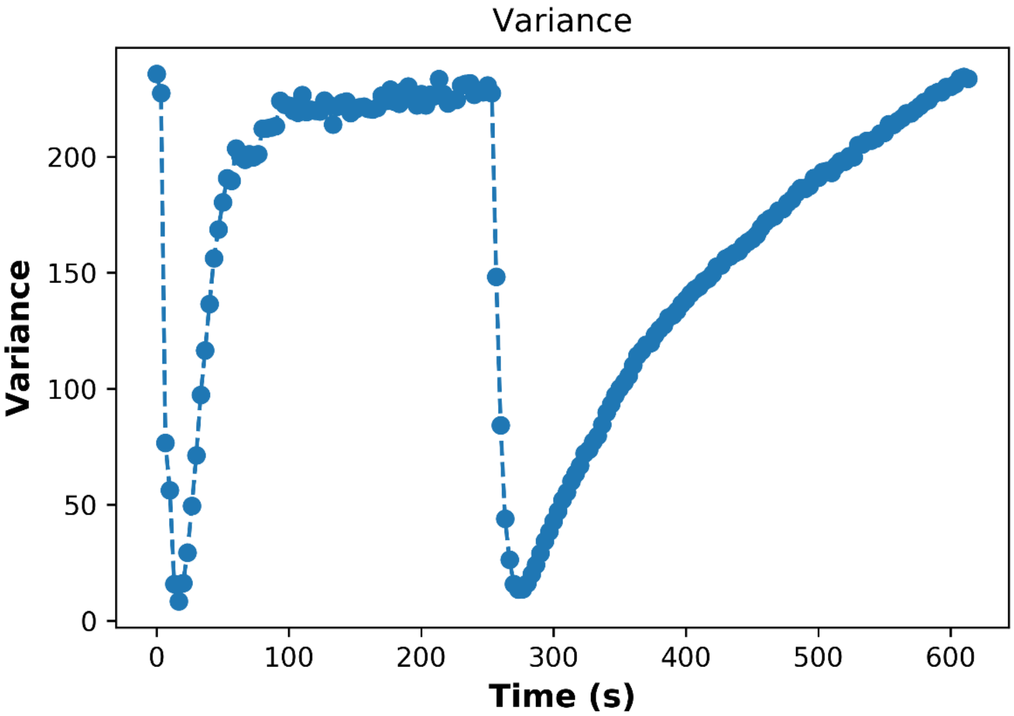

Variance
Variance
0
50
100
150
200
0
100
200
300
400
500
600
Time (s)

Table 1, Entry 5: 100 RPM, Anchor, No baffles, with probe

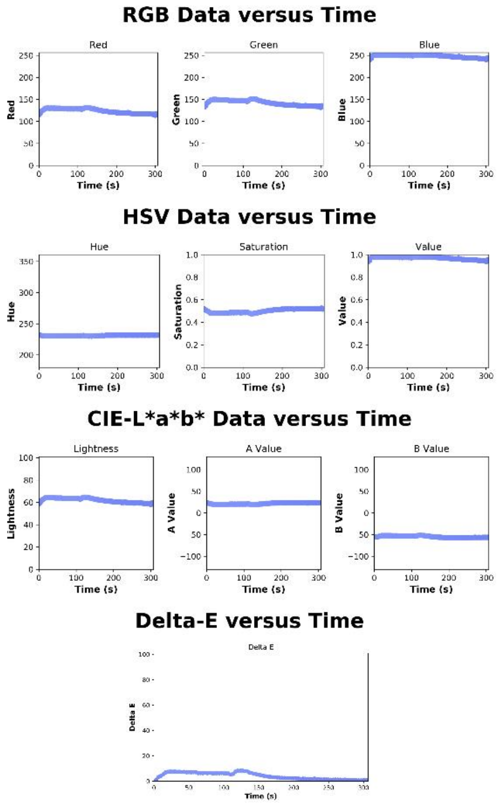

Kineticolor

Selected Region of Interest

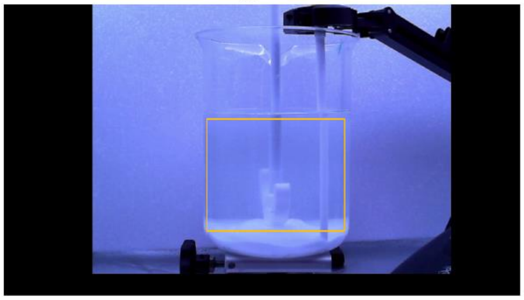

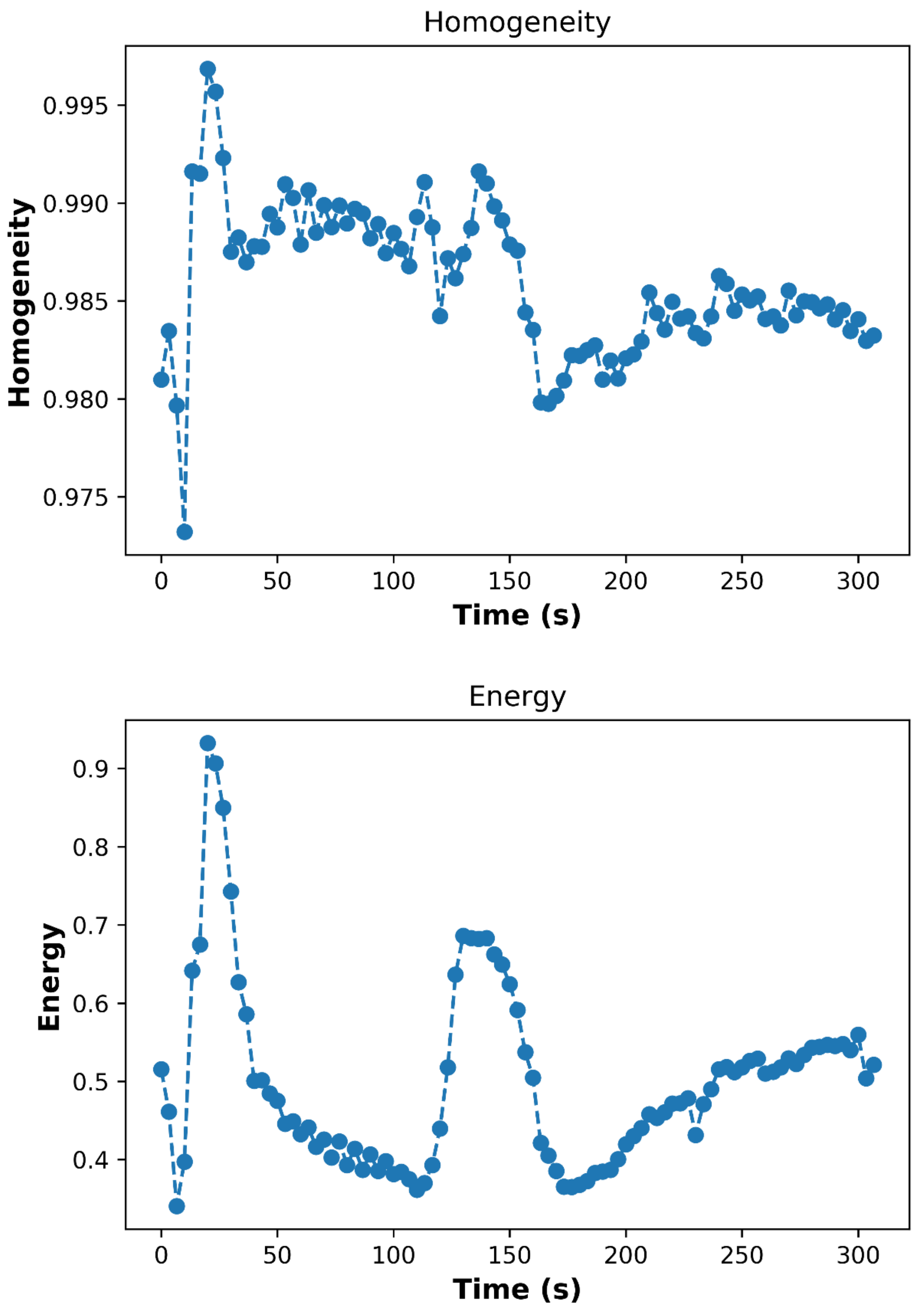
Homogeneity
0.995
0.990
0.985
0.980
0.975
Homogeneity
0
50
100
150
200
250
300
Time (s)
Energy
0.9
0.8
0.7
0.6
0.5
0.4
Energy
0
50
100
150
200
250
300
Time (s)

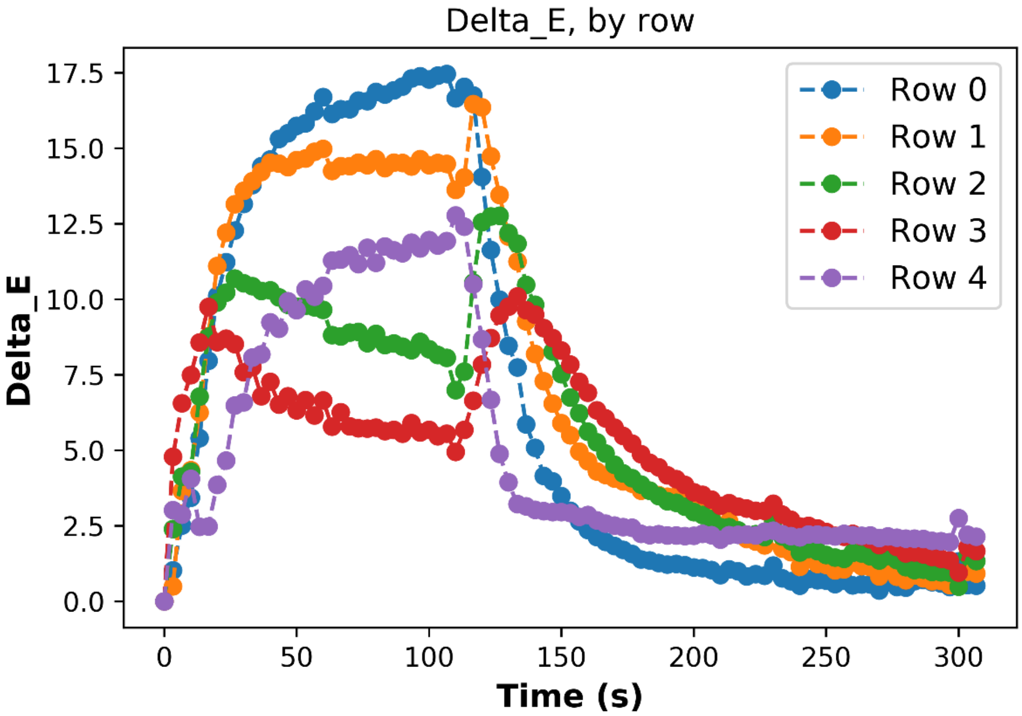

Delta_E, by row
Row 0
Row 1
Row 2
Row 3
Row 4
Delta_E
Time (s)

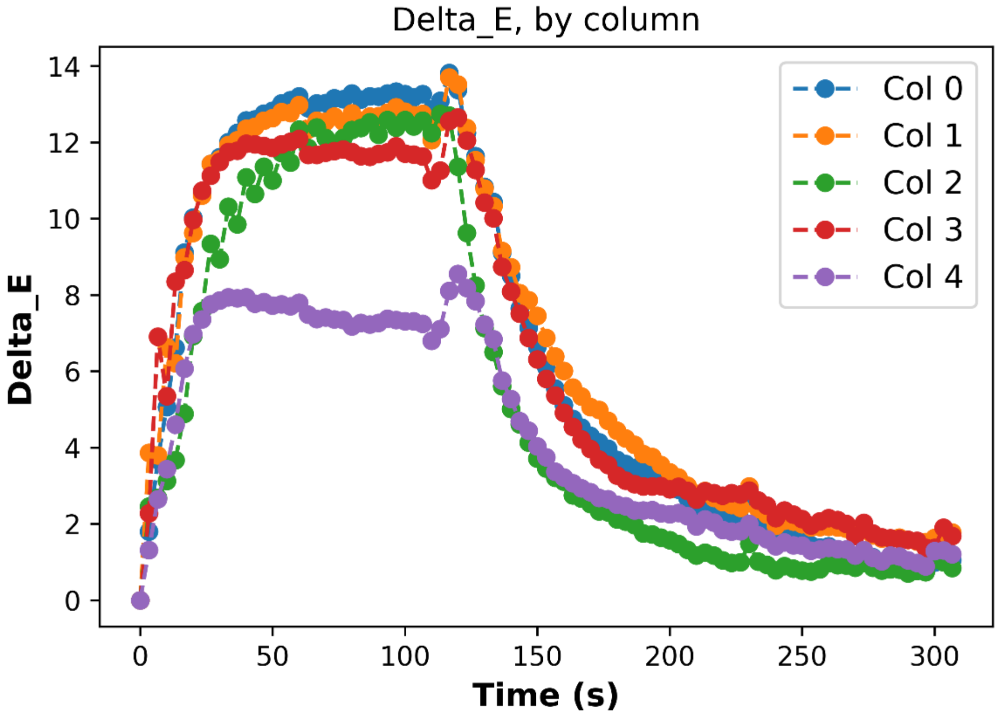

Delta_E, by column
Col 0
Col 1
Col 2
Col 3
Col 4
Delta_E
Time (s)

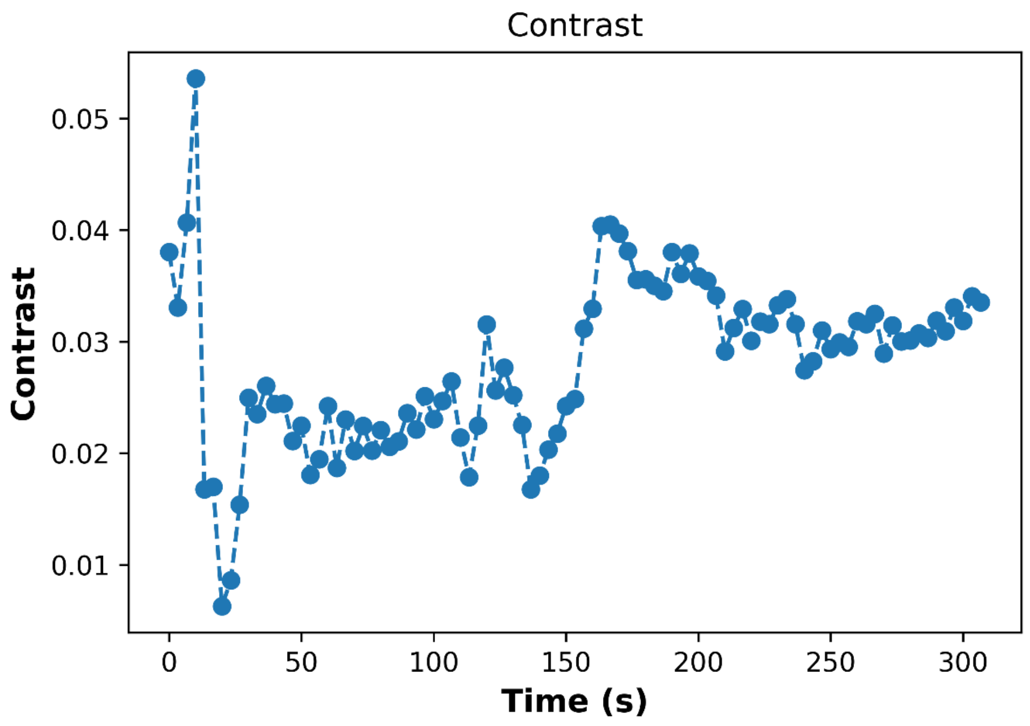

Contrast
0.05
0.04
0.03
0.02
0.01
Contrast
0
50
100
150
200
250
300
Time (s)

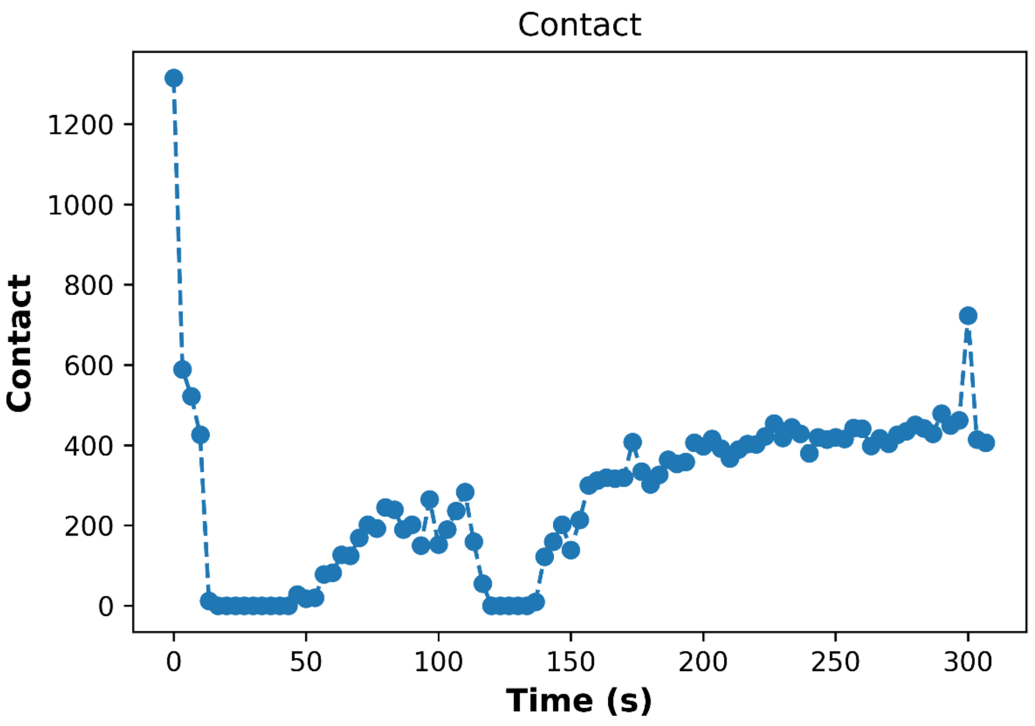

Contact
1200
1000
800
600
400
200
0
Contact
0
50
100
150
200
250
300
Time (s)

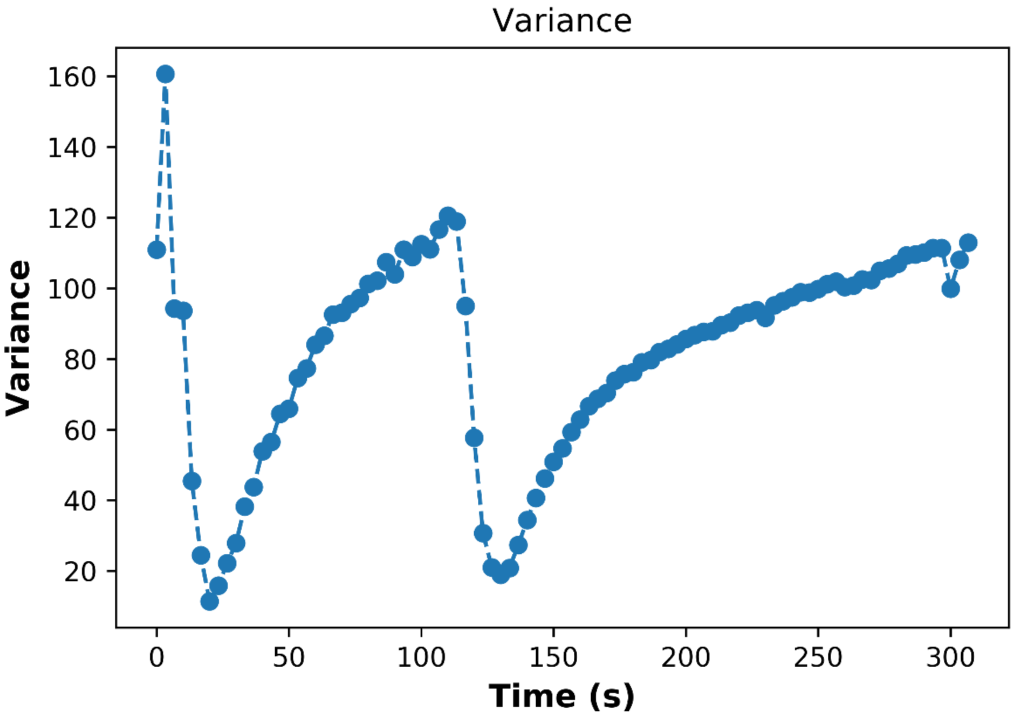
Variance
160
140
120
100
80
60
40
20
Variance
0
50
100
150
200
250
300
Time (s)

Table 1, Entry 6: 210 RPM, Anchor, No baffles

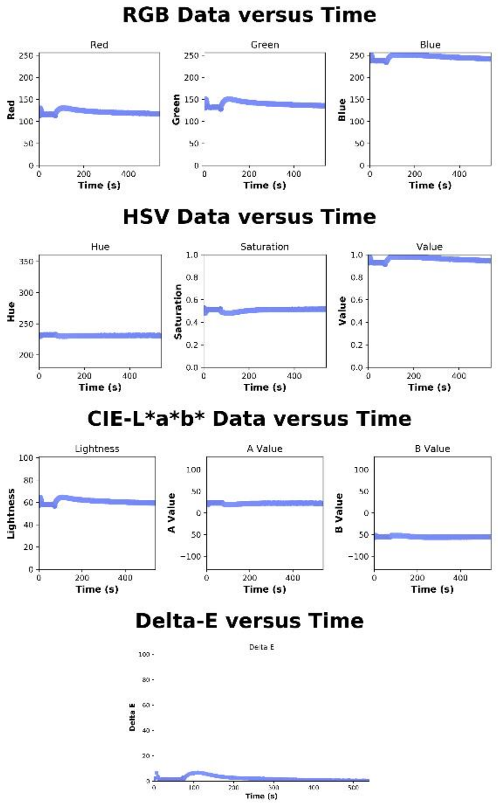

Kineticolor

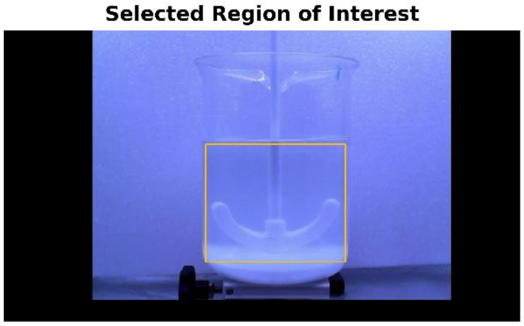

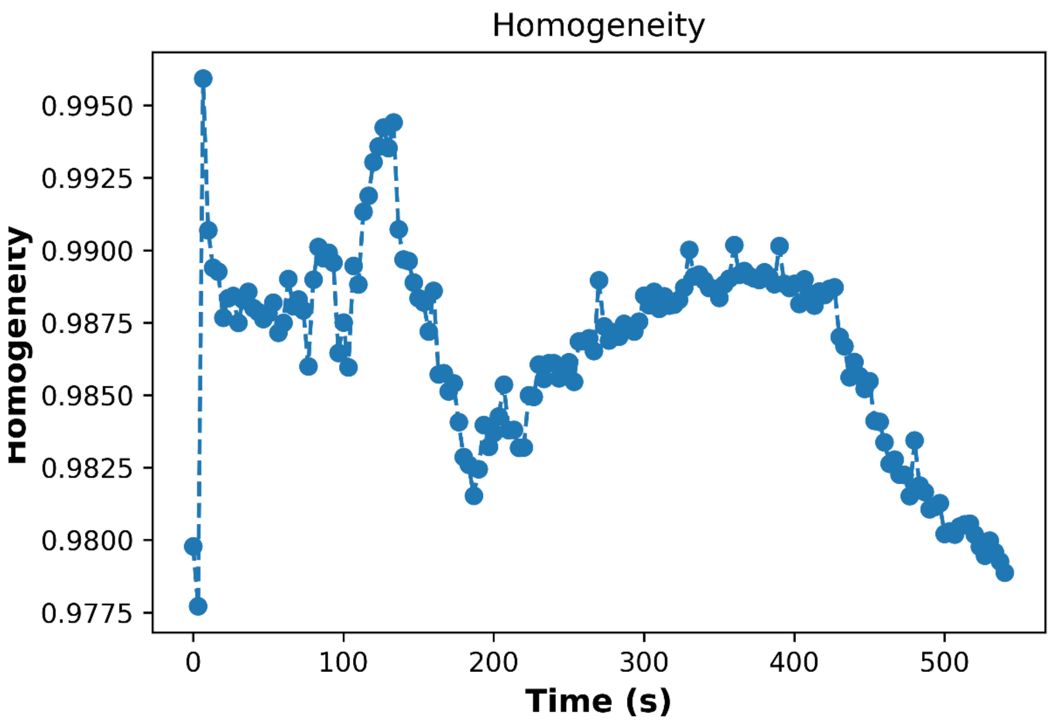
Homogeneity
0.9950
0.9925
0.9900
0.9875
0.9850
0.9825
0.9800
0.9775
Homogeneity
0
100
200
300
400
500
Time (s)

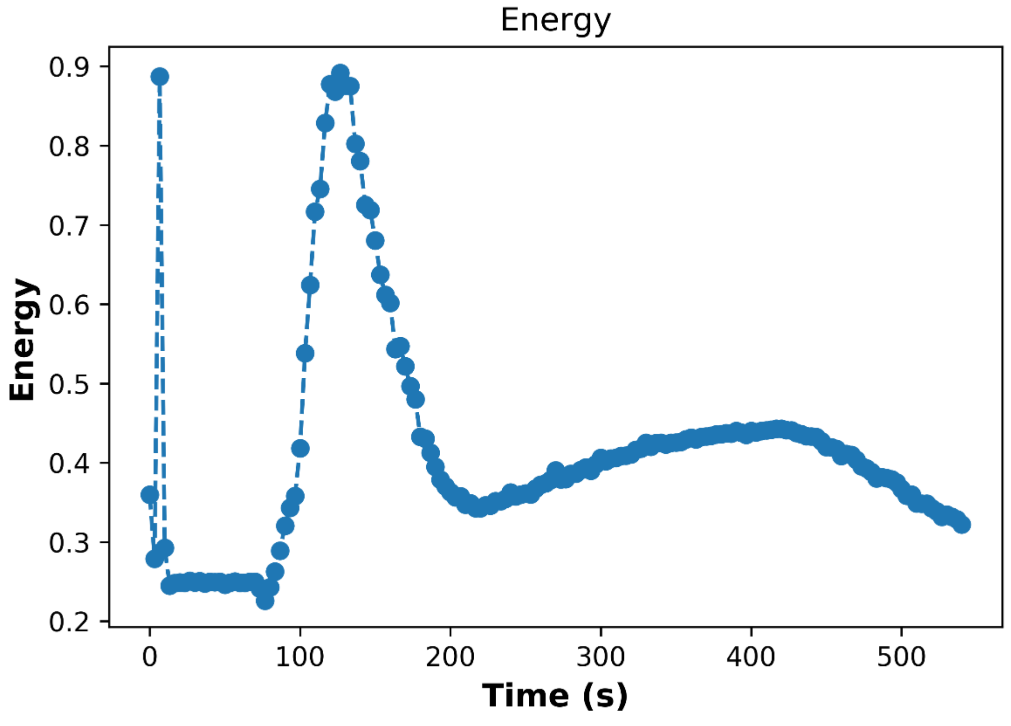
Energy
0.9
0.8
0.7
0.6
0.5
0.4
0.3
0.2
Energy
0
100
200
300
400
500
Time (s)

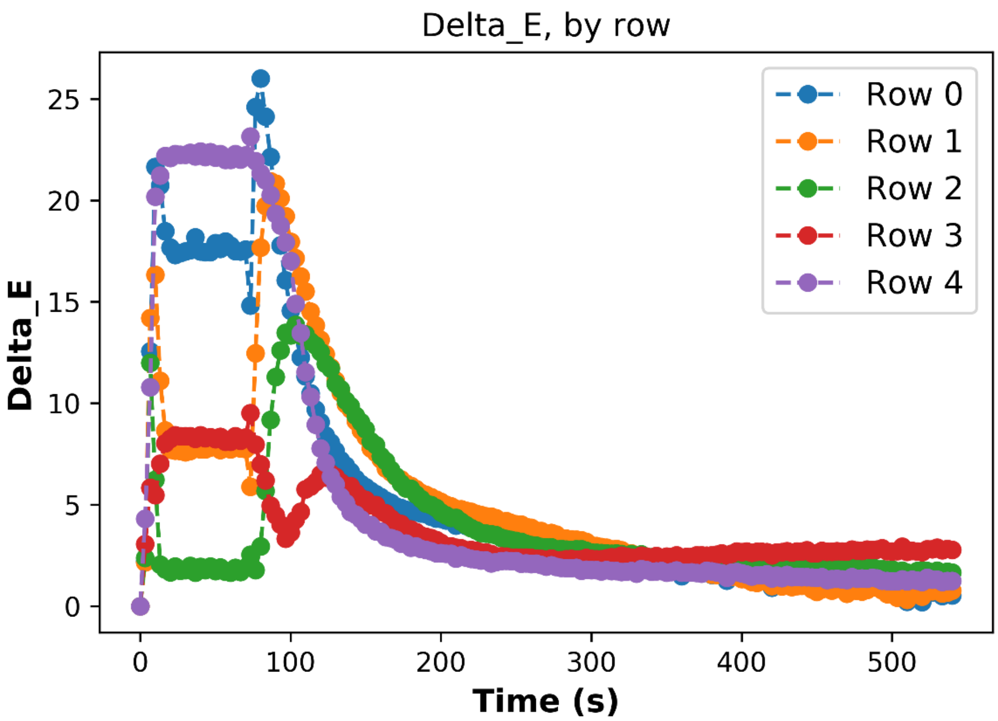
Delta_E, by row
Row 0
Row 1
Row 2
Row 3
Row 4
Delta_E
Time (s)
0
5
10
15
20
25
0
100
200
300
400
500

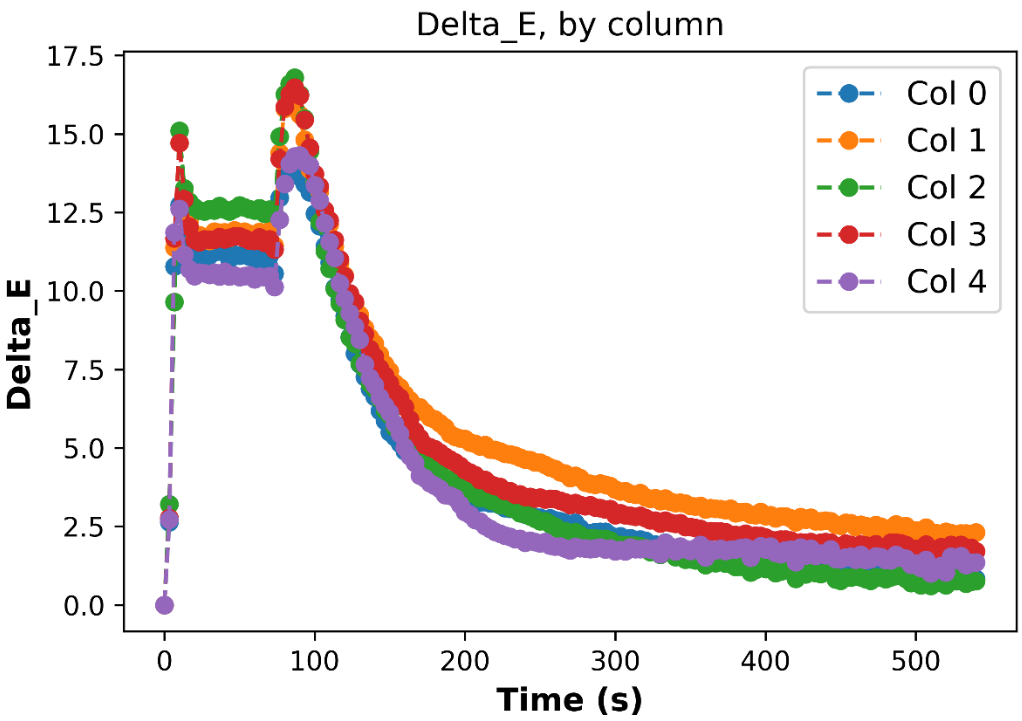
Delta_E, by column
Col 0
Col 1
Col 2
Col 3
Col 4
Delta_E
Time (s)
0.0
2.5
5.0
7.5
10.0
12.5
15.0
17.5
0
100
200
300
400
500

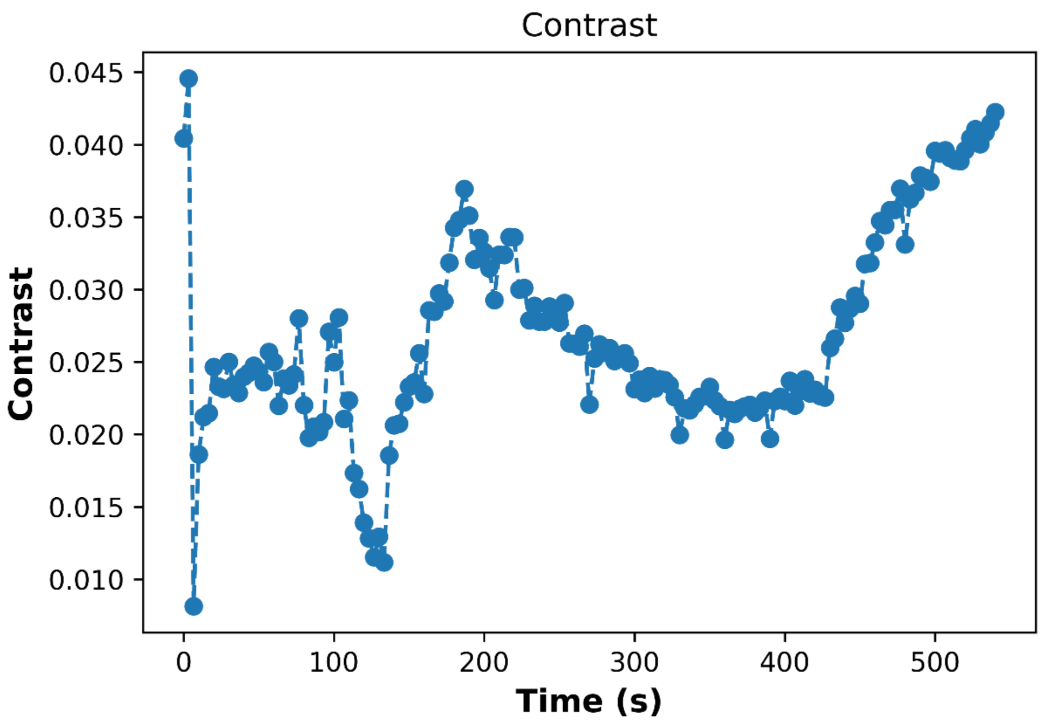

Contrast
0.045
0.040
0.035
0.030
0.025
0.020
0.015
0.010
Contrast
0
100
200
300
400
500
Time (s)

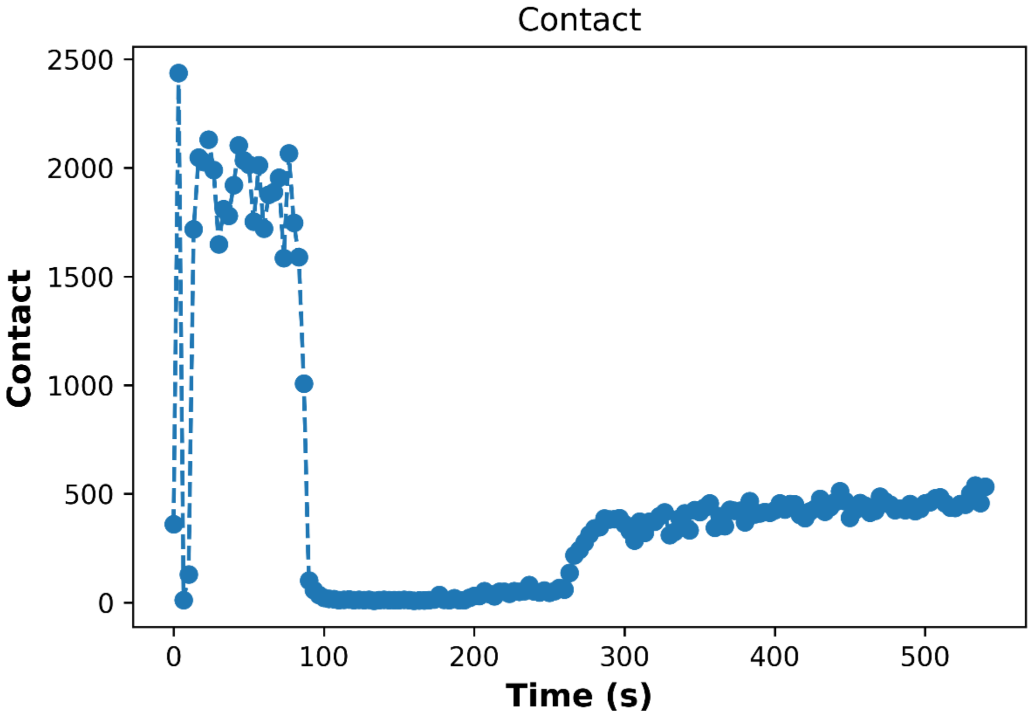

Contact
2500
2000
1500
1000
500
0
Contact
0
100
200
300
400
500
Time (s)

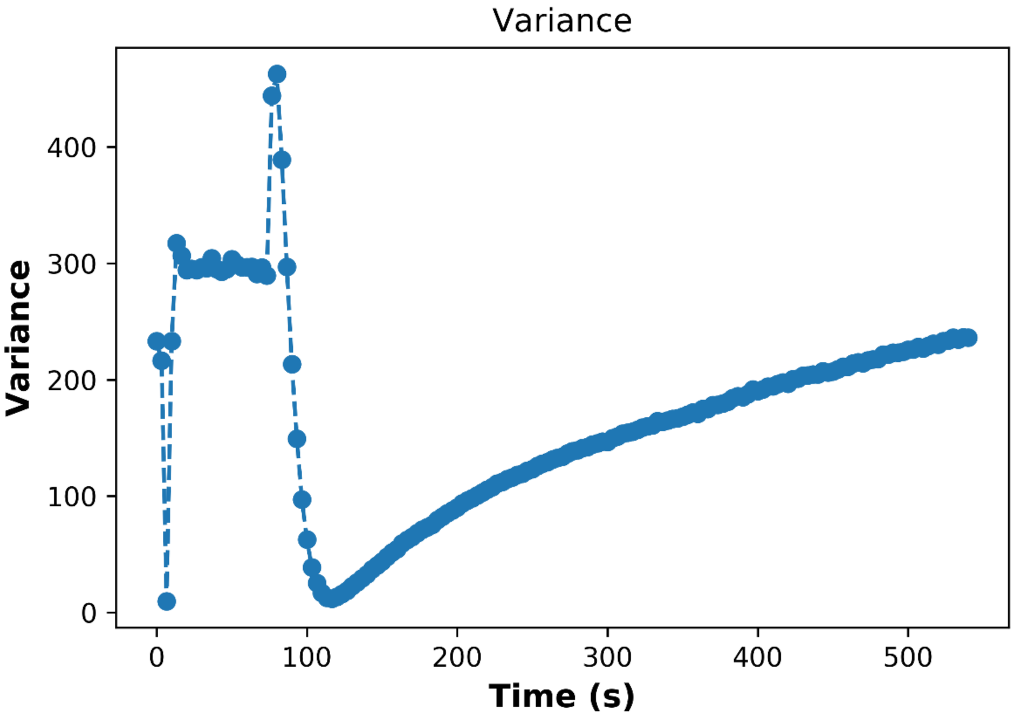

Variance
400
300
200
100
0
0
100
200
300
400
500
Variance
Time (s)

Table 1, Entry 6: 210 RPM, Anchor, No baffles, with probe

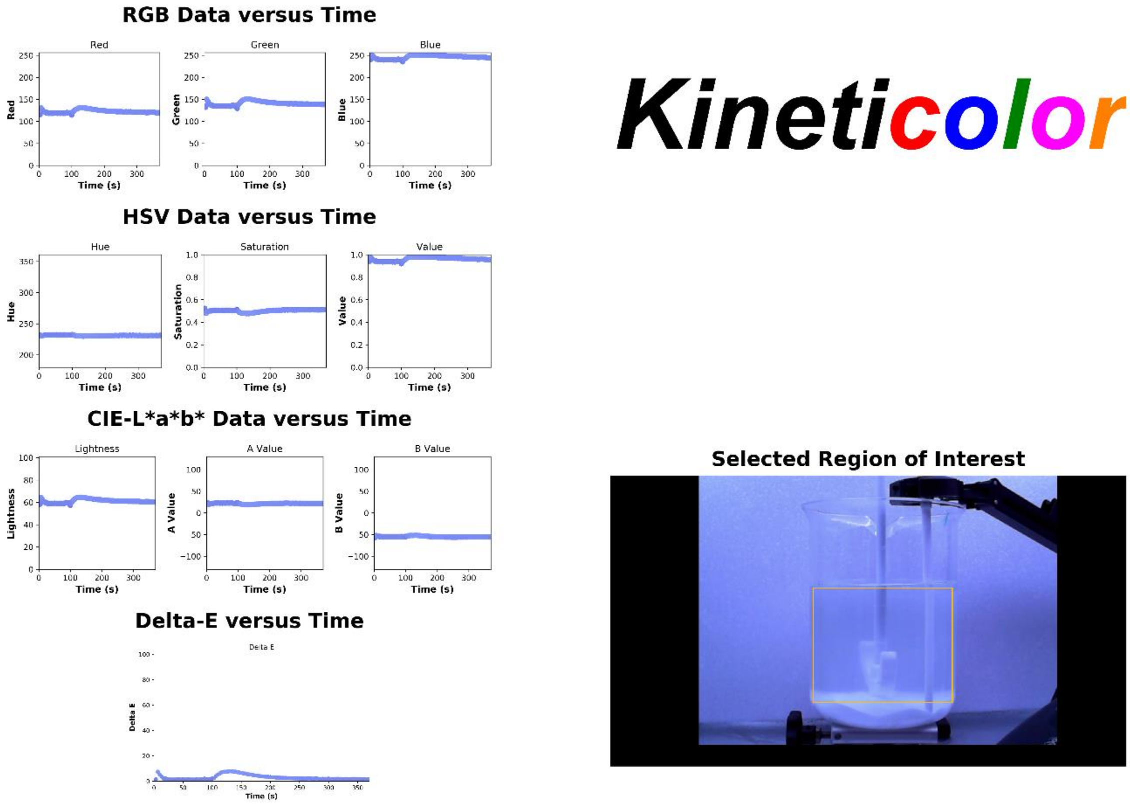

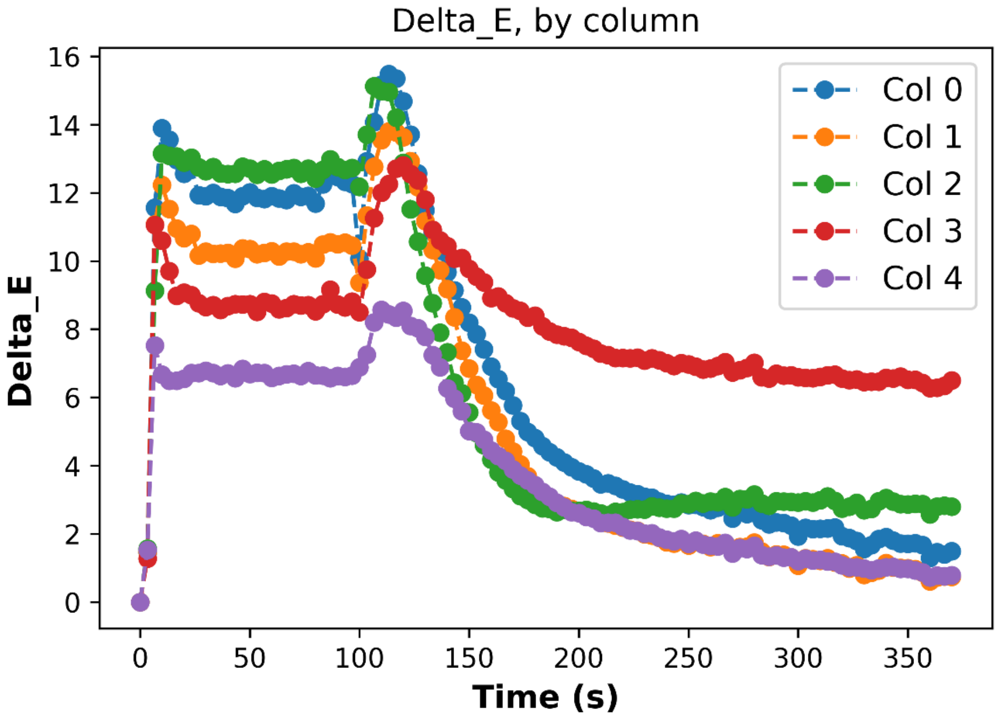

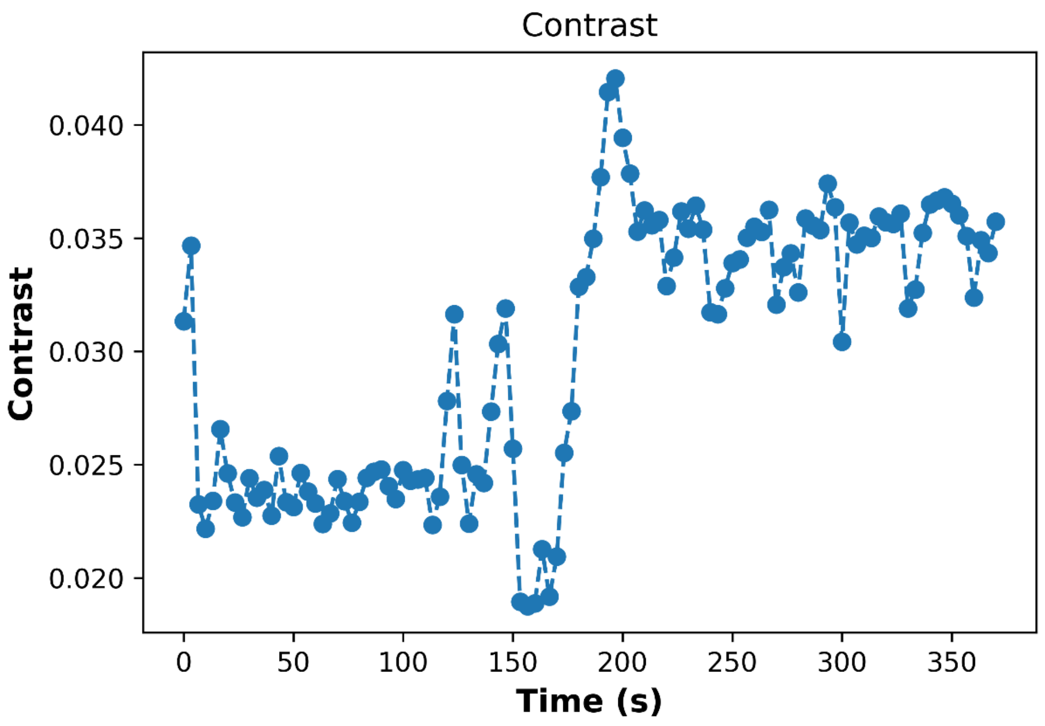
Contrast
Contrast
0.040
0.035
0.030
0.025
0.020
0
50
100
150
200
250
300
350
Time (s)

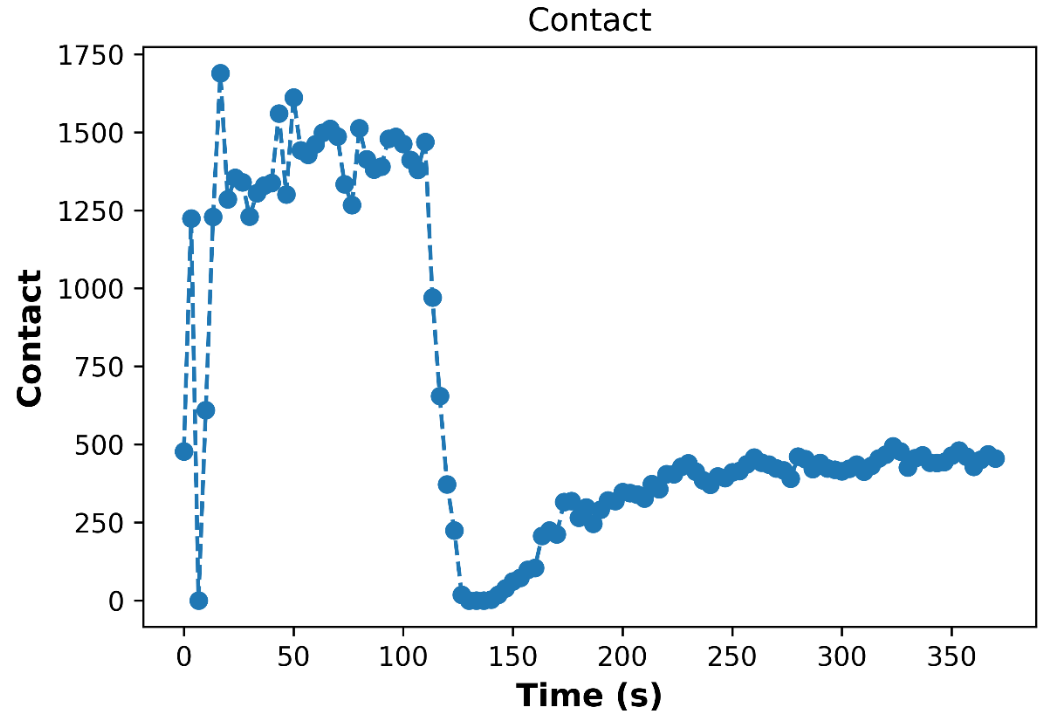
Contact
Contact
1750
1500
1250
1000
750
500
250
0
0
50
100
150
200
250
300
350
Time (s)

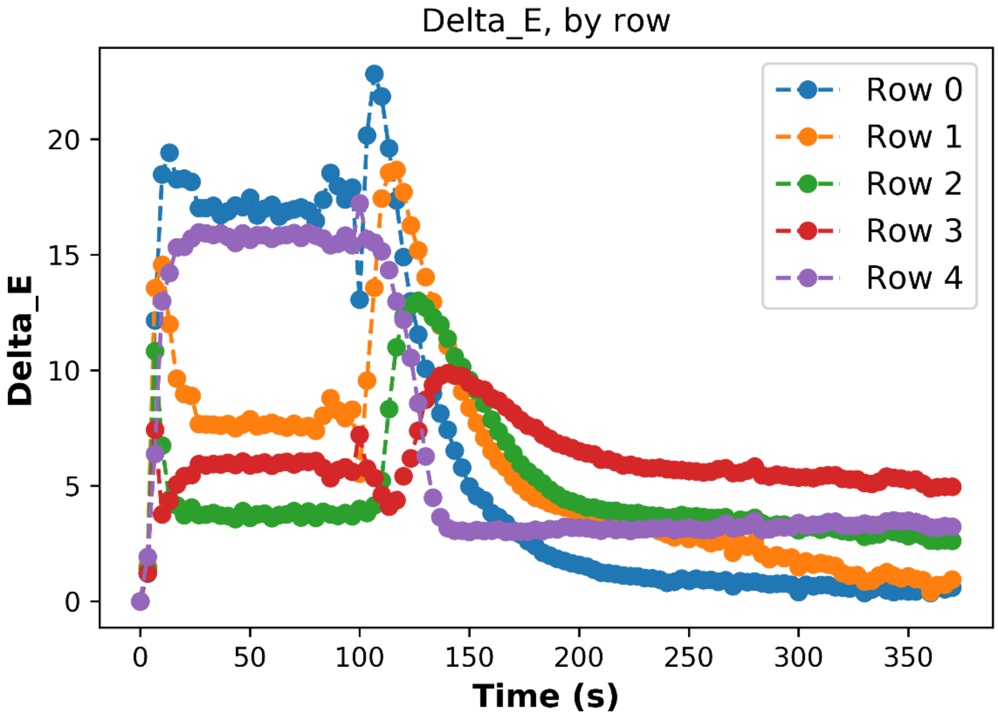
Delta_E, by row
Delta_E
Time (s)
Row 0
Row 1
Row 2
Row 3
Row 4
0
5
10
15
20
0
50
100
150
200
250
300
350

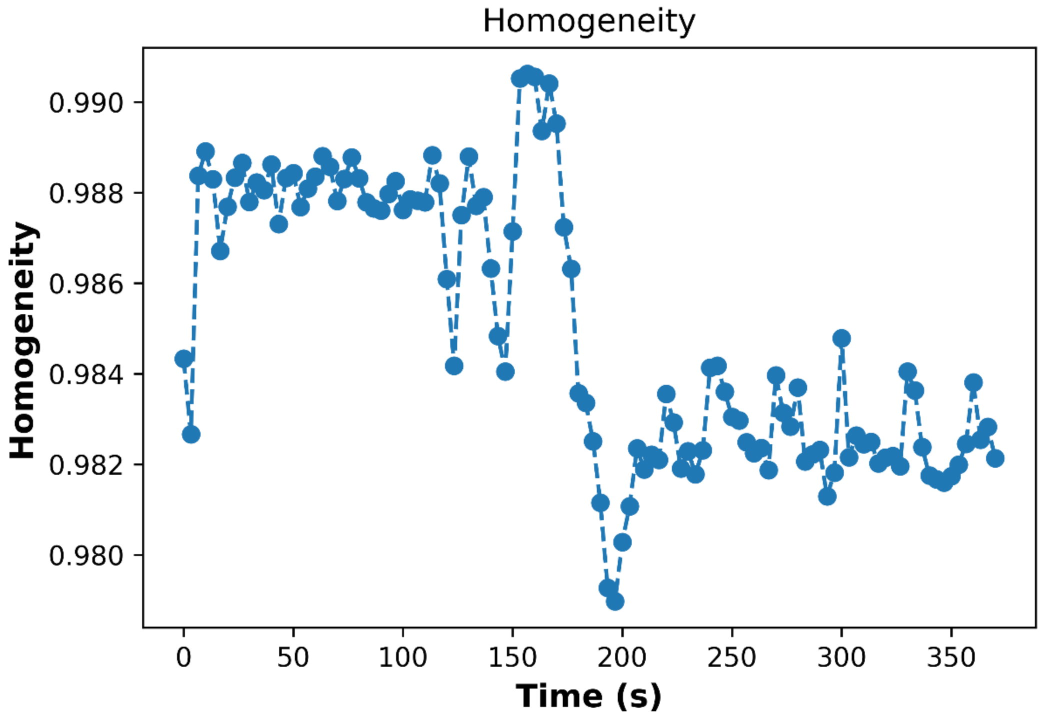

Homogeneity
Homogeneity
0.990
0.988
0.986
0.984
0.982
0.980
0
50
100
150
200
250
300
350
Time (s)

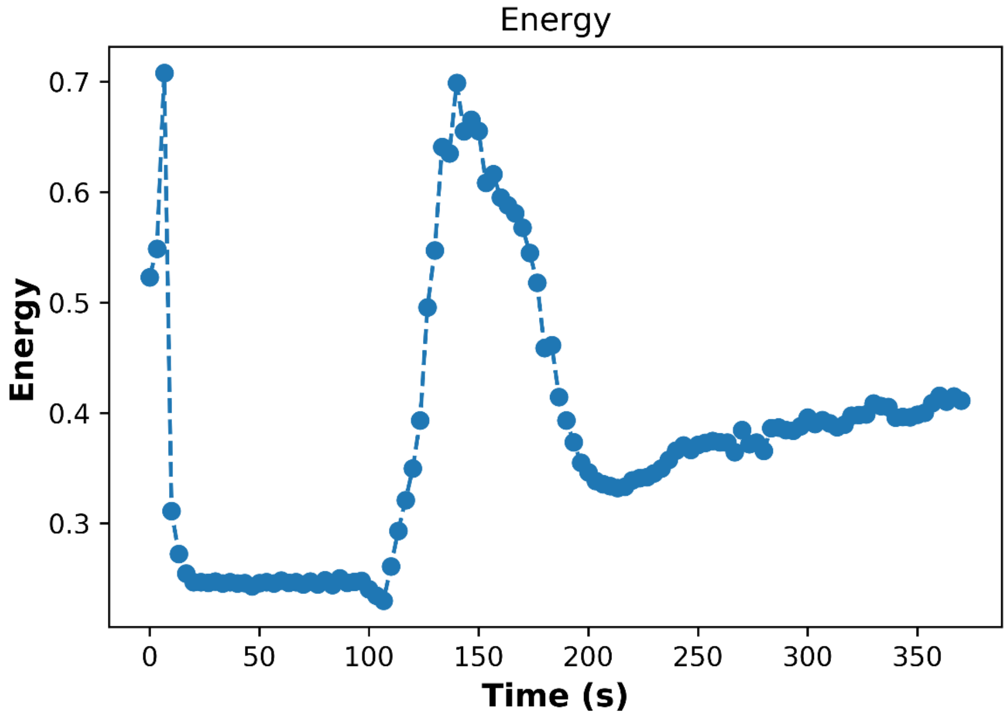

Energy
Energy
0.7
0.6
0.5
0.4
0.3
0
50
100
150
200
250
300
350
Time (s)

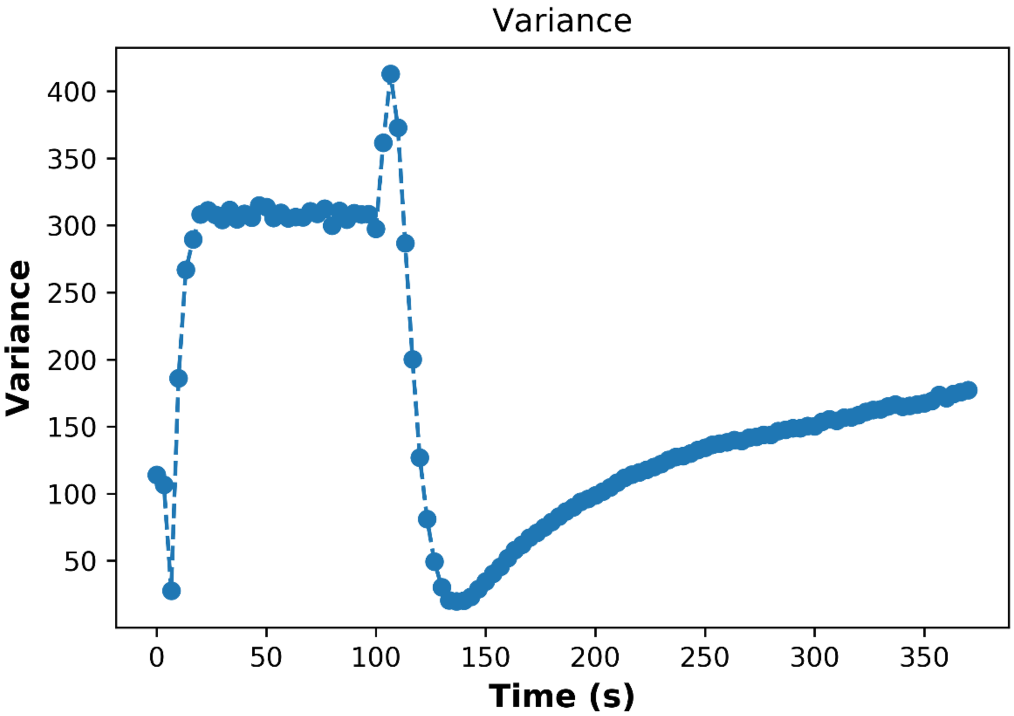
Variance
400
350
300
250
200
150
100
50
Variance
0
50
100
150
200
250
300
350
Time (s)

Table 1, Entry 7: 60 RPM, paddle, with baffle

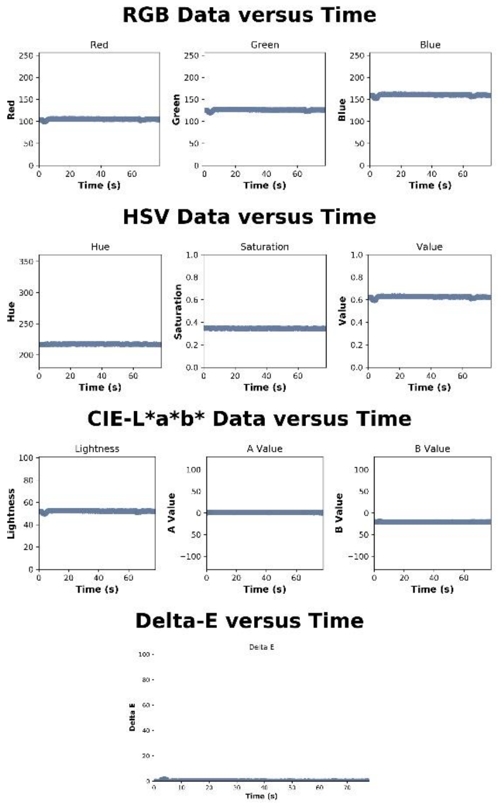

Kineticolor

Selected Region of Interest

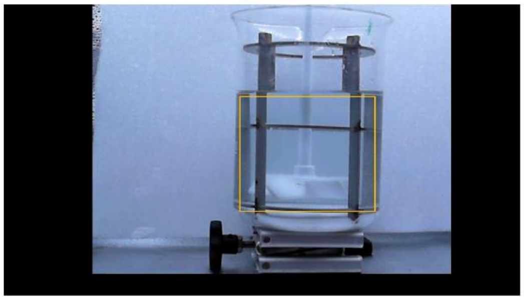

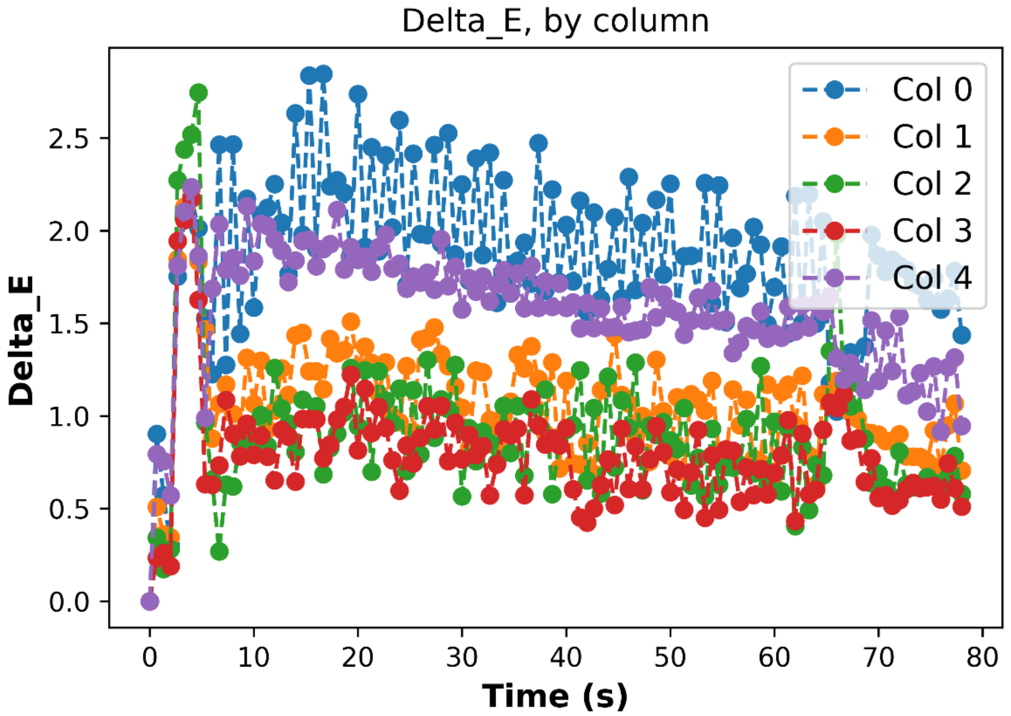
Delta_E, by column
Delta_E
Time (s)
Col 0
Col 1
Col 2
Col 3
Col 4
0.0
0.5
1.0
1.5
2.0
2.5
0
10
20
30
40
50
60
70
80

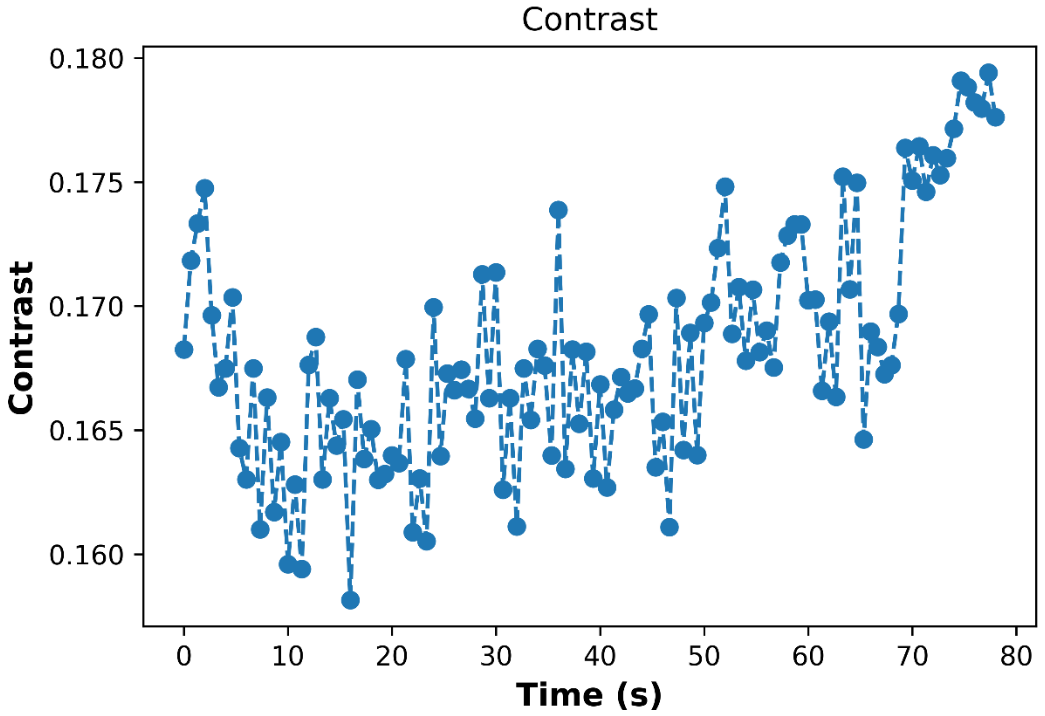
Contrast
Contrast
Time (s)
0.160
0.165
0.170
0.175
0.180
0
10
20
30
40
50
60
70
80

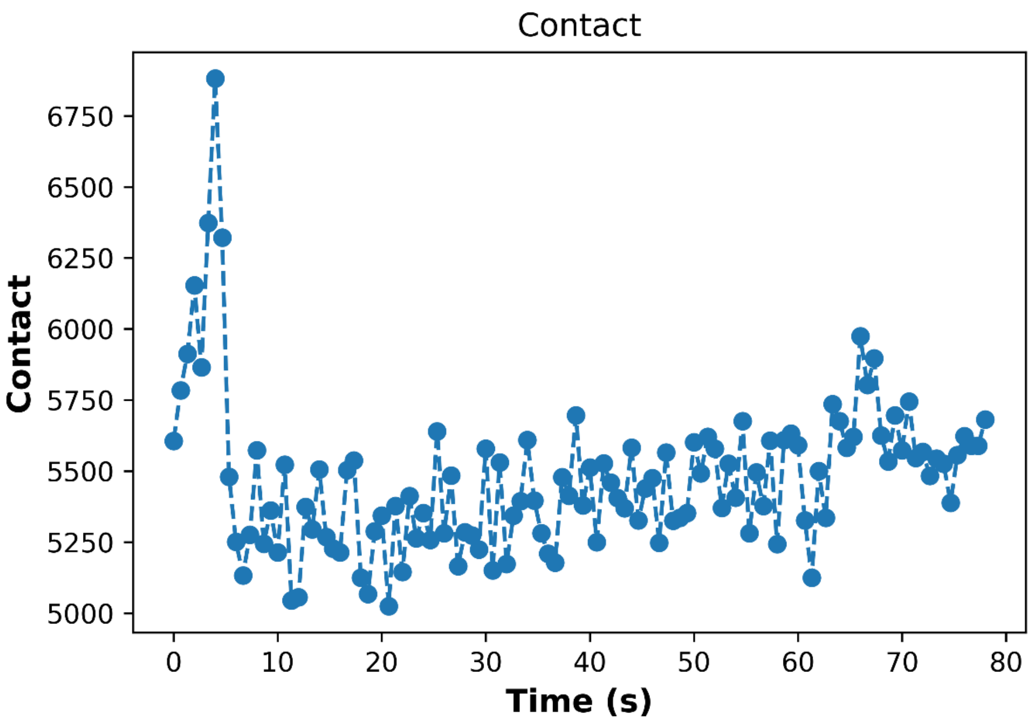
Contact
6750
6500
6250
6000
5750
5500
5250
5000
Contact
0
10
20
30
40
50
60
70
80
Time (s)

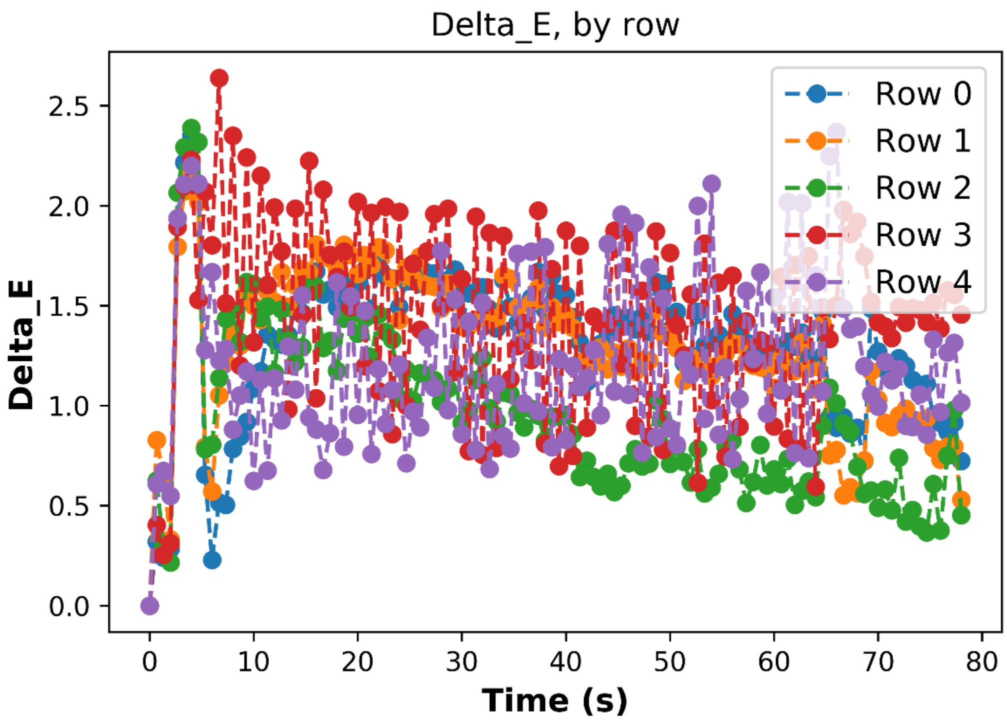
Delta_E, by row
Row 0
Row 1
Row 2
Row 3
Row 4
2.5
2.0
1.5
1.0
0.5
0.0
Delta_E
0
10
20
30
40
50
60
70
80
Time (s)

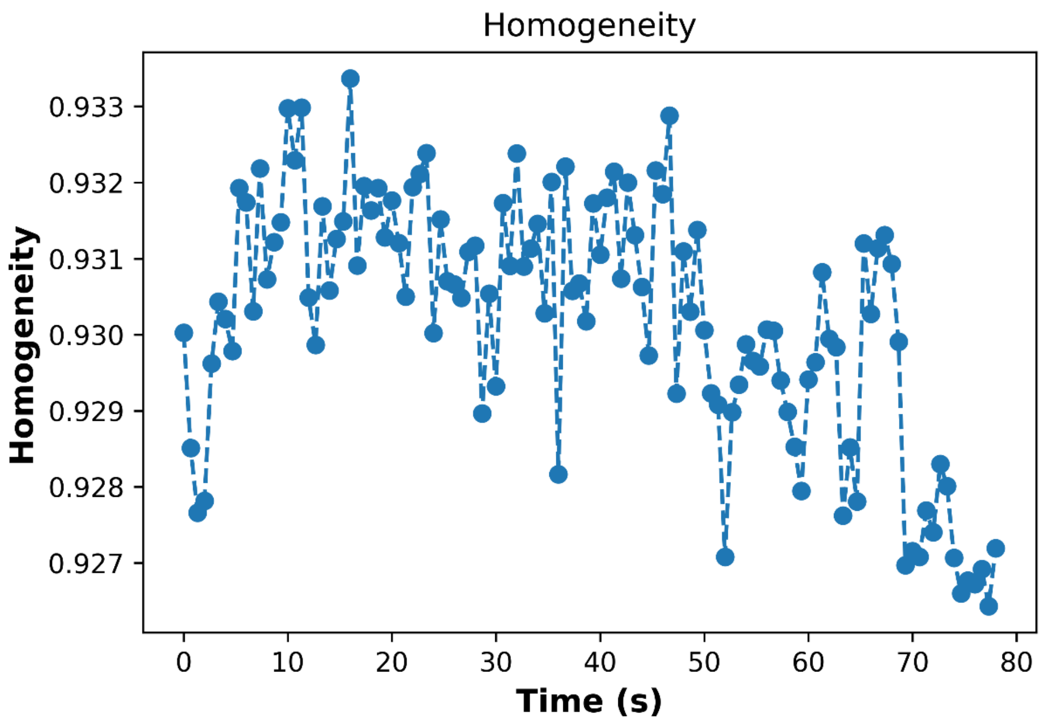

Homogeneity
0.933
0.932
0.931
0.930
0.929
0.928
0.927
Homogeneity
0
10
20
30
40
50
60
70
80
Time (s)

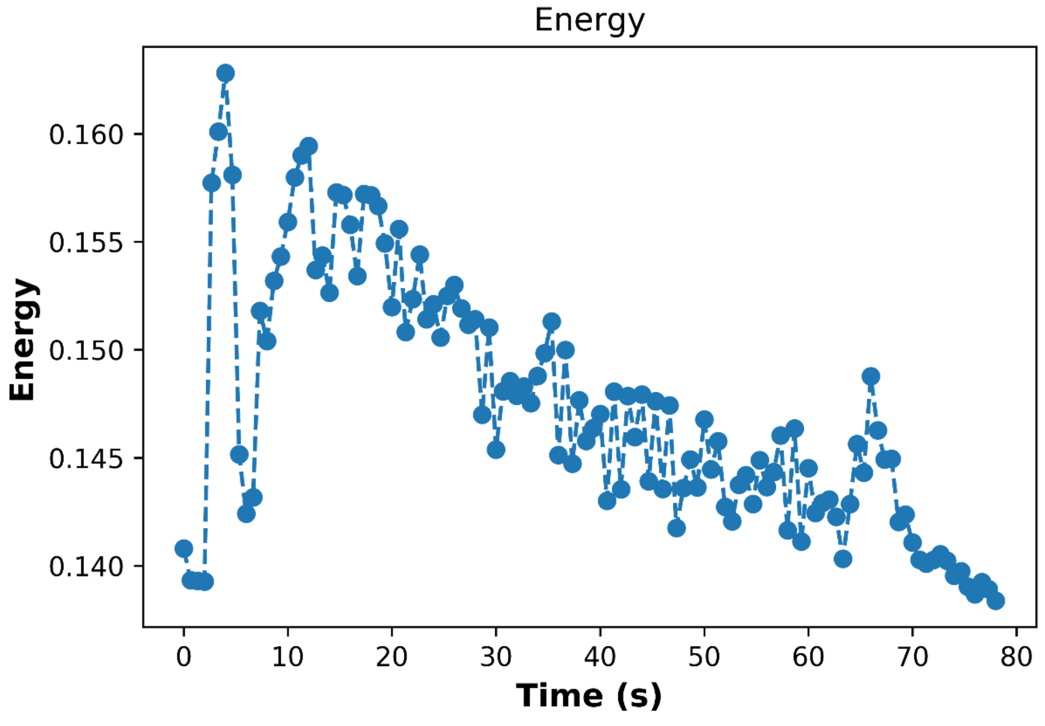

Energy
0.160
0.155
0.150
0.145
0.140
Energy
0
10
20
30
40
50
60
70
80
Time (s)

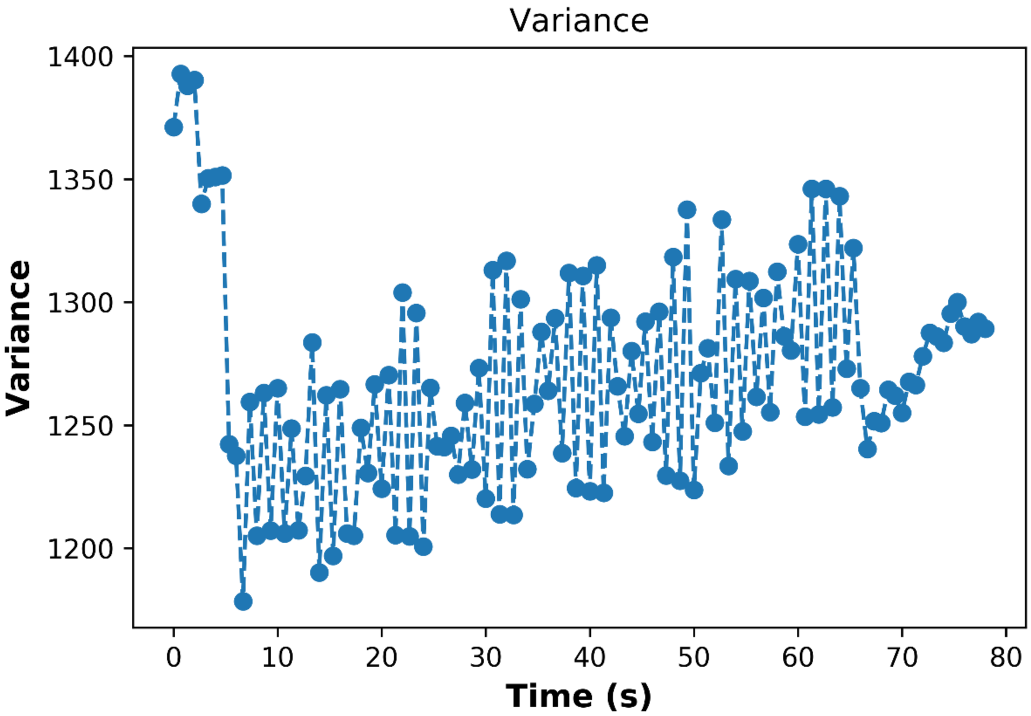

Variance
1400
1350
1300
1250
1200
Variance
0
10
20
30
40
50
60
70
80
Time (s)

Table 1, Entry 8: 100 RPM, paddle, with baffle, no probe

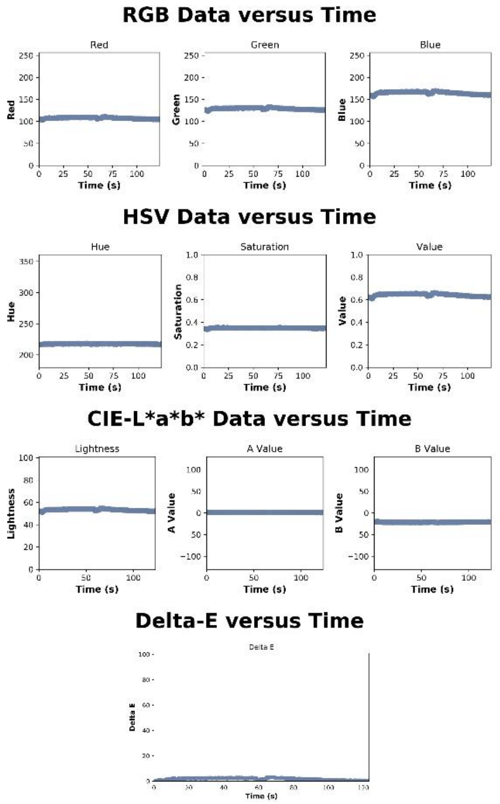

Kineticolor

Selected Region of Interest

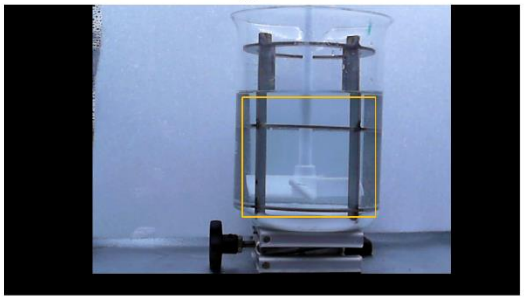

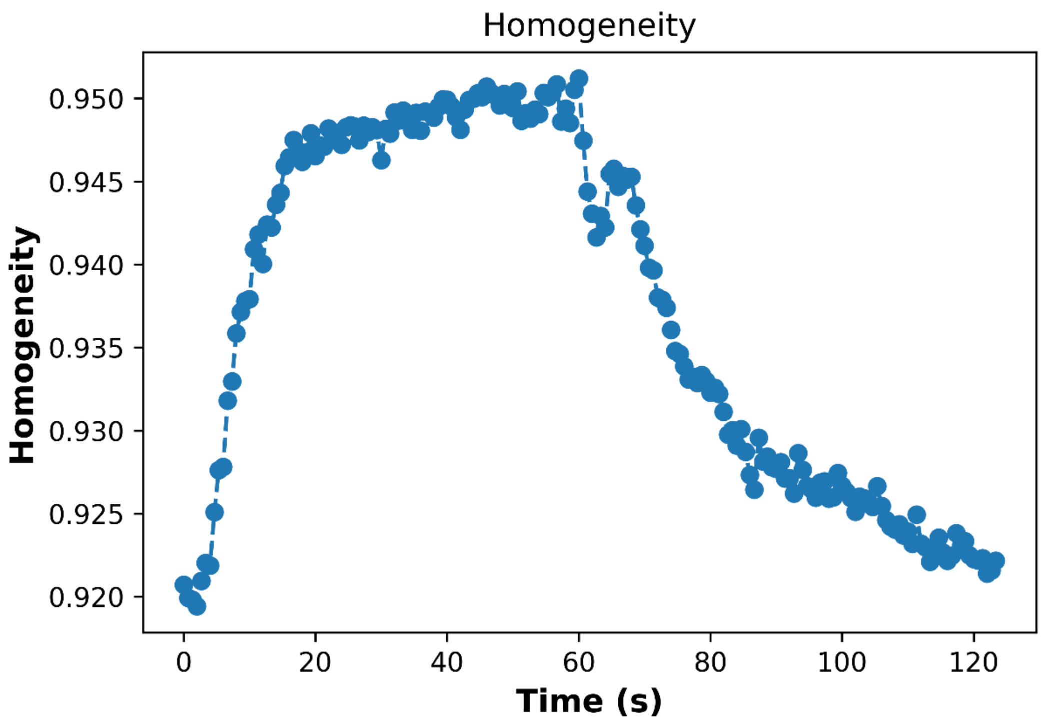
Homogeneity
Homogeneity
0.950
0.945
0.940
0.935
0.930
0.925
0.920
0
20
40
60
80
100
120
Time (s)

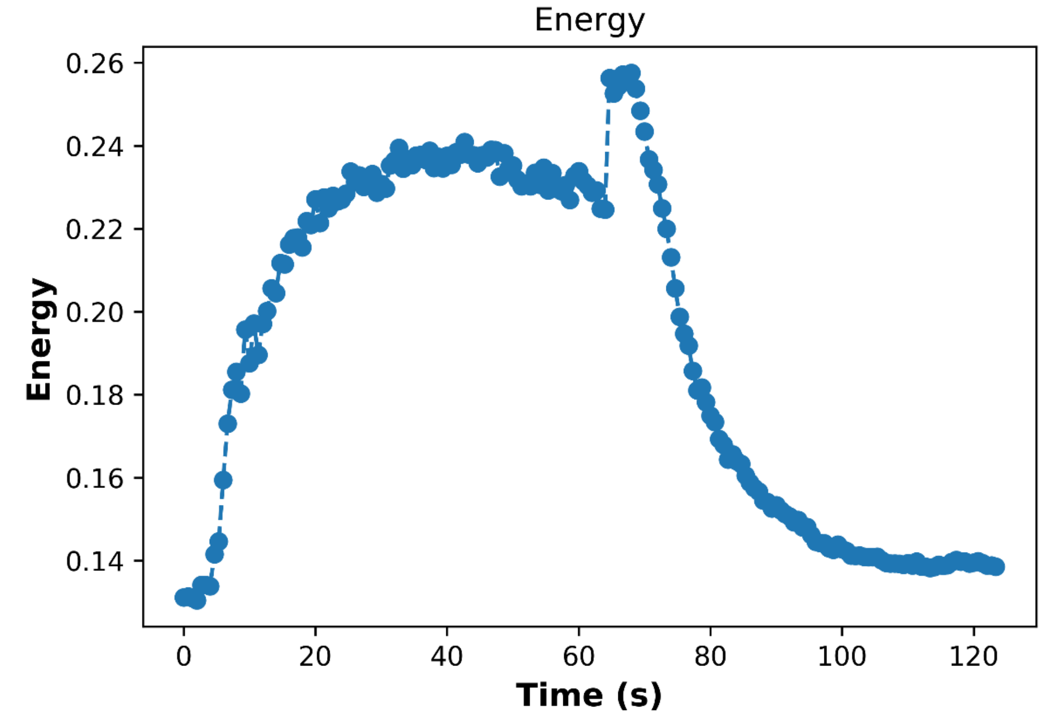
Energy
Energy
0.26
0.24
0.22
0.20
0.18
0.16
0.14
0
20
40
60
80
100
120
Time (s)

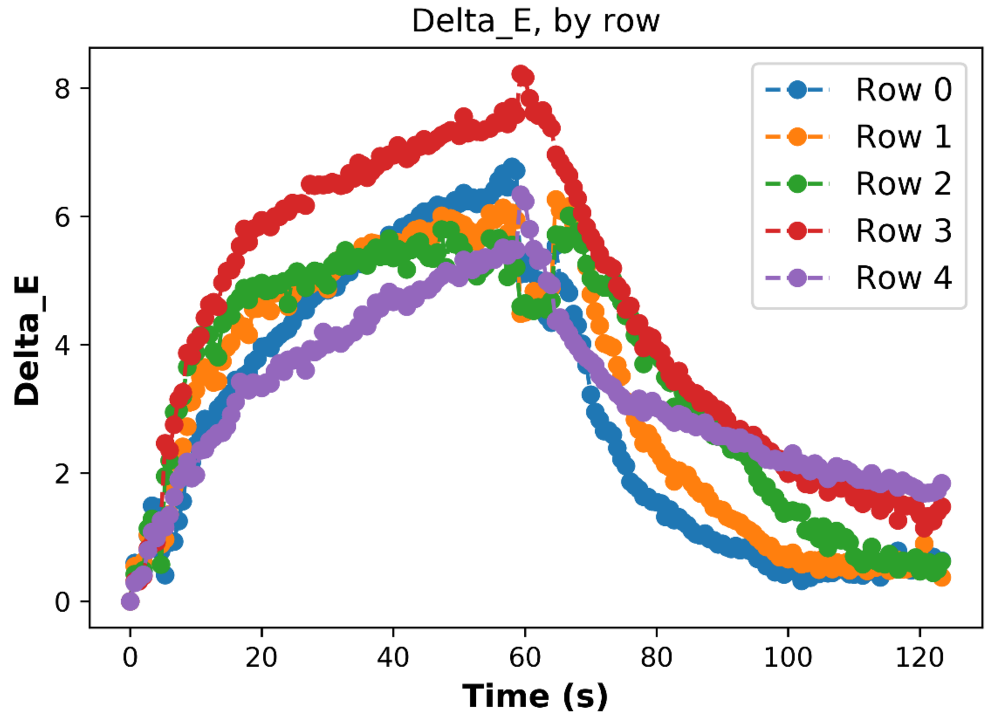
Delta_E, by row
Row 0
Row 1
Row 2
Row 3
Row 4
Delta_E
Time (s)
0
2
4
6
8
0
20
40
60
80
100
120

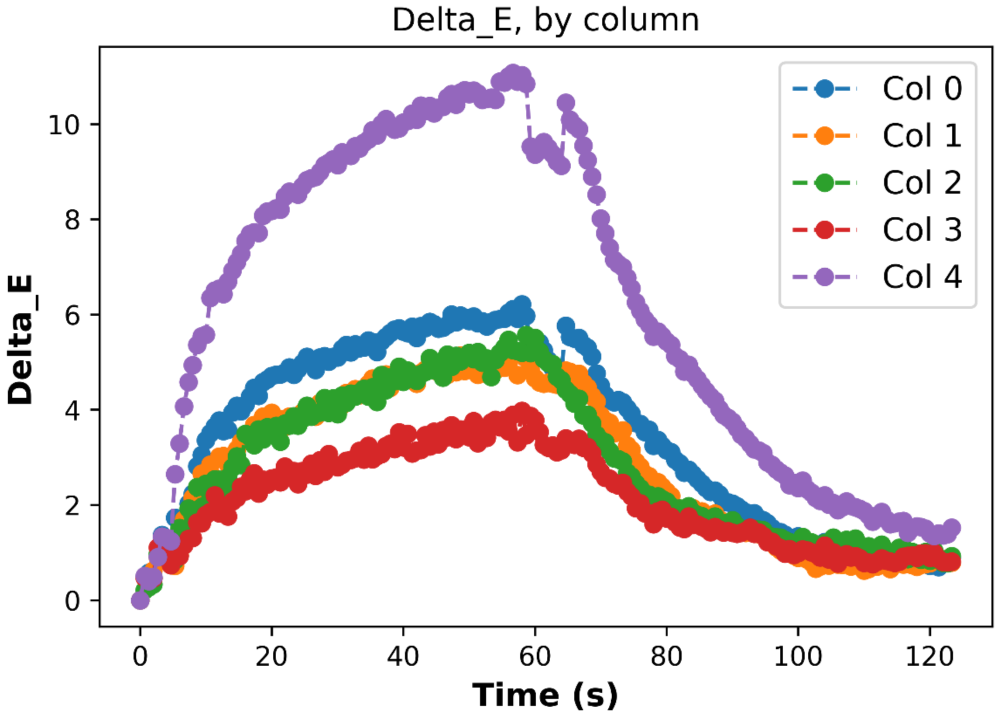
Delta_E, by column
Col 0
Col 1
Col 2
Col 3
Col 4
Delta_E
Time (s)
0
2
4
6
8
10
0
20
40
60
80
100
120

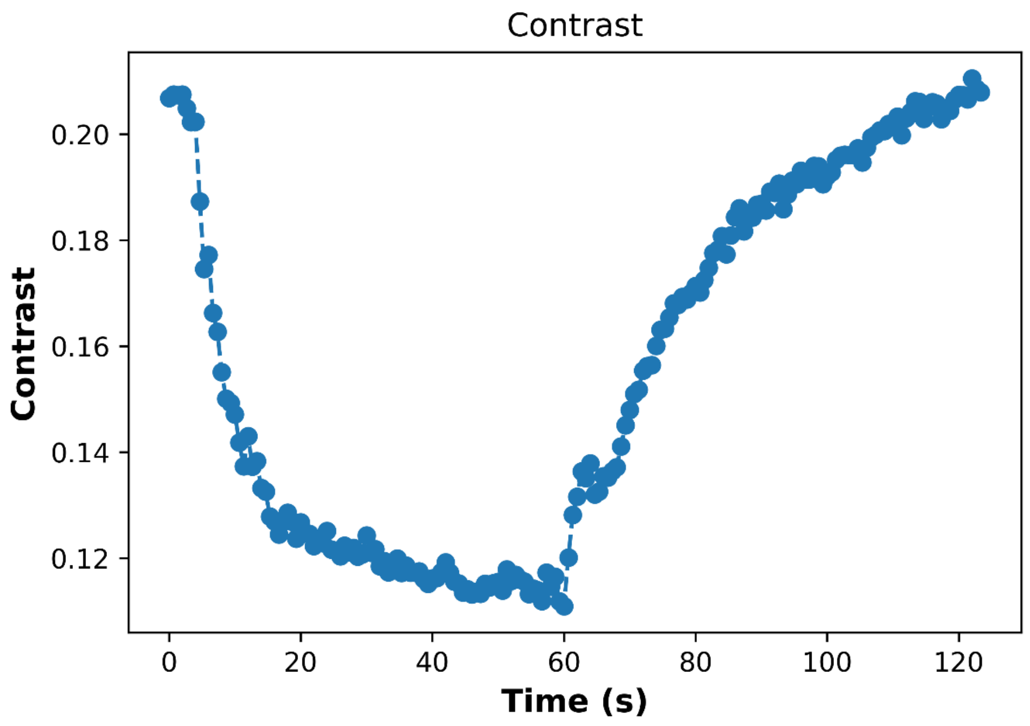
Contrast
Contrast
0.20
0.18
0.16
0.14
0.12
0
20
40
60
80
100
120
Time (s)

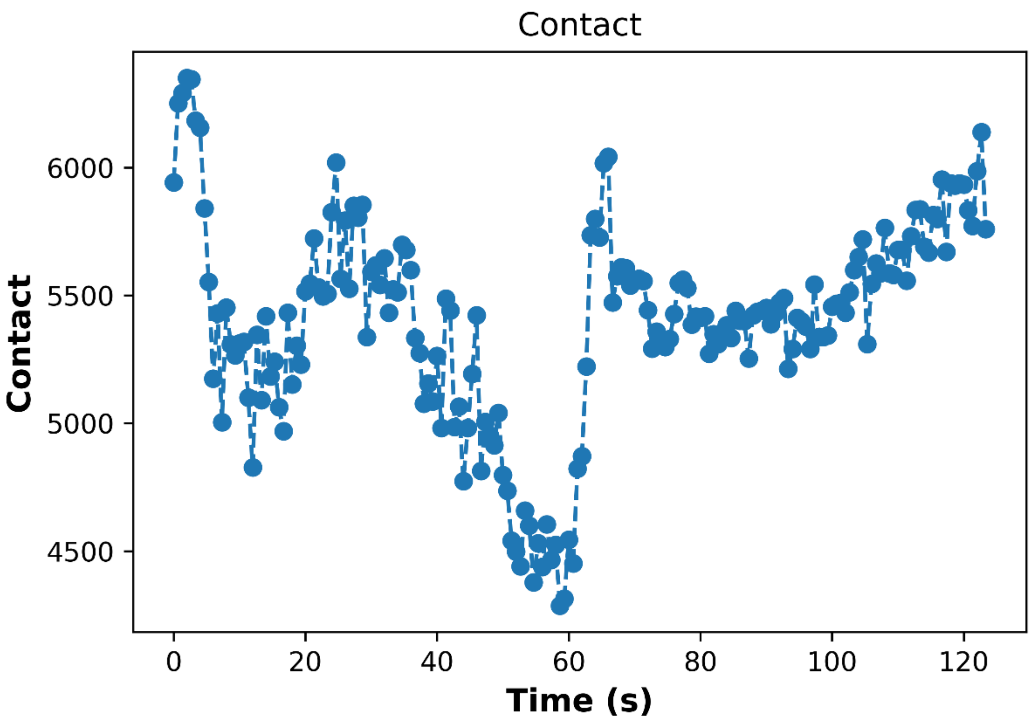
Contact
Contact
6000
5500
5000
4500
0
20
40
60
80
100
120
Time (s)

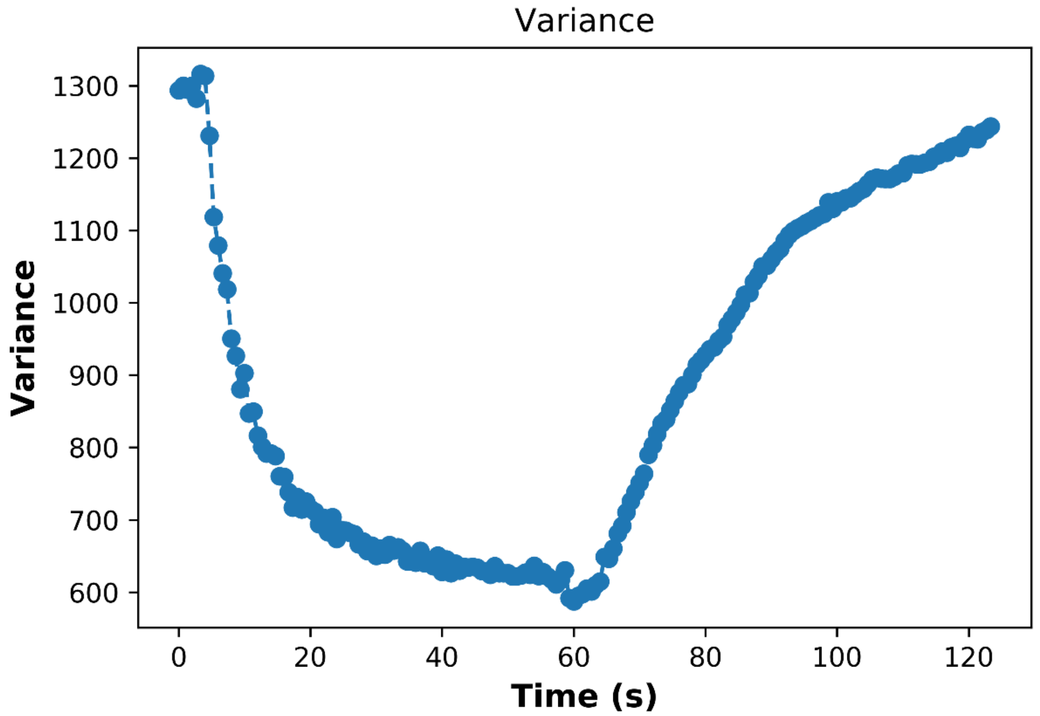
Variance
1300
1200
1100
1000
900
800
700
600
Variance
0
20
40
60
80
100
120
Time (s)

Table 1, Entry 9: 210 RPM, paddle, with baffle, no probe

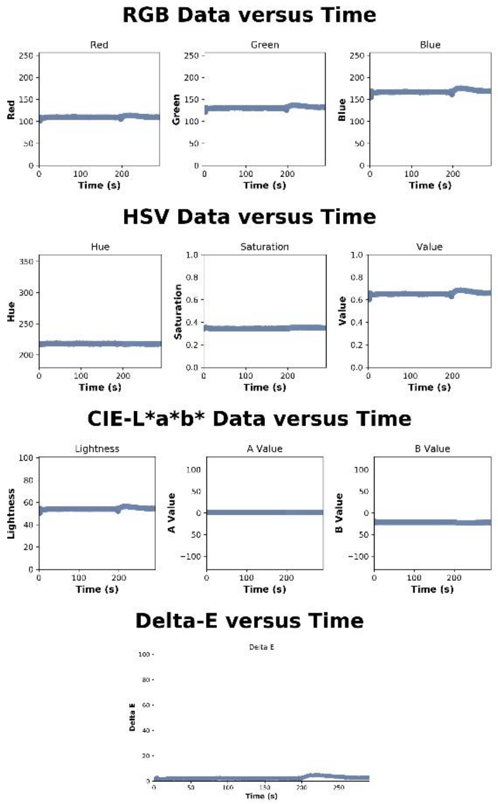

Kineticolor

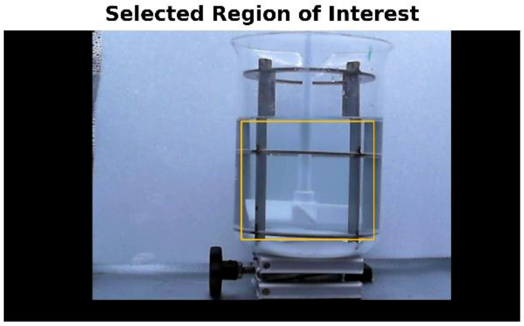

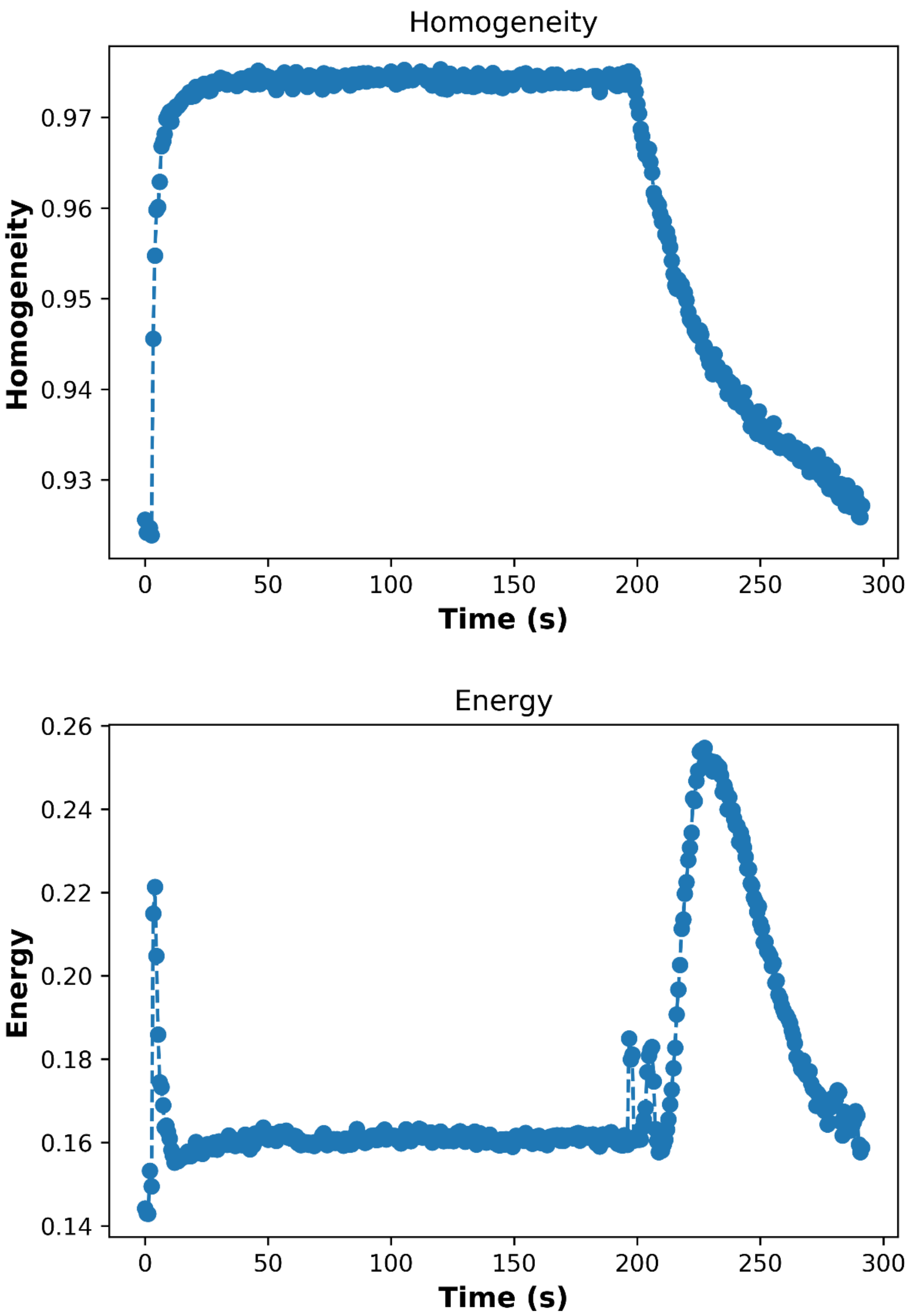
Homogeneity
Homogeneity
0.97
0.96
0.95
0.94
0.93
0
50
100
150
200
250
300
Time (s)
Energy
Energy
0.26
0.24
0.22
0.20
0.18
0.16
0.14
0
50
100
150
200
250
300
Time (s)

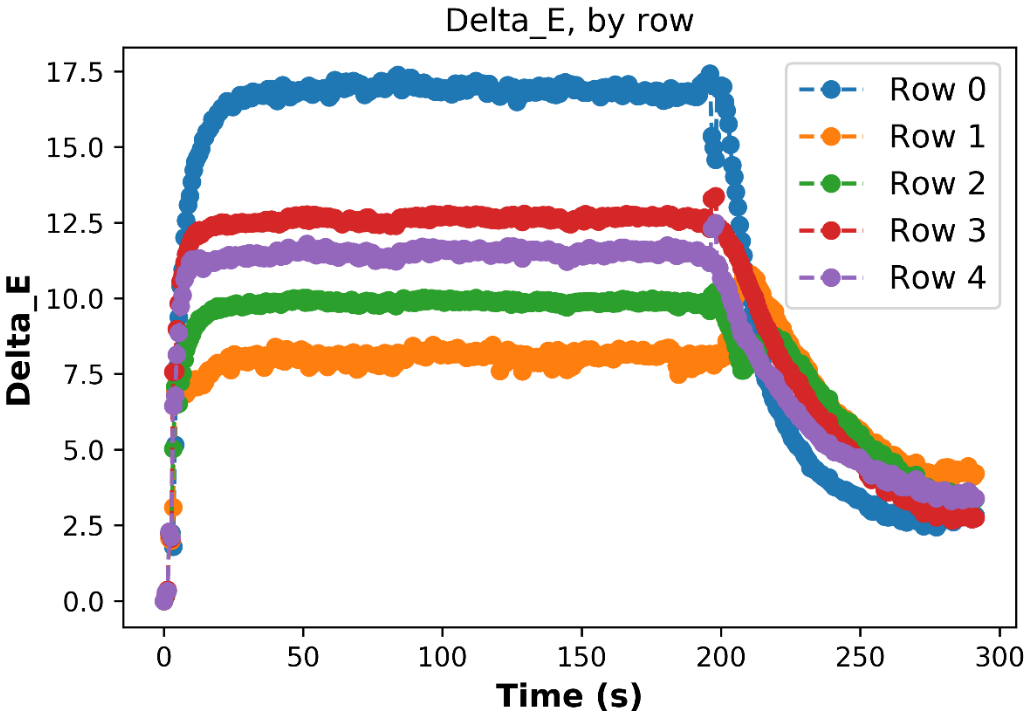
Delta_E, by row
Delta_E
Time (s)
Row 0
Row 1
Row 2
Row 3
Row 4
17.5
15.0
12.5
10.0
7.5
5.0
2.5
0.0
0
50
100
150
200
250
300

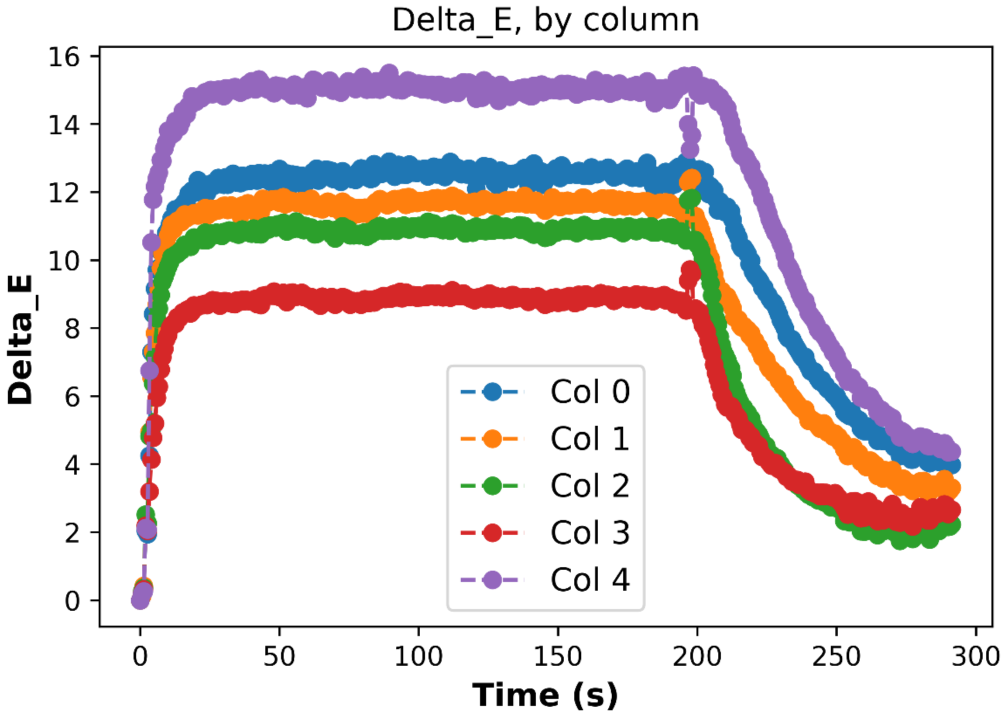
Delta_E, by column
Delta_E
Time (s)
Col 0
Col 1
Col 2
Col 3
Col 4
16
14
12
10
8
6
4
2
0
0
50
100
150
200
250
300

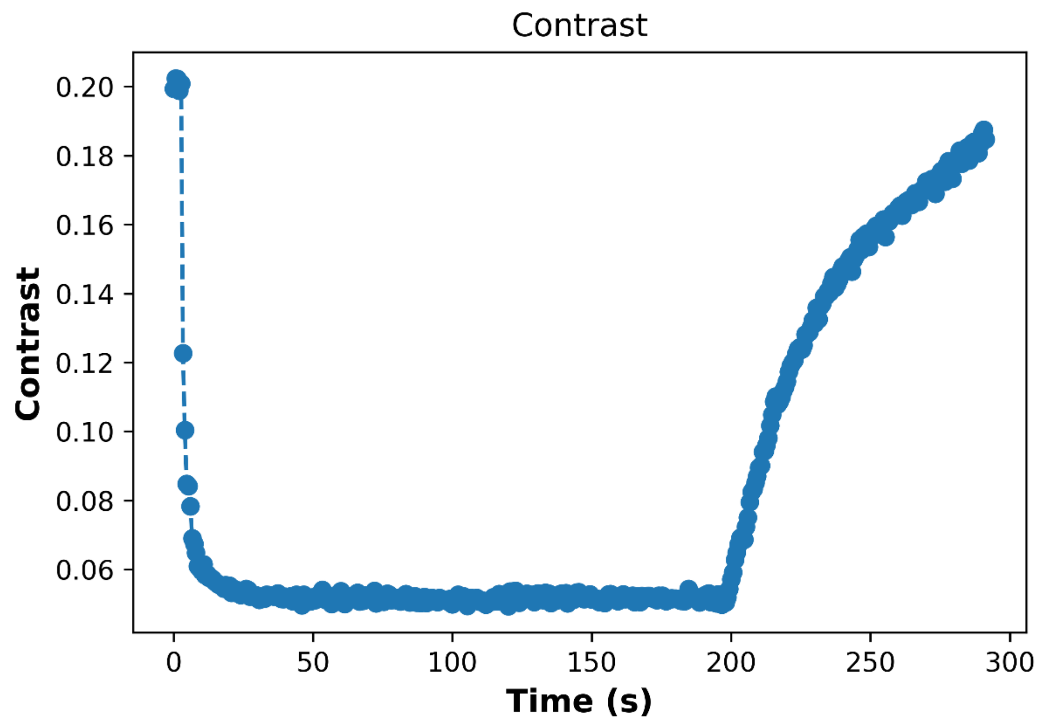

Contrast
0.20
0.18
0.16
0.14
0.12
0.10
0.08
0.06
Contrast
0
50
100
150
200
250
300
Time (s)

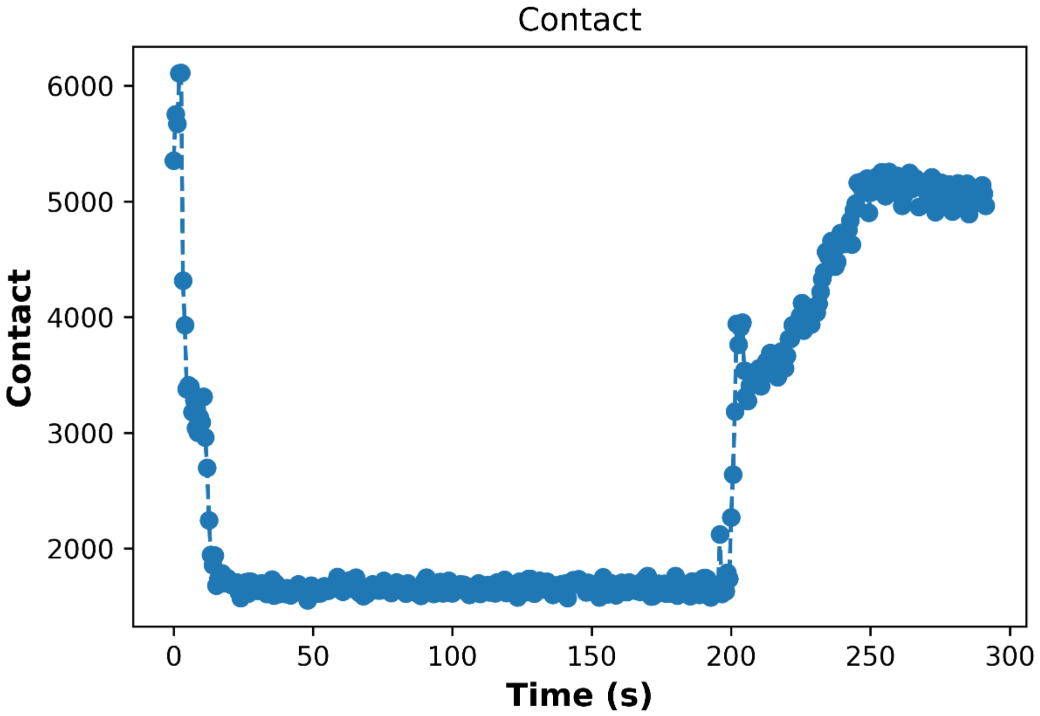

Contact
6000
5000
4000
3000
2000
Contact
0
50
100
150
200
250
300
Time (s)

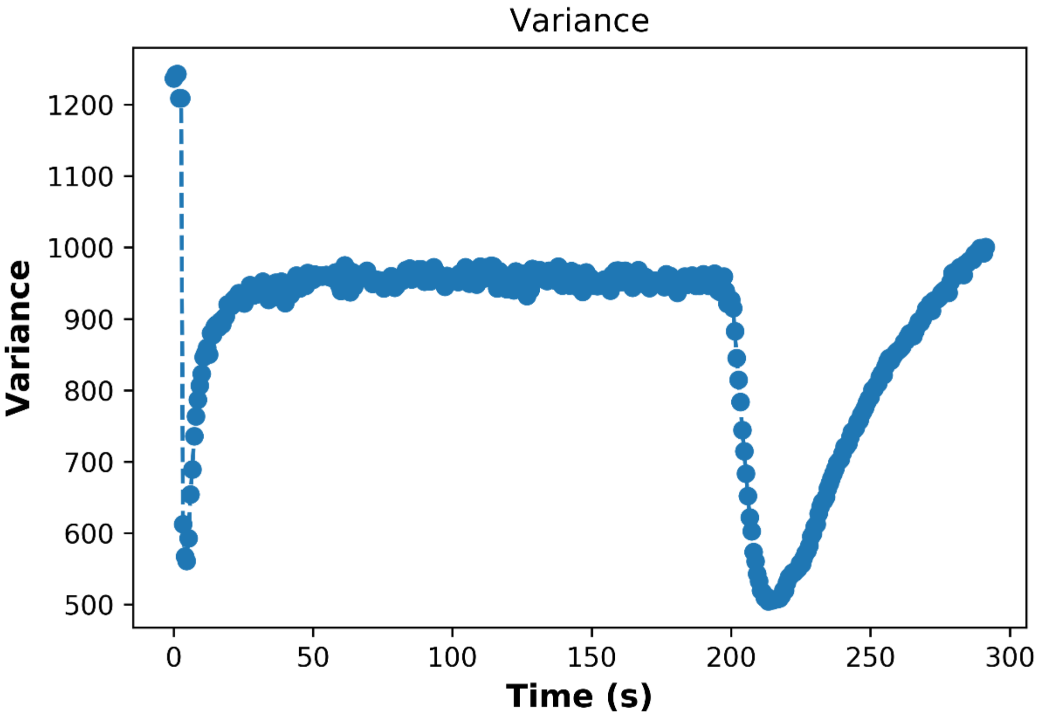

Variance
Variance
1200
1100
1000
900
800
700
600
500
0
50
100
150
200
250
300
Time (s)

Table 1, Entry 10: 100 RPM, anchor, no baffle, no probe

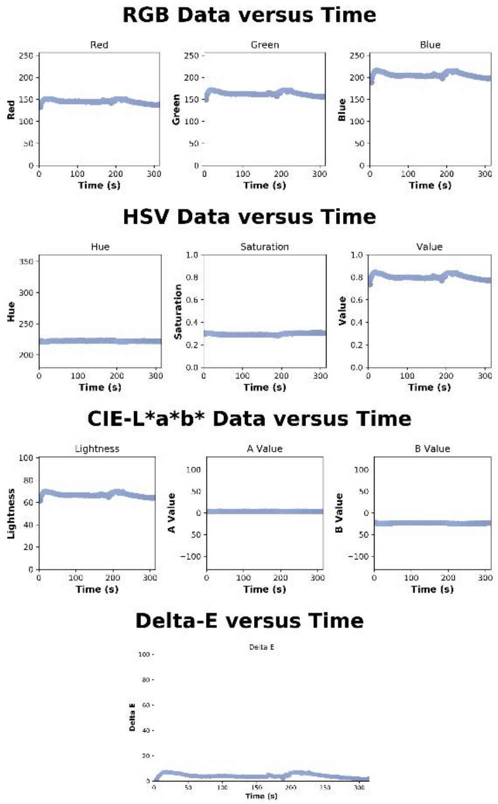

Kineticolor

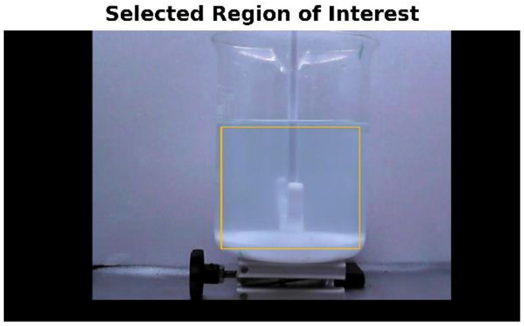

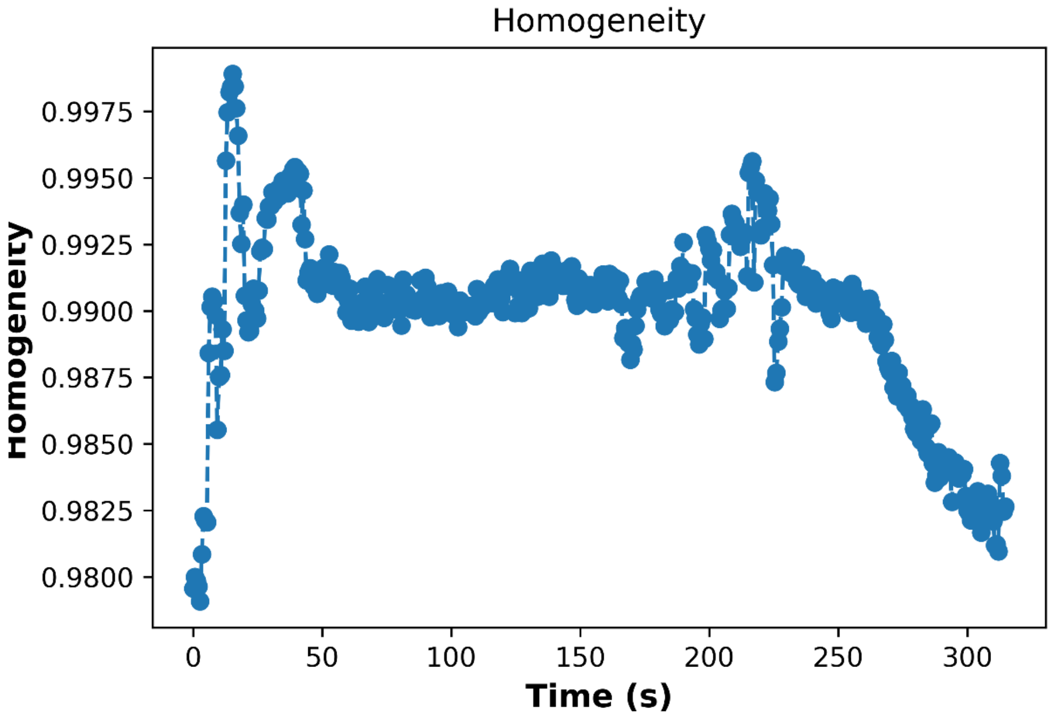
Homogeneity
0.9975
0.9950
0.9925
0.9900
0.9875
0.9850
0.9825
0.9800
Homogeneity
0
50
100
150
200
250
300
Time (s)

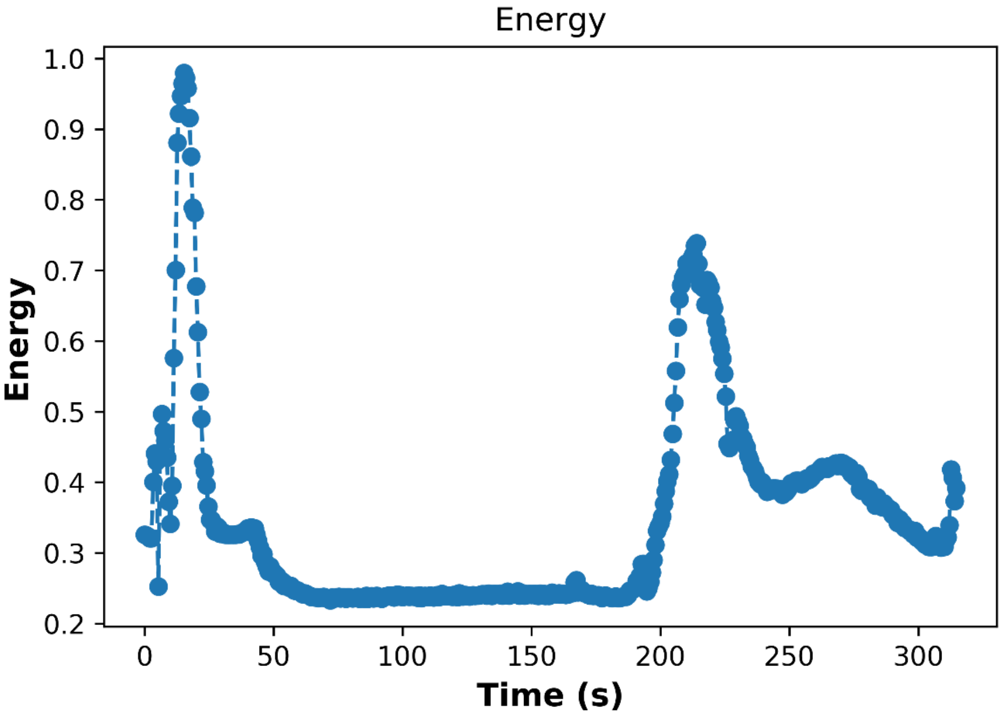
Energy
1.0
0.9
0.8
0.7
0.6
0.5
0.4
0.3
0.2
Energy
0
50
100
150
200
250
300
Time (s)

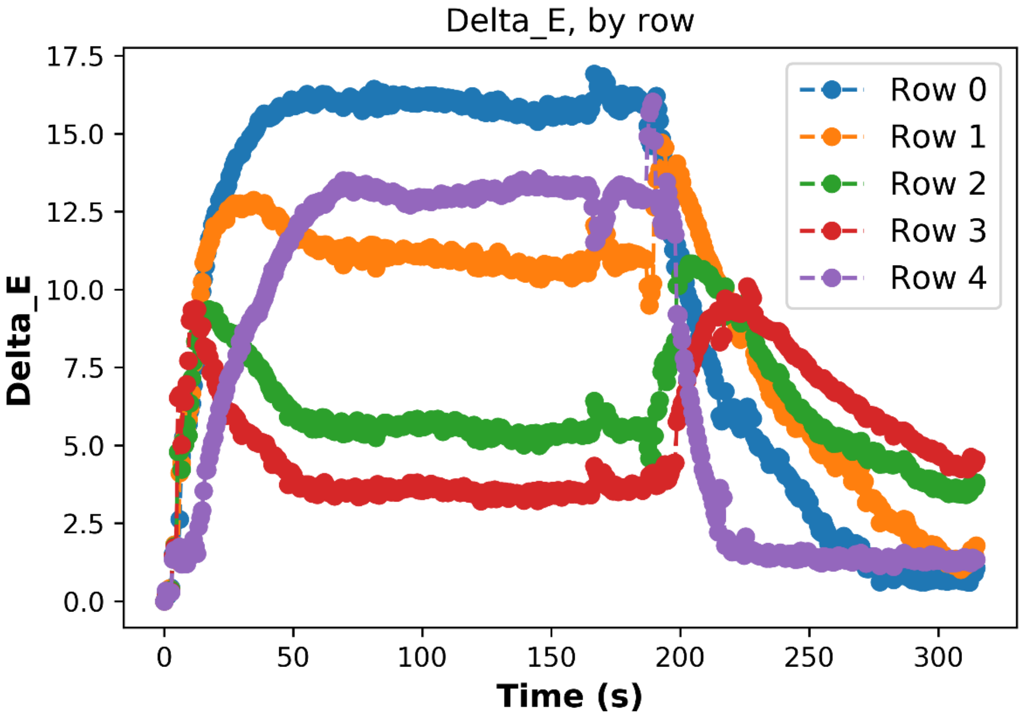
Delta_E, by row
Delta_E
Time (s)
Row 0
Row 1
Row 2
Row 3
Row 4
17.5
15.0
12.5
10.0
7.5
5.0
2.5
0.0
0
50
100
150
200
250
300

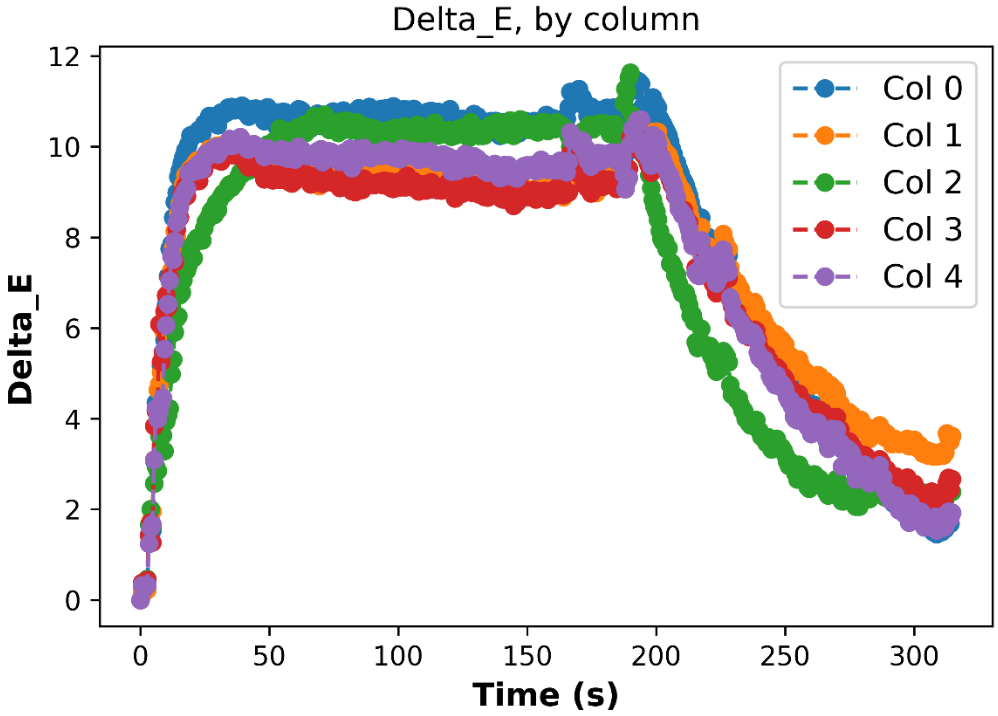
Delta_E, by column
Delta_E
Time (s)
Col 0
Col 1
Col 2
Col 3
Col 4
12
10
8
6
4
2
0
0
50
100
150
200
250
300

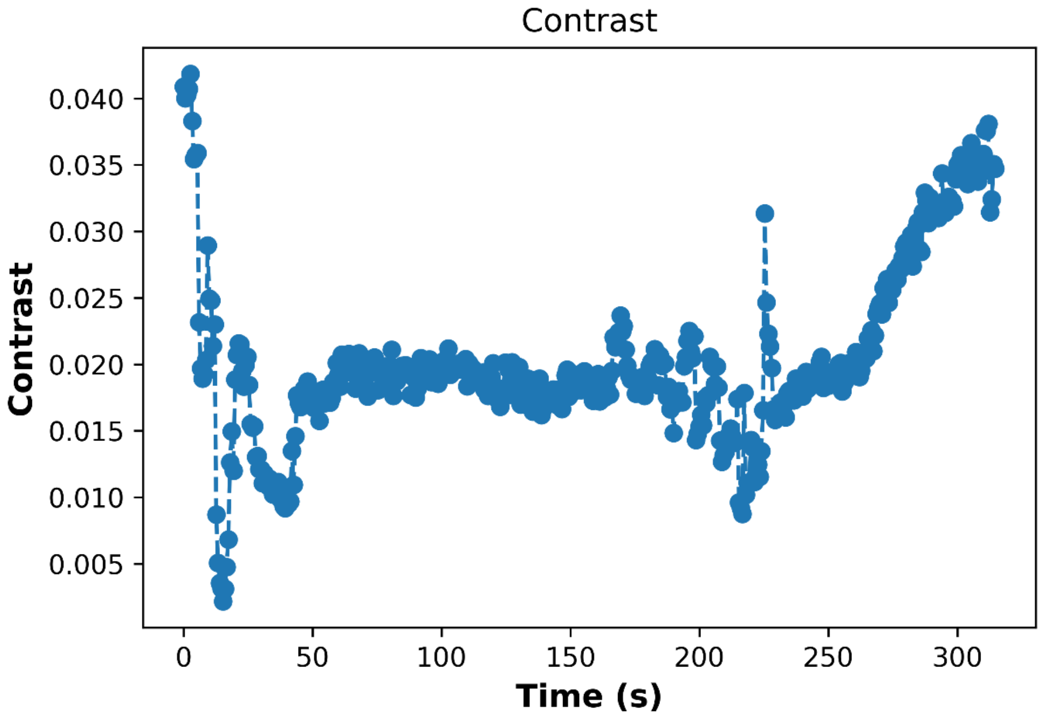
Contrast
Contrast
0.040
0.035
0.030
0.025
0.020
0.015
0.010
0.005
0
50
100
150
200
250
300
Time (s)

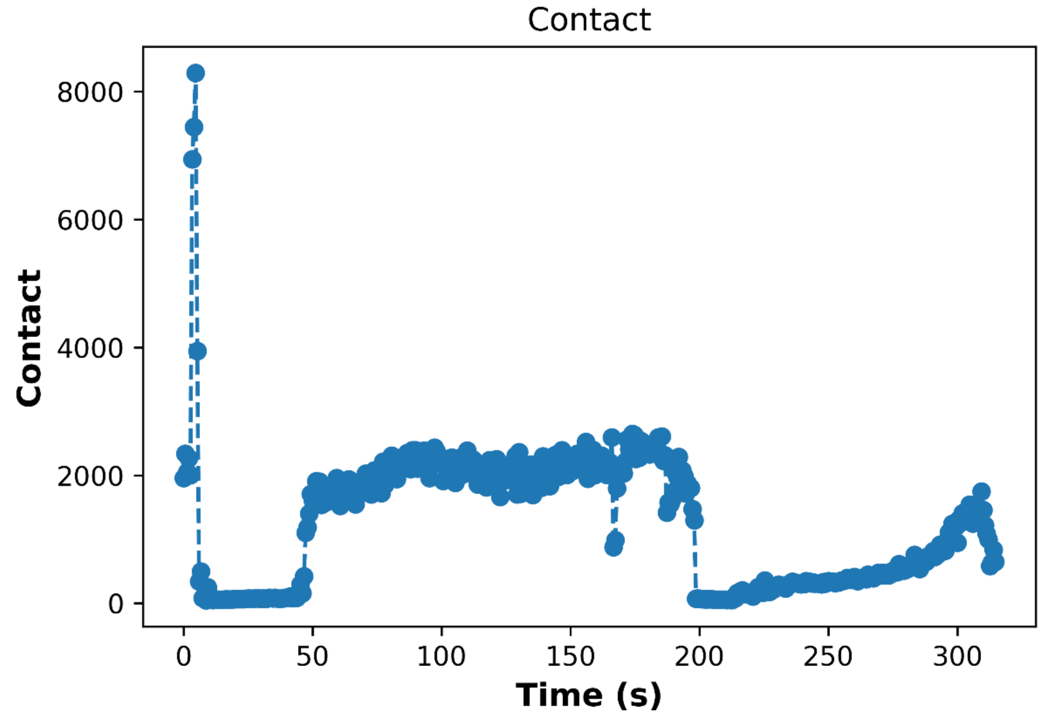
Contact
Contact
8000
6000
4000
2000
0
0
50
100
150
200
250
300
Time (s)

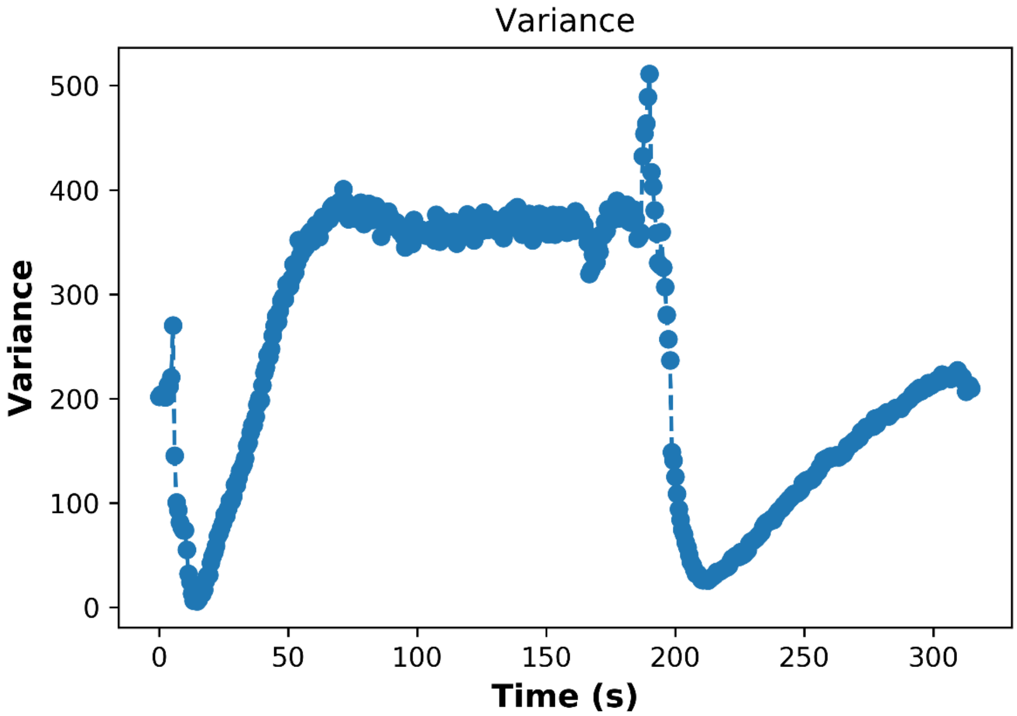

Variance
500
400
300
200
100
0
Variance
0
50
100
150
200
250
300
Time (s)

Table 1, Entry 10: 100 RPM, anchor, with baffle, no probe

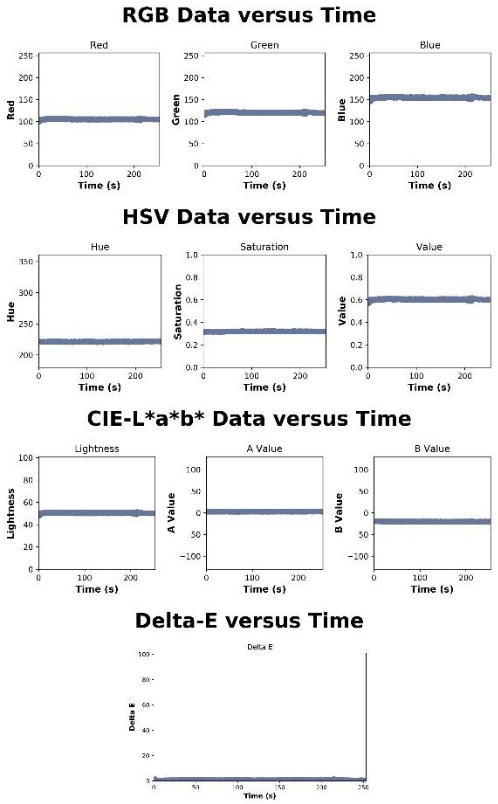

Kineticolor

Selected Region of Interest

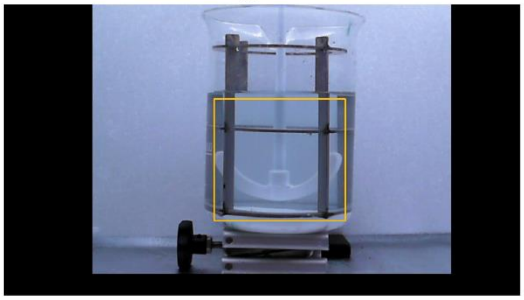

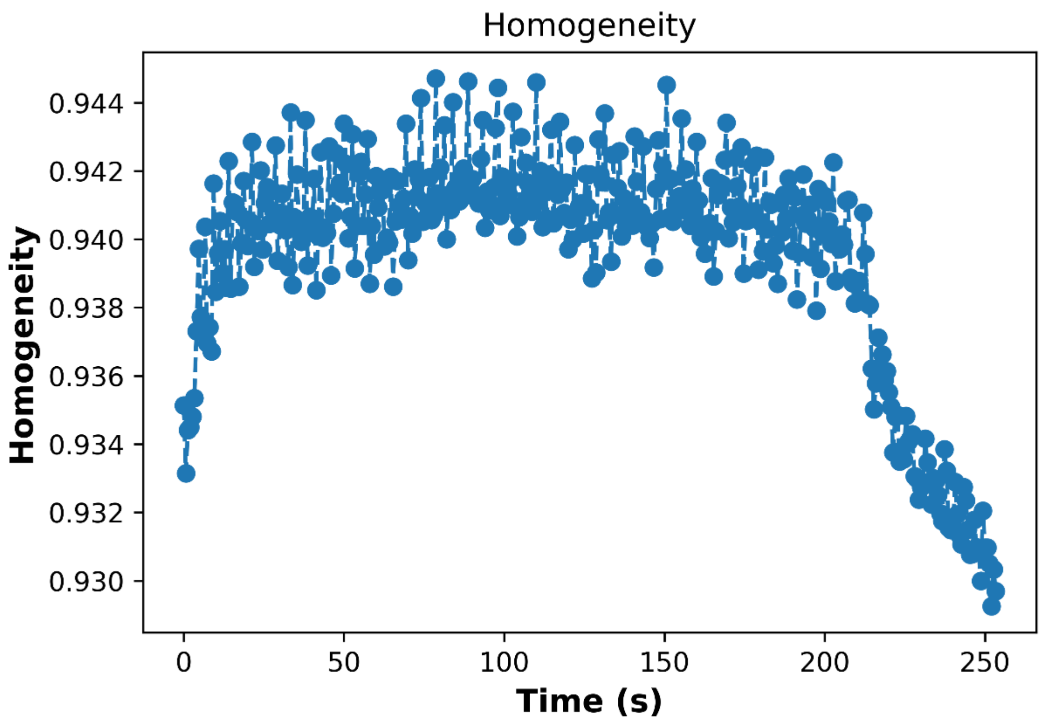
Homogeneity
0.944
0.942
0.940
0.938
0.936
0.934
0.932
0.930
Homogeneity
0
50
100
150
200
250
Time (s)

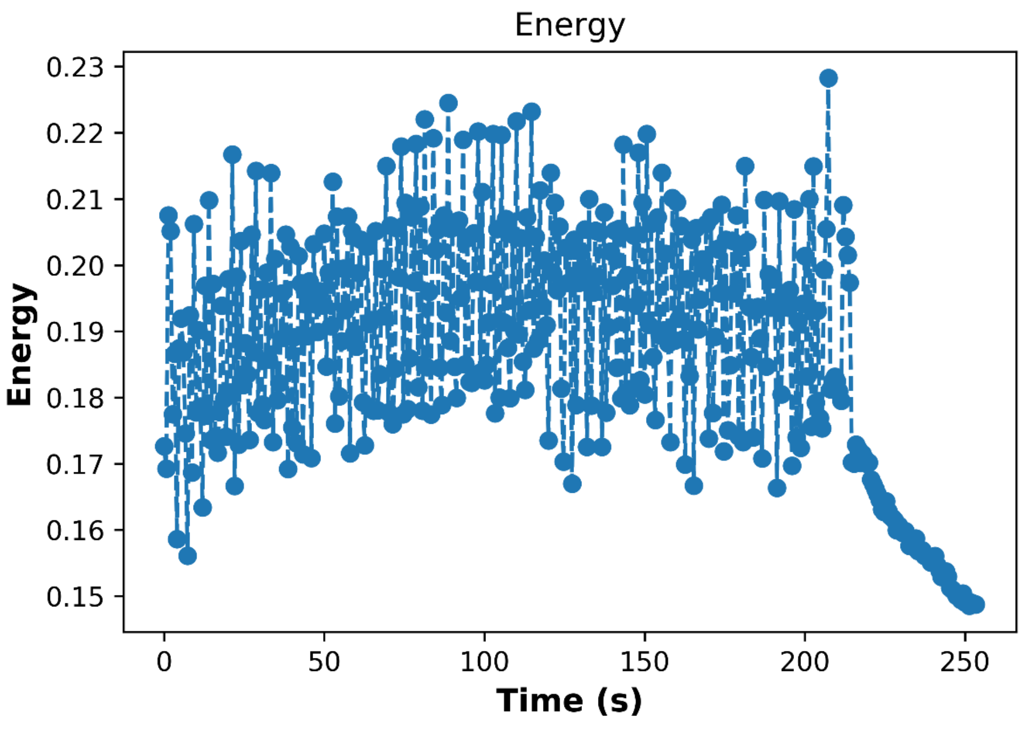
Energy
0.23
0.22
0.21
0.20
0.19
0.18
0.17
0.16
0.15
Energy
0
50
100
150
200
250
Time (s)

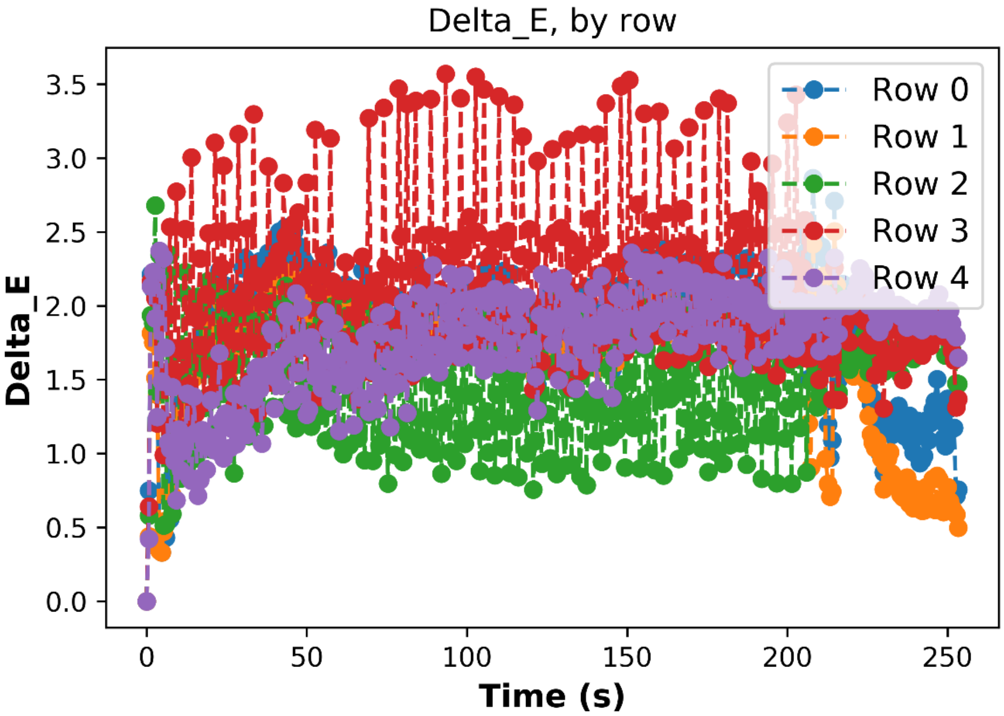
Delta_E, by row
Delta_E
Time (s)
Row 0
Row 1
Row 2
Row 3
Row 4
3.5
3.0
2.5
2.0
1.5
1.0
0.5
0.0
0
50
100
150
200
250

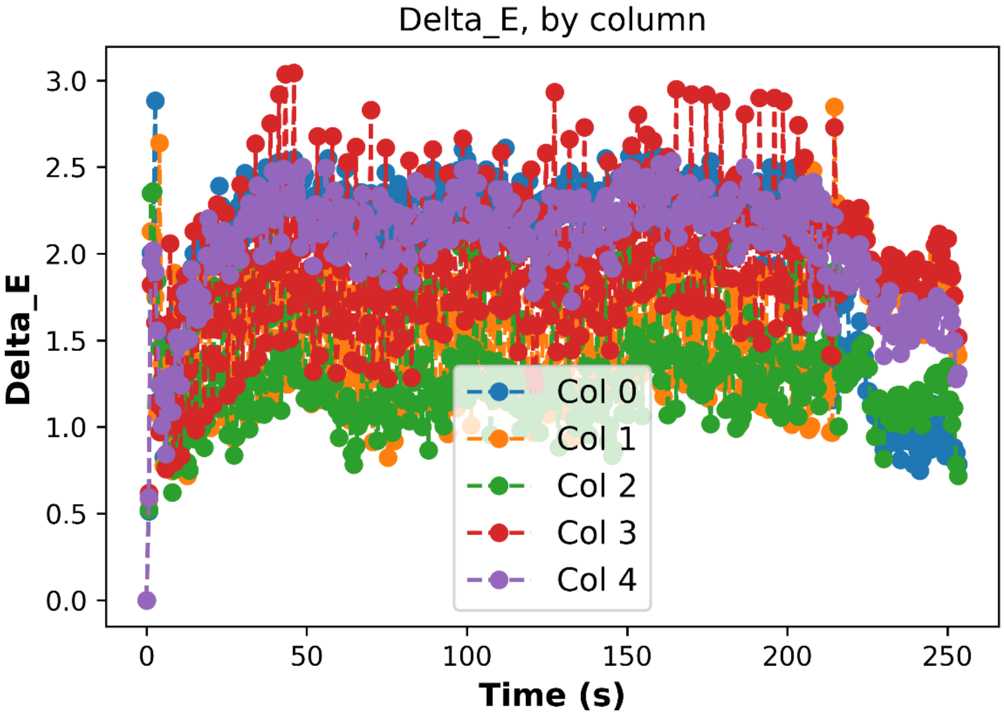
Delta_E, by column
Delta_E
Time (s)
Col 0
Col 1
Col 2
Col 3
Col 4
3.0
2.5
2.0
1.5
1.0
0.5
0.0
0
50
100
150
200
250

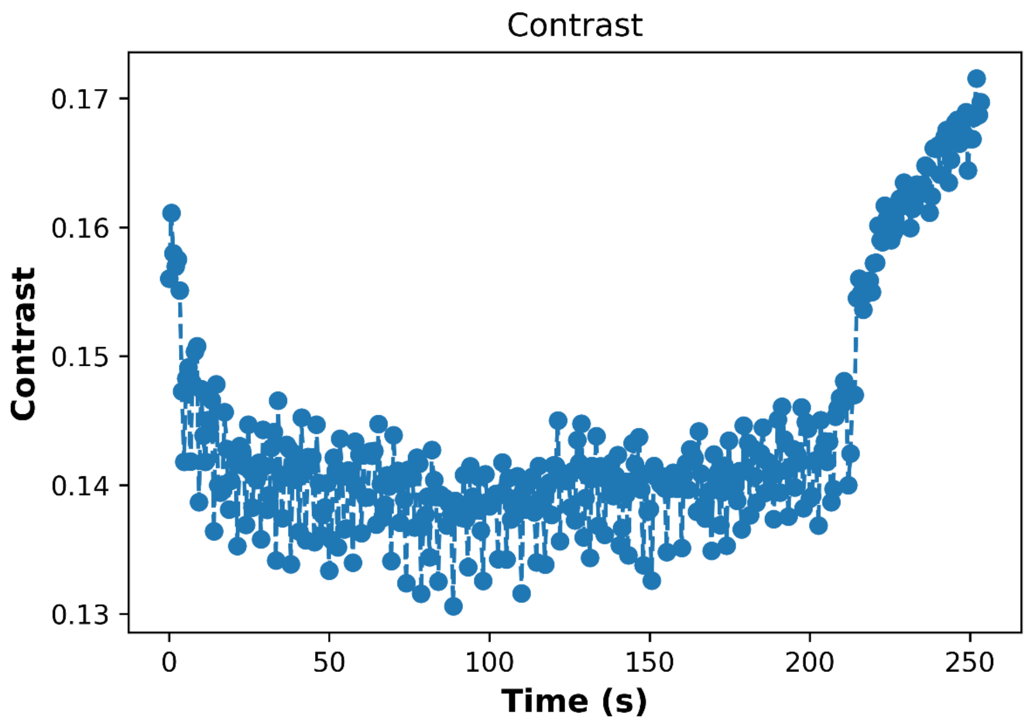
Contrast
0.17
0.16
0.15
0.14
0.13
Contrast
0
50
100
150
200
250
Time (s)

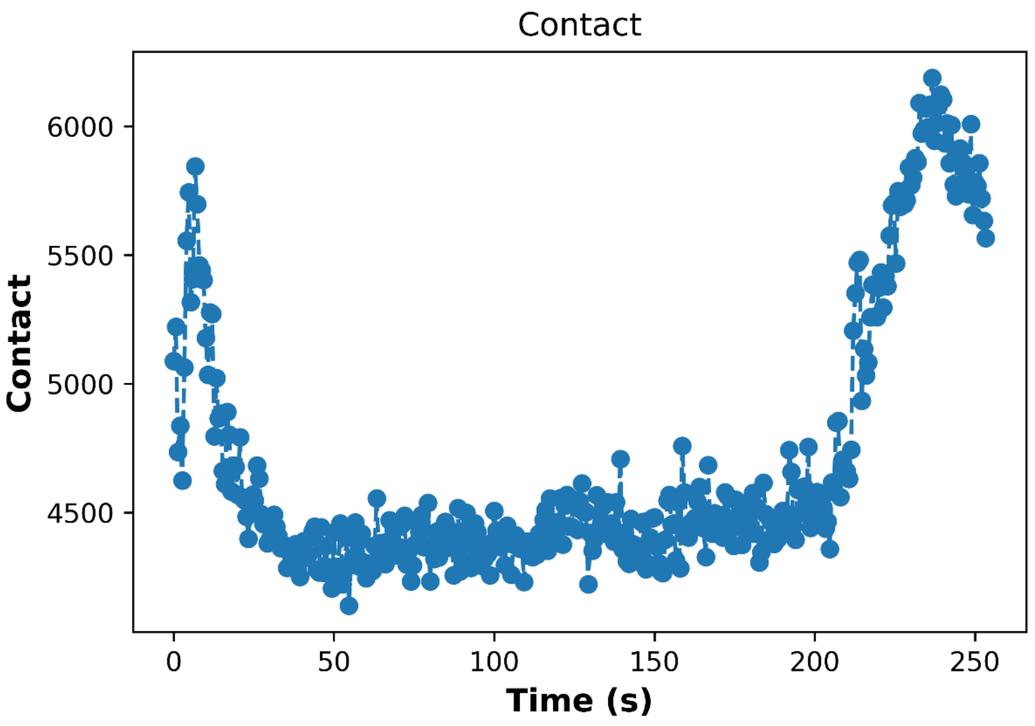
Contact
6000
5500
5000
4500
Contact
0
50
100
150
200
250
Time (s)

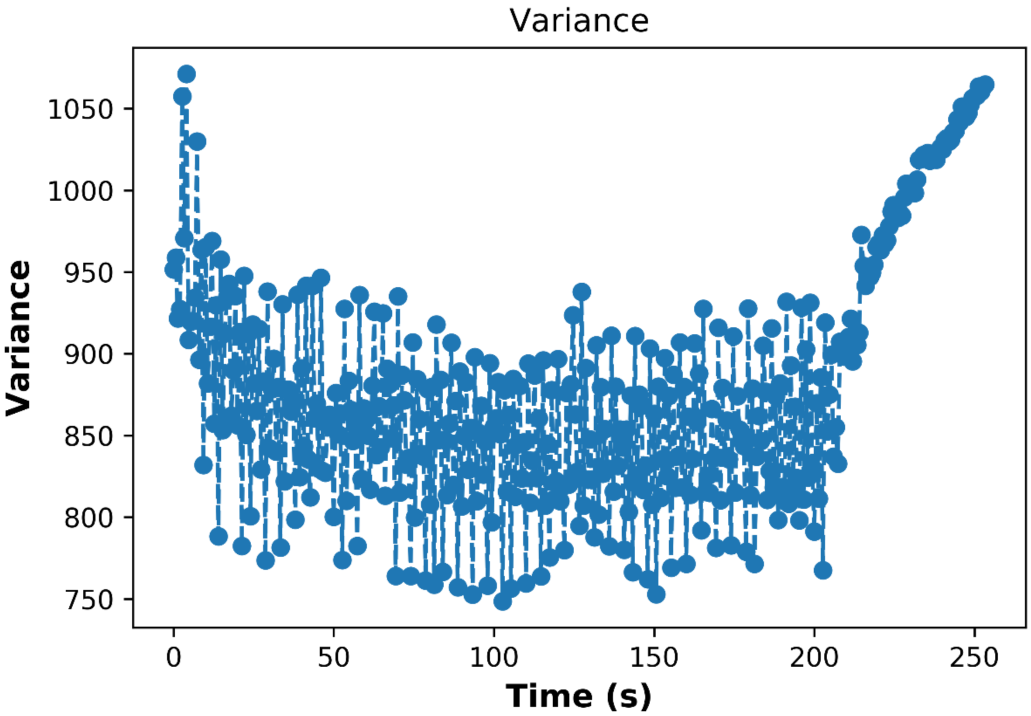

Variance
1050
1000
950
900
850
800
750
Variance
0
50
100
150
200
250
Time (s)

Table 1, Entry 11: 210 RPM, anchor, no baffle, no probe

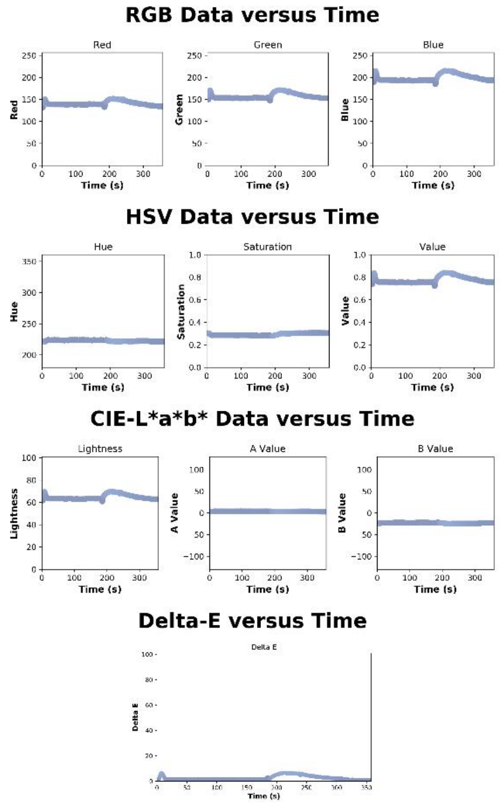

Kineticolor

Selected Region of Interest

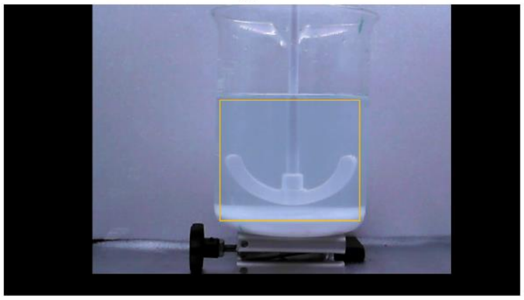

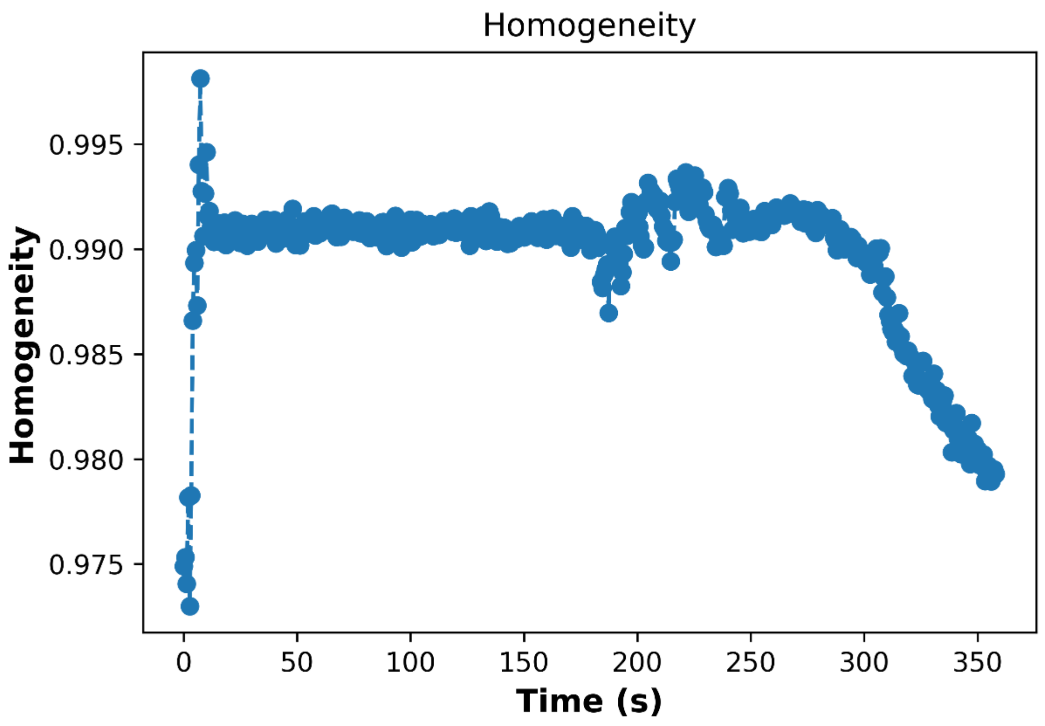
Homogeneity
Homogeneity
0.995
0.990
0.985
0.980
0.975
0
50
100
150
200
250
300
350
Time (s)

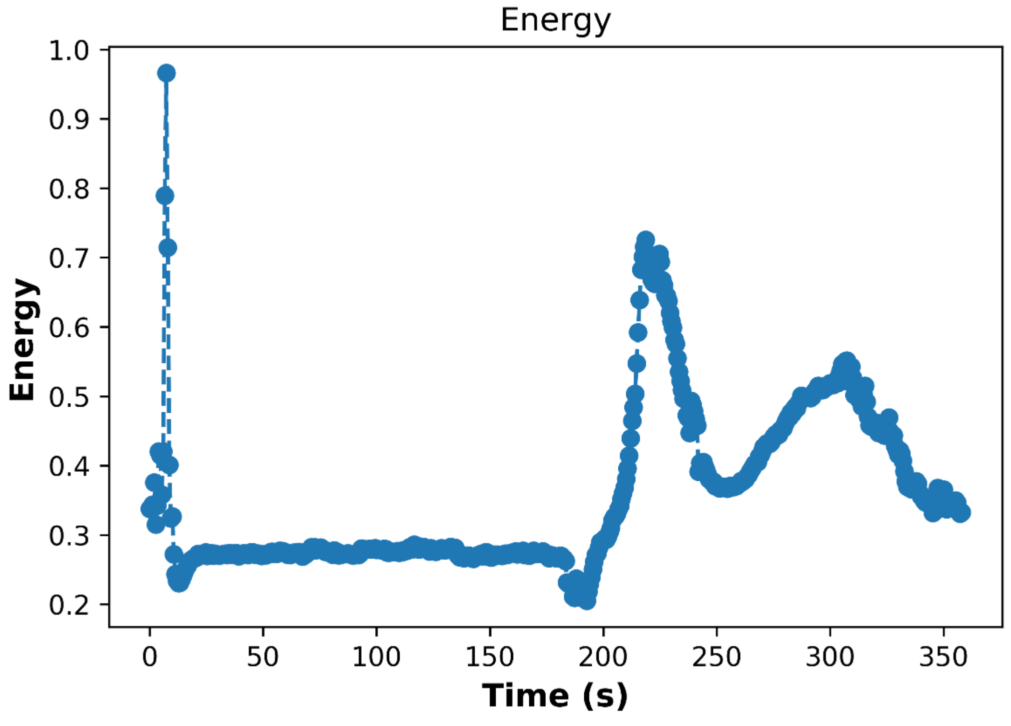
Energy
Energy
1.0
0.9
0.8
0.7
0.6
0.5
0.4
0.3
0.2
0
50
100
150
200
250
300
350
Time (s)

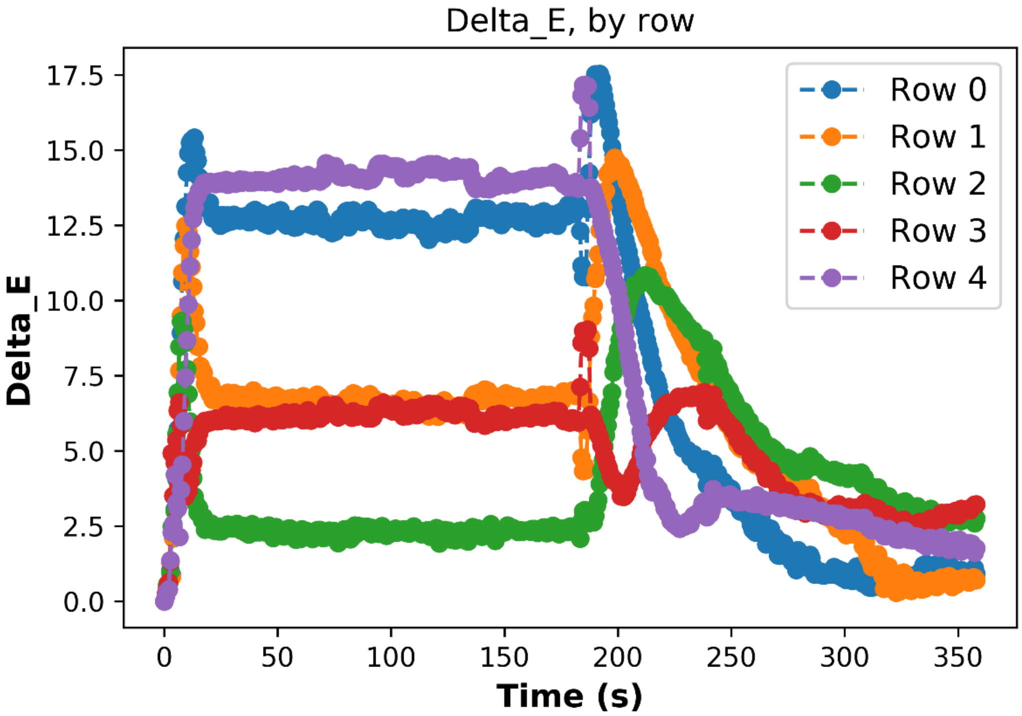

Delta_E, by row
Delta_E
Time (s)
Row 0
Row 1
Row 2
Row 3
Row 4
17.5
15.0
12.5
10.0
7.5
5.0
2.5
0.0
0
50
100
150
200
250
300
350

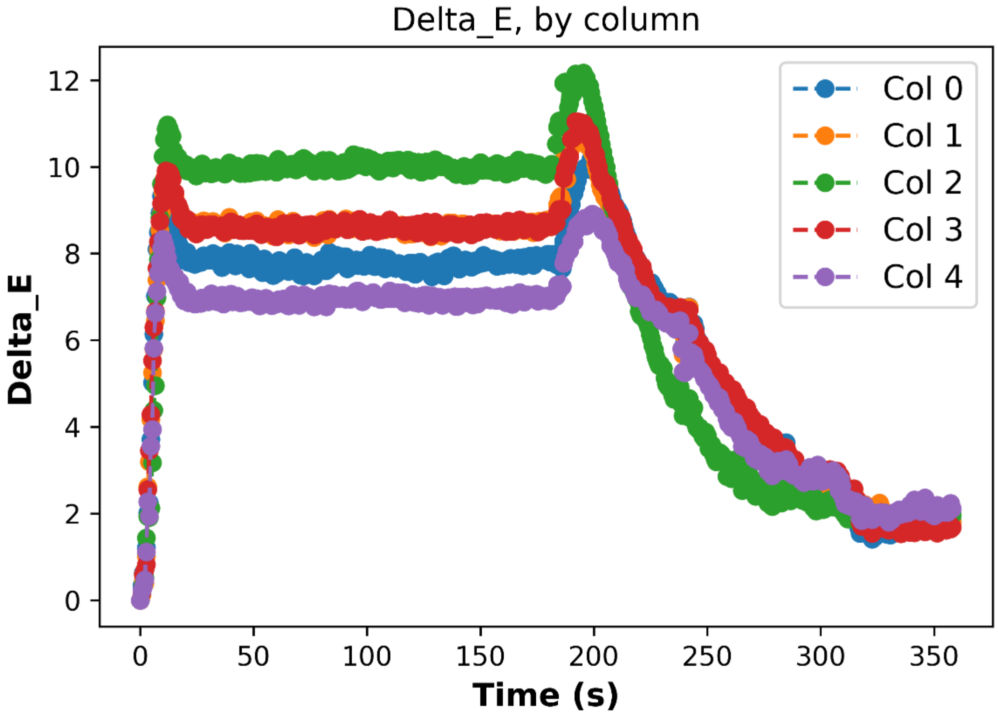

Delta_E, by column
Delta_E
Time (s)
Col 0
Col 1
Col 2
Col 3
Col 4
12
10
8
6
4
2
0
0
50
100
150
200
250
300
350

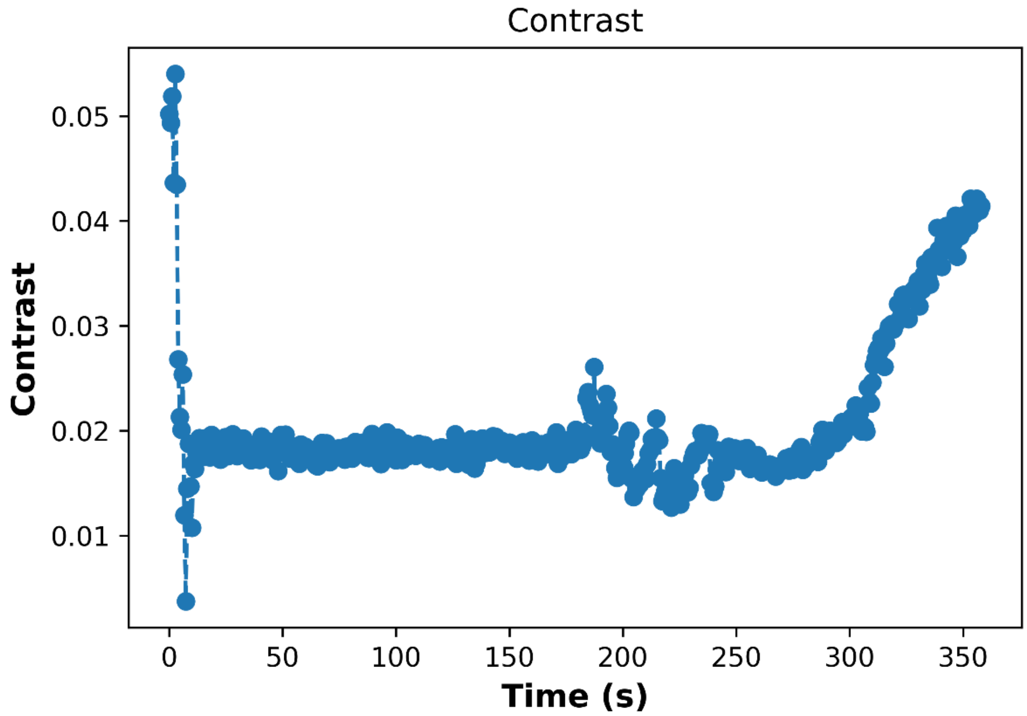
Contrast
Contrast
0.05
0.04
0.03
0.02
0.01
0
50
100
150
200
250
300
350
Time (s)

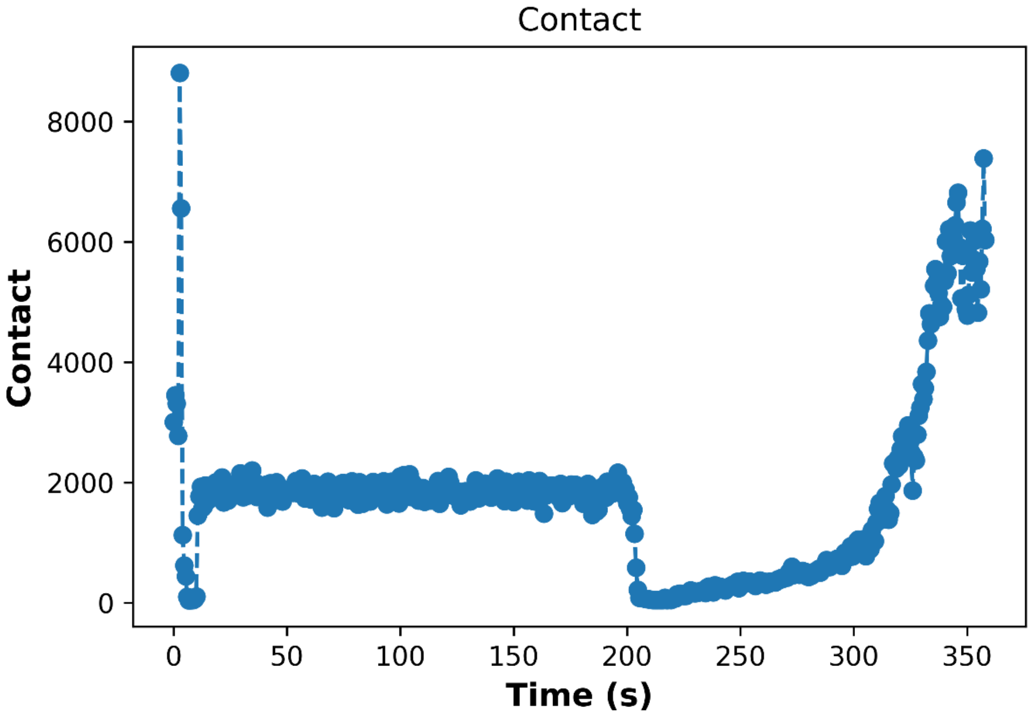
Contact
Contact
8000
6000
4000
2000
0
0
50
100
150
200
250
300
350
Time (s)

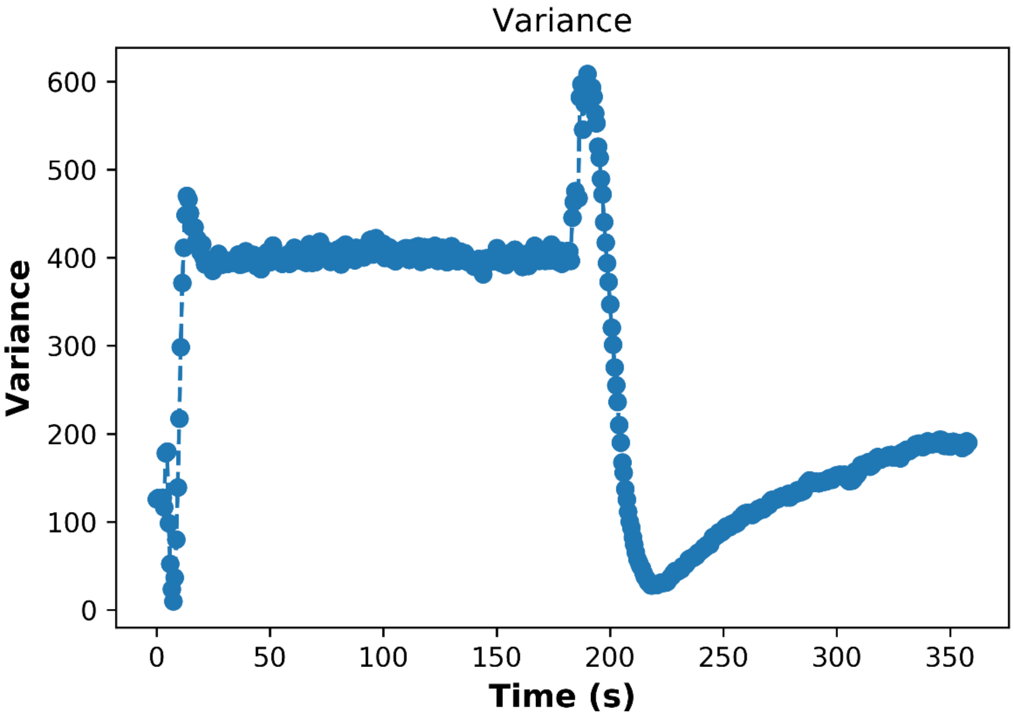
Variance
600
500
400
300
200
100
0
Variance
0
50
100
150
200
250
300
350
Time (s)

Table 1, Entry 11: 210 RPM, anchor, with baffle, no probe

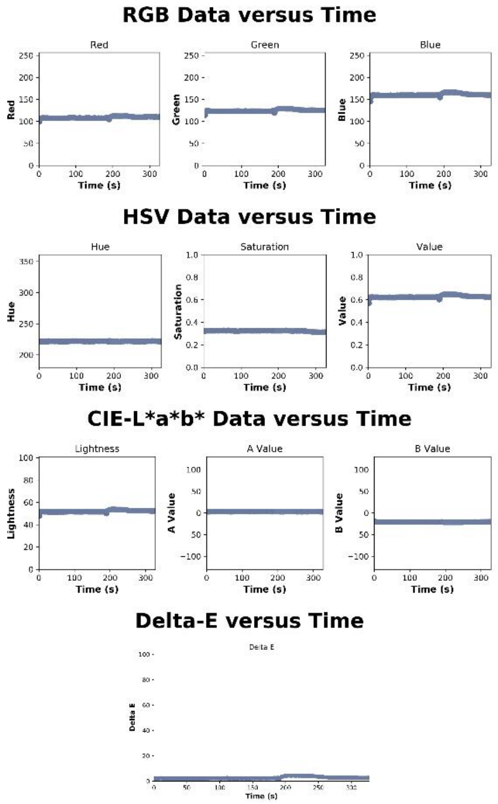

Kineticolor

Selected Region of Interest

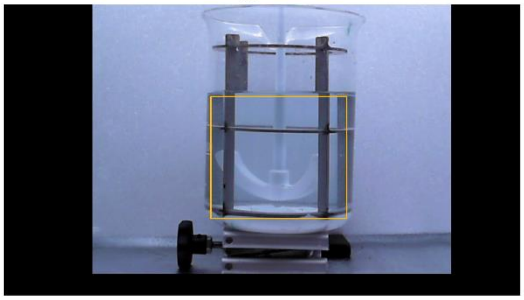

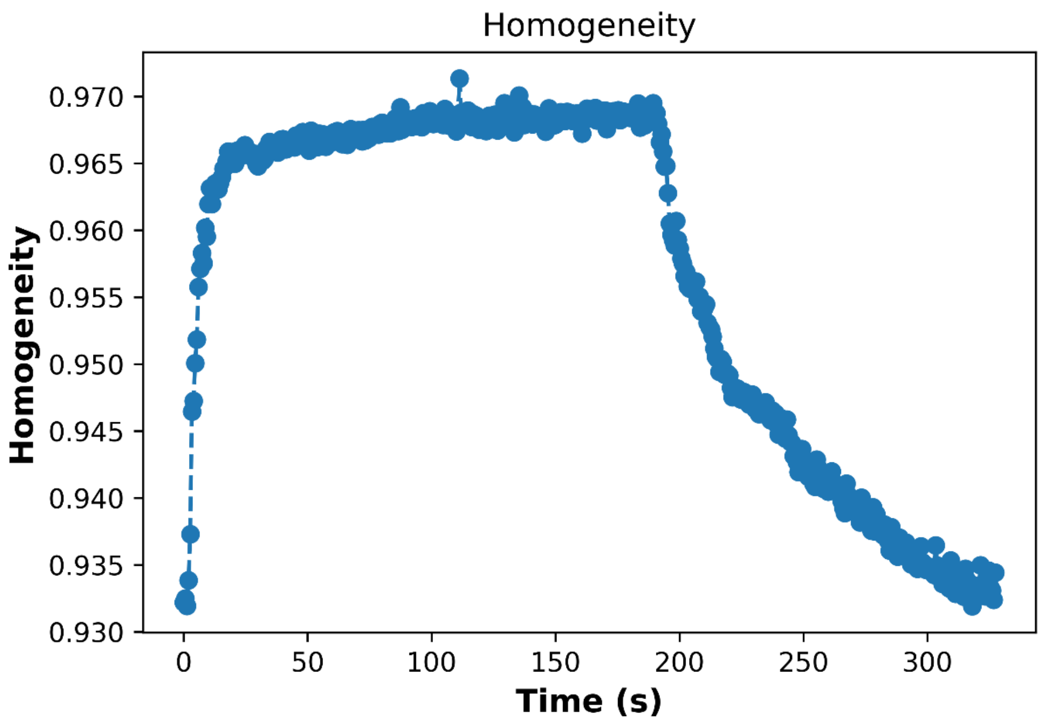

Homogeneity
Homogeneity
0.970
0.965
0.960
0.955
0.950
0.945
0.940
0.935
0.930
0
50
100
150
200
250
300
Time (s)

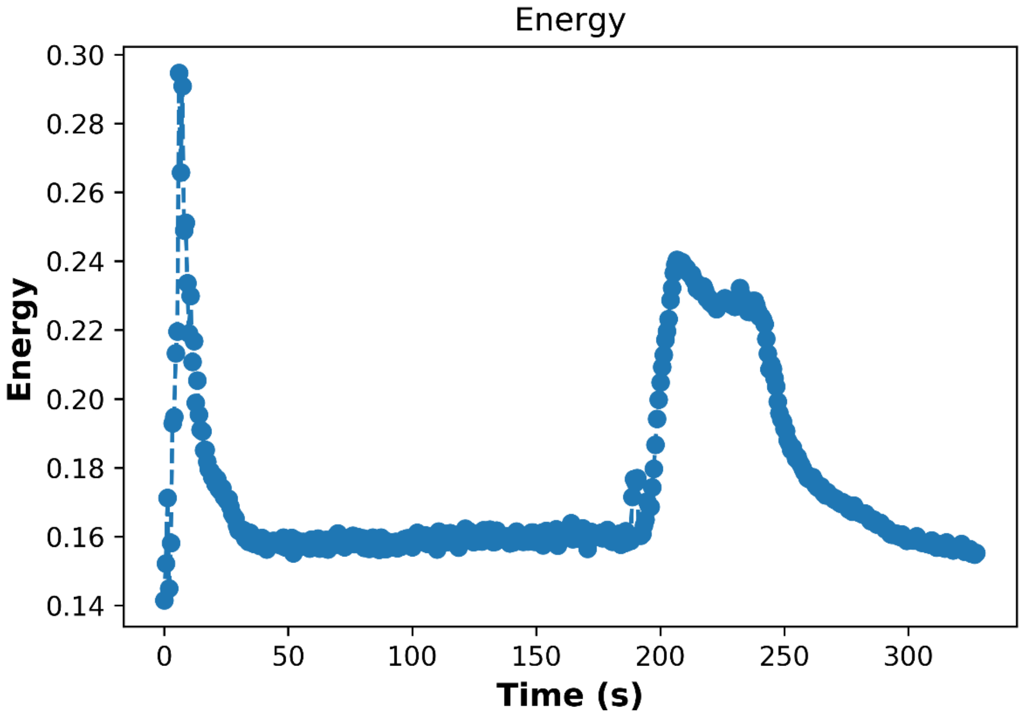

Energy
Energy
0.30
0.28
0.26
0.24
0.22
0.20
0.18
0.16
0.14
0
50
100
150
200
250
300
Time (s)

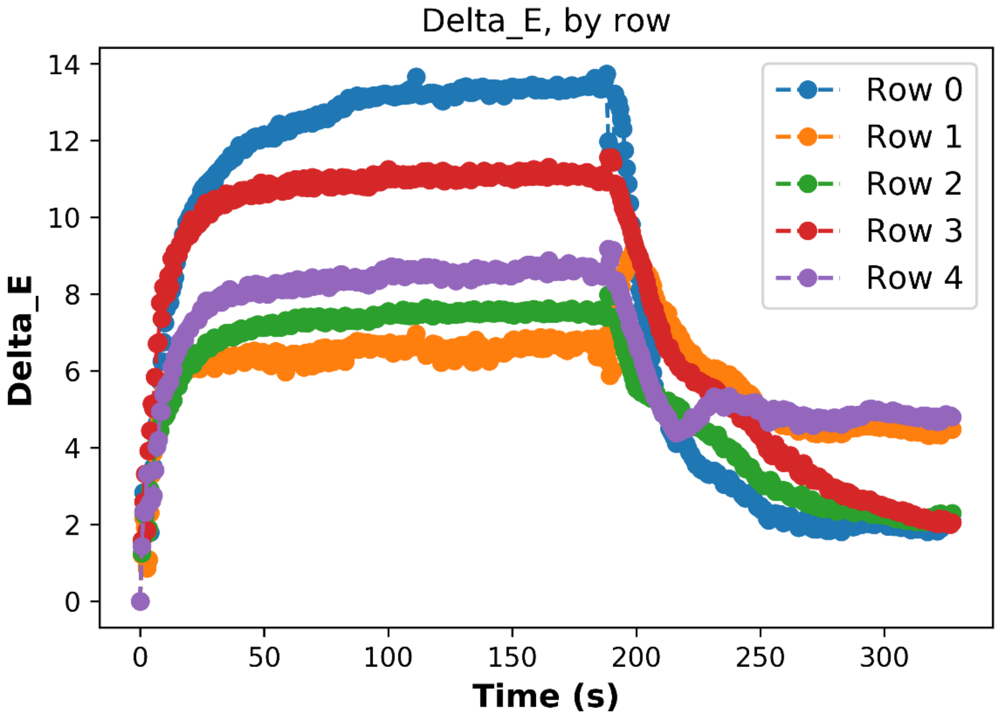
Delta_E, by row
Delta_E
Time (s)
Row 0
Row 1
Row 2
Row 3
Row 4
0
2
4
6
8
10
12
14
0
50
100
150
200
250
300

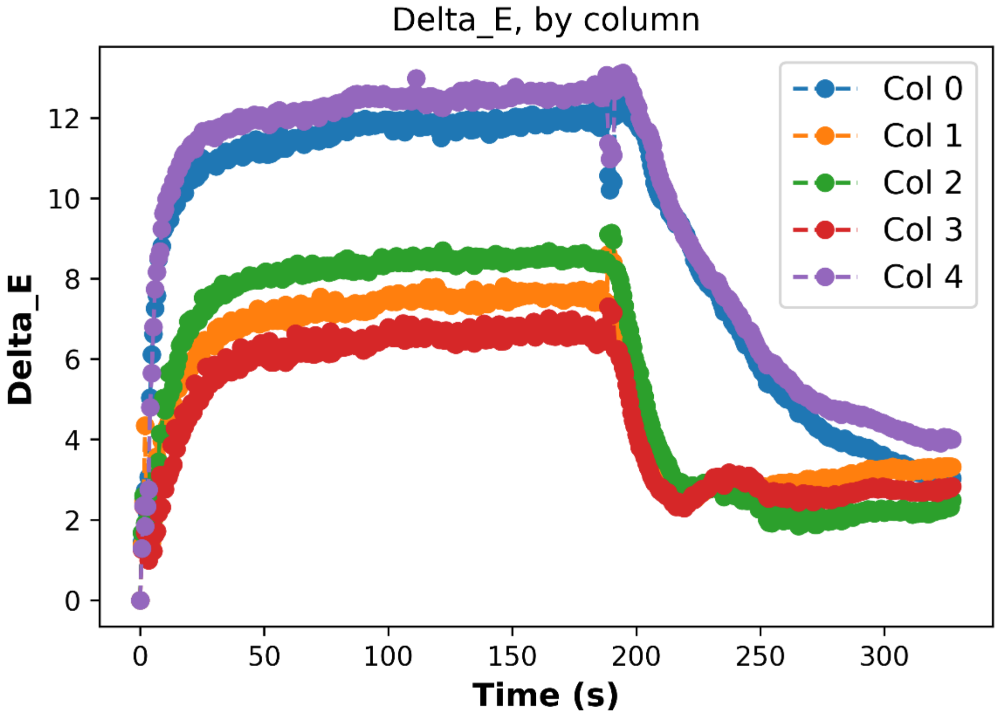
Delta_E, by column
Delta_E
Time (s)
Col 0
Col 1
Col 2
Col 3
Col 4
0
2
4
6
8
10
12
0
50
100
150
200
250
300

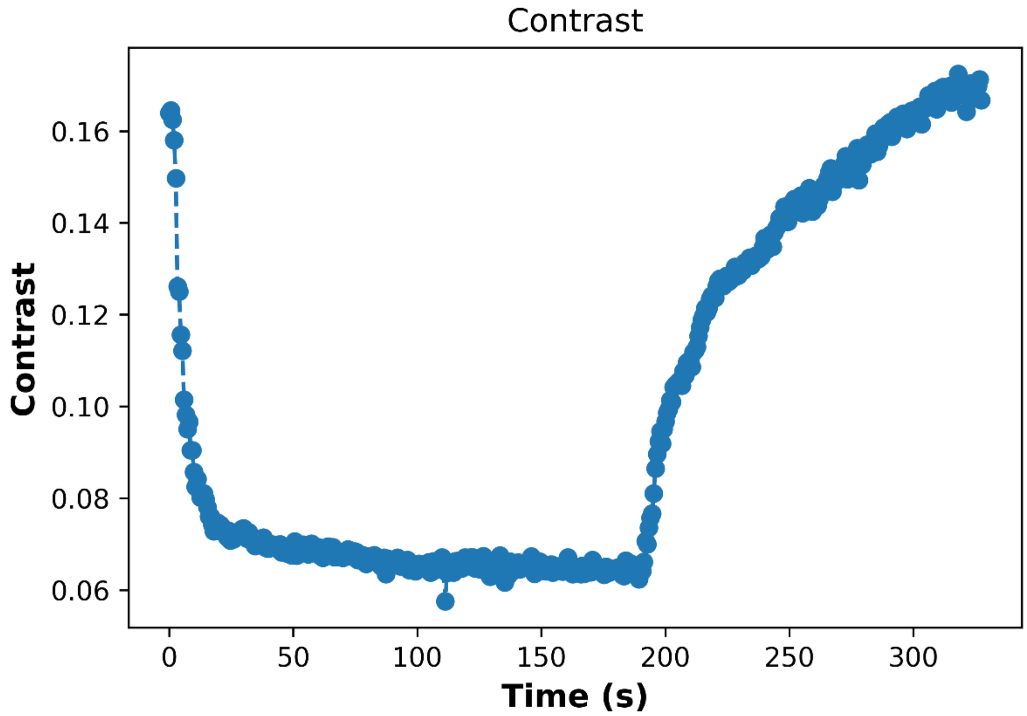
Contrast
Contrast
0.16
0.14
0.12
0.10
0.08
0.06
0
50
100
150
200
250
300
Time (s)

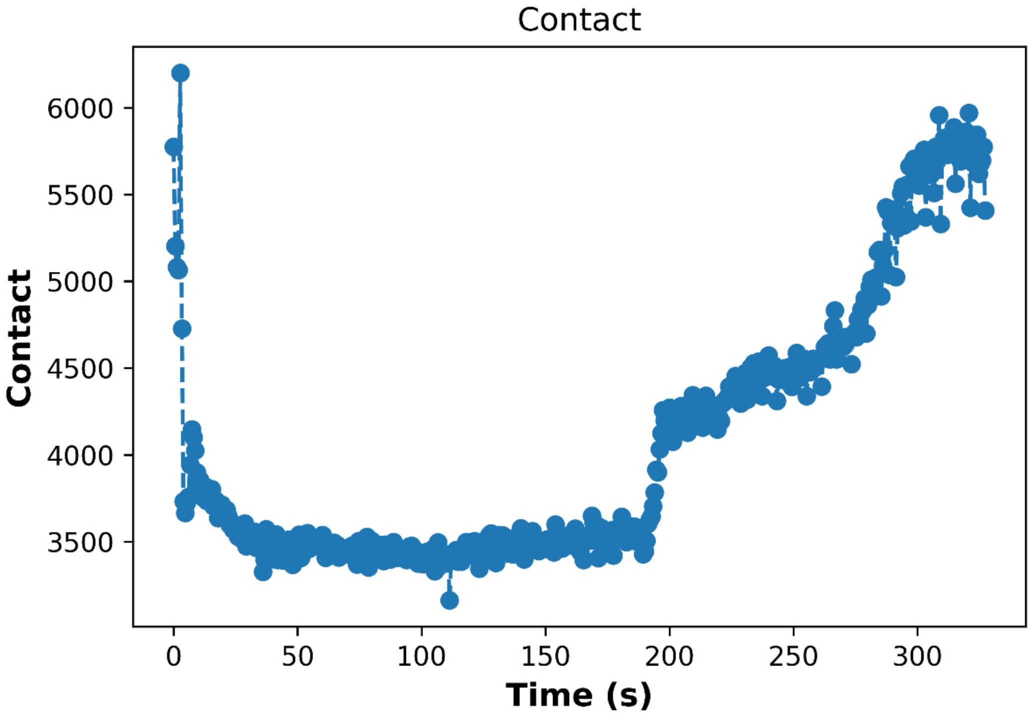
Contact
Contact
6000
5500
5000
4500
4000
3500
0
50
100
150
200
250
300
Time (s)

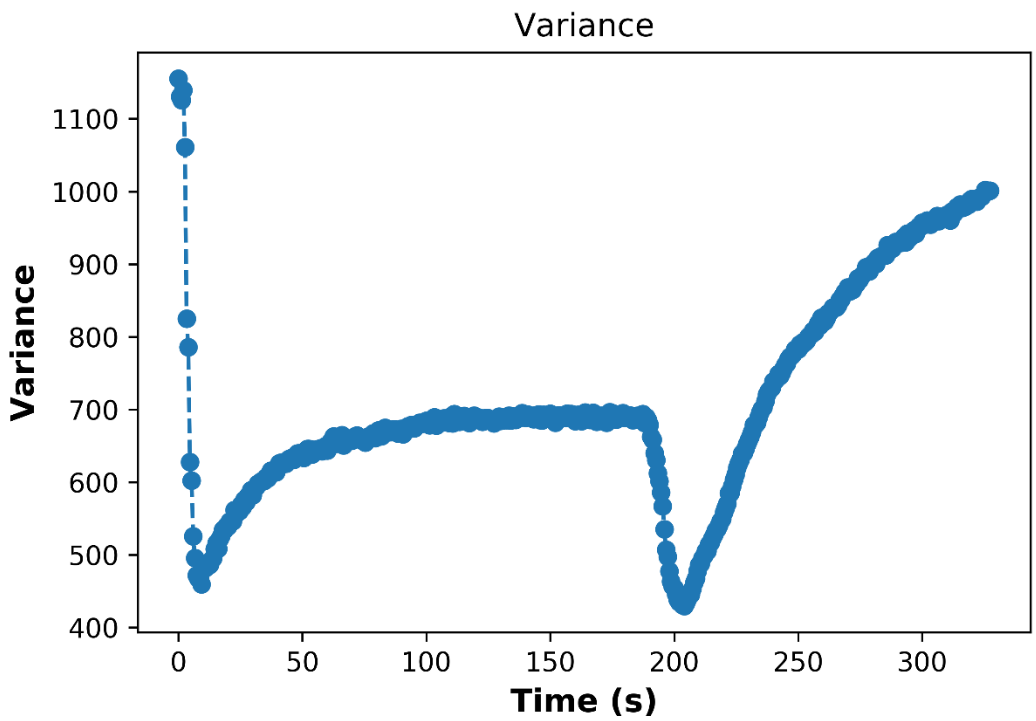

Exemplification of choosing different-colored vessel backgrounds

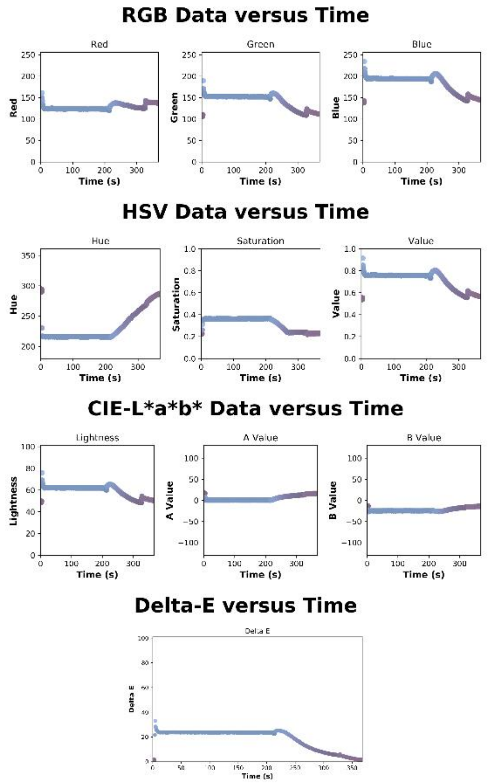

Kineticolor

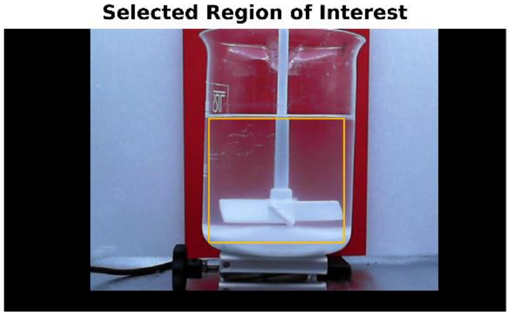

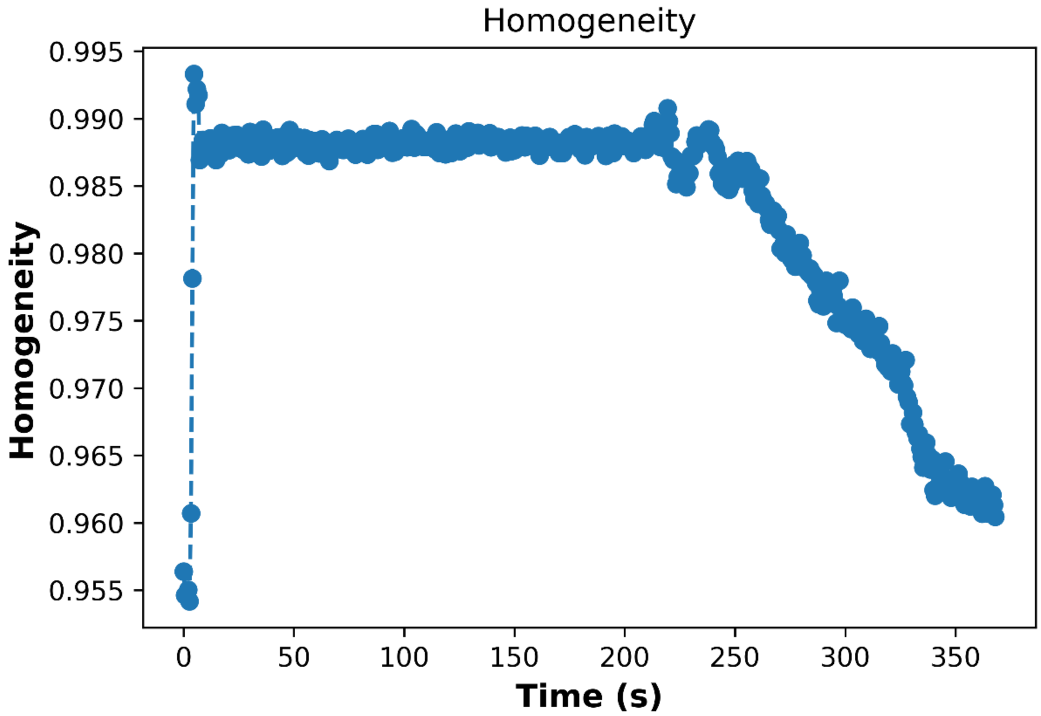
Homogeneity
Homogeneity
0.995
0.990
0.985
0.980
0.975
0.970
0.965
0.960
0.955
0
50
100
150
200
250
300
350
Time (s)

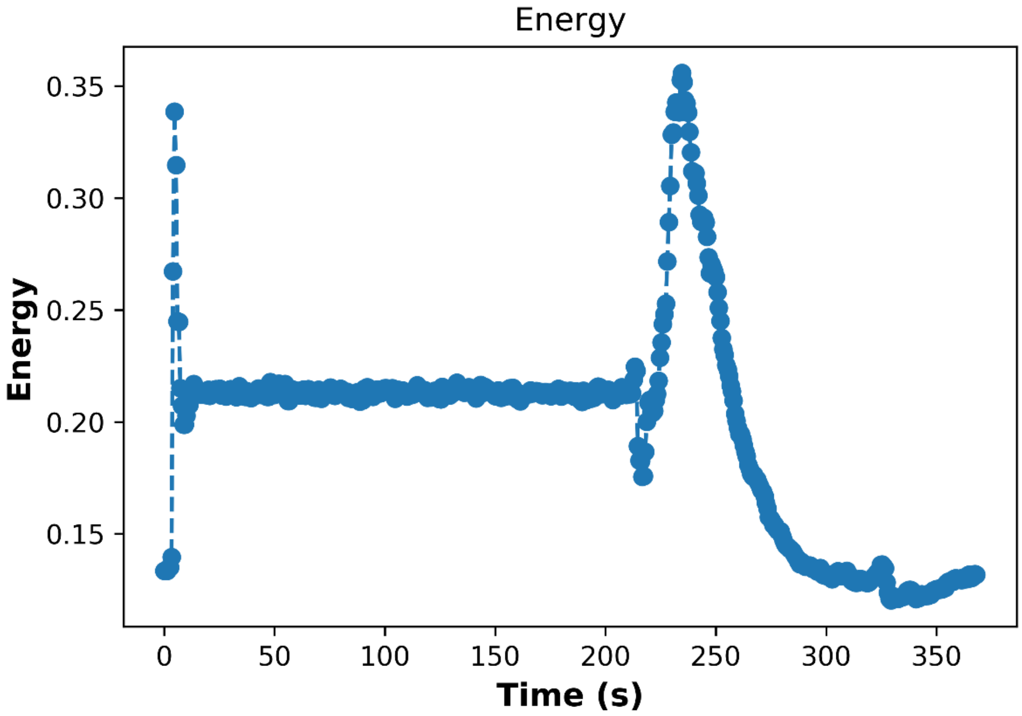
Energy
Energy
0.35
0.30
0.25
0.20
0.15
0
50
100
150
200
250
300
350
Time (s)

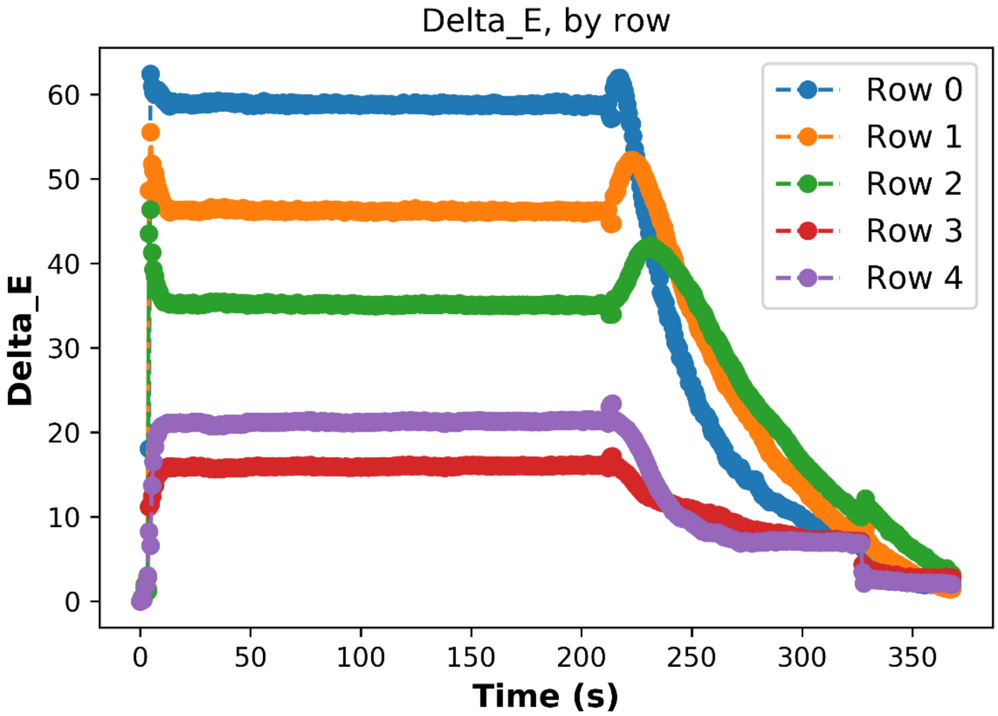

Delta_E, by row
Delta_E
Time (s)
Row 0
Row 1
Row 2
Row 3
Row 4
0
10
20
30
40
50
60
0
50
100
150
200
250
300
350

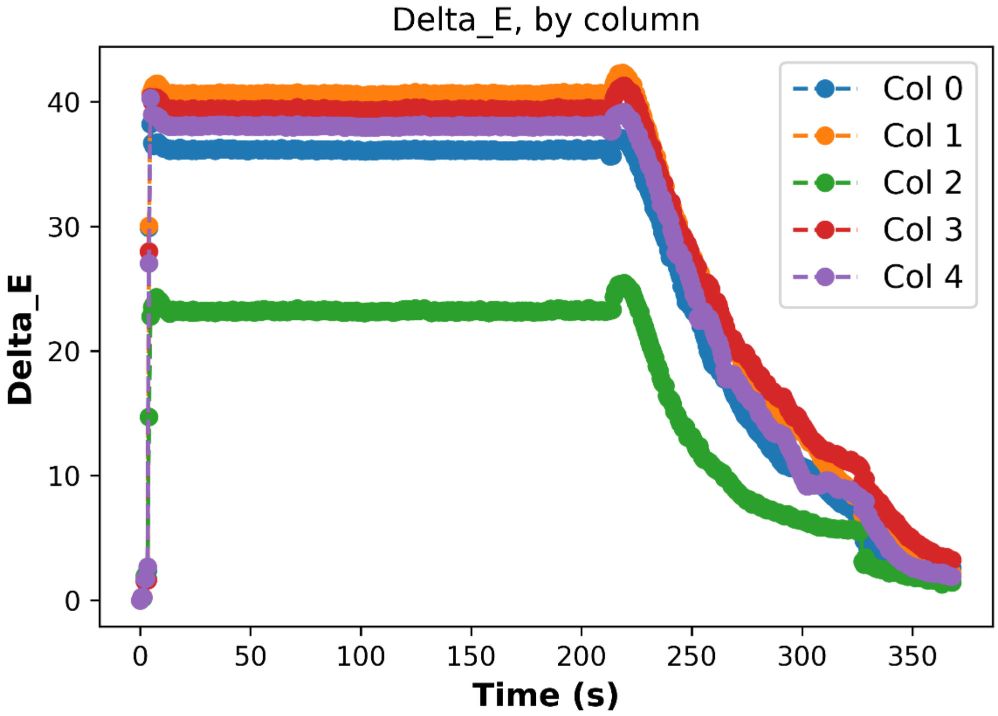

Delta_E, by column
Delta_E
Time (s)
Col 0
Col 1
Col 2
Col 3
Col 4
0
10
20
30
40
0
50
100
150
200
250
300
350

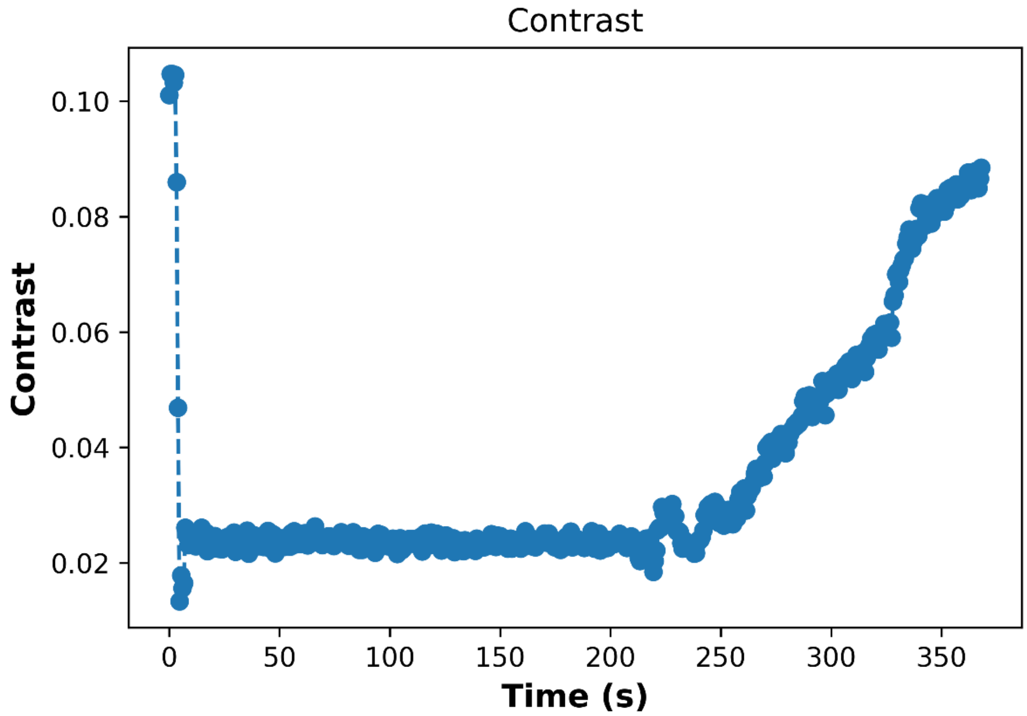
Contrast
0.10
0.08
0.06
0.04
0.02
0
50
100
150
200
250
300
350
Time (s)
Contrast

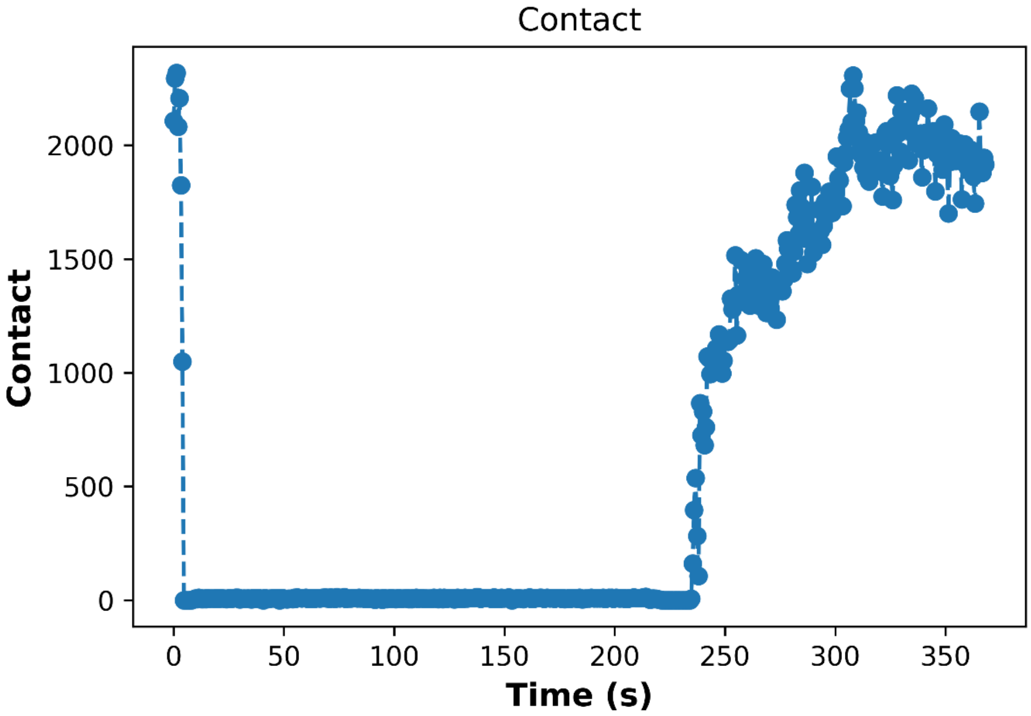
Contact
2000
1500
1000
500
0
0
50
100
150
200
250
300
350
Time (s)
Contact

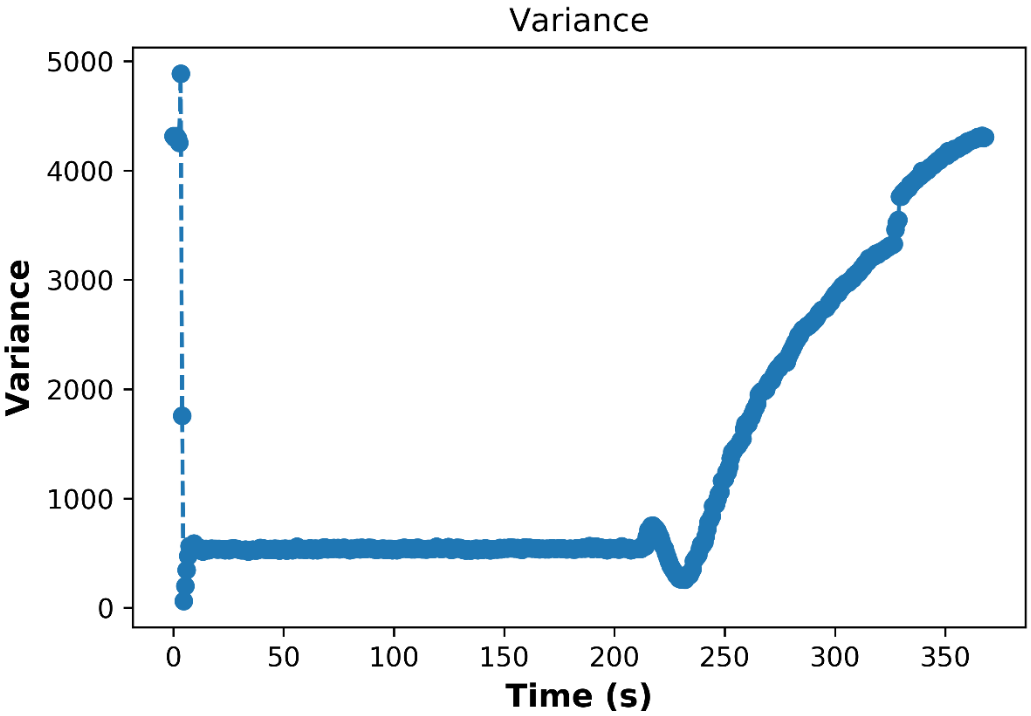

Variance
Variance
Time (s)
0
1000
2000
3000
4000
5000
0
50
100
150
200
250
300
350

5. pH Titrations as a Model System for Kinetic Imaging of Mixing Phenomena

5.1 Phenolphthalein titrations

Scheme 9 and 10 in the manuscript attend discussion on the analysis of legacy footage of titrations with acidified solutions of phenolphthalein and sodium hydroxide.

Below, the raw data for both videos – with and without baffles in the reactor – are provided.

phenolphthalein titration with no baffle in reactor

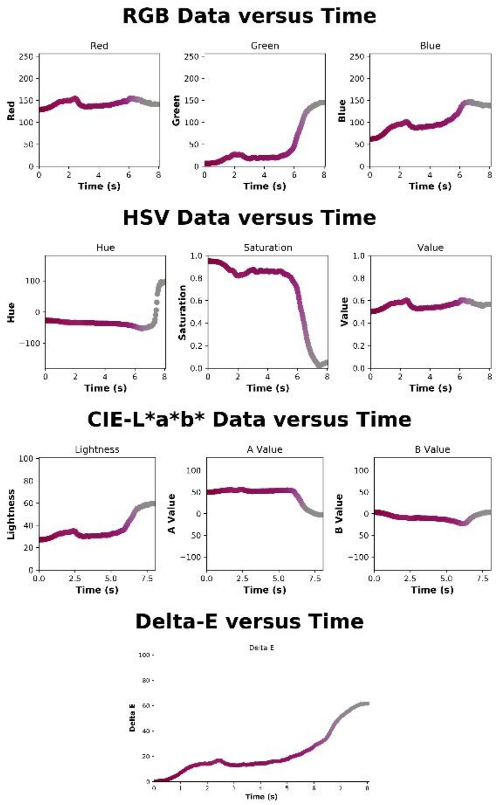

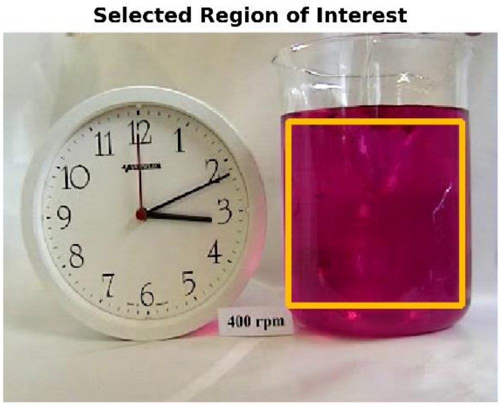

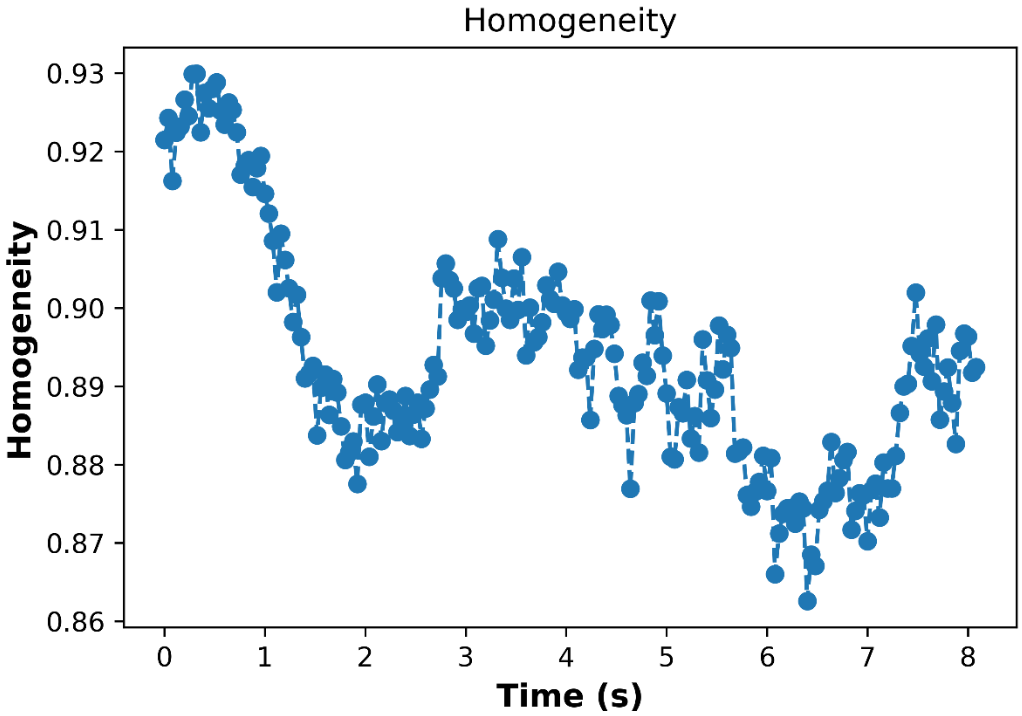

Homogeneity
Homogeneity
0.93
0.92
0.91
0.90
0.89
0.88
0.87
0.86
0
1
2
3
4
5
6
7
8
Time (s)

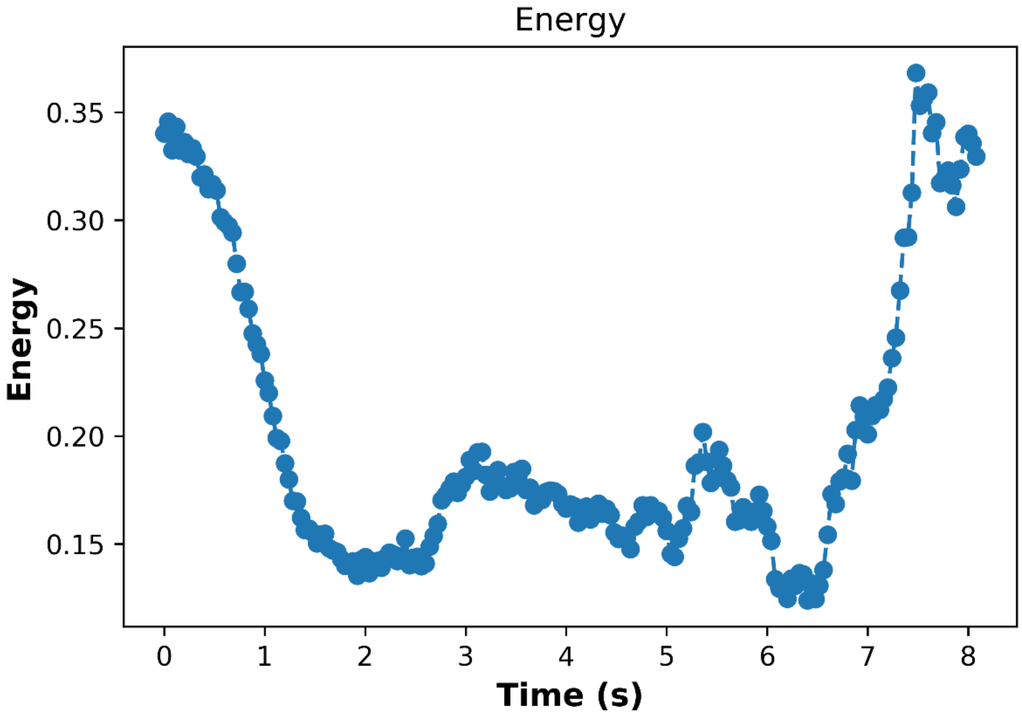

Energy
Energy
0.35
0.30
0.25
0.20
0.15
0
1
2
3
4
5
6
7
8
Time (s)

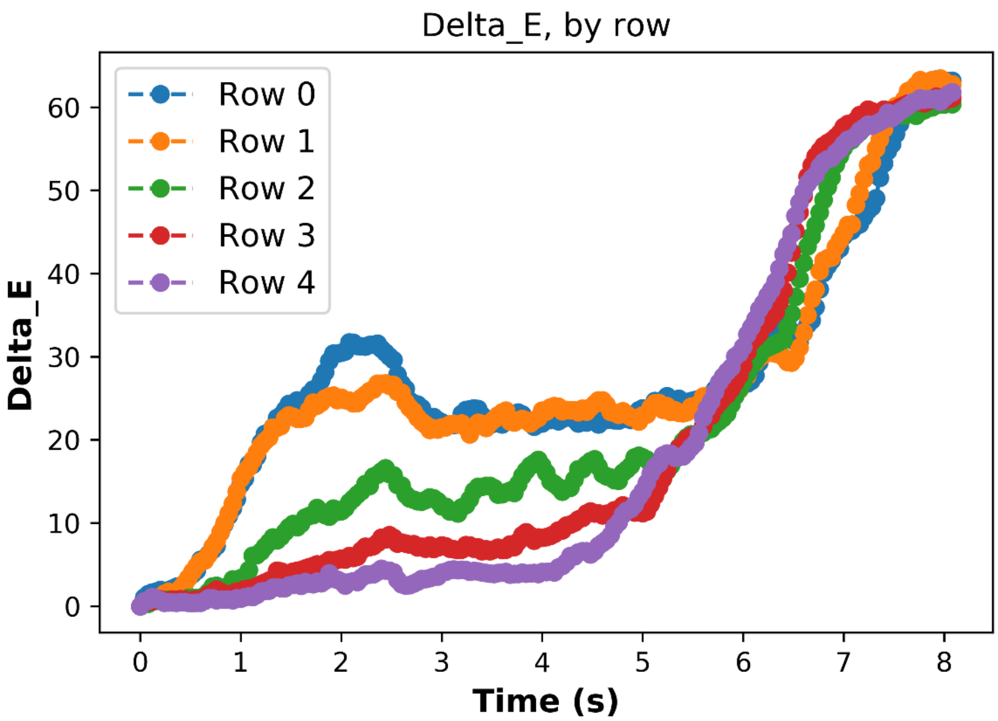
Delta_E, by row
Row 0
Row 1
Row 2
Row 3
Row 4
Delta_E
Time (s)
0
10
20
30
40
50
60
0
1
2
3
4
5
6
7
8

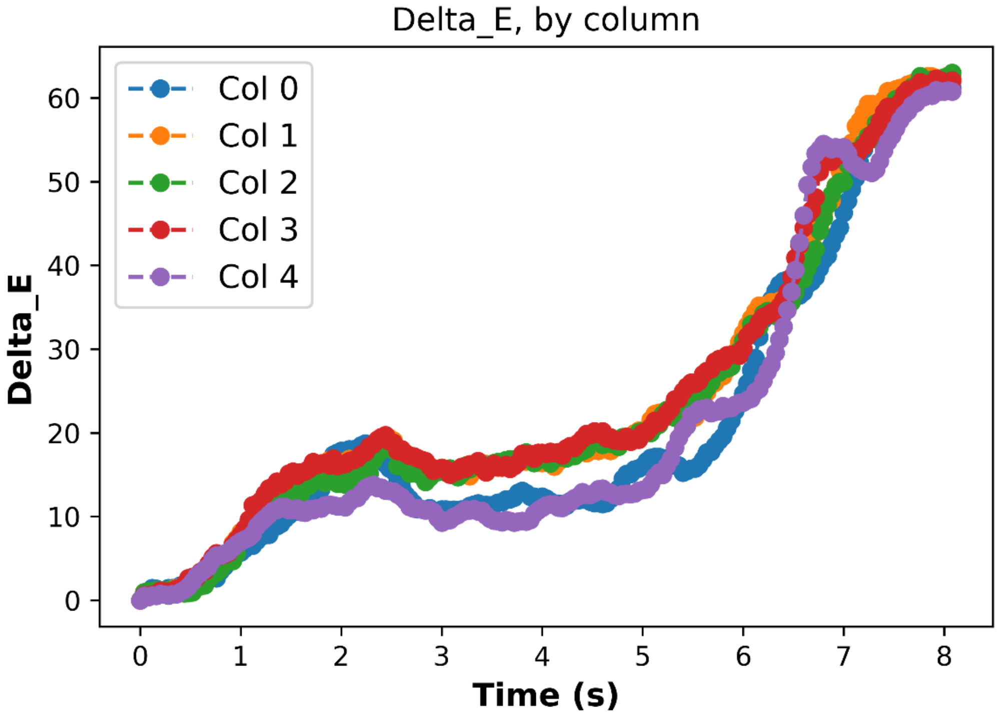
Delta_E, by column
Col 0
Col 1
Col 2
Col 3
Col 4
Delta_E
Time (s)
0
10
20
30
40
50
60
0
1
2
3
4
5
6
7
8

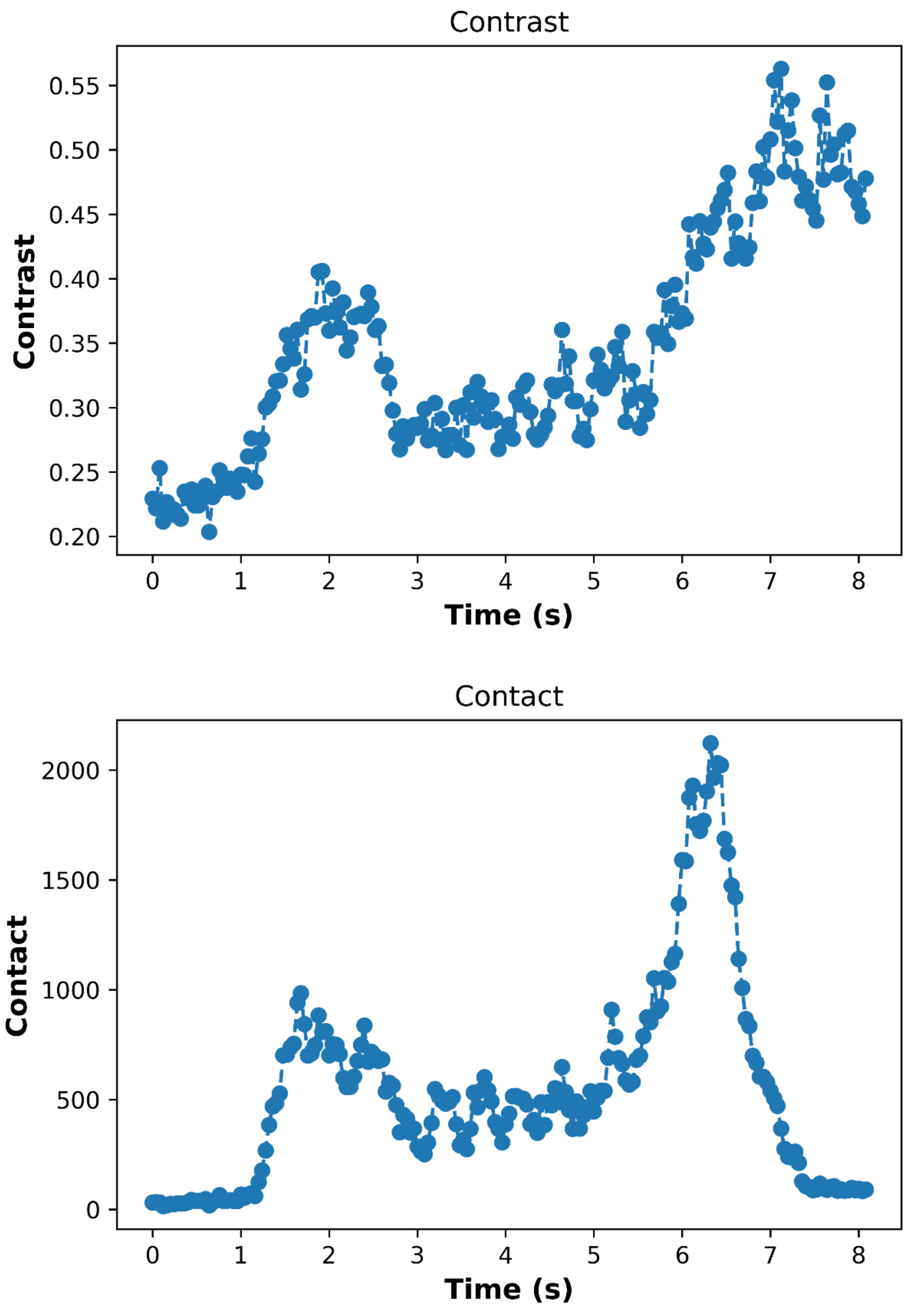

Contrast
0.55
0.50
0.45
0.40
0.35
0.30
0.25
0.20
Contrast
0
1
2
3
4
5
6
7
8
Time (s)
Contact
2000
1500
1000
500
0
Contact
0
1
2
3
4
5
6
7
8
Time (s)

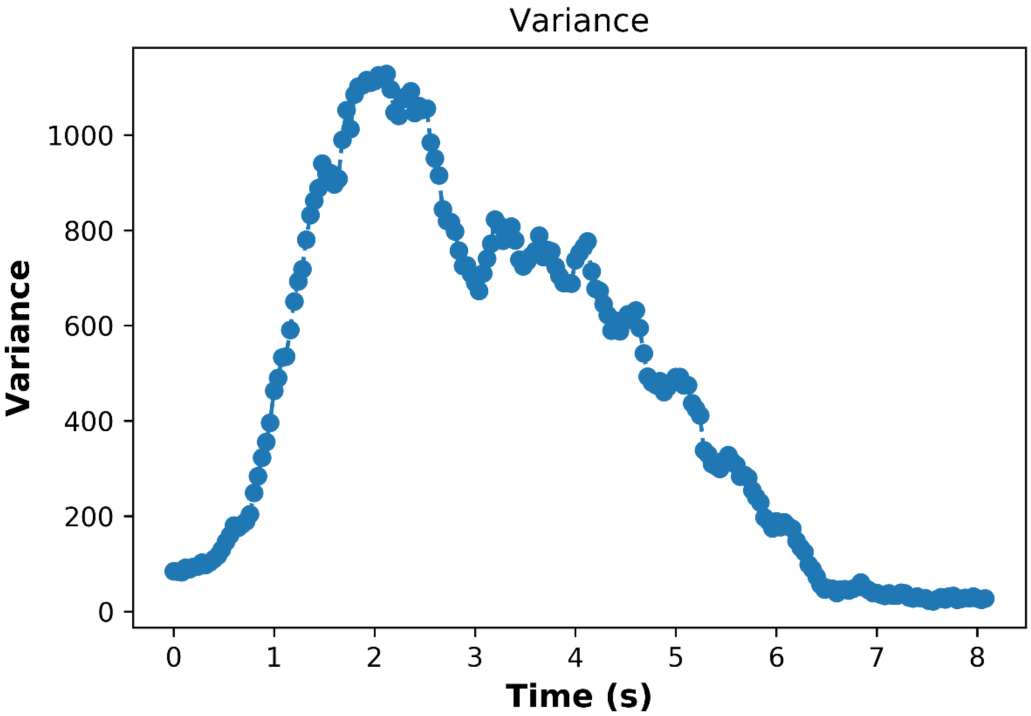

Variance
Variance
1000
800
600
400
200
0
0
1
2
3
4
5
6
7
8
Time (s)

phenolphthalein titration with baffle in reactor

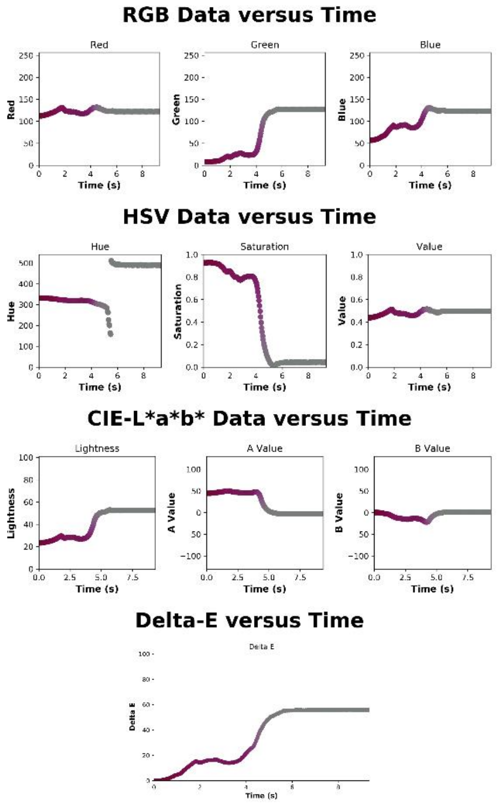

Kineticolor

Selected Region of Interest

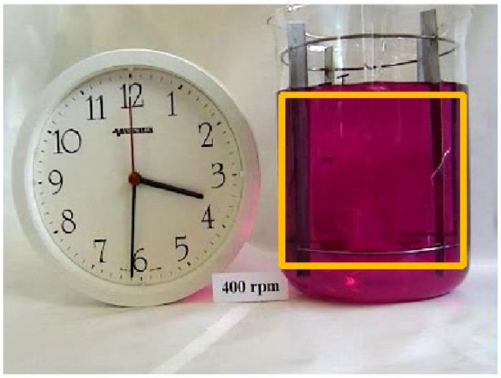

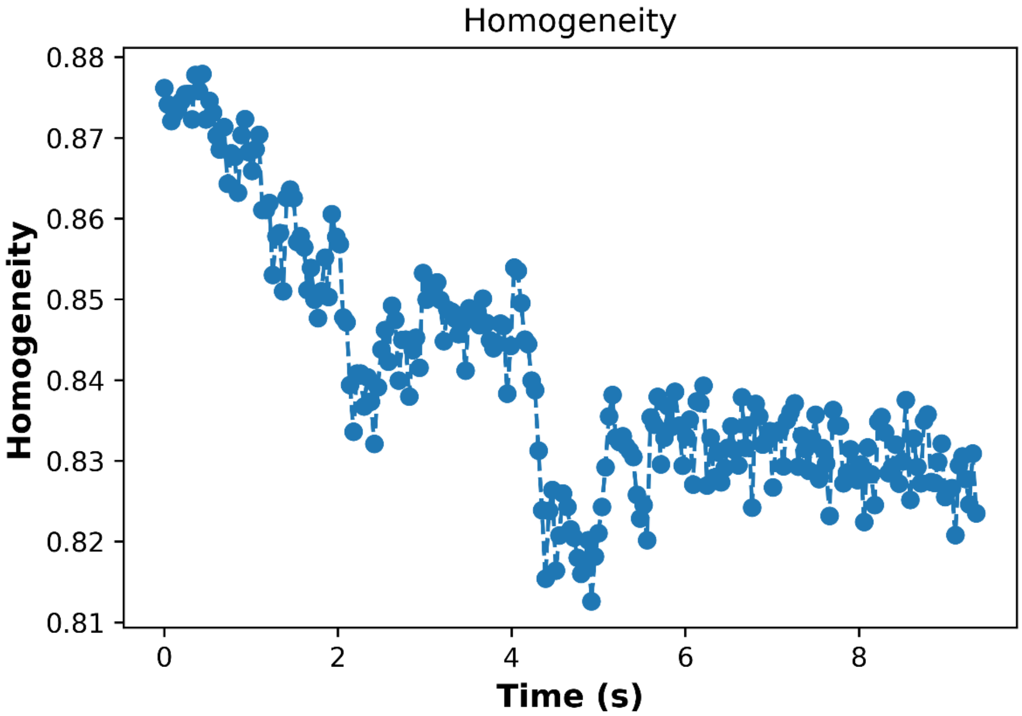

Homogeneity
0.88
0.87
0.86
0.85
0.84
0.83
0.82
0.81
Homogeneity
0
2
4
6
8
Time (s)

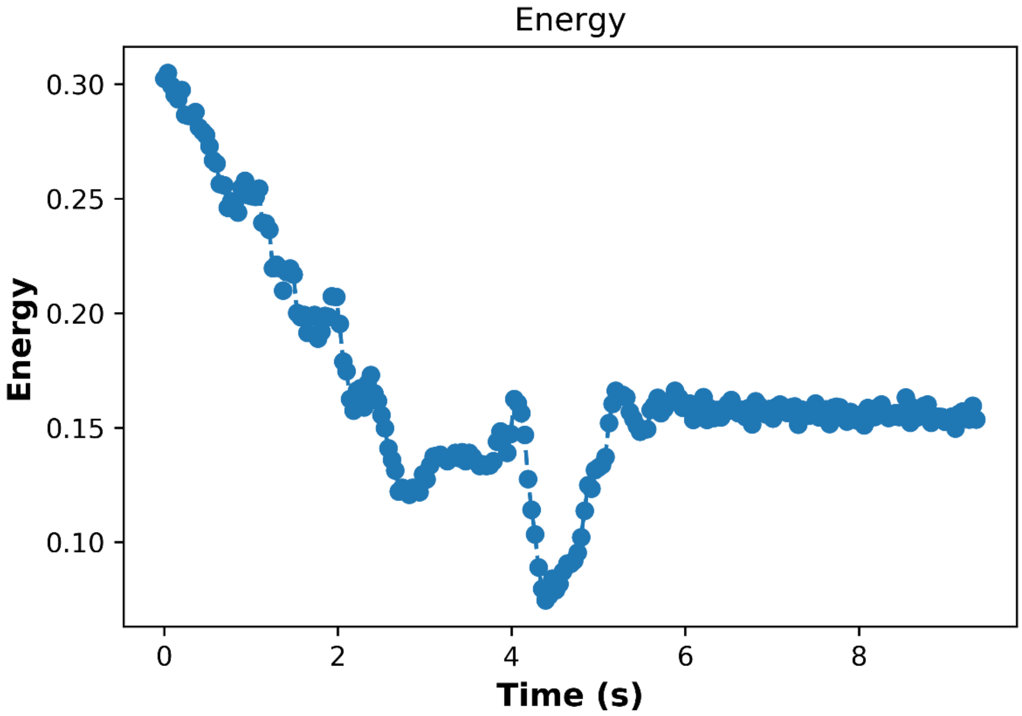

Energy
0.30
0.25
0.20
0.15
0.10
Energy
0
2
4
6
8
Time (s)

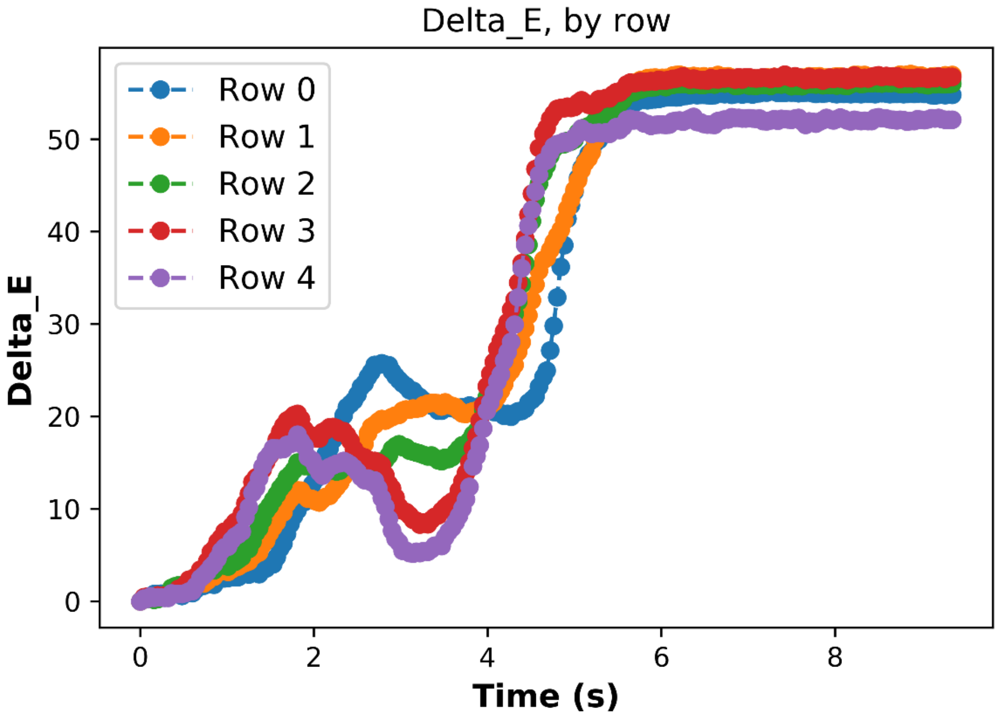
Delta_E, by row
Row 0
Row 1
Row 2
Row 3
Row 4
Delta_E
Time (s)
0
10
20
30
40
50
0
2
4
6
8

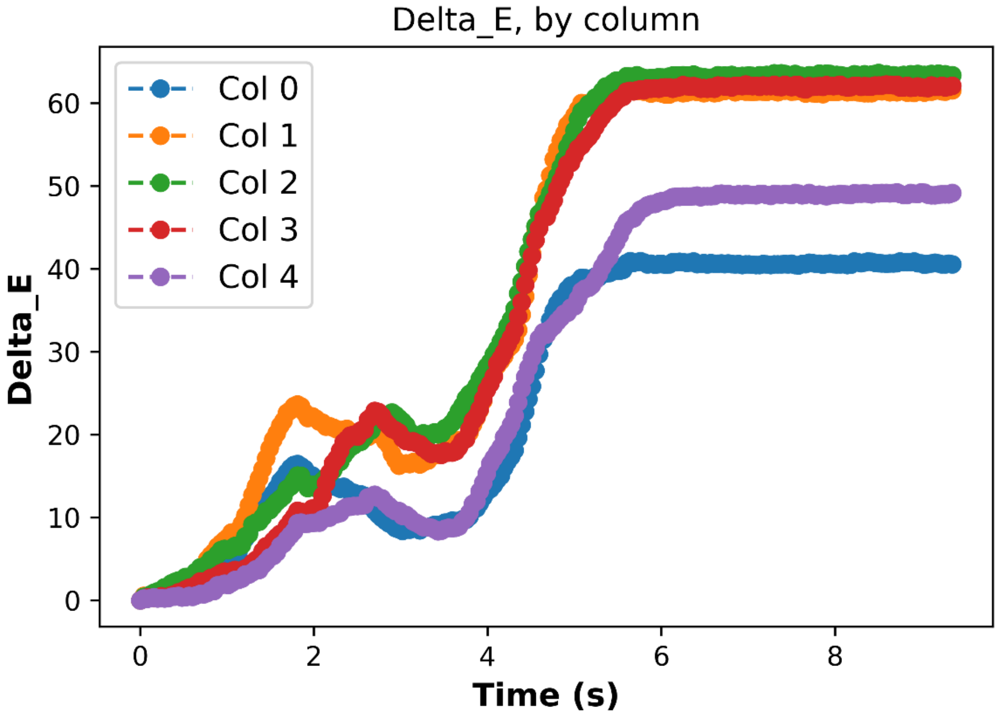
Delta_E, by column
Col 0
Col 1
Col 2
Col 3
Col 4
Delta_E
Time (s)
0
10
20
30
40
50
60
0
2
4
6
8

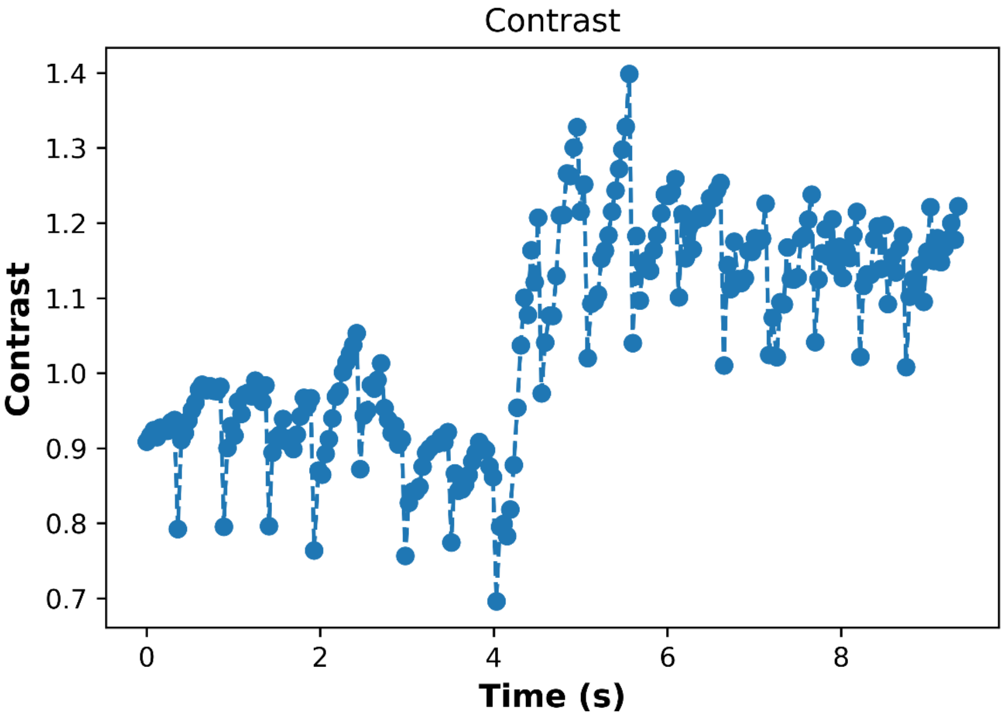
Contrast
1.4
1.3
1.2
1.1
1.0
0.9
0.8
0.7
Contrast
0
2
4
6
8
Time (s)

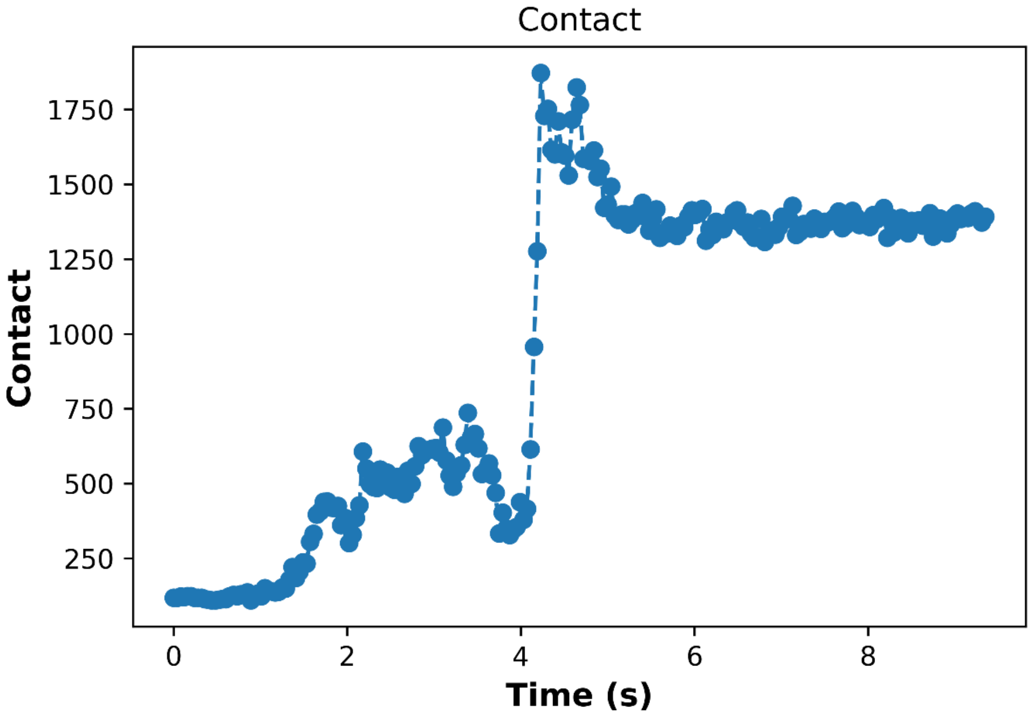
Contact
1750
1500
1250
1000
750
500
250
Contact
0
2
4
6
8
Time (s)

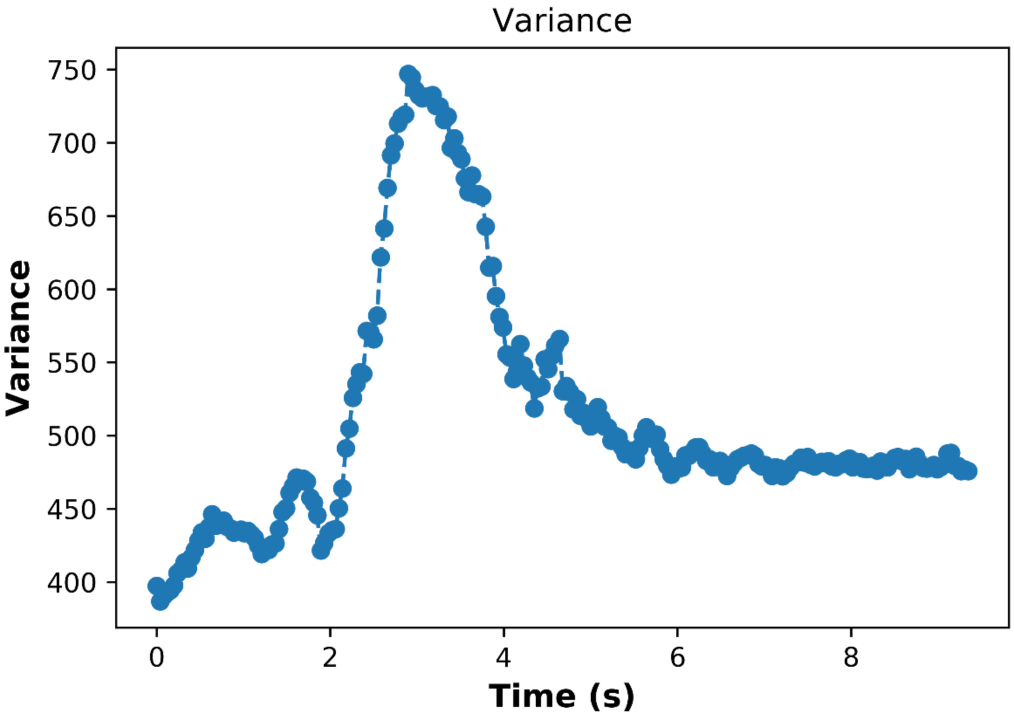

Variance
750
700
650
600
550
500
450
400
Variance
0
2
4
6
8
Time (s)

5.2 Bromothymol blue titrations

Schemes 11 and 12 in the manuscript attend additional titrations involving basified bromothymol blue and aqueous hydrochloric acid.

Below, raw data for each reaction – in Schlenk tubes, round bottom flasks, beakers, and STRs – is provided.

50 mL Schlenk tube, large oval stirrer bar (15x6 mm)

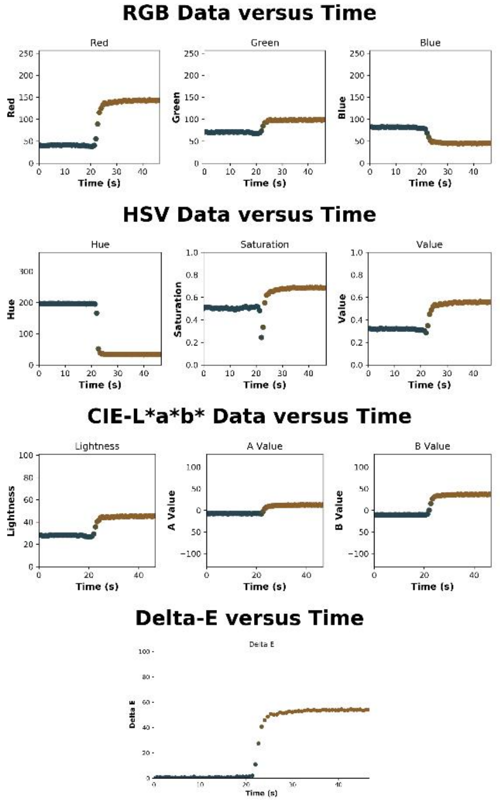

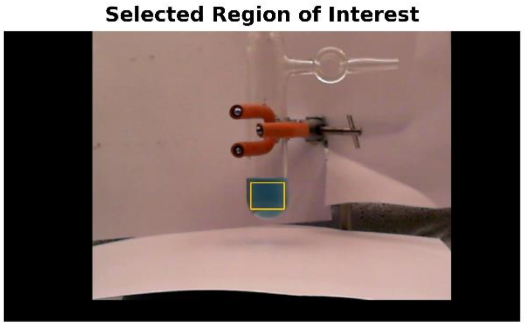

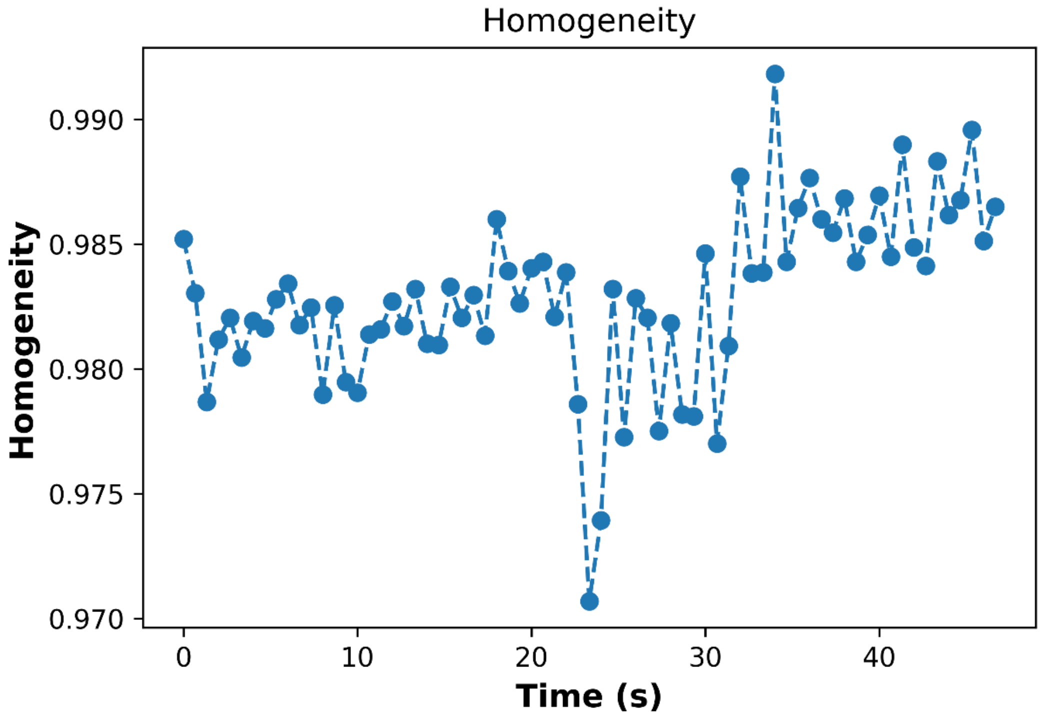
Homogeneity
Homogeneity
0.990
0.985
0.980
0.975
0.970
0
10
20
30
40
Time (s)

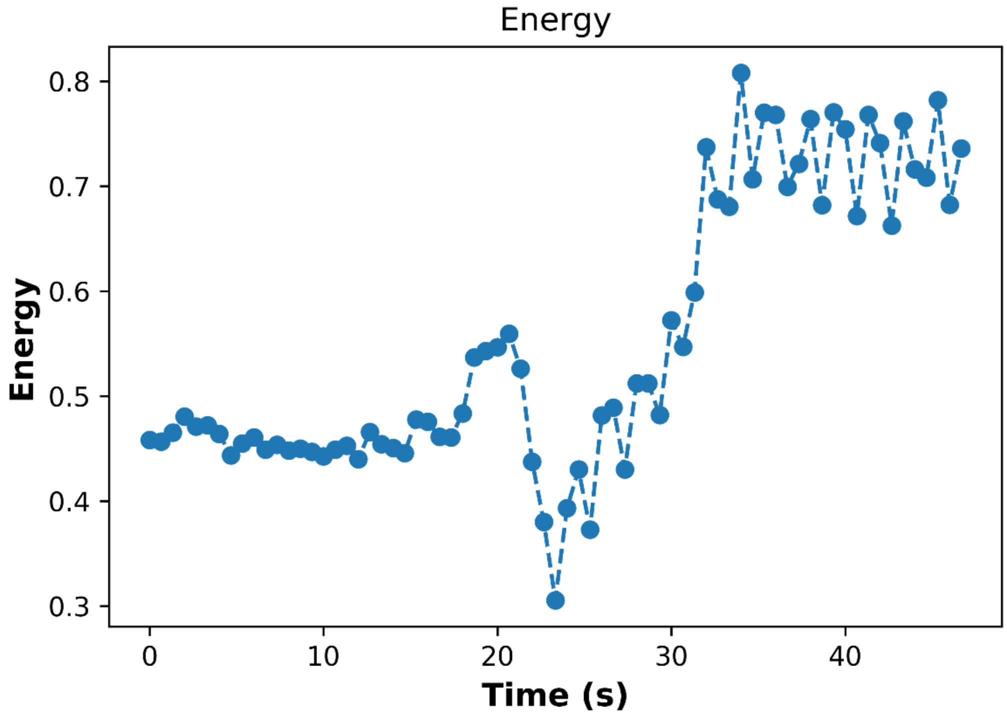
Energy
Energy
0.8
0.7
0.6
0.5
0.4
0.3
0
10
20
30
40
Time (s)

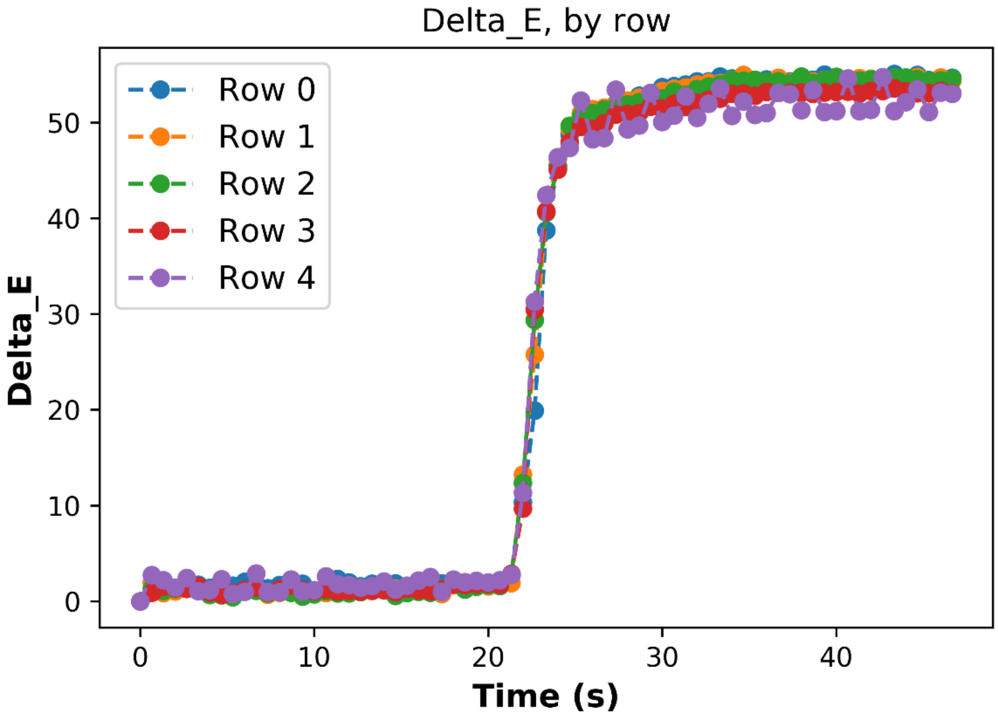
Delta_E, by row
Row 0
Row 1
Row 2
Row 3
Row 4
Delta_E
Time (s)
0
10
20
30
40
50

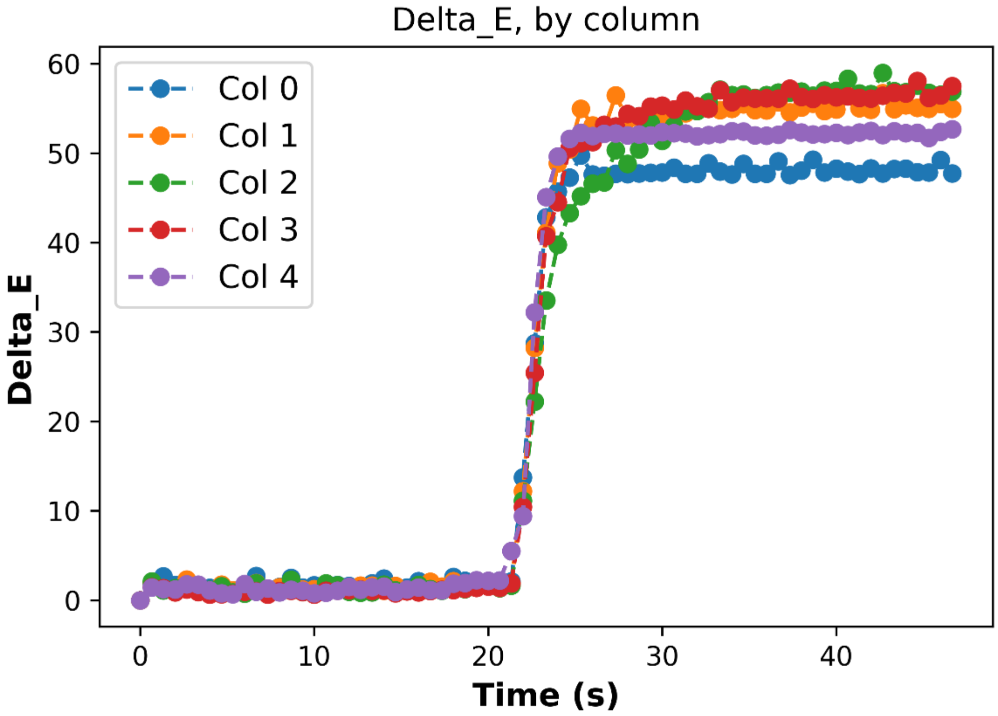
Delta_E, by column
Col 0
Col 1
Col 2
Col 3
Col 4
Delta_E
Time (s)
0
10
20
30
40
50
60

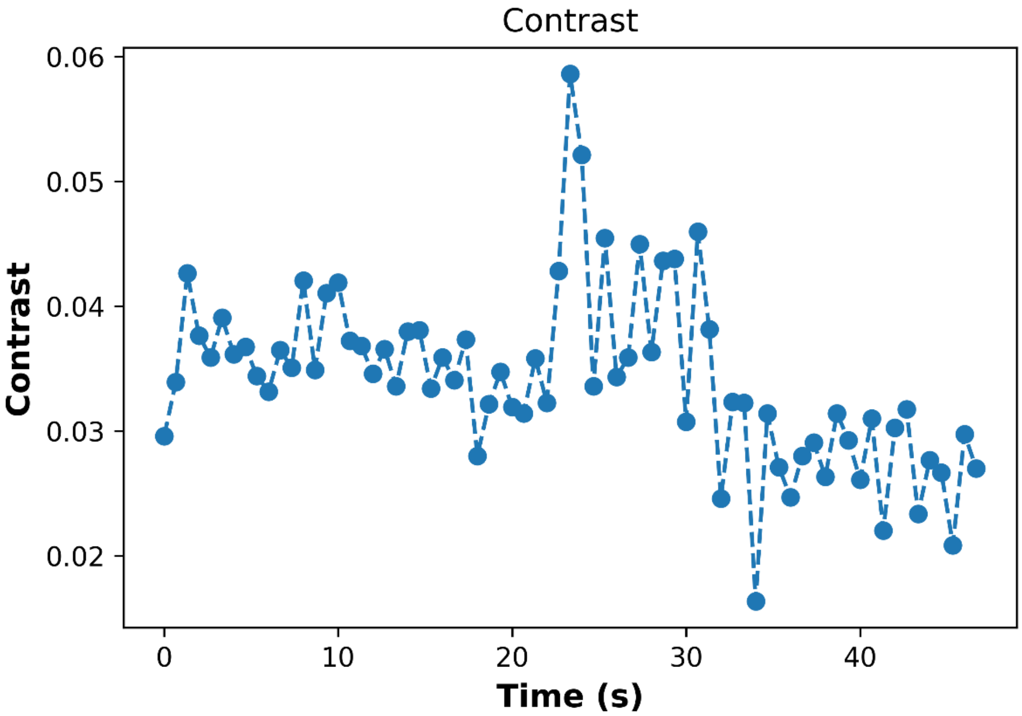
Contrast
0.06
0.05
0.04
0.03
0.02
Contrast
0
10
20
30
40
Time (s)

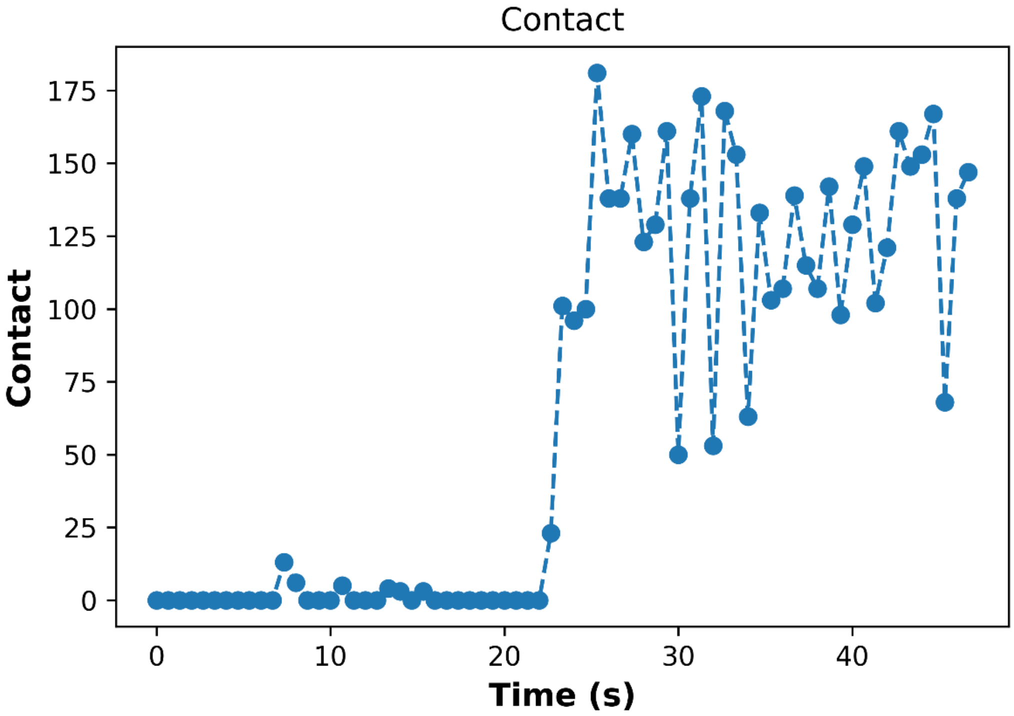
Contact
175
150
125
100
75
50
25
0
Contact
0
10
20
30
40
Time (s)

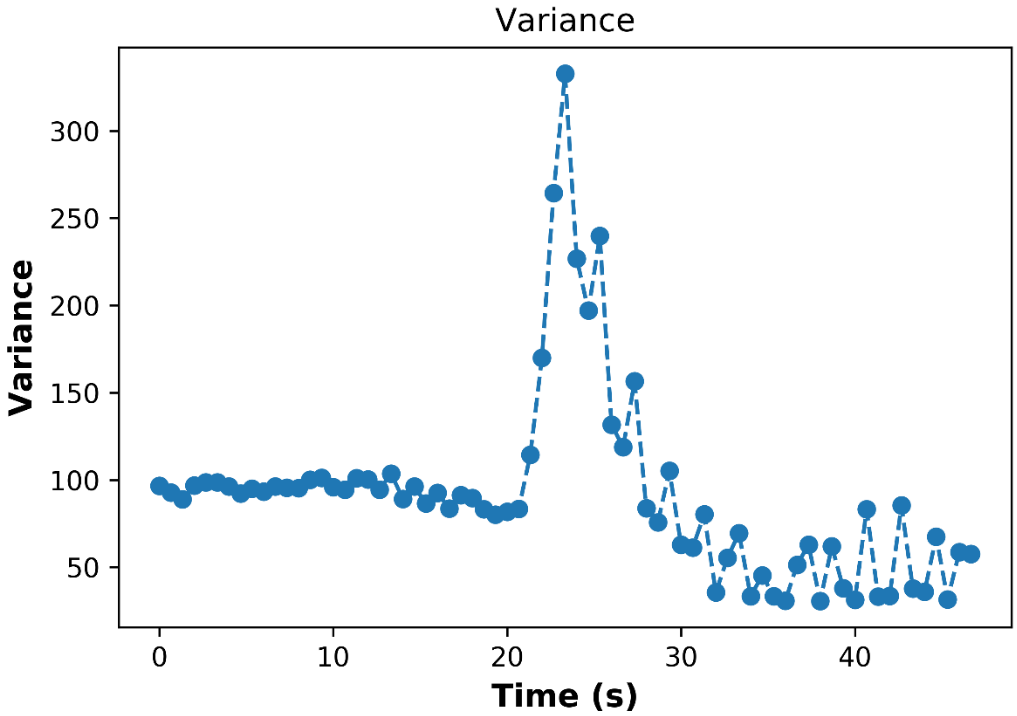
Variance
Variance
300
250
200
150
100
50
0
10
20
30
40
Time (s)

50 mL Schlenk tube, micro-stirrer bar (5x2 mm)

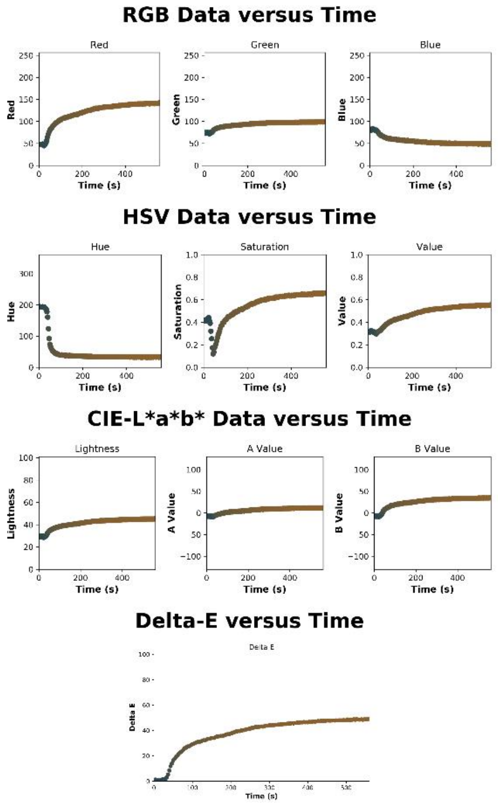

Kineticolor

Selected Region of Interest

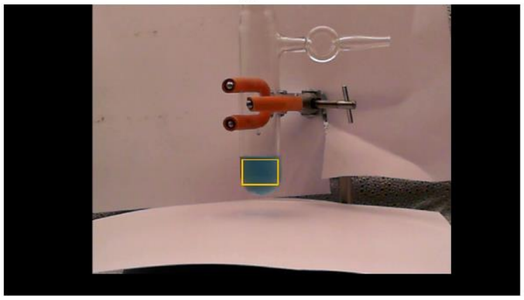

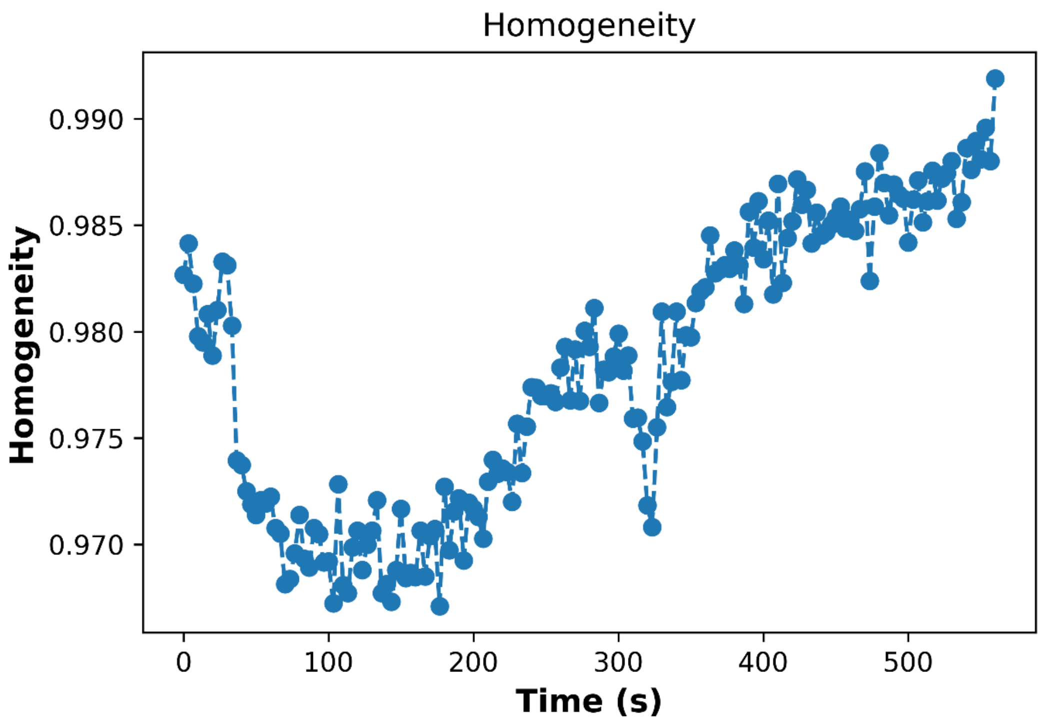

Homogeneity
0.990
0.985
0.980
0.975
0.970
Homogeneity
0
100
200
300
400
500
Time (s)

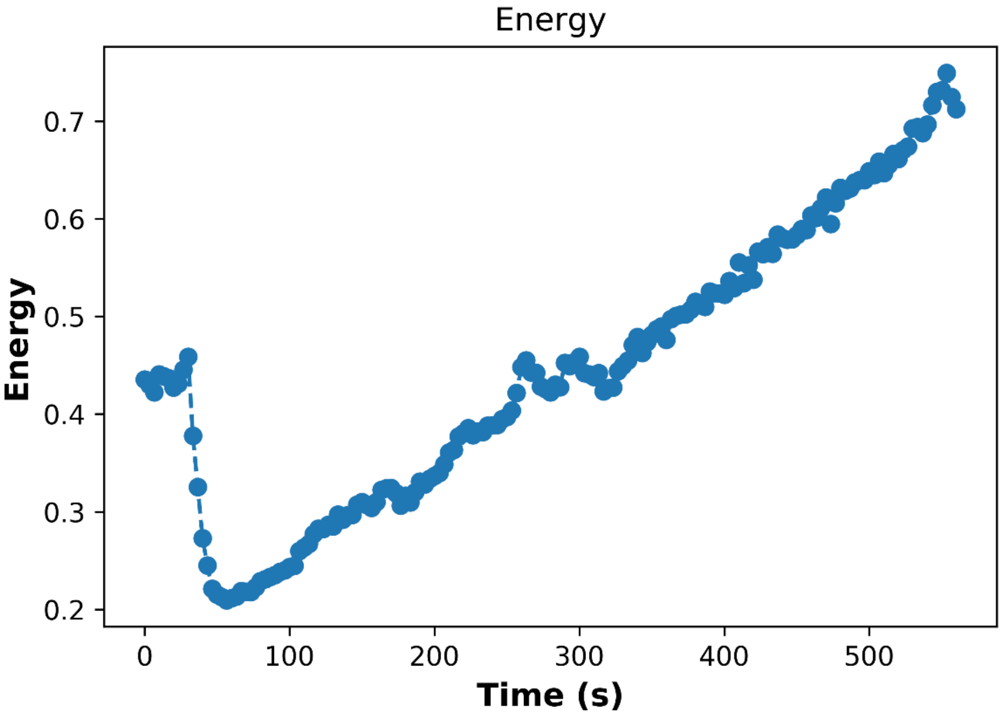

Energy
0.7
0.6
0.5
0.4
0.3
0.2
Energy
0
100
200
300
400
500
Time (s)

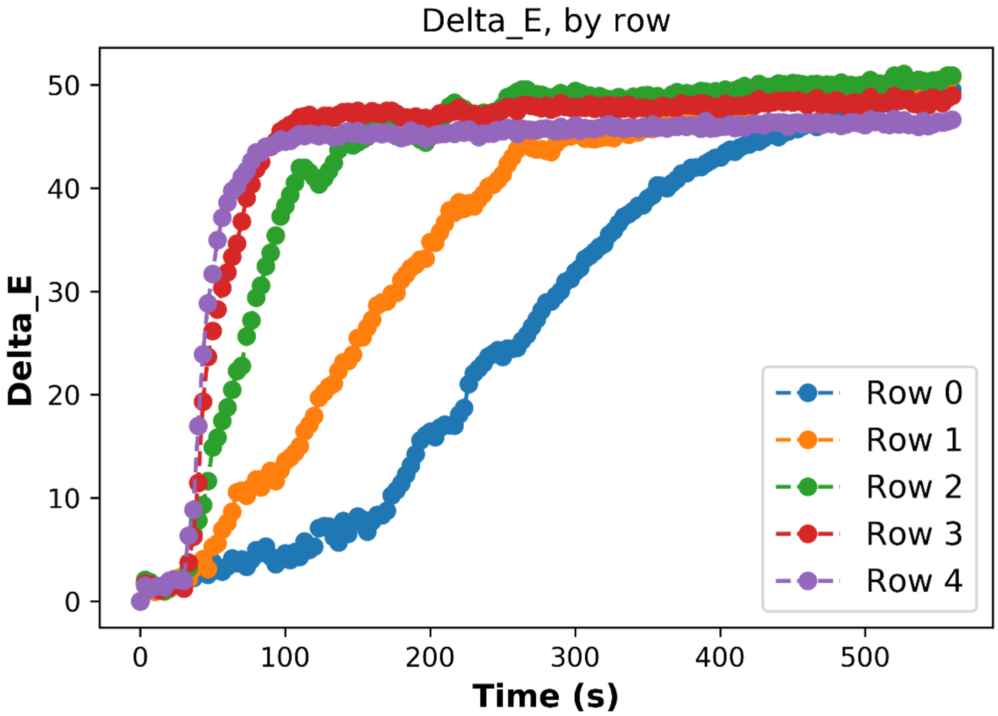
Delta_E, by row
Delta_E
Time (s)
Row 0
Row 1
Row 2
Row 3
Row 4
0
10
20
30
40
50
0
100
200
300
400
500

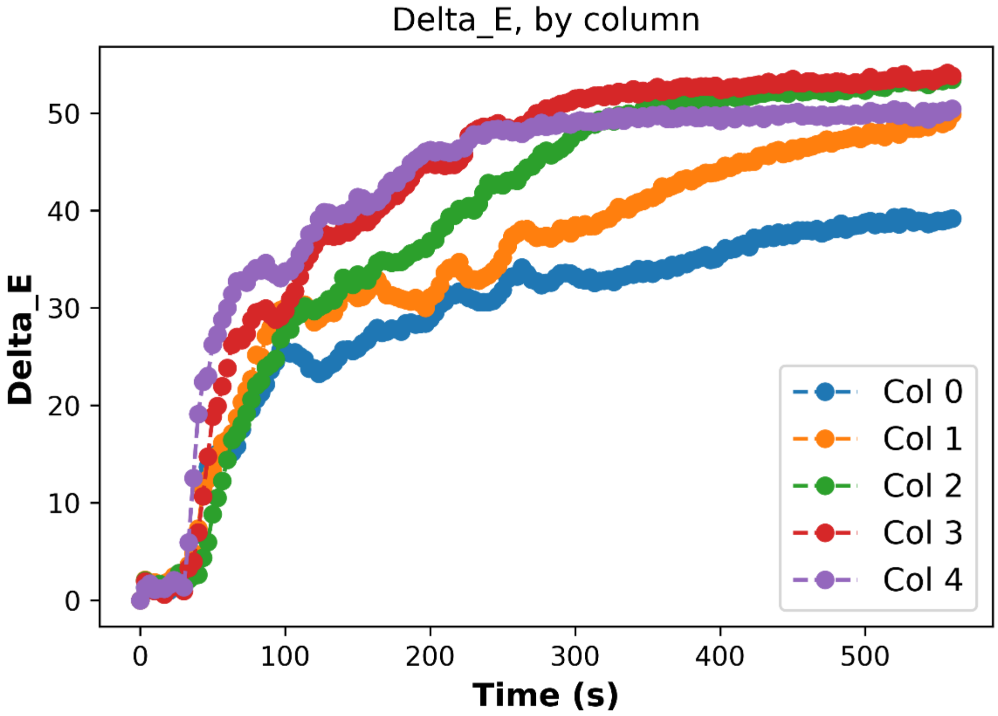
Delta_E, by column
Delta_E
Time (s)
Col 0
Col 1
Col 2
Col 3
Col 4
0
10
20
30
40
50
0
100
200
300
400
500

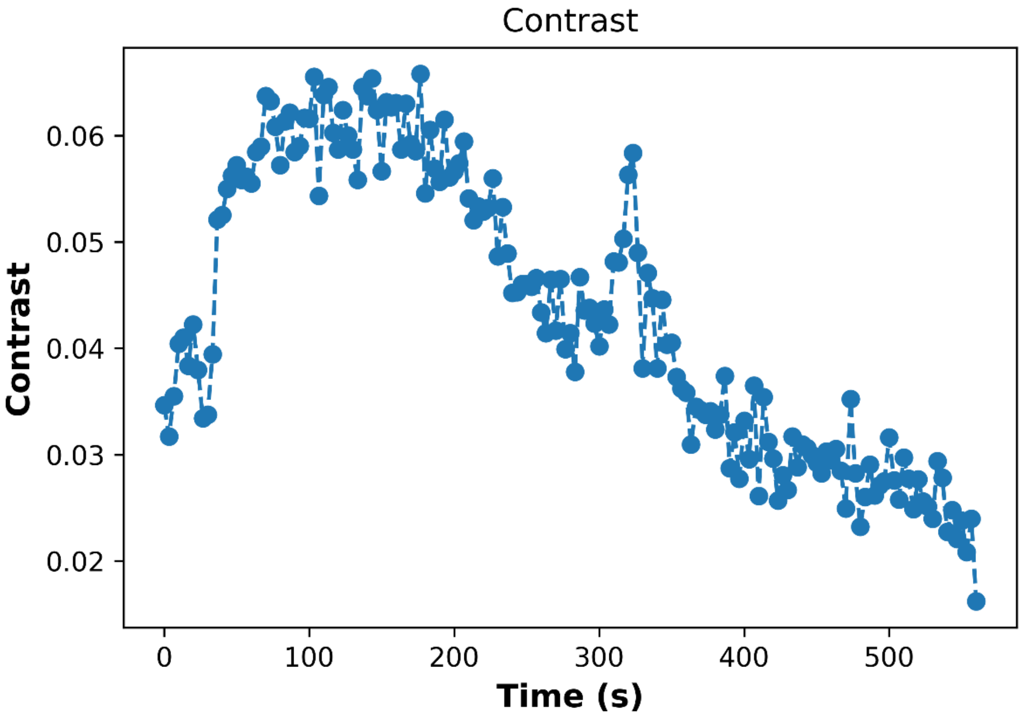
Contrast
0.06
0.05
0.04
0.03
0.02
Contrast
0
100
200
300
400
500
Time (s)

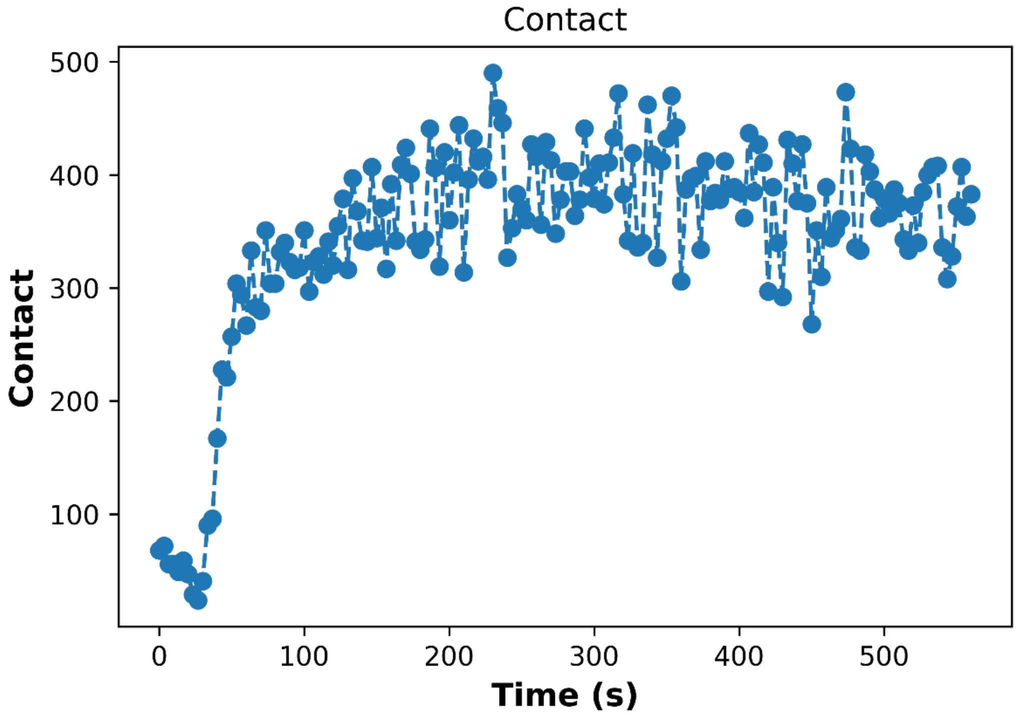
Contact
500
400
300
200
100
Contact
0
100
200
300
400
500
Time (s)

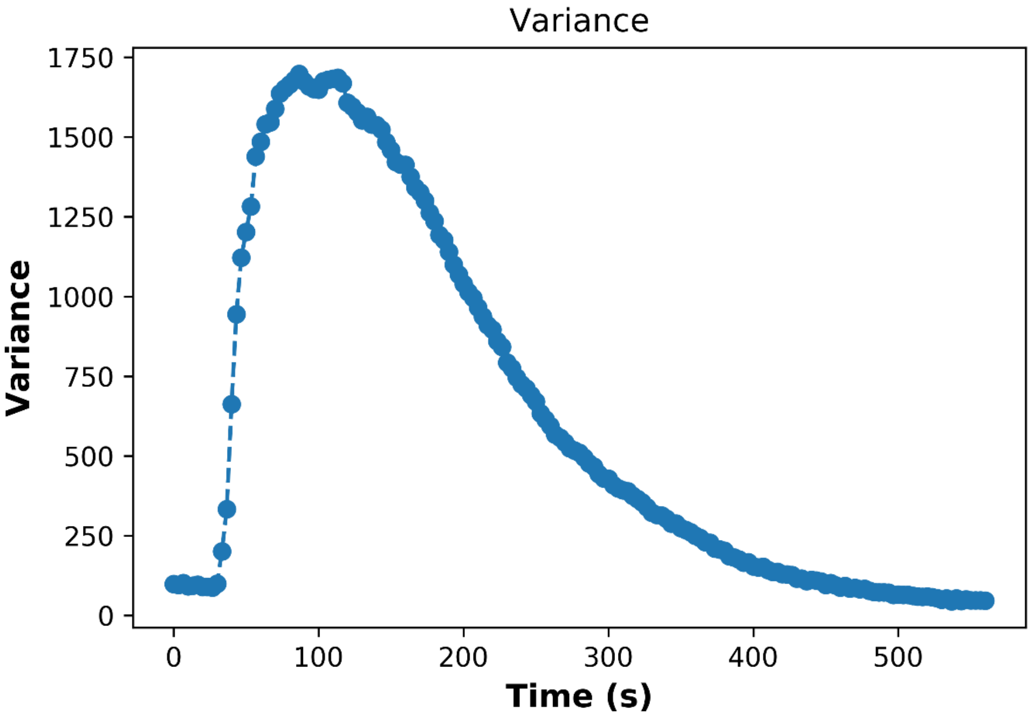
Variance
1750
1500
1250
1000
750
500
250
0
Variance
0
100
200
300
400
500
Time (s)

50 mL round bottom flask, large oval stirrer bar (15x6 mm)

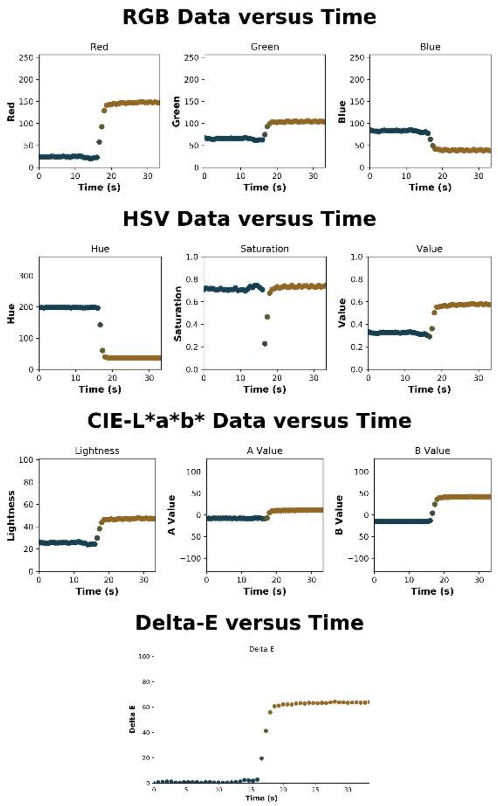

Kineticolor

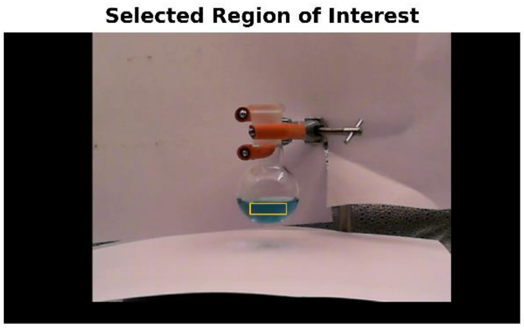

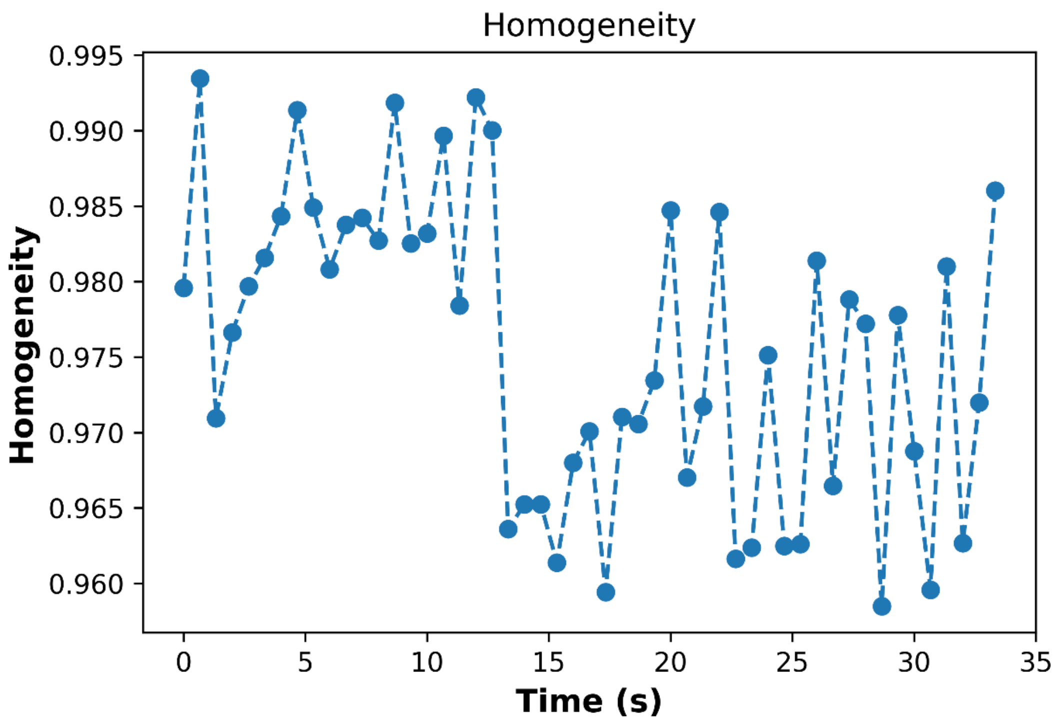

Homogeneity
Homogeneity
0.995
0.990
0.985
0.980
0.975
0.970
0.965
0.960
0
5
10
15
20
25
30
35
Time (s)

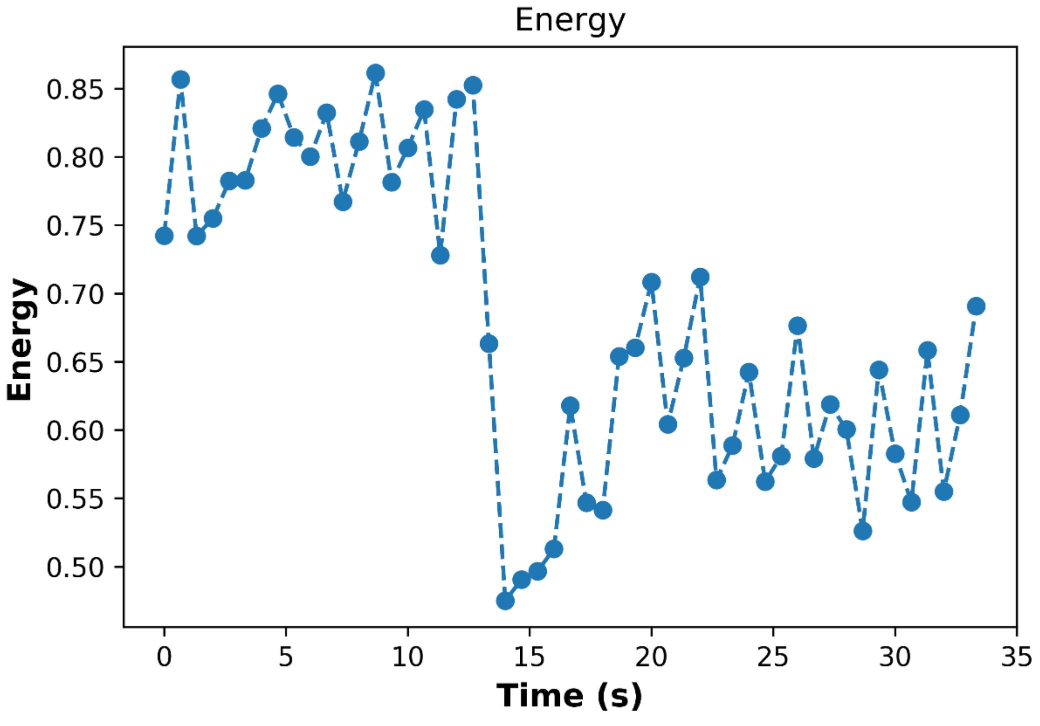

Energy
Energy
0.85
0.80
0.75
0.70
0.65
0.60
0.55
0.50
0
5
10
15
20
25
30
35
Time (s)

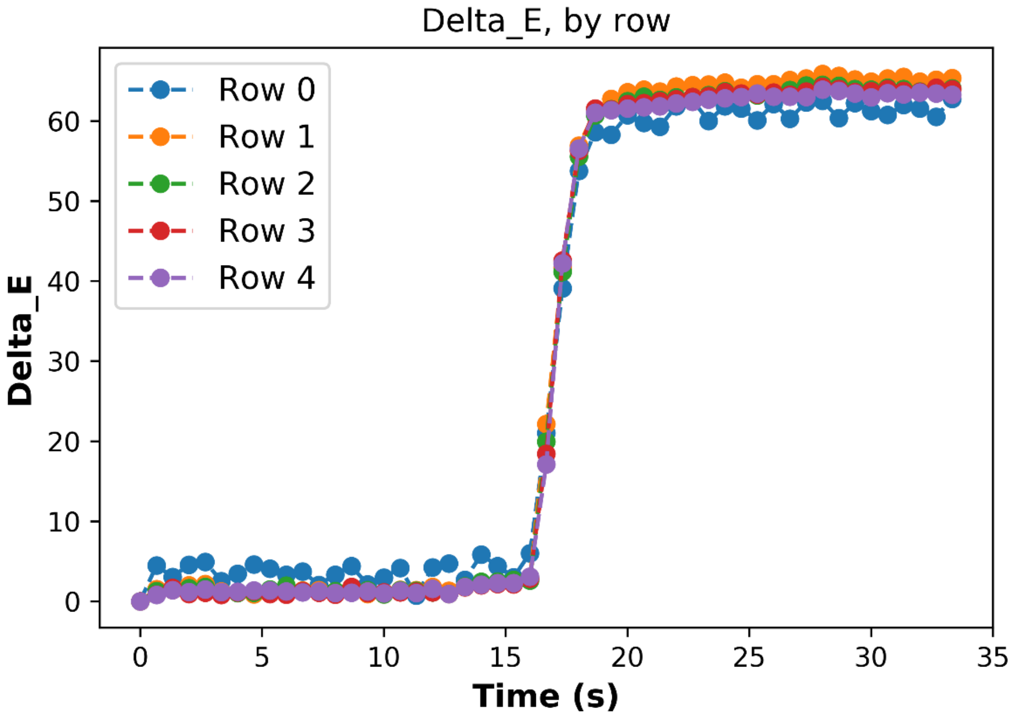
Delta_E, by row
Row 0
Row 1
Row 2
Row 3
Row 4
Delta_E
Time (s)

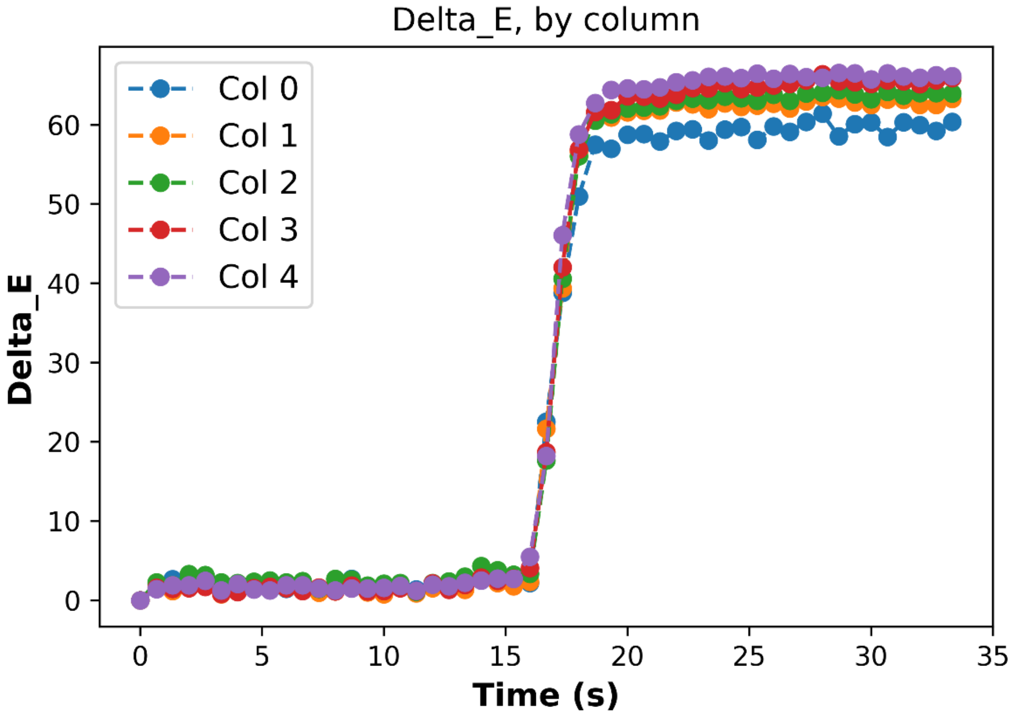
Delta_E, by column
Col 0
Col 1
Col 2
Col 3
Col 4
Delta_E
Time (s)

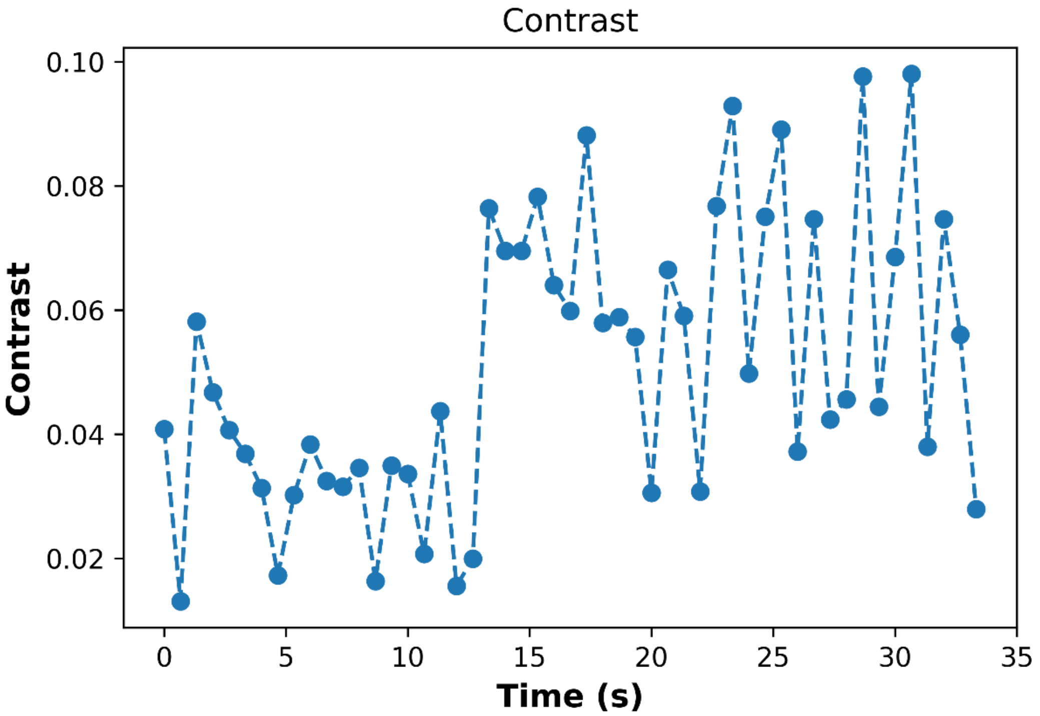
Contrast
0.10
0.08
0.06
0.04
0.02
Contrast
0
5
10
15
20
25
30
35
Time (s)

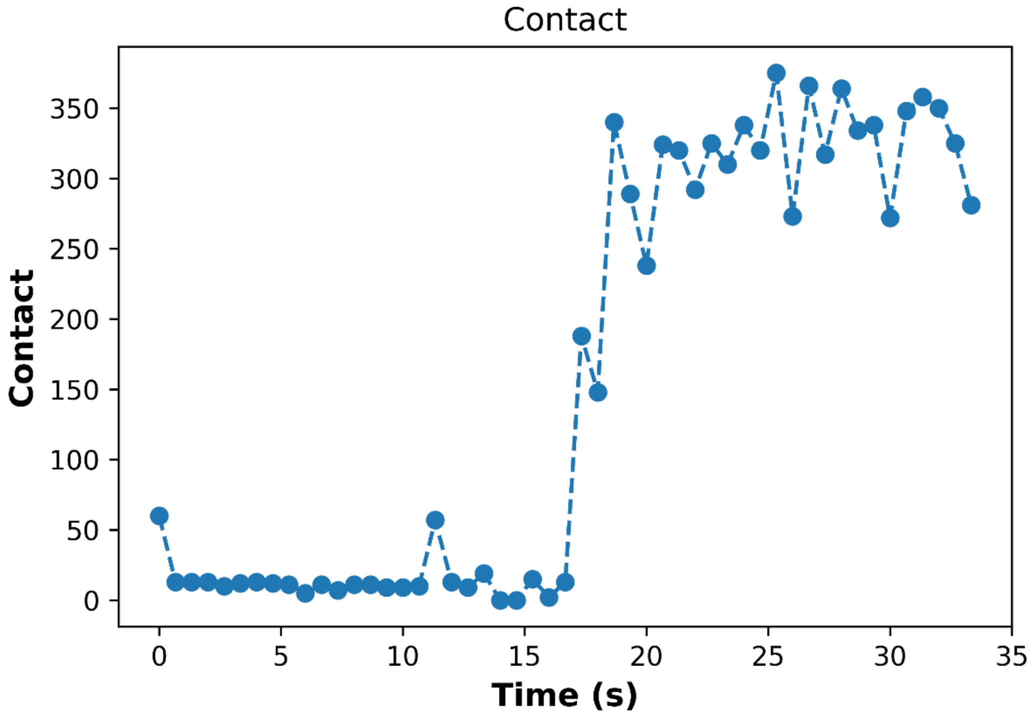
Contact
350
300
250
200
150
100
50
0
Contact
0
5
10
15
20
25
30
35
Time (s)

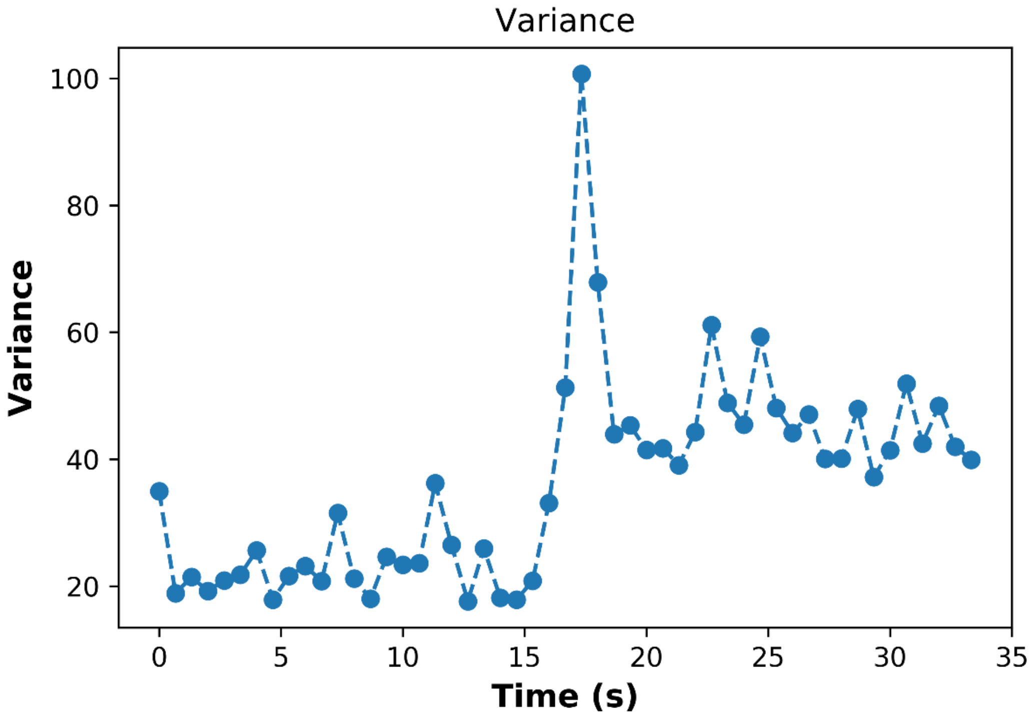
Variance
100
80
60
40
20
Variance
0
5
10
15
20
25
30
35
Time (s)

50 mL round bottom flask, micro-stirrer bar (5x2 mm)

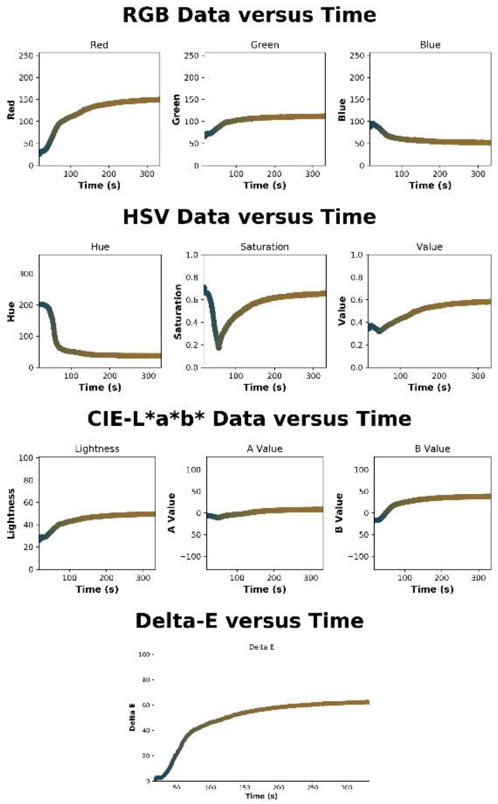

Kineticolor

Selected Region of Interest

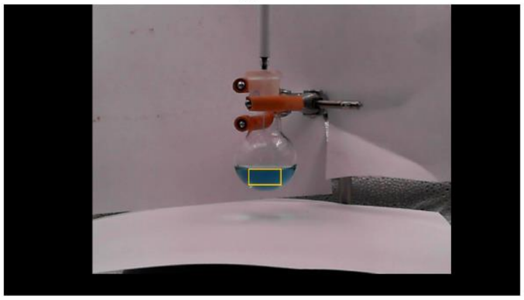

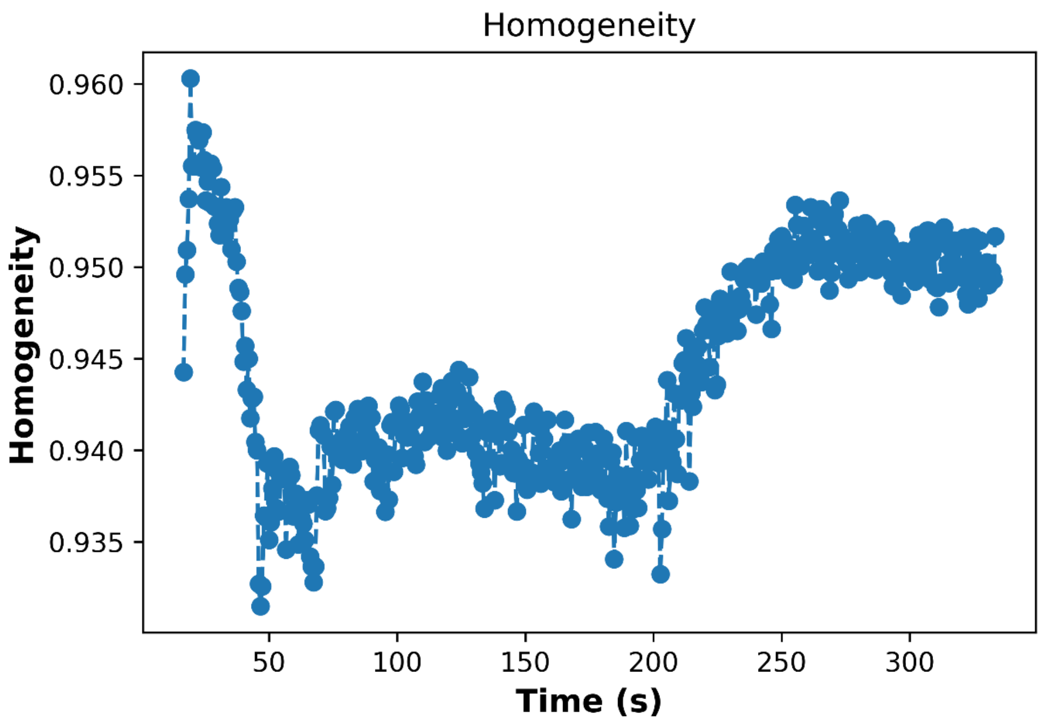
Homogeneity
0.960
0.955
0.950
0.945
0.940
0.935
Homogeneity
50
100
150
200
250
300
Time (s)

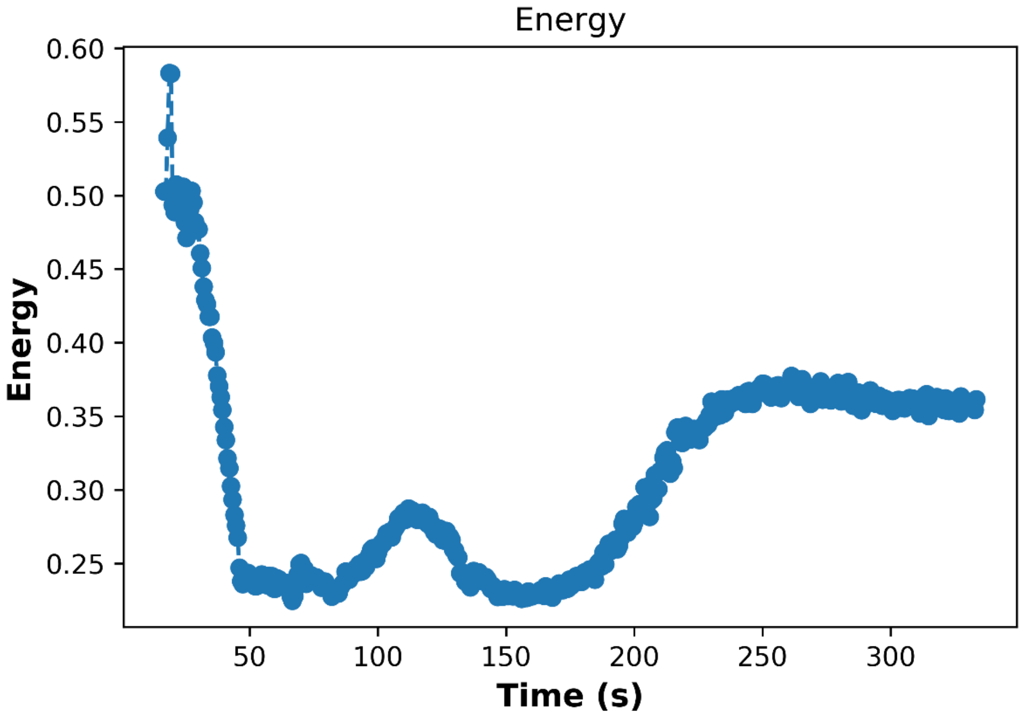
Energy
0.60
0.55
0.50
0.45
0.40
0.35
0.30
0.25
Energy
50
100
150
200
250
300
Time (s)

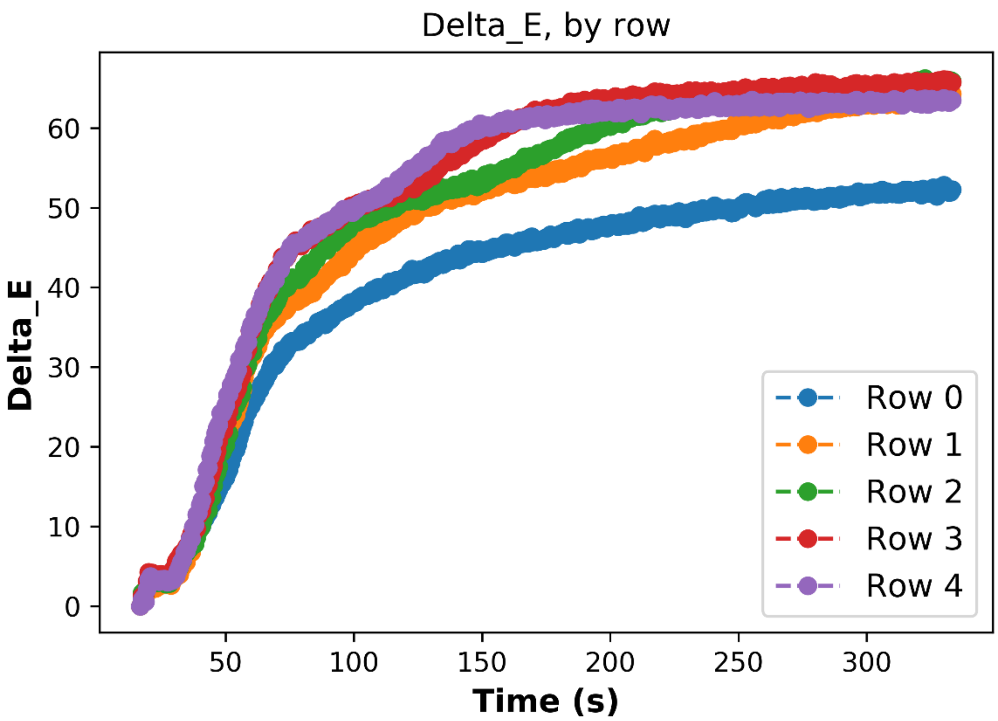
Delta_E, by row
Delta_E
Time (s)
0
10
20
30
40
50
60
50
100
150
200
250
300
Row 0
Row 1
Row 2
Row 3
Row 4

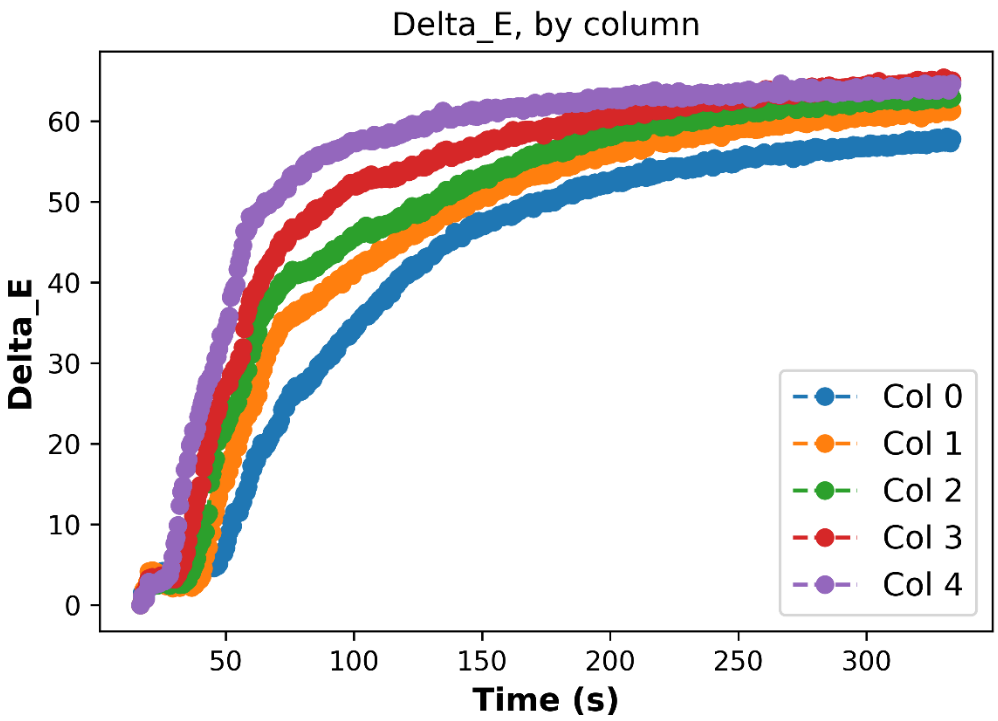
Delta_E, by column
Delta_E
Time (s)
0
10
20
30
40
50
60
50
100
150
200
250
300
Col 0
Col 1
Col 2
Col 3
Col 4

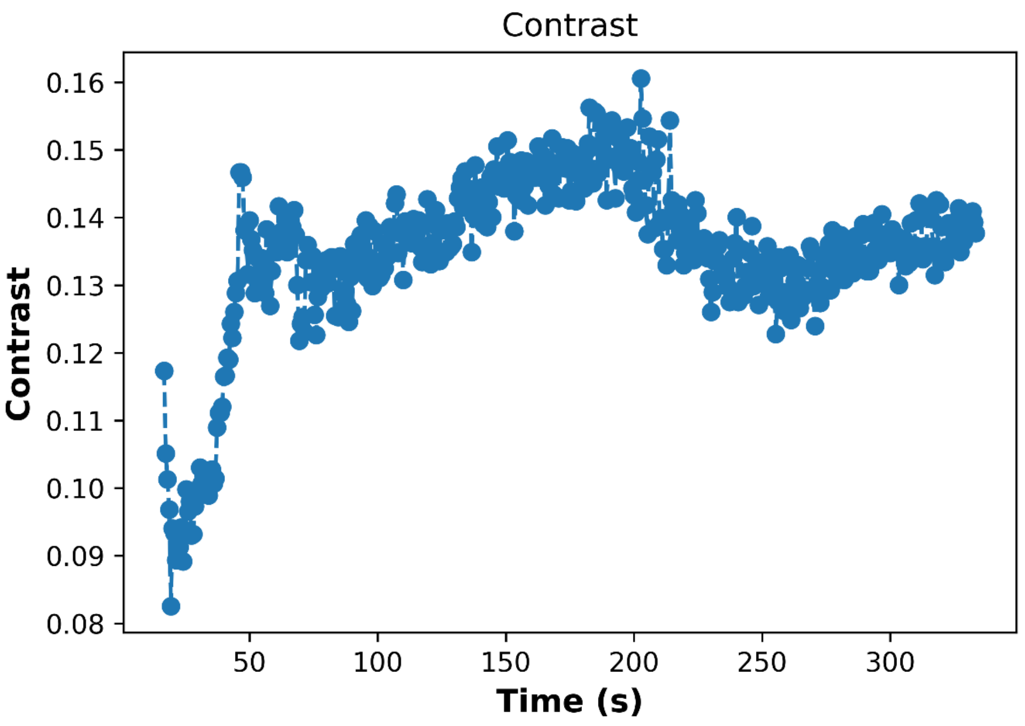
Contrast
0.16
0.15
0.14
0.13
0.12
0.11
0.10
0.09
0.08
Contrast
50
100
150
200
250
300
Time (s)

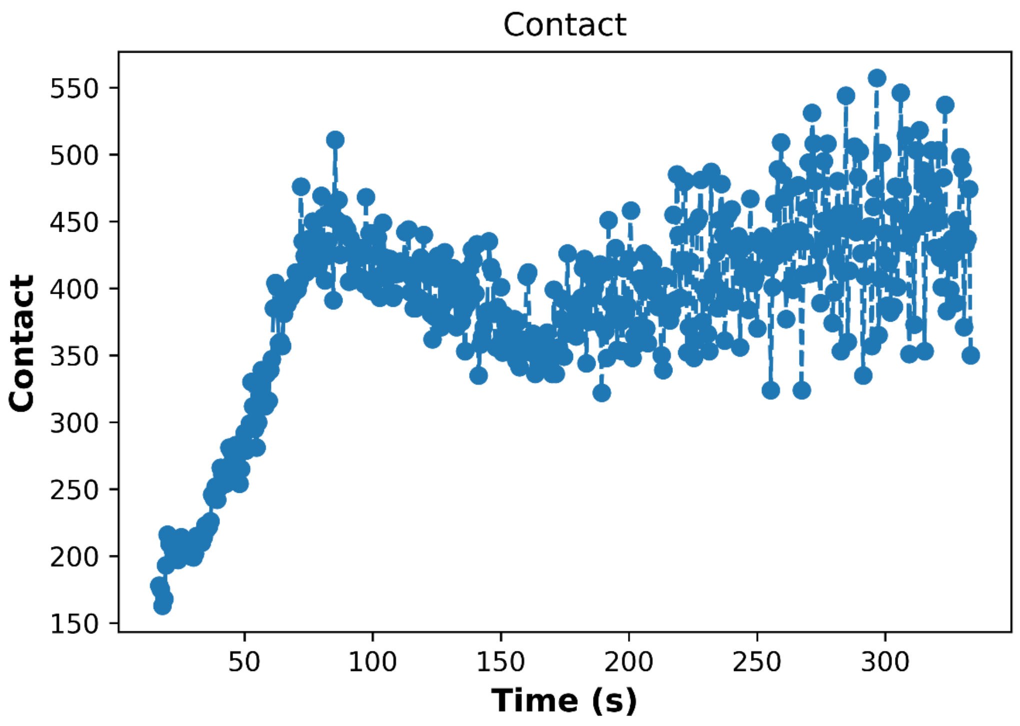
Contact
550
500
450
400
350
300
250
200
150
Contact
50
100
150
200
250
300
Time (s)

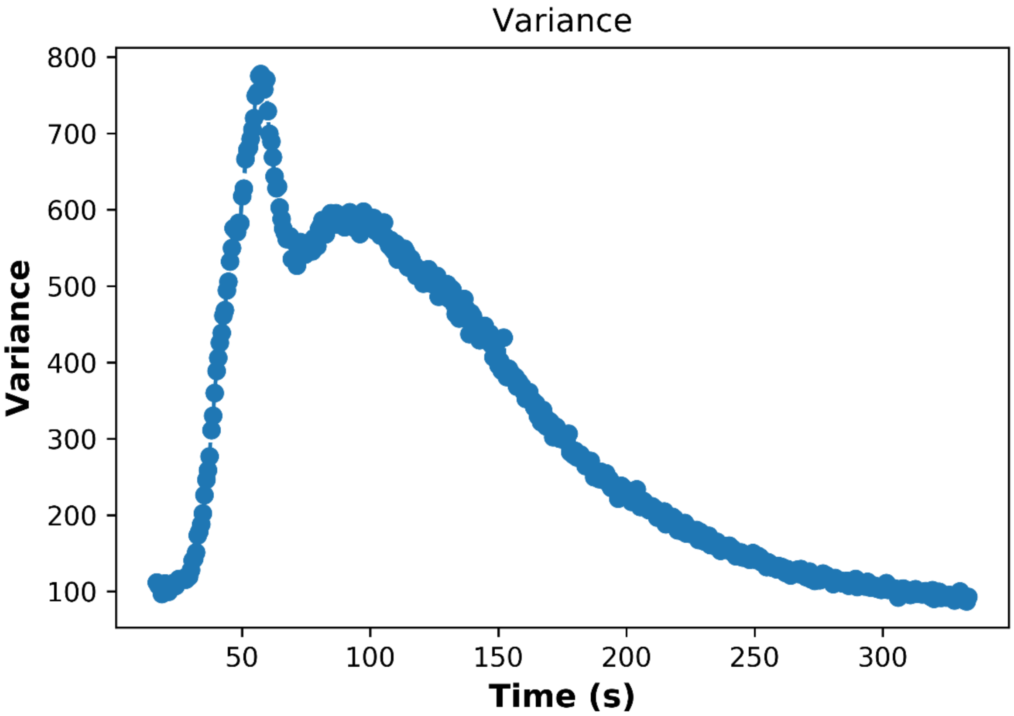

Variance
800
700
600
500
400
300
200
100
Variance
50
100
150
200
250
300
Time (s)

3 L beaker, no baffles or stirring

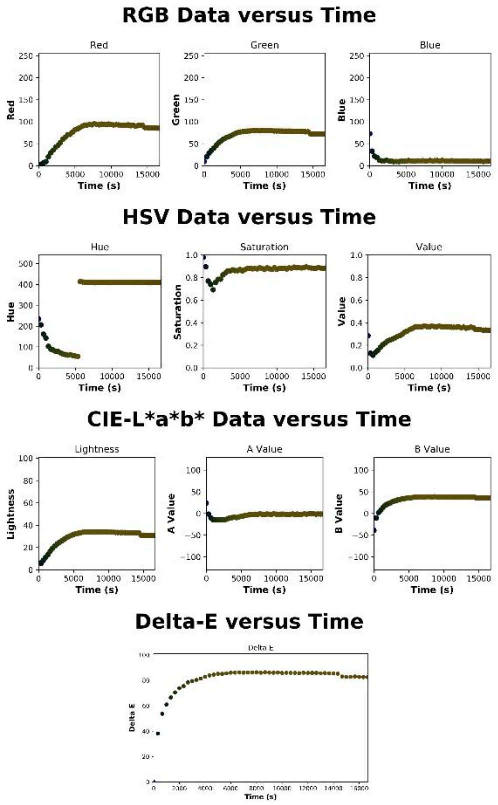

Kineticolor

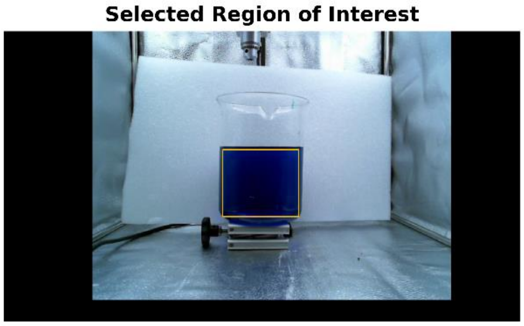

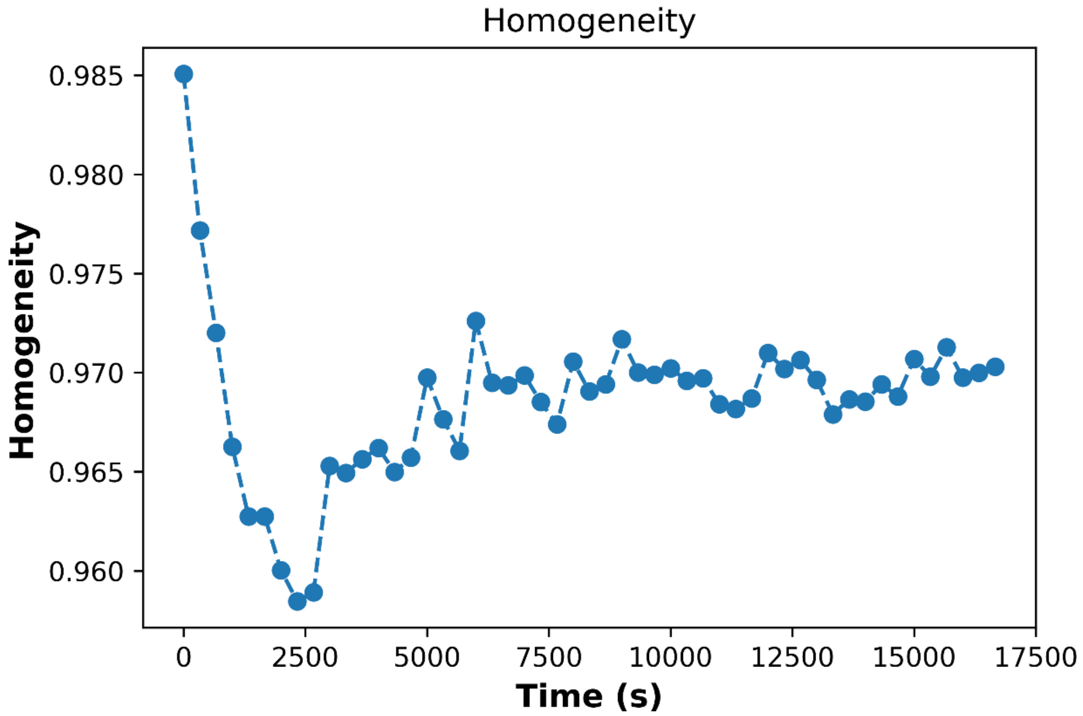
Homogeneity
0.985
0.980
0.975
0.970
0.965
0.960
Homogeneity
0
2500
5000
7500
10000
12500
15000
17500
Time (s)

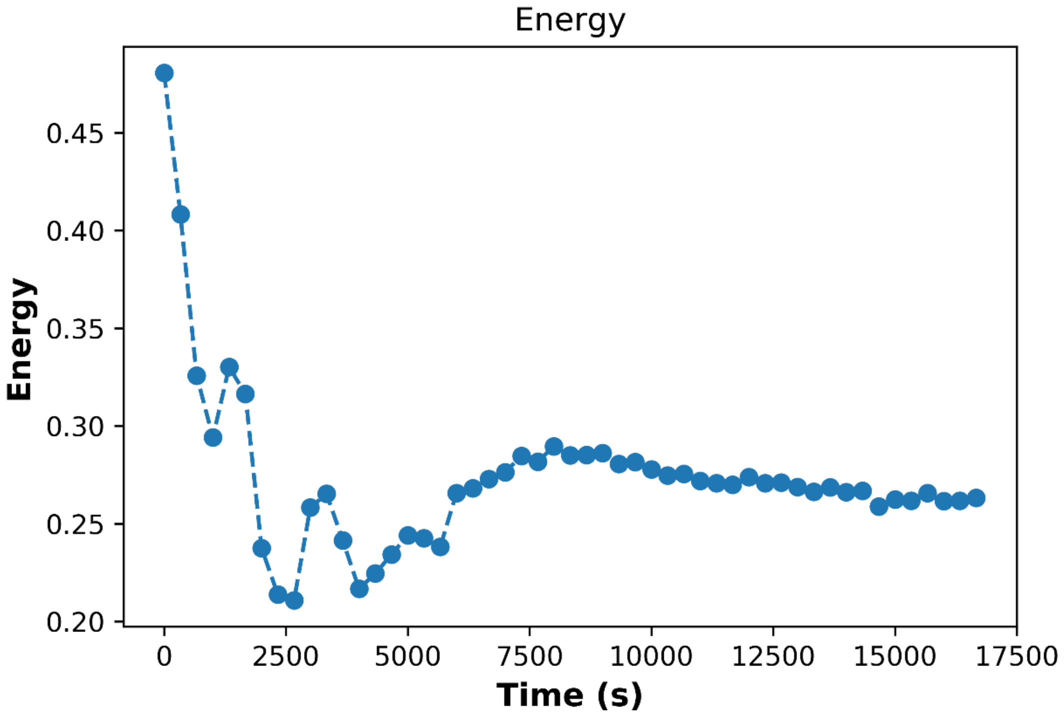
Energy
0.45
0.40
0.35
0.30
0.25
0.20
Energy
0
2500
5000
7500
10000
12500
15000
17500
Time (s)

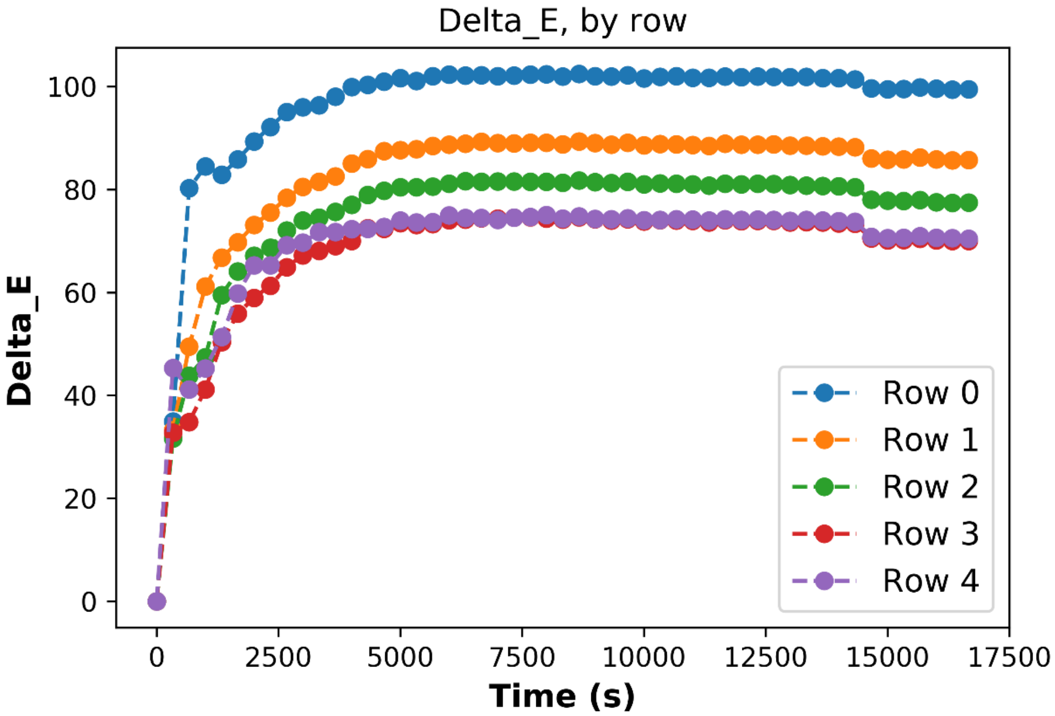
Delta_E, by row
Delta_E
Time (s)
Row 0
Row 1
Row 2
Row 3
Row 4
0
20
40
60
80
100
0
2500
5000
7500
10000
12500
15000
17500

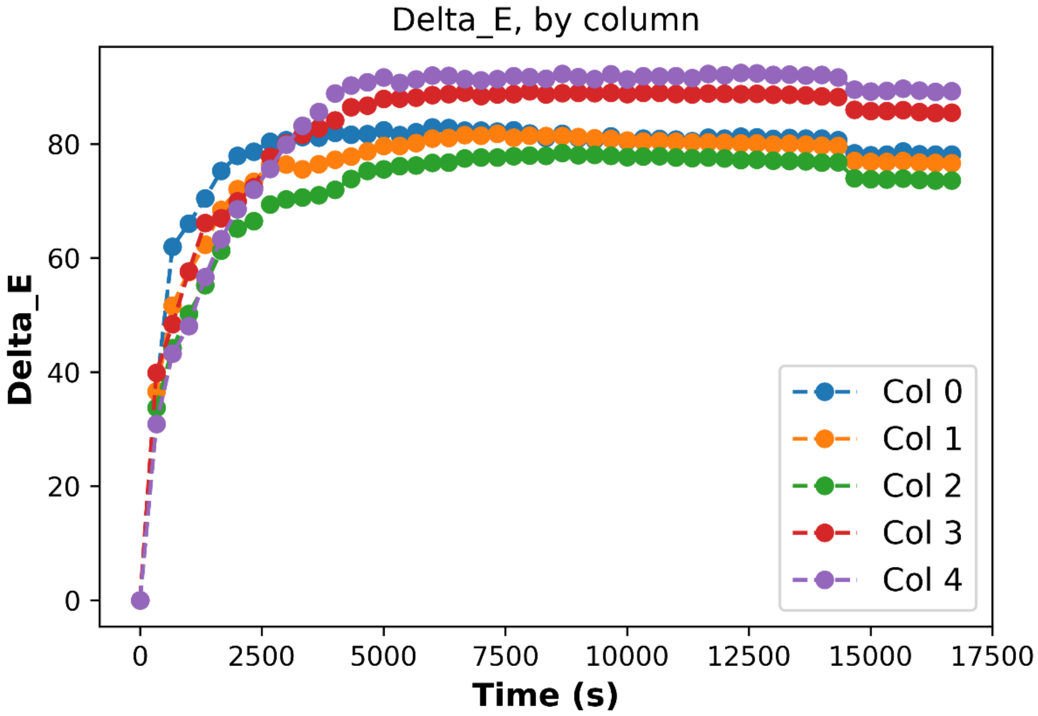
Delta_E, by column
Delta_E
Time (s)
Col 0
Col 1
Col 2
Col 3
Col 4
0
20
40
60
80
0
2500
5000
7500
10000
12500
15000
17500

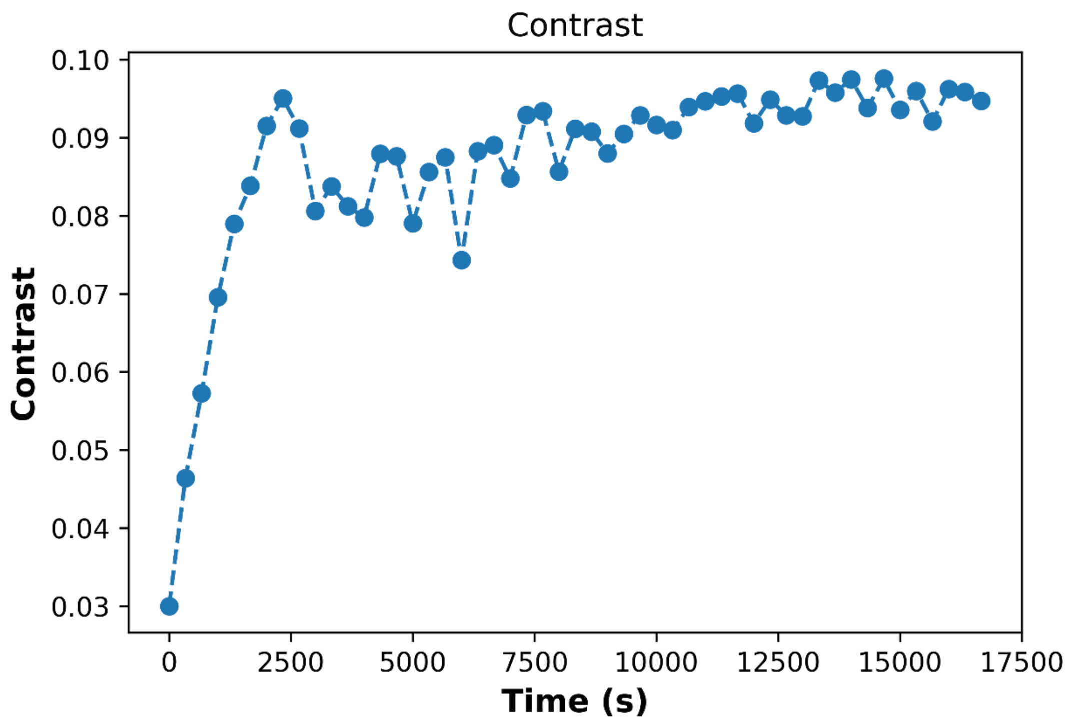

Contrast
Contrast
0.10
0.09
0.08
0.07
0.06
0.05
0.04
0.03
0
2500
5000
7500
10000
12500
15000
17500
Time (s)

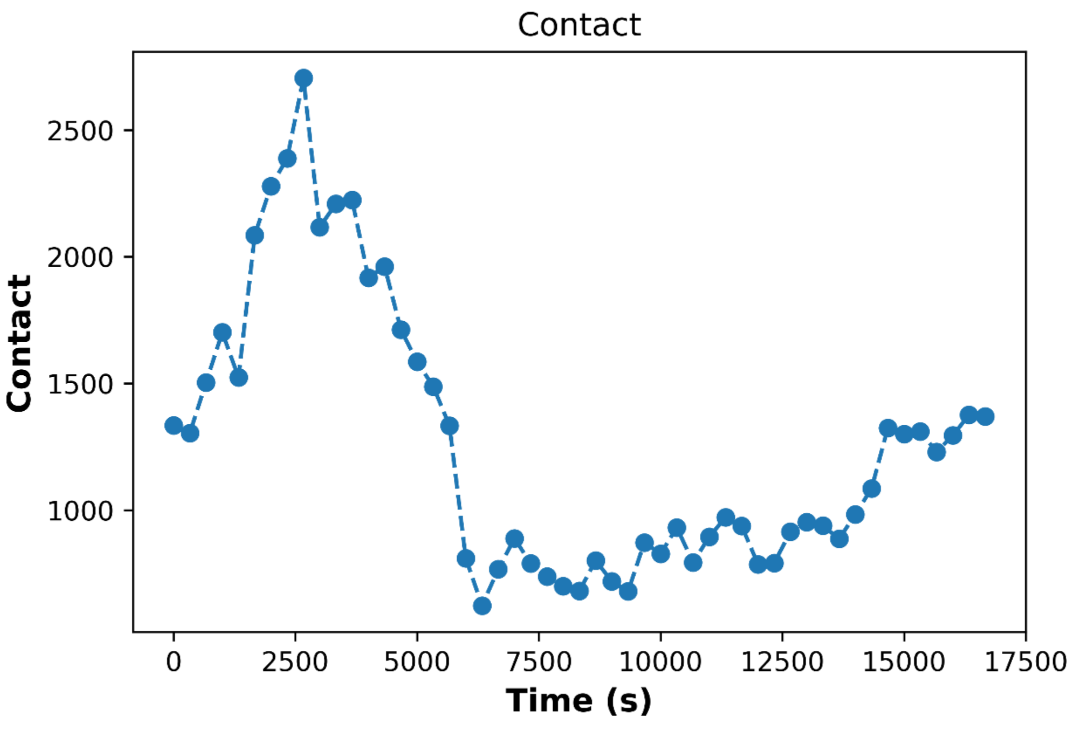

Contact
Contact
2500
2000
1500
1000
0
2500
5000
7500
10000
12500
15000
17500
Time (s)

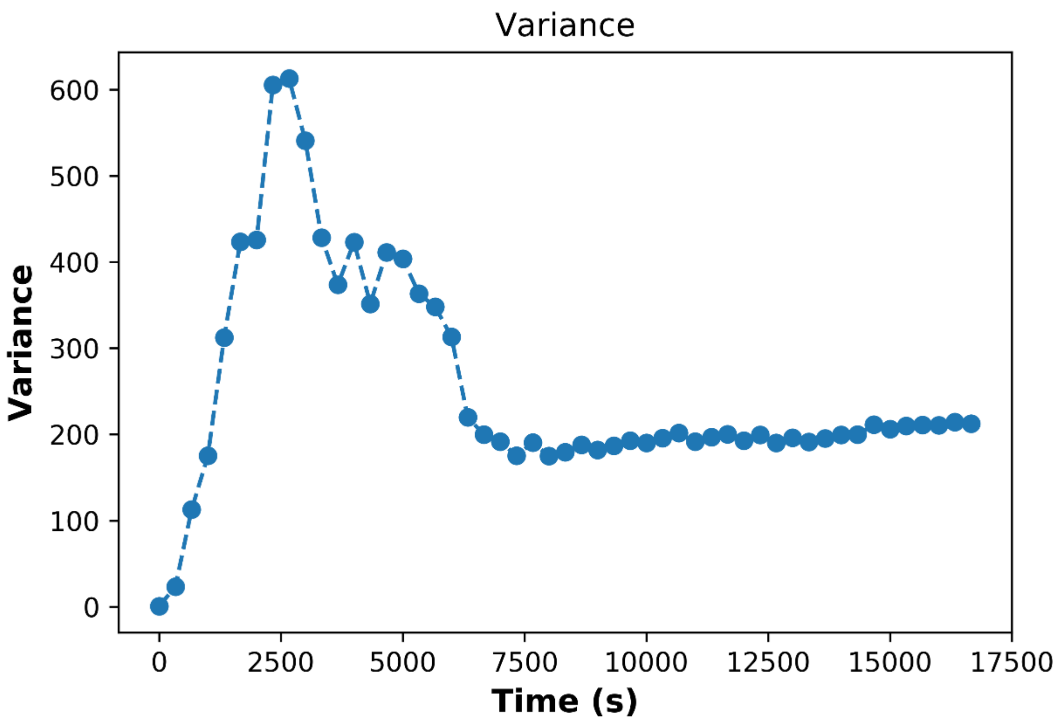

Variance
600
500
400
300
200
100
0
Variance
0
2500
5000
7500
10000
12500
15000
17500
Time (s)

5 L STR, paddle, no probe

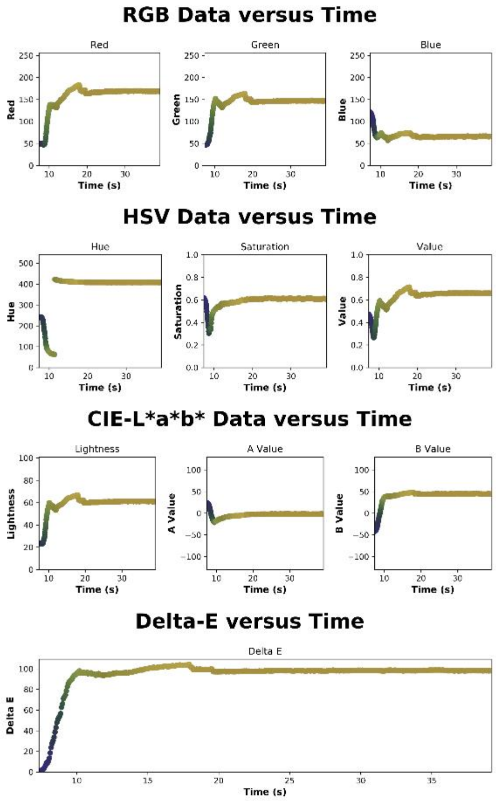

Kineticolor

Selected Region of Interest

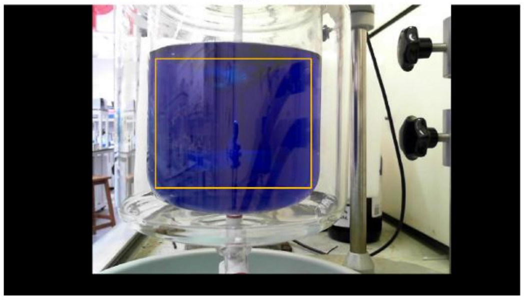

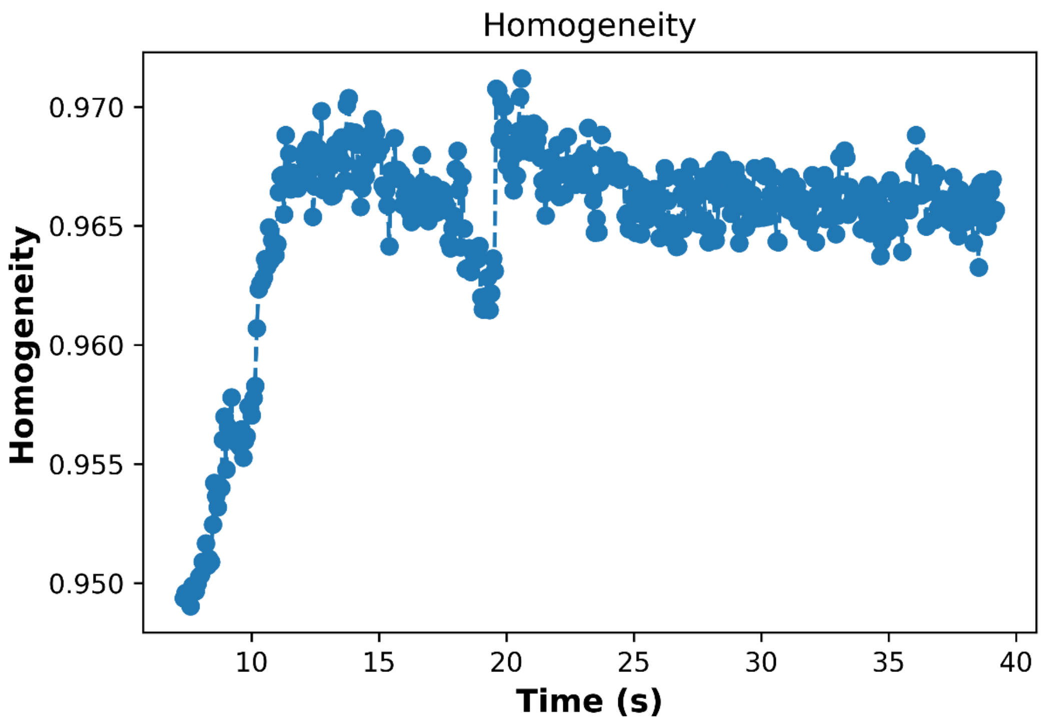
Homogeneity
Homogeneity
0.970
0.965
0.960
0.955
0.950
10
15
20
25
30
35
40
Time (s)

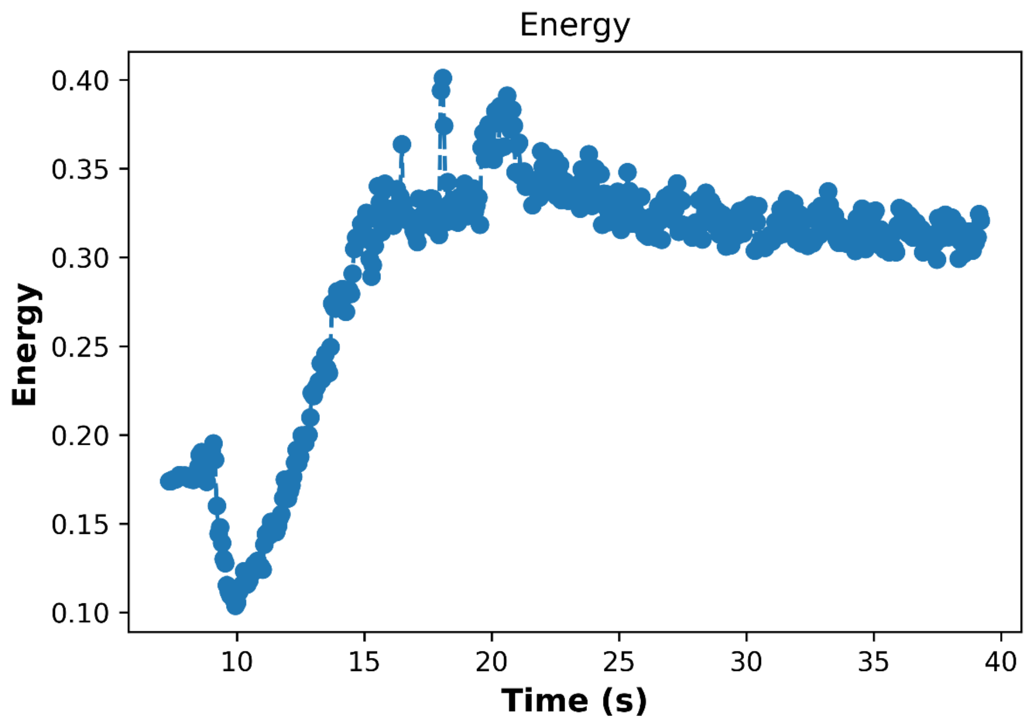
Energy
Energy
0.40
0.35
0.30
0.25
0.20
0.15
0.10
10
15
20
25
30
35
40
Time (s)

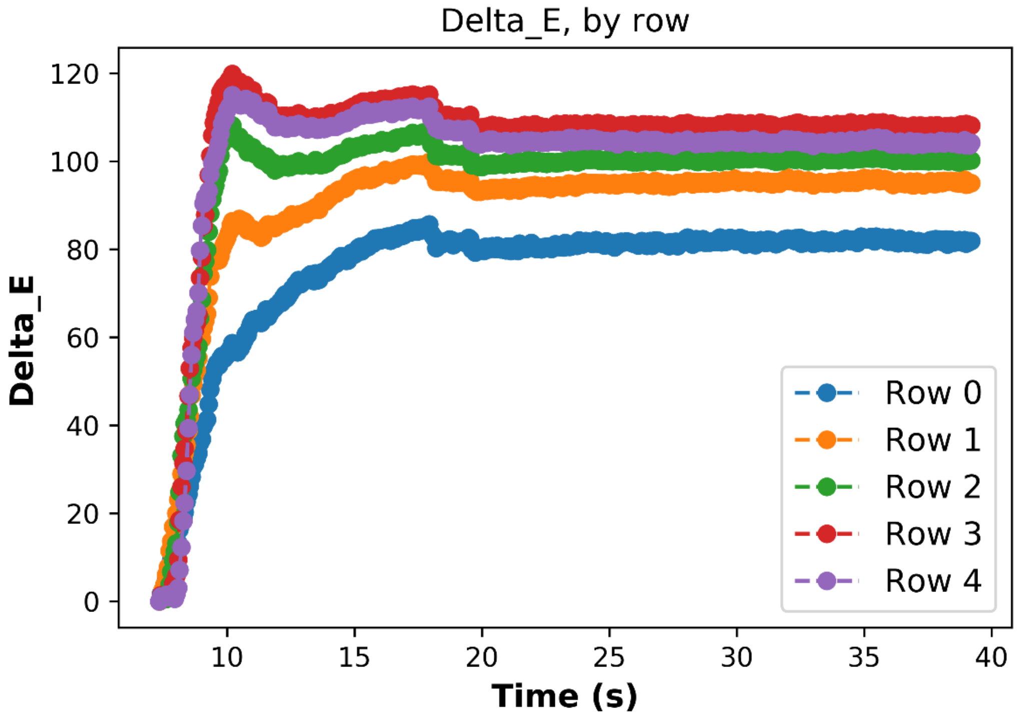
Delta_E, by row
Delta_E
Time (s)
0
20
40
60
80
100
120
10
15
20
25
30
35
40
Row 0
Row 1
Row 2
Row 3
Row 4

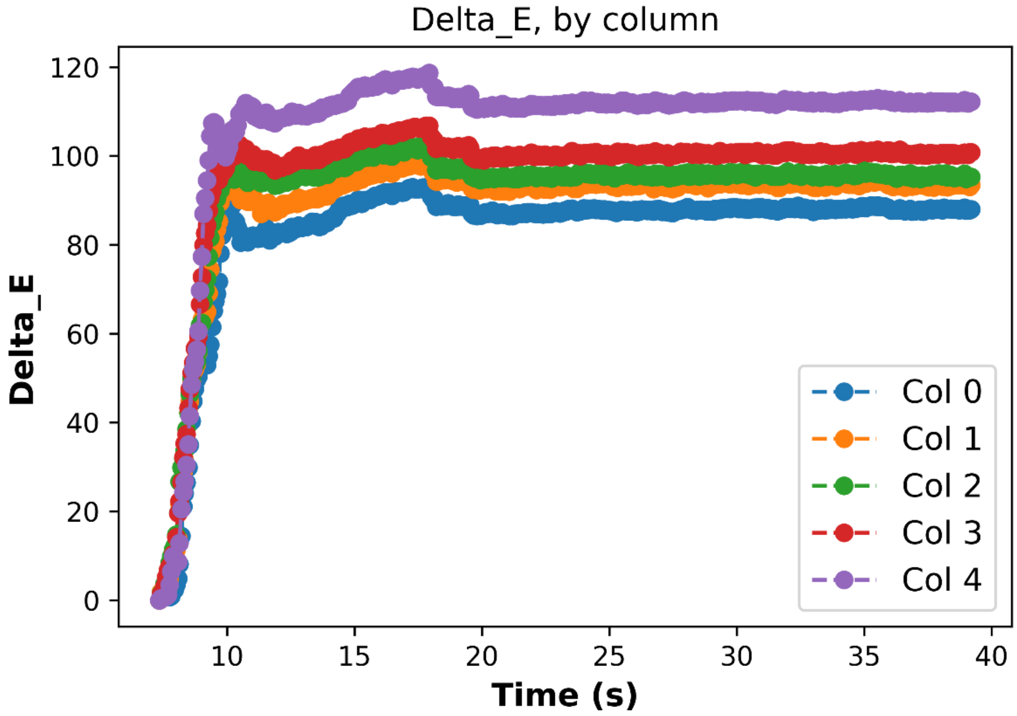
Delta_E, by column
Delta_E
Time (s)
0
20
40
60
80
100
120
10
15
20
25
30
35
40
Col 0
Col 1
Col 2
Col 3
Col 4

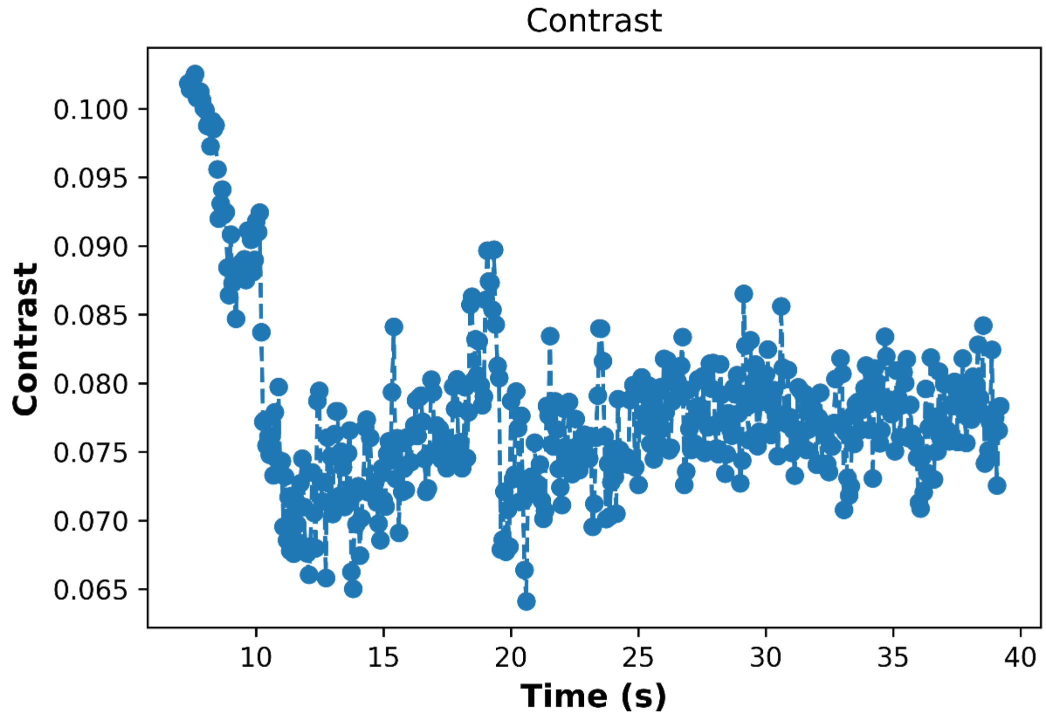
Contrast
Contrast
0.100
0.095
0.090
0.085
0.080
0.075
0.070
0.065
10
15
20
25
30
35
40
Time (s)

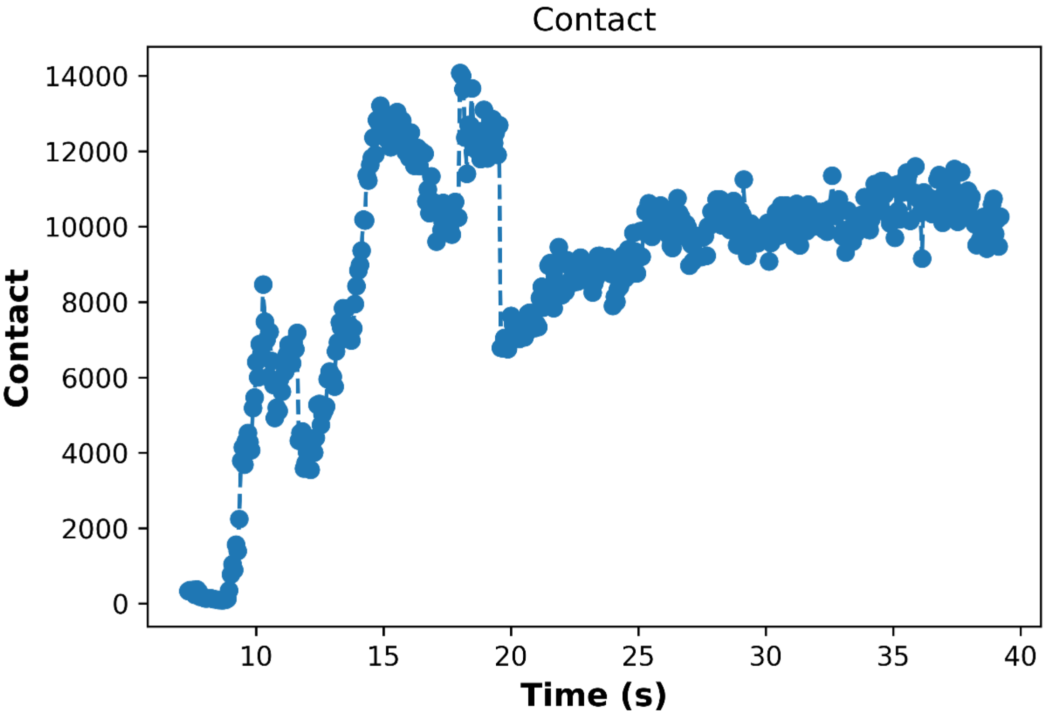
Contact
Contact
14000
12000
10000
8000
6000
4000
2000
0
10
15
20
25
30
35
40
Time (s)

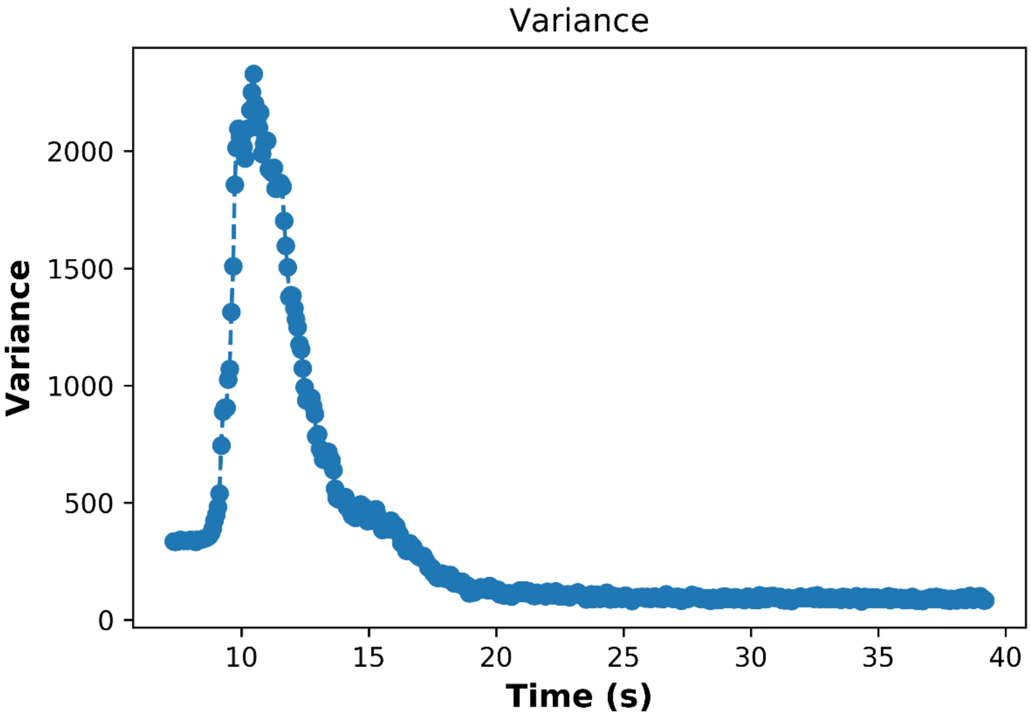

Variance
2000
1500
1000
500
0
Variance
10
15
20
25
30
35
40
Time (s)

5 L STR, paddle, with probe

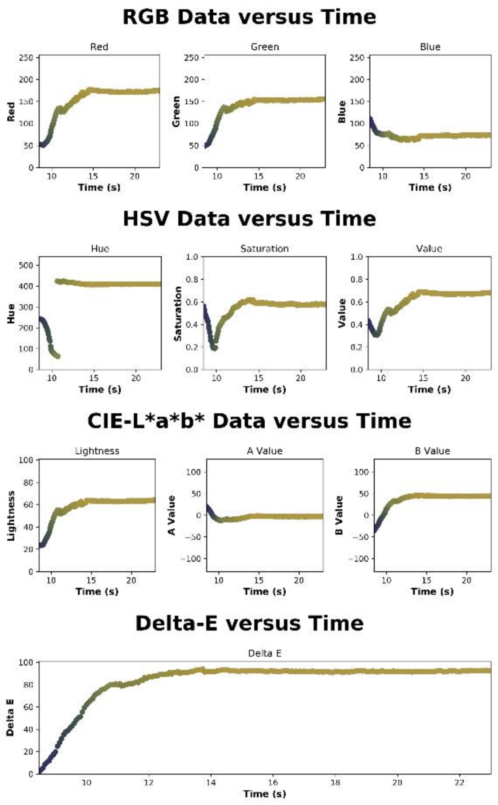

Kineticolor

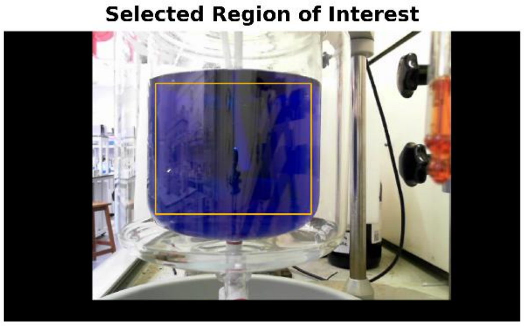

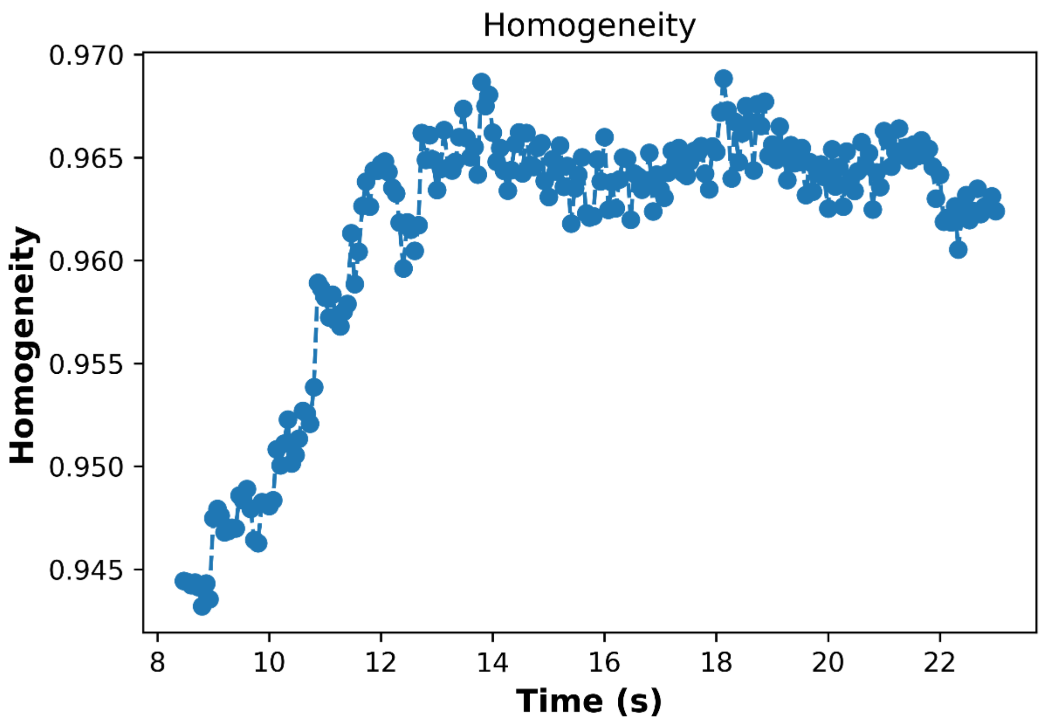
Homogeneity
Homogeneity
0.970
0.965
0.960
0.955
0.950
0.945
8
10
12
14
16
18
20
22
Time (s)

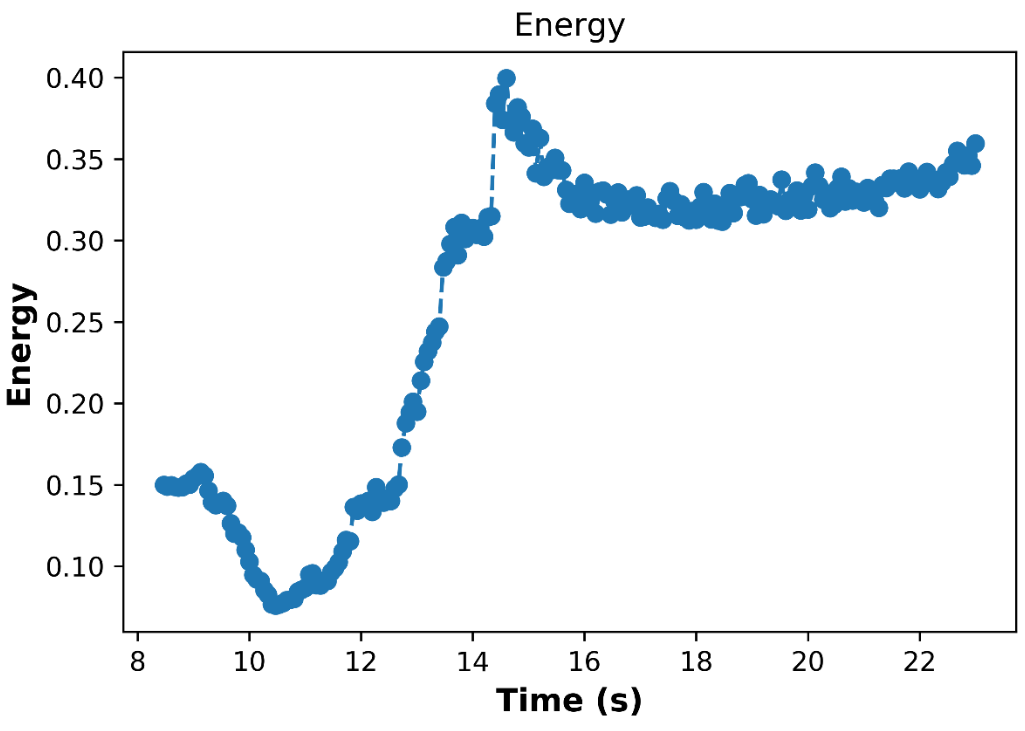
Energy
Energy
0.40
0.35
0.30
0.25
0.20
0.15
0.10
8
10
12
14
16
18
20
22
Time (s)

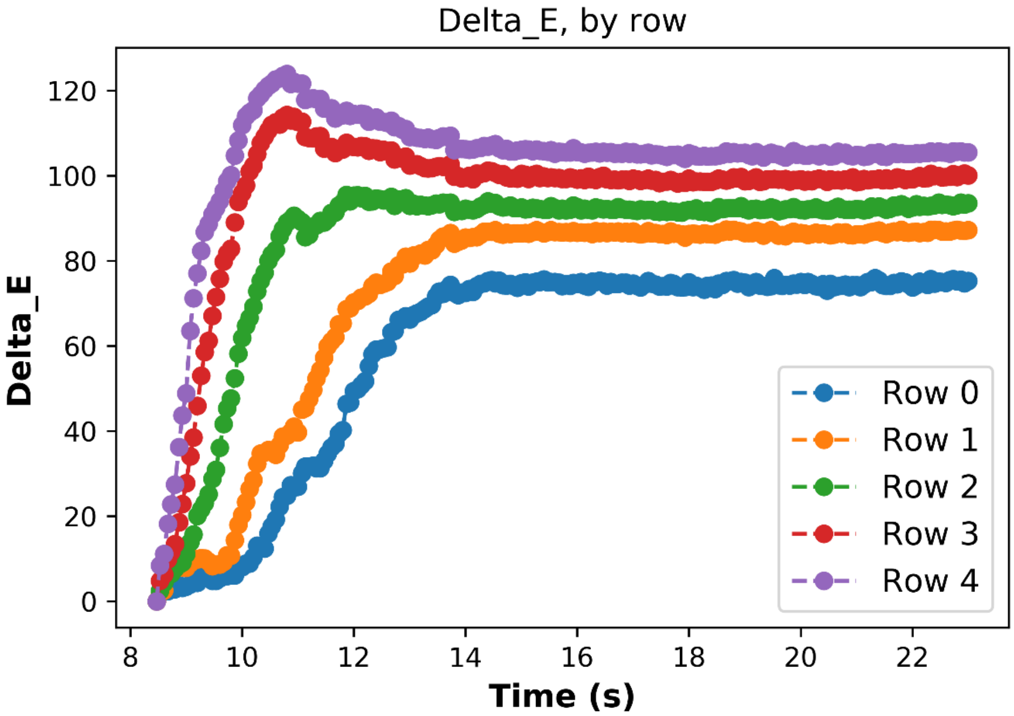
Delta_E, by row
Delta_E
Time (s)
Row 0
Row 1
Row 2
Row 3
Row 4
0
20
40
60
80
100
120
8
10
12
14
16
18
20
22

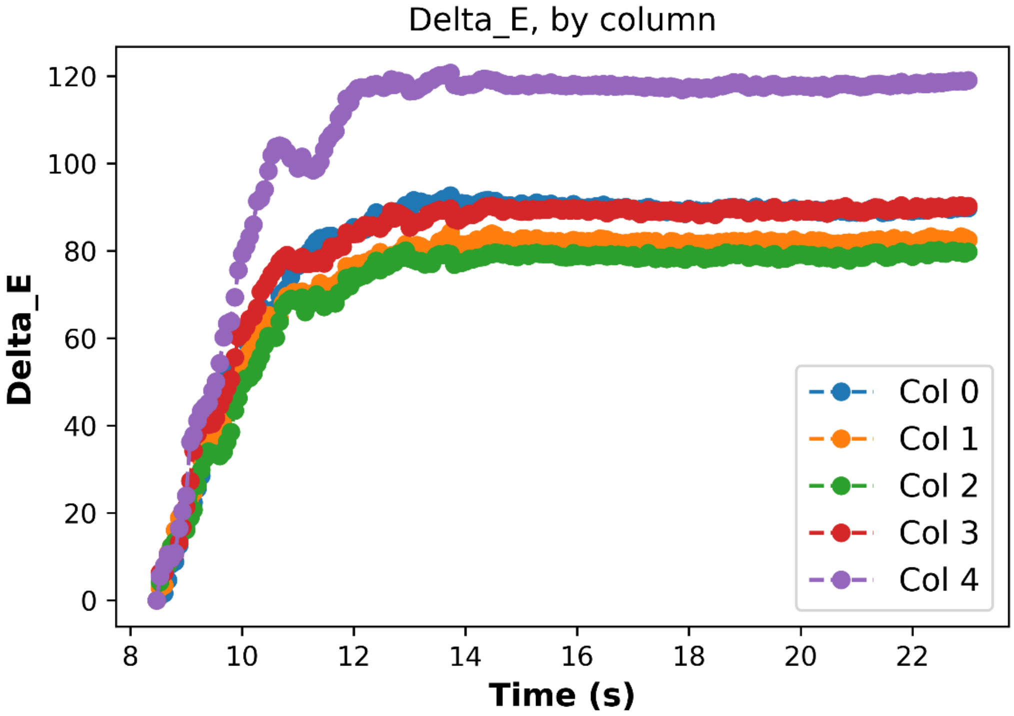
Delta_E, by column
Delta_E
Time (s)
Col 0
Col 1
Col 2
Col 3
Col 4
0
20
40
60
80
100
120
8
10
12
14
16
18
20
22

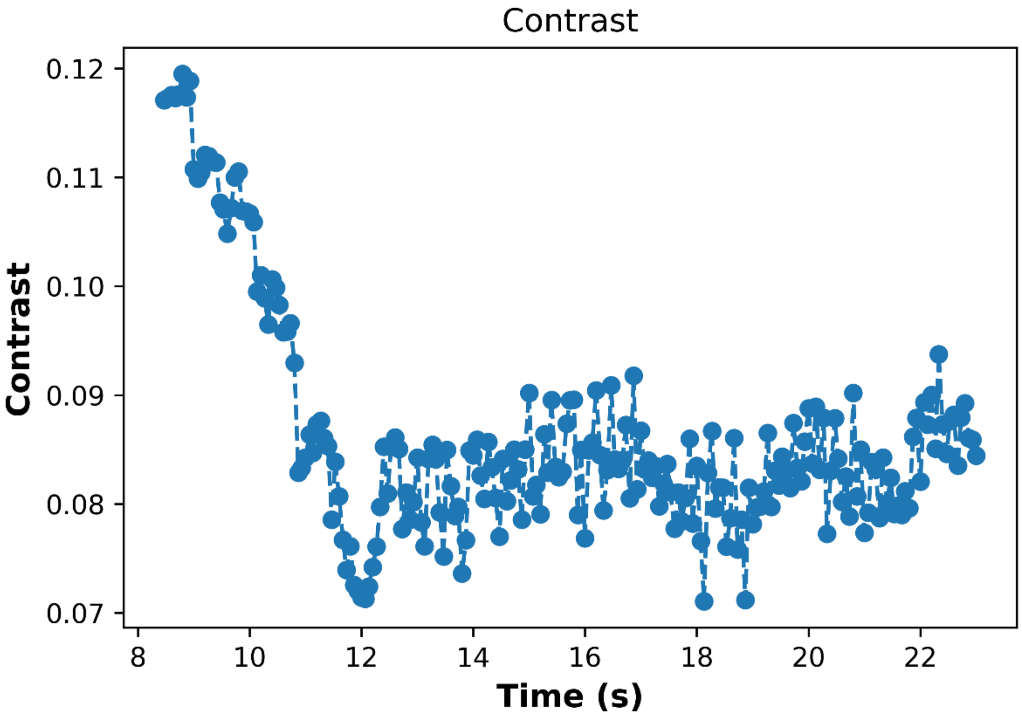
Contrast
Contrast
0.12
0.11
0.10
0.09
0.08
0.07
8
10
12
14
16
18
20
22
Time (s)

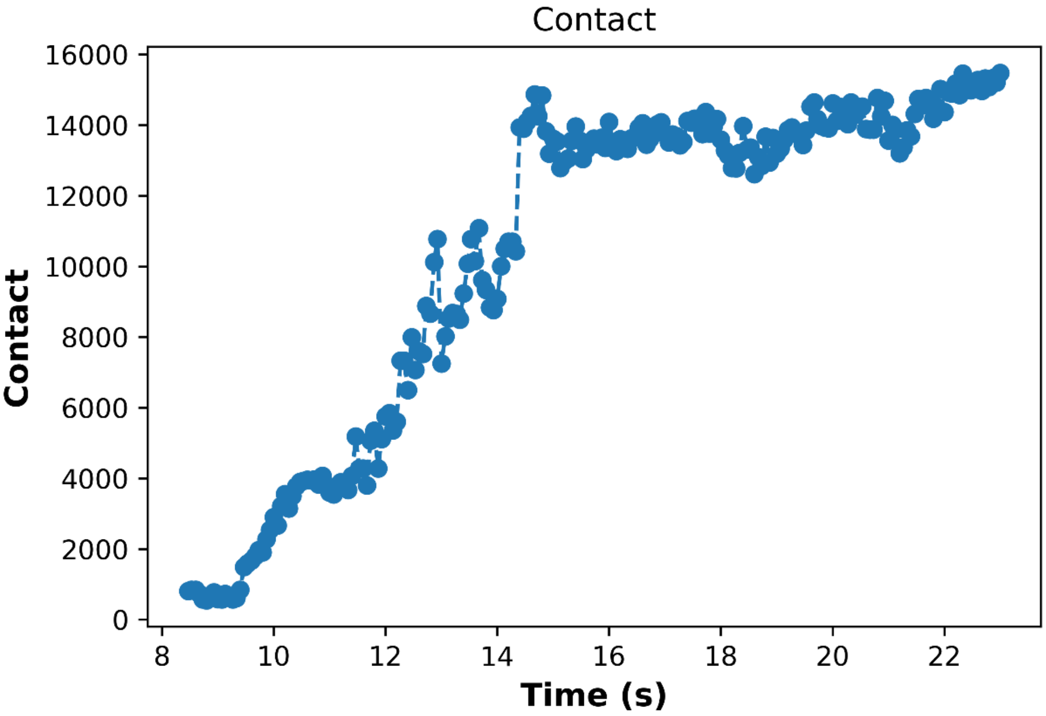
Contact
Contact
16000
14000
12000
10000
8000
6000
4000
2000
0
8
10
12
14
16
18
20
22
Time (s)

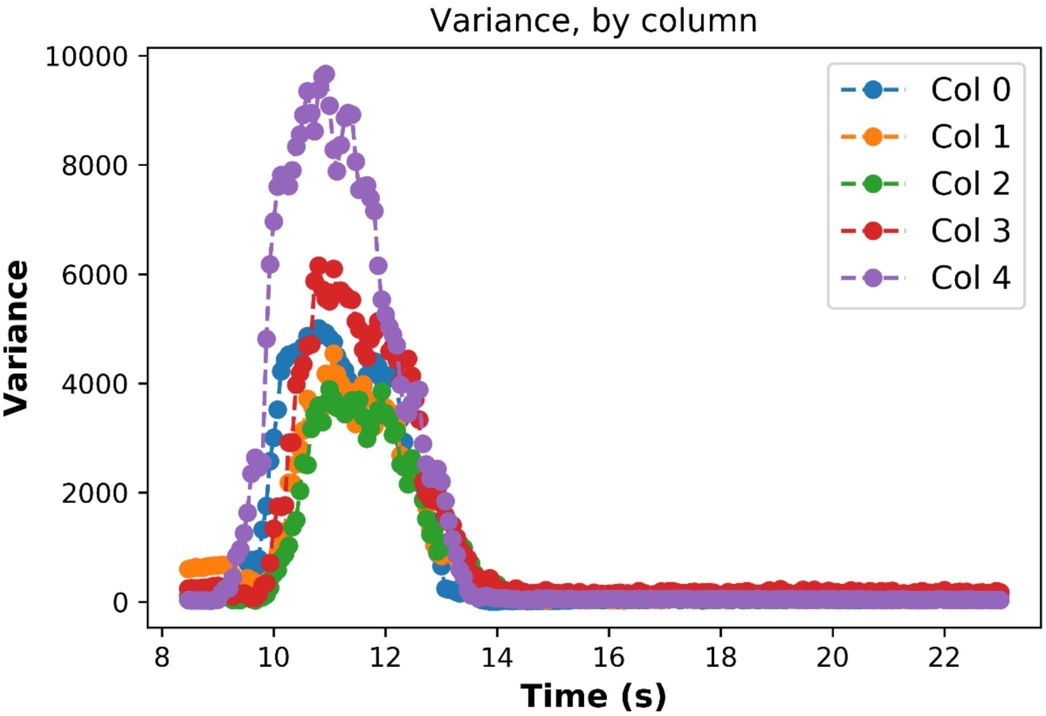

Variance, by column
10000
8000
6000
4000
2000
0
Variance
8
10
12
14
16
18
20
22
Time (s)
Col 0
Col 1
Col 2
Col 3
Col 4

5 L STR, anchor, no probe

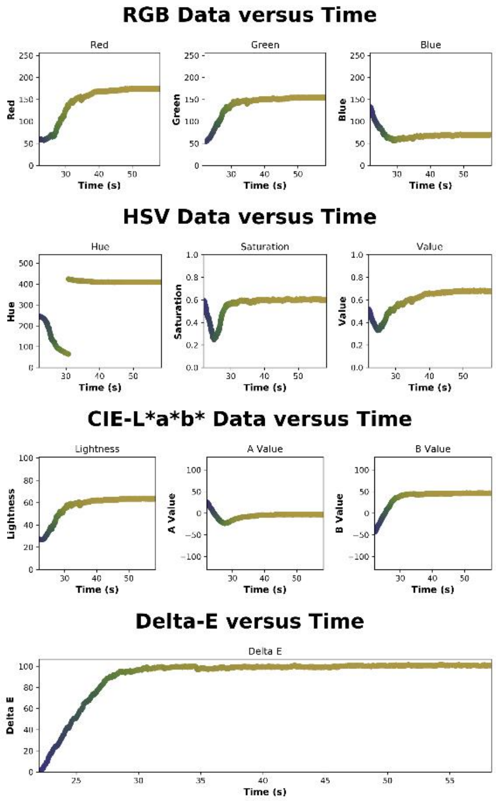

Kineticolor

Selected Region of Interest

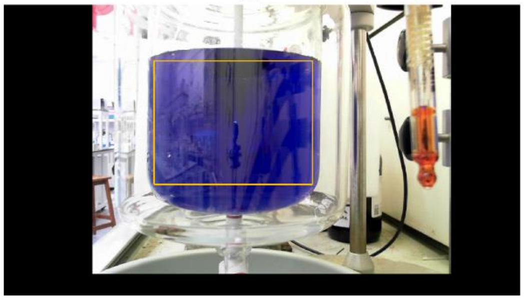

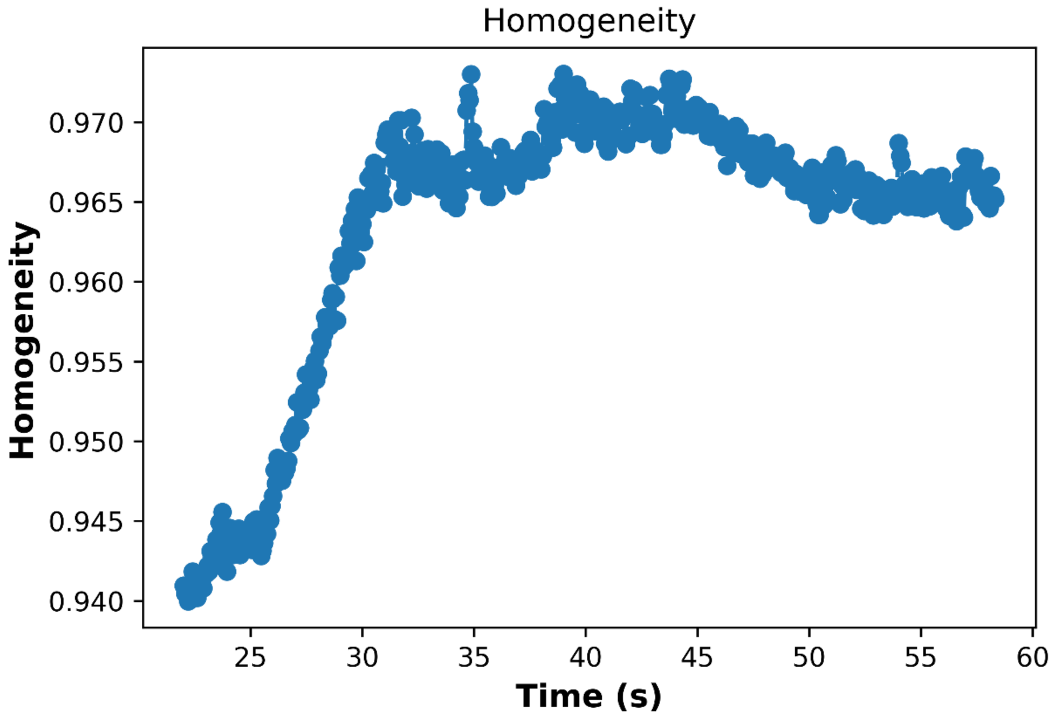
Homogeneity
Homogeneity
0.940
0.945
0.950
0.955
0.960
0.965
0.970
25
30
35
40
45
50
55
60
Time (s)

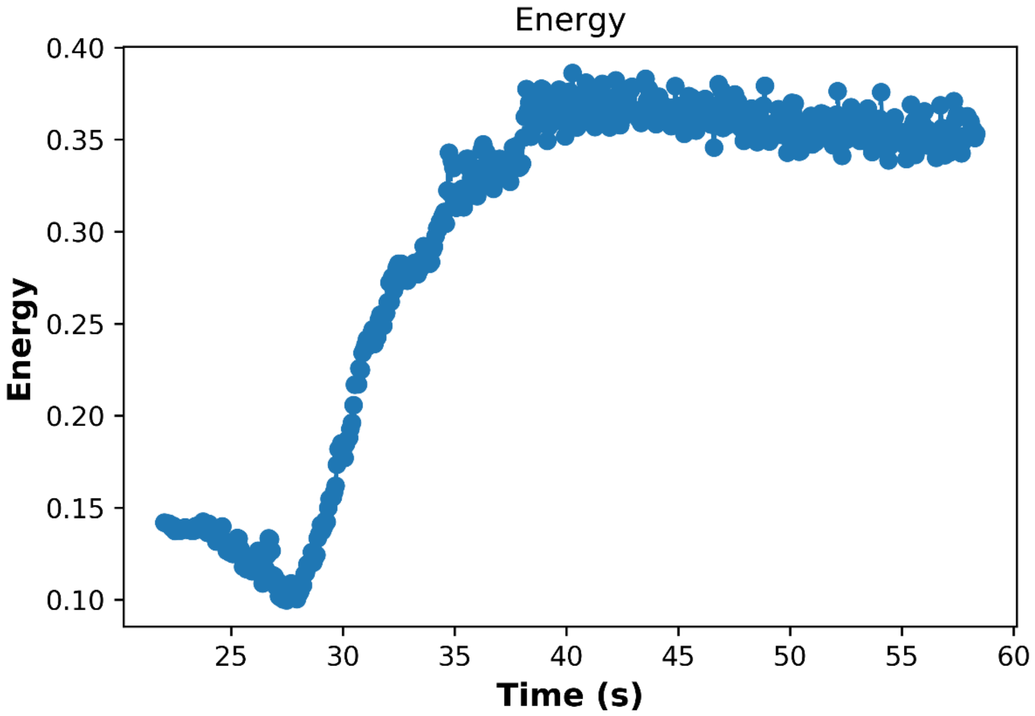
Energy
Energy
0.10
0.15
0.20
0.25
0.30
0.35
0.40
25
30
35
40
45
50
55
60
Time (s)

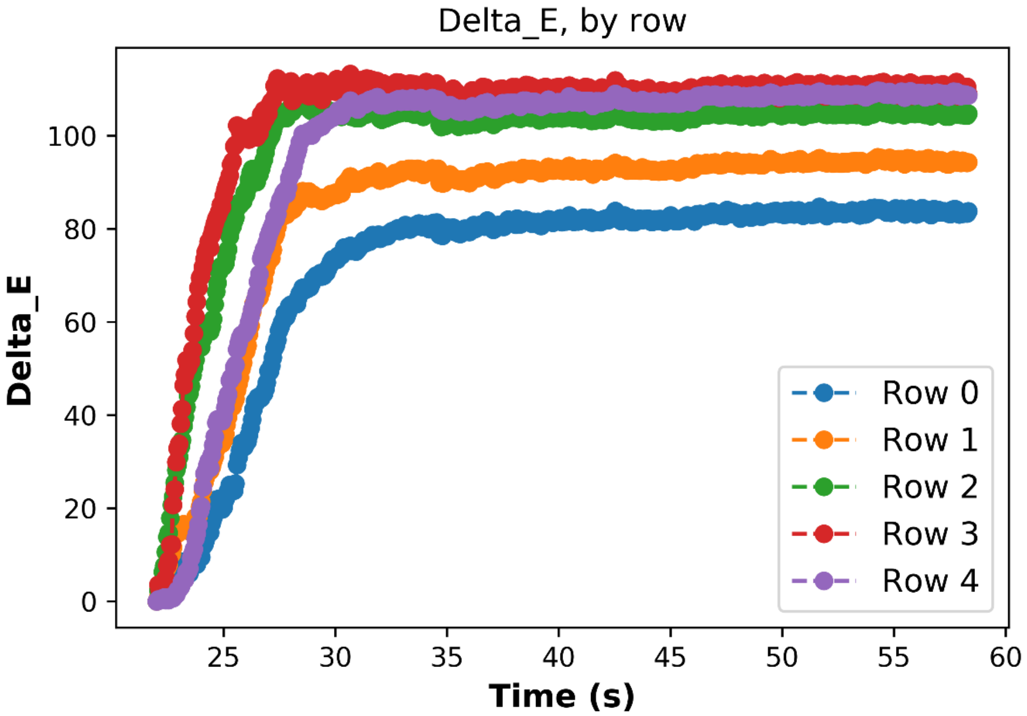

Delta_E, by row
Delta_E
Time (s)
Row 0
Row 1
Row 2
Row 3
Row 4
0
20
40
60
80
100
25
30
35
40
45
50
55
60

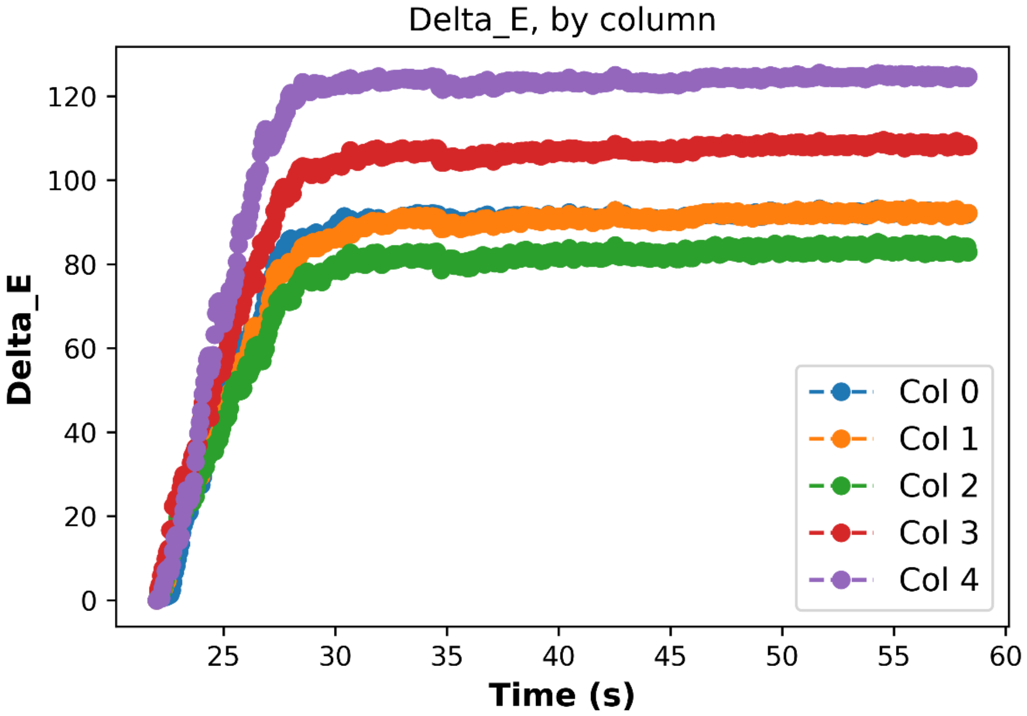

Delta_E, by column
Delta_E
Time (s)
Col 0
Col 1
Col 2
Col 3
Col 4
0
20
40
60
80
100
120
25
30
35
40
45
50
55
60

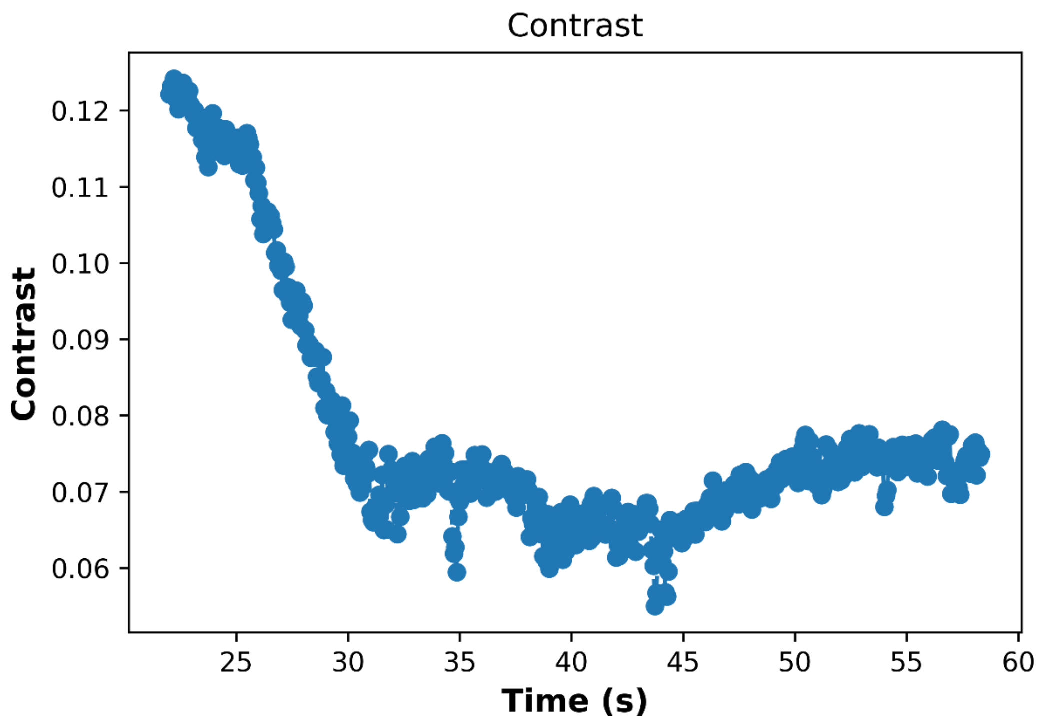
Contrast
0.12
0.11
0.10
0.09
0.08
0.07
0.06
Contrast
25
30
35
40
45
50
55
60
Time (s)

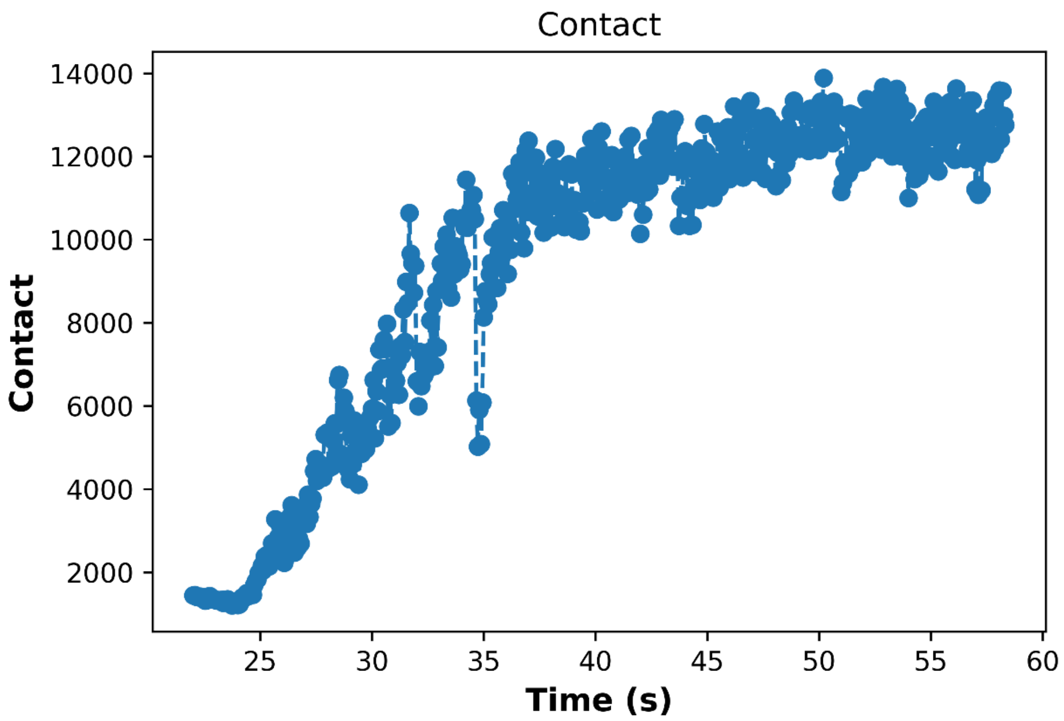
Contact
14000
12000
10000
8000
6000
4000
2000
Contact
25
30
35
40
45
50
55
60
Time (s)

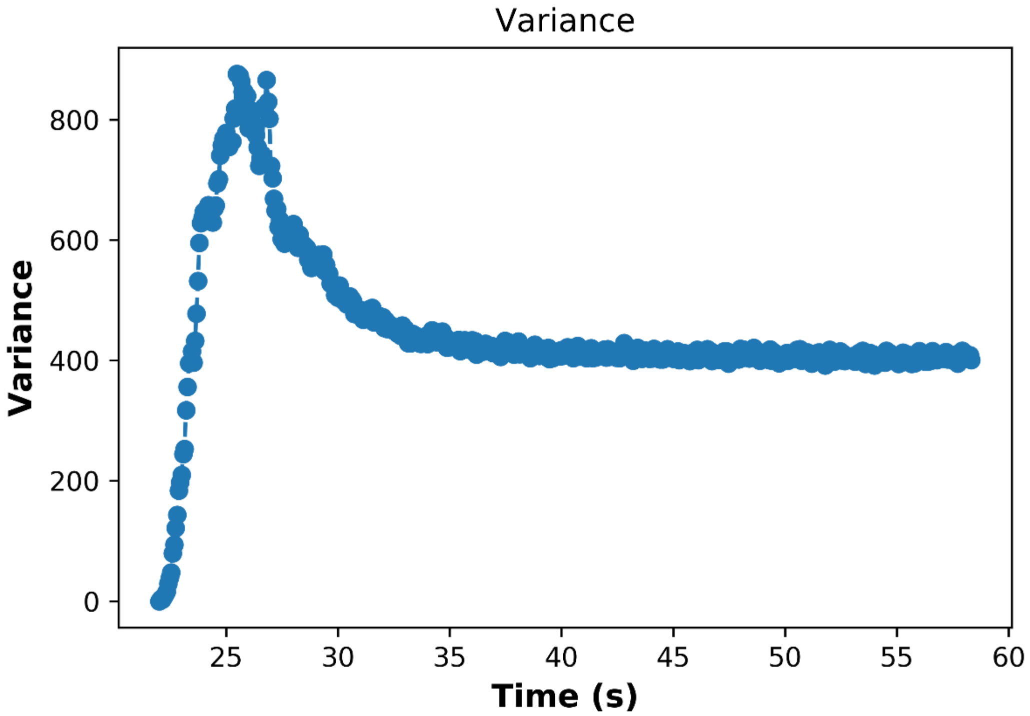

Variance
800
600
400
200
0
Variance
25
30
35
40
45
50
55
60
Time (s)

5 L STR, anchor, with probe

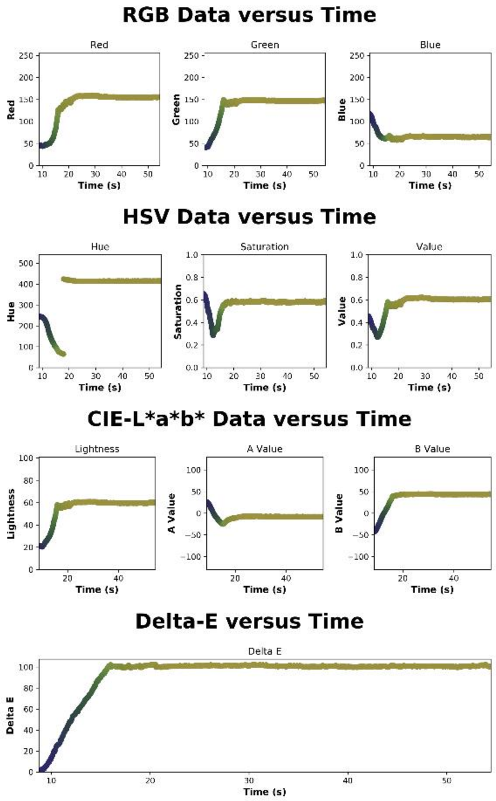

Selected Region of Interest

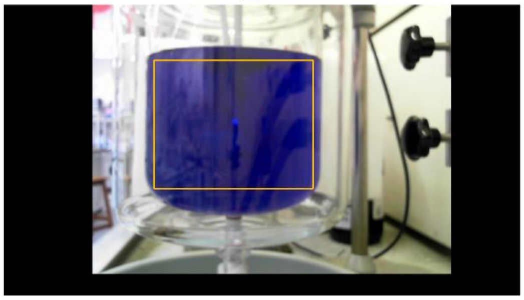

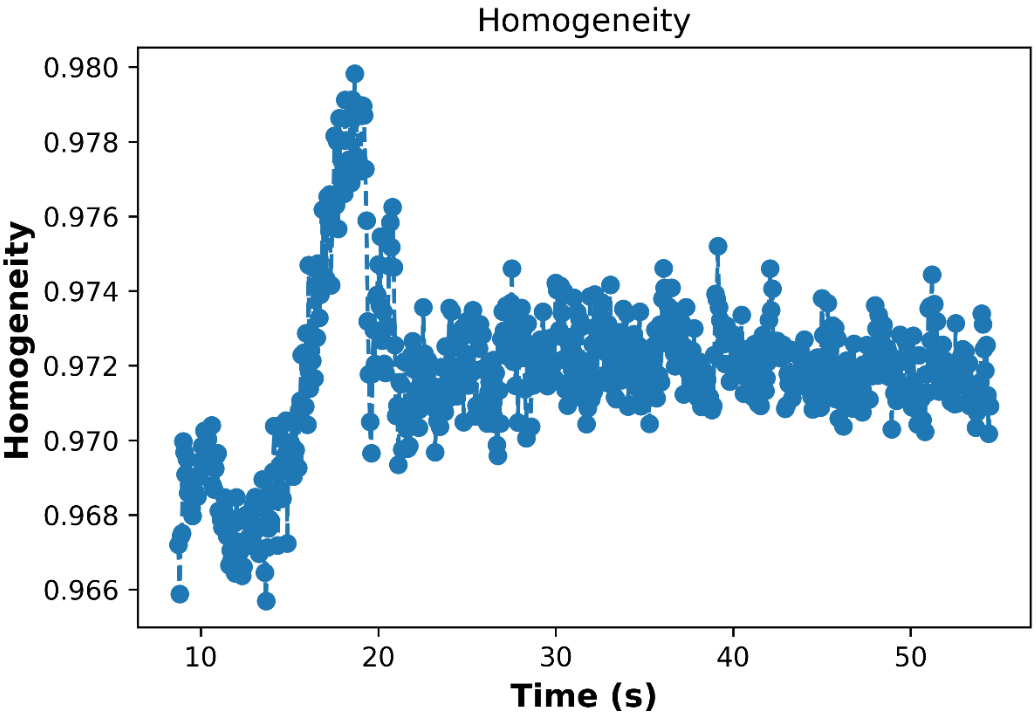

Homogeneity
0.980
0.978
0.976
0.974
0.972
0.970
0.968
0.966
Homogeneity
10
20
30
40
50
Time (s)

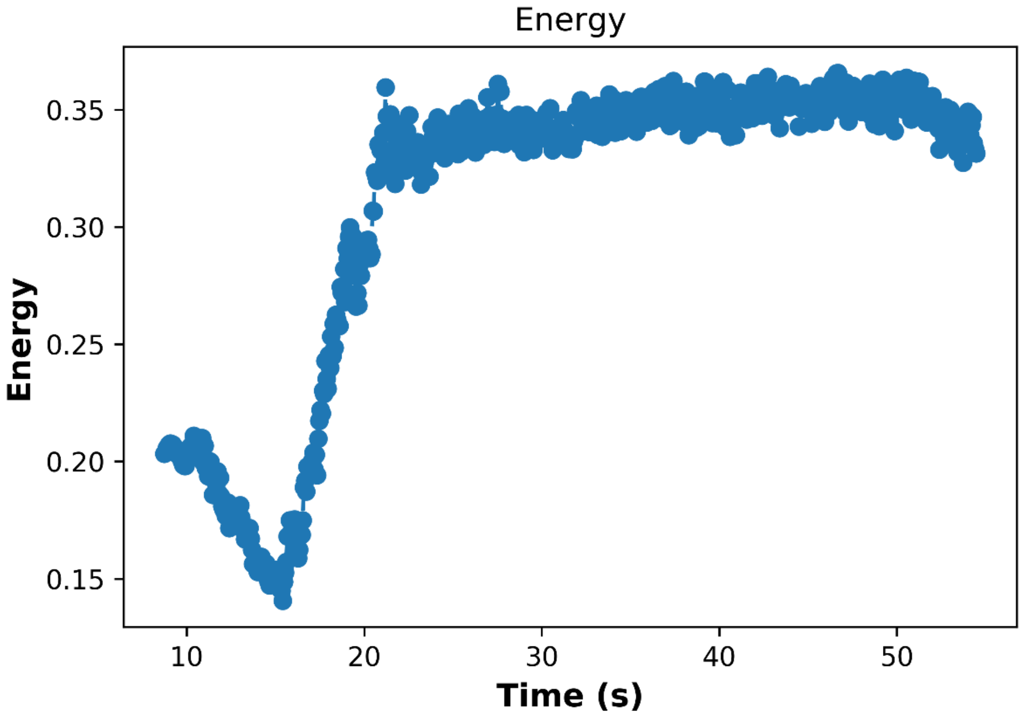

Energy
0.35
0.30
0.25
0.20
0.15
Energy
10
20
30
40
50
Time (s)

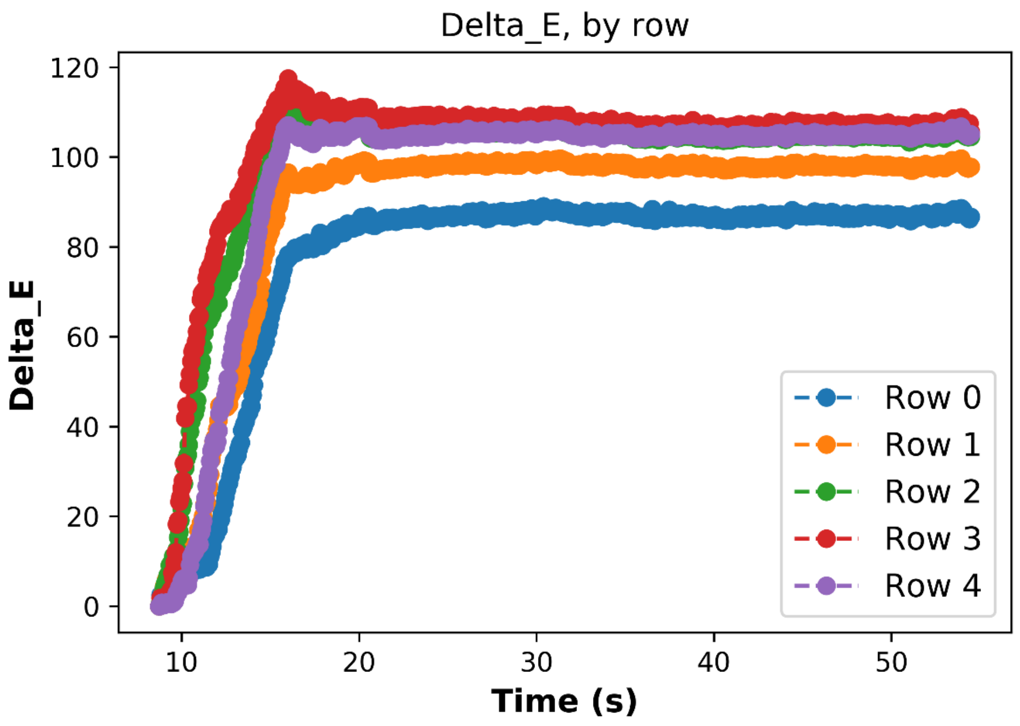
Delta_E, by row
Delta_E
Time (s)
Row 0
Row 1
Row 2
Row 3
Row 4
0
20
40
60
80
100
120
10
20
30
40
50

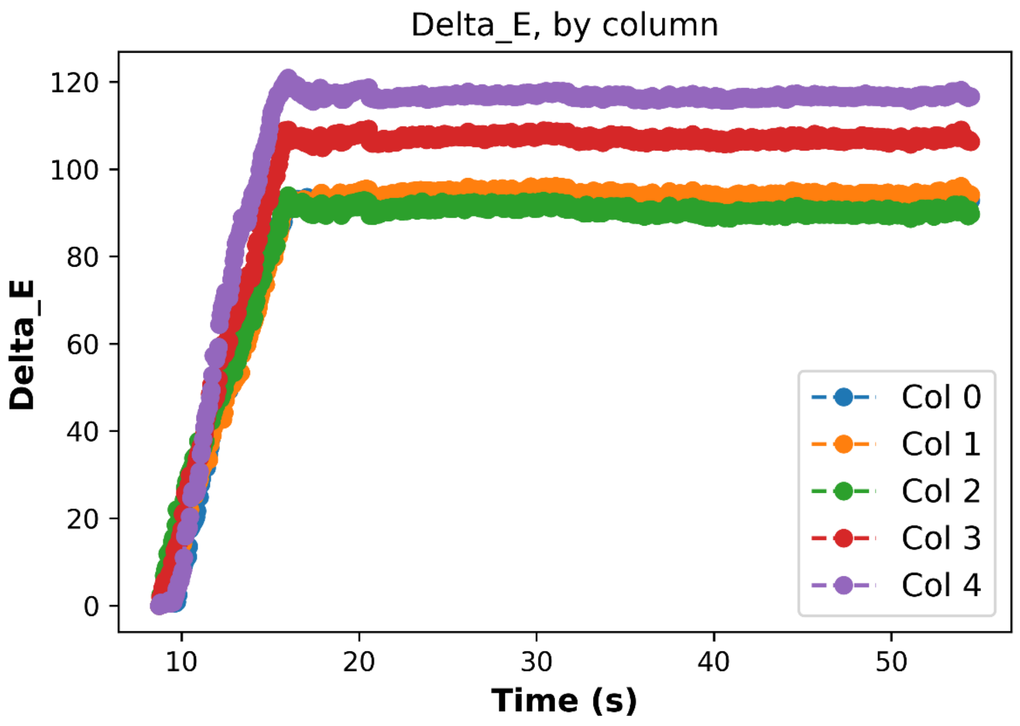
Delta_E, by column
Delta_E
Time (s)
Col 0
Col 1
Col 2
Col 3
Col 4
0
20
40
60
80
100
120
10
20
30
40
50

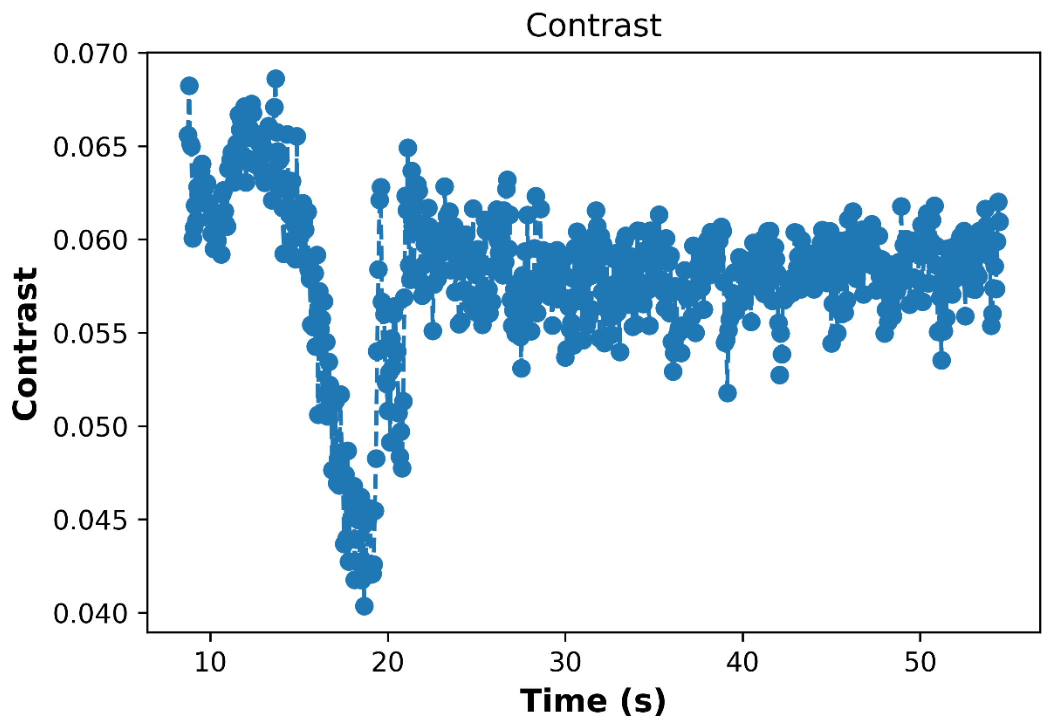
Contrast
0.070
0.065
0.060
0.055
0.050
0.045
0.040
Contrast
10
20
30
40
50
Time (s)

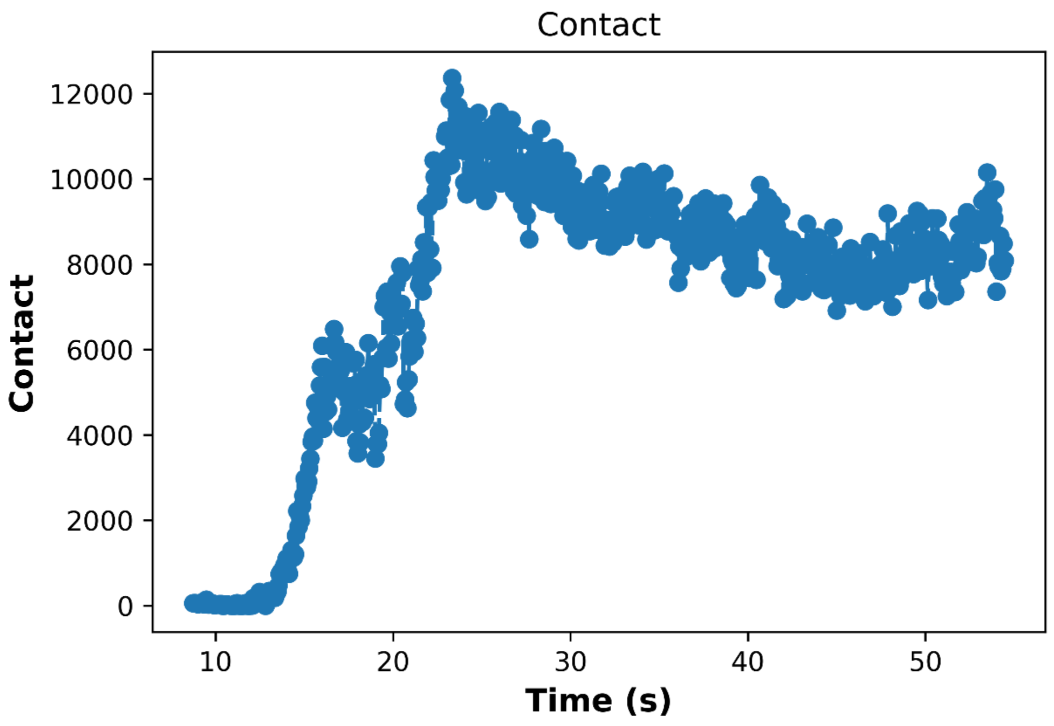
Contact
12000
10000
8000
6000
4000
2000
0
Contact
10
20
30
40
50
Time (s)

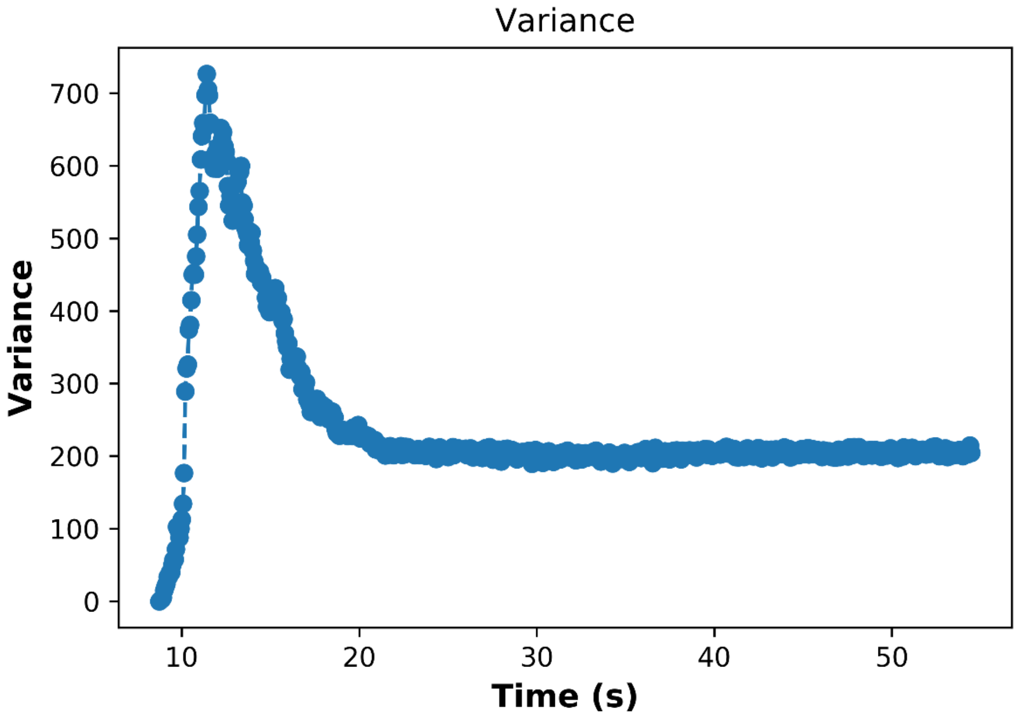

Variance
700
600
500
400
300
200
100
0
Variance
10
20
30
40
50
Time (s)

6. Colorimetric Kinetic Analysis of Competing, Mixing-sensitive Reactions

Scheme 13 in the manuscript attends comparative analysis of Villermaux-type competing reactions.

Below, the raw data from each reaction – with 50 RPM and 200 RPM stirring rates – are provided.

50 RPM stirring rate

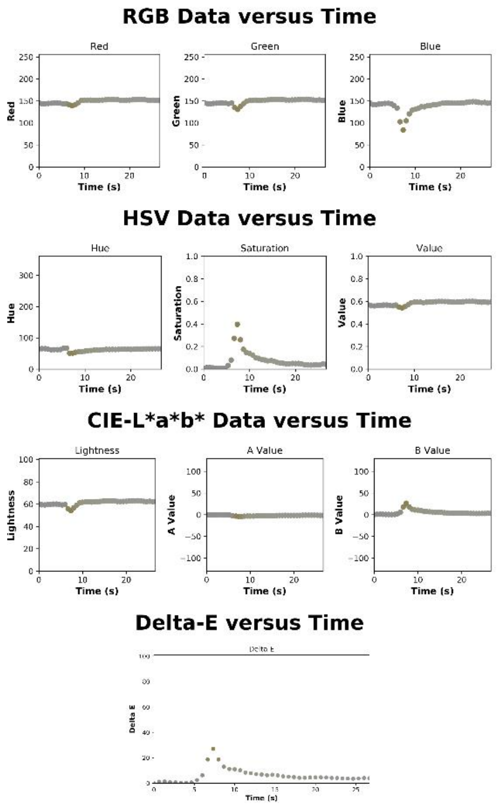

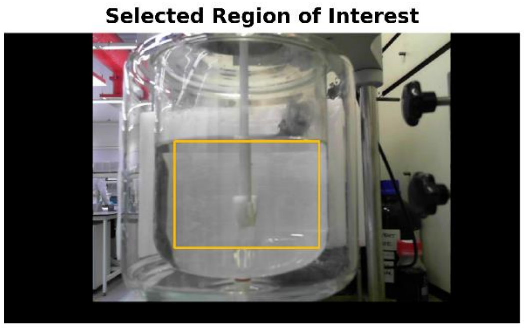

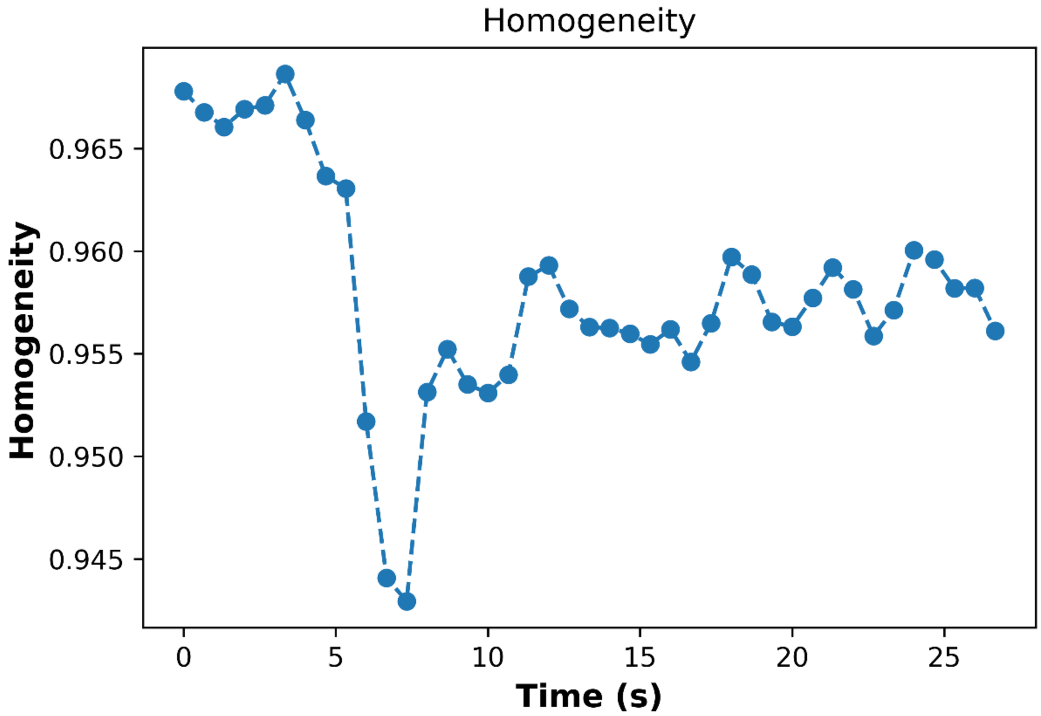
Homogeneity
Homogeneity
0.965
0.960
0.955
0.950
0.945
0
5
10
15
20
25
Time (s)

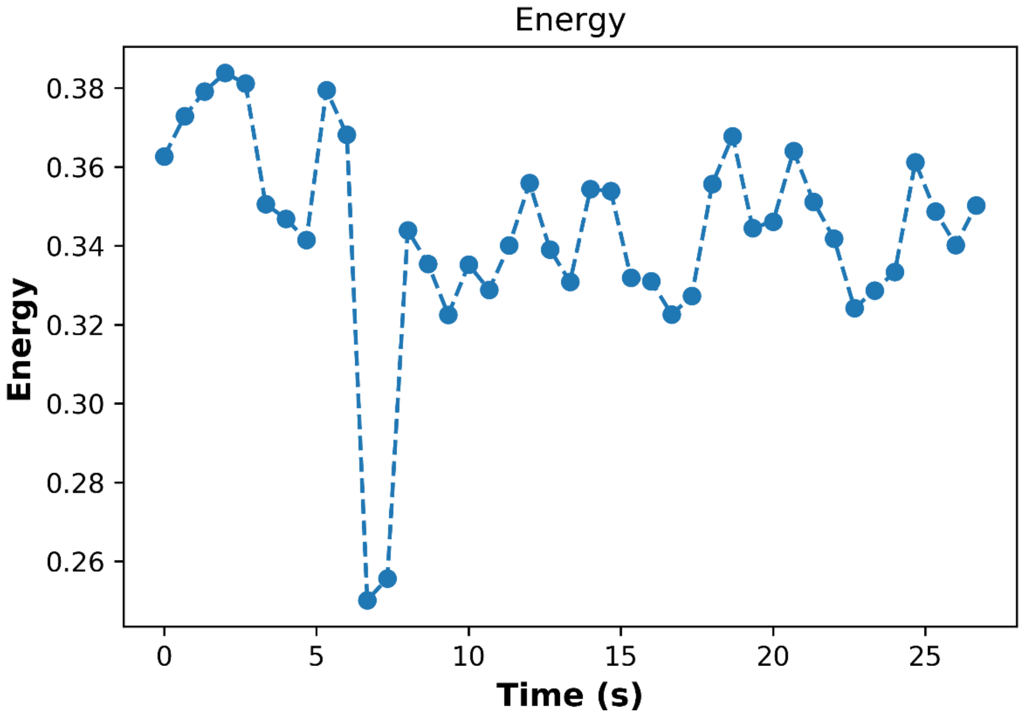
Energy
Energy
0.38
0.36
0.34
0.32
0.30
0.28
0.26
0
5
10
15
20
25
Time (s)

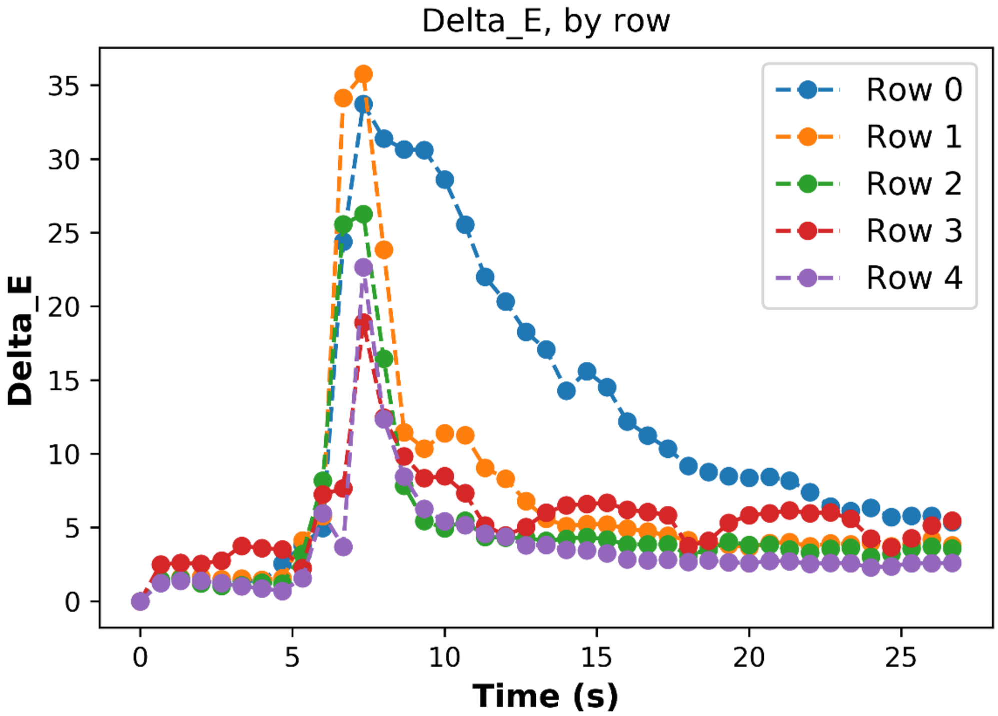

Delta_E, by row
Delta_E
Time (s)
Row 0
Row 1
Row 2
Row 3
Row 4
0
5
10
15
20
25
30
35
0
5
10
15
20
25

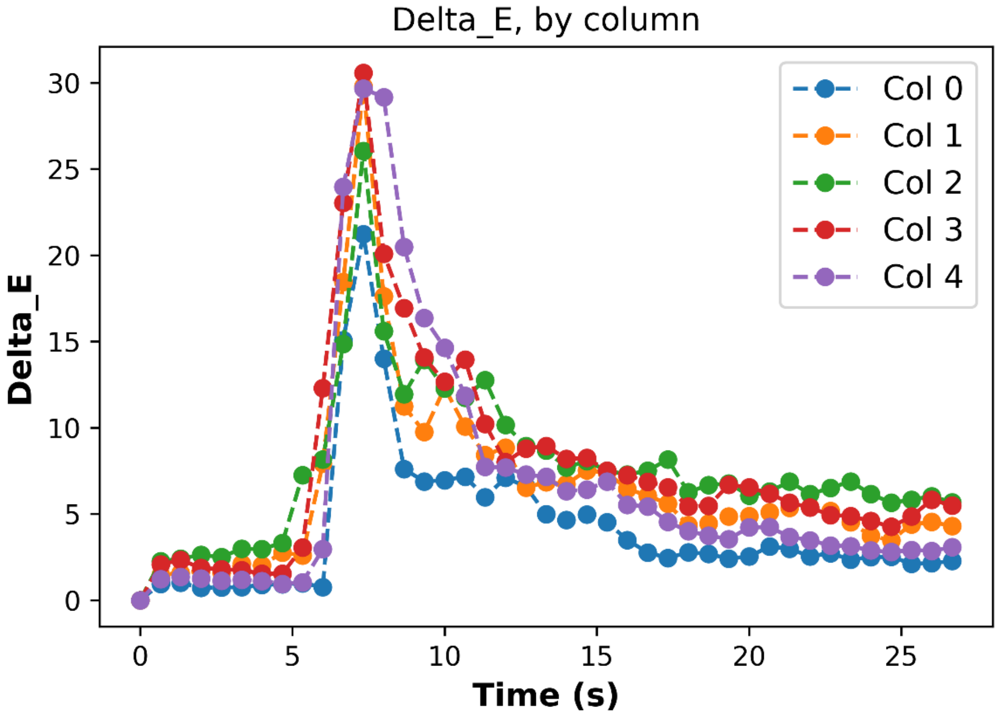

Delta_E, by column
Delta_E
Time (s)
Col 0
Col 1
Col 2
Col 3
Col 4
0
5
10
15
20
25
30
0
5
10
15
20
25

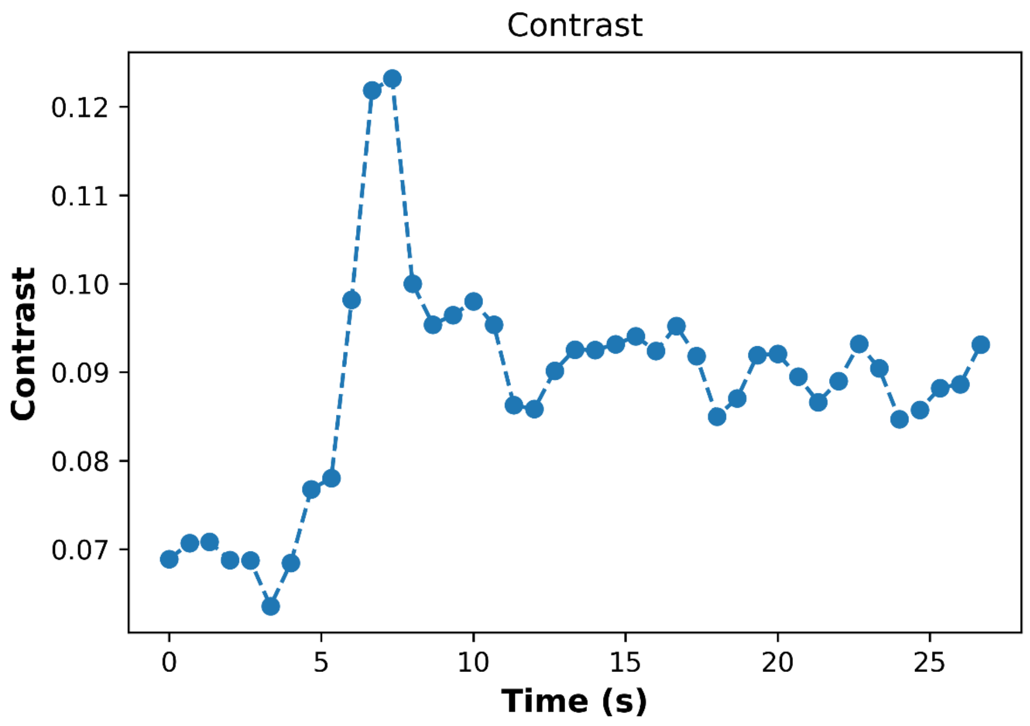

Contrast
0.12
0.11
0.10
0.09
0.08
0.07
Contrast
0
5
10
15
20
25
Time (s)

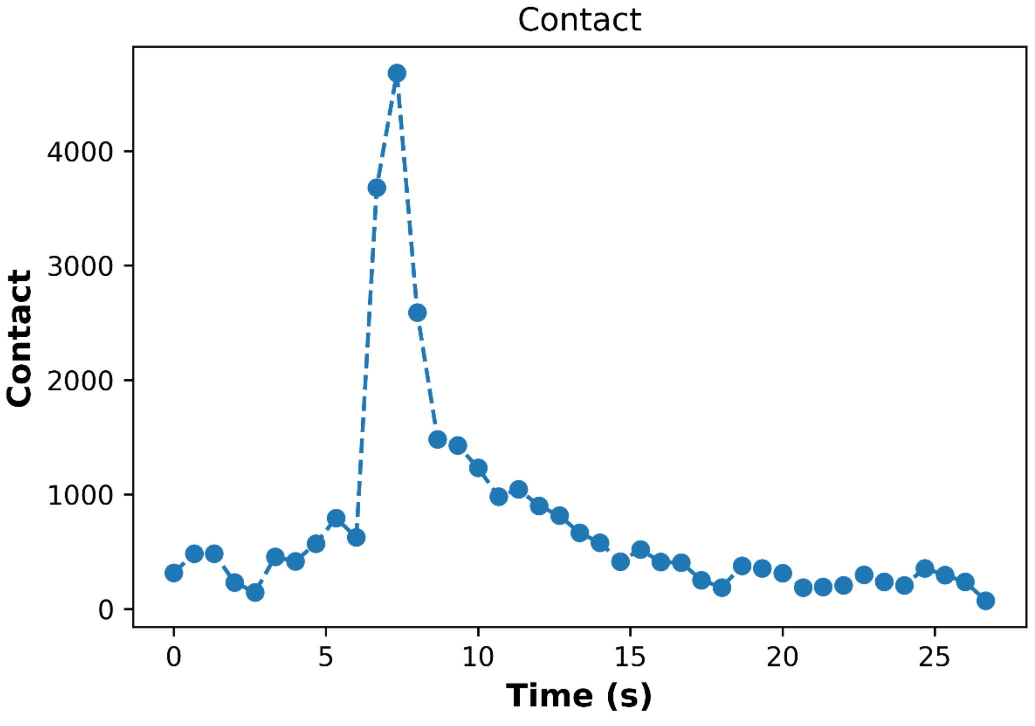

Contact
4000
3000
2000
1000
0
Contact
0
5
10
15
20
25
Time (s)

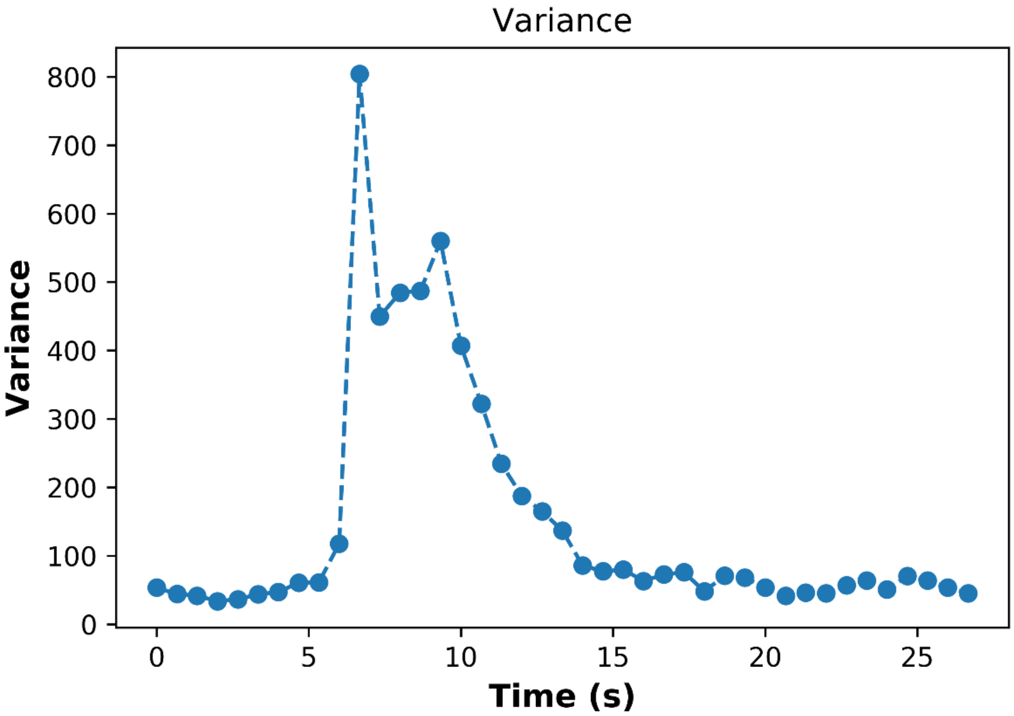
Variance
800
700
600
500
400
300
200
100
0
Variance
0
5
10
15
20
25
Time (s)

200 RPM stirring rate

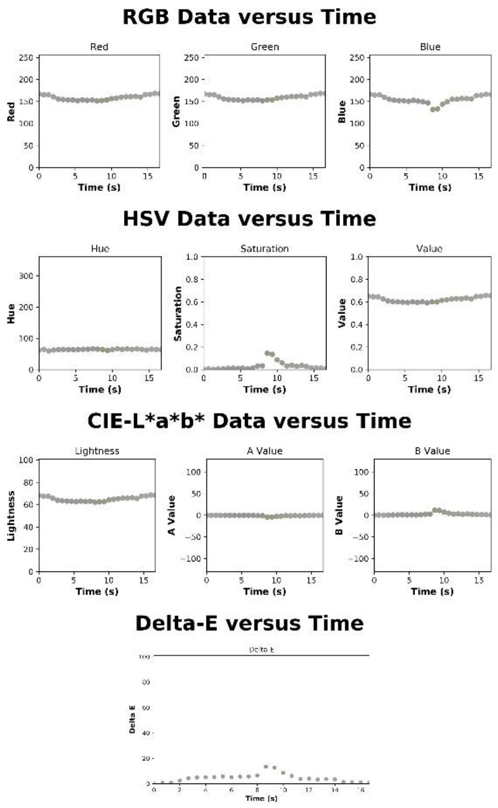

Kineticolor

Selected Region of Interest

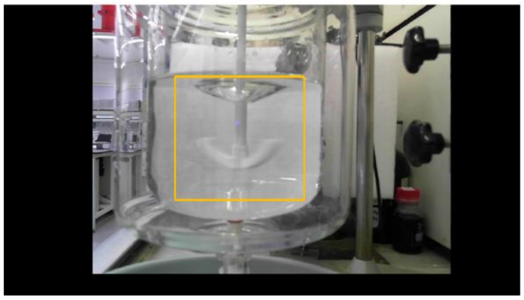

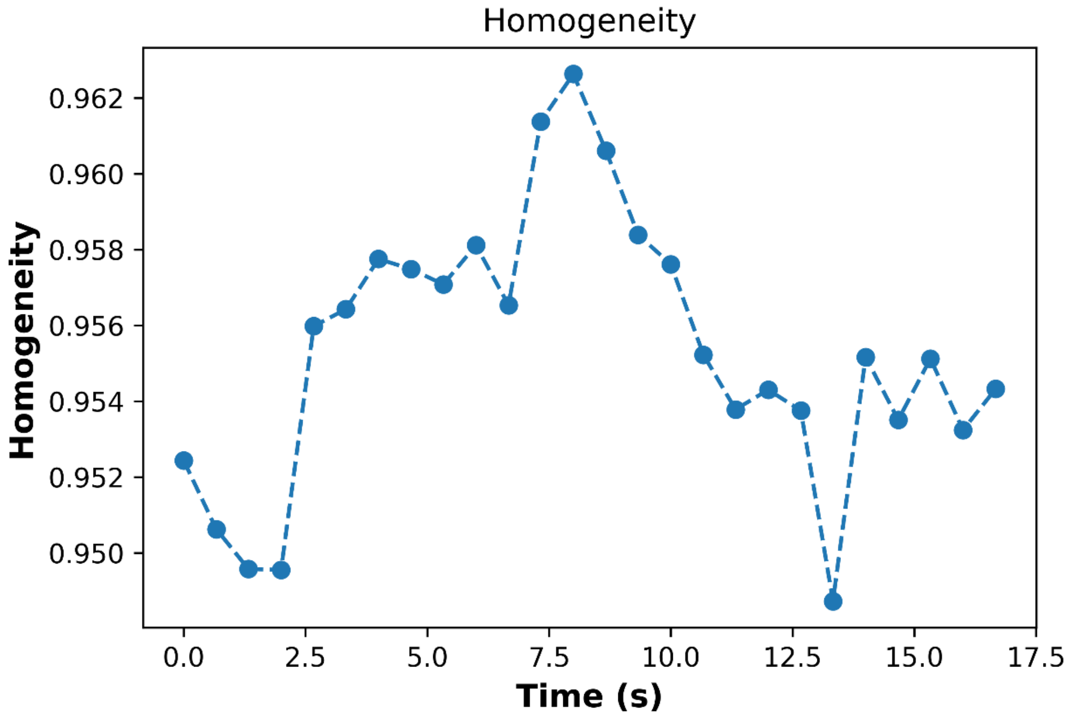
Homogeneity
0.962
0.960
0.958
0.956
0.954
0.952
0.950
Homogeneity
0.0
2.5
5.0
7.5
10.0
12.5
15.0
17.5
Time (s)

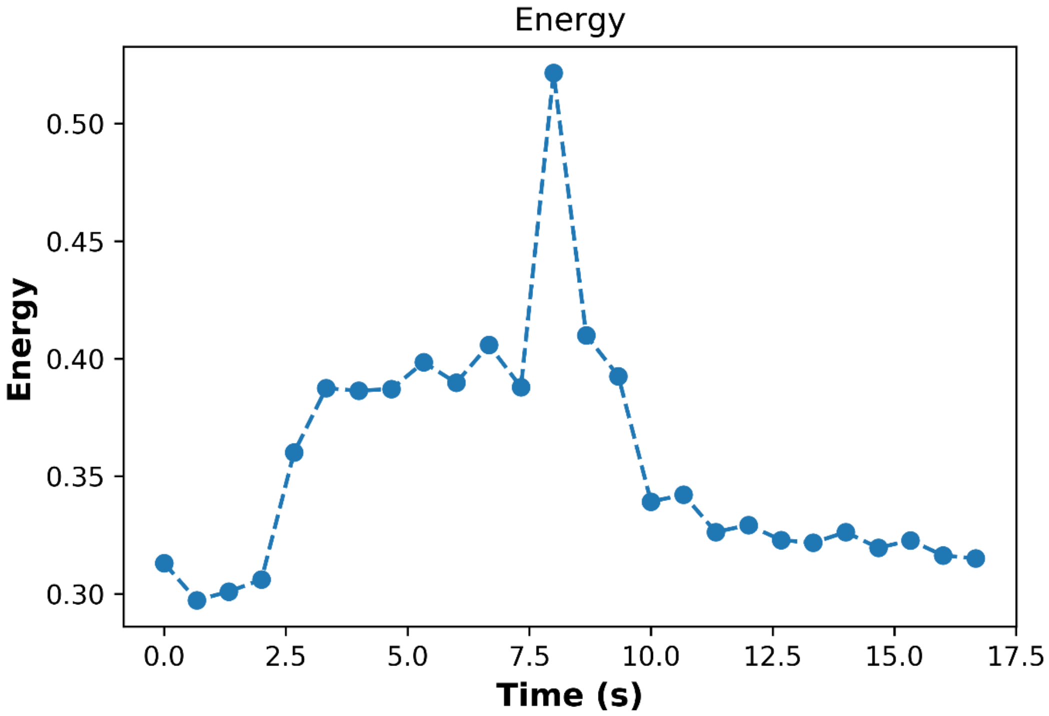
Energy
0.50
0.45
0.40
0.35
0.30
Energy
0.0
2.5
5.0
7.5
10.0
12.5
15.0
17.5
Time (s)

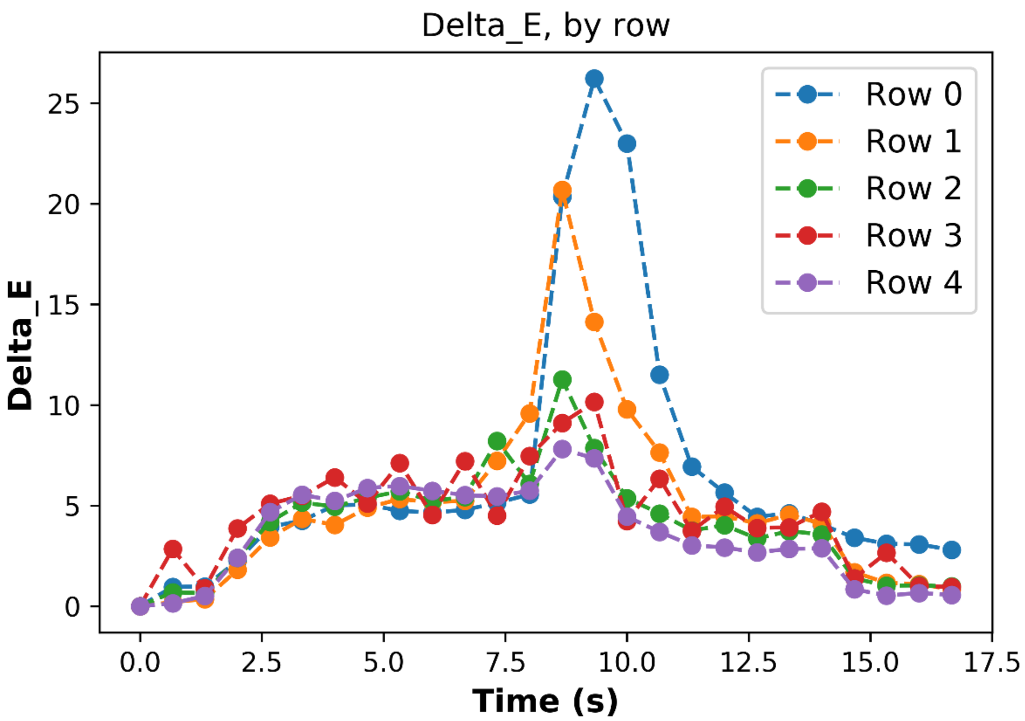

Delta_E, by row
Delta_E
Time (s)
Row 0
Row 1
Row 2
Row 3
Row 4
0
5
10
15
20
25
0.0
2.5
5.0
7.5
10.0
12.5
15.0
17.5

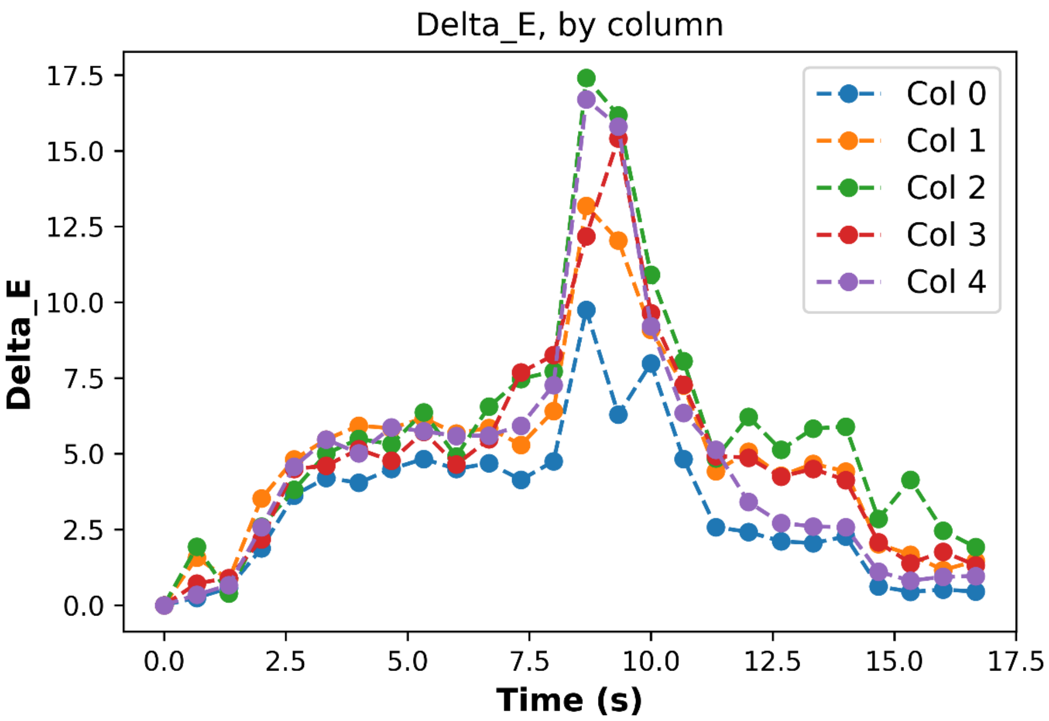

Delta_E, by column
Delta_E
Time (s)
Col 0
Col 1
Col 2
Col 3
Col 4
0.0
2.5
5.0
7.5
10.0
12.5
15.0
17.5

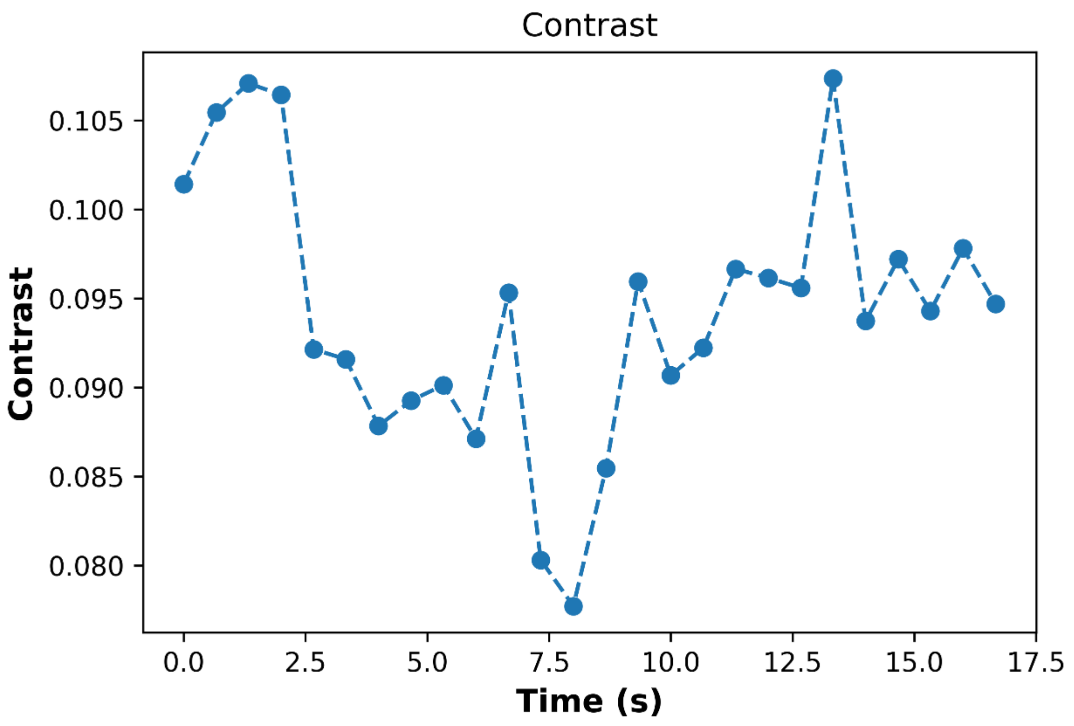
Contrast
0.105
0.100
0.095
0.090
0.085
0.080
Contrast
0.0
2.5
5.0
7.5
10.0
12.5
15.0
17.5
Time (s)

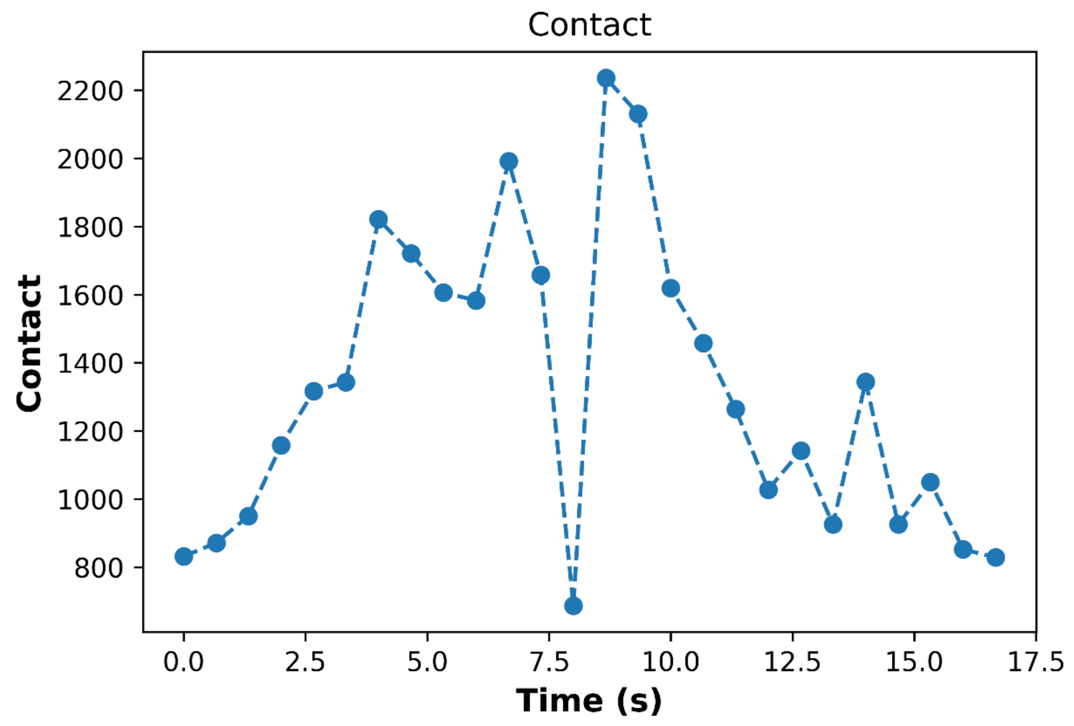
Contact
2200
2000
1800
1600
1400
1200
1000
800
Contact
0.0
2.5
5.0
7.5
10.0
12.5
15.0
17.5
Time (s)

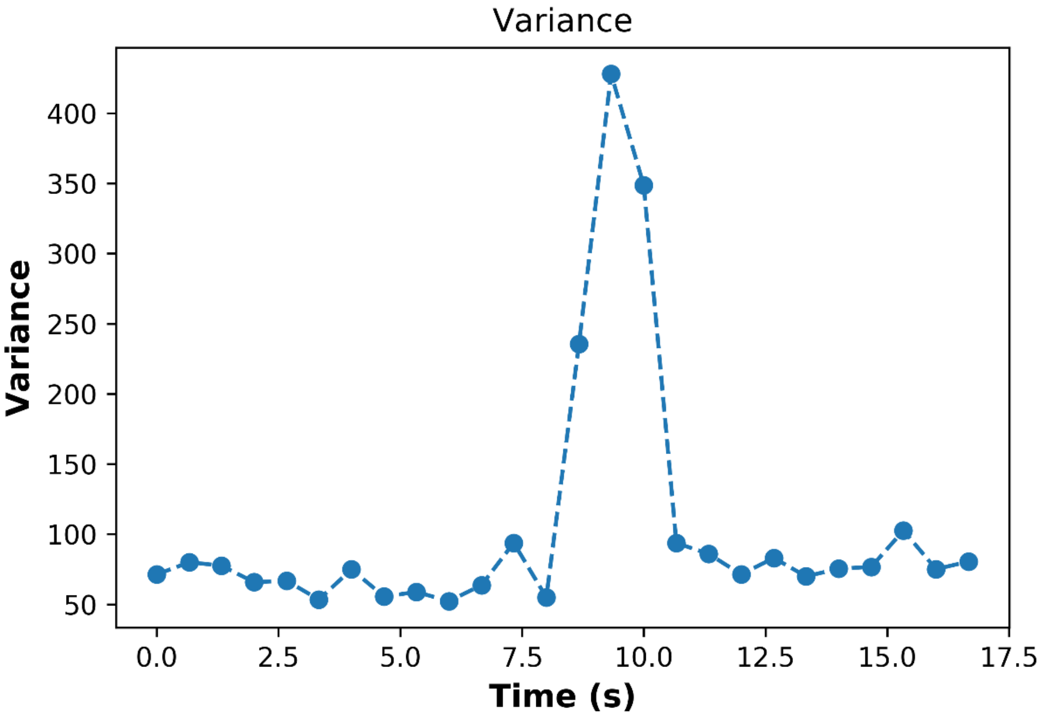

Variance
400
350
300
250
200
150
100
50
Variance
0.0
2.5
5.0
7.5
10.0
12.5
15.0
17.5
Time (s)

7. Application of Mixing Data in Highly Mixing Sensitive Chemistries

Schemes 14 and 15 attend S_NAr reactions run in a 5 L STR. The experimental set-up for these reactions is described in the Experimental SI.

Below, the raw imaging data for both reactions – run with 50 and 200 RPM stirring rates – are provided.

50 RPM

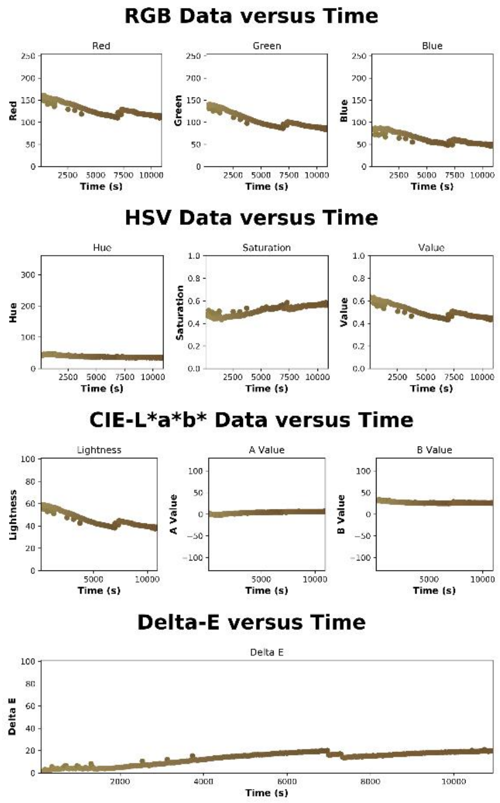

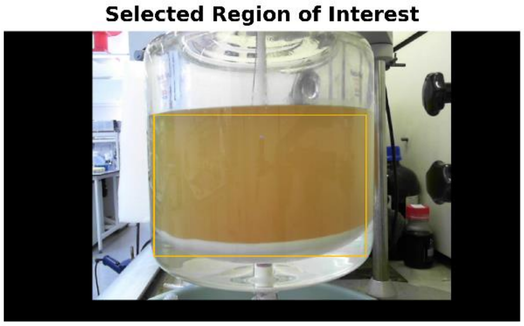

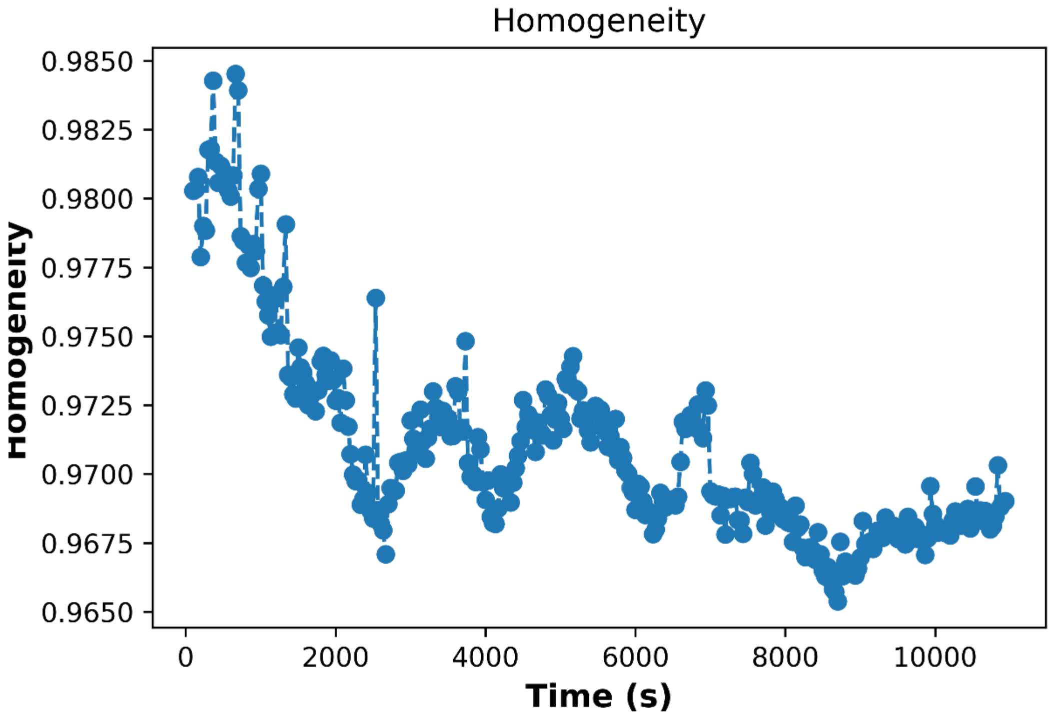
Homogeneity
0.9850
0.9825
0.9800
0.9775
0.9750
0.9725
0.9700
0.9675
0.9650
Homogeneity
0
2000
4000
6000
8000
10000
Time (s)

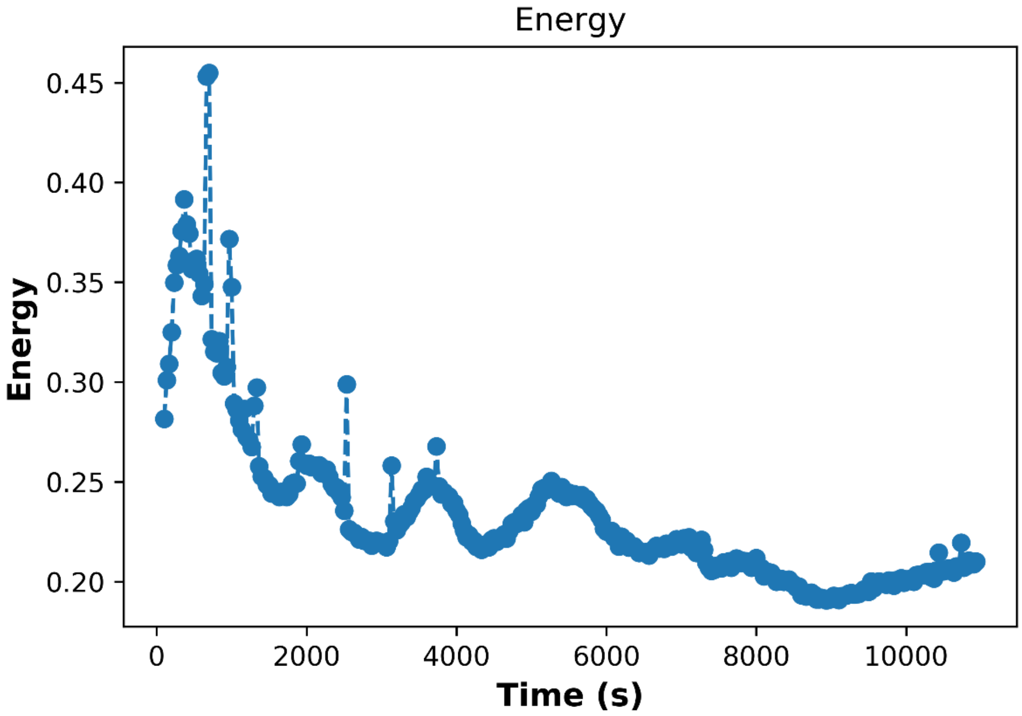
Energy
0.45
0.40
0.35
0.30
0.25
0.20
Energy
0
2000
4000
6000
8000
10000
Time (s)

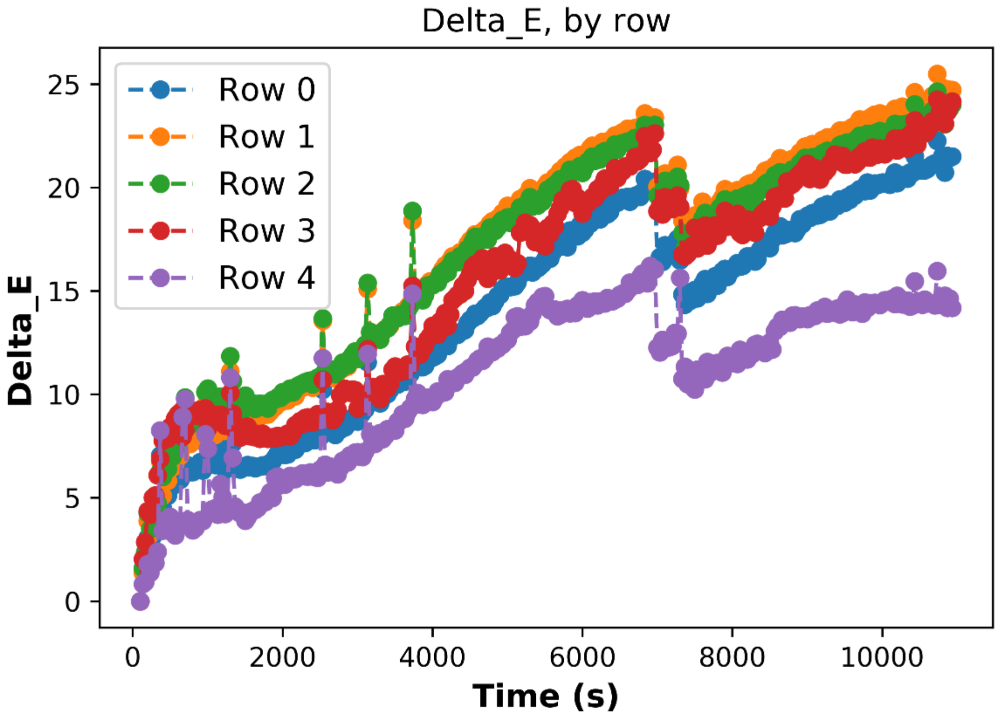

Delta_E, by row
Row 0
Row 1
Row 2
Row 3
Row 4
Delta_E
Time (s)
0
5
10
15
20
25
0
2000
4000
6000
8000
10000

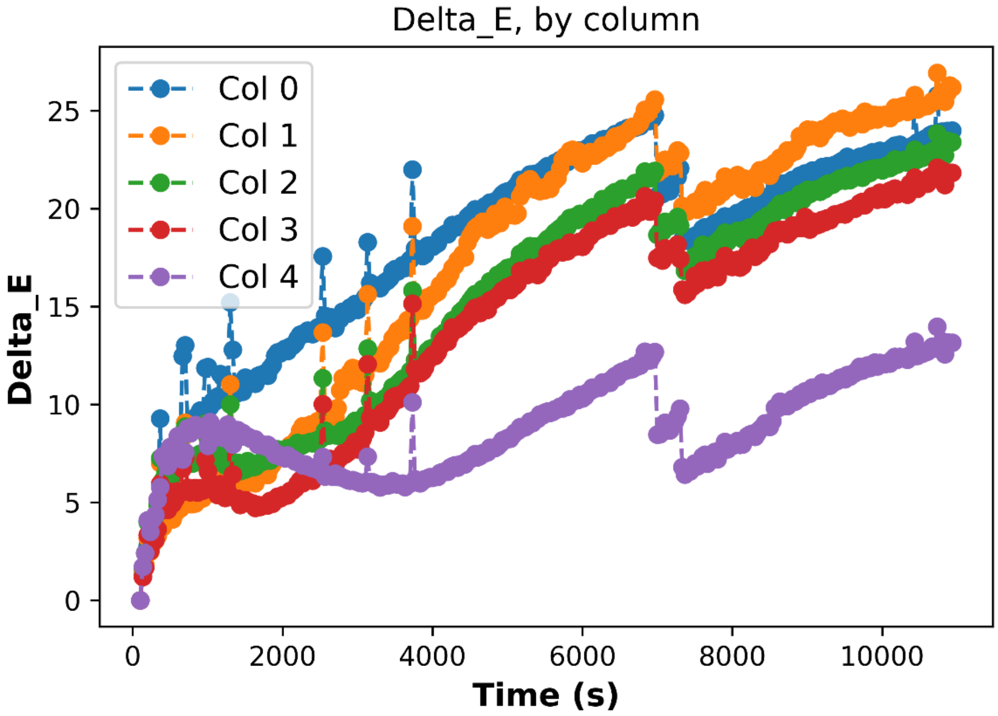

Delta_E, by column
Col 0
Col 1
Col 2
Col 3
Col 4
Delta_E
Time (s)
0
5
10
15
20
25
0
2000
4000
6000
8000
10000

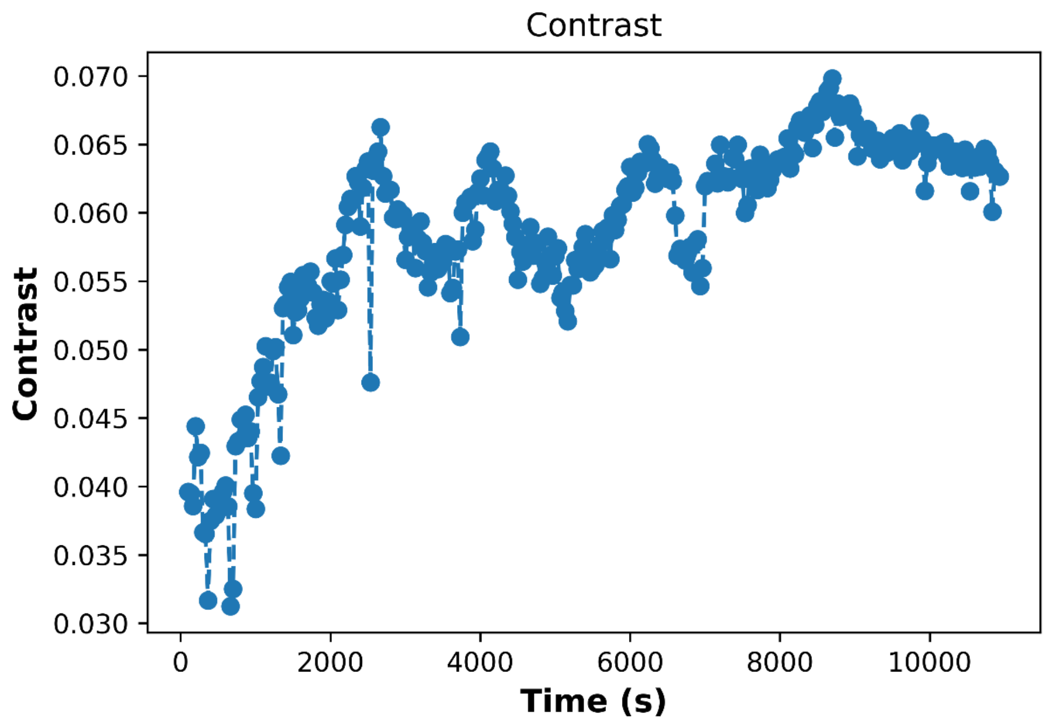

Contrast
Contrast
0.070
0.065
0.060
0.055
0.050
0.045
0.040
0.035
0.030
0
2000
4000
6000
8000
10000
Time (s)

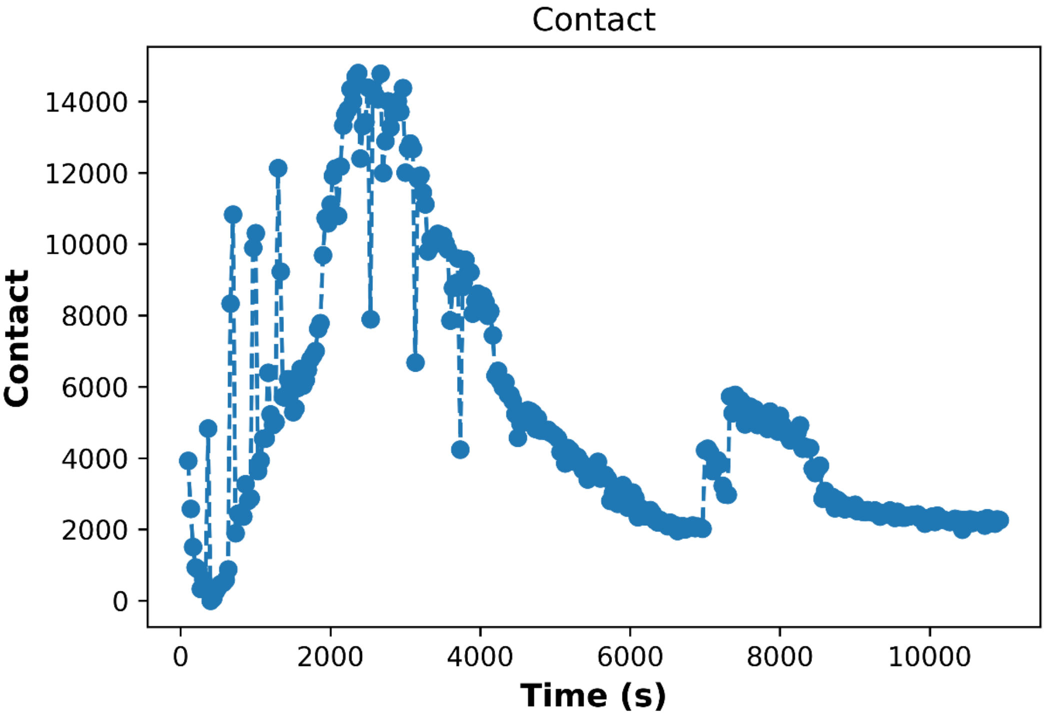

Contact
Contact
14000
12000
10000
8000
6000
4000
2000
0
0
2000
4000
6000
8000
10000
Time (s)

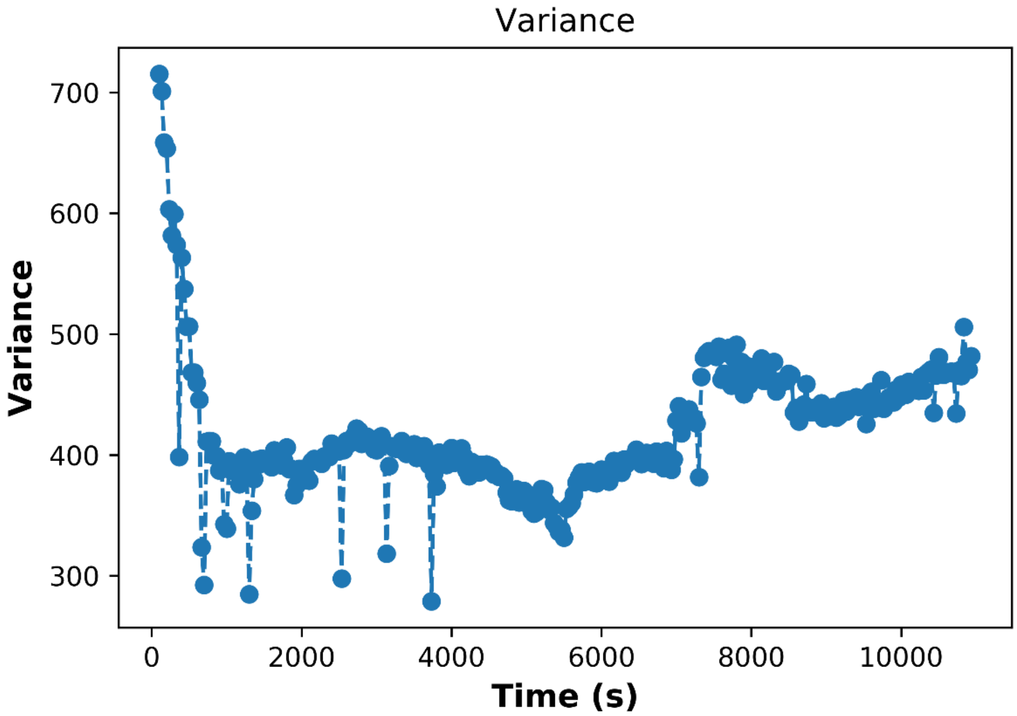

Variance
700
600
500
400
300
Variance
0
2000
4000
6000
8000
10000
Time (s)

188 RPM (full reactor analysis)

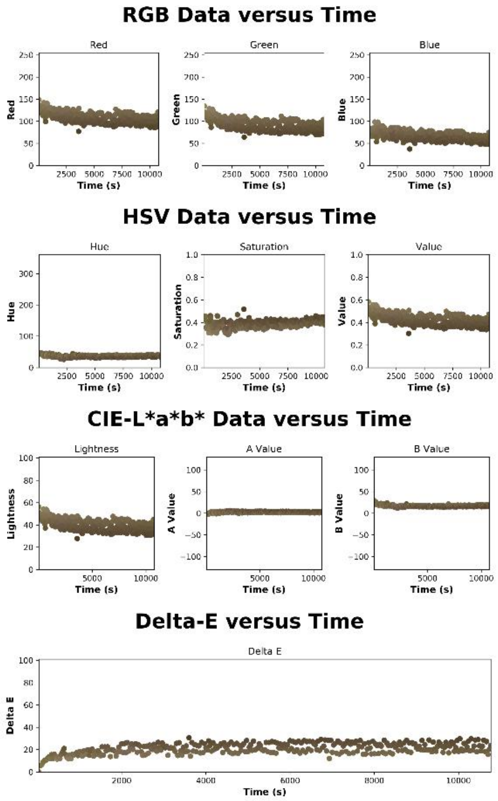

Kineticolor

Selected Region of Interest

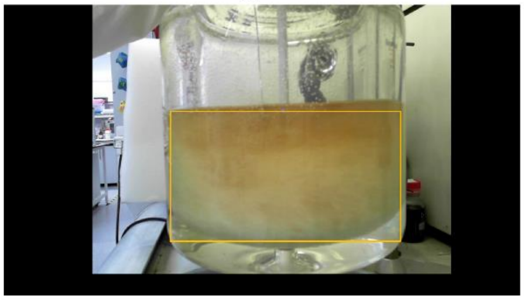

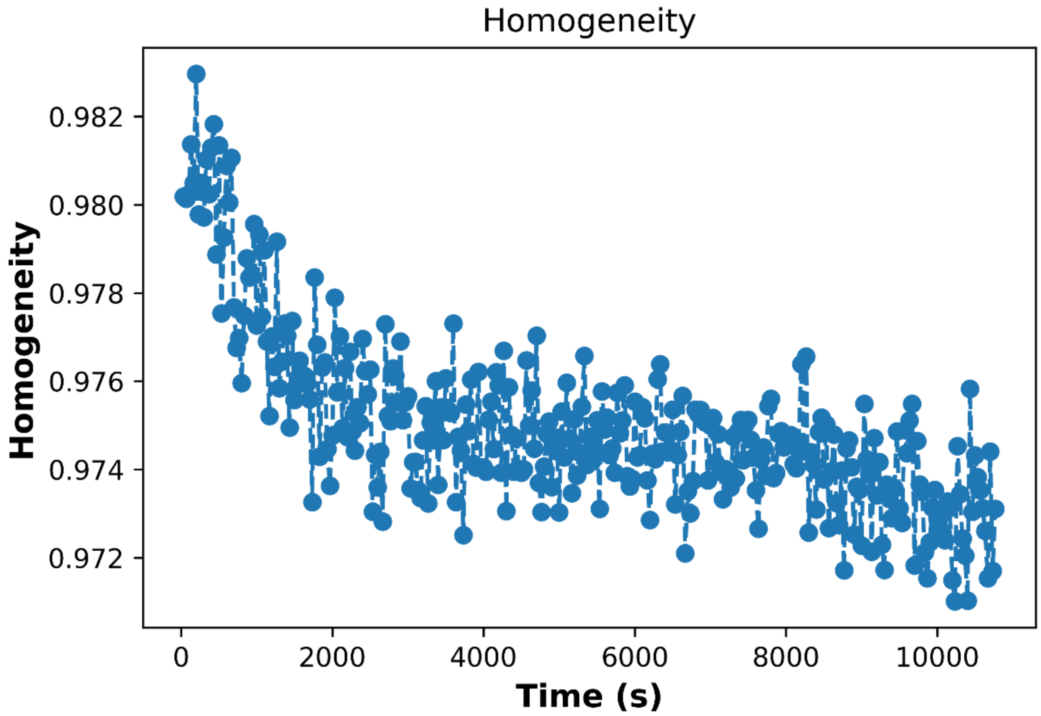
Homogeneity
0.982
0.980
0.978
0.976
0.974
0.972
Homogeneity
0
2000
4000
6000
8000
10000
Time (s)

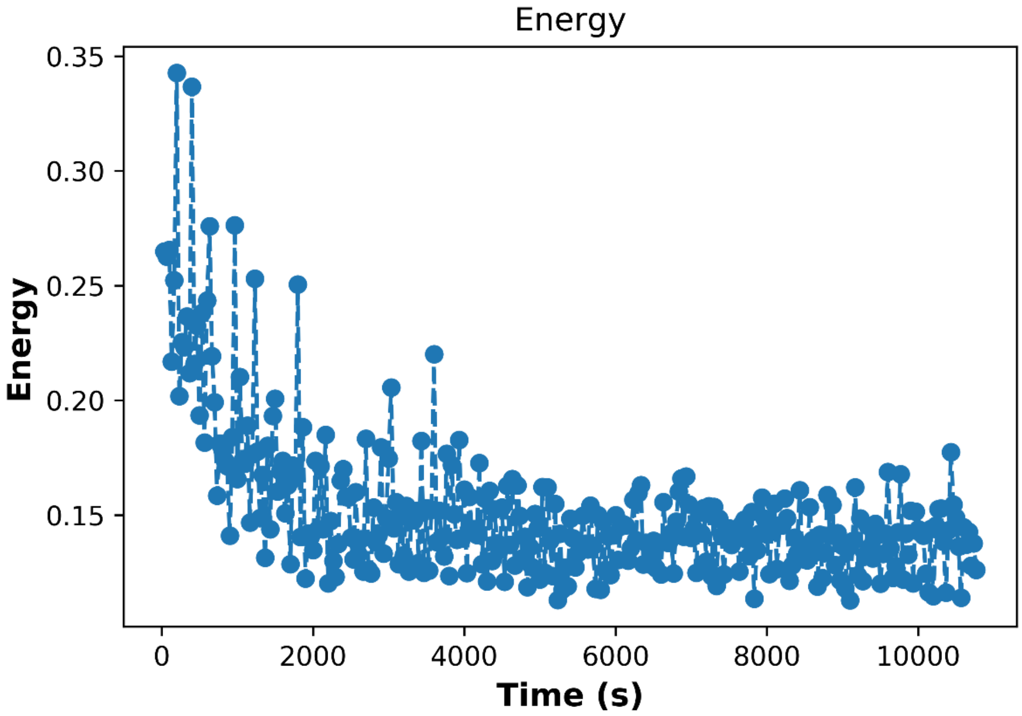
Energy
0.35
0.30
0.25
0.20
0.15
Energy
0
2000
4000
6000
8000
10000
Time (s)

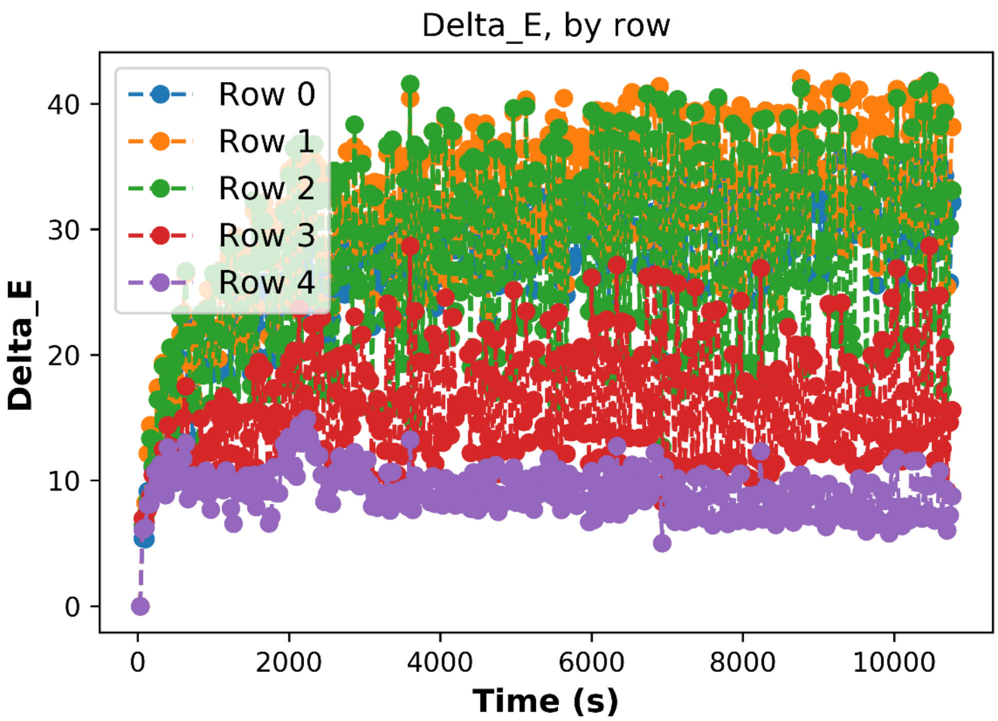
Delta_E, by row
Row 0
Row 1
Row 2
Row 3
Row 4
Delta_E
Time (s)
0
10
20
30
40
0
2000
4000
6000
8000
10000

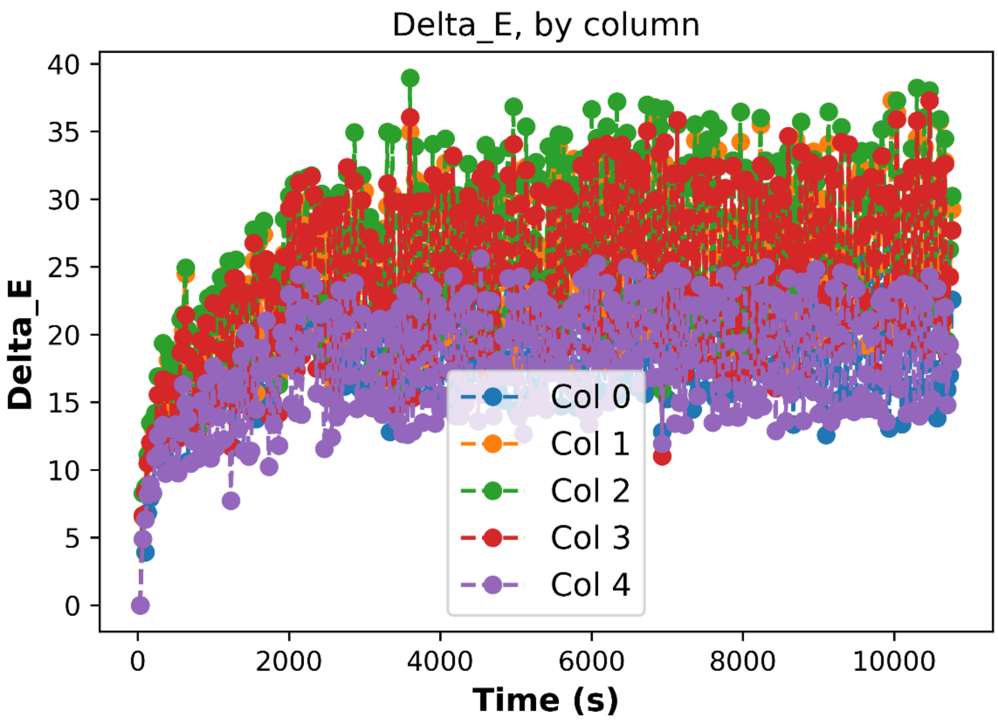
Delta_E, by column
Col 0
Col 1
Col 2
Col 3
Col 4
Delta_E
Time (s)
0
5
10
15
20
25
30
35
40
0
2000
4000
6000
8000
10000

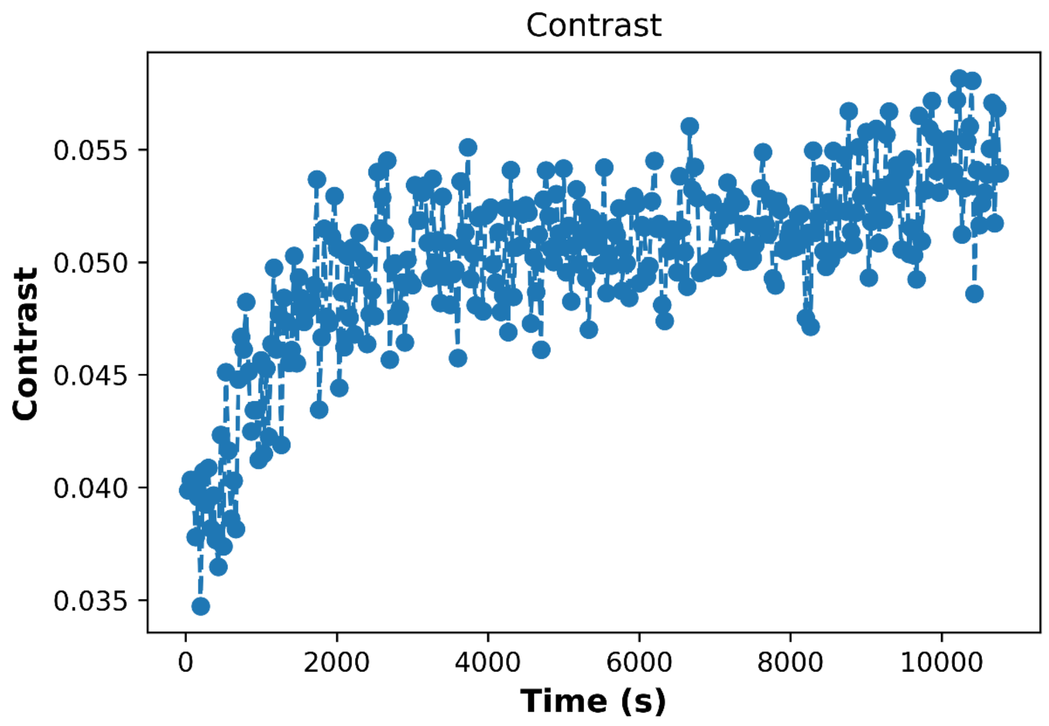
Contrast
0.055
0.050
0.045
0.040
0.035
Contrast
0
2000
4000
6000
8000
10000
Time (s)

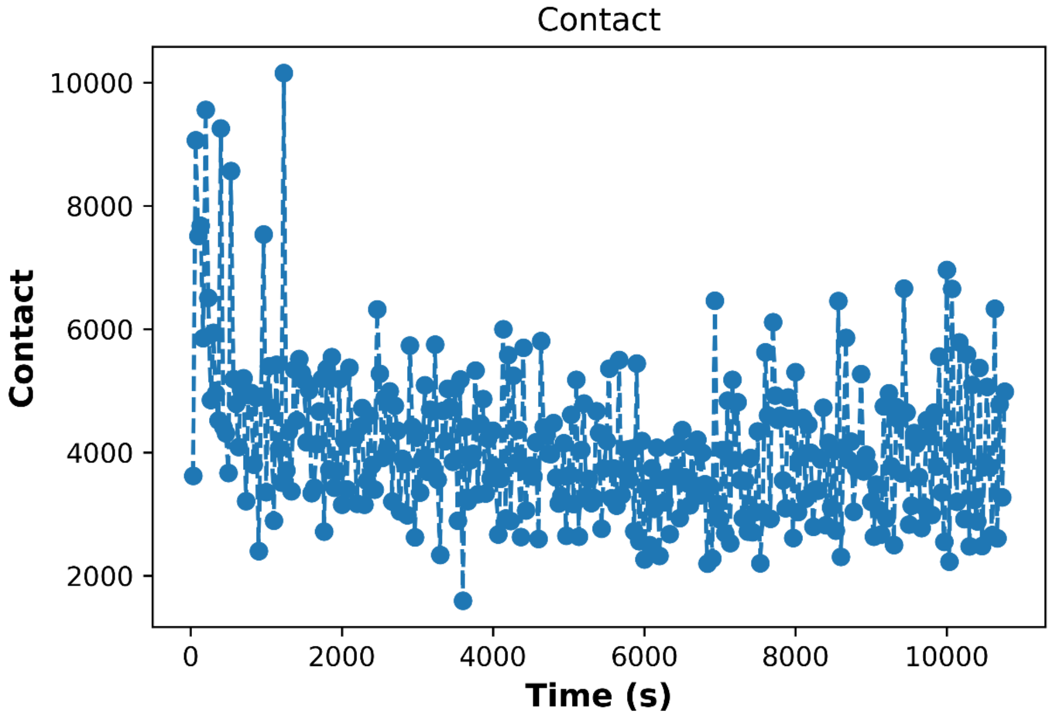
Contact
10000
8000
6000
4000
2000
Contact
0
2000
4000
6000
8000
10000
Time (s)

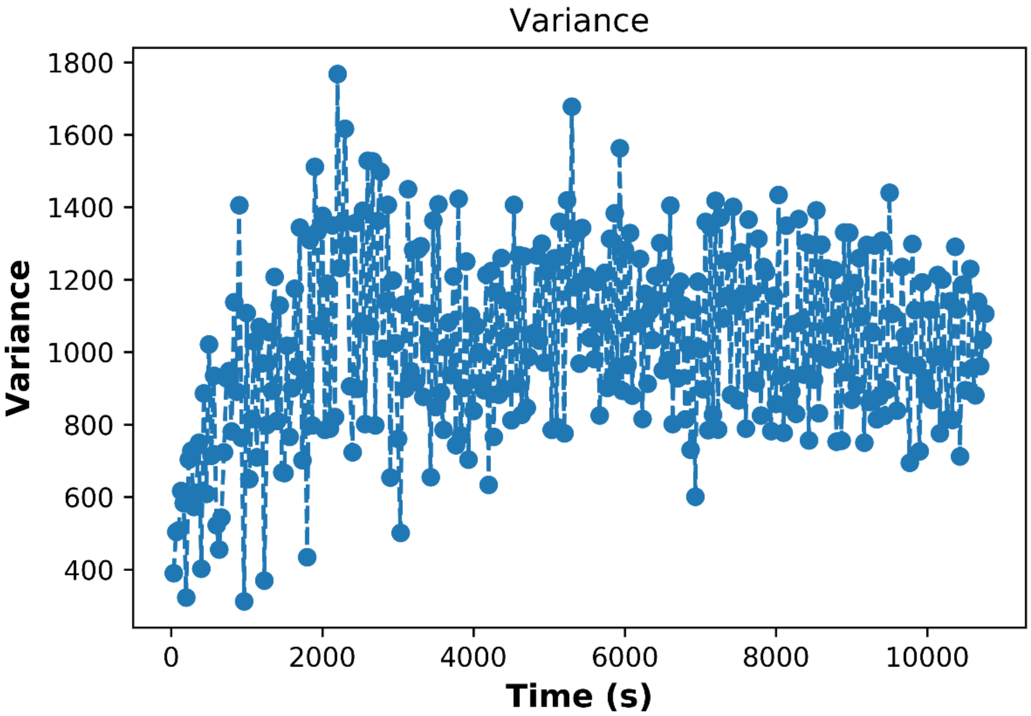
Variance
1800
1600
1400
1200
1000
800
600
400
Variance
0
2000
4000
6000
8000
10000
Time (s)

188 RPM (top 'layer' analysis; average colour analysis only)

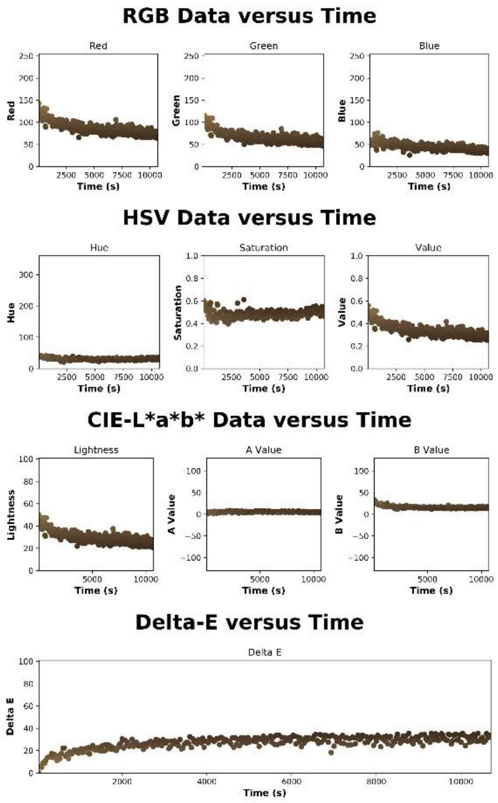

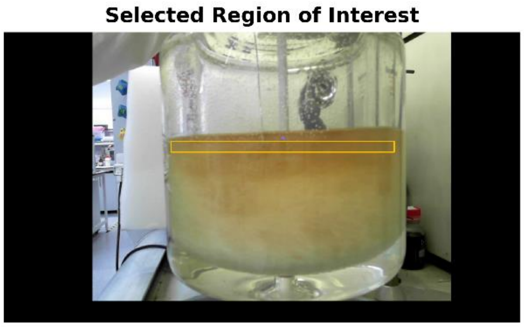

8. Mutual Information and Color-Concentration Regression Analyses

Mutual information analysis was applied as first described in our previous publication.[1]

Collected spreadsheet inputs, scripts, and outputs for the analysis are provided in a zipped folder as part of the supporting information collection.

8.1 Code for Mutual Information Calculations

The following code is also available in mutual_info.py in the **Collected Spreadsheet Outputs** zipped folder, as part of the supporting information suite.

```
import numpy as np
import pandas as pd
import matplotlib.pyplot as plt
from sklearn.feature_selection import f_regression,
mutual_info_regression

data = pd.read_csv("mi_input.csv")
data_np = data.to_numpy()
output = pd.DataFrame()

def mi_plot(data):

    fig = plt.figure()
    ax = fig.add_axes([0,0,1,1])
    ax.barh(list(data.index),data['MI NORM'])
    ax.set_yticklabels
    plt.show()
    fig.savefig('normalised MI.png', facecolor='w',dpi=600)


for i in range(data_np.shape[1]):
    x = data_np[:,i]
    x = x.reshape(-1,1)
    y = data_np[:,-1]
    y = y.reshape(-1,1)
    mi = mutual_info_regression(x, y)

    output[str(data.columns[i])+"_MI"] = mi
```

```
output = output.transpose()
print('output = ',output)
mi_max = max(output.iloc[:,0])
print('mi_max = ' ,mi_max)
print('mi = ',mi)

mi_norm = []
for mi in output.iloc[:,0]:

    mi_norm.append(mi/float(mi_max))

    print('current list = ', mi_norm)

output['MI NORM'] = mi_norm

output = output.sort_values(by=['MI NORM'])

output.to_csv("mi_output.csv")
mi_plot(output)
```

9. Further Details on Video Analysis

Video analysis was performed using the developmental video analysis software, *Kineticolor*. An early electrochemically-focused application of this software has been reported in the literature.[2] All video data in this report were analyzed using the March 2022 version of the Kineticolor software. A licensed version of the software is available on request.

All videos were analyzed by breaking videos into their constituent frames, and each frame being analyzed, at the pixel level, in turn. A user-selected region of interest was analyzed, averaging all pixel values in the selected range. All background data outside the selected region was ignored in the analysis. Data were analyzed according to a user-selected number of frames to be skipped. Additional spatial analysis of each video

frame was carried out in accordance with the mathematics and workflow described in **Sections 1 and 2 (above)**.

The analyzed frames were curated in plots of various color or mixing components versus time to enable semi-quantitative and comparative kinetic analysis between different video analysis datasets.

Extracted color data were provided from across a common subset of color models, namely: RGB, HSV, CIE-L*a*b*, and CIE-XYZ. For the purposes of this report, analysis primarily focused on plots of Delta E versus time. Delta E is the color-independent measure of contrast, measured as the Euclidean distance between two colors in the CIE_L*a*b* color space.[3]

10. References

1 C. Yan, M. Cowie, C. Howcutt, K. M. Wheelhouse, N. S. Hodnett, M. Kollie, M. Gildea, M. H. Goodfellow and M. Reid, *ChemRxiv*, , DOI:10.26434/chemrxiv-2022-n0wf3.

2 A. G. Wills, D. L. Poole, C. M. Alder and M. Reid, *ChemElectroChem*, 2020, **7**, 2771–2776.

3 L. F. Capitán-Vallvey, N. López-Ruiz, A. Martínez-Olmos, M. M. Erenas and A. J. Palma, *Anal. Chim. Acta*, 2015, **899**, 23–56.
