# Supplementary figures and images for: Computer Vision for Kinetic Analysis of Lab- and Process-Scale Mixing Phenomena (part 2 of 2)
Source: Org Process Res Dev. 2022 Nov 4;26(11):3073–88. doi: 10.1021/acs.oprd.2c00216 (PMC9680030; doi:10.1021/acs.oprd.2c00216)

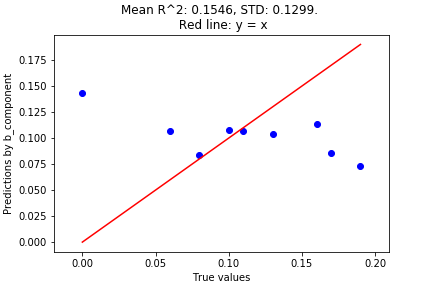

Supplement: Supplementary file 3 — op2c00216_si_005.zip [file op2c00216_si_005.zip › Collected spreadsheet outputs/Scheme 15 - statistical analysis/50 RPM/cross validation/b_component_CV_LOO.png]

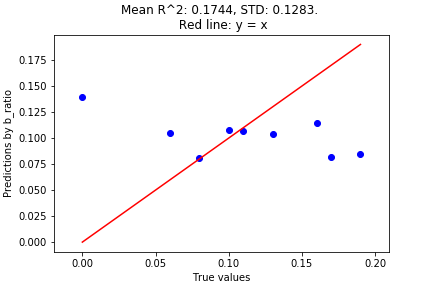

Supplement: Supplementary file 3 — op2c00216_si_005.zip [file op2c00216_si_005.zip › Collected spreadsheet outputs/Scheme 15 - statistical analysis/50 RPM/cross validation/b_ratio_CV_LOO.png]

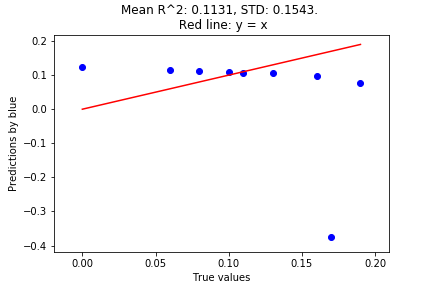

Supplement: Supplementary file 3 — op2c00216_si_005.zip [file op2c00216_si_005.zip › Collected spreadsheet outputs/Scheme 15 - statistical analysis/50 RPM/cross validation/blue_CV_LOO.png]

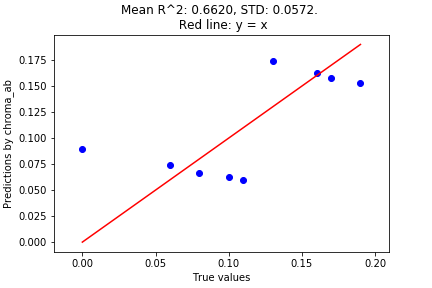

Supplement: Supplementary file 3 — op2c00216_si_005.zip [file op2c00216_si_005.zip › Collected spreadsheet outputs/Scheme 15 - statistical analysis/50 RPM/cross validation/chroma_ab_CV_LOO.png]

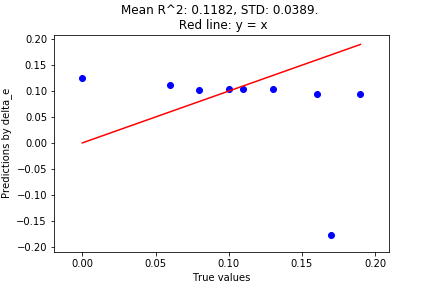

Supplement: Supplementary file 3 — op2c00216_si_005.zip [file op2c00216_si_005.zip › Collected spreadsheet outputs/Scheme 15 - statistical analysis/50 RPM/cross validation/delta_e_CV_LOO.png]

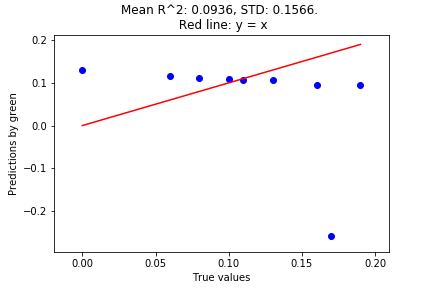

Supplement: Supplementary file 3 — op2c00216_si_005.zip [file op2c00216_si_005.zip › Collected spreadsheet outputs/Scheme 15 - statistical analysis/50 RPM/cross validation/green_CV_LOO.png]

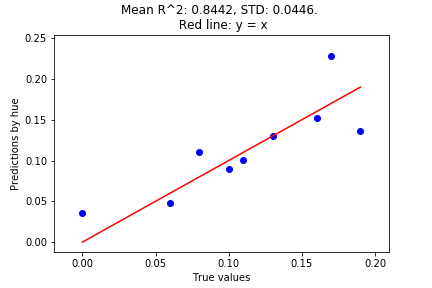

Supplement: Supplementary file 3 — op2c00216_si_005.zip [file op2c00216_si_005.zip › Collected spreadsheet outputs/Scheme 15 - statistical analysis/50 RPM/cross validation/hue_CV_LOO.png]

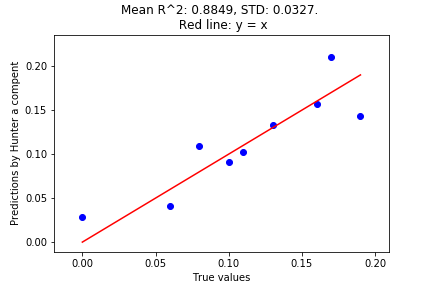

Supplement: Supplementary file 3 — op2c00216_si_005.zip [file op2c00216_si_005.zip › Collected spreadsheet outputs/Scheme 15 - statistical analysis/50 RPM/cross validation/Hunter a compent_CV_LOO.png]
