# Supplementary figures and images for: Computer Vision for Kinetic Analysis of Lab- and Process-Scale Mixing Phenomena (part 1 of 2)
Source: Org Process Res Dev. 2022 Nov 4;26(11):3073–88. doi: 10.1021/acs.oprd.2c00216 (PMC9680030; doi:10.1021/acs.oprd.2c00216)

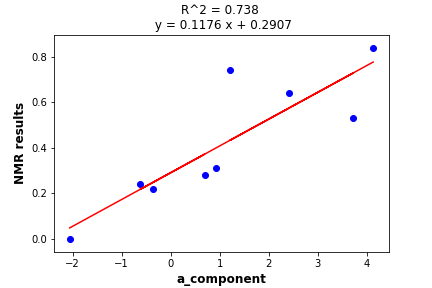

Supplement: Supplementary file 3 — op2c00216_si_005.zip [file op2c00216_si_005.zip › Collected spreadsheet outputs/Scheme 15 - statistical analysis/200 RPM - full reactor analysis/Correlation_a_component.png]

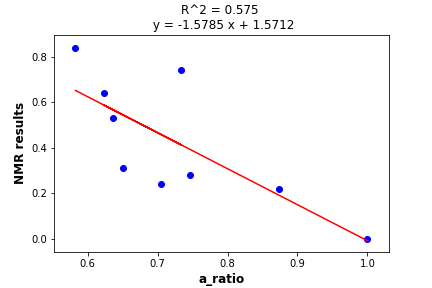

Supplement: Supplementary file 3 — op2c00216_si_005.zip [file op2c00216_si_005.zip › Collected spreadsheet outputs/Scheme 15 - statistical analysis/200 RPM - full reactor analysis/Correlation_a_ratio.png]

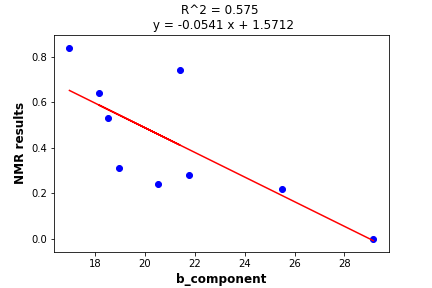

Supplement: Supplementary file 3 — op2c00216_si_005.zip [file op2c00216_si_005.zip › Collected spreadsheet outputs/Scheme 15 - statistical analysis/200 RPM - full reactor analysis/Correlation_b_component.png]

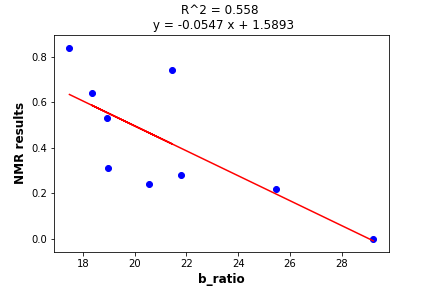

Supplement: Supplementary file 3 — op2c00216_si_005.zip [file op2c00216_si_005.zip › Collected spreadsheet outputs/Scheme 15 - statistical analysis/200 RPM - full reactor analysis/Correlation_b_ratio.png]

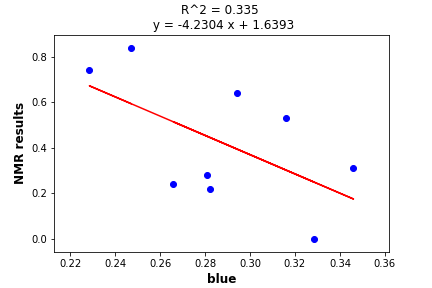

Supplement: Supplementary file 3 — op2c00216_si_005.zip [file op2c00216_si_005.zip › Collected spreadsheet outputs/Scheme 15 - statistical analysis/200 RPM - full reactor analysis/Correlation_blue.png]

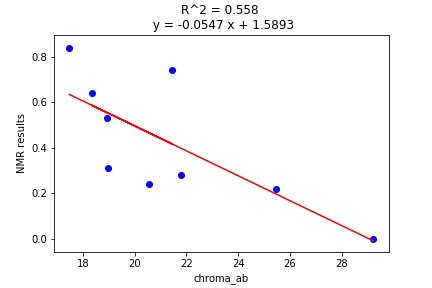

Supplement: Supplementary file 3 — op2c00216_si_005.zip [file op2c00216_si_005.zip › Collected spreadsheet outputs/Scheme 15 - statistical analysis/200 RPM - full reactor analysis/Correlation_chroma_ab.png]

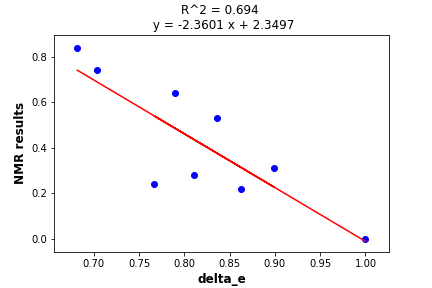

Supplement: Supplementary file 3 — op2c00216_si_005.zip [file op2c00216_si_005.zip › Collected spreadsheet outputs/Scheme 15 - statistical analysis/200 RPM - full reactor analysis/Correlation_delta_e.png]

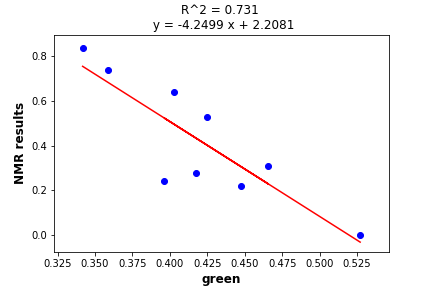

Supplement: Supplementary file 3 — op2c00216_si_005.zip [file op2c00216_si_005.zip › Collected spreadsheet outputs/Scheme 15 - statistical analysis/200 RPM - full reactor analysis/Correlation_green.png]

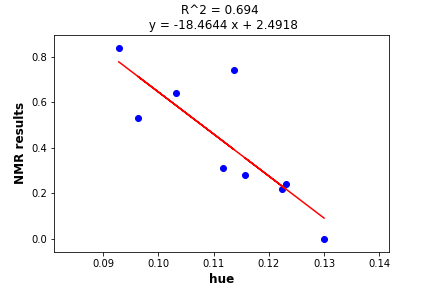

Supplement: Supplementary file 3 — op2c00216_si_005.zip [file op2c00216_si_005.zip › Collected spreadsheet outputs/Scheme 15 - statistical analysis/200 RPM - full reactor analysis/Correlation_hue.png]

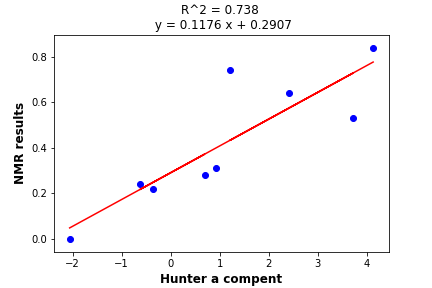

Supplement: Supplementary file 3 — op2c00216_si_005.zip [file op2c00216_si_005.zip › Collected spreadsheet outputs/Scheme 15 - statistical analysis/200 RPM - full reactor analysis/Correlation_Hunter a compent.png]

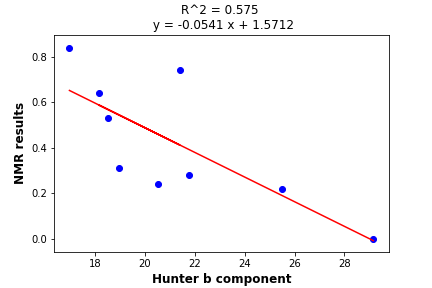

Supplement: Supplementary file 3 — op2c00216_si_005.zip [file op2c00216_si_005.zip › Collected spreadsheet outputs/Scheme 15 - statistical analysis/200 RPM - full reactor analysis/Correlation_Hunter b component.png]

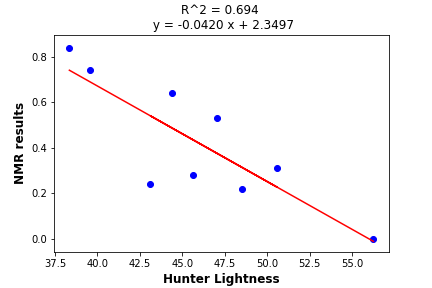

Supplement: Supplementary file 3 — op2c00216_si_005.zip [file op2c00216_si_005.zip › Collected spreadsheet outputs/Scheme 15 - statistical analysis/200 RPM - full reactor analysis/Correlation_Hunter Lightness.png]

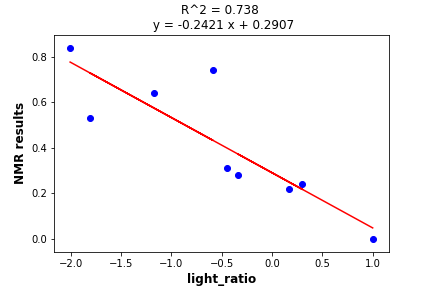

Supplement: Supplementary file 3 — op2c00216_si_005.zip [file op2c00216_si_005.zip › Collected spreadsheet outputs/Scheme 15 - statistical analysis/200 RPM - full reactor analysis/Correlation_light_ratio.png]

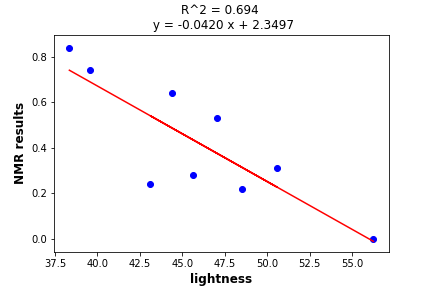

Supplement: Supplementary file 3 — op2c00216_si_005.zip [file op2c00216_si_005.zip › Collected spreadsheet outputs/Scheme 15 - statistical analysis/200 RPM - full reactor analysis/Correlation_lightness.png]

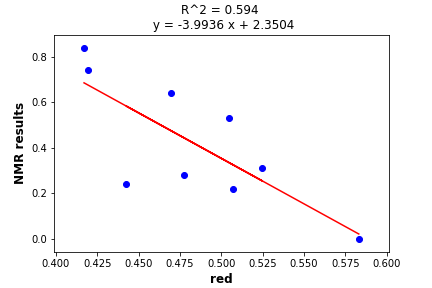

Supplement: Supplementary file 3 — op2c00216_si_005.zip [file op2c00216_si_005.zip › Collected spreadsheet outputs/Scheme 15 - statistical analysis/200 RPM - full reactor analysis/Correlation_red.png]

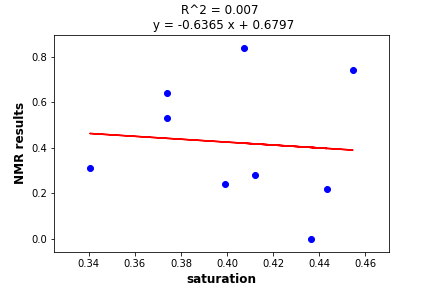

Supplement: Supplementary file 3 — op2c00216_si_005.zip [file op2c00216_si_005.zip › Collected spreadsheet outputs/Scheme 15 - statistical analysis/200 RPM - full reactor analysis/Correlation_saturation.png]

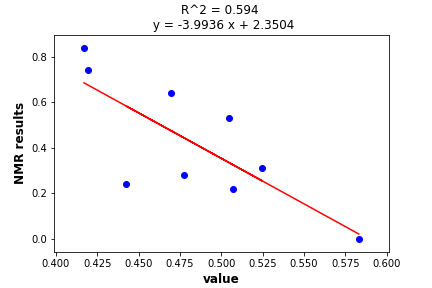

Supplement: Supplementary file 3 — op2c00216_si_005.zip [file op2c00216_si_005.zip › Collected spreadsheet outputs/Scheme 15 - statistical analysis/200 RPM - full reactor analysis/Correlation_value.png]

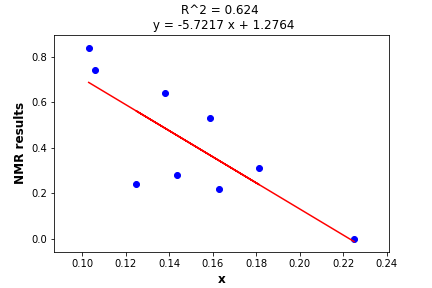

Supplement: Supplementary file 3 — op2c00216_si_005.zip [file op2c00216_si_005.zip › Collected spreadsheet outputs/Scheme 15 - statistical analysis/200 RPM - full reactor analysis/Correlation_x.png]

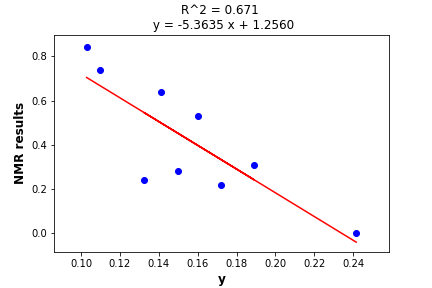

Supplement: Supplementary file 3 — op2c00216_si_005.zip [file op2c00216_si_005.zip › Collected spreadsheet outputs/Scheme 15 - statistical analysis/200 RPM - full reactor analysis/Correlation_y.png]

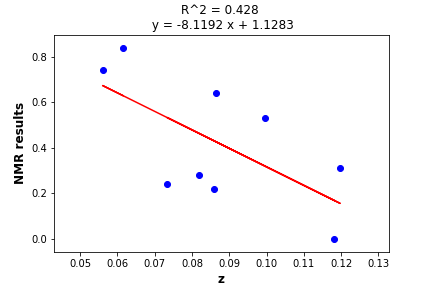

Supplement: Supplementary file 3 — op2c00216_si_005.zip [file op2c00216_si_005.zip › Collected spreadsheet outputs/Scheme 15 - statistical analysis/200 RPM - full reactor analysis/Correlation_z.png]

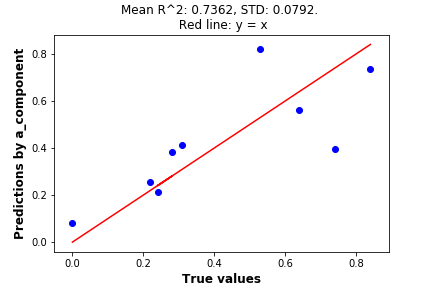

Supplement: Supplementary file 3 — op2c00216_si_005.zip [file op2c00216_si_005.zip › Collected spreadsheet outputs/Scheme 15 - statistical analysis/200 RPM - full reactor analysis/cross validation/a_component_CV_LOO.png]

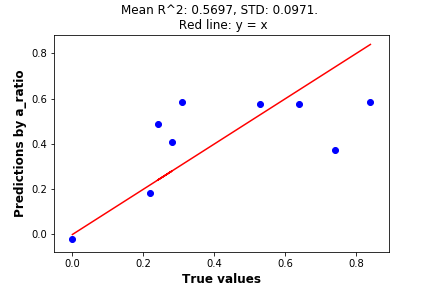

Supplement: Supplementary file 3 — op2c00216_si_005.zip [file op2c00216_si_005.zip › Collected spreadsheet outputs/Scheme 15 - statistical analysis/200 RPM - full reactor analysis/cross validation/a_ratio_CV_LOO.png]

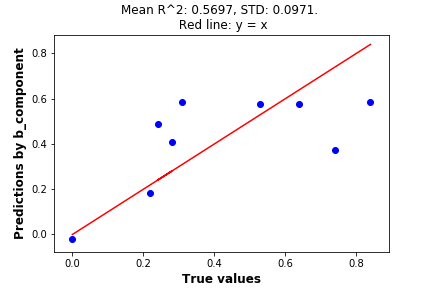

Supplement: Supplementary file 3 — op2c00216_si_005.zip [file op2c00216_si_005.zip › Collected spreadsheet outputs/Scheme 15 - statistical analysis/200 RPM - full reactor analysis/cross validation/b_component_CV_LOO.png]

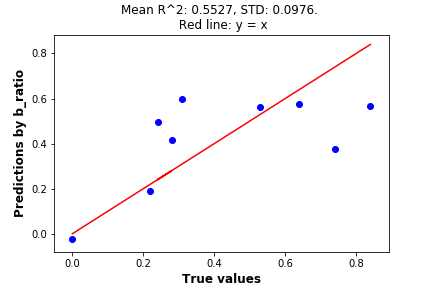

Supplement: Supplementary file 3 — op2c00216_si_005.zip [file op2c00216_si_005.zip › Collected spreadsheet outputs/Scheme 15 - statistical analysis/200 RPM - full reactor analysis/cross validation/b_ratio_CV_LOO.png]

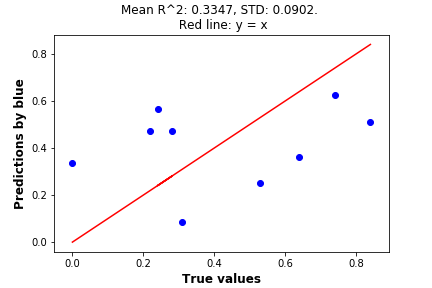

Supplement: Supplementary file 3 — op2c00216_si_005.zip [file op2c00216_si_005.zip › Collected spreadsheet outputs/Scheme 15 - statistical analysis/200 RPM - full reactor analysis/cross validation/blue_CV_LOO.png]

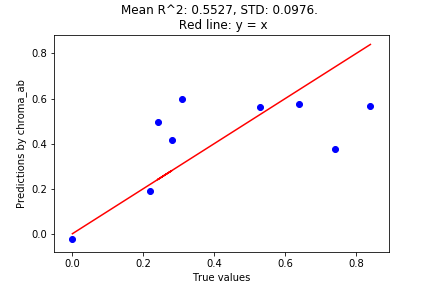

Supplement: Supplementary file 3 — op2c00216_si_005.zip [file op2c00216_si_005.zip › Collected spreadsheet outputs/Scheme 15 - statistical analysis/200 RPM - full reactor analysis/cross validation/chroma_ab_CV_LOO.png]

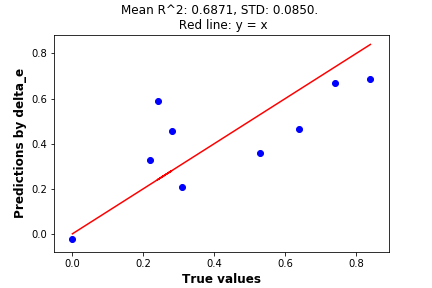

Supplement: Supplementary file 3 — op2c00216_si_005.zip [file op2c00216_si_005.zip › Collected spreadsheet outputs/Scheme 15 - statistical analysis/200 RPM - full reactor analysis/cross validation/delta_e_CV_LOO.png]

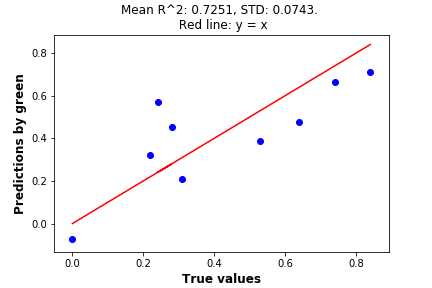

Supplement: Supplementary file 3 — op2c00216_si_005.zip [file op2c00216_si_005.zip › Collected spreadsheet outputs/Scheme 15 - statistical analysis/200 RPM - full reactor analysis/cross validation/green_CV_LOO.png]

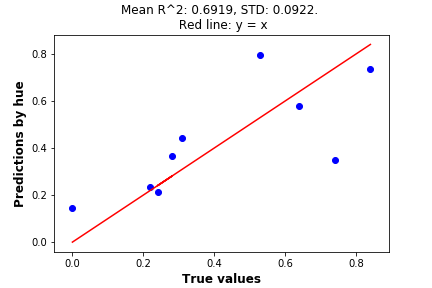

Supplement: Supplementary file 3 — op2c00216_si_005.zip [file op2c00216_si_005.zip › Collected spreadsheet outputs/Scheme 15 - statistical analysis/200 RPM - full reactor analysis/cross validation/hue_CV_LOO.png]

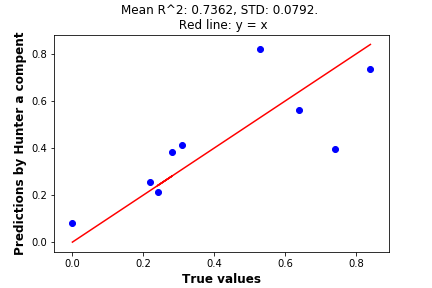

Supplement: Supplementary file 3 — op2c00216_si_005.zip [file op2c00216_si_005.zip › Collected spreadsheet outputs/Scheme 15 - statistical analysis/200 RPM - full reactor analysis/cross validation/Hunter a compent_CV_LOO.png]

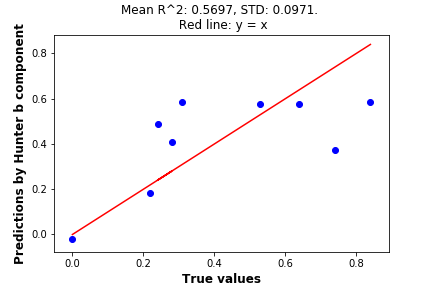

Supplement: Supplementary file 3 — op2c00216_si_005.zip [file op2c00216_si_005.zip › Collected spreadsheet outputs/Scheme 15 - statistical analysis/200 RPM - full reactor analysis/cross validation/Hunter b component_CV_LOO.png]

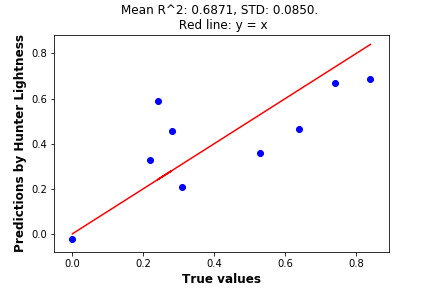

Supplement: Supplementary file 3 — op2c00216_si_005.zip [file op2c00216_si_005.zip › Collected spreadsheet outputs/Scheme 15 - statistical analysis/200 RPM - full reactor analysis/cross validation/Hunter Lightness_CV_LOO.png]

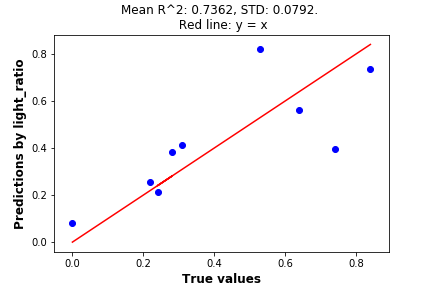

Supplement: Supplementary file 3 — op2c00216_si_005.zip [file op2c00216_si_005.zip › Collected spreadsheet outputs/Scheme 15 - statistical analysis/200 RPM - full reactor analysis/cross validation/light_ratio_CV_LOO.png]

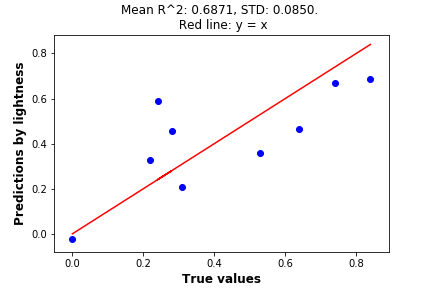

Supplement: Supplementary file 3 — op2c00216_si_005.zip [file op2c00216_si_005.zip › Collected spreadsheet outputs/Scheme 15 - statistical analysis/200 RPM - full reactor analysis/cross validation/lightness_CV_LOO.png]

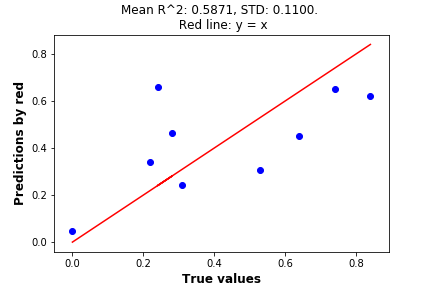

Supplement: Supplementary file 3 — op2c00216_si_005.zip [file op2c00216_si_005.zip › Collected spreadsheet outputs/Scheme 15 - statistical analysis/200 RPM - full reactor analysis/cross validation/red_CV_LOO.png]

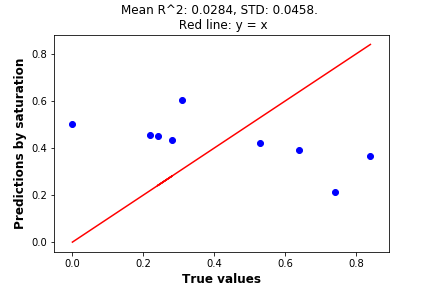

Supplement: Supplementary file 3 — op2c00216_si_005.zip [file op2c00216_si_005.zip › Collected spreadsheet outputs/Scheme 15 - statistical analysis/200 RPM - full reactor analysis/cross validation/saturation_CV_LOO.png]

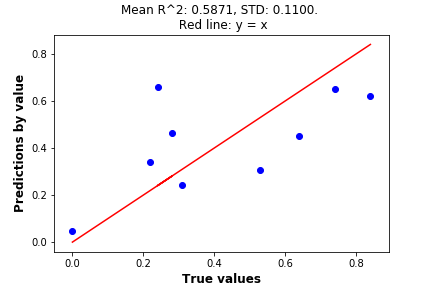

Supplement: Supplementary file 3 — op2c00216_si_005.zip [file op2c00216_si_005.zip › Collected spreadsheet outputs/Scheme 15 - statistical analysis/200 RPM - full reactor analysis/cross validation/value_CV_LOO.png]

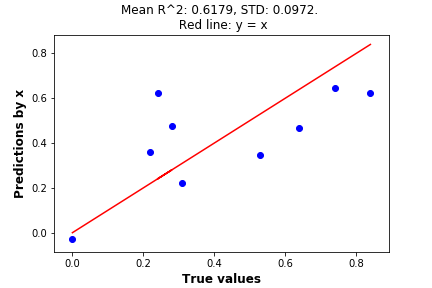

Supplement: Supplementary file 3 — op2c00216_si_005.zip [file op2c00216_si_005.zip › Collected spreadsheet outputs/Scheme 15 - statistical analysis/200 RPM - full reactor analysis/cross validation/x_CV_LOO.png]

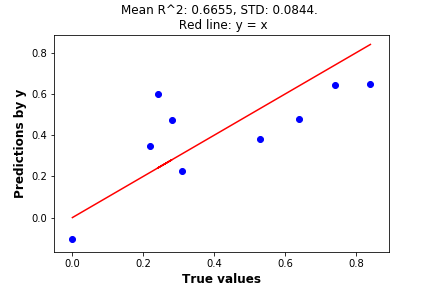

Supplement: Supplementary file 3 — op2c00216_si_005.zip [file op2c00216_si_005.zip › Collected spreadsheet outputs/Scheme 15 - statistical analysis/200 RPM - full reactor analysis/cross validation/y_CV_LOO.png]

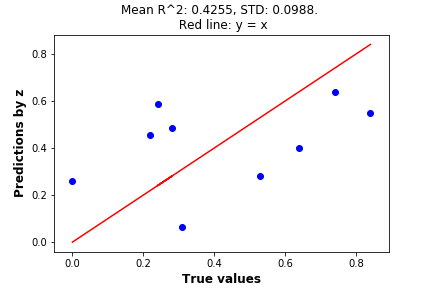

Supplement: Supplementary file 3 — op2c00216_si_005.zip [file op2c00216_si_005.zip › Collected spreadsheet outputs/Scheme 15 - statistical analysis/200 RPM - full reactor analysis/cross validation/z_CV_LOO.png]

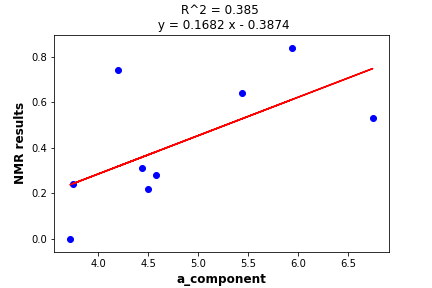

Supplement: Supplementary file 3 — op2c00216_si_005.zip [file op2c00216_si_005.zip › Collected spreadsheet outputs/Scheme 15 - statistical analysis/200 RPM - top layer analysis/Correlation_a_component.png]

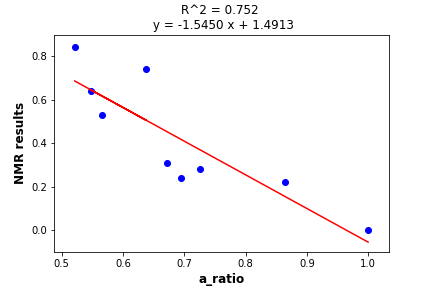

Supplement: Supplementary file 3 — op2c00216_si_005.zip [file op2c00216_si_005.zip › Collected spreadsheet outputs/Scheme 15 - statistical analysis/200 RPM - top layer analysis/Correlation_a_ratio.png]

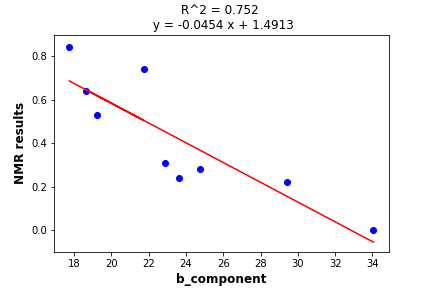

Supplement: Supplementary file 3 — op2c00216_si_005.zip [file op2c00216_si_005.zip › Collected spreadsheet outputs/Scheme 15 - statistical analysis/200 RPM - top layer analysis/Correlation_b_component.png]

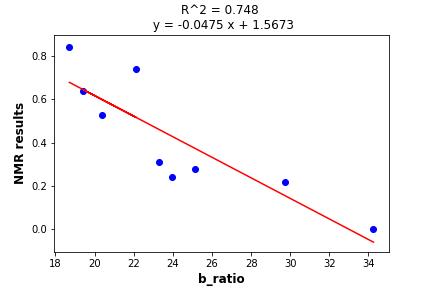

Supplement: Supplementary file 3 — op2c00216_si_005.zip [file op2c00216_si_005.zip › Collected spreadsheet outputs/Scheme 15 - statistical analysis/200 RPM - top layer analysis/Correlation_b_ratio.png]

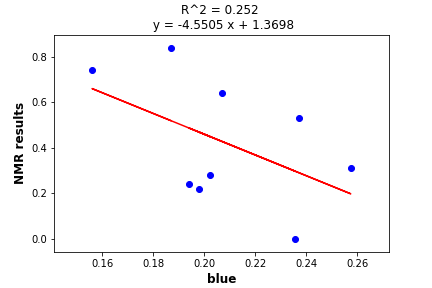

Supplement: Supplementary file 3 — op2c00216_si_005.zip [file op2c00216_si_005.zip › Collected spreadsheet outputs/Scheme 15 - statistical analysis/200 RPM - top layer analysis/Correlation_blue.png]

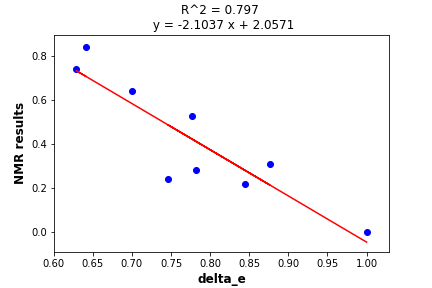

Supplement: Supplementary file 3 — op2c00216_si_005.zip [file op2c00216_si_005.zip › Collected spreadsheet outputs/Scheme 15 - statistical analysis/200 RPM - top layer analysis/Correlation_delta_e.png]

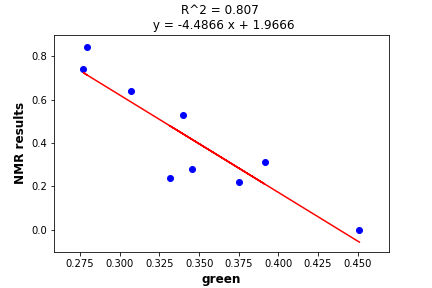

Supplement: Supplementary file 3 — op2c00216_si_005.zip [file op2c00216_si_005.zip › Collected spreadsheet outputs/Scheme 15 - statistical analysis/200 RPM - top layer analysis/Correlation_green.png]

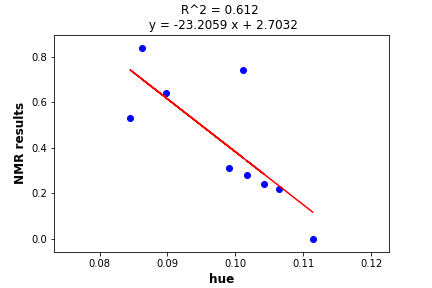

Supplement: Supplementary file 3 — op2c00216_si_005.zip [file op2c00216_si_005.zip › Collected spreadsheet outputs/Scheme 15 - statistical analysis/200 RPM - top layer analysis/Correlation_hue.png]

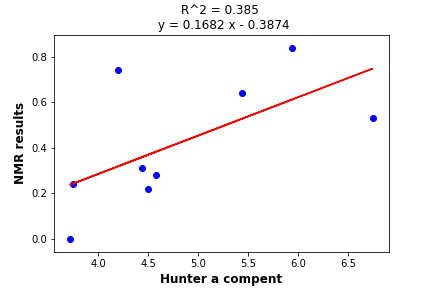

Supplement: Supplementary file 3 — op2c00216_si_005.zip [file op2c00216_si_005.zip › Collected spreadsheet outputs/Scheme 15 - statistical analysis/200 RPM - top layer analysis/Correlation_Hunter a compent.png]

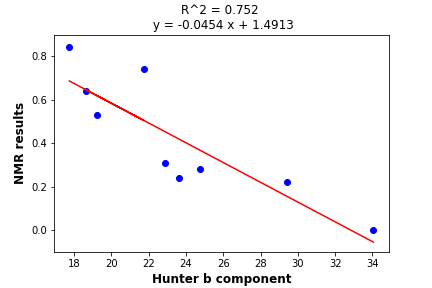

Supplement: Supplementary file 3 — op2c00216_si_005.zip [file op2c00216_si_005.zip › Collected spreadsheet outputs/Scheme 15 - statistical analysis/200 RPM - top layer analysis/Correlation_Hunter b component.png]

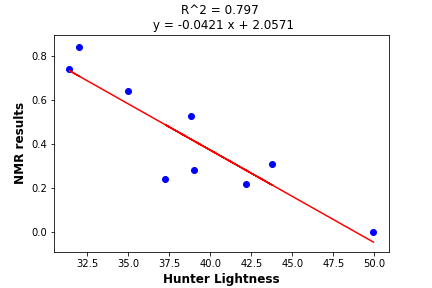

Supplement: Supplementary file 3 — op2c00216_si_005.zip [file op2c00216_si_005.zip › Collected spreadsheet outputs/Scheme 15 - statistical analysis/200 RPM - top layer analysis/Correlation_Hunter Lightness.png]

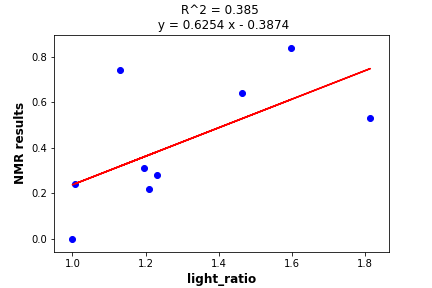

Supplement: Supplementary file 3 — op2c00216_si_005.zip [file op2c00216_si_005.zip › Collected spreadsheet outputs/Scheme 15 - statistical analysis/200 RPM - top layer analysis/Correlation_light_ratio.png]

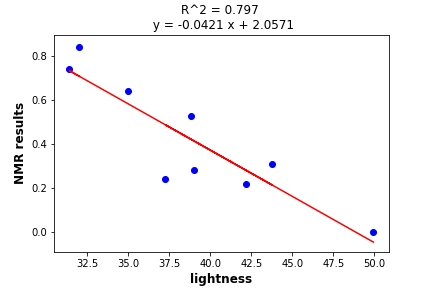

Supplement: Supplementary file 3 — op2c00216_si_005.zip [file op2c00216_si_005.zip › Collected spreadsheet outputs/Scheme 15 - statistical analysis/200 RPM - top layer analysis/Correlation_lightness.png]

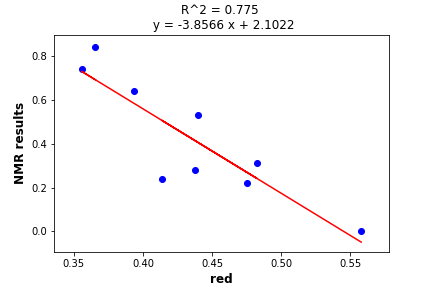

Supplement: Supplementary file 3 — op2c00216_si_005.zip [file op2c00216_si_005.zip › Collected spreadsheet outputs/Scheme 15 - statistical analysis/200 RPM - top layer analysis/Correlation_red.png]

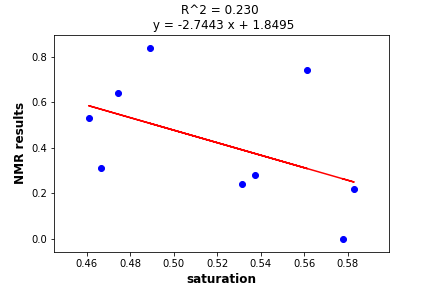

Supplement: Supplementary file 3 — op2c00216_si_005.zip [file op2c00216_si_005.zip › Collected spreadsheet outputs/Scheme 15 - statistical analysis/200 RPM - top layer analysis/Correlation_saturation.png]

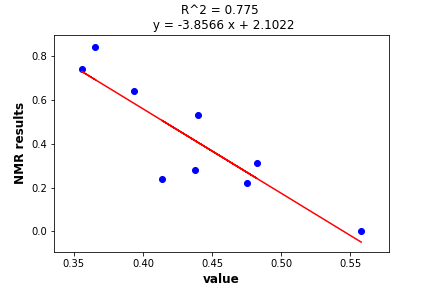

Supplement: Supplementary file 3 — op2c00216_si_005.zip [file op2c00216_si_005.zip › Collected spreadsheet outputs/Scheme 15 - statistical analysis/200 RPM - top layer analysis/Correlation_value.png]

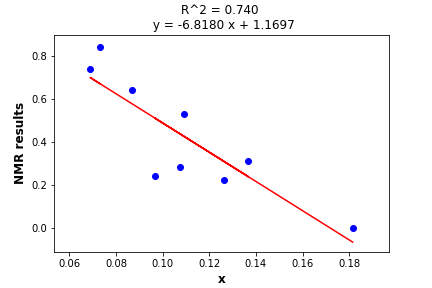

Supplement: Supplementary file 3 — op2c00216_si_005.zip [file op2c00216_si_005.zip › Collected spreadsheet outputs/Scheme 15 - statistical analysis/200 RPM - top layer analysis/Correlation_x.png]

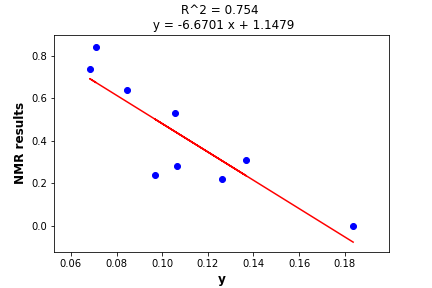

Supplement: Supplementary file 3 — op2c00216_si_005.zip [file op2c00216_si_005.zip › Collected spreadsheet outputs/Scheme 15 - statistical analysis/200 RPM - top layer analysis/Correlation_y.png]

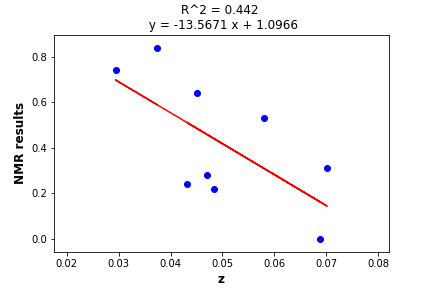

Supplement: Supplementary file 3 — op2c00216_si_005.zip [file op2c00216_si_005.zip › Collected spreadsheet outputs/Scheme 15 - statistical analysis/200 RPM - top layer analysis/Correlation_z.png]

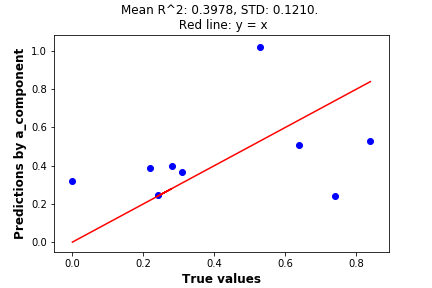

Supplement: Supplementary file 3 — op2c00216_si_005.zip [file op2c00216_si_005.zip › Collected spreadsheet outputs/Scheme 15 - statistical analysis/200 RPM - top layer analysis/cross validation/a_component_CV_LOO.png]

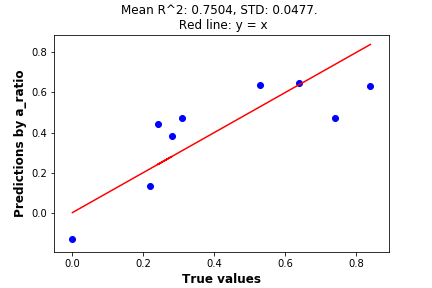

Supplement: Supplementary file 3 — op2c00216_si_005.zip [file op2c00216_si_005.zip › Collected spreadsheet outputs/Scheme 15 - statistical analysis/200 RPM - top layer analysis/cross validation/a_ratio_CV_LOO.png]

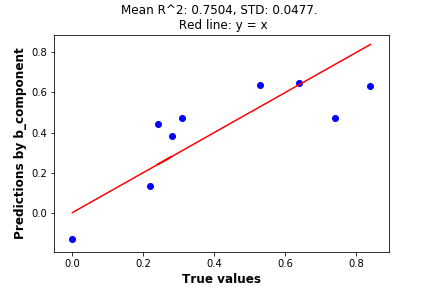

Supplement: Supplementary file 3 — op2c00216_si_005.zip [file op2c00216_si_005.zip › Collected spreadsheet outputs/Scheme 15 - statistical analysis/200 RPM - top layer analysis/cross validation/b_component_CV_LOO.png]

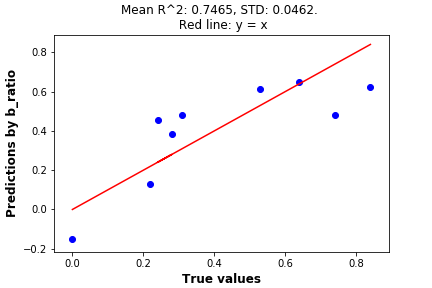

Supplement: Supplementary file 3 — op2c00216_si_005.zip [file op2c00216_si_005.zip › Collected spreadsheet outputs/Scheme 15 - statistical analysis/200 RPM - top layer analysis/cross validation/b_ratio_CV_LOO.png]

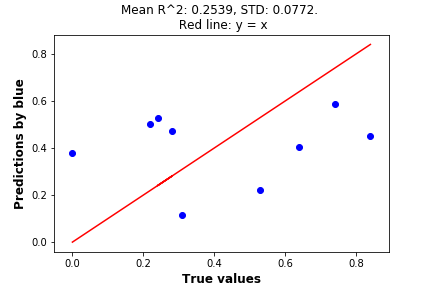

Supplement: Supplementary file 3 — op2c00216_si_005.zip [file op2c00216_si_005.zip › Collected spreadsheet outputs/Scheme 15 - statistical analysis/200 RPM - top layer analysis/cross validation/blue_CV_LOO.png]

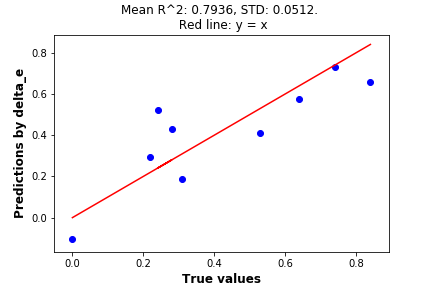

Supplement: Supplementary file 3 — op2c00216_si_005.zip [file op2c00216_si_005.zip › Collected spreadsheet outputs/Scheme 15 - statistical analysis/200 RPM - top layer analysis/cross validation/delta_e_CV_LOO.png]

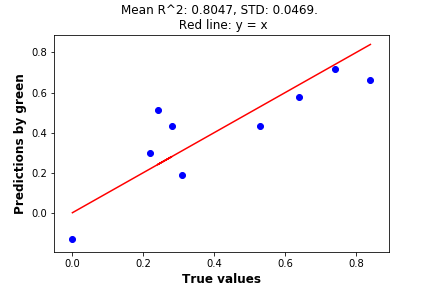

Supplement: Supplementary file 3 — op2c00216_si_005.zip [file op2c00216_si_005.zip › Collected spreadsheet outputs/Scheme 15 - statistical analysis/200 RPM - top layer analysis/cross validation/green_CV_LOO.png]

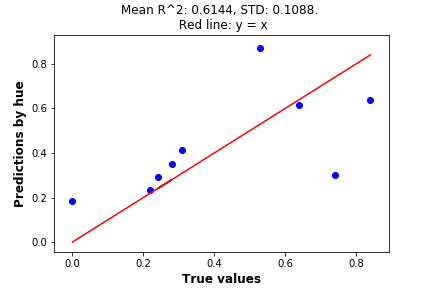

Supplement: Supplementary file 3 — op2c00216_si_005.zip [file op2c00216_si_005.zip › Collected spreadsheet outputs/Scheme 15 - statistical analysis/200 RPM - top layer analysis/cross validation/hue_CV_LOO.png]

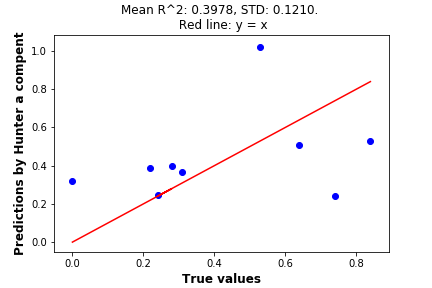

Supplement: Supplementary file 3 — op2c00216_si_005.zip [file op2c00216_si_005.zip › Collected spreadsheet outputs/Scheme 15 - statistical analysis/200 RPM - top layer analysis/cross validation/Hunter a compent_CV_LOO.png]

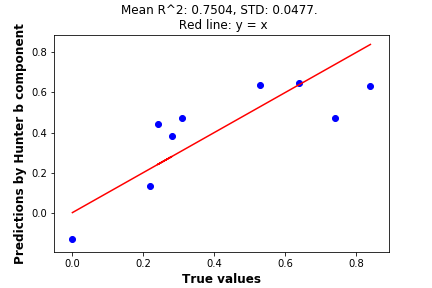

Supplement: Supplementary file 3 — op2c00216_si_005.zip [file op2c00216_si_005.zip › Collected spreadsheet outputs/Scheme 15 - statistical analysis/200 RPM - top layer analysis/cross validation/Hunter b component_CV_LOO.png]

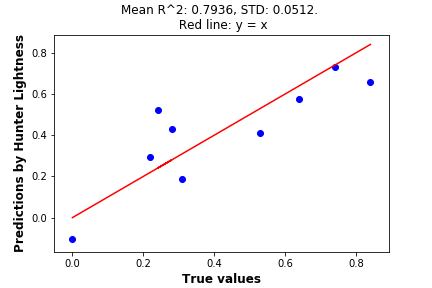

Supplement: Supplementary file 3 — op2c00216_si_005.zip [file op2c00216_si_005.zip › Collected spreadsheet outputs/Scheme 15 - statistical analysis/200 RPM - top layer analysis/cross validation/Hunter Lightness_CV_LOO.png]

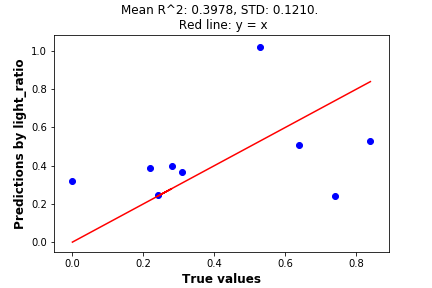

Supplement: Supplementary file 3 — op2c00216_si_005.zip [file op2c00216_si_005.zip › Collected spreadsheet outputs/Scheme 15 - statistical analysis/200 RPM - top layer analysis/cross validation/light_ratio_CV_LOO.png]

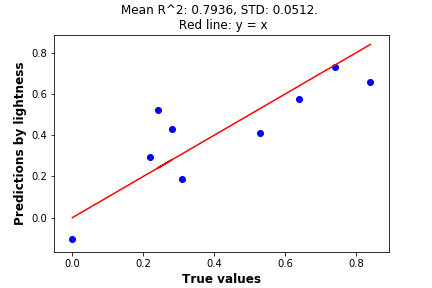

Supplement: Supplementary file 3 — op2c00216_si_005.zip [file op2c00216_si_005.zip › Collected spreadsheet outputs/Scheme 15 - statistical analysis/200 RPM - top layer analysis/cross validation/lightness_CV_LOO.png]

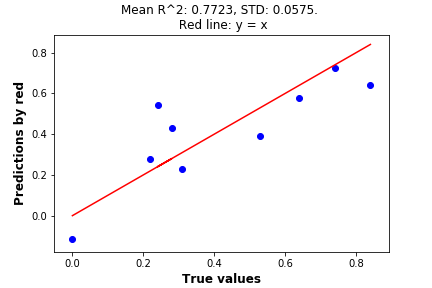

Supplement: Supplementary file 3 — op2c00216_si_005.zip [file op2c00216_si_005.zip › Collected spreadsheet outputs/Scheme 15 - statistical analysis/200 RPM - top layer analysis/cross validation/red_CV_LOO.png]

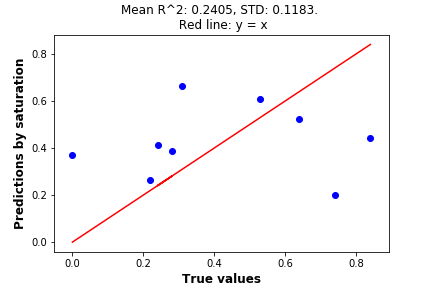

Supplement: Supplementary file 3 — op2c00216_si_005.zip [file op2c00216_si_005.zip › Collected spreadsheet outputs/Scheme 15 - statistical analysis/200 RPM - top layer analysis/cross validation/saturation_CV_LOO.png]

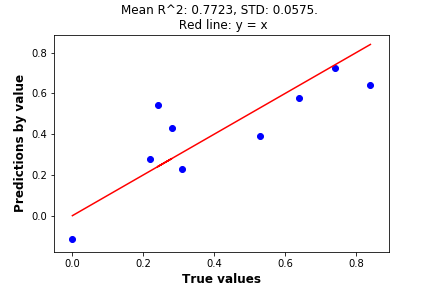

Supplement: Supplementary file 3 — op2c00216_si_005.zip [file op2c00216_si_005.zip › Collected spreadsheet outputs/Scheme 15 - statistical analysis/200 RPM - top layer analysis/cross validation/value_CV_LOO.png]

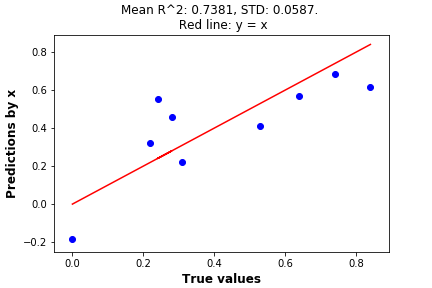

Supplement: Supplementary file 3 — op2c00216_si_005.zip [file op2c00216_si_005.zip › Collected spreadsheet outputs/Scheme 15 - statistical analysis/200 RPM - top layer analysis/cross validation/x_CV_LOO.png]

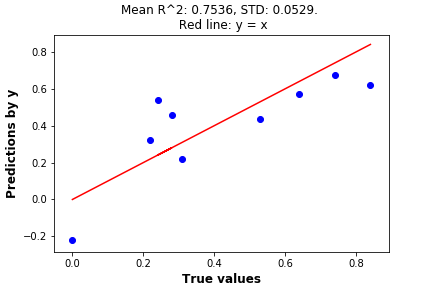

Supplement: Supplementary file 3 — op2c00216_si_005.zip [file op2c00216_si_005.zip › Collected spreadsheet outputs/Scheme 15 - statistical analysis/200 RPM - top layer analysis/cross validation/y_CV_LOO.png]

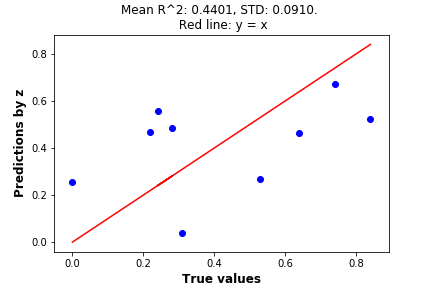

Supplement: Supplementary file 3 — op2c00216_si_005.zip [file op2c00216_si_005.zip › Collected spreadsheet outputs/Scheme 15 - statistical analysis/200 RPM - top layer analysis/cross validation/z_CV_LOO.png]

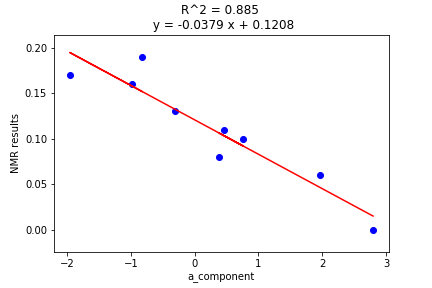

Supplement: Supplementary file 3 — op2c00216_si_005.zip [file op2c00216_si_005.zip › Collected spreadsheet outputs/Scheme 15 - statistical analysis/50 RPM/Correlation_a_component.png]

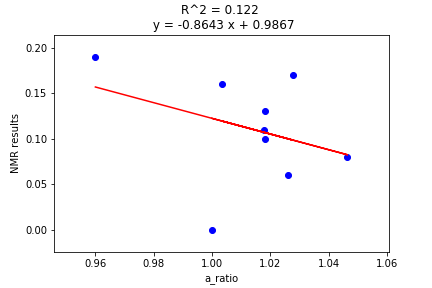

Supplement: Supplementary file 3 — op2c00216_si_005.zip [file op2c00216_si_005.zip › Collected spreadsheet outputs/Scheme 15 - statistical analysis/50 RPM/Correlation_a_ratio.png]

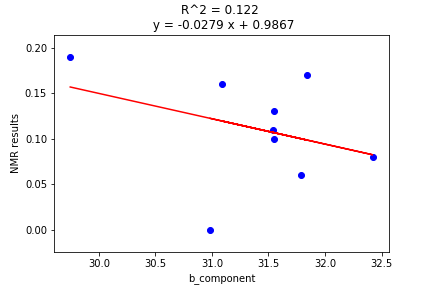

Supplement: Supplementary file 3 — op2c00216_si_005.zip [file op2c00216_si_005.zip › Collected spreadsheet outputs/Scheme 15 - statistical analysis/50 RPM/Correlation_b_component.png]

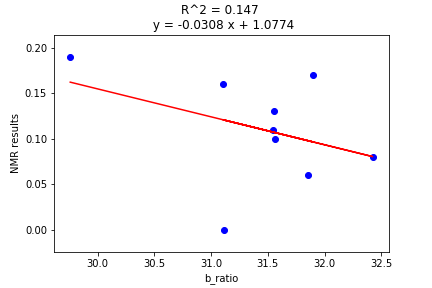

Supplement: Supplementary file 3 — op2c00216_si_005.zip [file op2c00216_si_005.zip › Collected spreadsheet outputs/Scheme 15 - statistical analysis/50 RPM/Correlation_b_ratio.png]

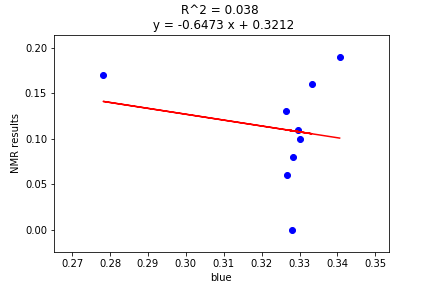

Supplement: Supplementary file 3 — op2c00216_si_005.zip [file op2c00216_si_005.zip › Collected spreadsheet outputs/Scheme 15 - statistical analysis/50 RPM/Correlation_blue.png]

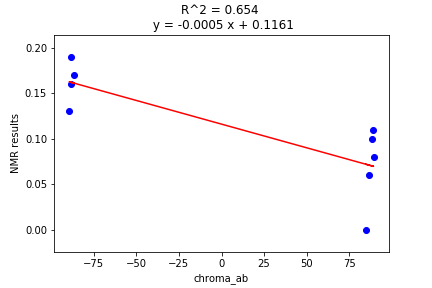

Supplement: Supplementary file 3 — op2c00216_si_005.zip [file op2c00216_si_005.zip › Collected spreadsheet outputs/Scheme 15 - statistical analysis/50 RPM/Correlation_chroma_ab.png]

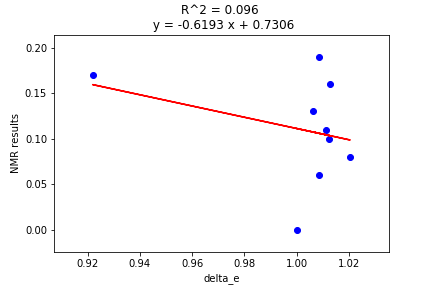

Supplement: Supplementary file 3 — op2c00216_si_005.zip [file op2c00216_si_005.zip › Collected spreadsheet outputs/Scheme 15 - statistical analysis/50 RPM/Correlation_delta_e.png]

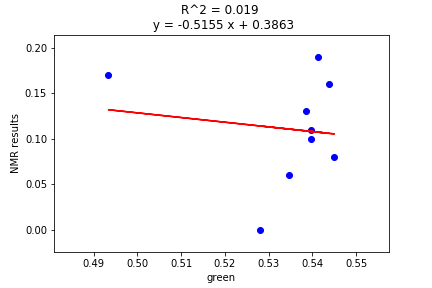

Supplement: Supplementary file 3 — op2c00216_si_005.zip [file op2c00216_si_005.zip › Collected spreadsheet outputs/Scheme 15 - statistical analysis/50 RPM/Correlation_green.png]

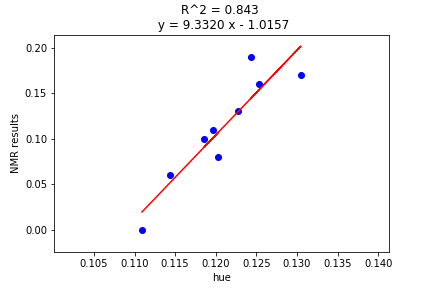

Supplement: Supplementary file 3 — op2c00216_si_005.zip [file op2c00216_si_005.zip › Collected spreadsheet outputs/Scheme 15 - statistical analysis/50 RPM/Correlation_hue.png]

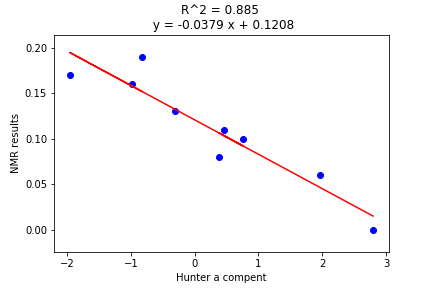

Supplement: Supplementary file 3 — op2c00216_si_005.zip [file op2c00216_si_005.zip › Collected spreadsheet outputs/Scheme 15 - statistical analysis/50 RPM/Correlation_Hunter a compent.png]

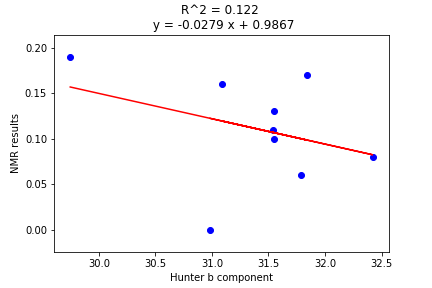

Supplement: Supplementary file 3 — op2c00216_si_005.zip [file op2c00216_si_005.zip › Collected spreadsheet outputs/Scheme 15 - statistical analysis/50 RPM/Correlation_Hunter b component.png]

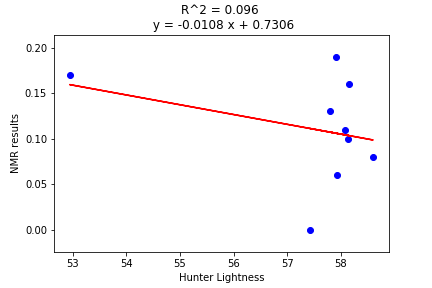

Supplement: Supplementary file 3 — op2c00216_si_005.zip [file op2c00216_si_005.zip › Collected spreadsheet outputs/Scheme 15 - statistical analysis/50 RPM/Correlation_Hunter Lightness.png]

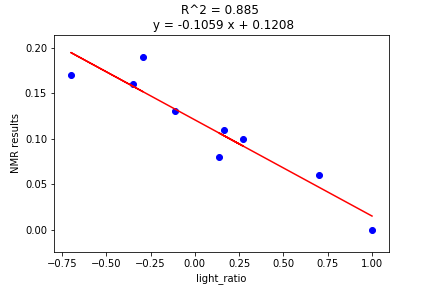

Supplement: Supplementary file 3 — op2c00216_si_005.zip [file op2c00216_si_005.zip › Collected spreadsheet outputs/Scheme 15 - statistical analysis/50 RPM/Correlation_light_ratio.png]

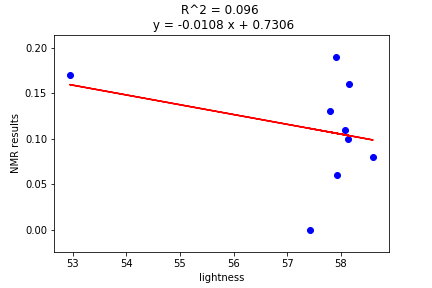

Supplement: Supplementary file 3 — op2c00216_si_005.zip [file op2c00216_si_005.zip › Collected spreadsheet outputs/Scheme 15 - statistical analysis/50 RPM/Correlation_lightness.png]

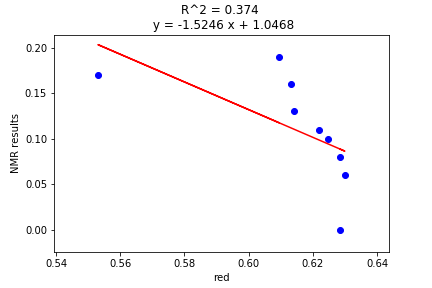

Supplement: Supplementary file 3 — op2c00216_si_005.zip [file op2c00216_si_005.zip › Collected spreadsheet outputs/Scheme 15 - statistical analysis/50 RPM/Correlation_red.png]

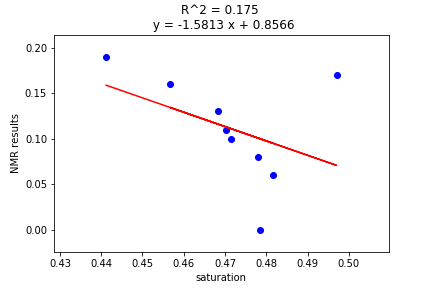

Supplement: Supplementary file 3 — op2c00216_si_005.zip [file op2c00216_si_005.zip › Collected spreadsheet outputs/Scheme 15 - statistical analysis/50 RPM/Correlation_saturation.png]

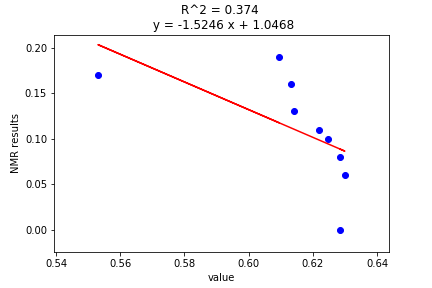

Supplement: Supplementary file 3 — op2c00216_si_005.zip [file op2c00216_si_005.zip › Collected spreadsheet outputs/Scheme 15 - statistical analysis/50 RPM/Correlation_value.png]

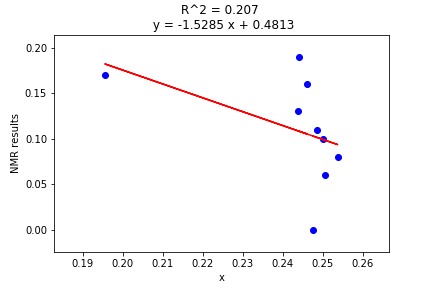

Supplement: Supplementary file 3 — op2c00216_si_005.zip [file op2c00216_si_005.zip › Collected spreadsheet outputs/Scheme 15 - statistical analysis/50 RPM/Correlation_x.png]

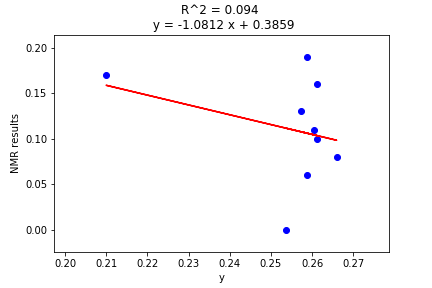

Supplement: Supplementary file 3 — op2c00216_si_005.zip [file op2c00216_si_005.zip › Collected spreadsheet outputs/Scheme 15 - statistical analysis/50 RPM/Correlation_y.png]

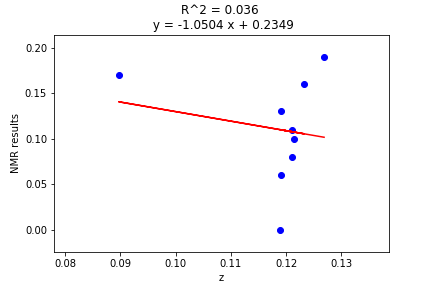

Supplement: Supplementary file 3 — op2c00216_si_005.zip [file op2c00216_si_005.zip › Collected spreadsheet outputs/Scheme 15 - statistical analysis/50 RPM/Correlation_z.png]

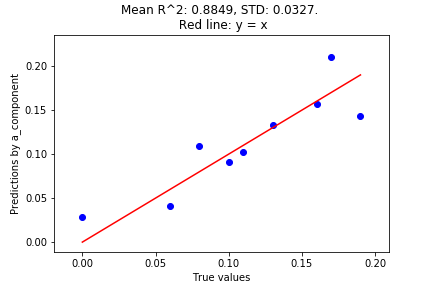

Supplement: Supplementary file 3 — op2c00216_si_005.zip [file op2c00216_si_005.zip › Collected spreadsheet outputs/Scheme 15 - statistical analysis/50 RPM/cross validation/a_component_CV_LOO.png]

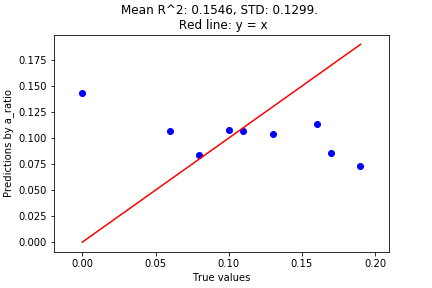

Supplement: Supplementary file 3 — op2c00216_si_005.zip [file op2c00216_si_005.zip › Collected spreadsheet outputs/Scheme 15 - statistical analysis/50 RPM/cross validation/a_ratio_CV_LOO.png]
